# Supplementary figures and images for: Ancestral SARS-CoV-2, but not Omicron, replicates less efficiently in primary pediatric nasal epithelial cells
Source: PLoS Biol. 2022 Aug 1;20(8):e3001728. doi: 10.1371/journal.pbio.3001728 (PMC9371332; doi:10.1371/journal.pbio.3001728)

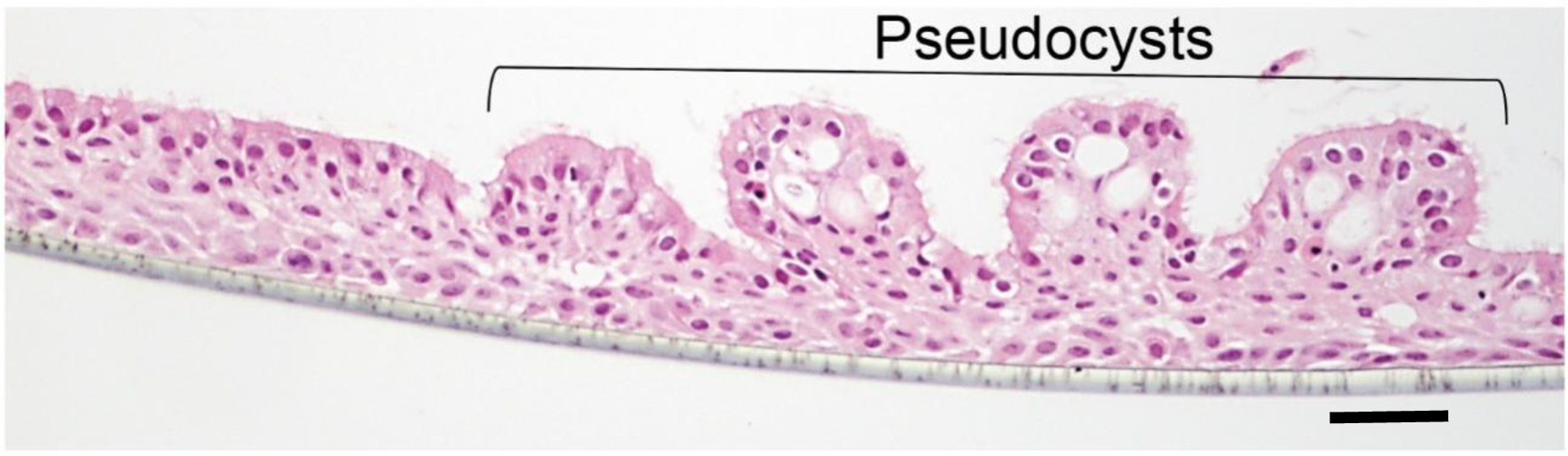

Supplement: S1 Fig — Representative HE-stained section of pediatric NECs culture differentiated at an air–liquid interface. Scale bar is equivalent of 150 μm. HE, hematoxylin–eosin; NEC, nasal epithelial cell. (TIF) [file pbio.3001728.s001.tif]

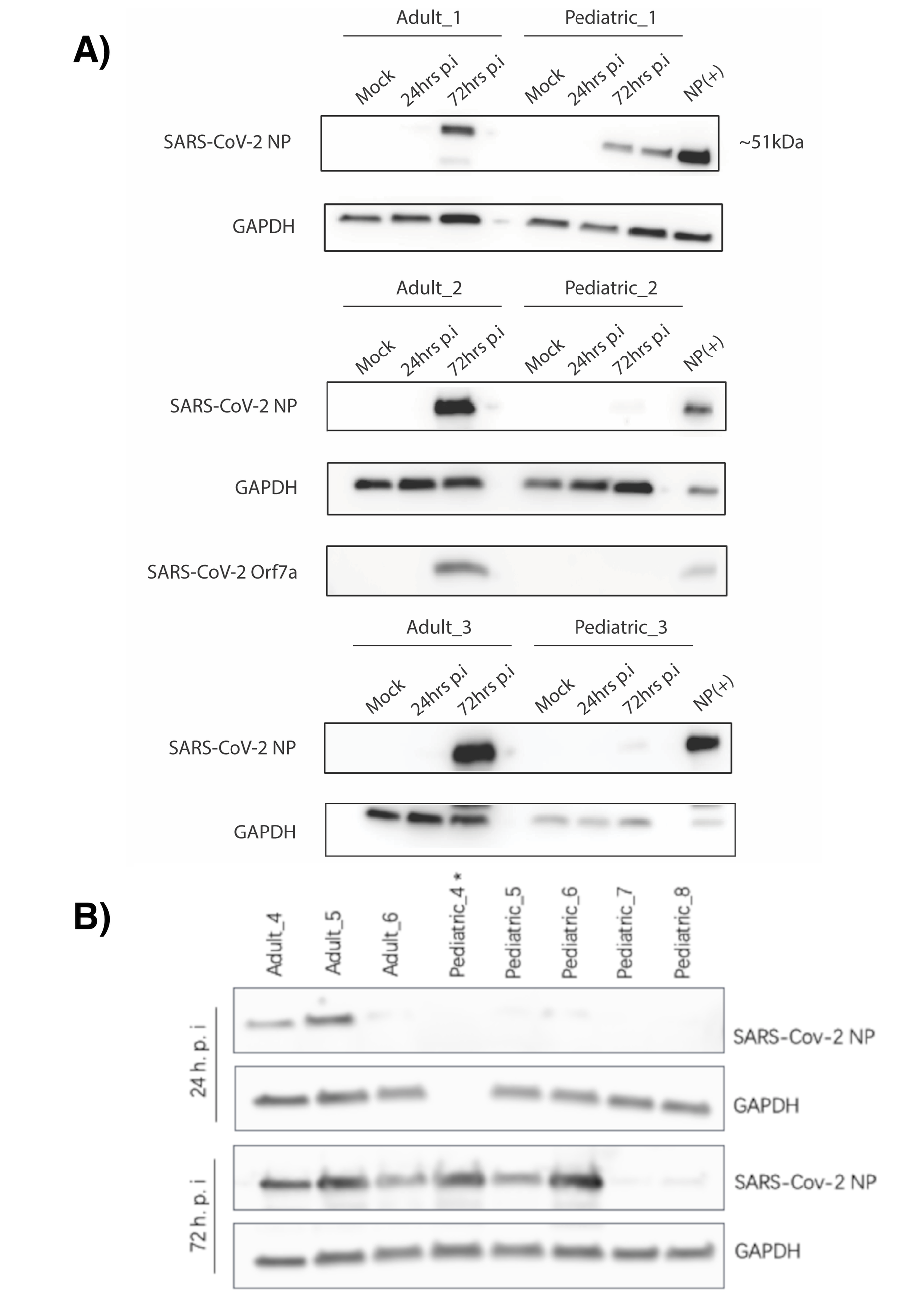

Supplement: S2 Fig — Western blot of adults and pediatric donors blotted for SARS-CoV-2 N at various time points post-infection; N = 5 adults (3 females, 2 males) and N = 8 children (4 females, 4 males). * NP levels of Pediatric donor 4 at 24 h.p.i are missing. Data are contained in S1 Data and raw western blot images are available in S1 Raw image. GAPDH, glyceraldehyde 3-phosphate dehydrogenase; h.p.i, hours post-infection; NEC, nasal epithelial cell; NP, nucleoprotein; SARS-CoV-2, Severe Acute Respiratory Syndrome Coronavirus 2. (TIF) [file pbio.3001728.s002.tif]

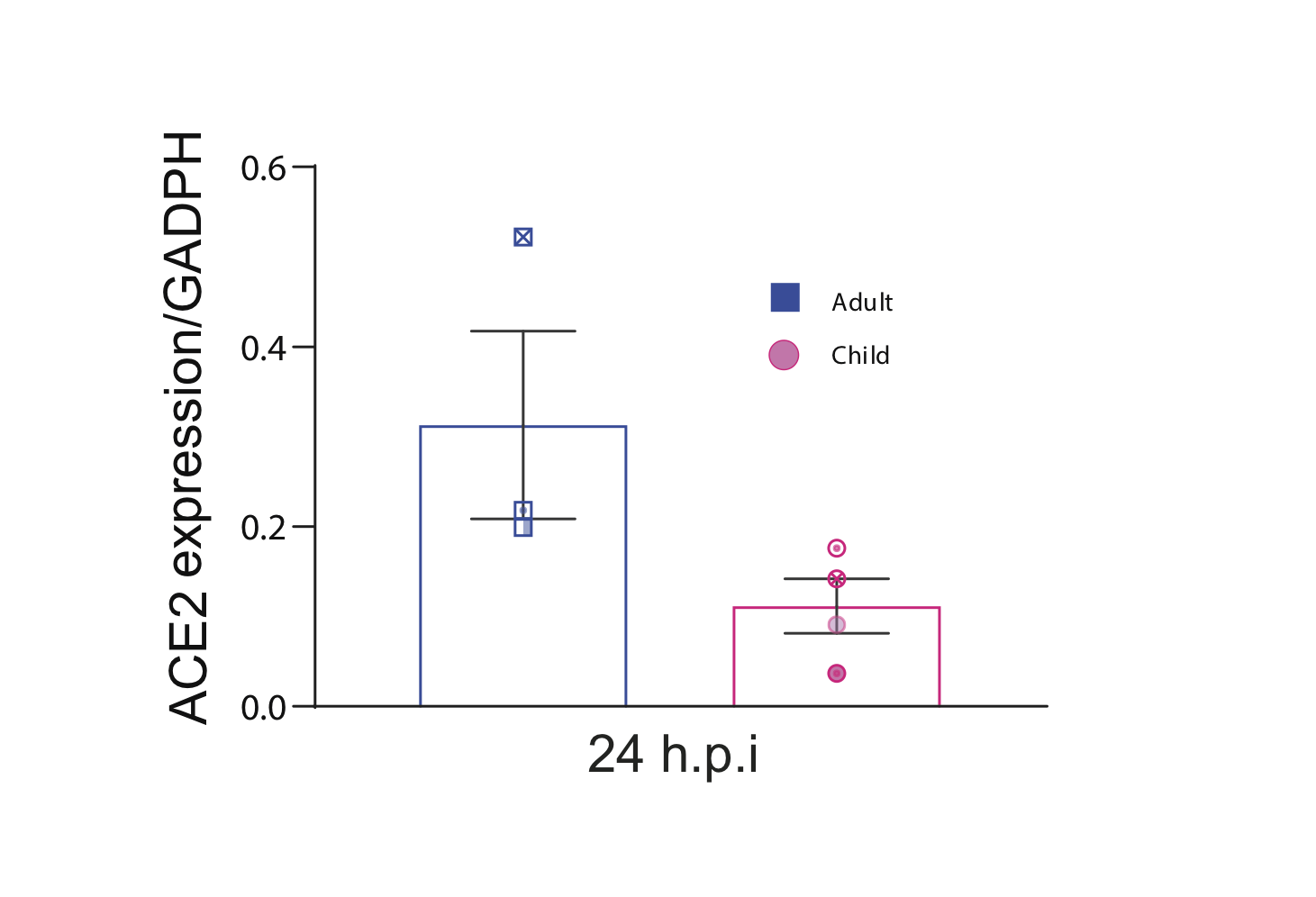

Supplement: S3 Fig — Western blot of adults and pediatric donors blotted for ACE at 24 h.p.i; N = 3 adults (1 females, 2 males) and N = 5 children (3 females, 2 males). Data are contained in S1 Data and raw western blot images are available in S1 Raw images. ACE2, angiotensin-converting enzyme 2; GAPDH, glyceraldehyde 3-phosphate dehydrogenase; h.p.i, hours post-infection; NEC, nasal epithelial cell; SARS-CoV-2, Severe Acute Respiratory Syndrome Coronavirus 2. (TIF) [file pbio.3001728.s003.tif]
