# Supplementary material for: Ancestral SARS-CoV-2, but not Omicron, replicates less efficiently in primary pediatric nasal epithelial cells
Source: PLoS Biol. 2022 Aug 1;20(8):e3001728. doi: 10.1371/journal.pbio.3001728 (PMC9371332; doi:10.1371/journal.pbio.3001728)
Supplement: S1 Table — DEGs were identified using DESeq2, genes with adjusted p-value less than 0.05 value were considered significant. DEG, differentially expressed gene; h.p.i, hours post-infection; NEC, nasal epithelial cell. (DOCX) [file pbio.3001728.s004.docx]

**S1 Table. Differentially expressed genes (DEGs) of infected pediatric NECs (72 hours post-infection) compared to adult cells with statistics.** DEGs were identified using DESeq2, genes with adjusted p-value less than 0.05 value were considered significant.

| Genes | **baseMean** | **log2FC** | **lfcSE** | **stat** | **pvalue** | **padj** |
| --- | --- | --- | --- | --- | --- | --- |
| ADH7 | 2874.532 | -1.00421 | 0.237966 | -4.21997 | 2.44E-05 | 0.041236 |
| ENPEP | 32.35705 | 2.58263 | 0.525359 | 4.915929 | 8.84E-07 | 0.006328 |
| GPATCH4 | 51.0056 | -1.19709 | 0.284169 | -4.21259 | 2.52E-05 | 0.041236 |
| IGF2 | 24.55162 | 3.851189 | 0.793619 | 4.852693 | 1.22E-06 | 0.006328 |
| MAP3K12 | 151.4382 | -2.38054 | 0.549252 | -4.33415 | 1.46E-05 | 0.032863 |
| MMP16 | 4.66984 | -5.37427 | 1.300314 | -4.13306 | 3.58E-05 | 0.049609 |
| PCDHA11 | 14.09029 | -3.17871 | 0.710306 | -4.47513 | 7.64E-06 | 0.019601 |
| PTPRQ | 60.26947 | -2.23112 | 0.492802 | -4.52742 | 5.97E-06 | 0.017879 |
| RYR3 | 99.46414 | 5.719201 | 1.185634 | 4.82375 | 1.41E-06 | 0.006328 |
| SAA4 | 8.002679 | 4.804457 | 1.13706 | 4.225334 | 2.39E-05 | 0.041236 |
| SLC26A4 | 114.124 | 4.29726 | 1.039886 | 4.132433 | 3.59E-05 | 0.049609 |
| ZBED6CL | 109.8951 | 1.398693 | 0.308122 | 4.539415 | 5.64E-06 | 0.017879 |
| ZFP57 | 16.79348 | 6.78271 | 1.351402 | 5.019018 | 5.19E-07 | 0.006328 |
