# Supplementary material for: Ancestral SARS-CoV-2, but not Omicron, replicates less efficiently in primary pediatric nasal epithelial cells
Source: PLoS Biol. 2022 Aug 1;20(8):e3001728. doi: 10.1371/journal.pbio.3001728 (PMC9371332; doi:10.1371/journal.pbio.3001728)
Supplement: S1 Data — (PDF) [file pbio.3001728.s006.pdf]

Fig 1C. Quantification of ACE2 immunofluorescence. Each data point represents the average of five separate images taken from one donor (adult (N=4, 2 females, 2 male) and pediatric (N=3, 2 females, 1 male))

Donor ID	Age Group	ACE2	Average ACE2
A1	Adult	7.894	7.883
A1	Adult	6.989	
A1	Adult	8.014	
A1	Adult	8.107	
A1	Adult	8.41	
A2	Adult	4.757	4.484
A2	Adult	4.416	
A2	Adult	4.298	
A2	Adult	4.488	
A2	Adult	4.461	
A3	Adult	5.235	4.951
A3	Adult	5.132	
A3	Adult	4.988	
A3	Adult	4.975	
A3	Adult	4.425	
A4	Adult	3.128	3.293
A4	Adult	3.056	
A4	Adult	3.092	
A4	Adult	3.896	
A4	Adult	3.294	
K1	Child	4.164	4.313
K1	Child	5.192	
K1	Child	4.229	
K1	Child	3.735	
K1	Child	4.247	
K2	Child	3.323	3.211
K2	Child	3.121	
K2	Child	3.125	
K2	Child	3.298	
K2	Child	3.186	
K3	Child	3.351	3.591
K3	Child	3.283	
K3	Child	3.253	
K3	Child	3.886	
K3	Child	4.184	

Fig 1D. Relative ACE2 and TMPRSS2 protein levels compared to GAPDH in adult (3 females, 2 males) and pediatric (3 females, 2 males) NECs.

Donor ID	Group	ACE2	TMPRSS2
A1	Adult	0.91	0.61
A2	Adult	0.18	0.63
A3	Adult	3.48	0.45
A4	Adult	3.97	1.07
A5	Adult	0.52	0.43
K1	Child	1.32	0.73
K2	Child	0.80	0.39
K3	Child	0.21	0.96
K4	Child	0.65	0.33
K5	Child	0.53	0.66

Fig 2A. Plaque forming units (PFU) of SARS-CoV-2 (QLD02) from the apical surface of nasal epithelial cells obtained at 24, 48 and 72 h.p.i. (PFU/mL).

Donor	Adult_24hpi	Child_24hpi	Adult_48hpi	Child_48hpi	Adult_72hpi	Child_72hpi
1	16400	2200*	133733	23733	1026667	177333
2	6933	480	116000	2080	1.8072e+007*	142133
3	120	120	3427	13333	4460000*	22667
4	11060	0	48000	14000	1200000	121333
5	60	827	58800	148000*	220000	1200000
6	660	40	65000	124000*	54800	400000
7	400	40	94000	28000	78000	800000
8	4600	420	20000	NA	22000	NA
9	-	140	-	440	-	520
10	-	300	-	320	-	760

*Note: Outliers were removed using ROUT's test (Q = 1%). hpi: Hours post infection

N=8 adults: 5 females, 3 males and N=10 children: 5 females, 5 males

Replicates Plaque forming units (PFU) data for adult NECs

Donor	Adult_24hpi	Adult_48hpi	Adult_72hpi
A1	NA	13200	600000
A1	16400	208000	880000
A1	NA	180000	1600000
A2	9600	144000	144000
A2	2400	88000	24000000
A2	8800	NA	96000000
A3	NA	1240	8800000
A3	120	7600	920000
A3	NA	1440	8000000
A4	21200	8000	1200000
A4	920	88000	NA
A5	40	104000	200000
A5	80	13600	240000
A6	680	10000	5600
A6	640	120000	104000
A7	120	128000	84000
A7	680	60000	72000
A8	4000	16000	20000
A8	5200	24000	24000

Replicates Plaque forming un

Donor	Child_24hpi
K1	1600
K1	2800
K1	NA
K2	240
K2	240
K2	960
K3	0
K3	120
K3	0
K4	0
K4	0
K4	0
K5	1160
K5	640
K5	680
K6	NA
K6	40
K7	80
K7	0
K8	320
K8	520
K9	160
K9	120

K10	280
K10	320

nits (PFU) data for pediatric NECs

Child_48hpi	Child_72hpi
32000	92000
36000	40000
3200	400000
1200	400000
3600	20000
1440	6400
4000	60000
4000	7600
32000	400
1600	240000
32000	28000
8400	96000
148000	1200000
NA	NA
NA	NA
152000	240000
96000	560000
32000	1160000
24000	440000
NA	NA
NA	NA
400	480
480	560

240	480
400	1040

Fig 2C. Relative SARS-CoV-2 (QLD02) NP levels compared to GAPDH in pediatric and adult NECs.

Donor	Adult_mock	Child_mock	Adult_24hpi	Child_24hpi	Adult_72hpi	Child_72hpi
1	0	0	0.01	0	2.57	0.069
2	0	0	0.022	0	1.143	0.62
3	0	0	NA	0.002	0.96	0.695
4	NA	NA	0.43	NA*	0.81	0.78
5	NA	NA	1	*0.02**	1	0.53
6	NA	NA	0.05	*0.07**	0.45	1.08
7	-	NA	-	0	-	0.1
8	-	NA	-	0	-	0.03

*Note: Outliers were removed using ROUT's test (Q = 1%). hpi: Hours post infection

* NP levels of Pediatric donor 4 at 24 hours post-infection is missing.

Fig 2D. Expression of ORF3a RNA in infected cells at various timepoints relative to HPRT expression.

Donor	Adult_24hpi	Child_24hpi	Adult_48hpi	Child_48hpi	Adult_72hpi	Child_72hpi
1	78379.14	13880.27	72669.37	13943.49	611757.54	93776.24
2	232.97	4804.32	24296.49	309.62	56820.64	63376.7
3	2723	3404.52	5170.11	469.64	2032275.7	27254.46
4	31146.12	635.77	40595.35	653.33	532504.34	2138.61
5	694.87	1521.68	15771.68	71044.27	15191.79	164820.73

* hpi: Hours post infection
(N=5 adults: 4 females, 1 male and N = 5 pediatric donors: 2 females, 3 males).

Fig 3A. Principal component analysis for the global transcriptional response of naive pediatric and adult NECs. (N
4 females, 1 male and N = 5 pediatric donors: 2 females, 3 males).

PC1	PC2	PC3	PC4	PC5	PC6	PC7	PC8	PC9	PC10
-95327.1	-50286.2	-73285.4	-52849.4	-17037.4	-29244	26788.17	-28510.7	-15302	4.02E-10
24806.05	-9320.16	32844.03	-27386.4	5093.232	-47863.4	-20482.9	-607.882	38800.91	4.02E-10
-14047.2	42255	86554.22	-48133.8	-49977.3	6105.17	27245.92	28718.51	-5942.26	4.02E-10
-48620	-53215.5	81999.56	-17243.4	9712.024	9688.337	-47228.8	-14579.1	-25631.8	4.02E-10
-66022.5	14839.11	-44964.3	41926.24	41987.52	-31127.5	-2582.25	37123.65	-18763.8	4.02E-10
304195.4	-22091	-72742.8	-15537.6	-20283.8	12382.09	-15328.5	6677.074	-5674.7	4.02E-10
119575.4	38637.06	54379.82	1986.511	66602.26	13539.5	36020.1	-16844	1227.331	4.02E-10
3681.293	-31242.3	23959.98	124380.3	-41092.9	-4612.41	9499.833	-13812.2	3037.351	4.02E-10
-99993.1	200677.4	-49933.2	353.2514	-7428.02	23190.2	-17279.3	-10195	7129.299	4.02E-10
-128248	-130254	-38811.9	-7495.63	12424.38	47941.96	3347.687	12029.57	21119.6	4.02E-10

l=5 adults:

Age Group
Adult
Adult
Adult
Adult
Adult
Paediatric
Paediatric
Paediatric
Paediatric
Paediatric

Fig 3B. DGEs analysis of infected cells (independent of donors' age). DEGs were identified using DESeq2, genes with adjusted p-value less than 0.05 value were considered significant.

Genes	baseMean	log2FoldChange	lfcSE	stat	pvalue	padj
ADH7	2874.532	-1.00421	0.237966	-4.21997	2.44E-05	0.041236
ENPEP	32.35705	2.58263	0.525359	4.915929	8.84E-07	0.006328
GPATCH4	51.0056	-1.19709	0.284169	-4.21259	2.52E-05	0.041236
IGF2	24.55162	3.851189	0.793619	4.852693	1.22E-06	0.006328
MAP3K12	151.4382	-2.38054	0.549252	-4.33415	1.46E-05	0.032863
MMP16	4.66984	-5.37427	1.300314	-4.13306	3.58E-05	0.049609
PCDHA11	14.09029	-3.17871	0.710306	-4.47513	7.64E-06	0.019601
PTPRQ	60.26947	-2.23112	0.492802	-4.52742	5.97E-06	0.017879
RYR3	99.46414	5.719201	1.185634	4.82375	1.41E-06	0.006328
SAA4	8.002679	4.804457	1.13706	4.225334	2.39E-05	0.041236
SLC26A4	114.124	4.29726	1.039886	4.132433	3.59E-05	0.049609
ZBED6CL	109.8951	1.398693	0.308122	4.539415	5.64E-06	0.017879
ZFP57	16.79348	6.78271	1.351402	5.019018	5.19E-07	0.006328

Fig 3C. Raw data of Gene ontology (GO) analysis of DEGs in infected pediatric NECs were displayed by th
of significantly GO enriched (Overrepresented p value < 0.05) results were marked in red, x-axis reflec
percentage over genes in each GO category; y-axis reflects different GO term:

	category	over_represented_pvalu	under_represented_pvalu	numDEInCat	numInCat
875	GO:0002376	0	1	19	482
3995	GO:0009615	0	1	16	101
9935	GO:0045071	0	1	7	41
9936	GO:0045087	0	1	17	410
11724	GO:0051607	0	1	21	177
12251	GO:0060337	0	1	14	53
12247	GO:0060333	3.52E-09	1	6	65
1313	GO:0003725	4.93E-09	1	6	69
13056	GO:0070106	1.38E-08	1	4	14
15616	GO:0140374	1.41E-08	1	4	14
7310	GO:0032728	1.59E-08	1	5	40
509	GO:0001730	3.03E-08	1	3	4
12465	GO:0060700	3.03E-08	1	3	4
1312	GO:0003724	3.69E-07	0.999999992	5	74
7199	GO:0032480	4.25E-07	0.999999996	4	31
3996	GO:0009617	6.72E-07	0.999999984	5	83
3113	GO:0006952	8.59E-07	0.999999979	5	87
15804	GO:1900246	8.87E-07	0.999999997	3	10
1634	GO:0004386	9.55E-06	0.99999961	5	143
7309	GO:0032727	1.29E-05	0.99999988	3	23
4919	GO:0016032	1.69E-05	0.999998223	8	577
1311	GO:0003723	2.32E-05	0.999995991	12	1473
15803	GO:1900245	2.35E-05	0.99999997	2	4
8885	GO:0039530	2.36E-05	0.99999997	2	4
10904	GO:0048248	2.41E-05	0.999999969	2	4
15005	GO:0098844	3.89E-05	0.999999926	2	5
11272	GO:0050688	3.95E-05	0.999999924	2	5
6955	GO:0032020	5.97E-05	0.999999846	2	6
4974	GO:0016185	8.13E-05	0.999999742	2	7
8884	GO:0039529	8.21E-05	0.999999737	2	7
1315	GO:0003727	9.43E-05	0.999998194	3	44
1263	GO:0003374	0.000108333	0.999999587	2	8
15023	GO:0098884	0.000139952	0.999999375	2	9
7862	GO:0034340	0.000140517	0.999999371	2	9
4333	GO:0010818	0.000141801	0.999999363	2	9
11324	GO:0050803	0.000174027	0.999999114	2	10
8272	GO:0035455	0.000177826	0.999999085	2	10
3116	GO:0006955	0.000190714	0.999985173	5	266
9781	GO:0044327	0.000256079	0.999998366	2	12
8274	GO:0035457	0.000302582	0.999997875	2	13

10011	GO:0045236	0.000308866	0.999997808	2	13
6932	GO:0031966	0.000312615	0.999981974	4	162
77	GO:0000166	0.000499979	0.999885584	11	1741
5556	GO:0019216	0.000533431	0.999981134	3	79
5226	GO:0016779	0.000535605	0.999981031	3	79
118	GO:0000266	0.000589604	0.999993982	2	18
13629	GO:0071360	0.000592135	0.999993943	2	18
7330	GO:0032760	0.000642429	0.999975737	3	84
12253	GO:0060339	0.000660984	0.999992817	2	19
11577	GO:0051281	0.000973818	0.999986927	2	23
9213	GO:0042742	0.001211656	0.999942718	3	104
10126	GO:0045648	0.001332313	0.999978814	2	27
3420	GO:0008015	0.001334118	0.999978771	2	27
4957	GO:0016126	0.001661806	0.999970261	2	30
810	GO:0002230	0.001766329	0.999967339	2	31
3418	GO:0008009	0.001925887	0.999962728	2	32
8252	GO:0035395	0.001986563	1	1	1
7403	GO:0032896	0.00198661	1	1	1
14050	GO:0072308	0.001997353	1	1	1
16540	GO:1903487	0.001997723	1	1	1
16700	GO:1903900	0.002000533	1	1	1
4356	GO:0010844	0.002008228	1	1	1
4054	GO:0009918	0.002010954	1	1	1
4958	GO:0016132	0.002010954	1	1	1
10749	GO:0047598	0.002010954	1	1	1
4378	GO:0010879	0.002017084	1	1	1
7788	GO:0034127	0.00202178	1	1	1
4334	GO:0010819	0.002036345	1	1	1
9879	GO:0044827	0.002046784	1	1	1
3964	GO:0009409	0.002134206	0.999956353	2	34
2398	GO:0005783	0.002194926	0.999470679	9	1464
10965	GO:0048471	0.002227455	0.999627368	6	679
14914	GO:0098586	0.002384856	0.999948255	2	36
2969	GO:0006695	0.002385893	0.999948221	2	36
13557	GO:0071222	0.002693914	0.999830684	3	138
6800	GO:0031623	0.003219772	0.999918066	2	42
12607	GO:0061025	0.003368522	0.999912203	2	43
13050	GO:0070098	0.003594898	0.999903053	2	44
7865	GO:0034344	0.003983093	0.999996116	1	2
8883	GO:0039528	0.003983093	0.999996116	1	2
14699	GO:0097233	0.003983212	0.999996116	1	2
10542	GO:0046725	0.003989508	0.999996104	1	2
1246	GO:0003340	0.00399278	0.999996097	1	2
16727	GO:1903966	0.003994667	0.999996094	1	2
7729	GO:0033862	0.004009738	0.999996064	1	2

8708	GO:0036430	0.004009738	0.999996064	1	2
8709	GO:0036431	0.004009738	0.999996064	1	2
11166	GO:0050145	0.004011322	0.999996061	1	2
12709	GO:0061326	0.004011846	0.99999606	1	2
10132	GO:0045655	0.004015285	0.999996053	1	2
7786	GO:0034124	0.004015357	0.999996053	1	2
14141	GO:0072665	0.004017058	0.99999605	1	2
1908	GO:0004798	0.004038859	0.999996007	1	2
2651	GO:0006227	0.004038859	0.999996007	1	2
2654	GO:0006233	0.004038859	0.999996007	1	2
5502	GO:0019043	0.004041637	0.999996001	1	2
13627	GO:0071357	0.004047221	0.99999599	1	2
1385	GO:0003920	0.004049192	0.999995986	1	2
16242	GO:1902560	0.004049192	0.999995986	1	2
16019	GO:1901740	0.004054385	0.999995976	1	2
5509	GO:0019060	0.004065656	0.999995953	1	2
12909	GO:0061844	0.004104625	0.999881261	2	47
5270	GO:0016887	0.004630466	0.999440138	4	339
8965	GO:0042127	0.004799975	0.99962858	3	170
2604	GO:0006139	0.004908517	0.999843864	2	52
2266	GO:0005525	0.00538534	0.999320325	4	352
7200	GO:0032481	0.005479519	0.999815257	2	55
6378	GO:0030593	0.005943456	0.999790847	2	57
5210	GO:0016717	0.00597615	0.999988335	1	3
1889	GO:0004768	0.0059901	0.99998828	1	3
13981	GO:0072162	0.00600222	0.999988232	1	3
13970	GO:0072136	0.006006475	0.999988216	1	3
17729	GO:2000110	0.006008281	0.999988209	1	3
11491	GO:0051097	0.006018047	0.99998817	1	3
13784	GO:0071659	0.006023592	0.999988148	1	3
4357	GO:0010845	0.006025069	0.999988143	1	3
13417	GO:0070859	0.006025136	0.999988142	1	3
3851	GO:0009041	0.006038781	0.999988088	1	3
117	GO:0000254	0.006039471	0.999988086	1	3
8410	GO:0035710	0.006039495	0.999988086	1	3
819	GO:0002253	0.006051645	0.999988038	1	3
2968	GO:0006694	0.006106404	0.999781996	2	58
2363	GO:0005737	0.00624308	0.997487922	21	6331
2265	GO:0005524	0.006286033	0.998352601	8	1410
7311	GO:0032729	0.006291057	0.999771826	2	59
9384	GO:0043154	0.006702192	0.999748631	2	61
11097	GO:0048763	0.00792357	0.999976914	1	4
7805	GO:0034157	0.007975704	0.999976609	1	4
2148	GO:0005219	0.007976571	0.999976604	1	4
9489	GO:0043367	0.007980272	0.999976582	1	4

15959	GO:1901509	0.007991801	0.999976515	1	4
1520	GO:0004127	0.008010354	0.999976405	1	4
17832	GO:2000342	0.008014058	0.999976383	1	4
7623	GO:0033489	0.008023432	0.999976328	1	4
7624	GO:0033490	0.008023432	0.999976328	1	4
8887	GO:0039534	0.008025704	0.999976314	1	4
2605	GO:0006144	0.008057558	0.999976126	1	4
9030	GO:0042296	0.00806543	0.999976079	1	4
2655	GO:0006235	0.008080919	0.999975987	1	4
6995	GO:0032091	0.008281488	0.999652598	2	68
14671	GO:0097190	0.008309798	0.999650793	2	68
4287	GO:0010742	0.009977652	0.999960926	1	5
7810	GO:0034165	0.009986891	0.999960853	1	5
13779	GO:0071650	0.010002241	0.999960732	1	5
7269	GO:0032649	0.010026473	0.999960542	1	5
4372	GO:0010873	0.010117691	0.999959819	1	5
2611	GO:0006163	0.010121338	0.99995979	1	5
13619	GO:0071346	0.010528306	0.999498419	2	77
9424	GO:0043231	0.010854629	0.997473723	6	942
5128	GO:0016525	0.01097264	0.999465612	2	79
12043	GO:0055114	0.011835303	0.998124053	4	442
1021	GO:0002735	0.011923982	0.99994183	1	6
6865	GO:0031730	0.011981956	0.999941262	1	6
5187	GO:0016628	0.011982947	0.999941252	1	6
3991	GO:0009597	0.011983594	0.999941246	1	6
10559	GO:0046822	0.011986284	0.99994122	1	6
14133	GO:0072594	0.011997597	0.999941108	1	6
11310	GO:0050778	0.012005791	0.999941028	1	6
11838	GO:0051902	0.012016506	0.999940922	1	6
13257	GO:0070508	0.012050457	0.999940587	1	6
3242	GO:0007189	0.012418624	0.999354115	2	84
2424	GO:0005829	0.012958355	0.994597013	17	4986
13591	GO:0071286	0.013889239	0.999918766	1	7
8824	GO:0038114	0.013916414	0.999918447	1	7
12504	GO:0060760	0.013919646	0.999918409	1	7
10561	GO:0046825	0.013999498	0.999917468	1	7
3750	GO:0008603	0.014003903	0.999917416	1	7
17939	GO:2000553	0.014008502	0.999917361	1	7
2404	GO:0005789	0.014206055	0.996493099	6	998
6711	GO:0031410	0.015460792	0.99667197	5	736
3527	GO:0008203	0.015645007	0.999079781	2	95
4693	GO:0015278	0.01579347	0.999892706	1	8
8823	GO:0038113	0.015874413	0.9998916	1	8
8889	GO:0039536	0.015929379	0.999890846	1	8
4154	GO:0010452	0.015954539	0.9998905	1	8

8962	GO:0042118	0.017905772	0.999859795	1	9
2647	GO:0006221	0.017918078	0.999859602	1	9
17906	GO:2000479	0.017937741	0.999859292	1	9
3890	GO:0009142	0.017971936	0.999858753	1	9
5761	GO:0019941	0.018093869	0.999856824	1	9
1389	GO:0003924	0.019049288	0.997548748	3	282
13604	GO:0071313	0.019857942	0.999825284	1	10
10645	GO:0046975	0.019861905	0.999825214	1	10
8943	GO:0042058	0.019927776	0.999824048	1	10
1044	GO:0002819	0.019931793	0.999823977	1	10
11347	GO:0050848	0.01997142	0.999823274	1	10
8273	GO:0035456	0.020053953	0.999821804	1	10
11666	GO:0051481	0.021741915	0.999788307	1	11
10070	GO:0045540	0.021779586	0.99978757	1	11
13374	GO:0070757	0.021805038	0.999787071	1	11
10624	GO:0046940	0.021915548	0.999784898	1	11
1000	GO:0002690	0.022033522	0.999782566	1	11
3526	GO:0008202	0.023254177	0.998308705	2	117
1289	GO:0003676	0.023664199	0.995383955	4	547
7115	GO:0032367	0.023823274	0.999743532	1	12
10627	GO:0046949	0.023846737	0.999743024	1	12
16027	GO:1901799	0.023854998	0.999742846	1	12
6172	GO:0030176	0.023891843	0.99823678	2	119
12248	GO:0060334	0.023907487	0.999741708	1	12
7308	GO:0032725	0.02391306	0.999741587	1	12
4933	GO:0016064	0.0239627	0.999740508	1	12
5710	GO:0019835	0.023969116	0.999740368	1	12
6694	GO:0031386	0.024155875	0.999736288	1	12
12254	GO:0060340	0.02576095	0.999697816	1	13
13774	GO:0071639	0.025796427	0.999696979	1	13
7198	GO:0032479	0.025812843	0.999696591	1	13
2921	GO:0006636	0.025832783	0.99969612	1	13
17877	GO:2000406	0.025891158	0.999694738	1	13
9359	GO:0043101	0.025948948	0.999693368	1	13
3104	GO:0006935	0.026381235	0.997946255	2	125
2253	GO:0005506	0.026633981	0.997915805	2	126
5108	GO:0016491	0.027288119	0.994439255	4	570
11789	GO:0051770	0.027623466	0.999650247	1	14
1298	GO:0003690	0.027625191	0.997795026	2	129
15709	GO:1900025	0.027676768	0.999648889	1	14
8941	GO:0042054	0.027744908	0.999647149	1	14
4444	GO:0010996	0.027815728	0.999645336	1	14
14293	GO:0090026	0.027982434	0.99964105	1	14
1288	GO:0003674	0.029394721	0.99387072	4	584
3525	GO:0008201	0.030116891	0.997481095	2	135

14209	GO:0080182	0.031471227	0.999541046	1	16
9250	GO:0042800	0.031603929	0.999537145	1	16
11283	GO:0050709	0.031704293	0.999534183	1	16
7586	GO:0033280	0.03172492	0.999533573	1	16
13053	GO:0070102	0.033406455	0.999480488	1	17
2262	GO:0005521	0.033526748	0.999476717	1	17
16413	GO:1903077	0.033527538	0.999476692	1	17
13608	GO:0071318	0.033649637	0.99947285	1	17
3767	GO:0008637	0.033657936	0.999472588	1	17
10216	GO:0045824	0.033699003	0.999471292	1	17
3269	GO:0007221	0.035279758	0.999418199	1	18
9482	GO:0043330	0.037518433	0.999339463	1	19
325	GO:0000979	0.037576574	0.9993374	1	19
15467	GO:0120020	0.037594281	0.999336771	1	19
14980	GO:0098793	0.037650875	0.996443917	2	153
1736	GO:0004550	0.037757751	0.99933095	1	19
2613	GO:0006165	0.037757751	0.99933095	1	19
2098	GO:0005125	0.038317553	0.996346376	2	153
3095	GO:0006915	0.039132699	0.991075764	4	640
16412	GO:1903076	0.039501741	0.999265237	1	20
11357	GO:0050860	0.039503467	0.999265173	1	20
6395	GO:0030659	0.039512888	0.996168215	2	157
6313	GO:0030424	0.039533968	0.993261739	3	377
8275	GO:0035458	0.03957374	0.999262537	1	20
717	GO:0002053	0.041232705	0.999196941	1	21
5149	GO:0016571	0.04133332	0.999192988	1	21
10138	GO:0045662	0.041501612	0.999186352	1	21
3754	GO:0008610	0.043194433	0.999116098	1	22
9937	GO:0045088	0.043271747	0.999112906	1	22
13276	GO:0070542	0.043318408	0.999110976	1	22
1734	GO:0004540	0.043435886	0.999106109	1	22
579	GO:0001836	0.043569256	0.999100567	1	22
3290	GO:0007267	0.044625164	0.995373739	2	167
8039	GO:0034704	0.045096359	0.999033905	1	23
7297	GO:0032703	0.04522396	0.999028384	1	23
9808	GO:0044389	0.045312345	0.999024551	1	23
2753	GO:0006401	0.045332363	0.999023682	1	23
10275	GO:0045944	0.04854393	0.985881048	5	999
8186	GO:0035257	0.048739861	0.998865935	1	25
5401	GO:0018024	0.050759689	0.998767143	1	26
2405	GO:0005790	0.050831196	0.998763635	1	26
8113	GO:0035035	0.050844911	0.998762961	1	26
2316	GO:0005640	0.050845267	0.998762944	1	26
4137	GO:0010332	0.050868682	0.998761794	1	26
4747	GO:0015630	0.051599754	0.994203796	2	182

2918	GO:0006631	0.051831258	0.994163441	2	182
6337	GO:0030507	0.05263726	0.998671407	1	27
2366	GO:0005741	0.052793453	0.993994118	2	184
2127	GO:0005164	0.053095827	0.99864793	1	27
8942	GO:0042056	0.053196441	0.99864275	1	27
11771	GO:0051721	0.054619063	0.998566509	1	28
9934	GO:0045070	0.054691128	0.998562686	1	28
3284	GO:0007259	0.054708035	0.998561788	1	28
5186	GO:0016627	0.054800397	0.998556879	1	28
643	GO:0001937	0.056543258	0.998460717	1	29
10638	GO:0046966	0.058515223	0.99834836	1	30
8390	GO:0035666	0.058705241	0.9983375	1	30
3885	GO:0009117	0.058911513	0.998325669	1	30
11541	GO:0051209	0.060324914	0.998241539	1	31
14674	GO:0097193	0.060628772	0.998223582	1	31
14503	GO:0090501	0.060774353	0.998214945	1	31
7473	GO:0033017	0.062210099	0.998126713	1	32
4451	GO:0012506	0.06223545	0.998125169	1	32
18178	GO:2001240	0.062474955	0.998110545	1	32
7315	GO:0032733	0.062494444	0.998109352	1	32
6332	GO:0030501	0.067862573	0.997760157	1	35
14132	GO:0072593	0.068171471	0.997739463	1	35
2257	GO:0005515	0.068529128	0.964195267	30	12266
5777	GO:0019985	0.069916686	0.997618816	1	36
11255	GO:0050661	0.071799385	0.997485216	1	37
11400	GO:0050918	0.07196473	0.997473466	1	37
3327	GO:0007389	0.075308521	0.997225968	1	39
13249	GO:0070491	0.075329526	0.997224399	1	39
11711	GO:0051591	0.07542913	0.997216952	1	39
5764	GO:0019955	0.075584093	0.997205345	1	39
9295	GO:0042981	0.077960064	0.98896411	2	230
9251	GO:0042802	0.078137763	0.971107621	6	1501
352	GO:0001102	0.079167598	0.996926178	1	41
14672	GO:0097191	0.079519979	0.99689835	1	41
11821	GO:0051879	0.080999625	0.996778228	1	42
4737	GO:0015485	0.081090055	0.996770922	1	42
15460	GO:0120009	0.082980461	0.996614384	1	43
9372	GO:0043124	0.084508887	0.996484684	1	44
7980	GO:0034605	0.084668956	0.996471145	1	44
11684	GO:0051539	0.084849272	0.996455862	1	44
5157	GO:0016579	0.086786201	0.986940782	2	245
6032	GO:0022627	0.087757372	0.99620277	1	45
5231	GO:0016787	0.089189593	0.965864467	6	1556
16655	GO:1903779	0.089954645	0.996003245	1	47
4746	GO:0015629	0.08998287	0.986176229	2	251

3422	GO:0008017	0.090573227	0.986033253	2	252
5559	GO:0019221	0.094785777	0.984997128	2	258
9534	GO:0043434	0.097432927	0.995290287	1	51
11583	GO:0051289	0.097548322	0.995278922	1	51
2917	GO:0006629	0.098246136	0.975750744	3	550
9948	GO:0045111	0.099137509	0.995119145	1	52
4498	GO:0014069	0.099900109	0.983700785	2	267
2313	GO:0005637	0.101058481	0.994922716	1	53
9624	GO:0043621	0.101072943	0.994921235	1	53
7328	GO:0032757	0.101287499	0.994899242	1	53
6702	GO:0031397	0.101310294	0.994896902	1	53
17639	GO:1990841	0.10275163	0.994746038	1	54
13618	GO:0071345	0.102915961	0.994728896	1	54
3994	GO:0009612	0.103066769	0.994713139	1	54
4167	GO:0010468	0.103834933	0.982675753	2	273
9640	GO:0043657	0.104717408	0.99453727	1	55
7770	GO:0034097	0.104779208	0.994530694	1	55
9127	GO:0042542	0.105171301	0.994488877	1	55
6296	GO:0030374	0.106225179	0.994373867	1	56
11040	GO:0048661	0.11006917	0.993946961	1	58
37	GO:0000079	0.110536787	0.99389435	1	58
5130	GO:0016529	0.111809438	0.993748178	1	59
13455	GO:0070936	0.113705145	0.993527995	1	60
6802	GO:0031625	0.115088739	0.979612395	2	290
327	GO:0000987	0.120569596	0.99269673	1	64
2919	GO:0006633	0.121011509	0.992641869	1	64
13620	GO:0071347	0.122951827	0.992396671	1	65
11681	GO:0051536	0.124709729	0.992170728	1	66
13661	GO:0071407	0.126269443	0.991967165	1	67
13189	GO:0070373	0.127787852	0.991766326	1	68
5499	GO:0019003	0.131735225	0.991233112	1	70
4179	GO:0010506	0.133074539	0.991047649	1	71
2282	GO:0005575	0.13330119	0.974251568	2	317
3115	GO:0006954	0.133401329	0.974220789	2	317
3380	GO:0007584	0.13501461	0.990776169	1	72
2176	GO:0005262	0.137820869	0.990374985	1	74
13584	GO:0071277	0.139835715	0.990082162	1	75
1961	GO:0004867	0.146083814	0.989144804	1	78
7327	GO:0032755	0.147115735	0.988984828	1	79
2319	GO:0005643	0.148458721	0.988775311	1	80
6350	GO:0030522	0.14846091	0.98877497	1	80
15501	GO:0120162	0.153867515	0.987912161	1	83
3064	GO:0006874	0.155449904	0.987653032	1	84
6282	GO:0030334	0.162023348	0.986545634	1	88
3363	GO:0007517	0.162226383	0.986510831	1	88

2415	GO:0005811	0.165864762	0.985875652	1	90
4165	GO:0010466	0.16832581	0.985437768	1	91
6309	GO:0030414	0.171700083	0.984824731	1	93
7392	GO:0032869	0.172070133	0.984755393	1	94
3274	GO:0007229	0.17356239	0.98447914	1	95
1944	GO:0004843	0.175356585	0.984143818	1	96
7254	GO:0032587	0.176937612	0.983844967	1	97
5743	GO:0019899	0.177137562	0.959387405	2	380
2364	GO:0005739	0.183920391	0.92031503	5	1505
11488	GO:0051091	0.183938183	0.982486485	1	101
9068	GO:0042383	0.190272771	0.981205824	1	105
11453	GO:0051028	0.190414974	0.981176662	1	105
11308	GO:0050776	0.191338781	0.98098662	1	105
1331	GO:0003824	0.191979433	0.953746589	2	400
7560	GO:0033209	0.198238711	0.979527882	1	109
1290	GO:0003677	0.199180021	0.904500368	6	1970
13626	GO:0071356	0.200794539	0.978971939	1	111
13308	GO:0070588	0.201237982	0.978873633	1	112
4233	GO:0010628	0.204791693	0.948634206	2	418
6075	GO:0030018	0.206667401	0.977664981	1	115
2487	GO:0005923	0.207905022	0.977383876	1	116
3033	GO:0006816	0.209441361	0.977032658	1	117
55	GO:0000122	0.209560146	0.927544143	3	788
4419	GO:0010951	0.210970639	0.976681704	1	117
4499	GO:0014070	0.215009662	0.975736052	1	120
88	GO:0000184	0.215329953	0.975662144	1	119
3571	GO:0008284	0.221781653	0.941511115	2	441
2309	GO:0005615	0.225174947	0.905572476	4	1209
2368	GO:0005743	0.226206023	0.939593184	2	444
6906	GO:0031902	0.226214765	0.973007454	1	127
3062	GO:0006869	0.227085894	0.97278797	1	128
2359	GO:0005730	0.227366685	0.918283762	3	820
2138	GO:0005178	0.231576189	0.971644605	1	131
4914	GO:0016020	0.235279011	0.849535919	17	7114
9349	GO:0043066	0.238328989	0.934198882	2	463
459	GO:0001650	0.241295513	0.969081968	1	137
2147	GO:0005216	0.249696611	0.966767076	1	143
11776	GO:0051726	0.261103625	0.963477301	1	150
9071	GO:0042393	0.263316338	0.962818233	1	152
17650	GO:1990904	0.270164772	0.960740054	1	156
7213	GO:0032496	0.271782322	0.96023991	1	157
5247	GO:0016829	0.272152567	0.960125267	1	157
5213	GO:0016740	0.272381122	0.86484148	5	1750
6934	GO:0031982	0.277503428	0.958441851	1	161
2283	GO:0005576	0.284876041	0.856313998	5	1775

817	GO:0002250	0.289416975	0.95455451	1	169
2463	GO:0005886	0.290386488	0.821198511	10	4063
1303	GO:0003700	0.298050707	0.904743356	2	544
2258	GO:0005516	0.300824202	0.950646184	1	178
6045	GO:0022857	0.303990897	0.949529525	1	180
3502	GO:0008168	0.307617206	0.948233751	1	182
9386	GO:0043161	0.310564115	0.947166444	1	184
2321	GO:0005654	0.313170578	0.807556012	9	3676
7062	GO:0032259	0.320167702	0.943601135	1	191
12026	GO:0055085	0.320318194	0.89252892	2	574
2312	GO:0005635	0.324197026	0.942065455	1	194
3029	GO:0006811	0.324682637	0.890055798	2	580
5436	GO:0018215	0.339060206	0.936194823	1	205
3223	GO:0007166	0.340834471	0.935472557	1	206
3222	GO:0007165	0.346951493	0.825709969	4	1480
322	GO:0000976	0.360834017	0.926995962	1	222
9252	GO:0042803	0.36168652	0.868036952	2	629
6283	GO:0030335	0.362203548	0.926393244	1	223
3239	GO:0007186	0.369253039	0.863301563	2	637
6931	GO:0031965	0.37141632	0.92226266	1	230
10245	GO:0045893	0.375334891	0.859435243	2	649
4048	GO:0009897	0.388320369	0.914335144	1	243
1943	GO:0004842	0.392381572	0.912361192	1	247
10582	GO:0046872	0.406175473	0.736859846	8	3506
7830	GO:0034220	0.414320708	0.901233373	1	265
93	GO:0000209	0.416170968	0.900258754	1	266
2388	GO:0005769	0.418935597	0.898789924	1	269
12819	GO:0061630	0.419153806	0.898673712	1	269
9115	GO:0042493	0.420938554	0.897719001	1	270
4081	GO:0010008	0.428843607	0.893421707	1	277
2825	GO:0006511	0.431984201	0.891684218	1	280
2722	GO:0006355	0.439203994	0.77781391	3	1208
3559	GO:0008270	0.444177138	0.811992539	2	746
3575	GO:0008289	0.450726144	0.880956886	1	296
256	GO:0000785	0.463145058	0.797693268	2	774
10028	GO:0045296	0.467672584	0.870710677	1	312
2085	GO:0005102	0.474339003	0.866535747	1	317
2386	GO:0005765	0.484308794	0.860132576	1	327
2707	GO:0006325	0.492714163	0.854585186	1	336
9348	GO:0043065	0.499739601	0.849844122	1	342
1326	GO:0003779	0.518913895	0.836398166	1	362
3572	GO:0008285	0.535309254	0.82430077	1	378
9600	GO:0043565	0.539460028	0.821146763	1	383
2385	GO:0005764	0.563214838	0.802373256	1	408
6314	GO:0030425	0.569102438	0.797522428	1	416

1294	GO:0003682	0.569207778	0.797435036	1	416
17636	GO:1990837	0.570985857	0.795955154	1	417
324	GO:0000978	0.600107756	0.677185476	2	997
326	GO:0000981	0.607488031	0.669756236	2	1010
3127	GO:0006974	0.643819597	0.728593148	1	508
2352	GO:0005694	0.66931225	0.701634043	1	544
5146	GO:0016567	0.683218535	0.686105959	1	565
2311	GO:0005634	0.685520939	0.444753642	11	5968
64	GO:0000139	0.696031096	0.671257161	1	585
4915	GO:0016021	0.705259329	0.438120897	8	4487
2255	GO:0005509	0.727825979	0.632010675	1	639
2408	GO:0005794	0.73224507	0.526811954	2	1271
4580	GO:0015031	0.738262193	0.61833377	1	657
5035	GO:0016301	0.739626869	0.616513818	1	661
5040	GO:0016310	0.743362752	0.611496704	1	668
1		0.748883764	0.400519092	37	18891
7458	GO:0032991	0.758537196	0.590545944	1	696
6099	GO:0030054	0.82357627	0.488927687	1	849
9305	GO:0042995	0.889133441	0.361164637	1	1070
2442	GO:0005856	0.938820026	0.237841126	1	1349
2724	GO:0006357	0.946435823	0.215841778	1	1411
10093	GO:0045597	1	0.930167727	0	36
6507	GO:0030955	1	0.978194318	0	11
4238	GO:0010634	1	0.941706156	0	30
1324	GO:0003774	1	0.86414964	0	73
3173	GO:0007049	1	0.265639651	0	650
11329	GO:0050808	1	0.895702044	0	55
5178	GO:0016607	1	0.448056331	0	396
2749	GO:0006397	1	0.472888924	0	369
2084	GO:0005096	1	0.588896102	0	263
4073	GO:0009986	1	0.337236208	0	535
2464	GO:0005887	1	0.0953624	0	1140
12531	GO:0060828	1	0.937817391	0	32
2	GO:0000002	1	0.978132247	0	11
3	GO:0000003	1	0.991910284	0	4
4	GO:0000009	1	0.995973121	0	2
5	GO:0000010	1	0.99597848	0	2
6	GO:0000012	1	0.984085335	0	8
7	GO:0000014	1	0.982099965	0	9
8	GO:0000015	1	0.991945513	0	4
9	GO:0000017	1	0.995999837	0	2
10	GO:0000018	1	0.990000298	0	5
11	GO:0000019	1	0.996007903	0	2
12	GO:0000022	1	0.996000761	0	2
13	GO:0000023	1	0.994039789	0	3

14	GO:0000026	1	0.993996842	0	3
15	GO:0000027	1	0.956334129	0	22
16	GO:0000028	1	0.971826049	0	14
17	GO:0000030	1	0.962661854	0	19
18	GO:0000032	1	0.997974765	0	1
19	GO:0000033	1	0.993948811	0	3
20	GO:0000035	1	0.997953503	0	1
21	GO:0000036	1	0.997953503	0	1
22	GO:0000038	1	0.962486671	0	19
23	GO:0000045	1	0.889844268	0	58
24	GO:0000048	1	0.991974363	0	4
25	GO:0000049	1	0.864895184	0	72
26	GO:0000050	1	0.981999271	0	9
27	GO:0000052	1	0.993961898	0	3
28	GO:0000053	1	0.997975673	0	1
29	GO:0000054	1	0.989987675	0	5
30	GO:0000055	1	0.985968045	0	7
31	GO:0000056	1	0.985926673	0	7
32	GO:0000062	1	0.966255566	0	17
33	GO:0000064	1	0.992026283	0	4
34	GO:0000070	1	0.939521224	0	31
35	GO:0000076	1	0.980089177	0	10
36	GO:0000077	1	0.934001007	0	34
38	GO:0000082	1	0.866818043	0	71
39	GO:0000083	1	0.945222801	0	28
40	GO:0000086	1	0.758803609	0	137
41	GO:0000095	1	0.997982324	0	1
42	GO:0000096	1	0.98801999	0	6
43	GO:0000098	1	0.987941148	0	6
44	GO:0000103	1	0.995997377	0	2
45	GO:0000104	1	0.995978998	0	2
46	GO:0000105	1	0.995989682	0	2
47	GO:0000109	1	0.988063437	0	6
48	GO:0000110	1	0.99399571	0	3
49	GO:0000111	1	0.998003243	0	1
50	GO:0000112	1	0.997990665	0	1
51	GO:0000117	1	0.99801343	0	1
52	GO:0000118	1	0.915665624	0	44
53	GO:0000120	1	0.995969731	0	2
54	GO:0000121	1	0.9979964	0	1
56	GO:0000123	1	0.947259137	0	27
57	GO:0000124	1	0.976086862	0	12
58	GO:0000125	1	0.98794345	0	6
59	GO:0000126	1	0.994006702	0	3
60	GO:0000127	1	0.98802336	0	6

61	GO:0000132	1	0.943418699	0	29
62	GO:0000137	1	0.952967507	0	24
63	GO:0000138	1	0.980061325	0	10
65	GO:0000145	1	0.960681849	0	20
66	GO:0000146	1	0.959040981	0	21
67	GO:0000149	1	0.884624741	0	61
68	GO:0000150	1	0.995969624	0	2
69	GO:0000151	1	0.801388334	0	110
70	GO:0000152	1	0.995987061	0	2
71	GO:0000153	1	0.997965121	0	1
72	GO:0000154	1	0.986012942	0	7
73	GO:0000159	1	0.964442318	0	18
74	GO:0000160	1	0.998004391	0	1
75	GO:0000164	1	0.97229404	0	14
76	GO:0000165	1	0.563001016	0	284
78	GO:0000170	1	0.997968061	0	1
79	GO:0000171	1	0.995953816	0	2
80	GO:0000172	1	0.983960143	0	8
81	GO:0000175	1	0.937587796	0	32
82	GO:0000176	1	0.968062038	0	16
83	GO:0000177	1	0.9760249	0	12
84	GO:0000178	1	0.96424709	0	18
85	GO:0000179	1	0.991945801	0	4
86	GO:0000182	1	0.990054503	0	5
87	GO:0000183	1	0.930124521	0	36
89	GO:0000185	1	0.976146481	0	12
90	GO:0000186	1	0.908271865	0	48
91	GO:0000187	1	0.788294072	0	118
92	GO:0000188	1	0.952812379	0	24
94	GO:0000210	1	0.993987201	0	3
95	GO:0000212	1	0.988034948	0	6
96	GO:0000213	1	0.995967687	0	2
97	GO:0000214	1	0.991939641	0	4
98	GO:0000215	1	0.995954978	0	2
99	GO:0000217	1	0.995997162	0	2
100	GO:0000220	1	0.992029667	0	4
101	GO:0000221	1	0.994002519	0	3
102	GO:0000224	1	0.997987219	0	1
103	GO:0000225	1	0.997963285	0	1
104	GO:0000226	1	0.760589093	0	136
105	GO:0000228	1	0.902504274	0	51
106	GO:0000235	1	0.99204374	0	4
107	GO:0000242	1	0.956851149	0	22
108	GO:0000243	1	0.989932147	0	5
109	GO:0000244	1	0.974217422	0	13

110	GO:0000245	1	0.948837853	0	26
111	GO:0000246	1	0.998009932	0	1
112	GO:0000247	1	0.997963324	0	1
113	GO:0000248	1	0.996005222	0	2
114	GO:0000250	1	0.998009713	0	1
115	GO:0000252	1	0.997971684	0	1
116	GO:0000253	1	0.991894228	0	4
119	GO:0000268	1	0.99199769	0	4
120	GO:0000271	1	0.998013437	0	1
121	GO:0000272	1	0.997983681	0	1
122	GO:0000274	1	0.995985075	0	2
123	GO:0000275	1	0.993983524	0	3
124	GO:0000276	1	0.978112005	0	11
125	GO:0000278	1	0.743474487	0	147
126	GO:0000281	1	0.906356535	0	49
127	GO:0000285	1	0.998013437	0	1
129	GO:0000288	1	0.976262441	0	12
130	GO:0000289	1	0.95684096	0	22
131	GO:0000290	1	0.982037588	0	9
132	GO:0000291	1	0.995962945	0	2
133	GO:0000293	1	0.994003684	0	3
134	GO:0000294	1	0.996007208	0	2
135	GO:0000295	1	0.997967026	0	1
136	GO:0000296	1	0.997983315	0	1
137	GO:0000298	1	0.98997282	0	5
138	GO:0000301	1	0.995990219	0	2
139	GO:0000302	1	0.962326684	0	19
140	GO:0000303	1	0.989930131	0	5
141	GO:0000304	1	0.997979874	0	1
142	GO:0000305	1	0.99797822	0	1
143	GO:0000306	1	0.997996022	0	1
144	GO:0000307	1	0.941320552	0	30
145	GO:0000308	1	0.996023736	0	2
146	GO:0000309	1	0.99400654	0	3
147	GO:0000320	1	0.994016072	0	3
148	GO:0000322	1	0.993974412	0	3
149	GO:0000323	1	0.998013165	0	1
150	GO:0000333	1	0.99800762	0	1
151	GO:0000334	1	0.997965887	0	1
152	GO:0000338	1	0.979971347	0	10
153	GO:0000339	1	0.97614886	0	12
154	GO:0000340	1	0.978178023	0	11
155	GO:0000346	1	0.974112056	0	13
156	GO:0000347	1	0.989949377	0	5
157	GO:0000349	1	0.993975932	0	3

158	GO:0000350	1	0.993950744	0	3
159	GO:0000354	1	0.996015877	0	2
160	GO:0000375	1	0.950883926	0	25
161	GO:0000379	1	0.993953375	0	3
162	GO:0000380	1	0.960583007	0	20
163	GO:0000381	1	0.893611457	0	56
164	GO:0000386	1	0.998002621	0	1
165	GO:0000387	1	0.944902269	0	28
166	GO:0000388	1	0.998013437	0	1
167	GO:0000389	1	0.98800789	0	6
168	GO:0000390	1	0.996006125	0	2
169	GO:0000395	1	0.987987494	0	6
170	GO:0000398	1	0.601238827	0	251
171	GO:0000400	1	0.9664753	0	17
172	GO:0000403	1	0.990066092	0	5
173	GO:0000404	1	0.998003243	0	1
174	GO:0000405	1	0.984150149	0	8
175	GO:0000406	1	0.995998824	0	2
176	GO:0000407	1	0.954935027	0	23
177	GO:0000408	1	0.987935496	0	6
178	GO:0000413	1	0.922208107	0	40
179	GO:0000414	1	0.998013437	0	1
180	GO:0000415	1	0.998013437	0	1
181	GO:0000416	1	0.998004597	0	1
182	GO:0000417	1	0.998007874	0	1
183	GO:0000421	1	0.926287871	0	38
184	GO:0000422	1	0.93186798	0	35
185	GO:0000423	1	0.982028641	0	9
186	GO:0000425	1	0.986075973	0	7
187	GO:0000430	1	0.995950914	0	2
188	GO:0000432	1	0.993944148	0	3
189	GO:0000435	1	0.998013437	0	1
190	GO:0000438	1	0.993933955	0	3
191	GO:0000439	1	0.978011624	0	11
192	GO:0000444	1	0.991968365	0	4
193	GO:0000445	1	0.987944944	0	6
194	GO:0000447	1	0.981983563	0	9
195	GO:0000448	1	0.996002154	0	2
196	GO:0000451	1	0.993968785	0	3
197	GO:0000453	1	0.99800266	0	1
198	GO:0000454	1	0.993900664	0	3
199	GO:0000455	1	0.987943754	0	6
200	GO:0000460	1	0.974105085	0	13
201	GO:0000461	1	0.997947973	0	1
202	GO:0000462	1	0.948896291	0	26

203	GO:0000463	1	0.972113308	0	14
204	GO:0000466	1	0.990006502	0	5
205	GO:0000467	1	0.981955139	0	9
206	GO:0000469	1	0.993932661	0	3
207	GO:0000470	1	0.972054479	0	14
208	GO:0000472	1	0.990007609	0	5
209	GO:0000479	1	0.994019176	0	3
210	GO:0000480	1	0.986036264	0	7
211	GO:0000481	1	0.998003205	0	1
212	GO:0000492	1	0.981947125	0	9
213	GO:0000493	1	0.995970795	0	2
214	GO:0000494	1	0.993919329	0	3
215	GO:0000495	1	0.99598697	0	2
216	GO:0000502	1	0.885387477	0	60
217	GO:0000506	1	0.985932882	0	7
218	GO:0000578	1	0.990052961	0	5
219	GO:0000700	1	0.997998115	0	1
220	GO:0000701	1	0.995946814	0	2
221	GO:0000703	1	0.995936787	0	2
222	GO:0000706	1	0.997990564	0	1
223	GO:0000707	1	0.997959006	0	1
224	GO:0000710	1	0.990046202	0	5
225	GO:0000711	1	0.995968958	0	2
226	GO:0000712	1	0.964490918	0	18
227	GO:0000715	1	0.954606089	0	23
228	GO:0000717	1	0.956607737	0	22
229	GO:0000718	1	0.997973945	0	1
230	GO:0000720	1	0.991997935	0	4
231	GO:0000721	1	0.997987078	0	1
232	GO:0000722	1	0.978122721	0	11
233	GO:0000723	1	0.9061832	0	49
234	GO:0000724	1	0.844551808	0	84
235	GO:0000725	1	0.993983524	0	3
236	GO:0000726	1	0.997984441	0	1
237	GO:0000727	1	0.976162437	0	12
238	GO:0000729	1	0.970384803	0	15
239	GO:0000730	1	0.99396697	0	3
240	GO:0000731	1	0.976200522	0	12
241	GO:0000732	1	0.998011553	0	1
242	GO:0000733	1	0.984120424	0	8
243	GO:0000735	1	0.998011808	0	1
244	GO:0000737	1	0.981987022	0	9
245	GO:0000738	1	0.983966327	0	8
246	GO:0000768	1	0.996004302	0	2
247	GO:0000773	1	0.997960141	0	1

248	GO:0000774	1	0.978076554	0	11
249	GO:0000775	1	0.738921424	0	150
250	GO:0000776	1	0.772524659	0	128
251	GO:0000777	1	0.817379445	0	100
252	GO:0000778	1	0.978076205	0	11
253	GO:0000779	1	0.966409667	0	17
255	GO:0000783	1	0.982064336	0	9
257	GO:0000786	1	0.835864703	0	89
258	GO:0000791	1	0.933951714	0	34
259	GO:0000792	1	0.911854403	0	46
260	GO:0000793	1	0.943524717	0	29
261	GO:0000794	1	0.924678335	0	39
262	GO:0000795	1	0.947000849	0	27
263	GO:0000796	1	0.98416539	0	8
264	GO:0000800	1	0.97430128	0	13
265	GO:0000801	1	0.985993163	0	7
266	GO:0000802	1	0.996015313	0	2
267	GO:0000803	1	0.996030529	0	2
268	GO:0000805	1	0.994051559	0	3
269	GO:0000806	1	0.998013125	0	1
270	GO:0000808	1	0.988002428	0	6
271	GO:0000810	1	0.993983524	0	3
272	GO:0000811	1	0.991975182	0	4
273	GO:0000812	1	0.976107724	0	12
274	GO:0000813	1	0.980034711	0	10
275	GO:0000814	1	0.993949504	0	3
276	GO:0000815	1	0.979987122	0	10
277	GO:0000818	1	0.991945627	0	4
278	GO:0000819	1	0.994050473	0	3
279	GO:0000820	1	0.997963596	0	1
280	GO:0000821	1	0.99798298	0	1
281	GO:0000822	1	0.994029931	0	3
282	GO:0000823	1	0.997975112	0	1
283	GO:0000825	1	0.995977306	0	2
284	GO:0000827	1	0.992050608	0	4
285	GO:0000828	1	0.984091654	0	8
286	GO:0000829	1	0.996030582	0	2
287	GO:0000831	1	0.997990665	0	1
288	GO:0000832	1	0.990033157	0	5
289	GO:0000835	1	0.997990564	0	1
290	GO:0000836	1	0.992034957	0	4
291	GO:0000839	1	0.985994002	0	7
292	GO:0000900	1	0.970378804	0	15
293	GO:0000902	1	0.855010348	0	78
294	GO:0000904	1	0.98616218	0	7

295	GO:0000915	1	0.992030325	0	4
296	GO:0000916	1	0.997981567	0	1
297	GO:0000917	1	0.997976058	0	1
298	GO:0000921	1	0.998012345	0	1
300	GO:0000923	1	0.990055908	0	5
301	GO:0000930	1	0.970359271	0	15
302	GO:0000932	1	0.830826707	0	92
303	GO:0000938	1	0.990034946	0	5
304	GO:0000939	1	0.995962316	0	2
305	GO:0000940	1	0.982104096	0	9
306	GO:0000941	1	0.995960837	0	2
307	GO:0000942	1	0.991968862	0	4
308	GO:0000956	1	0.962452521	0	19
309	GO:0000957	1	0.998011657	0	1
310	GO:0000958	1	0.994017197	0	3
311	GO:0000959	1	0.997994558	0	1
312	GO:0000961	1	0.996007903	0	2
313	GO:0000962	1	0.994023145	0	3
314	GO:0000963	1	0.997992183	0	1
315	GO:0000964	1	0.996006126	0	2
316	GO:0000965	1	0.996001945	0	2
317	GO:0000966	1	0.998005842	0	1
318	GO:0000971	1	0.997990564	0	1
319	GO:0000972	1	0.992000495	0	4
320	GO:0000973	1	0.995974262	0	2
321	GO:0000974	1	0.976065554	0	12
323	GO:0000977	1	0.541986753	0	303
328	GO:0000993	1	0.941496541	0	30
329	GO:0000994	1	0.997974835	0	1
330	GO:0000995	1	0.987990647	0	6
331	GO:0001002	1	0.994013422	0	3
332	GO:0001003	1	0.994013422	0	3
333	GO:0001006	1	0.988037798	0	6
334	GO:0001010	1	0.997993311	0	1
335	GO:0001016	1	0.997955277	0	1
336	GO:0001018	1	0.993991235	0	3
337	GO:0001042	1	0.99404219	0	3
338	GO:0001046	1	0.966366223	0	17
339	GO:0001054	1	0.99598177	0	2
340	GO:0001055	1	0.991935817	0	4
341	GO:0001056	1	0.99796438	0	1
342	GO:0001067	1	0.996024402	0	2
343	GO:0001069	1	0.996028876	0	2
344	GO:0001085	1	0.91545101	0	44
345	GO:0001091	1	0.993942709	0	3

346	GO:0001093	1	0.992032549	0	4
347	GO:0001094	1	0.980043034	0	10
348	GO:0001096	1	0.995995446	0	2
349	GO:0001097	1	0.996000667	0	2
350	GO:0001099	1	0.997964467	0	1
351	GO:0001100	1	0.997990564	0	1
353	GO:0001103	1	0.933935125	0	34
354	GO:0001112	1	0.998010172	0	1
355	GO:0001113	1	0.997994865	0	1
356	GO:0001147	1	0.996018459	0	2
357	GO:0001156	1	0.994051875	0	3
358	GO:0001161	1	0.996019259	0	2
359	GO:0001162	1	0.985957225	0	7
360	GO:0001164	1	0.984080408	0	8
361	GO:0001165	1	0.998011586	0	1
362	GO:0001172	1	0.99800762	0	1
363	GO:0001174	1	0.99797251	0	1
364	GO:0001179	1	0.997976263	0	1
365	GO:0001181	1	0.992058779	0	4
366	GO:0001188	1	0.982088162	0	9
367	GO:0001193	1	0.997957723	0	1
368	GO:0001207	1	0.99799824	0	1
369	GO:0001216	1	0.97815085	0	11
370	GO:0001217	1	0.984087766	0	8
371	GO:0001221	1	0.978164645	0	11
372	GO:0001222	1	0.976235592	0	12
373	GO:0001223	1	0.960720618	0	20
374	GO:0001225	1	0.988091366	0	6
375	GO:0001226	1	0.964528928	0	18
376	GO:0001227	1	0.580775328	0	269
377	GO:0001228	1	0.471112939	0	372
378	GO:0001306	1	0.998013437	0	1
379	GO:0001315	1	0.997960735	0	1
380	GO:0001401	1	0.974092143	0	13
381	GO:0001405	1	0.989967777	0	5
382	GO:0001409	1	0.99600301	0	2
383	GO:0001501	1	0.785649721	0	120
384	GO:0001502	1	0.968429879	0	16
385	GO:0001503	1	0.827431098	0	94
386	GO:0001504	1	0.982084363	0	9
387	GO:0001505	1	0.993986864	0	3
388	GO:0001507	1	0.995986428	0	2
389	GO:0001508	1	0.964435864	0	18
390	GO:0001510	1	0.958648273	0	21
391	GO:0001512	1	0.997963052	0	1

392	GO:0001514	1	0.984075161	0	8
393	GO:0001515	1	0.99797822	0	1
394	GO:0001516	1	0.9662882	0	17
395	GO:0001517	1	0.986056615	0	7
396	GO:0001518	1	0.972486674	0	14
397	GO:0001519	1	0.995985142	0	2
398	GO:0001520	1	0.987968344	0	6
399	GO:0001522	1	0.968153855	0	16
400	GO:0001523	1	0.911460295	0	46
401	GO:0001525	1	0.616344437	0	240
402	GO:0001527	1	0.978179276	0	11
403	GO:0001530	1	0.952739783	0	24
404	GO:0001532	1	0.997998394	0	1
405	GO:0001533	1	0.916928873	0	43
406	GO:0001534	1	0.991950242	0	4
407	GO:0001536	1	0.993983524	0	3
408	GO:0001537	1	0.991975587	0	4
409	GO:0001540	1	0.861606169	0	74
410	GO:0001541	1	0.931966186	0	35
411	GO:0001542	1	0.990084007	0	5
412	GO:0001543	1	0.998013437	0	1
413	GO:0001544	1	0.995998858	0	2
414	GO:0001545	1	0.99800808	0	1
415	GO:0001546	1	0.995955505	0	2
416	GO:0001547	1	0.994020064	0	3
417	GO:0001550	1	0.996029045	0	2
418	GO:0001552	1	0.997977617	0	1
419	GO:0001553	1	0.988119513	0	6
420	GO:0001554	1	0.994014763	0	3
421	GO:0001555	1	0.995973419	0	2
422	GO:0001556	1	0.962521595	0	19
423	GO:0001558	1	0.86700531	0	71
424	GO:0001560	1	0.996025353	0	2
425	GO:0001561	1	0.984005639	0	8
426	GO:0001562	1	0.997988262	0	1
427	GO:0001567	1	0.997967479	0	1
428	GO:0001568	1	0.904629868	0	50
429	GO:0001569	1	0.949184926	0	26
430	GO:0001570	1	0.900942095	0	52
431	GO:0001572	1	0.998012436	0	1
432	GO:0001573	1	0.992035544	0	4
433	GO:0001574	1	0.98198881	0	9
434	GO:0001576	1	0.997980071	0	1
435	GO:0001578	1	0.93970775	0	31
436	GO:0001579	1	0.996000009	0	2

437	GO:0001580	1	0.948407773	0	26
438	GO:0001581	1	0.998013216	0	1
439	GO:0001582	1	0.997987546	0	1
440	GO:0001587	1	0.99798234	0	1
441	GO:0001588	1	0.997984599	0	1
442	GO:0001591	1	0.995960298	0	2
443	GO:0001595	1	0.997962798	0	1
444	GO:0001602	1	0.993983578	0	3
445	GO:0001605	1	0.995972769	0	2
446	GO:0001607	1	0.997998452	0	1
447	GO:0001609	1	0.991960993	0	4
448	GO:0001614	1	0.989952917	0	5
449	GO:0001618	1	0.872223378	0	68
450	GO:0001621	1	0.995974394	0	2
451	GO:0001626	1	0.997998555	0	1
452	GO:0001631	1	0.997970063	0	1
453	GO:0001632	1	0.995975613	0	2
454	GO:0001635	1	0.991987892	0	4
455	GO:0001640	1	0.98810283	0	6
456	GO:0001641	1	0.997988821	0	1
457	GO:0001642	1	0.998009271	0	1
458	GO:0001649	1	0.816182036	0	101
460	GO:0001651	1	0.99595814	0	2
461	GO:0001652	1	0.985943639	0	7
462	GO:0001653	1	0.992038345	0	4
463	GO:0001654	1	0.926588035	0	38
464	GO:0001655	1	0.970283158	0	15
465	GO:0001656	1	0.945205084	0	28
466	GO:0001657	1	0.930247083	0	36
467	GO:0001658	1	0.93406474	0	34
468	GO:0001659	1	0.970287536	0	15
469	GO:0001660	1	0.993983759	0	3
470	GO:0001661	1	0.997981146	0	1
471	GO:0001662	1	0.960684189	0	20
472	GO:0001664	1	0.898503596	0	53
473	GO:0001665	1	0.987945917	0	6
474	GO:0001666	1	0.718325059	0	164
475	GO:0001667	1	0.986093992	0	7
476	GO:0001669	1	0.845659339	0	83
477	GO:0001671	1	0.952652756	0	24
478	GO:0001672	1	0.996030516	0	2
479	GO:0001673	1	0.976210234	0	12
480	GO:0001674	1	0.998013289	0	1
481	GO:0001675	1	0.972213589	0	14
482	GO:0001676	1	0.954781402	0	23

483	GO:0001678	1	0.956954489	0	22
484	GO:0001680	1	0.997983092	0	1
485	GO:0001681	1	0.99799195	0	1
486	GO:0001682	1	0.974037875	0	13
487	GO:0001691	1	0.995984509	0	2
488	GO:0001692	1	0.991966971	0	4
489	GO:0001694	1	0.99798523	0	1
490	GO:0001695	1	0.997958366	0	1
491	GO:0001696	1	0.985984516	0	7
492	GO:0001701	1	0.653700783	0	211
493	GO:0001702	1	0.974309865	0	13
494	GO:0001704	1	0.996030571	0	2
495	GO:0001705	1	0.997983203	0	1
496	GO:0001706	1	0.980035817	0	10
497	GO:0001707	1	0.9395235	0	31
498	GO:0001708	1	0.952954137	0	24
499	GO:0001709	1	0.972431447	0	14
500	GO:0001710	1	0.997993836	0	1
501	GO:0001711	1	0.988057733	0	6
502	GO:0001712	1	0.998003949	0	1
503	GO:0001714	1	0.993946746	0	3
504	GO:0001716	1	0.997979521	0	1
505	GO:0001725	1	0.872288406	0	68
506	GO:0001726	1	0.821406974	0	98
507	GO:0001727	1	0.9860698	0	7
508	GO:0001729	1	0.996008213	0	2
510	GO:0001731	1	0.978203221	0	11
511	GO:0001732	1	0.96817419	0	16
512	GO:0001733	1	0.995960114	0	2
513	GO:0001734	1	0.995972471	0	2
514	GO:0001735	1	0.996004691	0	2
515	GO:0001736	1	0.968469882	0	16
516	GO:0001738	1	0.986094767	0	7
517	GO:0001739	1	0.991979142	0	4
518	GO:0001740	1	0.992008642	0	4
519	GO:0001741	1	0.974203923	0	13
520	GO:0001750	1	0.882834364	0	62
521	GO:0001754	1	0.992048426	0	4
522	GO:0001755	1	0.917459716	0	43
523	GO:0001756	1	0.921016341	0	41
524	GO:0001757	1	0.992001668	0	4
525	GO:0001758	1	0.983946394	0	8
526	GO:0001759	1	0.984007567	0	8
527	GO:0001763	1	0.984088617	0	8
528	GO:0001764	1	0.828079756	0	94

529	GO:0001765	1	0.987965305	0	6
530	GO:0001766	1	0.995975826	0	2
531	GO:0001768	1	0.986054235	0	7
532	GO:0001770	1	0.998013437	0	1
533	GO:0001771	1	0.982105766	0	9
534	GO:0001772	1	0.92080521	0	41
535	GO:0001773	1	0.995962797	0	2
536	GO:0001774	1	0.95672537	0	22
537	GO:0001775	1	0.978107571	0	11
538	GO:0001776	1	0.987994863	0	6
539	GO:0001777	1	0.997956473	0	1
540	GO:0001778	1	0.976261054	0	12
541	GO:0001779	1	0.972412877	0	14
542	GO:0001780	1	0.986062605	0	7
543	GO:0001781	1	0.995958411	0	2
544	GO:0001782	1	0.958746469	0	21
545	GO:0001783	1	0.984057404	0	8
546	GO:0001784	1	0.921035056	0	41
547	GO:0001786	1	0.8989923	0	53
548	GO:0001787	1	0.998008808	0	1
549	GO:0001791	1	0.995976719	0	2
550	GO:0001792	1	0.998009857	0	1
551	GO:0001798	1	0.998013114	0	1
552	GO:0001805	1	0.997988572	0	1
553	GO:0001806	1	0.996007394	0	2
554	GO:0001808	1	0.997979299	0	1
555	GO:0001809	1	0.997967836	0	1
556	GO:0001811	1	0.997971783	0	1
557	GO:0001812	1	0.997988572	0	1
558	GO:0001814	1	0.997971783	0	1
559	GO:0001815	1	0.997973097	0	1
560	GO:0001817	1	0.924699084	0	39
561	GO:0001818	1	0.935871769	0	33
562	GO:0001819	1	0.886158134	0	60
563	GO:0001820	1	0.998012951	0	1
564	GO:0001821	1	0.993918701	0	3
565	GO:0001822	1	0.774505804	0	127
566	GO:0001823	1	0.97805794	0	11
567	GO:0001824	1	0.956491817	0	22
568	GO:0001825	1	0.962429679	0	19
569	GO:0001826	1	0.99402425	0	3
570	GO:0001827	1	0.995998174	0	2
571	GO:0001828	1	0.998012251	0	1
572	GO:0001829	1	0.974211198	0	13
573	GO:0001830	1	0.997972653	0	1

574	GO:0001831	1	0.998013437	0	1
575	GO:0001832	1	0.990037125	0	5
576	GO:0001833	1	0.9723214	0	14
577	GO:0001834	1	0.996007903	0	2
578	GO:0001835	1	0.948869068	0	26
580	GO:0001837	1	0.915561462	0	44
581	GO:0001838	1	0.994044278	0	3
582	GO:0001839	1	0.99396323	0	3
583	GO:0001840	1	0.99800067	0	1
584	GO:0001841	1	0.980147857	0	10
585	GO:0001842	1	0.99598822	0	2
586	GO:0001843	1	0.851675057	0	80
587	GO:0001844	1	0.985961424	0	7
588	GO:0001845	1	0.993972918	0	3
589	GO:0001846	1	0.998013437	0	1
590	GO:0001847	1	0.997971485	0	1
591	GO:0001848	1	0.990021057	0	5
592	GO:0001849	1	0.990020107	0	5
593	GO:0001850	1	0.997975698	0	1
594	GO:0001851	1	0.99201842	0	4
595	GO:0001855	1	0.995988779	0	2
596	GO:0001856	1	0.997984077	0	1
597	GO:0001861	1	0.998013437	0	1
598	GO:0001865	1	0.996017572	0	2
599	GO:0001866	1	0.995986379	0	2
600	GO:0001867	1	0.983919799	0	8
601	GO:0001869	1	0.995992719	0	2
602	GO:0001872	1	0.997985434	0	1
603	GO:0001875	1	0.990003299	0	5
604	GO:0001878	1	0.997983601	0	1
605	GO:0001880	1	0.994007383	0	3
606	GO:0001881	1	0.980093946	0	10
607	GO:0001882	1	0.993963241	0	3
608	GO:0001883	1	0.99396493	0	3
609	GO:0001885	1	0.991985709	0	4
610	GO:0001886	1	0.980255986	0	10
611	GO:0001887	1	0.990004694	0	5
612	GO:0001888	1	0.996009688	0	2
613	GO:0001889	1	0.847976645	0	82
614	GO:0001890	1	0.933810447	0	34
615	GO:0001891	1	0.949140784	0	26
616	GO:0001892	1	0.962637592	0	19
617	GO:0001893	1	0.984080429	0	8
618	GO:0001894	1	0.962689597	0	19
619	GO:0001895	1	0.943095155	0	29

620	GO:0001899	1	0.99798589	0	1
621	GO:0001906	1	0.997989689	0	1
622	GO:0001909	1	0.997976901	0	1
623	GO:0001910	1	0.997998115	0	1
624	GO:0001912	1	0.998009485	0	1
625	GO:0001913	1	0.987961999	0	6
626	GO:0001914	1	0.995948261	0	2
627	GO:0001915	1	0.986029728	0	7
628	GO:0001916	1	0.968138231	0	16
629	GO:0001917	1	0.902542797	0	51
630	GO:0001918	1	0.997991129	0	1
631	GO:0001919	1	0.989999613	0	5
632	GO:0001920	1	0.991995341	0	4
633	GO:0001921	1	0.97430129	0	13
634	GO:0001922	1	0.992032069	0	4
635	GO:0001923	1	0.995994761	0	2
636	GO:0001928	1	0.997982804	0	1
637	GO:0001931	1	0.976153612	0	12
638	GO:0001932	1	0.884496506	0	61
639	GO:0001933	1	0.853150308	0	79
640	GO:0001934	1	0.694107414	0	181
641	GO:0001935	1	0.970329266	0	15
642	GO:0001936	1	0.98807862	0	6
644	GO:0001938	1	0.877466002	0	65
645	GO:0001939	1	0.982076081	0	9
646	GO:0001940	1	0.988026706	0	6
647	GO:0001941	1	0.995943534	0	2
648	GO:0001942	1	0.932071387	0	35
649	GO:0001944	1	0.954852547	0	23
650	GO:0001945	1	0.976203147	0	12
651	GO:0001946	1	0.97821376	0	11
652	GO:0001947	1	0.902676864	0	51
653	GO:0001949	1	0.995991049	0	2
654	GO:0001951	1	0.994003409	0	3
655	GO:0001952	1	0.976277143	0	12
656	GO:0001953	1	0.970433894	0	15
657	GO:0001954	1	0.949108336	0	26
658	GO:0001955	1	0.984076045	0	8
659	GO:0001956	1	0.988018384	0	6
660	GO:0001957	1	0.986068228	0	7
661	GO:0001958	1	0.94924188	0	26
662	GO:0001959	1	0.986092578	0	7
663	GO:0001960	1	0.982069637	0	9
664	GO:0001961	1	0.980070877	0	10
665	GO:0001963	1	0.983949426	0	8

666	GO:0001964	1	0.970405344	0	15
667	GO:0001965	1	0.949012671	0	26
668	GO:0001966	1	0.998013437	0	1
669	GO:0001967	1	0.984128515	0	8
670	GO:0001968	1	0.951128625	0	25
671	GO:0001970	1	0.996030408	0	2
672	GO:0001971	1	0.996030731	0	2
673	GO:0001972	1	0.964392917	0	18
674	GO:0001973	1	0.986003471	0	7
675	GO:0001974	1	0.941622288	0	30
676	GO:0001975	1	0.943329041	0	29
677	GO:0001976	1	0.99600641	0	2
678	GO:0001980	1	0.997957119	0	1
679	GO:0001985	1	0.997981591	0	1
680	GO:0001992	1	0.991999512	0	4
681	GO:0001993	1	0.997979216	0	1
682	GO:0001994	1	0.995970166	0	2
683	GO:0001996	1	0.993968326	0	3
684	GO:0001997	1	0.995977703	0	2
685	GO:0001998	1	0.997977247	0	1
686	GO:0002001	1	0.995976333	0	2
687	GO:0002003	1	0.982057362	0	9
688	GO:0002009	1	0.962591596	0	19
689	GO:0002011	1	0.982209557	0	9
690	GO:0002016	1	0.995961158	0	2
691	GO:0002017	1	0.997976587	0	1
692	GO:0002018	1	0.995961158	0	2
693	GO:0002019	1	0.995991579	0	2
694	GO:0002020	1	0.825985912	0	95
695	GO:0002021	1	0.982091477	0	9
696	GO:0002023	1	0.993954774	0	3
697	GO:0002024	1	0.98209553	0	9
698	GO:0002025	1	0.995975333	0	2
699	GO:0002026	1	0.966386252	0	17
700	GO:0002027	1	0.945393497	0	28
701	GO:0002028	1	0.964470982	0	18
702	GO:0002029	1	0.993963946	0	3
703	GO:0002031	1	0.980013616	0	10
704	GO:0002032	1	0.995960222	0	2
705	GO:0002034	1	0.995974772	0	2
706	GO:0002035	1	0.998013437	0	1
707	GO:0002037	1	0.997980642	0	1
708	GO:0002038	1	0.996000269	0	2
709	GO:0002039	1	0.879348258	0	64
710	GO:0002040	1	0.939677478	0	31

711	GO:0002041	1	0.997990564	0	1
712	GO:0002042	1	0.960796374	0	20
713	GO:0002043	1	0.986051846	0	7
714	GO:0002046	1	0.993959666	0	3
715	GO:0002051	1	0.994032329	0	3
716	GO:0002052	1	0.958761688	0	21
718	GO:0002055	1	0.997955013	0	1
719	GO:0002058	1	0.99598094	0	2
720	GO:0002059	1	0.997980691	0	1
721	GO:0002060	1	0.995980648	0	2
722	GO:0002062	1	0.924787315	0	39
723	GO:0002063	1	0.964616366	0	18
724	GO:0002064	1	0.976287037	0	12
725	GO:0002066	1	0.998013437	0	1
726	GO:0002067	1	0.995988894	0	2
727	GO:0002068	1	0.992020887	0	4
728	GO:0002070	1	0.988010394	0	6
729	GO:0002071	1	0.997990564	0	1
730	GO:0002072	1	0.998001701	0	1
731	GO:0002074	1	0.992056089	0	4
732	GO:0002076	1	0.972309312	0	14
733	GO:0002077	1	0.997967422	0	1
734	GO:0002079	1	0.993975387	0	3
735	GO:0002080	1	0.960446526	0	20
736	GO:0002081	1	0.995992342	0	2
737	GO:0002082	1	0.976058325	0	12
738	GO:0002083	1	0.99797242	0	1
739	GO:0002084	1	0.980047096	0	10
740	GO:0002086	1	0.995961225	0	2
741	GO:0002087	1	0.980212793	0	10
742	GO:0002088	1	0.945363299	0	28
743	GO:0002089	1	0.978173872	0	11
744	GO:0002090	1	0.991966539	0	4
745	GO:0002091	1	0.978165826	0	11
746	GO:0002092	1	0.954934012	0	23
747	GO:0002093	1	0.992047862	0	4
748	GO:0002094	1	0.997998868	0	1
749	GO:0002095	1	0.995990685	0	2
750	GO:0002096	1	0.996012739	0	2
751	GO:0002098	1	0.968270799	0	16
752	GO:0002100	1	0.998013437	0	1
753	GO:0002101	1	0.997987966	0	1
754	GO:0002102	1	0.943519844	0	29
755	GO:0002112	1	0.997988898	0	1
756	GO:0002113	1	0.997983251	0	1

757	GO:0002114	1	0.995992743	0	2
758	GO:0002115	1	0.986049403	0	7
759	GO:0002116	1	0.978319035	0	11
760	GO:0002118	1	0.997998306	0	1
761	GO:0002121	1	0.996001542	0	2
762	GO:0002125	1	0.998008897	0	1
763	GO:0002127	1	0.997968677	0	1
764	GO:0002128	1	0.997977919	0	1
765	GO:0002133	1	0.996030299	0	2
766	GO:0002134	1	0.996004664	0	2
767	GO:0002135	1	0.997987328	0	1
768	GO:0002138	1	0.989975819	0	5
769	GO:0002141	1	0.99199718	0	4
770	GO:0002142	1	0.988034492	0	6
771	GO:0002143	1	0.991941704	0	4
772	GO:0002144	1	0.997980316	0	1
773	GO:0002151	1	0.9880656	0	6
774	GO:0002152	1	0.998000663	0	1
775	GO:0002153	1	0.995954609	0	2
776	GO:0002154	1	0.994023926	0	3
777	GO:0002155	1	0.993992132	0	3
778	GO:0002158	1	0.991969201	0	4
779	GO:0002159	1	0.992038679	0	4
780	GO:0002161	1	0.97814778	0	11
781	GO:0002162	1	0.980185039	0	10
782	GO:0002169	1	0.995995346	0	2
783	GO:0002175	1	0.994028905	0	3
784	GO:0002176	1	0.995974253	0	2
785	GO:0002177	1	0.970248893	0	15
786	GO:0002178	1	0.99397477	0	3
787	GO:0002181	1	0.838584874	0	86
788	GO:0002182	1	0.997949984	0	1
789	GO:0002183	1	0.966165042	0	17
790	GO:0002184	1	0.994021183	0	3
791	GO:0002188	1	0.992036	0	4
792	GO:0002189	1	0.991953592	0	4
793	GO:0002190	1	0.997972456	0	1
794	GO:0002191	1	0.99599506	0	2
795	GO:0002192	1	0.997972456	0	1
796	GO:0002193	1	0.994022951	0	3
797	GO:0002194	1	0.99799543	0	1
798	GO:0002196	1	0.993969914	0	3
799	GO:0002199	1	0.983950001	0	8
800	GO:0002204	1	0.995986829	0	2
801	GO:0002209	1	0.998013437	0	1

802	GO:0002215	1	0.998001552	0	1
803	GO:0002218	1	0.949094535	0	26
804	GO:0002220	1	0.997973997	0	1
805	GO:0002221	1	0.99002381	0	5
806	GO:0002223	1	0.813679918	0	102
807	GO:0002224	1	0.924500223	0	39
808	GO:0002227	1	0.968196489	0	16
809	GO:0002228	1	0.997997344	0	1
811	GO:0002232	1	0.991919857	0	4
812	GO:0002237	1	0.98199937	0	9
813	GO:0002238	1	0.997989906	0	1
814	GO:0002244	1	0.882830376	0	62
815	GO:0002246	1	0.997970777	0	1
816	GO:0002248	1	0.991988648	0	4
818	GO:0002251	1	0.997972608	0	1
820	GO:0002260	1	0.984126831	0	8
821	GO:0002262	1	0.980045726	0	10
822	GO:0002263	1	0.998013437	0	1
823	GO:0002264	1	0.997990564	0	1
824	GO:0002265	1	0.992035271	0	4
825	GO:0002266	1	0.997971783	0	1
826	GO:0002268	1	0.995975714	0	2
827	GO:0002269	1	0.993979208	0	3
828	GO:0002270	1	0.995983079	0	2
829	GO:0002276	1	0.995991663	0	2
830	GO:0002277	1	0.995975566	0	2
831	GO:0002280	1	0.996028655	0	2
832	GO:0002281	1	0.974210899	0	13
833	GO:0002282	1	0.993945846	0	3
834	GO:0002283	1	0.985965504	0	7
835	GO:0002286	1	0.983986368	0	8
836	GO:0002291	1	0.994032577	0	3
837	GO:0002292	1	0.995953994	0	2
838	GO:0002295	1	0.99799807	0	1
839	GO:0002296	1	0.994045946	0	3
840	GO:0002305	1	0.997986633	0	1
841	GO:0002309	1	0.991967058	0	4
842	GO:0002312	1	0.995935909	0	2
843	GO:0002313	1	0.993920784	0	3
844	GO:0002314	1	0.989957845	0	5
845	GO:0002315	1	0.982132005	0	9
846	GO:0002316	1	0.995985508	0	2
847	GO:0002317	1	0.997951264	0	1
848	GO:0002318	1	0.988038641	0	6
849	GO:0002319	1	0.998013437	0	1

850	GO:0002320	1	0.987956298	0	6
851	GO:0002322	1	0.990041749	0	5
852	GO:0002323	1	0.995930304	0	2
853	GO:0002326	1	0.992040569	0	4
854	GO:0002327	1	0.992056903	0	4
855	GO:0002328	1	0.992063417	0	4
856	GO:0002329	1	0.996027631	0	2
857	GO:0002331	1	0.996030731	0	2
858	GO:0002333	1	0.99801343	0	1
859	GO:0002334	1	0.997989208	0	1
860	GO:0002335	1	0.998011172	0	1
861	GO:0002337	1	0.995978147	0	2
862	GO:0002344	1	0.993954387	0	3
863	GO:0002347	1	0.997958366	0	1
864	GO:0002352	1	0.995942265	0	2
865	GO:0002353	1	0.995965066	0	2
866	GO:0002355	1	0.99798545	0	1
867	GO:0002357	1	0.986024	0	7
868	GO:0002358	1	0.997956473	0	1
869	GO:0002360	1	0.990016218	0	5
870	GO:0002361	1	0.995976488	0	2
871	GO:0002362	1	0.99599672	0	2
872	GO:0002363	1	0.997977247	0	1
873	GO:0002364	1	0.998008767	0	1
874	GO:0002366	1	0.993997318	0	3
876	GO:0002383	1	0.997972608	0	1
877	GO:0002384	1	0.995976565	0	2
878	GO:0002385	1	0.989984354	0	5
879	GO:0002395	1	0.995970264	0	2
880	GO:0002397	1	0.998001741	0	1
881	GO:0002398	1	0.998001741	0	1
882	GO:0002399	1	0.997967215	0	1
883	GO:0002407	1	0.978090669	0	11
884	GO:0002408	1	0.99593926	0	2
885	GO:0002412	1	0.99798205	0	1
886	GO:0002415	1	0.995991114	0	2
887	GO:0002416	1	0.997974565	0	1
888	GO:0002418	1	0.995964423	0	2
889	GO:0002419	1	0.997970795	0	1
890	GO:0002429	1	0.995980832	0	2
891	GO:0002430	1	0.984012237	0	8
892	GO:0002431	1	0.995951379	0	2
893	GO:0002432	1	0.998010722	0	1
894	GO:0002434	1	0.997991997	0	1
895	GO:0002435	1	0.998013437	0	1

896	GO:0002436	1	0.997971783	0	1
897	GO:0002437	1	0.960513239	0	20
898	GO:0002438	1	0.987979989	0	6
899	GO:0002439	1	0.995960208	0	2
900	GO:0002446	1	0.982053483	0	9
901	GO:0002447	1	0.997989208	0	1
902	GO:0002448	1	0.995988315	0	2
903	GO:0002451	1	0.99797134	0	1
904	GO:0002455	1	0.991935428	0	4
905	GO:0002456	1	0.978171076	0	11
906	GO:0002457	1	0.992002144	0	4
907	GO:0002458	1	0.997980488	0	1
908	GO:0002460	1	0.997981889	0	1
909	GO:0002467	1	0.984087599	0	8
910	GO:0002469	1	0.995932223	0	2
911	GO:0002470	1	0.997963674	0	1
912	GO:0002474	1	0.949038029	0	26
913	GO:0002476	1	0.991899816	0	4
914	GO:0002477	1	0.995942956	0	2
915	GO:0002479	1	0.864043463	0	72
916	GO:0002480	1	0.983904776	0	8
917	GO:0002481	1	0.997959982	0	1
918	GO:0002485	1	0.997970795	0	1
919	GO:0002486	1	0.993924211	0	3
920	GO:0002489	1	0.998007827	0	1
921	GO:0002491	1	0.995932223	0	2
922	GO:0002495	1	0.995985075	0	2
923	GO:0002502	1	0.995956708	0	2
924	GO:0002503	1	0.991882391	0	4
925	GO:0002504	1	0.97390754	0	13
926	GO:0002505	1	0.997994144	0	1
927	GO:0002507	1	0.993948714	0	3
928	GO:0002508	1	0.997988743	0	1
929	GO:0002509	1	0.997980488	0	1
930	GO:0002513	1	0.991966207	0	4
931	GO:0002517	1	0.997988821	0	1
932	GO:0002518	1	0.997964216	0	1
933	GO:0002519	1	0.99394189	0	3
934	GO:0002520	1	0.990057692	0	5
935	GO:0002521	1	0.991977576	0	4
936	GO:0002522	1	0.997981211	0	1
937	GO:0002523	1	0.976129068	0	12
938	GO:0002524	1	0.997999867	0	1
939	GO:0002526	1	0.976059957	0	12
940	GO:0002528	1	0.992003017	0	4

941	GO:0002532	1	0.993964393	0	3
942	GO:0002536	1	0.997957442	0	1
943	GO:0002537	1	0.998013431	0	1
944	GO:0002540	1	0.995949306	0	2
945	GO:0002541	1	0.997984299	0	1
946	GO:0002542	1	0.995966318	0	2
947	GO:0002543	1	0.996000556	0	2
948	GO:0002544	1	0.983959616	0	8
949	GO:0002548	1	0.952414271	0	24
950	GO:0002551	1	0.992013946	0	4
951	GO:0002553	1	0.993978141	0	3
952	GO:0002554	1	0.998012951	0	1
953	GO:0002561	1	0.998009198	0	1
954	GO:0002566	1	0.998013437	0	1
955	GO:0002572	1	0.997995407	0	1
956	GO:0002573	1	0.994051614	0	3
957	GO:0002575	1	0.998001964	0	1
958	GO:0002576	1	0.79773085	0	112
959	GO:0002577	1	0.997998144	0	1
960	GO:0002579	1	0.99796466	0	1
961	GO:0002581	1	0.998013437	0	1
962	GO:0002587	1	0.995972891	0	2
963	GO:0002588	1	0.995923521	0	2
964	GO:0002590	1	0.997975526	0	1
965	GO:0002605	1	0.991994045	0	4
966	GO:0002606	1	0.987989225	0	6
967	GO:0002617	1	0.998009752	0	1
968	GO:0002622	1	0.997971783	0	1
969	GO:0002625	1	0.997975818	0	1
970	GO:0002626	1	0.997977701	0	1
971	GO:0002634	1	0.990073406	0	5
972	GO:0002635	1	0.996006085	0	2
973	GO:0002636	1	0.99593831	0	2
974	GO:0002637	1	0.983967653	0	8
975	GO:0002638	1	0.984041586	0	8
976	GO:0002639	1	0.96236806	0	19
977	GO:0002643	1	0.995995133	0	2
978	GO:0002645	1	0.99797134	0	1
979	GO:0002651	1	0.99801192	0	1
980	GO:0002652	1	0.997983824	0	1
981	GO:0002663	1	0.99801192	0	1
982	GO:0002664	1	0.997997686	0	1
983	GO:0002666	1	0.993979138	0	3
984	GO:0002667	1	0.993980505	0	3
985	GO:0002669	1	0.989994509	0	5

986	GO:0002673	1	0.991942644	0	4
987	GO:0002674	1	0.989990321	0	5
988	GO:0002675	1	0.984014887	0	8
989	GO:0002676	1	0.997964679	0	1
990	GO:0002677	1	0.995998762	0	2
991	GO:0002679	1	0.996030578	0	2
992	GO:0002682	1	0.987982167	0	6
993	GO:0002683	1	0.998006804	0	1
994	GO:0002684	1	0.995992945	0	2
995	GO:0002685	1	0.988006017	0	6
996	GO:0002686	1	0.989952403	0	5
997	GO:0002687	1	0.978133383	0	11
998	GO:0002688	1	0.998010325	0	1
999	GO:0002689	1	0.996025449	0	2
1001	GO:0002691	1	0.997969548	0	1
1002	GO:0002692	1	0.997998379	0	1
1003	GO:0002693	1	0.990031533	0	5
1004	GO:0002695	1	0.995977234	0	2
1005	GO:0002710	1	0.998011136	0	1
1006	GO:0002711	1	0.994001904	0	3
1007	GO:0002715	1	0.997973097	0	1
1008	GO:0002717	1	0.995964241	0	2
1009	GO:0002718	1	0.994026722	0	3
1010	GO:0002719	1	0.989945575	0	5
1011	GO:0002720	1	0.972274914	0	14
1012	GO:0002721	1	0.997988572	0	1
1013	GO:0002724	1	0.996016133	0	2
1014	GO:0002725	1	0.989943072	0	5
1015	GO:0002726	1	0.982055066	0	9
1016	GO:0002728	1	0.995980781	0	2
1017	GO:0002729	1	0.985889782	0	7
1018	GO:0002730	1	0.996012295	0	2
1019	GO:0002731	1	0.998013384	0	1
1020	GO:0002732	1	0.992023734	0	4
1022	GO:0002737	1	0.997959046	0	1
1023	GO:0002747	1	0.997994144	0	1
1024	GO:0002752	1	0.99594917	0	2
1025	GO:0002753	1	0.978108187	0	11
1026	GO:0002755	1	0.943366881	0	29
1027	GO:0002756	1	0.960506858	0	20
1028	GO:0002757	1	0.997989968	0	1
1029	GO:0002758	1	0.997981211	0	1
1030	GO:0002759	1	0.995981507	0	2
1031	GO:0002761	1	0.998002986	0	1
1032	GO:0002762	1	0.993921005	0	3

1033	GO:0002763	1	0.996019605	0	2
1034	GO:0002764	1	0.995954122	0	2
1035	GO:0002765	1	0.995948993	0	2
1036	GO:0002767	1	0.995967596	0	2
1037	GO:0002768	1	0.987967917	0	6
1038	GO:0002774	1	0.993979546	0	3
1039	GO:0002787	1	0.998003862	0	1
1040	GO:0002790	1	0.99602609	0	2
1041	GO:0002792	1	0.997966363	0	1
1042	GO:0002793	1	0.995944091	0	2
1043	GO:0002803	1	0.993923436	0	3
1045	GO:0002820	1	0.995987388	0	2
1046	GO:0002821	1	0.987981571	0	6
1047	GO:0002826	1	0.993967866	0	3
1048	GO:0002827	1	0.982002726	0	9
1049	GO:0002828	1	0.997977936	0	1
1050	GO:0002829	1	0.990028382	0	5
1051	GO:0002830	1	0.99001705	0	5
1052	GO:0002838	1	0.997983824	0	1
1053	GO:0002839	1	0.997977819	0	1
1054	GO:0002840	1	0.998000642	0	1
1055	GO:0002841	1	0.995996881	0	2
1056	GO:0002842	1	0.993968498	0	3
1057	GO:0002845	1	0.997970594	0	1
1058	GO:0002851	1	0.99798403	0	1
1059	GO:0002854	1	0.995957069	0	2
1060	GO:0002857	1	0.997970959	0	1
1061	GO:0002859	1	0.995989082	0	2
1062	GO:0002860	1	0.989986356	0	5
1063	GO:0002862	1	0.837326113	0	88
1064	GO:0002863	1	0.993977789	0	3
1065	GO:0002864	1	0.997988495	0	1
1066	GO:0002865	1	0.99596633	0	2
1067	GO:0002866	1	0.995976346	0	2
1068	GO:0002875	1	0.997972205	0	1
1069	GO:0002876	1	0.997972866	0	1
1070	GO:0002879	1	0.997979381	0	1
1071	GO:0002885	1	0.997981373	0	1
1072	GO:0002891	1	0.993992669	0	3
1073	GO:0002897	1	0.997988743	0	1
1074	GO:0002902	1	0.994009079	0	3
1075	GO:0002903	1	0.986050479	0	7
1076	GO:0002904	1	0.993954062	0	3
1077	GO:0002906	1	0.993944821	0	3
1078	GO:0002920	1	0.995978544	0	2

1079	GO:0002922	1	0.993933022	0	3
1080	GO:0002923	1	0.998012965	0	1
1081	GO:0002924	1	0.993955257	0	3
1082	GO:0002925	1	0.991992761	0	4
1083	GO:0002926	1	0.995992301	0	2
1084	GO:0002930	1	0.995995263	0	2
1085	GO:0002931	1	0.889878348	0	58
1086	GO:0002933	1	0.985939824	0	7
1087	GO:0002934	1	0.988099235	0	6
1088	GO:0002939	1	0.99600774	0	2
1089	GO:0002940	1	0.995976208	0	2
1090	GO:0002943	1	0.989954366	0	5
1091	GO:0002944	1	0.996000405	0	2
1092	GO:0002945	1	0.996000405	0	2
1093	GO:0002946	1	0.99798556	0	1
1094	GO:0002947	1	0.991962516	0	4
1095	GO:0002949	1	0.993921977	0	3
1096	GO:0002950	1	0.994000085	0	3
1097	GO:0002951	1	0.995975104	0	2
1098	GO:0002953	1	0.997971972	0	1
1099	GO:0003002	1	0.998013437	0	1
1100	GO:0003006	1	0.990009672	0	5
1101	GO:0003007	1	0.906429565	0	49
1102	GO:0003009	1	0.956531441	0	22
1103	GO:0003011	1	0.998012955	0	1
1104	GO:0003012	1	0.995945553	0	2
1105	GO:0003014	1	0.982104699	0	9
1106	GO:0003015	1	0.988013621	0	6
1107	GO:0003016	1	0.974296066	0	13
1108	GO:0003017	1	0.996030731	0	2
1109	GO:0003025	1	0.998013437	0	1
1110	GO:0003026	1	0.997990564	0	1
1111	GO:0003032	1	0.99599577	0	2
1112	GO:0003044	1	0.997983808	0	1
1113	GO:0003050	1	0.996013969	0	2
1114	GO:0003051	1	0.995993757	0	2
1115	GO:0003056	1	0.992022962	0	4
1116	GO:0003057	1	0.997989294	0	1
1117	GO:0003058	1	0.997964968	0	1
1118	GO:0003062	1	0.996027698	0	2
1119	GO:0003063	1	0.997990402	0	1
1120	GO:0003064	1	0.995977217	0	2
1121	GO:0003065	1	0.997975043	0	1
1122	GO:0003069	1	0.997960735	0	1
1123	GO:0003073	1	0.978126667	0	11

1124	GO:0003081	1	0.990027655	0	5
1125	GO:0003084	1	0.976116661	0	12
1126	GO:0003085	1	0.986043334	0	7
1127	GO:0003091	1	0.93972095	0	31
1128	GO:0003093	1	0.992035272	0	4
1129	GO:0003094	1	0.982172488	0	9
1130	GO:0003095	1	0.993958048	0	3
1131	GO:0003096	1	0.995960383	0	2
1132	GO:0003097	1	0.996003563	0	2
1133	GO:0003099	1	0.997964968	0	1
1134	GO:0003100	1	0.989961239	0	5
1135	GO:0003104	1	0.99199165	0	4
1136	GO:0003105	1	0.994021911	0	3
1137	GO:0003106	1	0.997953993	0	1
1138	GO:0003108	1	0.997990564	0	1
1139	GO:0003117	1	0.997984157	0	1
1140	GO:0003127	1	0.994012749	0	3
1141	GO:0003128	1	0.997999867	0	1
1142	GO:0003130	1	0.993951509	0	3
1143	GO:0003131	1	0.997991726	0	1
1144	GO:0003136	1	0.997993311	0	1
1145	GO:0003138	1	0.994015943	0	3
1146	GO:0003139	1	0.97811049	0	11
1147	GO:0003140	1	0.993961885	0	3
1148	GO:0003143	1	0.986030037	0	7
1149	GO:0003147	1	0.997961189	0	1
1150	GO:0003148	1	0.953067306	0	24
1151	GO:0003149	1	0.986036399	0	7
1152	GO:0003150	1	0.993992771	0	3
1153	GO:0003151	1	0.919046618	0	42
1154	GO:0003156	1	0.997975026	0	1
1155	GO:0003157	1	0.992026448	0	4
1156	GO:0003158	1	0.990007834	0	5
1157	GO:0003160	1	0.996001752	0	2
1158	GO:0003161	1	0.972298776	0	14
1159	GO:0003162	1	0.992065124	0	4
1160	GO:0003163	1	0.990011888	0	5
1161	GO:0003165	1	0.996000933	0	2
1162	GO:0003166	1	0.995939593	0	2
1163	GO:0003167	1	0.995999746	0	2
1164	GO:0003169	1	0.99601928	0	2
1165	GO:0003170	1	0.980230139	0	10
1166	GO:0003171	1	0.995996517	0	2
1167	GO:0003174	1	0.997996859	0	1
1168	GO:0003176	1	0.990019648	0	5

1169	GO:0003177	1	0.992007372	0	4
1170	GO:0003179	1	0.986096865	0	7
1171	GO:0003180	1	0.949238344	0	26
1172	GO:0003181	1	0.978278895	0	11
1173	GO:0003182	1	0.998013437	0	1
1174	GO:0003183	1	0.986081244	0	7
1175	GO:0003184	1	0.970436306	0	15
1176	GO:0003185	1	0.998013437	0	1
1177	GO:0003186	1	0.992041271	0	4
1178	GO:0003188	1	0.996001283	0	2
1179	GO:0003190	1	0.993987728	0	3
1180	GO:0003192	1	0.994036568	0	3
1181	GO:0003193	1	0.997996859	0	1
1182	GO:0003195	1	0.99599129	0	2
1183	GO:0003197	1	0.988006752	0	6
1184	GO:0003198	1	0.982061549	0	9
1185	GO:0003199	1	0.995963695	0	2
1186	GO:0003203	1	0.968469323	0	16
1187	GO:0003205	1	0.995991461	0	2
1188	GO:0003207	1	0.998013437	0	1
1189	GO:0003208	1	0.984121074	0	8
1190	GO:0003209	1	0.988091734	0	6
1191	GO:0003210	1	0.995963977	0	2
1192	GO:0003211	1	0.994001151	0	3
1193	GO:0003213	1	0.996013982	0	2
1194	GO:0003214	1	0.986027105	0	7
1195	GO:0003215	1	0.980183058	0	10
1196	GO:0003219	1	0.995990664	0	2
1197	GO:0003220	1	0.996030731	0	2
1198	GO:0003221	1	0.998011079	0	1
1199	GO:0003222	1	0.974322594	0	13
1200	GO:0003223	1	0.988091939	0	6
1201	GO:0003226	1	0.998013437	0	1
1202	GO:0003228	1	0.997972608	0	1
1203	GO:0003229	1	0.994018232	0	3
1204	GO:0003231	1	0.988054726	0	6
1205	GO:0003236	1	0.997963363	0	1
1206	GO:0003241	1	0.995980754	0	2
1207	GO:0003245	1	0.997995094	0	1
1208	GO:0003247	1	0.997972367	0	1
1209	GO:0003251	1	0.998013437	0	1
1210	GO:0003252	1	0.996030731	0	2
1211	GO:0003253	1	0.995947645	0	2
1212	GO:0003254	1	0.988035691	0	6
1213	GO:0003256	1	0.994009591	0	3

1214	GO:0003257	1	0.996022732	0	2
1215	GO:0003259	1	0.997963363	0	1
1216	GO:0003260	1	0.997963363	0	1
1217	GO:0003264	1	0.998013437	0	1
1218	GO:0003266	1	0.993971444	0	3
1219	GO:0003270	1	0.998013437	0	1
1220	GO:0003271	1	0.997985143	0	1
1221	GO:0003272	1	0.986128233	0	7
1222	GO:0003273	1	0.992026917	0	4
1223	GO:0003274	1	0.994030525	0	3
1224	GO:0003278	1	0.997990564	0	1
1225	GO:0003279	1	0.972332936	0	14
1226	GO:0003281	1	0.936086124	0	33
1227	GO:0003283	1	0.98412414	0	8
1228	GO:0003289	1	0.992033479	0	4
1229	GO:0003290	1	0.997990564	0	1
1230	GO:0003300	1	0.982092703	0	9
1231	GO:0003308	1	0.996013952	0	2
1232	GO:0003309	1	0.988012387	0	6
1233	GO:0003310	1	0.995997181	0	2
1234	GO:0003322	1	0.998013437	0	1
1235	GO:0003323	1	0.976213641	0	12
1236	GO:0003330	1	0.997990564	0	1
1237	GO:0003331	1	0.989957429	0	5
1238	GO:0003332	1	0.992013499	0	4
1239	GO:0003333	1	0.958712626	0	21
1240	GO:0003334	1	0.982124767	0	9
1241	GO:0003335	1	0.997966629	0	1
1242	GO:0003336	1	0.996004641	0	2
1243	GO:0003337	1	0.987997037	0	6
1244	GO:0003338	1	0.994032415	0	3
1245	GO:0003339	1	0.997969178	0	1
1247	GO:0003341	1	0.926424799	0	38
1248	GO:0003342	1	0.997994666	0	1
1249	GO:0003344	1	0.988119663	0	6
1250	GO:0003345	1	0.99800626	0	1
1251	GO:0003350	1	0.998013178	0	1
1252	GO:0003351	1	0.958603067	0	21
1253	GO:0003352	1	0.989976387	0	5
1254	GO:0003353	1	0.997994443	0	1
1255	GO:0003356	1	0.988059863	0	6
1256	GO:0003357	1	0.99801259	0	1
1257	GO:0003358	1	0.995981797	0	2
1258	GO:0003359	1	0.997986219	0	1
1259	GO:0003360	1	0.990060331	0	5

1260	GO:0003363	1	0.998013437	0	1
1261	GO:0003365	1	0.994051303	0	3
1262	GO:0003366	1	0.996008328	0	2
1264	GO:0003376	1	0.974232621	0	13
1265	GO:0003382	1	0.966492121	0	17
1266	GO:0003383	1	0.996030731	0	2
1267	GO:0003400	1	0.991990129	0	4
1268	GO:0003401	1	0.996025937	0	2
1269	GO:0003402	1	0.994015943	0	3
1270	GO:0003404	1	0.995989749	0	2
1271	GO:0003406	1	0.991989076	0	4
1272	GO:0003407	1	0.968442544	0	16
1273	GO:0003408	1	0.996011598	0	2
1274	GO:0003409	1	0.99799889	0	1
1275	GO:0003412	1	0.998013437	0	1
1276	GO:0003413	1	0.990058414	0	5
1277	GO:0003415	1	0.996019466	0	2
1278	GO:0003416	1	0.984115676	0	8
1279	GO:0003417	1	0.982116197	0	9
1280	GO:0003418	1	0.993962799	0	3
1281	GO:0003419	1	0.997947599	0	1
1282	GO:0003420	1	0.997986766	0	1
1283	GO:0003421	1	0.998006297	0	1
1284	GO:0003429	1	0.998005803	0	1
1285	GO:0003430	1	0.994022214	0	3
1286	GO:0003431	1	0.996007161	0	2
1287	GO:0003433	1	0.995999091	0	2
1291	GO:0003678	1	0.886757315	0	60
1292	GO:0003680	1	0.984081942	0	8
1293	GO:0003681	1	0.995966387	0	2
1295	GO:0003684	1	0.882783821	0	62
1296	GO:0003688	1	0.954874087	0	23
1297	GO:0003689	1	0.982019443	0	9
1299	GO:0003691	1	0.982062485	0	9
1300	GO:0003692	1	0.997981623	0	1
1301	GO:0003696	1	0.991993167	0	4
1302	GO:0003697	1	0.802927212	0	109
1304	GO:0003707	1	0.954953873	0	23
1305	GO:0003711	1	0.99200636	0	4
1306	GO:0003712	1	0.774377208	0	127
1307	GO:0003713	1	0.628659834	0	230
1308	GO:0003714	1	0.692826218	0	182
1309	GO:0003720	1	0.988031062	0	6
1310	GO:0003721	1	0.99800762	0	1
1314	GO:0003726	1	0.994041559	0	3

1316	GO:0003729	1	0.667892027	0	200
1317	GO:0003730	1	0.858189085	0	76
1318	GO:0003735	1	0.722163019	0	159
1319	GO:0003743	1	0.895057826	0	55
1320	GO:0003746	1	0.948912633	0	26
1321	GO:0003747	1	0.986041486	0	7
1322	GO:0003755	1	0.922208107	0	40
1323	GO:0003756	1	0.96445478	0	18
1325	GO:0003777	1	0.892261695	0	57
1327	GO:0003785	1	0.948853346	0	26
1328	GO:0003796	1	0.993899059	0	3
1329	GO:0003810	1	0.989996246	0	5
1330	GO:0003823	1	0.97209043	0	14
1332	GO:0003826	1	0.995967523	0	2
1333	GO:0003827	1	0.997993157	0	1
1334	GO:0003828	1	0.992001873	0	4
1335	GO:0003829	1	0.994008077	0	3
1336	GO:0003830	1	0.998012929	0	1
1337	GO:0003831	1	0.991965082	0	4
1338	GO:0003834	1	0.99598669	0	2
1339	GO:0003835	1	0.996027171	0	2
1340	GO:0003836	1	0.99200446	0	4
1341	GO:0003837	1	0.997979776	0	1
1342	GO:0003839	1	0.993910875	0	3
1343	GO:0003841	1	0.974319092	0	13
1344	GO:0003842	1	0.997998159	0	1
1345	GO:0003844	1	0.997996052	0	1
1346	GO:0003845	1	0.995948806	0	2
1347	GO:0003846	1	0.989971895	0	5
1348	GO:0003847	1	0.983915758	0	8
1349	GO:0003851	1	0.997992871	0	1
1350	GO:0003853	1	0.998013437	0	1
1351	GO:0003854	1	0.991958571	0	4
1352	GO:0003857	1	0.983950977	0	8
1353	GO:0003858	1	0.995997361	0	2
1354	GO:0003860	1	0.997977424	0	1
1355	GO:0003863	1	0.995967523	0	2
1356	GO:0003865	1	0.993969634	0	3
1357	GO:0003867	1	0.998012706	0	1
1358	GO:0003868	1	0.997975147	0	1
1359	GO:0003870	1	0.997984093	0	1
1360	GO:0003872	1	0.993995087	0	3
1361	GO:0003873	1	0.992023081	0	4
1362	GO:0003874	1	0.997958946	0	1
1363	GO:0003875	1	0.995979828	0	2

1364	GO:0003876	1	0.994012009	0	3
1365	GO:0003877	1	0.997990564	0	1
1366	GO:0003878	1	0.998010385	0	1
1367	GO:0003880	1	0.995982348	0	2
1368	GO:0003881	1	0.997976485	0	1
1369	GO:0003882	1	0.997985858	0	1
1370	GO:0003883	1	0.996001528	0	2
1371	GO:0003884	1	0.997972026	0	1
1372	GO:0003886	1	0.994042001	0	3
1373	GO:0003887	1	0.95484333	0	23
1374	GO:0003896	1	0.995963749	0	2
1375	GO:0003899	1	0.924255522	0	39
1376	GO:0003905	1	0.997961976	0	1
1377	GO:0003906	1	0.979964165	0	10
1378	GO:0003908	1	0.997965447	0	1
1379	GO:0003909	1	0.994015992	0	3
1380	GO:0003910	1	0.994015992	0	3
1381	GO:0003916	1	0.988071939	0	6
1382	GO:0003917	1	0.992010431	0	4
1383	GO:0003918	1	0.99603051	0	2
1384	GO:0003919	1	0.997978145	0	1
1386	GO:0003921	1	0.997986078	0	1
1387	GO:0003922	1	0.997986078	0	1
1388	GO:0003923	1	0.995995505	0	2
1390	GO:0003925	1	0.939359086	0	31
1391	GO:0003934	1	0.997977827	0	1
1392	GO:0003937	1	0.997980218	0	1
1393	GO:0003938	1	0.993948798	0	3
1394	GO:0003939	1	0.997987078	0	1
1395	GO:0003940	1	0.997981777	0	1
1396	GO:0003941	1	0.99393812	0	3
1397	GO:0003943	1	0.995997189	0	2
1398	GO:0003944	1	0.997981986	0	1
1399	GO:0003945	1	0.990002302	0	5
1400	GO:0003947	1	0.99799462	0	1
1401	GO:0003948	1	0.997980071	0	1
1402	GO:0003950	1	0.950976738	0	25
1403	GO:0003951	1	0.968504061	0	16
1404	GO:0003952	1	0.997985749	0	1
1405	GO:0003953	1	0.972296546	0	14
1406	GO:0003954	1	0.97388786	0	13
1407	GO:0003955	1	0.993962448	0	3
1408	GO:0003956	1	0.993922529	0	3
1409	GO:0003957	1	0.998011963	0	1
1410	GO:0003958	1	0.994009791	0	3

1411	GO:0003960	1	0.991926859	0	4
1412	GO:0003963	1	0.997990564	0	1
1413	GO:0003964	1	0.99800762	0	1
1414	GO:0003968	1	0.99800762	0	1
1415	GO:0003972	1	0.997990564	0	1
1416	GO:0003973	1	0.998013437	0	1
1417	GO:0003974	1	0.997971918	0	1
1418	GO:0003975	1	0.997980984	0	1
1419	GO:0003976	1	0.993971451	0	3
1420	GO:0003977	1	0.99598776	0	2
1421	GO:0003978	1	0.997971918	0	1
1422	GO:0003979	1	0.997997344	0	1
1423	GO:0003980	1	0.996030099	0	2
1424	GO:0003983	1	0.997980968	0	1
1425	GO:0003984	1	0.997983585	0	1
1426	GO:0003985	1	0.989939888	0	5
1427	GO:0003986	1	0.989894057	0	5
1428	GO:0003987	1	0.992019137	0	4
1429	GO:0003988	1	0.98993842	0	5
1430	GO:0003989	1	0.994014214	0	3
1431	GO:0003990	1	0.997987865	0	1
1432	GO:0003993	1	0.983925456	0	8
1433	GO:0003994	1	0.994018204	0	3
1434	GO:0003995	1	0.982045603	0	9
1435	GO:0003997	1	0.990005041	0	5
1436	GO:0003998	1	0.995921708	0	2
1437	GO:0003999	1	0.997955013	0	1
1438	GO:0004000	1	0.984069197	0	8
1439	GO:0004001	1	0.997979513	0	1
1440	GO:0004013	1	0.994015924	0	3
1441	GO:0004014	1	0.997998357	0	1
1442	GO:0004016	1	0.980260302	0	10
1443	GO:0004017	1	0.98205089	0	9
1444	GO:0004018	1	0.997969493	0	1
1445	GO:0004019	1	0.995985075	0	2
1446	GO:0004020	1	0.995997377	0	2
1447	GO:0004021	1	0.995987498	0	2
1448	GO:0004022	1	0.983932098	0	8
1449	GO:0004024	1	0.985940876	0	7
1450	GO:0004027	1	0.995947142	0	2
1451	GO:0004028	1	0.989997213	0	5
1452	GO:0004029	1	0.972272217	0	14
1453	GO:0004030	1	0.989980608	0	5
1454	GO:0004031	1	0.993993087	0	3
1455	GO:0004032	1	0.975918528	0	12

1456	GO:0004033	1	0.977955286	0	11
1457	GO:0004034	1	0.997986375	0	1
1458	GO:0004035	1	0.995984251	0	2
1459	GO:0004040	1	0.995951767	0	2
1460	GO:0004042	1	0.997979809	0	1
1461	GO:0004043	1	0.998002542	0	1
1462	GO:0004044	1	0.998004859	0	1
1463	GO:0004045	1	0.991917883	0	4
1464	GO:0004046	1	0.987926835	0	6
1465	GO:0004047	1	0.995953763	0	2
1466	GO:0004051	1	0.995949306	0	2
1467	GO:0004052	1	0.991972127	0	4
1468	GO:0004053	1	0.997978287	0	1
1469	GO:0004055	1	0.997975673	0	1
1470	GO:0004056	1	0.997978487	0	1
1471	GO:0004057	1	0.997982115	0	1
1472	GO:0004058	1	0.997978943	0	1
1473	GO:0004059	1	0.997969289	0	1
1474	GO:0004060	1	0.993926613	0	3
1475	GO:0004061	1	0.997973416	0	1
1476	GO:0004062	1	0.983886453	0	8
1477	GO:0004063	1	0.995942509	0	2
1478	GO:0004064	1	0.987899875	0	6
1479	GO:0004065	1	0.972307327	0	14
1480	GO:0004066	1	0.995974059	0	2
1481	GO:0004067	1	0.995964896	0	2
1482	GO:0004068	1	0.995997587	0	2
1483	GO:0004069	1	0.995970589	0	2
1484	GO:0004070	1	0.998013437	0	1
1485	GO:0004074	1	0.995923309	0	2
1486	GO:0004075	1	0.997987359	0	1
1487	GO:0004077	1	0.998013437	0	1
1488	GO:0004078	1	0.998013437	0	1
1489	GO:0004079	1	0.998013437	0	1
1490	GO:0004080	1	0.998013437	0	1
1491	GO:0004081	1	0.995954612	0	2
1492	GO:0004082	1	0.991905896	0	4
1493	GO:0004083	1	0.997990564	0	1
1494	GO:0004084	1	0.995989427	0	2
1495	GO:0004085	1	0.995992972	0	2
1496	GO:0004087	1	0.998013437	0	1
1497	GO:0004088	1	0.996030731	0	2
1498	GO:0004089	1	0.974075635	0	13
1499	GO:0004090	1	0.989863631	0	5
1500	GO:0004092	1	0.997991641	0	1

1501	GO:0004095	1	0.992007031	0	4
1502	GO:0004096	1	0.989973124	0	5
1503	GO:0004098	1	0.997976994	0	1
1504	GO:0004103	1	0.995965947	0	2
1505	GO:0004104	1	0.995981469	0	2
1506	GO:0004105	1	0.995988715	0	2
1507	GO:0004108	1	0.997994098	0	1
1508	GO:0004109	1	0.995980503	0	2
1509	GO:0004111	1	0.987932202	0	6
1510	GO:0004112	1	0.996030731	0	2
1511	GO:0004113	1	0.998013218	0	1
1512	GO:0004114	1	0.958863431	0	21
1513	GO:0004115	1	0.970514792	0	15
1514	GO:0004117	1	0.993982997	0	3
1515	GO:0004118	1	0.994047774	0	3
1516	GO:0004119	1	0.99602607	0	2
1517	GO:0004122	1	0.99598273	0	2
1518	GO:0004123	1	0.997974251	0	1
1519	GO:0004126	1	0.977957735	0	11
1521	GO:0004128	1	0.985975005	0	7
1522	GO:0004129	1	0.954171429	0	23
1523	GO:0004132	1	0.997979109	0	1
1524	GO:0004133	1	0.998013437	0	1
1525	GO:0004134	1	0.998013437	0	1
1526	GO:0004135	1	0.998013437	0	1
1527	GO:0004136	1	0.995952769	0	2
1528	GO:0004137	1	0.996005151	0	2
1529	GO:0004138	1	0.995952769	0	2
1530	GO:0004139	1	0.997973945	0	1
1531	GO:0004140	1	0.99596674	0	2
1532	GO:0004142	1	0.993976848	0	3
1533	GO:0004143	1	0.980247754	0	10
1534	GO:0004144	1	0.993970415	0	3
1535	GO:0004145	1	0.993946641	0	3
1536	GO:0004146	1	0.996001221	0	2
1537	GO:0004148	1	0.998002843	0	1
1538	GO:0004149	1	0.99799209	0	1
1539	GO:0004151	1	0.998013437	0	1
1540	GO:0004152	1	0.997979743	0	1
1541	GO:0004155	1	0.997972599	0	1
1542	GO:0004157	1	0.995996033	0	2
1543	GO:0004161	1	0.995965062	0	2
1544	GO:0004163	1	0.997975216	0	1
1545	GO:0004164	1	0.997975078	0	1
1546	GO:0004165	1	0.993999571	0	3

1547	GO:0004167	1	0.993900838	0	3
1548	GO:0004168	1	0.997981857	0	1
1549	GO:0004169	1	0.984080062	0	8
1550	GO:0004170	1	0.997976468	0	1
1551	GO:0004174	1	0.99598633	0	2
1552	GO:0004175	1	0.838947274	0	87
1553	GO:0004176	1	0.986027566	0	7
1554	GO:0004177	1	0.93585742	0	33
1555	GO:0004180	1	0.9319763	0	35
1556	GO:0004181	1	0.950995884	0	25
1557	GO:0004185	1	0.989969143	0	5
1558	GO:0004190	1	0.958498677	0	21
1559	GO:0004197	1	0.865256296	0	72
1560	GO:0004198	1	0.970268427	0	15
1561	GO:0004222	1	0.831331453	0	92
1562	GO:0004252	1	0.781317985	0	122
1563	GO:0004298	1	0.973838711	0	13
1564	GO:0004300	1	0.979997191	0	10
1565	GO:0004301	1	0.989936403	0	5
1566	GO:0004303	1	0.966045182	0	17
1567	GO:0004304	1	0.997974904	0	1
1568	GO:0004305	1	0.991981611	0	4
1569	GO:0004306	1	0.997976212	0	1
1570	GO:0004307	1	0.99394687	0	3
1571	GO:0004308	1	0.993966343	0	3
1572	GO:0004309	1	0.990026352	0	5
1573	GO:0004310	1	0.997980382	0	1
1574	GO:0004311	1	0.995989766	0	2
1575	GO:0004312	1	0.995995107	0	2
1576	GO:0004313	1	0.998013437	0	1
1577	GO:0004314	1	0.995995107	0	2
1578	GO:0004315	1	0.995983044	0	2
1579	GO:0004316	1	0.998013437	0	1
1580	GO:0004317	1	0.996007903	0	2
1581	GO:0004320	1	0.995991857	0	2
1582	GO:0004321	1	0.9919591	0	4
1583	GO:0004322	1	0.988037278	0	6
1584	GO:0004324	1	0.997976892	0	1
1585	GO:0004325	1	0.998013437	0	1
1586	GO:0004326	1	0.997986485	0	1
1587	GO:0004329	1	0.996008463	0	2
1588	GO:0004331	1	0.990029311	0	5
1589	GO:0004332	1	0.993940721	0	3
1590	GO:0004333	1	0.997975078	0	1
1591	GO:0004334	1	0.997969048	0	1

1592	GO:0004335	1	0.995968237	0	2
1593	GO:0004336	1	0.997992987	0	1
1594	GO:0004337	1	0.995965062	0	2
1595	GO:0004339	1	0.996007903	0	2
1596	GO:0004340	1	0.992034495	0	4
1597	GO:0004341	1	0.99797739	0	1
1598	GO:0004342	1	0.995964628	0	2
1599	GO:0004343	1	0.998006846	0	1
1600	GO:0004345	1	0.996001468	0	2
1601	GO:0004346	1	0.995971026	0	2
1602	GO:0004347	1	0.997980055	0	1
1603	GO:0004348	1	0.995992346	0	2
1604	GO:0004349	1	0.998001306	0	1
1605	GO:0004350	1	0.998001306	0	1
1606	GO:0004351	1	0.997984584	0	1
1607	GO:0004352	1	0.995989342	0	2
1608	GO:0004353	1	0.995989342	0	2
1609	GO:0004354	1	0.995989342	0	2
1610	GO:0004356	1	0.996027652	0	2
1611	GO:0004357	1	0.996004042	0	2
1612	GO:0004359	1	0.993996587	0	3
1613	GO:0004360	1	0.996012088	0	2
1614	GO:0004361	1	0.997973681	0	1
1615	GO:0004362	1	0.997982996	0	1
1616	GO:0004363	1	0.997977044	0	1
1617	GO:0004364	1	0.95039636	0	25
1618	GO:0004365	1	0.997966382	0	1
1619	GO:0004366	1	0.992009974	0	4
1620	GO:0004367	1	0.996003077	0	2
1621	GO:0004368	1	0.996009811	0	2
1622	GO:0004370	1	0.996020987	0	2
1623	GO:0004371	1	0.997990564	0	1
1624	GO:0004372	1	0.995972487	0	2
1625	GO:0004373	1	0.993997058	0	3
1626	GO:0004375	1	0.998005759	0	1
1627	GO:0004376	1	0.993988326	0	3
1628	GO:0004377	1	0.997987172	0	1
1629	GO:0004378	1	0.997992925	0	1
1630	GO:0004379	1	0.9960294	0	2
1631	GO:0004382	1	0.982084832	0	9
1632	GO:0004383	1	0.9860718	0	7
1633	GO:0004385	1	0.982144148	0	9
1635	GO:0004392	1	0.995949002	0	2
1636	GO:0004394	1	0.998009034	0	1
1637	GO:0004395	1	0.997965466	0	1

1638	GO:0004396	1	0.992034495	0	4
1639	GO:0004397	1	0.997996731	0	1
1640	GO:0004398	1	0.99798523	0	1
1641	GO:0004402	1	0.935880316	0	33
1642	GO:0004407	1	0.962588881	0	19
1643	GO:0004408	1	0.997983283	0	1
1644	GO:0004411	1	0.997977399	0	1
1645	GO:0004415	1	0.985977885	0	7
1646	GO:0004416	1	0.993947628	0	3
1647	GO:0004418	1	0.997969289	0	1
1648	GO:0004419	1	0.997969926	0	1
1649	GO:0004420	1	0.998011252	0	1
1650	GO:0004421	1	0.995990578	0	2
1651	GO:0004422	1	0.995949771	0	2
1652	GO:0004423	1	0.998013437	0	1
1653	GO:0004427	1	0.989913499	0	5
1654	GO:0004430	1	0.992049638	0	4
1655	GO:0004435	1	0.955057719	0	23
1656	GO:0004438	1	0.966609909	0	17
1657	GO:0004439	1	0.982171407	0	9
1658	GO:0004441	1	0.997980112	0	1
1659	GO:0004445	1	0.988057976	0	6
1660	GO:0004446	1	0.997992012	0	1
1661	GO:0004447	1	0.996011076	0	2
1662	GO:0004448	1	0.995962075	0	2
1663	GO:0004449	1	0.99394331	0	3
1664	GO:0004450	1	0.995962075	0	2
1665	GO:0004452	1	0.997980805	0	1
1666	GO:0004454	1	0.99798534	0	1
1667	GO:0004457	1	0.997980071	0	1
1668	GO:0004458	1	0.997979224	0	1
1669	GO:0004459	1	0.991921317	0	4
1670	GO:0004461	1	0.995993512	0	2
1671	GO:0004462	1	0.997979348	0	1
1672	GO:0004463	1	0.997979825	0	1
1673	GO:0004464	1	0.989829158	0	5
1674	GO:0004465	1	0.993978781	0	3
1675	GO:0004466	1	0.991983753	0	4
1676	GO:0004467	1	0.976202889	0	12
1677	GO:0004468	1	0.992039587	0	4
1678	GO:0004470	1	0.991983081	0	4
1679	GO:0004471	1	0.994005581	0	3
1680	GO:0004473	1	0.994005581	0	3
1681	GO:0004474	1	0.998013437	0	1
1682	GO:0004475	1	0.997971774	0	1

1683	GO:0004476	1	0.997974765	0	1
1684	GO:0004477	1	0.993980066	0	3
1685	GO:0004478	1	0.995995939	0	2
1686	GO:0004479	1	0.997990495	0	1
1687	GO:0004482	1	0.998013437	0	1
1688	GO:0004483	1	0.995985075	0	2
1689	GO:0004484	1	0.998011042	0	1
1690	GO:0004485	1	0.995995346	0	2
1691	GO:0004486	1	0.99800132	0	1
1692	GO:0004487	1	0.993980066	0	3
1693	GO:0004488	1	0.991995057	0	4
1694	GO:0004489	1	0.998013437	0	1
1695	GO:0004490	1	0.997971521	0	1
1696	GO:0004491	1	0.997981631	0	1
1697	GO:0004493	1	0.997957643	0	1
1698	GO:0004494	1	0.997990564	0	1
1699	GO:0004496	1	0.997979817	0	1
1700	GO:0004497	1	0.849186543	0	81
1701	GO:0004498	1	0.997986485	0	1
1702	GO:0004499	1	0.989980886	0	5
1703	GO:0004500	1	0.997995064	0	1
1704	GO:0004502	1	0.998012931	0	1
1705	GO:0004503	1	0.995976117	0	2
1706	GO:0004504	1	0.998004627	0	1
1707	GO:0004505	1	0.995949074	0	2
1708	GO:0004506	1	0.997994267	0	1
1709	GO:0004508	1	0.997976434	0	1
1710	GO:0004509	1	0.997979067	0	1
1711	GO:0004510	1	0.995964069	0	2
1712	GO:0004512	1	0.997982884	0	1
1713	GO:0004513	1	0.99596192	0	2
1714	GO:0004514	1	0.993984713	0	3
1715	GO:0004515	1	0.992031607	0	4
1716	GO:0004516	1	0.997990564	0	1
1717	GO:0004517	1	0.994020685	0	3
1718	GO:0004518	1	0.766263863	0	132
1719	GO:0004519	1	0.844216449	0	84
1720	GO:0004520	1	0.980061023	0	10
1721	GO:0004521	1	0.935836386	0	33
1722	GO:0004522	1	0.995933104	0	2
1723	GO:0004523	1	0.987913811	0	6
1724	GO:0004525	1	0.993990066	0	3
1725	GO:0004526	1	0.977984058	0	11
1726	GO:0004527	1	0.900696042	0	52
1727	GO:0004528	1	0.988005719	0	6

1728	GO:0004530	1	0.989977333	0	5
1729	GO:0004531	1	0.997978753	0	1
1730	GO:0004532	1	0.992001445	0	4
1731	GO:0004534	1	0.994039626	0	3
1732	GO:0004535	1	0.974294227	0	13
1733	GO:0004536	1	0.985924698	0	7
1735	GO:0004549	1	0.998005669	0	1
1737	GO:0004551	1	0.987988722	0	6
1738	GO:0004553	1	0.941530299	0	30
1739	GO:0004555	1	0.997976297	0	1
1740	GO:0004556	1	0.991933575	0	4
1741	GO:0004557	1	0.995976961	0	2
1742	GO:0004558	1	0.992042059	0	4
1743	GO:0004559	1	0.986113623	0	7
1744	GO:0004560	1	0.995964717	0	2
1745	GO:0004561	1	0.99799099	0	1
1746	GO:0004563	1	0.991979824	0	4
1747	GO:0004565	1	0.991987806	0	4
1748	GO:0004566	1	0.993982859	0	3
1749	GO:0004567	1	0.997999194	0	1
1750	GO:0004568	1	0.993974123	0	3
1751	GO:0004569	1	0.998011533	0	1
1752	GO:0004571	1	0.986095727	0	7
1753	GO:0004572	1	0.99603042	0	2
1754	GO:0004573	1	0.997990208	0	1
1755	GO:0004576	1	0.993953576	0	3
1756	GO:0004577	1	0.997995109	0	1
1757	GO:0004578	1	0.998006968	0	1
1758	GO:0004579	1	0.989950279	0	5
1759	GO:0004581	1	0.997963625	0	1
1760	GO:0004582	1	0.997961288	0	1
1761	GO:0004586	1	0.995993546	0	2
1762	GO:0004587	1	0.997978312	0	1
1763	GO:0004588	1	0.997989069	0	1
1764	GO:0004590	1	0.997989069	0	1
1765	GO:0004591	1	0.994037969	0	3
1766	GO:0004594	1	0.991976896	0	4
1767	GO:0004595	1	0.997983442	0	1
1768	GO:0004596	1	0.982058433	0	9
1769	GO:0004597	1	0.998003428	0	1
1770	GO:0004598	1	0.998004627	0	1
1771	GO:0004601	1	0.941054406	0	30
1772	GO:0004602	1	0.96204447	0	19
1773	GO:0004603	1	0.997963324	0	1
1774	GO:0004605	1	0.994003605	0	3

1775	GO:0004607	1	0.997967158	0	1
1776	GO:0004608	1	0.997960141	0	1
1777	GO:0004609	1	0.99597418	0	2
1778	GO:0004610	1	0.997978603	0	1
1779	GO:0004611	1	0.997978061	0	1
1780	GO:0004613	1	0.997978061	0	1
1781	GO:0004614	1	0.993974819	0	3
1782	GO:0004615	1	0.995953728	0	2
1783	GO:0004616	1	0.997977668	0	1
1784	GO:0004617	1	0.997978951	0	1
1785	GO:0004618	1	0.997985482	0	1
1786	GO:0004619	1	0.991905896	0	4
1787	GO:0004620	1	0.945312369	0	28
1788	GO:0004621	1	0.997983307	0	1
1789	GO:0004622	1	0.95474004	0	23
1790	GO:0004623	1	0.948930436	0	26
1791	GO:0004629	1	0.982193909	0	9
1792	GO:0004630	1	0.989991928	0	5
1793	GO:0004631	1	0.997966077	0	1
1794	GO:0004632	1	0.997965419	0	1
1795	GO:0004633	1	0.997982179	0	1
1796	GO:0004634	1	0.991945513	0	4
1797	GO:0004637	1	0.998000955	0	1
1798	GO:0004638	1	0.99799951	0	1
1799	GO:0004639	1	0.99799951	0	1
1800	GO:0004641	1	0.998000955	0	1
1801	GO:0004642	1	0.998013363	0	1
1802	GO:0004643	1	0.997980218	0	1
1803	GO:0004644	1	0.998000955	0	1
1804	GO:0004645	1	0.99002652	0	5
1805	GO:0004647	1	0.995963187	0	2
1806	GO:0004648	1	0.997980585	0	1
1807	GO:0004649	1	0.996004225	0	2
1808	GO:0004651	1	0.995987421	0	2
1809	GO:0004652	1	0.984097654	0	8
1810	GO:0004653	1	0.964571898	0	18
1811	GO:0004654	1	0.998011657	0	1
1812	GO:0004655	1	0.99799724	0	1
1813	GO:0004656	1	0.989967065	0	5
1814	GO:0004657	1	0.997982179	0	1
1815	GO:0004658	1	0.995965778	0	2
1816	GO:0004659	1	0.974167917	0	13
1817	GO:0004660	1	0.993965874	0	3
1818	GO:0004661	1	0.995965922	0	2
1819	GO:0004662	1	0.995965922	0	2

1820	GO:0004663	1	0.991954251	0	4
1821	GO:0004666	1	0.996027991	0	2
1822	GO:0004667	1	0.995950925	0	2
1823	GO:0004668	1	0.992020464	0	4
1824	GO:0004671	1	0.998012424	0	1
1825	GO:0004672	1	0.366940106	0	495
1826	GO:0004673	1	0.99795485	0	1
1827	GO:0004674	1	0.450993769	0	394
1828	GO:0004675	1	0.978285987	0	11
1829	GO:0004676	1	0.998013437	0	1
1830	GO:0004677	1	0.99402909	0	3
1831	GO:0004679	1	0.988093565	0	6
1832	GO:0004683	1	0.962573746	0	19
1833	GO:0004686	1	0.998013437	0	1
1834	GO:0004687	1	0.994051874	0	3
1835	GO:0004689	1	0.992025763	0	4
1836	GO:0004691	1	0.988071106	0	6
1837	GO:0004692	1	0.996009057	0	2
1838	GO:0004693	1	0.945130916	0	28
1839	GO:0004694	1	0.992049464	0	4
1840	GO:0004697	1	0.968522547	0	16
1841	GO:0004698	1	0.970451723	0	15
1842	GO:0004699	1	0.996008448	0	2
1843	GO:0004703	1	0.987978387	0	6
1844	GO:0004704	1	0.988027426	0	6
1845	GO:0004705	1	0.993997621	0	3
1846	GO:0004706	1	0.984104621	0	8
1847	GO:0004707	1	0.972262131	0	14
1848	GO:0004708	1	0.964420982	0	18
1849	GO:0004709	1	0.956955104	0	22
1850	GO:0004711	1	0.986103529	0	7
1851	GO:0004712	1	0.945250873	0	28
1852	GO:0004713	1	0.784461729	0	121
1853	GO:0004714	1	0.801886273	0	110
1854	GO:0004715	1	0.913611694	0	45
1855	GO:0004719	1	0.994001287	0	3
1856	GO:0004720	1	0.990036017	0	5
1857	GO:0004721	1	0.75598435	0	139
1858	GO:0004722	1	0.834236798	0	90
1859	GO:0004723	1	0.994022537	0	3
1860	GO:0004725	1	0.819556792	0	99
1861	GO:0004726	1	0.982075245	0	9
1862	GO:0004727	1	0.99594953	0	2
1863	GO:0004729	1	0.997974269	0	1
1864	GO:0004731	1	0.994000321	0	3

1865	GO:0004733	1	0.998001224	0	1
1866	GO:0004735	1	0.991955078	0	4
1867	GO:0004736	1	0.998007762	0	1
1868	GO:0004738	1	0.998000115	0	1
1869	GO:0004739	1	0.995974512	0	2
1870	GO:0004740	1	0.990032502	0	5
1871	GO:0004741	1	0.996019145	0	2
1872	GO:0004742	1	0.998010344	0	1
1873	GO:0004743	1	0.995989458	0	2
1874	GO:0004744	1	0.997988448	0	1
1875	GO:0004745	1	0.966153911	0	17
1876	GO:0004747	1	0.997964177	0	1
1877	GO:0004748	1	0.994021545	0	3
1878	GO:0004749	1	0.991953592	0	4
1879	GO:0004750	1	0.997983458	0	1
1880	GO:0004751	1	0.997975853	0	1
1881	GO:0004753	1	0.998013437	0	1
1882	GO:0004754	1	0.998013437	0	1
1883	GO:0004756	1	0.995975033	0	2
1884	GO:0004757	1	0.997969067	0	1
1885	GO:0004758	1	0.990016376	0	5
1886	GO:0004760	1	0.997971738	0	1
1887	GO:0004766	1	0.997965658	0	1
1888	GO:0004767	1	0.987997526	0	6
1890	GO:0004769	1	0.995932726	0	2
1891	GO:0004771	1	0.986044307	0	7
1892	GO:0004772	1	0.998000348	0	1
1893	GO:0004773	1	0.998013437	0	1
1894	GO:0004775	1	0.993946852	0	3
1895	GO:0004776	1	0.993946852	0	3
1896	GO:0004777	1	0.998013139	0	1
1897	GO:0004778	1	0.995939606	0	2
1898	GO:0004781	1	0.995997377	0	2
1899	GO:0004782	1	0.995997587	0	2
1900	GO:0004784	1	0.989878281	0	5
1901	GO:0004788	1	0.997984426	0	1
1902	GO:0004790	1	0.997987515	0	1
1903	GO:0004791	1	0.987997484	0	6
1904	GO:0004792	1	0.993930079	0	3
1905	GO:0004794	1	0.995943824	0	2
1906	GO:0004796	1	0.995972982	0	2
1907	GO:0004797	1	0.995988551	0	2
1909	GO:0004799	1	0.997971413	0	1
1910	GO:0004800	1	0.995990655	0	2
1911	GO:0004801	1	0.997965064	0	1

1912	GO:0004802	1	0.997980414	0	1
1913	GO:0004803	1	0.994011168	0	3
1914	GO:0004806	1	0.952832886	0	24
1915	GO:0004807	1	0.997969123	0	1
1916	GO:0004809	1	0.993962046	0	3
1917	GO:0004810	1	0.997983092	0	1
1918	GO:0004812	1	0.920737521	0	41
1919	GO:0004813	1	0.99199401	0	4
1920	GO:0004814	1	0.993961102	0	3
1921	GO:0004815	1	0.995994231	0	2
1922	GO:0004816	1	0.995977641	0	2
1923	GO:0004817	1	0.995970989	0	2
1924	GO:0004818	1	0.996001231	0	2
1925	GO:0004819	1	0.995985075	0	2
1926	GO:0004820	1	0.997990564	0	1
1927	GO:0004821	1	0.995981097	0	2
1928	GO:0004822	1	0.995996976	0	2
1929	GO:0004823	1	0.996003907	0	2
1930	GO:0004824	1	0.997990564	0	1
1931	GO:0004825	1	0.995989229	0	2
1932	GO:0004826	1	0.991947649	0	4
1933	GO:0004827	1	0.995978664	0	2
1934	GO:0004828	1	0.995974348	0	2
1935	GO:0004829	1	0.993977787	0	3
1936	GO:0004830	1	0.995986095	0	2
1937	GO:0004831	1	0.99597574	0	2
1938	GO:0004832	1	0.995997881	0	2
1939	GO:0004833	1	0.993940123	0	3
1940	GO:0004835	1	0.998013147	0	1
1941	GO:0004838	1	0.997990727	0	1
1942	GO:0004839	1	0.994014001	0	3
1945	GO:0004844	1	0.991942293	0	4
1946	GO:0004849	1	0.99193781	0	4
1947	GO:0004850	1	0.995964646	0	2
1948	GO:0004852	1	0.997967215	0	1
1949	GO:0004853	1	0.997967779	0	1
1950	GO:0004854	1	0.998013436	0	1
1951	GO:0004855	1	0.998013436	0	1
1952	GO:0004856	1	0.998004233	0	1
1953	GO:0004857	1	0.92804134	0	37
1954	GO:0004859	1	0.985889402	0	7
1955	GO:0004860	1	0.922567435	0	40
1956	GO:0004861	1	0.976057156	0	12
1957	GO:0004862	1	0.984009835	0	8
1958	GO:0004864	1	0.922497677	0	40

1959	GO:0004865	1	0.981905273	0	9
1960	GO:0004866	1	0.929923875	0	36
1962	GO:0004869	1	0.952593377	0	24
1963	GO:0004873	1	0.995942418	0	2
1964	GO:0004875	1	0.987964529	0	6
1965	GO:0004876	1	0.997977802	0	1
1966	GO:0004877	1	0.998013437	0	1
1967	GO:0004878	1	0.995978601	0	2
1968	GO:0004879	1	0.904586745	0	50
1969	GO:0004883	1	0.998013437	0	1
1970	GO:0004888	1	0.678670019	0	192
1971	GO:0004890	1	0.974231094	0	13
1972	GO:0004896	1	0.90419091	0	50
1973	GO:0004897	1	0.994031195	0	3
1974	GO:0004900	1	0.997976092	0	1
1975	GO:0004903	1	0.998010412	0	1
1976	GO:0004904	1	0.996008621	0	2
1977	GO:0004905	1	0.995985648	0	2
1978	GO:0004906	1	0.995966819	0	2
1979	GO:0004908	1	0.988009194	0	6
1980	GO:0004909	1	0.995994721	0	2
1981	GO:0004910	1	0.997968602	0	1
1982	GO:0004911	1	0.993988051	0	3
1983	GO:0004912	1	0.995990225	0	2
1984	GO:0004913	1	0.99595942	0	2
1985	GO:0004914	1	0.995995432	0	2
1986	GO:0004915	1	0.996010011	0	2
1987	GO:0004917	1	0.995949858	0	2
1988	GO:0004918	1	0.997990719	0	1
1989	GO:0004919	1	0.997981211	0	1
1990	GO:0004920	1	0.995986025	0	2
1991	GO:0004921	1	0.993991629	0	3
1992	GO:0004923	1	0.988048751	0	6
1993	GO:0004924	1	0.988048751	0	6
1994	GO:0004925	1	0.998013437	0	1
1995	GO:0004930	1	0.464421221	0	376
1996	GO:0004931	1	0.989952917	0	5
1997	GO:0004935	1	0.986000723	0	7
1998	GO:0004937	1	0.995968263	0	2
1999	GO:0004938	1	0.993994068	0	3
2000	GO:0004939	1	0.997992152	0	1
2001	GO:0004940	1	0.997992152	0	1
2002	GO:0004941	1	0.997979216	0	1
2003	GO:0004945	1	0.995930701	0	2
2004	GO:0004946	1	0.997967083	0	1

2005	GO:0004947	1	0.995979032	0	2
2006	GO:0004948	1	0.996020014	0	2
2007	GO:0004949	1	0.994005619	0	3
2008	GO:0004950	1	0.972099496	0	14
2009	GO:0004952	1	0.993952854	0	3
2010	GO:0004955	1	0.991947464	0	4
2011	GO:0004956	1	0.99799342	0	1
2012	GO:0004957	1	0.989963849	0	5
2013	GO:0004958	1	0.998013356	0	1
2014	GO:0004960	1	0.997982988	0	1
2015	GO:0004961	1	0.997982988	0	1
2016	GO:0004962	1	0.996019502	0	2
2017	GO:0004965	1	0.994044673	0	3
2018	GO:0004966	1	0.995961004	0	2
2019	GO:0004967	1	0.995956535	0	2
2020	GO:0004968	1	0.996026925	0	2
2021	GO:0004969	1	0.992020686	0	4
2022	GO:0004970	1	0.978303602	0	11
2023	GO:0004971	1	0.998013436	0	1
2024	GO:0004972	1	0.986149219	0	7
2025	GO:0004974	1	0.99395366	0	3
2026	GO:0004977	1	0.99799581	0	1
2027	GO:0004979	1	0.997976331	0	1
2028	GO:0004980	1	0.99799581	0	1
2029	GO:0004982	1	0.995948268	0	2
2030	GO:0004983	1	0.98402359	0	8
2031	GO:0004984	1	0.805118962	0	106
2032	GO:0004985	1	0.983964747	0	8
2033	GO:0004990	1	0.998010369	0	1
2034	GO:0004991	1	0.997989952	0	1
2035	GO:0004992	1	0.994003923	0	3
2036	GO:0004993	1	0.970192061	0	15
2037	GO:0004994	1	0.997993743	0	1
2038	GO:0004995	1	0.995987453	0	2
2039	GO:0004996	1	0.995975291	0	2
2040	GO:0004998	1	0.9960099	0	2
2041	GO:0004999	1	0.996002246	0	2
2042	GO:0005000	1	0.98999811	0	5
2043	GO:0005001	1	0.966671512	0	17
2044	GO:0005003	1	0.974404315	0	13
2045	GO:0005004	1	0.9900811	0	5
2046	GO:0005005	1	0.970436935	0	15
2047	GO:0005006	1	0.99598173	0	2
2048	GO:0005007	1	0.990061538	0	5
2049	GO:0005008	1	0.998013437	0	1

2050	GO:0005009	1	0.996030731	0	2
2051	GO:0005010	1	0.994051862	0	3
2052	GO:0005011	1	0.996023816	0	2
2053	GO:0005017	1	0.998013436	0	1
2054	GO:0005018	1	0.998013437	0	1
2055	GO:0005019	1	0.995987294	0	2
2056	GO:0005020	1	0.998013176	0	1
2057	GO:0005021	1	0.986139292	0	7
2058	GO:0005024	1	0.982213644	0	9
2059	GO:0005025	1	0.990071955	0	5
2060	GO:0005026	1	0.99801192	0	1
2061	GO:0005030	1	0.996009765	0	2
2062	GO:0005031	1	0.982011928	0	9
2063	GO:0005034	1	0.997996769	0	1
2064	GO:0005035	1	0.995978738	0	2
2065	GO:0005041	1	0.972406488	0	14
2066	GO:0005042	1	0.990076461	0	5
2067	GO:0005043	1	0.998003737	0	1
2068	GO:0005044	1	0.919182912	0	42
2069	GO:0005046	1	0.993939532	0	3
2070	GO:0005047	1	0.985992836	0	7
2071	GO:0005048	1	0.992002821	0	4
2072	GO:0005049	1	0.978259236	0	11
2073	GO:0005052	1	0.996003873	0	2
2074	GO:0005053	1	0.997969603	0	1
2075	GO:0005055	1	0.995952354	0	2
2076	GO:0005068	1	0.976185144	0	12
2077	GO:0005078	1	0.97826006	0	11
2078	GO:0005080	1	0.89904728	0	53
2079	GO:0005085	1	0.661031475	0	206
2080	GO:0005092	1	0.97423615	0	13
2081	GO:0005093	1	0.995974609	0	2
2082	GO:0005094	1	0.995944831	0	2
2083	GO:0005095	1	0.972267526	0	14
2086	GO:0005104	1	0.96835288	0	16
2087	GO:0005105	1	0.989932258	0	5
2088	GO:0005109	1	0.937651982	0	32
2089	GO:0005111	1	0.991926766	0	4
2090	GO:0005112	1	0.953076504	0	24
2091	GO:0005113	1	0.984026238	0	8
2092	GO:0005114	1	0.986079975	0	7
2093	GO:0005115	1	0.998013437	0	1
2094	GO:0005119	1	0.994033182	0	3
2095	GO:0005121	1	0.997989906	0	1
2096	GO:0005123	1	0.970231704	0	15

2097	GO:0005124	1	0.991933772	0	4
2099	GO:0005126	1	0.987844797	0	6
2100	GO:0005127	1	0.982058774	0	9
2101	GO:0005129	1	0.997955846	0	1
2102	GO:0005130	1	0.997970174	0	1
2103	GO:0005131	1	0.992041408	0	4
2104	GO:0005132	1	0.995930304	0	2
2105	GO:0005133	1	0.997989053	0	1
2106	GO:0005134	1	0.993973057	0	3
2107	GO:0005136	1	0.995974752	0	2
2108	GO:0005137	1	0.997981341	0	1
2109	GO:0005138	1	0.986003493	0	7
2110	GO:0005139	1	0.995970663	0	2
2111	GO:0005141	1	0.997972205	0	1
2112	GO:0005142	1	0.997984331	0	1
2113	GO:0005143	1	0.989989736	0	5
2114	GO:0005146	1	0.995983431	0	2
2115	GO:0005149	1	0.974200604	0	13
2116	GO:0005150	1	0.99598192	0	2
2117	GO:0005151	1	0.995992164	0	2
2118	GO:0005152	1	0.993990708	0	3
2119	GO:0005153	1	0.995985075	0	2
2120	GO:0005154	1	0.937740086	0	32
2121	GO:0005157	1	0.993942497	0	3
2122	GO:0005158	1	0.95895554	0	21
2123	GO:0005159	1	0.972259523	0	14
2124	GO:0005160	1	0.970301806	0	15
2125	GO:0005161	1	0.970475008	0	15
2126	GO:0005163	1	0.989924662	0	5
2128	GO:0005165	1	0.997965083	0	1
2129	GO:0005168	1	0.988108745	0	6
2130	GO:0005169	1	0.998013318	0	1
2131	GO:0005170	1	0.998013318	0	1
2132	GO:0005171	1	0.995962101	0	2
2133	GO:0005172	1	0.987981704	0	6
2134	GO:0005173	1	0.992073054	0	4
2135	GO:0005174	1	0.997983187	0	1
2136	GO:0005175	1	0.997953646	0	1
2137	GO:0005176	1	0.99197299	0	4
2139	GO:0005179	1	0.88191341	0	62
2140	GO:0005183	1	0.995925821	0	2
2141	GO:0005184	1	0.985861814	0	7
2142	GO:0005198	1	0.738696978	0	150
2143	GO:0005200	1	0.837561008	0	88
2144	GO:0005201	1	0.782930053	0	122

2145	GO:0005212	1	0.979916011	0	10
2146	GO:0005215	1	0.95486687	0	23
2149	GO:0005220	1	0.994051875	0	3
2150	GO:0005221	1	0.994010011	0	3
2151	GO:0005222	1	0.984124498	0	8
2152	GO:0005223	1	0.988037707	0	6
2153	GO:0005225	1	0.990084089	0	5
2154	GO:0005227	1	0.976304301	0	12
2155	GO:0005228	1	0.996029659	0	2
2156	GO:0005229	1	0.97237582	0	14
2157	GO:0005230	1	0.930069528	0	36
2158	GO:0005231	1	0.998008787	0	1
2159	GO:0005234	1	0.998012012	0	1
2160	GO:0005237	1	0.986070775	0	7
2161	GO:0005242	1	0.97227784	0	14
2162	GO:0005243	1	0.962388115	0	19
2163	GO:0005244	1	0.785761135	0	120
2164	GO:0005245	1	0.936045975	0	33
2165	GO:0005246	1	0.948992864	0	26
2166	GO:0005247	1	0.978207774	0	11
2167	GO:0005248	1	0.962836703	0	19
2168	GO:0005249	1	0.900972121	0	52
2169	GO:0005250	1	0.992023417	0	4
2170	GO:0005251	1	0.947293514	0	27
2171	GO:0005252	1	0.998007319	0	1
2172	GO:0005253	1	0.992021182	0	4
2173	GO:0005254	1	0.884643872	0	61
2174	GO:0005260	1	0.998013437	0	1
2175	GO:0005261	1	0.910264802	0	47
2177	GO:0005267	1	0.87940265	0	64
2178	GO:0005272	1	0.945529062	0	28
2179	GO:0005275	1	0.997978153	0	1
2180	GO:0005277	1	0.997976127	0	1
2181	GO:0005280	1	0.994019034	0	3
2182	GO:0005283	1	0.996015026	0	2
2183	GO:0005289	1	0.997984536	0	1
2184	GO:0005290	1	0.98999955	0	5
2185	GO:0005292	1	0.997984536	0	1
2186	GO:0005295	1	0.996029942	0	2
2187	GO:0005298	1	0.996027581	0	2
2188	GO:0005302	1	0.997996731	0	1
2189	GO:0005308	1	0.994007548	0	3
2190	GO:0005309	1	0.998002264	0	1
2191	GO:0005310	1	0.995988851	0	2
2192	GO:0005313	1	0.978177749	0	11

2193	GO:0005314	1	0.992019095	0	4
2194	GO:0005315	1	0.990022327	0	5
2195	GO:0005316	1	0.997998701	0	1
2196	GO:0005319	1	0.951179944	0	25
2197	GO:0005324	1	0.972184841	0	14
2198	GO:0005326	1	0.972269882	0	14
2199	GO:0005328	1	0.997999739	0	1
2200	GO:0005330	1	0.994001056	0	3
2201	GO:0005332	1	0.99199354	0	4
2202	GO:0005334	1	0.995979822	0	2
2203	GO:0005335	1	0.996007371	0	2
2204	GO:0005337	1	0.985976791	0	7
2205	GO:0005338	1	0.995960442	0	2
2206	GO:0005342	1	0.997992732	0	1
2207	GO:0005343	1	0.997998802	0	1
2208	GO:0005344	1	0.981836089	0	9
2209	GO:0005345	1	0.993959725	0	3
2210	GO:0005347	1	0.98602094	0	7
2211	GO:0005351	1	0.993996841	0	3
2212	GO:0005353	1	0.993962196	0	3
2213	GO:0005354	1	0.998012716	0	1
2214	GO:0005355	1	0.976125668	0	12
2215	GO:0005362	1	0.997983203	0	1
2216	GO:0005365	1	0.996030731	0	2
2217	GO:0005366	1	0.998013437	0	1
2218	GO:0005367	1	0.996030012	0	2
2219	GO:0005368	1	0.994020353	0	3
2220	GO:0005369	1	0.998013437	0	1
2221	GO:0005372	1	0.992044133	0	4
2222	GO:0005375	1	0.990061829	0	5
2223	GO:0005381	1	0.984060485	0	8
2224	GO:0005384	1	0.986087036	0	7
2225	GO:0005385	1	0.960559079	0	20
2226	GO:0005388	1	0.982222591	0	9
2227	GO:0005391	1	0.986043379	0	7
2228	GO:0005412	1	0.988031283	0	6
2229	GO:0005415	1	0.993959725	0	3
2230	GO:0005427	1	0.996004867	0	2
2231	GO:0005432	1	0.990052153	0	5
2232	GO:0005436	1	0.988027847	0	6
2233	GO:0005452	1	0.980238383	0	10
2234	GO:0005456	1	0.997975457	0	1
2235	GO:0005457	1	0.997997568	0	1
2236	GO:0005459	1	0.991918498	0	4
2237	GO:0005460	1	0.997964003	0	1

2238	GO:0005461	1	0.995989633	0	2
2239	GO:0005462	1	0.992003772	0	4
2240	GO:0005463	1	0.995989633	0	2
2241	GO:0005464	1	0.998013437	0	1
2242	GO:0005471	1	0.991954059	0	4
2243	GO:0005477	1	0.998002224	0	1
2244	GO:0005483	1	0.993998598	0	3
2245	GO:0005484	1	0.929976347	0	36
2246	GO:0005496	1	0.943328043	0	29
2247	GO:0005497	1	0.991919015	0	4
2248	GO:0005499	1	0.99399146	0	3
2249	GO:0005501	1	0.991854484	0	4
2250	GO:0005502	1	0.991999657	0	4
2251	GO:0005503	1	0.991986187	0	4
2252	GO:0005504	1	0.958343861	0	21
2254	GO:0005507	1	0.898384428	0	53
2256	GO:0005513	1	0.972241642	0	14
2259	GO:0005518	1	0.879456098	0	64
2260	GO:0005519	1	0.998007349	0	1
2261	GO:0005520	1	0.958545481	0	21
2263	GO:0005522	1	0.980167556	0	10
2264	GO:0005523	1	0.972138324	0	14
2267	GO:0005527	1	0.997965409	0	1
2268	GO:0005528	1	0.980001099	0	10
2269	GO:0005534	1	0.991957028	0	4
2270	GO:0005536	1	0.980131878	0	10
2271	GO:0005537	1	0.964311114	0	18
2272	GO:0005539	1	0.947269492	0	27
2273	GO:0005540	1	0.970400927	0	15
2274	GO:0005542	1	0.977974618	0	11
2275	GO:0005543	1	0.809466944	0	105
2276	GO:0005544	1	0.904275857	0	50
2277	GO:0005545	1	0.972427639	0	14
2278	GO:0005546	1	0.856527912	0	77
2279	GO:0005547	1	0.928565864	0	37
2280	GO:0005548	1	0.978248693	0	11
2281	GO:0005549	1	0.946329448	0	27
2284	GO:0005577	1	0.990036414	0	5
2285	GO:0005579	1	0.990011573	0	5
2286	GO:0005581	1	0.856765088	0	77
2287	GO:0005582	1	0.9980133	0	1
2288	GO:0005583	1	0.995997814	0	2
2289	GO:0005584	1	0.996030681	0	2
2290	GO:0005585	1	0.998012944	0	1
2291	GO:0005586	1	0.998013412	0	1

2292	GO:0005587	1	0.98813833	0	6
2293	GO:0005588	1	0.992076855	0	4
2294	GO:0005589	1	0.994004633	0	3
2295	GO:0005590	1	0.998013437	0	1
2296	GO:0005591	1	0.997988595	0	1
2297	GO:0005592	1	0.998013437	0	1
2298	GO:0005594	1	0.993991713	0	3
2299	GO:0005595	1	0.998013437	0	1
2300	GO:0005596	1	0.998013437	0	1
2301	GO:0005597	1	0.998013431	0	1
2302	GO:0005600	1	0.997995293	0	1
2303	GO:0005604	1	0.831595149	0	92
2304	GO:0005606	1	0.994051875	0	3
2305	GO:0005607	1	0.996030334	0	2
2306	GO:0005608	1	0.996030731	0	2
2307	GO:0005610	1	0.994046729	0	3
2308	GO:0005614	1	0.978244902	0	11
2310	GO:0005622	1	0.982043112	0	9
2314	GO:0005638	1	0.991948385	0	4
2315	GO:0005639	1	0.97423234	0	13
2317	GO:0005641	1	0.982108392	0	9
2318	GO:0005642	1	0.988067353	0	6
2320	GO:0005652	1	0.982059535	0	9
2322	GO:0005655	1	0.989919868	0	5
2323	GO:0005656	1	0.995965435	0	2
2324	GO:0005657	1	0.945272943	0	28
2325	GO:0005658	1	0.98998804	0	5
2326	GO:0005662	1	0.974119284	0	13
2327	GO:0005663	1	0.989952687	0	5
2328	GO:0005664	1	0.982062939	0	9
2329	GO:0005665	1	0.971985636	0	14
2330	GO:0005666	1	0.964246134	0	18
2331	GO:0005667	1	0.681665312	0	190
2332	GO:0005668	1	0.995953157	0	2
2333	GO:0005669	1	0.935575756	0	33
2334	GO:0005671	1	0.968353972	0	16
2335	GO:0005672	1	0.989929562	0	5
2336	GO:0005673	1	0.9959694	0	2
2337	GO:0005674	1	0.995960552	0	2
2338	GO:0005675	1	0.97605891	0	12
2339	GO:0005677	1	0.982095391	0	9
2340	GO:0005680	1	0.95462332	0	23
2341	GO:0005681	1	0.717434473	0	164
2342	GO:0005682	1	0.966202321	0	17
2343	GO:0005683	1	0.985837538	0	7

2344	GO:0005684	1	0.978027156	0	11
2345	GO:0005685	1	0.962215186	0	19
2346	GO:0005686	1	0.958339323	0	21
2347	GO:0005687	1	0.979861162	0	10
2348	GO:0005688	1	0.983858839	0	8
2349	GO:0005689	1	0.946700255	0	27
2350	GO:0005690	1	0.995976272	0	2
2351	GO:0005691	1	0.998010534	0	1
2353	GO:0005697	1	0.958582822	0	21
2354	GO:0005712	1	0.996003487	0	2
2355	GO:0005713	1	0.997997671	0	1
2356	GO:0005715	1	0.997986141	0	1
2357	GO:0005721	1	0.954944253	0	23
2358	GO:0005726	1	0.990082086	0	5
2360	GO:0005731	1	0.997973786	0	1
2361	GO:0005732	1	0.981854396	0	9
2362	GO:0005736	1	0.974060731	0	13
2365	GO:0005740	1	0.974023903	0	13
2367	GO:0005742	1	0.981886862	0	9
2369	GO:0005744	1	0.977938926	0	11
2370	GO:0005745	1	0.995987659	0	2
2371	GO:0005746	1	0.973984304	0	13
2372	GO:0005747	1	0.903134547	0	50
2373	GO:0005749	1	0.99192948	0	4
2374	GO:0005750	1	0.973970343	0	13
2375	GO:0005751	1	0.979799128	0	10
2376	GO:0005753	1	0.968309862	0	16
2377	GO:0005754	1	0.997990564	0	1
2378	GO:0005757	1	0.98794081	0	6
2379	GO:0005758	1	0.848705074	0	81
2380	GO:0005759	1	0.471146975	0	370
2381	GO:0005760	1	0.994005109	0	3
2382	GO:0005761	1	0.946459078	0	27
2383	GO:0005762	1	0.890403587	0	57
2384	GO:0005763	1	0.944556964	0	28
2387	GO:0005768	1	0.245669101	0	688
2389	GO:0005770	1	0.751288092	0	142
2390	GO:0005771	1	0.926215379	0	38
2391	GO:0005773	1	0.950814513	0	25
2392	GO:0005774	1	0.952900908	0	24
2393	GO:0005776	1	0.867021231	0	71
2394	GO:0005777	1	0.784860453	0	120
2395	GO:0005778	1	0.877328521	0	65
2396	GO:0005779	1	0.96823336	0	16
2397	GO:0005782	1	0.909433563	0	47

2399	GO:0005784	1	0.989915422	0	5
2400	GO:0005785	1	0.991939682	0	4
2401	GO:0005786	1	0.98792741	0	6
2402	GO:0005787	1	0.989926833	0	5
2403	GO:0005788	1	0.57478781	0	274
2406	GO:0005791	1	0.88956311	0	58
2407	GO:0005793	1	0.854790287	0	78
2409	GO:0005795	1	0.928370988	0	37
2410	GO:0005796	1	0.863320471	0	73
2411	GO:0005797	1	0.980030468	0	10
2412	GO:0005798	1	0.964443016	0	18
2413	GO:0005801	1	0.886364805	0	60
2414	GO:0005802	1	0.701478171	0	176
2416	GO:0005813	1	0.351288453	0	515
2417	GO:0005814	1	0.755970716	0	139
2418	GO:0005815	1	0.446472852	0	398
2419	GO:0005818	1	0.998013437	0	1
2420	GO:0005819	1	0.642809237	0	219
2421	GO:0005826	1	0.992011888	0	4
2422	GO:0005827	1	0.990035776	0	5
2423	GO:0005828	1	0.986114202	0	7
2425	GO:0005832	1	0.981987429	0	9
2426	GO:0005833	1	0.987815269	0	6
2427	GO:0005834	1	0.941305011	0	30
2428	GO:0005838	1	0.975987739	0	12
2429	GO:0005839	1	0.96200318	0	19
2430	GO:0005840	1	0.66703216	0	198
2431	GO:0005844	1	0.920798344	0	41
2432	GO:0005845	1	0.978195867	0	11
2433	GO:0005846	1	0.993995783	0	3
2434	GO:0005847	1	0.964499426	0	18
2435	GO:0005848	1	0.99798341	0	1
2436	GO:0005849	1	0.988064061	0	6
2437	GO:0005850	1	0.990034428	0	5
2438	GO:0005851	1	0.987958162	0	6
2439	GO:0005852	1	0.966227516	0	17
2440	GO:0005853	1	0.991928295	0	4
2441	GO:0005854	1	0.993949272	0	3
2443	GO:0005858	1	0.970413748	0	15
2444	GO:0005859	1	0.983937499	0	8
2445	GO:0005861	1	0.983833884	0	8
2446	GO:0005862	1	0.991962956	0	4
2447	GO:0005863	1	0.994039112	0	3
2448	GO:0005865	1	0.980110297	0	10
2449	GO:0005868	1	0.960493263	0	20

2450	GO:0005869	1	0.97797745	0	11
2451	GO:0005871	1	0.908398067	0	48
2452	GO:0005873	1	0.994028228	0	3
2453	GO:0005874	1	0.501836849	0	341
2454	GO:0005875	1	0.93040172	0	36
2455	GO:0005876	1	0.919017463	0	42
2456	GO:0005879	1	0.984076218	0	8
2457	GO:0005880	1	0.995977018	0	2
2458	GO:0005881	1	0.889752842	0	58
2459	GO:0005882	1	0.828809969	0	93
2460	GO:0005883	1	0.978235084	0	11
2461	GO:0005884	1	0.848054994	0	82
2462	GO:0005885	1	0.979944696	0	10
2465	GO:0005889	1	0.998002778	0	1
2466	GO:0005890	1	0.978058973	0	11
2467	GO:0005891	1	0.953111507	0	24
2468	GO:0005892	1	0.97418684	0	13
2469	GO:0005893	1	0.996009596	0	2
2470	GO:0005895	1	0.997981341	0	1
2471	GO:0005896	1	0.9939818	0	3
2472	GO:0005898	1	0.998007667	0	1
2473	GO:0005899	1	0.994051875	0	3
2474	GO:0005900	1	0.996004865	0	2
2475	GO:0005901	1	0.875888269	0	66
2476	GO:0005902	1	0.884719957	0	61
2477	GO:0005903	1	0.89544084	0	55
2478	GO:0005905	1	0.876011998	0	66
2479	GO:0005911	1	0.70052036	0	177
2480	GO:0005912	1	0.722116952	0	162
2481	GO:0005914	1	0.997999496	0	1
2482	GO:0005915	1	0.982178318	0	9
2483	GO:0005916	1	0.982189368	0	9
2484	GO:0005918	1	0.998013392	0	1
2485	GO:0005921	1	0.943221727	0	29
2486	GO:0005922	1	0.964327281	0	18
2488	GO:0005925	1	0.434440022	0	411
2489	GO:0005927	1	0.995975348	0	2
2490	GO:0005929	1	0.476239981	0	366
2491	GO:0005930	1	0.814553817	0	102
2492	GO:0005938	1	0.717520249	0	165
2493	GO:0005940	1	0.972224266	0	14
2494	GO:0005942	1	0.960769949	0	20
2495	GO:0005943	1	0.994042931	0	3
2496	GO:0005944	1	0.99404055	0	3
2497	GO:0005945	1	0.993995087	0	3

2498	GO:0005947	1	0.991981425	0	4
2499	GO:0005948	1	0.997983585	0	1
2500	GO:0005952	1	0.98405469	0	8
2501	GO:0005953	1	0.995965922	0	2
2502	GO:0005954	1	0.992057873	0	4
2503	GO:0005955	1	0.990037422	0	5
2504	GO:0005956	1	0.989912058	0	5
2505	GO:0005958	1	0.992008315	0	4
2506	GO:0005960	1	0.995979678	0	2
2507	GO:0005964	1	0.990054666	0	5
2508	GO:0005965	1	0.993965874	0	3
2509	GO:0005967	1	0.990015049	0	5
2510	GO:0005968	1	0.991995704	0	4
2511	GO:0005969	1	0.998013437	0	1
2512	GO:0005971	1	0.996001074	0	2
2513	GO:0005975	1	0.638433309	0	222
2514	GO:0005976	1	0.995977545	0	2
2515	GO:0005977	1	0.930308478	0	36
2516	GO:0005978	1	0.958684101	0	21
2517	GO:0005979	1	0.978180698	0	11
2518	GO:0005980	1	0.970444807	0	15
2519	GO:0005981	1	0.992019904	0	4
2520	GO:0005983	1	0.998013437	0	1
2521	GO:0005984	1	0.997981777	0	1
2522	GO:0005985	1	0.998003076	0	1
2523	GO:0005986	1	0.99594082	0	2
2524	GO:0005989	1	0.996017128	0	2
2525	GO:0005991	1	0.997976297	0	1
2526	GO:0005993	1	0.997976297	0	1
2527	GO:0005996	1	0.995962615	0	2
2528	GO:0005997	1	0.995965115	0	2
2529	GO:0005998	1	0.998004233	0	1
2530	GO:0005999	1	0.995949495	0	2
2531	GO:0006000	1	0.980028143	0	10
2532	GO:0006002	1	0.980089128	0	10
2533	GO:0006003	1	0.990029311	0	5
2534	GO:0006004	1	0.986020439	0	7
2535	GO:0006006	1	0.89706451	0	54
2536	GO:0006007	1	0.994000329	0	3
2537	GO:0006011	1	0.995951817	0	2
2538	GO:0006012	1	0.982018038	0	9
2539	GO:0006013	1	0.984097472	0	8
2540	GO:0006014	1	0.995944058	0	2
2541	GO:0006015	1	0.989960805	0	5
2542	GO:0006020	1	0.986080418	0	7

2543	GO:0006021	1	0.99396501	0	3
2544	GO:0006024	1	0.932159393	0	35
2545	GO:0006027	1	0.952993506	0	24
2546	GO:0006029	1	0.98799883	0	6
2547	GO:0006032	1	0.989947317	0	5
2548	GO:0006041	1	0.995989386	0	2
2549	GO:0006043	1	0.995964628	0	2
2550	GO:0006044	1	0.966376305	0	17
2551	GO:0006045	1	0.99602255	0	2
2552	GO:0006046	1	0.993948438	0	3
2553	GO:0006047	1	0.98602568	0	7
2554	GO:0006048	1	0.978071226	0	11
2555	GO:0006049	1	0.997993157	0	1
2556	GO:0006051	1	0.995944712	0	2
2557	GO:0006054	1	0.992034187	0	4
2558	GO:0006055	1	0.997964968	0	1
2559	GO:0006060	1	0.997987078	0	1
2560	GO:0006061	1	0.997968061	0	1
2561	GO:0006062	1	0.997987078	0	1
2562	GO:0006065	1	0.994003501	0	3
2563	GO:0006066	1	0.987937336	0	6
2564	GO:0006067	1	0.997978287	0	1
2565	GO:0006068	1	0.975994849	0	12
2566	GO:0006069	1	0.976082979	0	12
2567	GO:0006071	1	0.981995734	0	9
2568	GO:0006072	1	0.986093875	0	7
2569	GO:0006081	1	0.979999297	0	10
2570	GO:0006082	1	0.958474727	0	21
2571	GO:0006084	1	0.988063959	0	6
2572	GO:0006085	1	0.988003864	0	6
2573	GO:0006086	1	0.992007253	0	4
2574	GO:0006089	1	0.990020067	0	5
2575	GO:0006090	1	0.958564192	0	21
2576	GO:0006091	1	0.920669171	0	41
2577	GO:0006094	1	0.924172729	0	39
2578	GO:0006096	1	0.937508076	0	32
2579	GO:0006097	1	0.995962075	0	2
2580	GO:0006098	1	0.976044065	0	12
2581	GO:0006099	1	0.935617243	0	33
2582	GO:0006101	1	0.990049923	0	5
2583	GO:0006102	1	0.987939624	0	6
2584	GO:0006103	1	0.964390309	0	18
2585	GO:0006104	1	0.989979545	0	5
2586	GO:0006105	1	0.987976892	0	6
2587	GO:0006106	1	0.997975078	0	1

2588	GO:0006107	1	0.983947851	0	8
2589	GO:0006108	1	0.985967973	0	7
2590	GO:0006109	1	0.998013437	0	1
2591	GO:0006110	1	0.91911587	0	42
2592	GO:0006111	1	0.968365809	0	16
2593	GO:0006112	1	0.978158568	0	11
2594	GO:0006114	1	0.991980114	0	4
2595	GO:0006116	1	0.991966268	0	4
2596	GO:0006117	1	0.997987849	0	1
2597	GO:0006119	1	0.948469985	0	26
2598	GO:0006120	1	0.904967695	0	49
2599	GO:0006121	1	0.991930636	0	4
2600	GO:0006122	1	0.973988508	0	13
2601	GO:0006123	1	0.964073424	0	18
2602	GO:0006124	1	0.99397697	0	3
2603	GO:0006127	1	0.996009811	0	2
2606	GO:0006145	1	0.997996294	0	1
2607	GO:0006147	1	0.998013378	0	1
2608	GO:0006148	1	0.996002625	0	2
2609	GO:0006154	1	0.991985922	0	4
2610	GO:0006157	1	0.997970558	0	1
2612	GO:0006164	1	0.960471612	0	20
2614	GO:0006166	1	0.987937953	0	6
2615	GO:0006167	1	0.991934614	0	4
2616	GO:0006168	1	0.995927396	0	2
2617	GO:0006170	1	0.995952769	0	2
2618	GO:0006171	1	0.982234844	0	9
2619	GO:0006172	1	0.989987967	0	5
2620	GO:0006173	1	0.998002979	0	1
2621	GO:0006174	1	0.997990564	0	1
2622	GO:0006175	1	0.997979513	0	1
2623	GO:0006177	1	0.993958218	0	3
2624	GO:0006178	1	0.997968322	0	1
2625	GO:0006182	1	0.984047341	0	8
2626	GO:0006183	1	0.977867452	0	11
2627	GO:0006185	1	0.997960081	0	1
2628	GO:0006186	1	0.997990564	0	1
2629	GO:0006188	1	0.994012009	0	3
2630	GO:0006189	1	0.988027195	0	6
2631	GO:0006193	1	0.99796226	0	1
2632	GO:0006195	1	0.978092481	0	11
2633	GO:0006196	1	0.992025804	0	4
2634	GO:0006198	1	0.982215647	0	9
2635	GO:0006203	1	0.995980294	0	2
2636	GO:0006204	1	0.993969684	0	3

2637	GO:0006206	1	0.993953602	0	3
2638	GO:0006207	1	0.988059751	0	6
2639	GO:0006208	1	0.99598094	0	2
2640	GO:0006210	1	0.993970499	0	3
2641	GO:0006211	1	0.994051875	0	3
2642	GO:0006212	1	0.99598094	0	2
2643	GO:0006213	1	0.993931649	0	3
2644	GO:0006214	1	0.997996294	0	1
2645	GO:0006218	1	0.995964646	0	2
2646	GO:0006220	1	0.989986423	0	5
2648	GO:0006222	1	0.991968116	0	4
2649	GO:0006225	1	0.997992152	0	1
2650	GO:0006226	1	0.995959573	0	2
2652	GO:0006228	1	0.979880297	0	10
2653	GO:0006231	1	0.995954528	0	2
2656	GO:0006241	1	0.97790412	0	11
2657	GO:0006248	1	0.991955992	0	4
2658	GO:0006249	1	0.987936205	0	6
2659	GO:0006253	1	0.997962564	0	1
2660	GO:0006254	1	0.998013437	0	1
2661	GO:0006256	1	0.99601066	0	2
2662	GO:0006258	1	0.997966837	0	1
2663	GO:0006259	1	0.943260868	0	29
2664	GO:0006260	1	0.728783626	0	157
2665	GO:0006261	1	0.950895884	0	25
2666	GO:0006264	1	0.982060976	0	9
2667	GO:0006265	1	0.98211465	0	9
2668	GO:0006266	1	0.986025541	0	7
2669	GO:0006267	1	0.984074098	0	8
2670	GO:0006268	1	0.96839901	0	16
2671	GO:0006269	1	0.988001921	0	6
2672	GO:0006270	1	0.920728644	0	41
2673	GO:0006271	1	0.974171536	0	13
2674	GO:0006272	1	0.992001186	0	4
2675	GO:0006273	1	0.994021287	0	3
2676	GO:0006275	1	0.954884648	0	23
2677	GO:0006278	1	0.994015837	0	3
2678	GO:0006281	1	0.447988194	0	396
2679	GO:0006282	1	0.980082095	0	10
2680	GO:0006283	1	0.864518429	0	72
2681	GO:0006284	1	0.939390421	0	31
2682	GO:0006285	1	0.989970176	0	5
2683	GO:0006286	1	0.995939237	0	2
2684	GO:0006287	1	0.931761278	0	35
2685	GO:0006288	1	0.993958136	0	3

2686	GO:0006289	1	0.924423027	0	39
2687	GO:0006290	1	0.990017854	0	5
2688	GO:0006293	1	0.958632262	0	21
2689	GO:0006294	1	0.943156982	0	29
2690	GO:0006295	1	0.958632262	0	21
2691	GO:0006296	1	0.928075647	0	37
2692	GO:0006297	1	0.954662858	0	23
2693	GO:0006298	1	0.945234609	0	28
2694	GO:0006301	1	0.978122121	0	11
2695	GO:0006302	1	0.819343582	0	99
2696	GO:0006303	1	0.873911841	0	67
2697	GO:0006304	1	0.997972608	0	1
2698	GO:0006306	1	0.951178361	0	25
2699	GO:0006307	1	0.980018781	0	10
2700	GO:0006308	1	0.985953923	0	7
2701	GO:0006309	1	0.970292836	0	15
2702	GO:0006310	1	0.819014817	0	99
2703	GO:0006311	1	0.995988008	0	2
2704	GO:0006312	1	0.988019968	0	6
2705	GO:0006313	1	0.996002396	0	2
2706	GO:0006323	1	0.993932919	0	3
2708	GO:0006333	1	0.982086545	0	9
2709	GO:0006334	1	0.829186538	0	93
2710	GO:0006335	1	0.937624053	0	32
2711	GO:0006336	1	0.949103628	0	26
2712	GO:0006337	1	0.968388689	0	16
2713	GO:0006338	1	0.821321175	0	98
2714	GO:0006342	1	0.920797889	0	41
2715	GO:0006346	1	0.988014082	0	6
2716	GO:0006348	1	0.982007214	0	9
2717	GO:0006349	1	0.974288451	0	13
2718	GO:0006351	1	0.804405546	0	108
2719	GO:0006352	1	0.922525683	0	40
2720	GO:0006353	1	0.980120359	0	10
2721	GO:0006354	1	0.995957738	0	2
2723	GO:0006356	1	0.993941085	0	3
2725	GO:0006359	1	0.984026443	0	8
2726	GO:0006360	1	0.983999781	0	8
2727	GO:0006361	1	0.935524207	0	33
2728	GO:0006362	1	0.99200793	0	4
2729	GO:0006363	1	0.939284446	0	31
2730	GO:0006364	1	0.652271369	0	210
2731	GO:0006366	1	0.700803844	0	176
2732	GO:0006367	1	0.763253412	0	134
2733	GO:0006368	1	0.875310234	0	66

2734	GO:0006369	1	0.933767769	0	34
2735	GO:0006370	1	0.937375761	0	32
2736	GO:0006376	1	0.956788554	0	22
2737	GO:0006378	1	0.947228711	0	27
2738	GO:0006379	1	0.984000836	0	8
2739	GO:0006382	1	0.99006018	0	5
2740	GO:0006383	1	0.954663982	0	23
2741	GO:0006384	1	0.986047013	0	7
2742	GO:0006386	1	0.997956149	0	1
2743	GO:0006388	1	0.970085736	0	15
2744	GO:0006390	1	0.980063146	0	10
2745	GO:0006391	1	0.991947845	0	4
2746	GO:0006392	1	0.997990564	0	1
2747	GO:0006393	1	0.995985075	0	2
2748	GO:0006396	1	0.822592826	0	97
2750	GO:0006398	1	0.990007152	0	5
2751	GO:0006399	1	0.995961922	0	2
2752	GO:0006400	1	0.933748282	0	34
2754	GO:0006402	1	0.941446245	0	30
2755	GO:0006403	1	0.994009354	0	3
2756	GO:0006404	1	0.998013437	0	1
2757	GO:0006405	1	0.897006367	0	54
2758	GO:0006406	1	0.816037845	0	101
2759	GO:0006407	1	0.995984749	0	2
2760	GO:0006408	1	0.995958443	0	2
2761	GO:0006409	1	0.93404878	0	34
2762	GO:0006412	1	0.536704116	0	305
2763	GO:0006413	1	0.745818397	0	144
2764	GO:0006414	1	0.943141185	0	29
2765	GO:0006415	1	0.982059585	0	9
2766	GO:0006417	1	0.746799693	0	145
2767	GO:0006418	1	0.916929376	0	43
2768	GO:0006419	1	0.99199401	0	4
2769	GO:0006420	1	0.993961102	0	3
2770	GO:0006421	1	0.995977641	0	2
2771	GO:0006422	1	0.997990564	0	1
2772	GO:0006423	1	0.995970989	0	2
2773	GO:0006424	1	0.996001231	0	2
2774	GO:0006425	1	0.995985075	0	2
2775	GO:0006426	1	0.997990564	0	1
2776	GO:0006427	1	0.995981097	0	2
2777	GO:0006428	1	0.995996976	0	2
2778	GO:0006429	1	0.996003907	0	2
2779	GO:0006430	1	0.997990564	0	1
2780	GO:0006431	1	0.995989229	0	2

2781	GO:0006432	1	0.993946791	0	3
2782	GO:0006433	1	0.995978664	0	2
2783	GO:0006434	1	0.995974348	0	2
2784	GO:0006435	1	0.993977787	0	3
2785	GO:0006436	1	0.995986095	0	2
2786	GO:0006437	1	0.99597574	0	2
2787	GO:0006438	1	0.995997881	0	2
2788	GO:0006446	1	0.94137107	0	30
2789	GO:0006448	1	0.99600531	0	2
2790	GO:0006449	1	0.986071172	0	7
2791	GO:0006450	1	0.987887741	0	6
2792	GO:0006457	1	0.726175329	0	158
2793	GO:0006458	1	0.99392091	0	3
2794	GO:0006464	1	0.812473339	0	103
2795	GO:0006465	1	0.978049721	0	11
2796	GO:0006468	1	0.307504603	0	581
2797	GO:0006469	1	0.842510446	0	85
2798	GO:0006470	1	0.698709839	0	178
2799	GO:0006471	1	0.956724239	0	22
2800	GO:0006473	1	0.97028808	0	15
2801	GO:0006474	1	0.984008876	0	8
2802	GO:0006475	1	0.991980874	0	4
2803	GO:0006476	1	0.970298145	0	15
2804	GO:0006477	1	0.990021335	0	5
2805	GO:0006478	1	0.995961175	0	2
2806	GO:0006479	1	0.939477467	0	31
2807	GO:0006480	1	0.997990564	0	1
2808	GO:0006481	1	0.995982348	0	2
2809	GO:0006482	1	0.980188846	0	10
2810	GO:0006486	1	0.719897449	0	163
2811	GO:0006487	1	0.913339091	0	45
2812	GO:0006488	1	0.970274059	0	15
2813	GO:0006489	1	0.986001686	0	7
2814	GO:0006490	1	0.991977533	0	4
2815	GO:0006491	1	0.968482255	0	16
2816	GO:0006493	1	0.930325229	0	36
2817	GO:0006497	1	0.976160856	0	12
2818	GO:0006499	1	0.992050768	0	4
2819	GO:0006501	1	0.992028489	0	4
2820	GO:0006505	1	0.993997486	0	3
2821	GO:0006506	1	0.939339038	0	31
2822	GO:0006507	1	0.997983307	0	1
2823	GO:0006508	1	0.335167857	0	537
2824	GO:0006509	1	0.962587917	0	19
2826	GO:0006513	1	0.939645173	0	31

2827	GO:0006515	1	0.974282784	0	13
2828	GO:0006516	1	0.974136954	0	13
2829	GO:0006517	1	0.984062231	0	8
2830	GO:0006518	1	0.976210989	0	12
2831	GO:0006520	1	0.939321655	0	31
2832	GO:0006521	1	0.90340201	0	50
2833	GO:0006525	1	0.989957594	0	5
2834	GO:0006526	1	0.993945948	0	3
2835	GO:0006527	1	0.988011312	0	6
2836	GO:0006528	1	0.997965639	0	1
2837	GO:0006529	1	0.995974059	0	2
2838	GO:0006531	1	0.987954953	0	6
2839	GO:0006532	1	0.995970589	0	2
2840	GO:0006533	1	0.99193034	0	4
2841	GO:0006534	1	0.993986205	0	3
2842	GO:0006535	1	0.99598273	0	2
2843	GO:0006536	1	0.970259052	0	15
2844	GO:0006537	1	0.990031182	0	5
2845	GO:0006538	1	0.992003947	0	4
2846	GO:0006539	1	0.99797696	0	1
2847	GO:0006540	1	0.997984584	0	1
2848	GO:0006541	1	0.960637537	0	20
2849	GO:0006542	1	0.996027652	0	2
2850	GO:0006543	1	0.991988167	0	4
2851	GO:0006544	1	0.988013774	0	6
2852	GO:0006545	1	0.991989448	0	4
2853	GO:0006546	1	0.991982026	0	4
2854	GO:0006547	1	0.989979135	0	5
2855	GO:0006548	1	0.98400619	0	8
2856	GO:0006549	1	0.997972053	0	1
2857	GO:0006550	1	0.989946194	0	5
2858	GO:0006551	1	0.997972053	0	1
2859	GO:0006552	1	0.987984618	0	6
2860	GO:0006554	1	0.976161553	0	12
2861	GO:0006555	1	0.987951823	0	6
2862	GO:0006556	1	0.991986237	0	4
2863	GO:0006557	1	0.997998357	0	1
2864	GO:0006558	1	0.98992771	0	5
2865	GO:0006559	1	0.98791603	0	6
2866	GO:0006560	1	0.995984286	0	2
2867	GO:0006561	1	0.991965756	0	4
2868	GO:0006562	1	0.995984286	0	2
2869	GO:0006563	1	0.987961999	0	6
2870	GO:0006564	1	0.98595126	0	7
2871	GO:0006565	1	0.991952369	0	4

2872	GO:0006566	1	0.997978951	0	1
2873	GO:0006567	1	0.993923261	0	3
2874	GO:0006568	1	0.993972461	0	3
2875	GO:0006569	1	0.979980811	0	10
2876	GO:0006570	1	0.991938504	0	4
2877	GO:0006572	1	0.989923463	0	5
2878	GO:0006573	1	0.995957684	0	2
2879	GO:0006574	1	0.989939837	0	5
2880	GO:0006575	1	0.997990564	0	1
2881	GO:0006576	1	0.993999825	0	3
2882	GO:0006577	1	0.997986657	0	1
2883	GO:0006579	1	0.995986395	0	2
2884	GO:0006580	1	0.995986981	0	2
2885	GO:0006581	1	0.997987865	0	1
2886	GO:0006582	1	0.99600238	0	2
2887	GO:0006583	1	0.995967901	0	2
2888	GO:0006584	1	0.980024993	0	10
2889	GO:0006589	1	0.997995064	0	1
2890	GO:0006590	1	0.972367154	0	14
2891	GO:0006591	1	0.995964192	0	2
2892	GO:0006592	1	0.998001306	0	1
2893	GO:0006595	1	0.985903989	0	7
2894	GO:0006596	1	0.975993494	0	12
2895	GO:0006597	1	0.995975667	0	2
2896	GO:0006598	1	0.993927897	0	3
2897	GO:0006600	1	0.983921333	0	8
2898	GO:0006601	1	0.995952406	0	2
2899	GO:0006605	1	0.922653839	0	40
2900	GO:0006606	1	0.844853855	0	84
2901	GO:0006607	1	0.960802786	0	20
2902	GO:0006610	1	0.992008957	0	4
2903	GO:0006611	1	0.945325234	0	28
2904	GO:0006612	1	0.907873922	0	48
2905	GO:0006613	1	0.980038496	0	10
2906	GO:0006614	1	0.831858602	0	90
2907	GO:0006616	1	0.983987077	0	8
2908	GO:0006617	1	0.995944583	0	2
2909	GO:0006620	1	0.984070236	0	8
2910	GO:0006621	1	0.984019913	0	8
2911	GO:0006622	1	0.964583567	0	18
2912	GO:0006623	1	0.968421931	0	16
2913	GO:0006624	1	0.995940218	0	2
2914	GO:0006625	1	0.982081922	0	9
2915	GO:0006626	1	0.939065694	0	31
2916	GO:0006627	1	0.989938391	0	5

2920	GO:0006635	1	0.905936145	0	49
2922	GO:0006637	1	0.948758448	0	26
2923	GO:0006638	1	0.997996934	0	1
2924	GO:0006639	1	0.998013408	0	1
2925	GO:0006640	1	0.989951958	0	5
2926	GO:0006641	1	0.948999687	0	26
2927	GO:0006642	1	0.992040629	0	4
2928	GO:0006643	1	0.995977983	0	2
2929	GO:0006644	1	0.88432732	0	61
2930	GO:0006646	1	0.972271545	0	14
2931	GO:0006649	1	0.998013437	0	1
2932	GO:0006650	1	0.982059129	0	9
2933	GO:0006651	1	0.9859859	0	7
2934	GO:0006654	1	0.913691094	0	45
2935	GO:0006655	1	0.990014697	0	5
2936	GO:0006656	1	0.939386953	0	31
2937	GO:0006657	1	0.985994596	0	7
2938	GO:0006658	1	0.983957611	0	8
2939	GO:0006659	1	0.991980841	0	4
2940	GO:0006660	1	0.993947925	0	3
2941	GO:0006661	1	0.858836362	0	76
2942	GO:0006662	1	0.995921147	0	2
2943	GO:0006663	1	0.991988643	0	4
2944	GO:0006664	1	0.993960417	0	3
2945	GO:0006665	1	0.895282648	0	55
2946	GO:0006666	1	0.998013197	0	1
2947	GO:0006667	1	0.997968061	0	1
2948	GO:0006668	1	0.997998933	0	1
2949	GO:0006669	1	0.99799382	0	1
2950	GO:0006670	1	0.982007121	0	9
2951	GO:0006672	1	0.952888785	0	24
2952	GO:0006677	1	0.995970937	0	2
2953	GO:0006678	1	0.99798512	0	1
2954	GO:0006679	1	0.995961751	0	2
2955	GO:0006680	1	0.995992346	0	2
2956	GO:0006682	1	0.991961256	0	4
2957	GO:0006683	1	0.997992987	0	1
2958	GO:0006684	1	0.992007298	0	4
2959	GO:0006685	1	0.987997526	0	6
2960	GO:0006686	1	0.986038065	0	7
2961	GO:0006687	1	0.926366211	0	38
2962	GO:0006688	1	0.98405226	0	8
2963	GO:0006689	1	0.987979293	0	6
2964	GO:0006690	1	0.980063561	0	10
2965	GO:0006691	1	0.966261901	0	17

2966	GO:0006692	1	0.998012967	0	1
2967	GO:0006693	1	0.943119727	0	29
2970	GO:0006699	1	0.945160624	0	28
2971	GO:0006700	1	0.979988919	0	10
2972	GO:0006701	1	0.993922975	0	3
2973	GO:0006702	1	0.983981035	0	8
2974	GO:0006703	1	0.977972765	0	11
2975	GO:0006704	1	0.987908091	0	6
2976	GO:0006705	1	0.997979067	0	1
2977	GO:0006706	1	0.989973412	0	5
2978	GO:0006707	1	0.97998454	0	10
2979	GO:0006710	1	0.989951525	0	5
2980	GO:0006711	1	0.997974904	0	1
2981	GO:0006713	1	0.997975026	0	1
2982	GO:0006714	1	0.998005077	0	1
2983	GO:0006720	1	0.995968282	0	2
2984	GO:0006725	1	0.992001615	0	4
2985	GO:0006726	1	0.99396176	0	3
2986	GO:0006729	1	0.985914064	0	7
2987	GO:0006730	1	0.935675724	0	33
2988	GO:0006734	1	0.978062506	0	11
2989	GO:0006738	1	0.997985277	0	1
2990	GO:0006739	1	0.980036163	0	10
2991	GO:0006740	1	0.993991876	0	3
2992	GO:0006741	1	0.993974439	0	3
2993	GO:0006742	1	0.993955956	0	3
2994	GO:0006743	1	0.996013199	0	2
2995	GO:0006744	1	0.970110414	0	15
2996	GO:0006747	1	0.997978145	0	1
2997	GO:0006749	1	0.909146588	0	47
2998	GO:0006750	1	0.968261032	0	16
2999	GO:0006751	1	0.983945063	0	8
3000	GO:0006753	1	0.995925977	0	2
3001	GO:0006754	1	0.943214135	0	29
3002	GO:0006756	1	0.997990564	0	1
3003	GO:0006757	1	0.997990564	0	1
3004	GO:0006760	1	0.993978488	0	3
3005	GO:0006766	1	0.986015578	0	7
3006	GO:0006768	1	0.9820595	0	9
3007	GO:0006771	1	0.986007179	0	7
3008	GO:0006772	1	0.993962035	0	3
3009	GO:0006776	1	0.98799328	0	6
3010	GO:0006777	1	0.988007155	0	6
3011	GO:0006778	1	0.990011225	0	5
3012	GO:0006779	1	0.980046326	0	10

3013	GO:0006780	1	0.997967215	0	1
3014	GO:0006781	1	0.997981373	0	1
3015	GO:0006782	1	0.983974108	0	8
3016	GO:0006783	1	0.9508643	0	25
3017	GO:0006784	1	0.996011705	0	2
3018	GO:0006788	1	0.995949002	0	2
3019	GO:0006789	1	0.995972995	0	2
3020	GO:0006790	1	0.974216446	0	13
3021	GO:0006796	1	0.96824353	0	16
3022	GO:0006797	1	0.997992012	0	1
3023	GO:0006798	1	0.990026352	0	5
3024	GO:0006801	1	0.958492594	0	21
3025	GO:0006805	1	0.83864735	0	87
3026	GO:0006807	1	0.962605141	0	19
3027	GO:0006808	1	0.995966431	0	2
3028	GO:0006809	1	0.9683153	0	16
3030	GO:0006812	1	0.869010202	0	70
3031	GO:0006813	1	0.791827676	0	116
3032	GO:0006814	1	0.792004749	0	116
3034	GO:0006817	1	0.982103369	0	9
3035	GO:0006820	1	0.956857039	0	22
3036	GO:0006821	1	0.86699035	0	71
3037	GO:0006824	1	0.989976742	0	5
3038	GO:0006825	1	0.976187777	0	12
3039	GO:0006826	1	0.966430859	0	17
3040	GO:0006828	1	0.986110832	0	7
3041	GO:0006829	1	0.952846108	0	24
3042	GO:0006833	1	0.972138726	0	14
3043	GO:0006835	1	0.995988851	0	2
3044	GO:0006836	1	0.888372901	0	59
3045	GO:0006837	1	0.996028669	0	2
3046	GO:0006839	1	0.962417739	0	19
3047	GO:0006842	1	0.997997664	0	1
3048	GO:0006843	1	0.997971738	0	1
3049	GO:0006844	1	0.993969822	0	3
3050	GO:0006848	1	0.997984457	0	1
3051	GO:0006850	1	0.995985075	0	2
3052	GO:0006851	1	0.952810522	0	24
3053	GO:0006853	1	0.980173013	0	10
3054	GO:0006855	1	0.986140056	0	7
3055	GO:0006857	1	0.992028913	0	4
3056	GO:0006858	1	0.993958116	0	3
3057	GO:0006862	1	0.99598926	0	2
3058	GO:0006864	1	0.995985008	0	2
3059	GO:0006865	1	0.89360242	0	56

3060	GO:0006867	1	0.993967518	0	3
3061	GO:0006868	1	0.984053486	0	8
3063	GO:0006873	1	0.990008561	0	5
3065	GO:0006875	1	0.989937595	0	5
3066	GO:0006876	1	0.996007952	0	2
3067	GO:0006878	1	0.968264143	0	16
3068	GO:0006879	1	0.891490821	0	57
3069	GO:0006880	1	0.993913141	0	3
3070	GO:0006882	1	0.950677042	0	25
3071	GO:0006883	1	0.964548967	0	18
3072	GO:0006884	1	0.958764769	0	21
3073	GO:0006885	1	0.962654331	0	19
3074	GO:0006886	1	0.535254707	0	309
3075	GO:0006887	1	0.799954218	0	111
3076	GO:0006888	1	0.698128444	0	178
3077	GO:0006890	1	0.851253133	0	80
3078	GO:0006891	1	0.94333517	0	29
3079	GO:0006892	1	0.891556046	0	57
3080	GO:0006893	1	0.951163189	0	25
3081	GO:0006895	1	0.970462952	0	15
3082	GO:0006896	1	0.982097448	0	9
3084	GO:0006898	1	0.827614529	0	94
3085	GO:0006900	1	0.972150575	0	14
3086	GO:0006903	1	0.982100139	0	9
3087	GO:0006904	1	0.939536183	0	31
3088	GO:0006906	1	0.932080589	0	35
3089	GO:0006907	1	0.991952487	0	4
3090	GO:0006909	1	0.87415059	0	67
3091	GO:0006910	1	0.984032969	0	8
3092	GO:0006911	1	0.954795659	0	23
3093	GO:0006913	1	0.948822504	0	26
3094	GO:0006914	1	0.699736589	0	177
3096	GO:0006919	1	0.840601448	0	86
3097	GO:0006921	1	0.995966228	0	2
3098	GO:0006924	1	0.987937146	0	6
3099	GO:0006925	1	0.997996776	0	1
3100	GO:0006929	1	0.990071945	0	5
3101	GO:0006930	1	0.984082788	0	8
3102	GO:0006931	1	0.998013437	0	1
3103	GO:0006933	1	0.995992911	0	2
3105	GO:0006936	1	0.839157794	0	87
3106	GO:0006937	1	0.964165572	0	18
3107	GO:0006939	1	0.970411659	0	15
3108	GO:0006940	1	0.972173258	0	14
3109	GO:0006941	1	0.972234463	0	14

3110	GO:0006942	1	0.984007595	0	8
3111	GO:0006948	1	0.997996022	0	1
3112	GO:0006949	1	0.993992004	0	3
3114	GO:0006953	1	0.948812383	0	26
3117	GO:0006956	1	0.952774387	0	24
3118	GO:0006957	1	0.978138018	0	11
3119	GO:0006958	1	0.949037124	0	26
3120	GO:0006959	1	0.924288216	0	39
3121	GO:0006963	1	0.998011136	0	1
3122	GO:0006965	1	0.998011136	0	1
3123	GO:0006968	1	0.935622533	0	33
3124	GO:0006970	1	0.962572161	0	19
3125	GO:0006971	1	0.994035271	0	3
3126	GO:0006972	1	0.98604582	0	7
3128	GO:0006975	1	0.98402876	0	8
3129	GO:0006977	1	0.893279835	0	56
3130	GO:0006978	1	0.972195294	0	14
3131	GO:0006979	1	0.780007373	0	123
3132	GO:0006982	1	0.997974522	0	1
3133	GO:0006983	1	0.978196451	0	11
3134	GO:0006984	1	0.998013437	0	1
3135	GO:0006986	1	0.877162044	0	65
3136	GO:0006990	1	0.993976766	0	3
3137	GO:0006991	1	0.991987435	0	4
3138	GO:0006995	1	0.977959399	0	11
3139	GO:0006996	1	0.972304369	0	14
3140	GO:0006997	1	0.952820133	0	24
3141	GO:0006998	1	0.962491592	0	19
3142	GO:0006999	1	0.98409554	0	8
3143	GO:0007000	1	0.993989302	0	3
3144	GO:0007004	1	0.943307848	0	29
3145	GO:0007005	1	0.825593317	0	95
3146	GO:0007006	1	0.990046017	0	5
3147	GO:0007007	1	0.960441583	0	20
3148	GO:0007009	1	0.958862975	0	21
3149	GO:0007010	1	0.736573859	0	152
3150	GO:0007014	1	0.998004446	0	1
3151	GO:0007015	1	0.77596105	0	126
3152	GO:0007017	1	0.931671579	0	35
3153	GO:0007018	1	0.857114504	0	77
3154	GO:0007019	1	0.974240724	0	13
3155	GO:0007020	1	0.960721665	0	20
3156	GO:0007021	1	0.979962907	0	10
3157	GO:0007023	1	0.987972129	0	6
3158	GO:0007026	1	0.949243651	0	26

3159	GO:0007028	1	0.996030731	0	2
3160	GO:0007029	1	0.9246067	0	39
3161	GO:0007030	1	0.793704983	0	115
3162	GO:0007031	1	0.954776566	0	23
3163	GO:0007032	1	0.926558412	0	38
3164	GO:0007033	1	0.984070373	0	8
3165	GO:0007034	1	0.966267344	0	17
3166	GO:0007035	1	0.974239169	0	13
3167	GO:0007039	1	0.997987912	0	1
3168	GO:0007040	1	0.917308248	0	43
3169	GO:0007041	1	0.960768126	0	20
3170	GO:0007042	1	0.978002351	0	11
3171	GO:0007043	1	0.934284028	0	34
3172	GO:0007044	1	0.99207686	0	4
3174	GO:0007050	1	0.780424543	0	123
3175	GO:0007051	1	0.96639456	0	17
3176	GO:0007052	1	0.778756685	0	124
3177	GO:0007056	1	0.995965381	0	2
3178	GO:0007057	1	0.993945992	0	3
3179	GO:0007059	1	0.832644327	0	91
3180	GO:0007060	1	0.995986039	0	2
3181	GO:0007062	1	0.958744153	0	21
3182	GO:0007063	1	0.998001957	0	1
3183	GO:0007064	1	0.970393197	0	15
3184	GO:0007066	1	0.997959006	0	1
3185	GO:0007072	1	0.997973786	0	1
3186	GO:0007076	1	0.920893544	0	41
3187	GO:0007077	1	0.976160763	0	12
3188	GO:0007079	1	0.992030525	0	4
3189	GO:0007080	1	0.922498365	0	40
3190	GO:0007084	1	0.902287217	0	51
3191	GO:0007088	1	0.95673339	0	22
3192	GO:0007089	1	0.99200199	0	4
3193	GO:0007091	1	0.986073318	0	7
3194	GO:0007093	1	0.974214883	0	13
3195	GO:0007094	1	0.950905855	0	25
3196	GO:0007095	1	0.958746523	0	21
3197	GO:0007096	1	0.928001641	0	37
3198	GO:0007097	1	0.972423271	0	14
3199	GO:0007098	1	0.919030667	0	42
3200	GO:0007099	1	0.958825895	0	21
3201	GO:0007100	1	0.988054211	0	6
3202	GO:0007112	1	0.997990564	0	1
3203	GO:0007113	1	0.993936801	0	3
3204	GO:0007127	1	0.984028543	0	8

3205	GO:0007129	1	0.945252088	0	28
3206	GO:0007130	1	0.964414273	0	18
3207	GO:0007131	1	0.941397295	0	30
3208	GO:0007140	1	0.951108591	0	25
3209	GO:0007141	1	0.95473172	0	23
3210	GO:0007143	1	0.98412476	0	8
3211	GO:0007144	1	0.987994479	0	6
3212	GO:0007154	1	0.920788368	0	41
3213	GO:0007155	1	0.301706079	0	590
3214	GO:0007156	1	0.734350916	0	154
3215	GO:0007157	1	0.921037738	0	41
3216	GO:0007158	1	0.970479318	0	15
3217	GO:0007159	1	0.951095644	0	25
3218	GO:0007160	1	0.838193249	0	88
3219	GO:0007161	1	0.992048849	0	4
3220	GO:0007162	1	0.919294114	0	42
3221	GO:0007163	1	0.911593836	0	46
3224	GO:0007167	1	0.984138321	0	8
3225	GO:0007168	1	0.986011596	0	7
3226	GO:0007169	1	0.789023314	0	118
3227	GO:0007171	1	0.976245856	0	12
3228	GO:0007172	1	0.984073409	0	8
3229	GO:0007173	1	0.908247531	0	48
3230	GO:0007174	1	0.995966825	0	2
3231	GO:0007175	1	0.972244107	0	14
3232	GO:0007176	1	0.992021036	0	4
3233	GO:0007178	1	0.968534389	0	16
3234	GO:0007179	1	0.829584863	0	93
3235	GO:0007181	1	0.993987108	0	3
3236	GO:0007182	1	0.990045975	0	5
3237	GO:0007183	1	0.988026105	0	6
3238	GO:0007185	1	0.988095981	0	6
3240	GO:0007187	1	0.939288044	0	31
3241	GO:0007188	1	0.924515568	0	39
3243	GO:0007190	1	0.945293145	0	28
3244	GO:0007191	1	0.984032793	0	8
3245	GO:0007193	1	0.904461622	0	50
3246	GO:0007194	1	0.9702998	0	15
3247	GO:0007195	1	0.991999408	0	4
3248	GO:0007196	1	0.98810283	0	6
3249	GO:0007197	1	0.987983367	0	6
3250	GO:0007198	1	0.993971617	0	3
3251	GO:0007199	1	0.997987857	0	1
3252	GO:0007200	1	0.922606898	0	40
3253	GO:0007202	1	0.943308179	0	29

3254	GO:0007204	1	0.784822167	0	120
3255	GO:0007205	1	0.954882873	0	23
3256	GO:0007206	1	0.996026713	0	2
3257	GO:0007207	1	0.995976347	0	2
3258	GO:0007208	1	0.99798234	0	1
3259	GO:0007210	1	0.993941938	0	3
3260	GO:0007212	1	0.964454036	0	18
3261	GO:0007213	1	0.972173172	0	14
3262	GO:0007214	1	0.962647099	0	19
3263	GO:0007215	1	0.978262364	0	11
3264	GO:0007216	1	0.976275112	0	12
3265	GO:0007217	1	0.989958013	0	5
3266	GO:0007218	1	0.89478402	0	55
3267	GO:0007219	1	0.815978828	0	101
3268	GO:0007220	1	0.982064336	0	9
3270	GO:0007223	1	0.928589829	0	37
3271	GO:0007224	1	0.875829164	0	66
3272	GO:0007225	1	0.998012211	0	1
3273	GO:0007228	1	0.994000517	0	3
3275	GO:0007231	1	0.994033192	0	3
3276	GO:0007249	1	0.880672789	0	63
3277	GO:0007250	1	0.964444604	0	18
3278	GO:0007252	1	0.976267263	0	12
3279	GO:0007253	1	0.983939217	0	8
3280	GO:0007254	1	0.897156629	0	54
3281	GO:0007256	1	0.980197021	0	10
3282	GO:0007257	1	0.924843579	0	39
3283	GO:0007258	1	0.992004614	0	4
3285	GO:0007260	1	0.986070282	0	7
3286	GO:0007263	1	0.972146742	0	14
3287	GO:0007264	1	0.796766997	0	113
3288	GO:0007265	1	0.867025901	0	71
3289	GO:0007266	1	0.902553724	0	51
3291	GO:0007268	1	0.661512854	0	205
3292	GO:0007269	1	0.935804415	0	33
3293	GO:0007270	1	0.988076496	0	6
3294	GO:0007271	1	0.960503483	0	20
3295	GO:0007274	1	0.968447698	0	16
3296	GO:0007275	1	0.145745241	0	939
3297	GO:0007276	1	0.952819192	0	24
3298	GO:0007281	1	0.935943062	0	33
3299	GO:0007283	1	0.466446529	0	376
3300	GO:0007284	1	0.994023016	0	3
3301	GO:0007286	1	0.863250725	0	73
3302	GO:0007288	1	0.954721635	0	23

3303	GO:0007289	1	0.985975759	0	7
3304	GO:0007290	1	0.998012308	0	1
3305	GO:0007292	1	0.964362726	0	18
3306	GO:0007296	1	0.992036543	0	4
3307	GO:0007315	1	0.997985246	0	1
3308	GO:0007320	1	0.997972026	0	1
3309	GO:0007338	1	0.902275966	0	51
3310	GO:0007339	1	0.935488416	0	33
3311	GO:0007340	1	0.968279067	0	16
3312	GO:0007341	1	0.987942437	0	6
3313	GO:0007342	1	0.98183935	0	9
3314	GO:0007343	1	0.997983713	0	1
3315	GO:0007344	1	0.997974295	0	1
3316	GO:0007346	1	0.909657914	0	47
3317	GO:0007352	1	0.99600978	0	2
3318	GO:0007354	1	0.997998919	0	1
3319	GO:0007356	1	0.997974974	0	1
3320	GO:0007368	1	0.872069569	0	68
3321	GO:0007369	1	0.945273798	0	28
3322	GO:0007371	1	0.998004776	0	1
3323	GO:0007379	1	0.988016279	0	6
3324	GO:0007386	1	0.9960164	0	2
3325	GO:0007387	1	0.997989914	0	1
3326	GO:0007388	1	0.997989914	0	1
3328	GO:0007398	1	0.97424062	0	13
3329	GO:0007399	1	0.366538655	0	495
3330	GO:0007400	1	0.997986219	0	1
3331	GO:0007402	1	0.995947273	0	2
3332	GO:0007403	1	0.99800067	0	1
3333	GO:0007405	1	0.968459485	0	16
3334	GO:0007406	1	0.989998503	0	5
3335	GO:0007409	1	0.839702271	0	87
3336	GO:0007411	1	0.659482587	0	207
3337	GO:0007412	1	0.993950535	0	3
3338	GO:0007413	1	0.966538384	0	17
3339	GO:0007416	1	0.893955939	0	56
3340	GO:0007417	1	0.782257235	0	122
3341	GO:0007418	1	0.993997881	0	3
3342	GO:0007420	1	0.607748719	0	247
3343	GO:0007422	1	0.947102535	0	27
3344	GO:0007423	1	0.990027841	0	5
3345	GO:0007431	1	0.996015863	0	2
3346	GO:0007435	1	0.988060586	0	6
3347	GO:0007440	1	0.998013437	0	1
3348	GO:0007442	1	0.992034882	0	4

3349	GO:0007468	1	0.99797812	0	1
3350	GO:0007492	1	0.956820091	0	22
3351	GO:0007493	1	0.998001633	0	1
3352	GO:0007494	1	0.986063535	0	7
3353	GO:0007495	1	0.99799718	0	1
3354	GO:0007497	1	0.996026426	0	2
3355	GO:0007498	1	0.943388468	0	29
3356	GO:0007499	1	0.99801181	0	1
3357	GO:0007500	1	0.995972205	0	2
3358	GO:0007501	1	0.993987797	0	3
3359	GO:0007506	1	0.995993909	0	2
3360	GO:0007507	1	0.637851751	0	223
3361	GO:0007509	1	0.996007903	0	2
3362	GO:0007512	1	0.984055173	0	8
3364	GO:0007518	1	0.997984576	0	1
3365	GO:0007519	1	0.918893737	0	42
3366	GO:0007520	1	0.96465983	0	18
3367	GO:0007521	1	0.996017343	0	2
3368	GO:0007522	1	0.998013437	0	1
3369	GO:0007525	1	0.994026578	0	3
3370	GO:0007528	1	0.93609404	0	33
3371	GO:0007529	1	0.996019181	0	2
3372	GO:0007530	1	0.993948764	0	3
3373	GO:0007548	1	0.96243188	0	19
3374	GO:0007549	1	0.995985075	0	2
3375	GO:0007565	1	0.849293276	0	81
3376	GO:0007566	1	0.937553467	0	32
3377	GO:0007567	1	0.988034292	0	6
3378	GO:0007568	1	0.727014887	0	158
3379	GO:0007569	1	0.947201232	0	27
3381	GO:0007585	1	0.945246812	0	28
3382	GO:0007586	1	0.966146076	0	17
3383	GO:0007588	1	0.948986046	0	26
3384	GO:0007589	1	0.991941237	0	4
3385	GO:0007595	1	0.924733142	0	39
3386	GO:0007596	1	0.728470878	0	157
3387	GO:0007597	1	0.976035846	0	12
3388	GO:0007598	1	0.997984299	0	1
3389	GO:0007599	1	0.922511286	0	40
3390	GO:0007600	1	0.98404311	0	8
3391	GO:0007601	1	0.727292793	0	158
3392	GO:0007602	1	0.968222005	0	16
3393	GO:0007603	1	0.988030226	0	6
3394	GO:0007604	1	0.998001003	0	1
3395	GO:0007605	1	0.777592828	0	125

3396	GO:0007606	1	0.99195235	0	4
3397	GO:0007608	1	0.778044011	0	123
3398	GO:0007610	1	0.990036729	0	5
3399	GO:0007611	1	0.900912327	0	52
3400	GO:0007612	1	0.89034678	0	58
3401	GO:0007613	1	0.872411266	0	68
3402	GO:0007614	1	0.978107045	0	11
3403	GO:0007616	1	0.943582451	0	29
3404	GO:0007617	1	0.987990339	0	6
3405	GO:0007618	1	0.997961445	0	1
3406	GO:0007620	1	0.995967866	0	2
3407	GO:0007621	1	0.994007019	0	3
3408	GO:0007622	1	0.997992206	0	1
3409	GO:0007623	1	0.853048928	0	79
3410	GO:0007624	1	0.996004823	0	2
3411	GO:0007625	1	0.982044335	0	9
3412	GO:0007626	1	0.879584025	0	64
3413	GO:0007628	1	0.953053054	0	24
3414	GO:0007631	1	0.970198517	0	15
3415	GO:0007632	1	0.991993255	0	4
3416	GO:0007635	1	0.992004693	0	4
3417	GO:0007638	1	0.996030731	0	2
3419	GO:0008013	1	0.84848607	0	82
3421	GO:0008016	1	0.949005322	0	26
3423	GO:0008020	1	0.985945067	0	7
3424	GO:0008021	1	0.761760784	0	135
3425	GO:0008022	1	0.673470373	0	196
3426	GO:0008023	1	0.95870019	0	21
3427	GO:0008024	1	0.984097623	0	8
3428	GO:0008028	1	0.968337291	0	16
3429	GO:0008033	1	0.815270334	0	101
3430	GO:0008035	1	0.981999239	0	9
3431	GO:0008037	1	0.984069355	0	8
3432	GO:0008038	1	0.990051388	0	5
3433	GO:0008039	1	0.997994566	0	1
3434	GO:0008043	1	0.995926424	0	2
3435	GO:0008045	1	0.947168829	0	27
3436	GO:0008046	1	0.982183439	0	9
3437	GO:0008047	1	0.907725475	0	48
3438	GO:0008048	1	0.995963929	0	2
3439	GO:0008049	1	0.997977061	0	1
3440	GO:0008050	1	0.996002728	0	2
3441	GO:0008052	1	0.99796016	0	1
3442	GO:0008053	1	0.96827075	0	16
3443	GO:0008057	1	0.998013437	0	1

3444	GO:0008061	1	0.98598817	0	7
3445	GO:0008063	1	0.988026545	0	6
3446	GO:0008064	1	0.98399282	0	8
3447	GO:0008065	1	0.994034036	0	3
3448	GO:0008066	1	0.978290775	0	11
3449	GO:0008073	1	0.993910101	0	3
3450	GO:0008074	1	0.995985075	0	2
3451	GO:0008076	1	0.860023422	0	75
3452	GO:0008078	1	0.995928581	0	2
3453	GO:0008079	1	0.998004159	0	1
3454	GO:0008080	1	0.954727885	0	23
3455	GO:0008081	1	0.901090159	0	52
3456	GO:0008083	1	0.782779569	0	121
3457	GO:0008088	1	0.992026873	0	4
3458	GO:0008089	1	0.939584259	0	31
3459	GO:0008090	1	0.978176685	0	11
3460	GO:0008091	1	0.984202978	0	8
3461	GO:0008092	1	0.884802786	0	61
3462	GO:0008093	1	0.962626755	0	19
3463	GO:0008094	1	0.930478631	0	36
3464	GO:0008097	1	0.979917322	0	10
3465	GO:0008104	1	0.758594178	0	137
3466	GO:0008106	1	0.991911661	0	4
3467	GO:0008107	1	0.99600925	0	2
3468	GO:0008108	1	0.997966837	0	1
3469	GO:0008109	1	0.994005938	0	3
3470	GO:0008111	1	0.997978178	0	1
3471	GO:0008112	1	0.99797125	0	1
3472	GO:0008113	1	0.995940363	0	2
3473	GO:0008115	1	0.997985293	0	1
3474	GO:0008116	1	0.998013426	0	1
3475	GO:0008117	1	0.996025123	0	2
3476	GO:0008118	1	0.995960557	0	2
3477	GO:0008119	1	0.997998144	0	1
3478	GO:0008120	1	0.997972277	0	1
3479	GO:0008121	1	0.981835356	0	9
3480	GO:0008124	1	0.995949074	0	2
3481	GO:0008126	1	0.9919707	0	4
3482	GO:0008127	1	0.997966097	0	1
3483	GO:0008131	1	0.98803597	0	6
3484	GO:0008134	1	0.54520732	0	300
3485	GO:0008135	1	0.956734648	0	22
3486	GO:0008137	1	0.914223346	0	44
3487	GO:0008138	1	0.924461387	0	39
3488	GO:0008139	1	0.951180846	0	25

3489	GO:0008140	1	0.980051611	0	10
3490	GO:0008142	1	0.986060807	0	7
3491	GO:0008143	1	0.960661362	0	20
3492	GO:0008144	1	0.982110534	0	9
3493	GO:0008146	1	0.913314307	0	45
3494	GO:0008147	1	0.99590833	0	2
3495	GO:0008150	1	0.383384064	0	471
3496	GO:0008152	1	0.708499552	0	171
3497	GO:0008154	1	0.960566793	0	20
3498	GO:0008156	1	0.968353383	0	16
3499	GO:0008157	1	0.947193064	0	27
3500	GO:0008158	1	0.993992156	0	3
3501	GO:0008160	1	0.995982947	0	2
3503	GO:0008170	1	0.987922209	0	6
3504	GO:0008171	1	0.983945589	0	8
3505	GO:0008173	1	0.974205623	0	13
3506	GO:0008174	1	0.998013437	0	1
3507	GO:0008175	1	0.982100405	0	9
3508	GO:0008176	1	0.995944175	0	2
3509	GO:0008177	1	0.99595217	0	2
3510	GO:0008179	1	0.976238836	0	12
3511	GO:0008180	1	0.931937771	0	35
3512	GO:0008184	1	0.994013624	0	3
3513	GO:0008186	1	0.990066501	0	5
3514	GO:0008187	1	0.995998301	0	2
3515	GO:0008188	1	0.976079227	0	12
3516	GO:0008190	1	0.980095658	0	10
3517	GO:0008191	1	0.970184272	0	15
3518	GO:0008192	1	0.998011042	0	1
3519	GO:0008193	1	0.997966211	0	1
3520	GO:0008194	1	0.962360554	0	19
3521	GO:0008195	1	0.97620974	0	12
3522	GO:0008198	1	0.958575283	0	21
3523	GO:0008199	1	0.985950349	0	7
3524	GO:0008200	1	0.986004315	0	7
3528	GO:0008206	1	0.970269418	0	15
3529	GO:0008207	1	0.989920253	0	5
3530	GO:0008209	1	0.964336709	0	18
3531	GO:0008210	1	0.945039219	0	28
3532	GO:0008211	1	0.989946947	0	5
3533	GO:0008215	1	0.995950776	0	2
3534	GO:0008216	1	0.997963945	0	1
3535	GO:0008217	1	0.897021462	0	54
3536	GO:0008219	1	0.928329802	0	37
3537	GO:0008228	1	0.993991279	0	3

3538	GO:0008233	1	0.410084704	0	439
3539	GO:0008234	1	0.768380399	0	131
3540	GO:0008235	1	0.962593774	0	19
3541	GO:0008236	1	0.795799147	0	113
3542	GO:0008237	1	0.745302743	0	146
3543	GO:0008238	1	0.991975986	0	4
3544	GO:0008239	1	0.980138631	0	10
3545	GO:0008240	1	0.994025854	0	3
3546	GO:0008241	1	0.996005665	0	2
3547	GO:0008242	1	0.993935396	0	3
3548	GO:0008247	1	0.993951207	0	3
3549	GO:0008250	1	0.975960459	0	12
3550	GO:0008251	1	0.990072395	0	5
3551	GO:0008252	1	0.995934562	0	2
3552	GO:0008253	1	0.976151557	0	12
3553	GO:0008254	1	0.997990564	0	1
3554	GO:0008260	1	0.99598212	0	2
3555	GO:0008263	1	0.995988258	0	2
3556	GO:0008265	1	0.997990293	0	1
3557	GO:0008266	1	0.954866584	0	23
3558	GO:0008269	1	0.997982002	0	1
3560	GO:0008271	1	0.978225086	0	11
3561	GO:0008272	1	0.966459532	0	17
3562	GO:0008273	1	0.992036421	0	4
3563	GO:0008274	1	0.984120281	0	8
3564	GO:0008275	1	0.992054052	0	4
3565	GO:0008276	1	0.978051808	0	11
3566	GO:0008277	1	0.920882552	0	41
3567	GO:0008278	1	0.984164771	0	8
3568	GO:0008281	1	0.996028827	0	2
3569	GO:0008282	1	0.994032173	0	3
3570	GO:0008283	1	0.749590306	0	143
3573	GO:0008286	1	0.85309659	0	79
3574	GO:0008287	1	0.992006666	0	4
3576	GO:0008290	1	0.986019186	0	7
3577	GO:0008292	1	0.997988914	0	1
3578	GO:0008294	1	0.998013437	0	1
3579	GO:0008295	1	0.993957788	0	3
3580	GO:0008296	1	0.987942166	0	6
3581	GO:0008297	1	0.997990564	0	1
3582	GO:0008298	1	0.996013967	0	2
3583	GO:0008299	1	0.97213478	0	14
3584	GO:0008301	1	0.970252127	0	15
3585	GO:0008305	1	0.94943658	0	26
3586	GO:0008306	1	0.956683435	0	22

3587	GO:0008307	1	0.928175621	0	37
3588	GO:0008308	1	0.989912939	0	5
3589	GO:0008309	1	0.995956263	0	2
3590	GO:0008310	1	0.989985187	0	5
3591	GO:0008311	1	0.995951927	0	2
3592	GO:0008312	1	0.985933065	0	7
3593	GO:0008315	1	0.997970155	0	1
3594	GO:0008318	1	0.99397702	0	3
3595	GO:0008320	1	0.956519761	0	22
3596	GO:0008324	1	0.96451716	0	18
3597	GO:0008327	1	0.958667973	0	21
3598	GO:0008328	1	0.978338416	0	11
3599	GO:0008330	1	0.980076207	0	10
3600	GO:0008331	1	0.982194869	0	9
3601	GO:0008332	1	0.996030731	0	2
3602	GO:0008333	1	0.915465488	0	44
3603	GO:0008334	1	0.975967136	0	12
3604	GO:0008336	1	0.997976757	0	1
3605	GO:0008340	1	0.964561678	0	18
3606	GO:0008343	1	0.991984673	0	4
3607	GO:0008344	1	0.917272253	0	43
3608	GO:0008347	1	0.980079925	0	10
3609	GO:0008349	1	0.992027551	0	4
3610	GO:0008352	1	0.997989131	0	1
3611	GO:0008353	1	0.976186235	0	12
3612	GO:0008354	1	0.986064873	0	7
3613	GO:0008355	1	0.996010953	0	2
3614	GO:0008356	1	0.990047261	0	5
3615	GO:0008360	1	0.754476779	0	140
3616	GO:0008361	1	0.956891953	0	22
3617	GO:0008366	1	0.988053771	0	6
3618	GO:0008373	1	0.964430273	0	18
3619	GO:0008374	1	0.968287275	0	16
3620	GO:0008375	1	0.932002578	0	35
3621	GO:0008376	1	0.95485138	0	23
3622	GO:0008378	1	0.982057361	0	9
3623	GO:0008379	1	0.989847384	0	5
3624	GO:0008380	1	0.552135954	0	293
3625	GO:0008381	1	0.974276475	0	13
3626	GO:0008384	1	0.994019382	0	3
3627	GO:0008385	1	0.986043377	0	7
3628	GO:0008386	1	0.997978128	0	1
3629	GO:0008387	1	0.99798314	0	1
3630	GO:0008389	1	0.997974644	0	1
3631	GO:0008390	1	0.997995231	0	1

3632	GO:0008391	1	0.985982058	0	7
3633	GO:0008392	1	0.9701103	0	15
3634	GO:0008395	1	0.944951942	0	28
3635	GO:0008396	1	0.995972105	0	2
3636	GO:0008397	1	0.998007019	0	1
3637	GO:0008398	1	0.997995521	0	1
3638	GO:0008401	1	0.981997988	0	9
3639	GO:0008403	1	0.997997008	0	1
3640	GO:0008404	1	0.99394172	0	3
3641	GO:0008405	1	0.995956563	0	2
3642	GO:0008406	1	0.980027497	0	10
3643	GO:0008408	1	0.958626623	0	21
3644	GO:0008409	1	0.974259657	0	13
3645	GO:0008410	1	0.991970648	0	4
3646	GO:0008413	1	0.995937479	0	2
3647	GO:0008417	1	0.980162115	0	10
3648	GO:0008418	1	0.99595709	0	2
3649	GO:0008419	1	0.997988991	0	1
3650	GO:0008420	1	0.986058515	0	7
3651	GO:0008422	1	0.992005628	0	4
3652	GO:0008424	1	0.99799873	0	1
3653	GO:0008425	1	0.997965466	0	1
3654	GO:0008426	1	0.993941287	0	3
3655	GO:0008427	1	0.993923328	0	3
3656	GO:0008428	1	0.997976875	0	1
3657	GO:0008429	1	0.980050909	0	10
3658	GO:0008430	1	0.983997357	0	8
3659	GO:0008431	1	0.995987785	0	2
3660	GO:0008432	1	0.982085646	0	9
3661	GO:0008437	1	0.997978686	0	1
3662	GO:0008440	1	0.991987031	0	4
3663	GO:0008441	1	0.995980566	0	2
3664	GO:0008442	1	0.997978253	0	1
3665	GO:0008443	1	0.997997597	0	1
3666	GO:0008444	1	0.995991181	0	2
3667	GO:0008445	1	0.997972026	0	1
3668	GO:0008446	1	0.997973062	0	1
3669	GO:0008448	1	0.997975827	0	1
3670	GO:0008449	1	0.992050225	0	4
3671	GO:0008452	1	0.997990564	0	1
3672	GO:0008453	1	0.997971738	0	1
3673	GO:0008454	1	0.993992321	0	3
3674	GO:0008455	1	0.997989828	0	1
3675	GO:0008456	1	0.998004621	0	1
3676	GO:0008457	1	0.991982611	0	4

3677	GO:0008458	1	0.99399737	0	3
3678	GO:0008459	1	0.996000937	0	2
3679	GO:0008460	1	0.997977061	0	1
3680	GO:0008465	1	0.997964949	0	1
3681	GO:0008466	1	0.995978962	0	2
3682	GO:0008467	1	0.987940208	0	6
3683	GO:0008469	1	0.987978785	0	6
3684	GO:0008470	1	0.998011761	0	1
3685	GO:0008474	1	0.972154078	0	14
3686	GO:0008475	1	0.994008545	0	3
3687	GO:0008476	1	0.995961175	0	2
3688	GO:0008478	1	0.998013437	0	1
3689	GO:0008479	1	0.995961394	0	2
3690	GO:0008480	1	0.997999042	0	1
3691	GO:0008481	1	0.99597412	0	2
3692	GO:0008482	1	0.997984014	0	1
3693	GO:0008483	1	0.960549609	0	20
3694	GO:0008484	1	0.964575644	0	18
3695	GO:0008486	1	0.98997282	0	5
3696	GO:0008488	1	0.997996971	0	1
3697	GO:0008489	1	0.994029112	0	3
3698	GO:0008493	1	0.997976306	0	1
3699	GO:0008494	1	0.982031239	0	9
3700	GO:0008495	1	0.997994374	0	1
3701	GO:0008499	1	0.987997267	0	6
3702	GO:0008502	1	0.995969368	0	2
3703	GO:0008503	1	0.986013436	0	7
3704	GO:0008504	1	0.988044126	0	6
3705	GO:0008506	1	0.99199124	0	4
3706	GO:0008507	1	0.998002817	0	1
3707	GO:0008508	1	0.987930703	0	6
3708	GO:0008509	1	0.974383636	0	13
3709	GO:0008510	1	0.99008868	0	5
3710	GO:0008511	1	0.99405058	0	3
3711	GO:0008513	1	0.995961911	0	2
3712	GO:0008514	1	0.978235638	0	11
3713	GO:0008517	1	0.992026947	0	4
3714	GO:0008518	1	0.997991888	0	1
3715	GO:0008519	1	0.980106993	0	10
3716	GO:0008520	1	0.99600176	0	2
3717	GO:0008521	1	0.997989115	0	1
3718	GO:0008523	1	0.99799894	0	1
3719	GO:0008525	1	0.988032944	0	6
3720	GO:0008526	1	0.980106656	0	10
3721	GO:0008527	1	0.977875851	0	11

3722	GO:0008528	1	0.94509328	0	28
3723	GO:0008531	1	0.997989828	0	1
3724	GO:0008532	1	0.980054532	0	10
3725	GO:0008534	1	0.997977171	0	1
3726	GO:0008535	1	0.984006584	0	8
3727	GO:0008537	1	0.993928355	0	3
3728	GO:0008540	1	0.975994274	0	12
3729	GO:0008541	1	0.983916823	0	8
3730	GO:0008542	1	0.924780386	0	39
3731	GO:0008543	1	0.868618328	0	70
3732	GO:0008544	1	0.83691571	0	88
3733	GO:0008545	1	0.996008728	0	2
3734	GO:0008553	1	0.986057239	0	7
3735	GO:0008556	1	0.990047305	0	5
3736	GO:0008559	1	0.972476247	0	14
3737	GO:0008568	1	0.984122458	0	8
3738	GO:0008569	1	0.966701247	0	17
3739	GO:0008574	1	0.966688713	0	17
3740	GO:0008579	1	0.99801328	0	1
3741	GO:0008582	1	0.99003669	0	5
3742	GO:0008584	1	0.827605499	0	94
3743	GO:0008585	1	0.958817503	0	21
3744	GO:0008589	1	0.960760207	0	20
3745	GO:0008592	1	0.994020076	0	3
3746	GO:0008593	1	0.960621872	0	20
3747	GO:0008594	1	0.992003936	0	4
3748	GO:0008595	1	0.988079455	0	6
3749	GO:0008597	1	0.991975906	0	4
3751	GO:0008607	1	0.993977411	0	3
3752	GO:0008608	1	0.978078139	0	11
3753	GO:0008609	1	0.998013437	0	1
3755	GO:0008611	1	0.986039782	0	7
3756	GO:0008612	1	0.995944669	0	2
3757	GO:0008615	1	0.995985754	0	2
3758	GO:0008617	1	0.997960497	0	1
3759	GO:0008622	1	0.989950267	0	5
3760	GO:0008623	1	0.99399468	0	3
3761	GO:0008625	1	0.94312656	0	29
3762	GO:0008626	1	0.998013436	0	1
3763	GO:0008627	1	0.996005372	0	2
3764	GO:0008630	1	0.911578268	0	46
3765	GO:0008631	1	0.972301441	0	14
3766	GO:0008635	1	0.985992022	0	7
3768	GO:0008641	1	0.980115463	0	10
3769	GO:0008643	1	0.928271925	0	37

3770	GO:0008645	1	0.978118158	0	11
3771	GO:0008649	1	0.98593308	0	7
3772	GO:0008650	1	0.995997147	0	2
3773	GO:0008652	1	0.950809266	0	25
3774	GO:0008653	1	0.997984931	0	1
3775	GO:0008654	1	0.895178942	0	55
3776	GO:0008655	1	0.997959405	0	1
3777	GO:0008656	1	0.970232381	0	15
3778	GO:0008659	1	0.996007903	0	2
3779	GO:0008663	1	0.997990564	0	1
3780	GO:0008667	1	0.997993203	0	1
3781	GO:0008670	1	0.995940739	0	2
3782	GO:0008682	1	0.997987951	0	1
3783	GO:0008689	1	0.997965466	0	1
3784	GO:0008693	1	0.996007903	0	2
3785	GO:0008700	1	0.997990564	0	1
3786	GO:0008705	1	0.998013437	0	1
3787	GO:0008709	1	0.997959295	0	1
3788	GO:0008720	1	0.997979224	0	1
3789	GO:0008721	1	0.997986313	0	1
3790	GO:0008725	1	0.997961976	0	1
3791	GO:0008732	1	0.995972487	0	2
3792	GO:0008745	1	0.993928168	0	3
3793	GO:0008746	1	0.99597784	0	2
3794	GO:0008747	1	0.997970941	0	1
3795	GO:0008753	1	0.994011726	0	3
3796	GO:0008757	1	0.970132388	0	15
3797	GO:0008761	1	0.998013293	0	1
3798	GO:0008768	1	0.995922269	0	2
3799	GO:0008775	1	0.997975647	0	1
3800	GO:0008781	1	0.995943222	0	2
3801	GO:0008783	1	0.997981841	0	1
3802	GO:0008785	1	0.99797105	0	1
3803	GO:0008794	1	0.997958486	0	1
3804	GO:0008796	1	0.997960041	0	1
3805	GO:0008798	1	0.997984749	0	1
3806	GO:0008802	1	0.998002542	0	1
3807	GO:0008803	1	0.997960041	0	1
3808	GO:0008808	1	0.99800547	0	1
3809	GO:0008811	1	0.997956978	0	1
3810	GO:0008812	1	0.998003586	0	1
3811	GO:0008817	1	0.998008808	0	1
3812	GO:0008821	1	0.987991562	0	6
3813	GO:0008823	1	0.994042865	0	3
3814	GO:0008828	1	0.997954504	0	1

3815	GO:0008832	1	0.997997404	0	1
3816	GO:0008843	1	0.997983681	0	1
3817	GO:0008852	1	0.997991795	0	1
3818	GO:0008853	1	0.991953973	0	4
3819	GO:0008859	1	0.997959385	0	1
3820	GO:0008865	1	0.992034495	0	4
3821	GO:0008887	1	0.997997967	0	1
3822	GO:0008889	1	0.996004245	0	2
3823	GO:0008890	1	0.997971394	0	1
3824	GO:0008892	1	0.998013378	0	1
3825	GO:0008893	1	0.99796111	0	1
3826	GO:0008894	1	0.997990309	0	1
3827	GO:0008897	1	0.997992244	0	1
3828	GO:0008900	1	0.995976344	0	2
3829	GO:0008903	1	0.997962427	0	1
3830	GO:0008929	1	0.997969123	0	1
3831	GO:0008934	1	0.993978668	0	3
3832	GO:0008940	1	0.994002664	0	3
3833	GO:0008941	1	0.997986766	0	1
3834	GO:0008948	1	0.991990571	0	4
3835	GO:0008955	1	0.99800712	0	1
3836	GO:0008962	1	0.997987974	0	1
3837	GO:0008963	1	0.997980984	0	1
3838	GO:0008967	1	0.995979309	0	2
3839	GO:0008970	1	0.980054909	0	10
3840	GO:0008973	1	0.9979981	0	1
3841	GO:0008983	1	0.997990564	0	1
3842	GO:0008988	1	0.995954383	0	2
3843	GO:0008995	1	0.997988044	0	1
3844	GO:0009000	1	0.997986094	0	1
3845	GO:0009007	1	0.996020318	0	2
3846	GO:0009008	1	0.992019296	0	4
3847	GO:0009013	1	0.998013139	0	1
3848	GO:0009019	1	0.992008481	0	4
3849	GO:0009020	1	0.997977919	0	1
3850	GO:0009032	1	0.995952194	0	2
3852	GO:0009048	1	0.98021513	0	10
3853	GO:0009051	1	0.991959935	0	4
3854	GO:0009052	1	0.98995041	0	5
3855	GO:0009055	1	0.860785691	0	74
3856	GO:0009056	1	0.993993864	0	3
3857	GO:0009058	1	0.931897759	0	35
3858	GO:0009060	1	0.939114926	0	31
3859	GO:0009062	1	0.974258793	0	13
3860	GO:0009063	1	0.993948543	0	3

3861	GO:0009064	1	0.974238879	0	13
3862	GO:0009066	1	0.97811806	0	11
3863	GO:0009069	1	0.995991566	0	2
3864	GO:0009070	1	0.99800132	0	1
3865	GO:0009071	1	0.997980496	0	1
3866	GO:0009072	1	0.987921683	0	6
3867	GO:0009074	1	0.997990727	0	1
3868	GO:0009078	1	0.995987498	0	2
3869	GO:0009081	1	0.995989427	0	2
3870	GO:0009082	1	0.995989427	0	2
3871	GO:0009083	1	0.960506734	0	20
3872	GO:0009086	1	0.978105671	0	11
3873	GO:0009087	1	0.998000404	0	1
3874	GO:0009097	1	0.993935403	0	3
3875	GO:0009098	1	0.995989427	0	2
3876	GO:0009099	1	0.993980916	0	3
3877	GO:0009100	1	0.976134973	0	12
3878	GO:0009101	1	0.976199764	0	12
3879	GO:0009104	1	0.997984931	0	1
3880	GO:0009106	1	0.998002843	0	1
3881	GO:0009107	1	0.997973035	0	1
3882	GO:0009113	1	0.988018798	0	6
3883	GO:0009115	1	0.998013436	0	1
3884	GO:0009116	1	0.962315875	0	19
3886	GO:0009124	1	0.997981389	0	1
3887	GO:0009132	1	0.997960497	0	1
3888	GO:0009133	1	0.997981389	0	1
3889	GO:0009134	1	0.984052275	0	8
3891	GO:0009143	1	0.987967856	0	6
3892	GO:0009152	1	0.997969493	0	1
3893	GO:0009154	1	0.997956413	0	1
3894	GO:0009156	1	0.987990869	0	6
3895	GO:0009157	1	0.991957188	0	4
3896	GO:0009159	1	0.997990564	0	1
3897	GO:0009165	1	0.976083292	0	12
3898	GO:0009166	1	0.993980613	0	3
3899	GO:0009168	1	0.968308679	0	16
3900	GO:0009181	1	0.997979751	0	1
3901	GO:0009190	1	0.968548544	0	16
3902	GO:0009191	1	0.997964351	0	1
3903	GO:0009200	1	0.998012703	0	1
3904	GO:0009204	1	0.99796226	0	1
3905	GO:0009214	1	0.998013218	0	1
3906	GO:0009217	1	0.997978935	0	1
3907	GO:0009220	1	0.993986692	0	3

3908	GO:0009223	1	0.995934562	0	2
3909	GO:0009225	1	0.995990748	0	2
3910	GO:0009226	1	0.997990564	0	1
3911	GO:0009229	1	0.997984426	0	1
3912	GO:0009231	1	0.997989828	0	1
3913	GO:0009234	1	0.998003649	0	1
3914	GO:0009235	1	0.964351161	0	18
3915	GO:0009236	1	0.994025922	0	3
3916	GO:0009240	1	0.997980805	0	1
3917	GO:0009247	1	0.983992677	0	8
3918	GO:0009249	1	0.987850005	0	6
3919	GO:0009253	1	0.991900375	0	4
3920	GO:0009256	1	0.997984426	0	1
3921	GO:0009257	1	0.996008463	0	2
3922	GO:0009258	1	0.996013877	0	2
3923	GO:0009259	1	0.995955454	0	2
3924	GO:0009262	1	0.997999696	0	1
3925	GO:0009263	1	0.994021545	0	3
3926	GO:0009264	1	0.991925868	0	4
3927	GO:0009266	1	0.983966224	0	8
3928	GO:0009267	1	0.868740724	0	70
3929	GO:0009268	1	0.976186719	0	12
3930	GO:0009295	1	0.997952806	0	1
3931	GO:0009298	1	0.991920296	0	4
3932	GO:0009299	1	0.98802548	0	6
3933	GO:0009301	1	0.991992155	0	4
3934	GO:0009303	1	0.974157549	0	13
3935	GO:0009304	1	0.996020652	0	2
3936	GO:0009305	1	0.998013437	0	1
3937	GO:0009306	1	0.907871668	0	48
3938	GO:0009308	1	0.984023536	0	8
3939	GO:0009309	1	0.997963974	0	1
3940	GO:0009311	1	0.960498148	0	20
3941	GO:0009312	1	0.95669842	0	22
3942	GO:0009313	1	0.980111743	0	10
3943	GO:0009314	1	0.941507345	0	30
3944	GO:0009317	1	0.99797563	0	1
3945	GO:0009328	1	0.995962849	0	2
3946	GO:0009330	1	0.998013436	0	1
3947	GO:0009331	1	0.994024275	0	3
3948	GO:0009353	1	0.99796661	0	1
3949	GO:0009360	1	0.997989735	0	1
3950	GO:0009361	1	0.997969141	0	1
3951	GO:0009368	1	0.995951688	0	2
3952	GO:0009374	1	0.990047113	0	5

3953	GO:0009378	1	0.98811046	0	6
3954	GO:0009383	1	0.993997165	0	3
3955	GO:0009384	1	0.998013293	0	1
3956	GO:0009386	1	0.995959048	0	2
3957	GO:0009395	1	0.966451228	0	17
3958	GO:0009396	1	0.995948428	0	2
3959	GO:0009398	1	0.997989828	0	1
3960	GO:0009403	1	0.99799634	0	1
3961	GO:0009404	1	0.98213242	0	9
3962	GO:0009405	1	0.998013364	0	1
3963	GO:0009408	1	0.922517018	0	40
3965	GO:0009410	1	0.974178182	0	13
3966	GO:0009411	1	0.907890201	0	48
3967	GO:0009414	1	0.989973643	0	5
3968	GO:0009416	1	0.954776116	0	23
3969	GO:0009435	1	0.978108301	0	11
3970	GO:0009436	1	0.995966285	0	2
3971	GO:0009437	1	0.984048521	0	8
3972	GO:0009438	1	0.995942324	0	2
3973	GO:0009440	1	0.995936174	0	2
3974	GO:0009441	1	0.998013437	0	1
3975	GO:0009443	1	0.998013437	0	1
3976	GO:0009444	1	0.998013301	0	1
3977	GO:0009445	1	0.997990564	0	1
3978	GO:0009446	1	0.993946976	0	3
3979	GO:0009447	1	0.995940064	0	2
3980	GO:0009448	1	0.994016497	0	3
3981	GO:0009449	1	0.994012718	0	3
3982	GO:0009450	1	0.996029704	0	2
3983	GO:0009451	1	0.972144686	0	14
3984	GO:0009452	1	0.998004245	0	1
3985	GO:0009566	1	0.924521735	0	39
3986	GO:0009582	1	0.994002571	0	3
3987	GO:0009583	1	0.995993111	0	2
3988	GO:0009584	1	0.985990578	0	7
3989	GO:0009593	1	0.997956413	0	1
3990	GO:0009595	1	0.994043839	0	3
3992	GO:0009609	1	0.989950026	0	5
3993	GO:0009611	1	0.879132747	0	64
3997	GO:0009620	1	0.990006166	0	5
3998	GO:0009624	1	0.995956195	0	2
3999	GO:0009629	1	0.987932899	0	6
4000	GO:0009631	1	0.99401344	0	3
4001	GO:0009635	1	0.989956427	0	5
4002	GO:0009636	1	0.842637333	0	85

4003	GO:0009637	1	0.991965978	0	4
4004	GO:0009642	1	0.986049367	0	7
4005	GO:0009644	1	0.997980022	0	1
4006	GO:0009645	1	0.998001484	0	1
4007	GO:0009648	1	0.993951238	0	3
4008	GO:0009649	1	0.98798459	0	6
4009	GO:0009650	1	0.974138771	0	13
4010	GO:0009651	1	0.984058862	0	8
4011	GO:0009653	1	0.806141111	0	107
4012	GO:0009673	1	0.998003837	0	1
4013	GO:0009682	1	0.997989906	0	1
4014	GO:0009692	1	0.997988324	0	1
4015	GO:0009725	1	0.902412367	0	51
4016	GO:0009740	1	0.997995376	0	1
4017	GO:0009743	1	0.978131493	0	11
4018	GO:0009744	1	0.988067721	0	6
4019	GO:0009749	1	0.875562662	0	66
4020	GO:0009750	1	0.988028623	0	6
4021	GO:0009751	1	0.99798686	0	1
4022	GO:0009755	1	0.93190509	0	35
4023	GO:0009756	1	0.995985854	0	2
4024	GO:0009785	1	0.996011072	0	2
4025	GO:0009786	1	0.991967547	0	4
4026	GO:0009789	1	0.998010357	0	1
4027	GO:0009791	1	0.883172097	0	62
4028	GO:0009792	1	0.958758902	0	21
4029	GO:0009794	1	0.997987888	0	1
4030	GO:0009798	1	0.995930946	0	2
4031	GO:0009804	1	0.985943185	0	7
4032	GO:0009812	1	0.987920958	0	6
4033	GO:0009820	1	0.995971725	0	2
4034	GO:0009822	1	0.993948013	0	3
4035	GO:0009826	1	0.995993425	0	2
4036	GO:0009838	1	0.987946337	0	6
4037	GO:0009841	1	0.99798441	0	1
4038	GO:0009855	1	0.997998511	0	1
4039	GO:0009880	1	0.952948411	0	24
4040	GO:0009881	1	0.987996036	0	6
4041	GO:0009882	1	0.996011072	0	2
4042	GO:0009887	1	0.753040281	0	141
4043	GO:0009888	1	0.930567343	0	36
4044	GO:0009890	1	0.995918007	0	2
4045	GO:0009892	1	0.998000656	0	1
4046	GO:0009893	1	0.995993006	0	2
4047	GO:0009895	1	0.997997716	0	1

4049	GO:0009898	1	0.904545378	0	50
4050	GO:0009912	1	0.994022477	0	3
4051	GO:0009913	1	0.98407866	0	8
4052	GO:0009914	1	0.997983649	0	1
4053	GO:0009917	1	0.997985764	0	1
4055	GO:0009922	1	0.987988928	0	6
4056	GO:0009923	1	0.995989933	0	2
4057	GO:0009925	1	0.908251425	0	48
4058	GO:0009931	1	0.986021458	0	7
4059	GO:0009946	1	0.997986922	0	1
4060	GO:0009948	1	0.97217743	0	14
4061	GO:0009949	1	0.997971213	0	1
4062	GO:0009950	1	0.986015852	0	7
4063	GO:0009952	1	0.875770046	0	66
4064	GO:0009953	1	0.932112375	0	35
4065	GO:0009954	1	0.96852959	0	16
4066	GO:0009956	1	0.998006674	0	1
4067	GO:0009957	1	0.994017593	0	3
4068	GO:0009966	1	0.917178878	0	43
4069	GO:0009967	1	0.984113384	0	8
4070	GO:0009968	1	0.887949661	0	59
4071	GO:0009972	1	0.977957735	0	11
4072	GO:0009982	1	0.974098745	0	13
4074	GO:0009987	1	0.976157977	0	12
4075	GO:0009988	1	0.98801143	0	6
4076	GO:0009991	1	0.991936187	0	4
4077	GO:0009992	1	0.989964792	0	5
4078	GO:0009994	1	0.99394878	0	3
4079	GO:0010001	1	0.972370473	0	14
4080	GO:0010002	1	0.998013192	0	1
4082	GO:0010021	1	0.997996716	0	1
4083	GO:0010025	1	0.995997464	0	2
4084	GO:0010032	1	0.988093883	0	6
4085	GO:0010033	1	0.851006163	0	80
4086	GO:0010034	1	0.998002211	0	1
4087	GO:0010035	1	0.970318538	0	15
4088	GO:0010038	1	0.968239189	0	16
4089	GO:0010039	1	0.968389123	0	16
4090	GO:0010040	1	0.991963509	0	4
4091	GO:0010041	1	0.993997653	0	3
4092	GO:0010042	1	0.986013246	0	7
4093	GO:0010043	1	0.944983349	0	28
4094	GO:0010044	1	0.991978028	0	4
4095	GO:0010046	1	0.993926247	0	3
4096	GO:0010070	1	0.997985293	0	1

4097	GO:0010106	1	0.997977701	0	1
4098	GO:0010121	1	0.997978312	0	1
4099	GO:0010124	1	0.995943736	0	2
4100	GO:0010133	1	0.995984286	0	2
4101	GO:0010142	1	0.995990578	0	2
4102	GO:0010155	1	0.995972615	0	2
4103	GO:0010157	1	0.998013437	0	1
4104	GO:0010159	1	0.993997145	0	3
4105	GO:0010164	1	0.997971972	0	1
4106	GO:0010165	1	0.960617718	0	20
4107	GO:0010171	1	0.997983394	0	1
4108	GO:0010172	1	0.988053531	0	6
4109	GO:0010181	1	0.972285397	0	14
4110	GO:0010186	1	0.997988898	0	1
4111	GO:0010189	1	0.997973071	0	1
4112	GO:0010193	1	0.991909365	0	4
4113	GO:0010212	1	0.909861707	0	47
4114	GO:0010216	1	0.986133788	0	7
4115	GO:0010224	1	0.984032912	0	8
4116	GO:0010225	1	0.98014231	0	10
4117	GO:0010226	1	0.980113359	0	10
4118	GO:0010232	1	0.997996294	0	1
4119	GO:0010243	1	0.941349845	0	30
4120	GO:0010248	1	0.980052655	0	10
4121	GO:0010255	1	0.99596914	0	2
4122	GO:0010256	1	0.996030731	0	2
4123	GO:0010257	1	0.995958212	0	2
4124	GO:0010259	1	0.972264446	0	14
4125	GO:0010260	1	0.995980597	0	2
4126	GO:0010265	1	0.988027119	0	6
4127	GO:0010266	1	0.995956661	0	2
4128	GO:0010269	1	0.984002892	0	8
4129	GO:0010272	1	0.997987912	0	1
4130	GO:0010273	1	0.97974702	0	10
4131	GO:0010288	1	0.958619855	0	21
4132	GO:0010309	1	0.997973009	0	1
4133	GO:0010310	1	0.987896186	0	6
4134	GO:0010312	1	0.995974908	0	2
4135	GO:0010314	1	0.96259234	0	19
4136	GO:0010324	1	0.98797137	0	6
4138	GO:0010348	1	0.997990564	0	1
4139	GO:0010359	1	0.995985075	0	2
4140	GO:0010360	1	0.996006084	0	2
4141	GO:0010369	1	0.976202279	0	12
4142	GO:0010370	1	0.995988617	0	2

4143	GO:0010385	1	0.990048711	0	5
4144	GO:0010387	1	0.989961893	0	5
4145	GO:0010389	1	0.849631789	0	81
4146	GO:0010390	1	0.976265342	0	12
4147	GO:0010420	1	0.997965466	0	1
4148	GO:0010424	1	0.99800641	0	1
4149	GO:0010428	1	0.998013437	0	1
4150	GO:0010430	1	0.989997454	0	5
4151	GO:0010436	1	0.99598669	0	2
4152	GO:0010446	1	0.997962035	0	1
4153	GO:0010447	1	0.97810113	0	11
4155	GO:0010453	1	0.997974922	0	1
4156	GO:0010454	1	0.993956702	0	3
4157	GO:0010455	1	0.997976841	0	1
4158	GO:0010457	1	0.976347389	0	12
4159	GO:0010458	1	0.976091167	0	12
4160	GO:0010459	1	0.98602499	0	7
4161	GO:0010460	1	0.96441932	0	18
4162	GO:0010463	1	0.995982072	0	2
4163	GO:0010464	1	0.998013437	0	1
4164	GO:0010465	1	0.998013437	0	1
4166	GO:0010467	1	0.917309944	0	43
4168	GO:0010469	1	0.978186573	0	11
4169	GO:0010470	1	0.982180569	0	9
4170	GO:0010477	1	0.997998115	0	1
4171	GO:0010481	1	0.99801181	0	1
4172	GO:0010482	1	0.993980542	0	3
4173	GO:0010484	1	0.992059802	0	4
4174	GO:0010485	1	0.984094892	0	8
4175	GO:0010494	1	0.860326695	0	75
4176	GO:0010498	1	0.972271334	0	14
4177	GO:0010499	1	0.958168064	0	21
4178	GO:0010501	1	0.98409037	0	8
4180	GO:0010507	1	0.911716445	0	46
4181	GO:0010508	1	0.882852718	0	62
4182	GO:0010509	1	0.998013412	0	1
4183	GO:0010510	1	0.984117131	0	8
4184	GO:0010512	1	0.995987292	0	2
4185	GO:0010513	1	0.99798234	0	1
4186	GO:0010517	1	0.997996934	0	1
4187	GO:0010518	1	0.98407522	0	8
4188	GO:0010519	1	0.997967045	0	1
4189	GO:0010521	1	0.989965154	0	5
4190	GO:0010522	1	0.997993296	0	1
4191	GO:0010523	1	0.99402658	0	3

4192	GO:0010524	1	0.982006497	0	9
4193	GO:0010526	1	0.993995838	0	3
4194	GO:0010529	1	0.964429629	0	18
4195	GO:0010533	1	0.997964448	0	1
4196	GO:0010536	1	0.989953616	0	5
4197	GO:0010543	1	0.98997886	0	5
4198	GO:0010544	1	0.9840373	0	8
4199	GO:0010557	1	0.998006351	0	1
4200	GO:0010559	1	0.998005976	0	1
4201	GO:0010560	1	0.99597126	0	2
4202	GO:0010564	1	0.978192245	0	11
4203	GO:0010565	1	0.992037878	0	4
4204	GO:0010566	1	0.997996671	0	1
4205	GO:0010569	1	0.962489025	0	19
4206	GO:0010571	1	0.988090041	0	6
4207	GO:0010572	1	0.992019479	0	4
4208	GO:0010573	1	0.98600963	0	7
4209	GO:0010574	1	0.995947563	0	2
4210	GO:0010575	1	0.951060226	0	25
4211	GO:0010585	1	0.997977382	0	1
4212	GO:0010586	1	0.968357857	0	16
4213	GO:0010587	1	0.988029599	0	6
4214	GO:0010591	1	0.980028011	0	10
4215	GO:0010592	1	0.949189285	0	26
4216	GO:0010593	1	0.994015808	0	3
4217	GO:0010594	1	0.98213566	0	9
4218	GO:0010595	1	0.882884688	0	62
4219	GO:0010596	1	0.958798072	0	21
4220	GO:0010603	1	0.996021498	0	2
4221	GO:0010606	1	0.98812363	0	6
4222	GO:0010607	1	0.995972488	0	2
4223	GO:0010608	1	0.947374595	0	27
4224	GO:0010609	1	0.993967496	0	3
4225	GO:0010610	1	0.998013437	0	1
4226	GO:0010611	1	0.997974067	0	1
4227	GO:0010613	1	0.96267241	0	19
4228	GO:0010614	1	0.982069467	0	9
4229	GO:0010621	1	0.99393798	0	3
4230	GO:0010623	1	0.997969271	0	1
4231	GO:0010625	1	0.997985882	0	1
4232	GO:0010626	1	0.992032337	0	4
4234	GO:0010629	1	0.61102322	0	244
4235	GO:0010631	1	0.99007241	0	5
4236	GO:0010632	1	0.988118211	0	6
4237	GO:0010633	1	0.970306027	0	15

4239	GO:0010635	1	0.997989952	0	1
4240	GO:0010636	1	0.991996786	0	4
4241	GO:0010637	1	0.982074757	0	9
4242	GO:0010638	1	0.99201117	0	4
4243	GO:0010640	1	0.998007827	0	1
4244	GO:0010641	1	0.995996303	0	2
4245	GO:0010642	1	0.984046822	0	8
4246	GO:0010643	1	0.995995305	0	2
4247	GO:0010644	1	0.986029357	0	7
4248	GO:0010649	1	0.986069279	0	7
4249	GO:0010650	1	0.998013437	0	1
4250	GO:0010652	1	0.995997075	0	2
4251	GO:0010656	1	0.991979276	0	4
4252	GO:0010657	1	0.99799233	0	1
4253	GO:0010659	1	0.987973491	0	6
4254	GO:0010664	1	0.989976153	0	5
4255	GO:0010666	1	0.976237705	0	12
4256	GO:0010667	1	0.96241608	0	19
4257	GO:0010668	1	0.992042731	0	4
4258	GO:0010669	1	0.986047244	0	7
4259	GO:0010693	1	0.994009107	0	3
4260	GO:0010694	1	0.988106676	0	6
4261	GO:0010698	1	0.991948377	0	4
4262	GO:0010700	1	0.992001763	0	4
4263	GO:0010701	1	0.994007378	0	3
4264	GO:0010705	1	0.996006352	0	2
4265	GO:0010706	1	0.990069992	0	5
4266	GO:0010707	1	0.998010924	0	1
4267	GO:0010710	1	0.996000248	0	2
4268	GO:0010711	1	0.997955724	0	1
4269	GO:0010712	1	0.997975423	0	1
4270	GO:0010715	1	0.990063692	0	5
4271	GO:0010716	1	0.993965662	0	3
4272	GO:0010717	1	0.974375866	0	13
4273	GO:0010718	1	0.917272142	0	43
4274	GO:0010719	1	0.943263949	0	29
4275	GO:0010721	1	0.997979199	0	1
4276	GO:0010722	1	0.998013437	0	1
4277	GO:0010724	1	0.995992566	0	2
4278	GO:0010727	1	0.995988391	0	2
4279	GO:0010729	1	0.98991566	0	5
4280	GO:0010730	1	0.992008832	0	4
4281	GO:0010734	1	0.995938944	0	2
4282	GO:0010735	1	0.994019206	0	3
4283	GO:0010736	1	0.998010126	0	1

4284	GO:0010737	1	0.980146631	0	10
4285	GO:0010738	1	0.974292623	0	13
4286	GO:0010739	1	0.978133244	0	11
4288	GO:0010743	1	0.997985513	0	1
4289	GO:0010744	1	0.972162609	0	14
4290	GO:0010745	1	0.976201654	0	12
4291	GO:0010747	1	0.996009457	0	2
4292	GO:0010748	1	0.992055668	0	4
4293	GO:0010749	1	0.998001964	0	1
4294	GO:0010750	1	0.992006411	0	4
4295	GO:0010751	1	0.993987574	0	3
4296	GO:0010752	1	0.996023517	0	2
4297	GO:0010753	1	0.989932246	0	5
4298	GO:0010754	1	0.990071755	0	5
4299	GO:0010755	1	0.998013437	0	1
4300	GO:0010756	1	0.987933171	0	6
4301	GO:0010757	1	0.987978523	0	6
4302	GO:0010758	1	0.99401387	0	3
4303	GO:0010759	1	0.968220142	0	16
4304	GO:0010760	1	0.98990229	0	5
4305	GO:0010761	1	0.976232649	0	12
4306	GO:0010762	1	0.976250966	0	12
4307	GO:0010763	1	0.974302568	0	13
4308	GO:0010764	1	0.984027562	0	8
4309	GO:0010765	1	0.97044216	0	15
4310	GO:0010766	1	0.986106008	0	7
4311	GO:0010767	1	0.997989503	0	1
4312	GO:0010768	1	0.998013437	0	1
4313	GO:0010769	1	0.998013437	0	1
4314	GO:0010780	1	0.998013437	0	1
4315	GO:0010792	1	0.988016144	0	6
4316	GO:0010793	1	0.99003645	0	5
4317	GO:0010795	1	0.995974293	0	2
4318	GO:0010796	1	0.995963996	0	2
4319	GO:0010800	1	0.947245229	0	27
4320	GO:0010801	1	0.960550614	0	20
4321	GO:0010803	1	0.939606223	0	31
4322	GO:0010804	1	0.966219211	0	17
4323	GO:0010807	1	0.986046436	0	7
4324	GO:0010808	1	0.998013437	0	1
4325	GO:0010810	1	0.99598665	0	2
4326	GO:0010811	1	0.939641838	0	31
4327	GO:0010812	1	0.966533957	0	17
4328	GO:0010813	1	0.997969391	0	1
4329	GO:0010814	1	0.998013105	0	1

4330	GO:0010815	1	0.992005569	0	4
4331	GO:0010816	1	0.998013105	0	1
4332	GO:0010817	1	0.992016064	0	4
4335	GO:0010820	1	0.978109963	0	11
4336	GO:0010821	1	0.958602229	0	21
4337	GO:0010822	1	0.990003184	0	5
4338	GO:0010823	1	0.993935607	0	3
4339	GO:0010824	1	0.96057227	0	20
4340	GO:0010825	1	0.992006883	0	4
4341	GO:0010826	1	0.989984275	0	5
4342	GO:0010827	1	0.982134103	0	9
4343	GO:0010828	1	0.990049338	0	5
4344	GO:0010829	1	0.989950084	0	5
4345	GO:0010830	1	0.990026863	0	5
4346	GO:0010831	1	0.984111579	0	8
4347	GO:0010832	1	0.974184937	0	13
4348	GO:0010833	1	0.982037371	0	9
4349	GO:0010835	1	0.990034985	0	5
4350	GO:0010836	1	0.993945242	0	3
4351	GO:0010837	1	0.986043423	0	7
4352	GO:0010838	1	0.978173272	0	11
4353	GO:0010839	1	0.970280969	0	15
4354	GO:0010841	1	0.99801271	0	1
4355	GO:0010842	1	0.958900547	0	21
4358	GO:0010847	1	0.996019312	0	2
4359	GO:0010848	1	0.996012914	0	2
4360	GO:0010849	1	0.997981809	0	1
4361	GO:0010855	1	0.996003745	0	2
4362	GO:0010856	1	0.989973584	0	5
4363	GO:0010857	1	0.997989146	0	1
4364	GO:0010858	1	0.998000642	0	1
4365	GO:0010859	1	0.998010498	0	1
4366	GO:0010862	1	0.926437589	0	38
4367	GO:0010863	1	0.974350462	0	13
4368	GO:0010866	1	0.995976461	0	2
4369	GO:0010867	1	0.974169856	0	13
4370	GO:0010868	1	0.996020721	0	2
4371	GO:0010872	1	0.99196358	0	4
4373	GO:0010874	1	0.986022385	0	7
4374	GO:0010875	1	0.95673959	0	22
4375	GO:0010876	1	0.992022519	0	4
4376	GO:0010877	1	0.997963771	0	1
4377	GO:0010878	1	0.998000348	0	1
4379	GO:0010880	1	0.96635976	0	17
4380	GO:0010881	1	0.96057807	0	20

4381	GO:0010882	1	0.984121305	0	8
4382	GO:0010883	1	0.984110744	0	8
4383	GO:0010884	1	0.978181106	0	11
4384	GO:0010886	1	0.988055145	0	6
4385	GO:0010887	1	0.982067908	0	9
4386	GO:0010888	1	0.982051842	0	9
4387	GO:0010890	1	0.985926717	0	7
4388	GO:0010891	1	0.990035314	0	5
4389	GO:0010894	1	0.992004619	0	4
4390	GO:0010897	1	0.992019258	0	4
4391	GO:0010898	1	0.987927727	0	6
4392	GO:0010899	1	0.996006662	0	2
4393	GO:0010900	1	0.997949301	0	1
4394	GO:0010902	1	0.997954626	0	1
4395	GO:0010903	1	0.997958205	0	1
4396	GO:0010905	1	0.997981631	0	1
4397	GO:0010906	1	0.952948377	0	24
4398	GO:0010907	1	0.980161644	0	10
4399	GO:0010909	1	0.994010367	0	3
4400	GO:0010916	1	0.993887335	0	3
4401	GO:0010917	1	0.972020633	0	14
4402	GO:0010918	1	0.978135427	0	11
4403	GO:0010920	1	0.997992136	0	1
4404	GO:0010921	1	0.995971328	0	2
4405	GO:0010922	1	0.989988767	0	5
4406	GO:0010923	1	0.908273862	0	48
4407	GO:0010925	1	0.997992136	0	1
4408	GO:0010933	1	0.998013437	0	1
4409	GO:0010936	1	0.992011501	0	4
4410	GO:0010939	1	0.997981809	0	1
4411	GO:0010940	1	0.981955806	0	9
4412	GO:0010941	1	0.98595131	0	7
4413	GO:0010942	1	0.909665272	0	47
4414	GO:0010944	1	0.978099132	0	11
4415	GO:0010945	1	0.99592395	0	2
4416	GO:0010948	1	0.997990564	0	1
4417	GO:0010949	1	0.997989301	0	1
4418	GO:0010950	1	0.972039441	0	14
4420	GO:0010952	1	0.972125999	0	14
4421	GO:0010954	1	0.978168278	0	11
4422	GO:0010955	1	0.974122303	0	13
4423	GO:0010956	1	0.993996516	0	3
4424	GO:0010957	1	0.991975403	0	4
4425	GO:0010960	1	0.98810577	0	6
4426	GO:0010961	1	0.994025208	0	3

4427	GO:0010965	1	0.998013437	0	1
4428	GO:0010966	1	0.998013437	0	1
4429	GO:0010968	1	0.997982836	0	1
4430	GO:0010970	1	0.994008055	0	3
4431	GO:0010971	1	0.949076845	0	26
4432	GO:0010972	1	0.892556368	0	56
4433	GO:0010975	1	0.93410717	0	34
4434	GO:0010976	1	0.787050394	0	119
4435	GO:0010977	1	0.873983774	0	67
4436	GO:0010980	1	0.996002509	0	2
4437	GO:0010983	1	0.993965338	0	3
4438	GO:0010988	1	0.995962048	0	2
4439	GO:0010989	1	0.98598988	0	7
4440	GO:0010990	1	0.998007582	0	1
4441	GO:0010991	1	0.992031225	0	4
4442	GO:0010992	1	0.994042357	0	3
4443	GO:0010994	1	0.987961833	0	6
4445	GO:0010997	1	0.984029224	0	8
4446	GO:0010998	1	0.992049464	0	4
4447	GO:0010999	1	0.991963717	0	4
4448	GO:0012501	1	0.950864242	0	25
4449	GO:0012502	1	0.997982564	0	1
4450	GO:0012505	1	0.721117984	0	162
4452	GO:0012507	1	0.893247303	0	56
4453	GO:0012510	1	0.995943619	0	2
4454	GO:0014002	1	0.968306881	0	16
4455	GO:0014003	1	0.954691273	0	23
4456	GO:0014004	1	0.99798512	0	1
4457	GO:0014005	1	0.992013285	0	4
4458	GO:0014008	1	0.997981889	0	1
4459	GO:0014009	1	0.986057122	0	7
4460	GO:0014010	1	0.99599813	0	2
4461	GO:0014012	1	0.987965571	0	6
4462	GO:0014013	1	0.996006046	0	2
4463	GO:0014015	1	0.996012408	0	2
4464	GO:0014016	1	0.997986922	0	1
4465	GO:0014028	1	0.994002757	0	3
4466	GO:0014029	1	0.986061599	0	7
4467	GO:0014031	1	0.990057064	0	5
4468	GO:0014032	1	0.954863058	0	23
4469	GO:0014033	1	0.984108604	0	8
4470	GO:0014034	1	0.998010994	0	1
4471	GO:0014036	1	0.998006804	0	1
4472	GO:0014037	1	0.982153078	0	9
4473	GO:0014038	1	0.997974295	0	1

4474	GO:0014040	1	0.994005238	0	3
4475	GO:0014041	1	0.99597886	0	2
4476	GO:0014042	1	0.990086673	0	5
4477	GO:0014043	1	0.995989121	0	2
4478	GO:0014044	1	0.980156372	0	10
4479	GO:0014045	1	0.995953562	0	2
4480	GO:0014047	1	0.988052782	0	6
4481	GO:0014048	1	0.995989691	0	2
4482	GO:0014049	1	0.990022584	0	5
4483	GO:0014050	1	0.99399166	0	3
4484	GO:0014051	1	0.998013437	0	1
4485	GO:0014052	1	0.998010734	0	1
4486	GO:0014053	1	0.998012706	0	1
4487	GO:0014054	1	0.995993812	0	2
4488	GO:0014056	1	0.997989325	0	1
4489	GO:0014057	1	0.995976879	0	2
4490	GO:0014059	1	0.954831681	0	23
4491	GO:0014061	1	0.995979423	0	2
4492	GO:0014063	1	0.994005702	0	3
4493	GO:0014064	1	0.998011379	0	1
4494	GO:0014065	1	0.928540044	0	37
4495	GO:0014066	1	0.956786017	0	22
4496	GO:0014067	1	0.978177003	0	11
4497	GO:0014068	1	0.848266491	0	82
4500	GO:0014074	1	0.992026036	0	4
4501	GO:0014075	1	0.974242482	0	13
4502	GO:0014701	1	0.982203832	0	9
4503	GO:0014704	1	0.911932866	0	46
4504	GO:0014705	1	0.998009617	0	1
4505	GO:0014706	1	0.991989254	0	4
4506	GO:0014707	1	0.995968234	0	2
4507	GO:0014718	1	0.995969882	0	2
4508	GO:0014719	1	0.994010848	0	3
4509	GO:0014722	1	0.99799233	0	1
4510	GO:0014731	1	0.986115348	0	7
4511	GO:0014732	1	0.991980213	0	4
4512	GO:0014733	1	0.996017057	0	2
4513	GO:0014736	1	0.995985031	0	2
4514	GO:0014737	1	0.998013437	0	1
4515	GO:0014740	1	0.995983339	0	2
4516	GO:0014741	1	0.997991532	0	1
4517	GO:0014801	1	0.998013437	0	1
4518	GO:0014802	1	0.991960356	0	4
4519	GO:0014804	1	0.997977969	0	1
4520	GO:0014805	1	0.997969861	0	1

4521	GO:0014806	1	0.995960849	0	2
4522	GO:0014807	1	0.991999983	0	4
4523	GO:0014808	1	0.989985947	0	5
4524	GO:0014809	1	0.993970202	0	3
4525	GO:0014810	1	0.997955906	0	1
4526	GO:0014816	1	0.992040332	0	4
4527	GO:0014819	1	0.993989452	0	3
4528	GO:0014820	1	0.998013437	0	1
4529	GO:0014823	1	0.913380835	0	45
4530	GO:0014824	1	0.983996459	0	8
4531	GO:0014826	1	0.993966972	0	3
4532	GO:0014827	1	0.991961757	0	4
4533	GO:0014829	1	0.990027598	0	5
4534	GO:0014832	1	0.992009598	0	4
4535	GO:0014834	1	0.990011618	0	5
4536	GO:0014835	1	0.997982996	0	1
4537	GO:0014839	1	0.991948647	0	4
4538	GO:0014841	1	0.996030731	0	2
4539	GO:0014842	1	0.990009395	0	5
4540	GO:0014843	1	0.998013437	0	1
4541	GO:0014846	1	0.996026905	0	2
4542	GO:0014850	1	0.954848981	0	23
4543	GO:0014854	1	0.993956796	0	3
4544	GO:0014857	1	0.997989472	0	1
4545	GO:0014858	1	0.995965762	0	2
4546	GO:0014861	1	0.998013349	0	1
4547	GO:0014866	1	0.997977936	0	1
4548	GO:0014870	1	0.997977969	0	1
4549	GO:0014873	1	0.995982373	0	2
4550	GO:0014876	1	0.997967648	0	1
4551	GO:0014878	1	0.998013437	0	1
4552	GO:0014883	1	0.989992723	0	5
4553	GO:0014886	1	0.99799835	0	1
4554	GO:0014889	1	0.997987997	0	1
4555	GO:0014891	1	0.997990285	0	1
4556	GO:0014894	1	0.984134485	0	8
4557	GO:0014895	1	0.997953993	0	1
4558	GO:0014896	1	0.998013437	0	1
4559	GO:0014898	1	0.97625448	0	12
4560	GO:0014901	1	0.996016888	0	2
4561	GO:0014902	1	0.98209374	0	9
4562	GO:0014904	1	0.992018172	0	4
4563	GO:0014905	1	0.9919625	0	4
4564	GO:0014908	1	0.996016888	0	2
4565	GO:0014909	1	0.986028088	0	7

4566	GO:0014910	1	0.990043481	0	5
4567	GO:0014911	1	0.96257938	0	19
4568	GO:0014912	1	0.972361347	0	14
4569	GO:0014916	1	0.994014555	0	3
4570	GO:0015002	1	0.997990564	0	1
4571	GO:0015012	1	0.968481758	0	16
4572	GO:0015014	1	0.98416289	0	8
4573	GO:0015015	1	0.99005868	0	5
4574	GO:0015016	1	0.992057288	0	4
4575	GO:0015018	1	0.99396088	0	3
4576	GO:0015019	1	0.998013223	0	1
4577	GO:0015020	1	0.948923068	0	26
4578	GO:0015026	1	0.941535823	0	30
4579	GO:0015030	1	0.891416374	0	57
4581	GO:0015035	1	0.962318998	0	19
4582	GO:0015036	1	0.989993096	0	5
4583	GO:0015037	1	0.985941548	0	7
4584	GO:0015038	1	0.995929794	0	2
4585	GO:0015039	1	0.997976892	0	1
4586	GO:0015055	1	0.997975715	0	1
4587	GO:0015057	1	0.99201205	0	4
4588	GO:0015067	1	0.997987997	0	1
4589	GO:0015068	1	0.997987997	0	1
4590	GO:0015074	1	0.984044845	0	8
4591	GO:0015075	1	0.976096266	0	12
4592	GO:0015078	1	0.939465711	0	31
4593	GO:0015079	1	0.992054113	0	4
4594	GO:0015081	1	0.988083868	0	6
4595	GO:0015085	1	0.984178781	0	8
4596	GO:0015086	1	0.992048429	0	4
4597	GO:0015087	1	0.996010884	0	2
4598	GO:0015093	1	0.994032421	0	3
4599	GO:0015094	1	0.998009073	0	1
4600	GO:0015095	1	0.968421454	0	16
4601	GO:0015098	1	0.997976901	0	1
4602	GO:0015099	1	0.998009073	0	1
4603	GO:0015100	1	0.998009073	0	1
4604	GO:0015101	1	0.990003679	0	5
4605	GO:0015105	1	0.997990564	0	1
4606	GO:0015106	1	0.970460542	0	15
4607	GO:0015108	1	0.968505236	0	16
4608	GO:0015111	1	0.994036627	0	3
4609	GO:0015112	1	0.997989007	0	1
4610	GO:0015114	1	0.996030731	0	2
4611	GO:0015116	1	0.972287867	0	14

4612	GO:0015117	1	0.991950994	0	4
4613	GO:0015125	1	0.978140337	0	11
4614	GO:0015126	1	0.998012379	0	1
4615	GO:0015127	1	0.998013026	0	1
4616	GO:0015129	1	0.988037983	0	6
4617	GO:0015130	1	0.99800882	0	1
4618	GO:0015131	1	0.991950994	0	4
4619	GO:0015132	1	0.996024416	0	2
4620	GO:0015136	1	0.99799915	0	1
4621	GO:0015137	1	0.992009936	0	4
4622	GO:0015139	1	0.99600434	0	2
4623	GO:0015140	1	0.991950994	0	4
4624	GO:0015141	1	0.986001323	0	7
4625	GO:0015142	1	0.997997664	0	1
4626	GO:0015143	1	0.994010012	0	3
4627	GO:0015144	1	0.997999237	0	1
4628	GO:0015146	1	0.996030012	0	2
4629	GO:0015150	1	0.996030012	0	2
4630	GO:0015151	1	0.995999837	0	2
4631	GO:0015152	1	0.997980365	0	1
4632	GO:0015164	1	0.998002231	0	1
4633	GO:0015165	1	0.98995265	0	5
4634	GO:0015166	1	0.998013437	0	1
4635	GO:0015168	1	0.996003563	0	2
4636	GO:0015171	1	0.941608655	0	30
4637	GO:0015172	1	0.998003712	0	1
4638	GO:0015173	1	0.993998756	0	3
4639	GO:0015174	1	0.984075387	0	8
4640	GO:0015175	1	0.978195397	0	11
4641	GO:0015179	1	0.974276892	0	13
4642	GO:0015180	1	0.982107992	0	9
4643	GO:0015181	1	0.99402909	0	3
4644	GO:0015182	1	0.993967518	0	3
4645	GO:0015183	1	0.982073879	0	9
4646	GO:0015184	1	0.992001132	0	4
4647	GO:0015185	1	0.992008609	0	4
4648	GO:0015186	1	0.984053486	0	8
4649	GO:0015187	1	0.984065654	0	8
4650	GO:0015188	1	0.998013349	0	1
4651	GO:0015189	1	0.99402909	0	3
4652	GO:0015190	1	0.990015738	0	5
4653	GO:0015191	1	0.997990975	0	1
4654	GO:0015193	1	0.988075187	0	6
4655	GO:0015194	1	0.988022805	0	6
4656	GO:0015195	1	0.99801003	0	1

4657	GO:0015196	1	0.996001462	0	2
4658	GO:0015199	1	0.998013349	0	1
4659	GO:0015204	1	0.989972982	0	5
4660	GO:0015205	1	0.99798441	0	1
4661	GO:0015207	1	0.995981712	0	2
4662	GO:0015211	1	0.993969058	0	3
4663	GO:0015212	1	0.997979307	0	1
4664	GO:0015213	1	0.989958375	0	5
4665	GO:0015214	1	0.997976127	0	1
4666	GO:0015216	1	0.99404106	0	3
4667	GO:0015217	1	0.991961989	0	4
4668	GO:0015218	1	0.995985008	0	2
4669	GO:0015220	1	0.99401264	0	3
4670	GO:0015225	1	0.994023886	0	3
4671	GO:0015226	1	0.993970427	0	3
4672	GO:0015227	1	0.993961751	0	3
4673	GO:0015228	1	0.995983296	0	2
4674	GO:0015229	1	0.99600176	0	2
4675	GO:0015230	1	0.995981627	0	2
4676	GO:0015232	1	0.982078044	0	9
4677	GO:0015233	1	0.99799894	0	1
4678	GO:0015234	1	0.993992799	0	3
4679	GO:0015245	1	0.991976115	0	4
4680	GO:0015247	1	0.992071505	0	4
4681	GO:0015248	1	0.974399862	0	13
4682	GO:0015250	1	0.980018538	0	10
4683	GO:0015252	1	0.993977139	0	3
4684	GO:0015254	1	0.991908827	0	4
4685	GO:0015265	1	0.998008459	0	1
4686	GO:0015267	1	0.960431435	0	20
4687	GO:0015269	1	0.981994718	0	9
4688	GO:0015271	1	0.98807452	0	6
4689	GO:0015272	1	0.992031736	0	4
4690	GO:0015275	1	0.994022233	0	3
4691	GO:0015276	1	0.951166786	0	25
4692	GO:0015277	1	0.994018557	0	3
4694	GO:0015279	1	0.986087786	0	7
4695	GO:0015280	1	0.980142251	0	10
4696	GO:0015288	1	0.989916973	0	5
4697	GO:0015292	1	0.997990564	0	1
4698	GO:0015293	1	0.81135178	0	104
4699	GO:0015295	1	0.992000215	0	4
4700	GO:0015296	1	0.996013832	0	2
4701	GO:0015297	1	0.884716259	0	61
4702	GO:0015299	1	0.966558614	0	17

4703	GO:0015301	1	0.964631137	0	18
4704	GO:0015317	1	0.997973168	0	1
4705	GO:0015319	1	0.997994098	0	1
4706	GO:0015327	1	0.998013437	0	1
4707	GO:0015333	1	0.994012826	0	3
4708	GO:0015347	1	0.976242949	0	12
4709	GO:0015349	1	0.990048461	0	5
4710	GO:0015350	1	0.996009224	0	2
4711	GO:0015361	1	0.997985576	0	1
4712	GO:0015362	1	0.998008156	0	1
4713	GO:0015367	1	0.99797487	0	1
4714	GO:0015368	1	0.998013253	0	1
4715	GO:0015369	1	0.998013391	0	1
4716	GO:0015375	1	0.997997701	0	1
4717	GO:0015377	1	0.984197923	0	8
4718	GO:0015378	1	0.99602848	0	2
4719	GO:0015379	1	0.982230702	0	9
4720	GO:0015382	1	0.996002251	0	2
4721	GO:0015385	1	0.976268159	0	12
4722	GO:0015386	1	0.978234842	0	11
4723	GO:0015389	1	0.993959725	0	3
4724	GO:0015390	1	0.997982243	0	1
4725	GO:0015410	1	0.996005983	0	2
4726	GO:0015417	1	0.99800761	0	1
4727	GO:0015431	1	0.986138878	0	7
4728	GO:0015432	1	0.992060509	0	4
4729	GO:0015433	1	0.992028846	0	4
4730	GO:0015439	1	0.997994159	0	1
4731	GO:0015440	1	0.994015417	0	3
4732	GO:0015450	1	0.995953416	0	2
4733	GO:0015453	1	0.997990564	0	1
4734	GO:0015459	1	0.924546975	0	39
4735	GO:0015464	1	0.974154936	0	13
4736	GO:0015467	1	0.993983593	0	3
4738	GO:0015491	1	0.992044866	0	4
4739	GO:0015495	1	0.997987748	0	1
4740	GO:0015499	1	0.997989046	0	1
4741	GO:0015501	1	0.994033405	0	3
4742	GO:0015552	1	0.997998802	0	1
4743	GO:0015562	1	0.976297444	0	12
4744	GO:0015607	1	0.998003887	0	1
4745	GO:0015616	1	0.990039696	0	5
4748	GO:0015631	1	0.875769327	0	66
4749	GO:0015636	1	0.993974112	0	3
4750	GO:0015643	1	0.97804922	0	11

4751	GO:0015645	1	0.9919591	0	4
4752	GO:0015647	1	0.995974294	0	2
4753	GO:0015651	1	0.991993548	0	4
4754	GO:0015658	1	0.998003396	0	1
4755	GO:0015660	1	0.997989046	0	1
4756	GO:0015670	1	0.989945217	0	5
4757	GO:0015671	1	0.977898874	0	11
4758	GO:0015672	1	0.995955994	0	2
4759	GO:0015675	1	0.998009073	0	1
4760	GO:0015676	1	0.998009073	0	1
4761	GO:0015677	1	0.98812826	0	6
4762	GO:0015680	1	0.997960952	0	1
4763	GO:0015689	1	0.997976901	0	1
4764	GO:0015692	1	0.998009073	0	1
4765	GO:0015693	1	0.97235487	0	14
4766	GO:0015694	1	0.998013026	0	1
4767	GO:0015695	1	0.984025808	0	8
4768	GO:0015696	1	0.985975108	0	7
4769	GO:0015697	1	0.991993548	0	4
4770	GO:0015698	1	0.941709601	0	30
4771	GO:0015700	1	0.997990564	0	1
4772	GO:0015701	1	0.924518463	0	39
4773	GO:0015705	1	0.992061643	0	4
4774	GO:0015706	1	0.997989007	0	1
4775	GO:0015707	1	0.998006135	0	1
4776	GO:0015709	1	0.991950994	0	4
4777	GO:0015711	1	0.964548086	0	18
4778	GO:0015712	1	0.997996904	0	1
4779	GO:0015718	1	0.964476732	0	18
4780	GO:0015721	1	0.941491682	0	30
4781	GO:0015722	1	0.994039248	0	3
4782	GO:0015723	1	0.998013026	0	1
4783	GO:0015724	1	0.997989046	0	1
4784	GO:0015727	1	0.997976841	0	1
4785	GO:0015728	1	0.99800882	0	1
4786	GO:0015729	1	0.991950994	0	4
4787	GO:0015730	1	0.997998802	0	1
4788	GO:0015732	1	0.992064176	0	4
4789	GO:0015734	1	0.988095741	0	6
4790	GO:0015739	1	0.99799915	0	1
4791	GO:0015742	1	0.995986968	0	2
4792	GO:0015744	1	0.997997664	0	1
4793	GO:0015746	1	0.994026356	0	3
4794	GO:0015747	1	0.992009432	0	4
4795	GO:0015750	1	0.996030012	0	2

4796	GO:0015755	1	0.989953219	0	5
4797	GO:0015756	1	0.996030012	0	2
4798	GO:0015757	1	0.996026112	0	2
4799	GO:0015760	1	0.991981552	0	4
4800	GO:0015770	1	0.99199124	0	4
4801	GO:0015774	1	0.998013267	0	1
4802	GO:0015779	1	0.998002231	0	1
4803	GO:0015782	1	0.997975457	0	1
4804	GO:0015786	1	0.995951013	0	2
4805	GO:0015787	1	0.995989633	0	2
4806	GO:0015789	1	0.995989633	0	2
4807	GO:0015790	1	0.998013437	0	1
4808	GO:0015791	1	0.998013437	0	1
4809	GO:0015793	1	0.987943995	0	6
4810	GO:0015797	1	0.997989046	0	1
4811	GO:0015798	1	0.992047301	0	4
4812	GO:0015801	1	0.997992678	0	1
4813	GO:0015802	1	0.997983824	0	1
4814	GO:0015803	1	0.993993475	0	3
4815	GO:0015804	1	0.974292749	0	13
4816	GO:0015805	1	0.997982324	0	1
4817	GO:0015807	1	0.97822517	0	11
4818	GO:0015808	1	0.986062595	0	7
4819	GO:0015809	1	0.992017898	0	4
4820	GO:0015810	1	0.978156753	0	11
4821	GO:0015811	1	0.989993268	0	5
4822	GO:0015812	1	0.996002857	0	2
4823	GO:0015813	1	0.970261644	0	15
4824	GO:0015816	1	0.982142199	0	9
4825	GO:0015817	1	0.995981161	0	2
4826	GO:0015819	1	0.996007903	0	2
4827	GO:0015820	1	0.992053012	0	4
4828	GO:0015821	1	0.997990975	0	1
4829	GO:0015822	1	0.994023086	0	3
4830	GO:0015823	1	0.995998228	0	2
4831	GO:0015824	1	0.986128699	0	7
4832	GO:0015825	1	0.988022805	0	6
4833	GO:0015826	1	0.99801003	0	1
4834	GO:0015827	1	0.991998526	0	4
4835	GO:0015828	1	0.997996731	0	1
4836	GO:0015829	1	0.998003943	0	1
4837	GO:0015833	1	0.98213808	0	9
4838	GO:0015835	1	0.99399551	0	3
4839	GO:0015837	1	0.9959598	0	2
4840	GO:0015838	1	0.998013349	0	1

4841	GO:0015840	1	0.991974338	0	4
4842	GO:0015842	1	0.995974999	0	2
4843	GO:0015844	1	0.986048238	0	7
4844	GO:0015850	1	0.998013429	0	1
4845	GO:0015851	1	0.99798441	0	1
4846	GO:0015853	1	0.991957179	0	4
4847	GO:0015854	1	0.997986954	0	1
4848	GO:0015855	1	0.993959725	0	3
4849	GO:0015858	1	0.991956309	0	4
4850	GO:0015860	1	0.991963195	0	4
4851	GO:0015861	1	0.997979307	0	1
4852	GO:0015862	1	0.989958375	0	5
4853	GO:0015864	1	0.997982243	0	1
4854	GO:0015865	1	0.996022289	0	2
4855	GO:0015866	1	0.989988351	0	5
4856	GO:0015867	1	0.980099499	0	10
4857	GO:0015868	1	0.997972143	0	1
4858	GO:0015871	1	0.990066565	0	5
4859	GO:0015872	1	0.993996277	0	3
4860	GO:0015874	1	0.995979822	0	2
4861	GO:0015876	1	0.997989115	0	1
4862	GO:0015878	1	0.994023886	0	3
4863	GO:0015879	1	0.991962775	0	4
4864	GO:0015881	1	0.994007548	0	3
4865	GO:0015882	1	0.99600176	0	2
4866	GO:0015884	1	0.98599482	0	7
4867	GO:0015886	1	0.984053369	0	8
4868	GO:0015887	1	0.99799894	0	1
4869	GO:0015888	1	0.994012986	0	3
4870	GO:0015889	1	0.989937735	0	5
4871	GO:0015891	1	0.995942297	0	2
4872	GO:0015904	1	0.997976306	0	1
4873	GO:0015908	1	0.97227354	0	14
4874	GO:0015909	1	0.975982784	0	12
4875	GO:0015910	1	0.992020156	0	4
4876	GO:0015911	1	0.991994345	0	4
4877	GO:0015912	1	0.995967422	0	2
4878	GO:0015913	1	0.995986395	0	2
4879	GO:0015914	1	0.917381181	0	43
4880	GO:0015916	1	0.998003887	0	1
4881	GO:0015917	1	0.990099628	0	5
4883	GO:0015919	1	0.998003887	0	1
4884	GO:0015920	1	0.997989487	0	1
4885	GO:0015923	1	0.994046863	0	3
4886	GO:0015926	1	0.997990208	0	1

4887	GO:0015927	1	0.997976297	0	1
4888	GO:0015929	1	0.997990564	0	1
4889	GO:0015931	1	0.995960442	0	2
4890	GO:0015934	1	0.962127438	0	19
4891	GO:0015935	1	0.942487311	0	29
4892	GO:0015936	1	0.990043738	0	5
4893	GO:0015937	1	0.978019834	0	11
4894	GO:0015938	1	0.997960497	0	1
4895	GO:0015939	1	0.985972355	0	7
4896	GO:0015942	1	0.995965152	0	2
4897	GO:0015949	1	0.946914241	0	27
4898	GO:0015961	1	0.997967045	0	1
4899	GO:0015964	1	0.997962006	0	1
4900	GO:0015966	1	0.993997272	0	3
4901	GO:0015980	1	0.995966533	0	2
4902	GO:0015986	1	0.960545168	0	20
4903	GO:0015990	1	0.989957942	0	5
4904	GO:0015995	1	0.997972519	0	1
4905	GO:0016004	1	0.982041314	0	9
4906	GO:0016005	1	0.996004742	0	2
4907	GO:0016010	1	0.970364732	0	15
4908	GO:0016011	1	0.996027162	0	2
4909	GO:0016012	1	0.994008748	0	3
4910	GO:0016013	1	0.991975156	0	4
4911	GO:0016015	1	0.991956953	0	4
4912	GO:0016018	1	0.970079851	0	15
4913	GO:0016019	1	0.991911691	0	4
4916	GO:0016024	1	0.974284955	0	13
4917	GO:0016028	1	0.998003523	0	1
4918	GO:0016031	1	0.995956464	0	2
4920	GO:0016034	1	0.997966837	0	1
4921	GO:0016035	1	0.991974332	0	4
4922	GO:0016036	1	0.996000341	0	2
4923	GO:0016042	1	0.815840299	0	101
4924	GO:0016043	1	0.980271977	0	10
4925	GO:0016045	1	0.980039586	0	10
4926	GO:0016046	1	0.996002777	0	2
4927	GO:0016048	1	0.988068842	0	6
4928	GO:0016050	1	0.972372974	0	14
4929	GO:0016051	1	0.983967054	0	8
4930	GO:0016052	1	0.991955024	0	4
4931	GO:0016055	1	0.64541759	0	217
4932	GO:0016056	1	0.984002249	0	8
4934	GO:0016070	1	0.907567446	0	48
4935	GO:0016071	1	0.988014475	0	6

4936	GO:0016072	1	0.994022803	0	3
4937	GO:0016073	1	0.997990433	0	1
4938	GO:0016074	1	0.997963577	0	1
4939	GO:0016075	1	0.970206697	0	15
4940	GO:0016076	1	0.993972003	0	3
4941	GO:0016077	1	0.998013437	0	1
4942	GO:0016078	1	0.996029327	0	2
4943	GO:0016079	1	0.960687059	0	20
4944	GO:0016080	1	0.992053171	0	4
4945	GO:0016081	1	0.980216486	0	10
4946	GO:0016082	1	0.976289896	0	12
4947	GO:0016094	1	0.997998868	0	1
4948	GO:0016095	1	0.997989115	0	1
4949	GO:0016098	1	0.987937043	0	6
4950	GO:0016101	1	0.993971617	0	3
4951	GO:0016104	1	0.998009713	0	1
4952	GO:0016116	1	0.997992183	0	1
4953	GO:0016119	1	0.997992183	0	1
4954	GO:0016121	1	0.99598669	0	2
4955	GO:0016122	1	0.997992183	0	1
4956	GO:0016125	1	0.956649168	0	22
4959	GO:0016139	1	0.989976534	0	5
4960	GO:0016149	1	0.998004159	0	1
4961	GO:0016150	1	0.997990564	0	1
4962	GO:0016151	1	0.997974365	0	1
4963	GO:0016153	1	0.997999888	0	1
4964	GO:0016154	1	0.997972777	0	1
4965	GO:0016155	1	0.996013877	0	2
4966	GO:0016165	1	0.993974509	0	3
4967	GO:0016167	1	0.995958644	0	2
4968	GO:0016170	1	0.998012951	0	1
4969	GO:0016174	1	0.982150701	0	9
4971	GO:0016176	1	0.983988129	0	8
4972	GO:0016180	1	0.974283349	0	13
4973	GO:0016182	1	0.996023965	0	2
4975	GO:0016188	1	0.978210484	0	11
4976	GO:0016189	1	0.99401721	0	3
4977	GO:0016191	1	0.99204739	0	4
4978	GO:0016192	1	0.567559718	0	280
4979	GO:0016197	1	0.834211686	0	90
4980	GO:0016198	1	0.997997657	0	1
4981	GO:0016199	1	0.990066456	0	5
4982	GO:0016202	1	0.99006058	0	5
4983	GO:0016203	1	0.998013435	0	1
4984	GO:0016206	1	0.997982988	0	1

4985	GO:0016208	1	0.974174734	0	13
4986	GO:0016209	1	0.952525475	0	24
4987	GO:0016212	1	0.991972994	0	4
4988	GO:0016213	1	0.996011391	0	2
4989	GO:0016222	1	0.995984004	0	2
4990	GO:0016226	1	0.956563858	0	22
4991	GO:0016229	1	0.985900548	0	7
4992	GO:0016230	1	0.995974332	0	2
4993	GO:0016231	1	0.997990564	0	1
4994	GO:0016233	1	0.950955659	0	25
4995	GO:0016234	1	0.958640372	0	21
4996	GO:0016235	1	0.937859103	0	32
4997	GO:0016236	1	0.825542435	0	95
4998	GO:0016239	1	0.943401255	0	29
4999	GO:0016240	1	0.986064393	0	7
5000	GO:0016241	1	0.866728547	0	71
5001	GO:0016242	1	0.970454349	0	15
5002	GO:0016243	1	0.995988552	0	2
5003	GO:0016246	1	0.99600655	0	2
5004	GO:0016247	1	0.9899151	0	5
5005	GO:0016248	1	0.998005976	0	1
5006	GO:0016250	1	0.997990804	0	1
5007	GO:0016251	1	0.926047198	0	38
5008	GO:0016254	1	0.968211354	0	16
5009	GO:0016255	1	0.988014766	0	6
5010	GO:0016256	1	0.998013429	0	1
5011	GO:0016259	1	0.993998635	0	3
5012	GO:0016260	1	0.995975033	0	2
5013	GO:0016261	1	0.997986094	0	1
5014	GO:0016262	1	0.993992191	0	3
5015	GO:0016263	1	0.993955386	0	3
5016	GO:0016264	1	0.984064729	0	8
5017	GO:0016266	1	0.900790381	0	52
5018	GO:0016267	1	0.993955386	0	3
5019	GO:0016269	1	0.997986516	0	1
5020	GO:0016272	1	0.985815786	0	7
5021	GO:0016274	1	0.980032228	0	10
5022	GO:0016277	1	0.99798512	0	1
5023	GO:0016278	1	0.997990564	0	1
5024	GO:0016279	1	0.960601246	0	20
5025	GO:0016281	1	0.976178306	0	12
5026	GO:0016282	1	0.96617504	0	17
5027	GO:0016286	1	0.991960636	0	4
5028	GO:0016287	1	0.99798958	0	1
5029	GO:0016290	1	0.972087488	0	14

5030	GO:0016295	1	0.995991857	0	2
5031	GO:0016296	1	0.995991857	0	2
5032	GO:0016297	1	0.995991857	0	2
5033	GO:0016298	1	0.974189151	0	13
5034	GO:0016300	1	0.994001127	0	3
5036	GO:0016303	1	0.98026447	0	10
5037	GO:0016307	1	0.982154973	0	9
5038	GO:0016308	1	0.984111523	0	8
5039	GO:0016309	1	0.994027222	0	3
5041	GO:0016311	1	0.69843948	0	178
5042	GO:0016312	1	0.996014876	0	2
5043	GO:0016314	1	0.991993862	0	4
5044	GO:0016316	1	0.996025715	0	2
5045	GO:0016320	1	0.995982939	0	2
5046	GO:0016321	1	0.993974319	0	3
5047	GO:0016322	1	0.982120539	0	9
5048	GO:0016323	1	0.660426143	0	206
5049	GO:0016324	1	0.516385179	0	327
5050	GO:0016327	1	0.958674678	0	21
5051	GO:0016328	1	0.875820543	0	66
5052	GO:0016331	1	0.978228285	0	11
5053	GO:0016332	1	0.998006669	0	1
5054	GO:0016338	1	0.962303435	0	19
5055	GO:0016339	1	0.924908449	0	39
5056	GO:0016340	1	0.996030729	0	2
5057	GO:0016342	1	0.941773957	0	30
5058	GO:0016344	1	0.996000801	0	2
5059	GO:0016358	1	0.934059072	0	34
5060	GO:0016361	1	0.990043844	0	5
5061	GO:0016362	1	0.998013437	0	1
5062	GO:0016363	1	0.811254177	0	104
5063	GO:0016401	1	0.991983039	0	4
5064	GO:0016402	1	0.997992082	0	1
5065	GO:0016403	1	0.995978269	0	2
5066	GO:0016404	1	0.996005595	0	2
5067	GO:0016405	1	0.995979114	0	2
5068	GO:0016407	1	0.96249835	0	19
5069	GO:0016409	1	0.952848679	0	24
5070	GO:0016410	1	0.970243024	0	15
5071	GO:0016411	1	0.997990076	0	1
5072	GO:0016415	1	0.997959405	0	1
5073	GO:0016418	1	0.998013437	0	1
5074	GO:0016419	1	0.995995107	0	2
5075	GO:0016421	1	0.995977484	0	2
5076	GO:0016422	1	0.993960134	0	3

5077	GO:0016423	1	0.994014508	0	3
5078	GO:0016427	1	0.995972587	0	2
5079	GO:0016428	1	0.991986715	0	4
5080	GO:0016429	1	0.991971961	0	4
5081	GO:0016430	1	0.997990564	0	1
5082	GO:0016432	1	0.993990305	0	3
5083	GO:0016433	1	0.995970495	0	2
5084	GO:0016435	1	0.993956877	0	3
5085	GO:0016437	1	0.997983092	0	1
5086	GO:0016441	1	0.990081563	0	5
5087	GO:0016442	1	0.97238983	0	14
5088	GO:0016444	1	0.998012536	0	1
5089	GO:0016445	1	0.993945569	0	3
5090	GO:0016446	1	0.976188827	0	12
5091	GO:0016447	1	0.990023455	0	5
5092	GO:0016453	1	0.997980886	0	1
5093	GO:0016458	1	0.990044495	0	5
5094	GO:0016459	1	0.913792251	0	45
5095	GO:0016460	1	0.986023048	0	7
5096	GO:0016461	1	0.986053336	0	7
5097	GO:0016462	1	0.98796623	0	6
5098	GO:0016469	1	0.989965707	0	5
5099	GO:0016471	1	0.970117154	0	15
5100	GO:0016476	1	0.994006305	0	3
5102	GO:0016479	1	0.995992204	0	2
5103	GO:0016480	1	0.993932068	0	3
5104	GO:0016482	1	0.996001334	0	2
5105	GO:0016485	1	0.872426303	0	68
5106	GO:0016486	1	0.96829365	0	16
5107	GO:0016488	1	0.995940417	0	2
5109	GO:0016493	1	0.966238702	0	17
5110	GO:0016494	1	0.985959825	0	7
5111	GO:0016495	1	0.997997106	0	1
5112	GO:0016496	1	0.997998203	0	1
5113	GO:0016497	1	0.997985309	0	1
5114	GO:0016500	1	0.99002329	0	5
5115	GO:0016501	1	0.997979973	0	1
5116	GO:0016503	1	0.995936578	0	2
5117	GO:0016504	1	0.980072842	0	10
5118	GO:0016505	1	0.991936163	0	4
5119	GO:0016507	1	0.997995155	0	1
5120	GO:0016508	1	0.99598869	0	2
5121	GO:0016509	1	0.996005735	0	2
5122	GO:0016513	1	0.994031261	0	3
5123	GO:0016514	1	0.964519567	0	18

5124	GO:0016517	1	0.997978378	0	1
5125	GO:0016519	1	0.99797915	0	1
5126	GO:0016520	1	0.998011635	0	1
5127	GO:0016524	1	0.995985075	0	2
5129	GO:0016528	1	0.980120511	0	10
5131	GO:0016530	1	0.99794986	0	1
5132	GO:0016531	1	0.991857562	0	4
5133	GO:0016532	1	0.995976346	0	2
5134	GO:0016533	1	0.995973201	0	2
5135	GO:0016538	1	0.937543409	0	32
5136	GO:0016539	1	0.995954962	0	2
5137	GO:0016540	1	0.952800207	0	24
5138	GO:0016553	1	0.992015702	0	4
5139	GO:0016554	1	0.977990688	0	11
5140	GO:0016556	1	0.995957194	0	2
5141	GO:0016557	1	0.99195731	0	4
5142	GO:0016558	1	0.978125969	0	11
5143	GO:0016559	1	0.980007981	0	10
5144	GO:0016560	1	0.992012097	0	4
5145	GO:0016561	1	0.993988128	0	3
5147	GO:0016569	1	0.996030731	0	2
5148	GO:0016570	1	0.976039268	0	12
5150	GO:0016572	1	0.9742125	0	13
5151	GO:0016573	1	0.904426412	0	50
5152	GO:0016574	1	0.976212387	0	12
5153	GO:0016575	1	0.919161225	0	42
5154	GO:0016576	1	0.988039456	0	6
5155	GO:0016577	1	0.992066822	0	4
5156	GO:0016578	1	0.974211471	0	13
5158	GO:0016580	1	0.974276512	0	13
5159	GO:0016581	1	0.968449234	0	16
5160	GO:0016584	1	0.972288773	0	14
5161	GO:0016586	1	0.996030731	0	2
5162	GO:0016589	1	0.986080968	0	7
5163	GO:0016590	1	0.998013437	0	1
5164	GO:0016591	1	0.984143088	0	8
5165	GO:0016592	1	0.922589428	0	40
5166	GO:0016593	1	0.984095694	0	8
5167	GO:0016594	1	0.97617391	0	12
5168	GO:0016595	1	0.980218738	0	10
5169	GO:0016596	1	0.997990564	0	1
5170	GO:0016597	1	0.962433361	0	19
5171	GO:0016598	1	0.995961908	0	2
5172	GO:0016600	1	0.986083761	0	7
5173	GO:0016601	1	0.958757159	0	21

5174	GO:0016602	1	0.99198122	0	4
5175	GO:0016603	1	0.995958822	0	2
5176	GO:0016604	1	0.523555645	0	320
5177	GO:0016605	1	0.817876243	0	100
5179	GO:0016608	1	0.997954504	0	1
5180	GO:0016614	1	0.991981709	0	4
5181	GO:0016615	1	0.993927927	0	3
5182	GO:0016616	1	0.920501132	0	41
5183	GO:0016618	1	0.997964949	0	1
5184	GO:0016620	1	0.958656146	0	21
5185	GO:0016624	1	0.990040266	0	5
5188	GO:0016638	1	0.998001224	0	1
5189	GO:0016639	1	0.995989342	0	2
5190	GO:0016641	1	0.990036017	0	5
5191	GO:0016651	1	0.976006657	0	12
5192	GO:0016653	1	0.996000126	0	2
5193	GO:0016655	1	0.981852638	0	9
5194	GO:0016661	1	0.993956119	0	3
5195	GO:0016667	1	0.995952524	0	2
5196	GO:0016668	1	0.990001303	0	5
5197	GO:0016670	1	0.996004691	0	2
5198	GO:0016671	1	0.990014981	0	5
5199	GO:0016679	1	0.997988324	0	1
5200	GO:0016684	1	0.989952932	0	5
5201	GO:0016701	1	0.995947955	0	2
5202	GO:0016702	1	0.972236926	0	14
5203	GO:0016705	1	0.863051555	0	73
5204	GO:0016706	1	0.958640834	0	21
5205	GO:0016709	1	0.968403099	0	16
5206	GO:0016711	1	0.997988324	0	1
5207	GO:0016712	1	0.946946868	0	27
5208	GO:0016714	1	0.995964069	0	2
5209	GO:0016715	1	0.9960036	0	2
5211	GO:0016722	1	0.996003158	0	2
5212	GO:0016723	1	0.997998773	0	1
5214	GO:0016742	1	0.992024854	0	4
5215	GO:0016743	1	0.998013437	0	1
5216	GO:0016746	1	0.731357163	0	155
5217	GO:0016747	1	0.964203134	0	18
5218	GO:0016757	1	0.650425059	0	213
5219	GO:0016758	1	0.952780909	0	24
5220	GO:0016763	1	0.983970531	0	8
5221	GO:0016765	1	0.988020952	0	6
5222	GO:0016768	1	0.997973345	0	1
5223	GO:0016772	1	0.974141234	0	13

5224	GO:0016773	1	0.966437522	0	17
5225	GO:0016776	1	0.993974502	0	3
5227	GO:0016780	1	0.989965708	0	5
5228	GO:0016783	1	0.991934466	0	4
5229	GO:0016784	1	0.997969391	0	1
5230	GO:0016785	1	0.998013431	0	1
5232	GO:0016788	1	0.947000169	0	27
5233	GO:0016790	1	0.976062974	0	12
5234	GO:0016791	1	0.757378874	0	138
5235	GO:0016793	1	0.997997404	0	1
5236	GO:0016798	1	0.859713616	0	75
5237	GO:0016799	1	0.986021898	0	7
5238	GO:0016805	1	0.980092448	0	10
5239	GO:0016807	1	0.995985075	0	2
5240	GO:0016810	1	0.974267746	0	13
5241	GO:0016811	1	0.976044611	0	12
5242	GO:0016812	1	0.994008427	0	3
5243	GO:0016813	1	0.995964116	0	2
5244	GO:0016814	1	0.997990789	0	1
5245	GO:0016817	1	0.997986375	0	1
5246	GO:0016818	1	0.976226917	0	12
5248	GO:0016830	1	0.997979249	0	1
5249	GO:0016831	1	0.960484095	0	20
5250	GO:0016833	1	0.997969926	0	1
5251	GO:0016836	1	0.964257849	0	18
5252	GO:0016841	1	0.997996731	0	1
5253	GO:0016844	1	0.997990564	0	1
5254	GO:0016846	1	0.995941763	0	2
5255	GO:0016849	1	0.966538398	0	17
5256	GO:0016853	1	0.771718125	0	128
5257	GO:0016854	1	0.997975776	0	1
5258	GO:0016857	1	0.993998759	0	3
5259	GO:0016859	1	0.9979602	0	1
5260	GO:0016860	1	0.998006674	0	1
5261	GO:0016863	1	0.993999571	0	3
5262	GO:0016866	1	0.996004186	0	2
5263	GO:0016868	1	0.981999986	0	9
5264	GO:0016874	1	0.76343867	0	134
5265	GO:0016878	1	0.995989843	0	2
5266	GO:0016879	1	0.996030731	0	2
5267	GO:0016881	1	0.99798654	0	1
5268	GO:0016884	1	0.993959391	0	3
5269	GO:0016886	1	0.997990564	0	1
5271	GO:0016888	1	0.993976237	0	3
5272	GO:0016890	1	0.99598445	0	2

5273	GO:0016891	1	0.992000498	0	4
5274	GO:0016892	1	0.997990564	0	1
5275	GO:0016907	1	0.989987231	0	5
5276	GO:0016917	1	0.998013437	0	1
5277	GO:0016918	1	0.985879269	0	7
5278	GO:0016920	1	0.998013437	0	1
5279	GO:0016922	1	0.95494425	0	23
5280	GO:0016925	1	0.886354777	0	60
5281	GO:0016926	1	0.982163023	0	9
5282	GO:0016933	1	0.995974469	0	2
5283	GO:0016934	1	0.991996471	0	4
5284	GO:0016935	1	0.996002247	0	2
5285	GO:0016936	1	0.983830607	0	8
5286	GO:0016937	1	0.998013437	0	1
5287	GO:0016938	1	0.997993643	0	1
5288	GO:0016939	1	0.994033039	0	3
5289	GO:0016941	1	0.994007712	0	3
5290	GO:0016942	1	0.994026967	0	3
5291	GO:0016964	1	0.998013437	0	1
5292	GO:0016971	1	0.994001921	0	3
5293	GO:0016972	1	0.988039576	0	6
5294	GO:0016973	1	0.956670252	0	22
5295	GO:0016992	1	0.997973035	0	1
5296	GO:0016997	1	0.995974161	0	2
5297	GO:0016998	1	0.997960101	0	1
5298	GO:0016999	1	0.998013026	0	1
5299	GO:0017002	1	0.992036906	0	4
5300	GO:0017004	1	0.995952378	0	2
5301	GO:0017005	1	0.998003586	0	1
5302	GO:0017015	1	0.956897835	0	22
5303	GO:0017017	1	0.976114706	0	12
5304	GO:0017018	1	0.998012626	0	1
5305	GO:0017020	1	0.994000654	0	3
5306	GO:0017022	1	0.953046531	0	24
5307	GO:0017024	1	0.997958165	0	1
5308	GO:0017025	1	0.948888567	0	26
5309	GO:0017038	1	0.991985508	0	4
5310	GO:0017040	1	0.985948484	0	7
5311	GO:0017041	1	0.997968378	0	1
5312	GO:0017046	1	0.951027931	0	25
5313	GO:0017050	1	0.993975404	0	3
5314	GO:0017053	1	0.900919537	0	52
5315	GO:0017054	1	0.995961854	0	2
5316	GO:0017055	1	0.995989958	0	2
5317	GO:0017056	1	0.949175901	0	26

5318	GO:0017057	1	0.995978309	0	2
5319	GO:0017059	1	0.990016376	0	5
5320	GO:0017060	1	0.997982083	0	1
5321	GO:0017061	1	0.996007182	0	2
5322	GO:0017064	1	0.993955079	0	3
5323	GO:0017065	1	0.997971123	0	1
5324	GO:0017069	1	0.974138083	0	13
5325	GO:0017070	1	0.976052053	0	12
5326	GO:0017071	1	0.998003731	0	1
5327	GO:0017075	1	0.954970644	0	23
5328	GO:0017076	1	0.987984133	0	6
5329	GO:0017077	1	0.991958109	0	4
5330	GO:0017080	1	0.943251289	0	29
5331	GO:0017081	1	0.987991541	0	6
5332	GO:0017083	1	0.992027809	0	4
5333	GO:0017085	1	0.992030803	0	4
5334	GO:0017087	1	0.998006825	0	1
5335	GO:0017089	1	0.997978303	0	1
5336	GO:0017090	1	0.99799342	0	1
5337	GO:0017095	1	0.994043296	0	3
5338	GO:0017096	1	0.997966837	0	1
5339	GO:0017098	1	0.997987328	0	1
5340	GO:0017099	1	0.991960971	0	4
5341	GO:0017101	1	0.978051172	0	11
5342	GO:0017108	1	0.982097825	0	9
5343	GO:0017109	1	0.996004042	0	2
5344	GO:0017110	1	0.97611677	0	12
5345	GO:0017111	1	0.980151736	0	10
5346	GO:0017113	1	0.997996294	0	1
5347	GO:0017116	1	0.970255961	0	15
5348	GO:0017118	1	0.997968248	0	1
5349	GO:0017119	1	0.976160396	0	12
5350	GO:0017121	1	0.986025752	0	7
5351	GO:0017122	1	0.998013396	0	1
5352	GO:0017124	1	0.77613727	0	126
5353	GO:0017125	1	0.998012223	0	1
5354	GO:0017126	1	0.995978545	0	2
5355	GO:0017128	1	0.98207691	0	9
5356	GO:0017129	1	0.993905766	0	3
5357	GO:0017130	1	0.998013437	0	1
5358	GO:0017134	1	0.954868754	0	23
5359	GO:0017136	1	0.991971508	0	4
5360	GO:0017143	1	0.995981555	0	2
5361	GO:0017144	1	0.947005111	0	27
5362	GO:0017145	1	0.986102153	0	7

5363	GO:0017146	1	0.98222806	0	9
5364	GO:0017147	1	0.945347836	0	28
5365	GO:0017148	1	0.851355117	0	80
5366	GO:0017150	1	0.989954366	0	5
5367	GO:0017151	1	0.986056034	0	7
5368	GO:0017153	1	0.996009718	0	2
5369	GO:0017154	1	0.976374575	0	12
5370	GO:0017156	1	0.945354528	0	28
5371	GO:0017157	1	0.935784454	0	33
5372	GO:0017158	1	0.968382895	0	16
5373	GO:0017159	1	0.993970209	0	3
5374	GO:0017161	1	0.996025715	0	2
5375	GO:0017162	1	0.982124158	0	9
5376	GO:0017166	1	0.976328126	0	12
5377	GO:0017168	1	0.998007874	0	1
5378	GO:0017171	1	0.989964611	0	5
5379	GO:0017174	1	0.997961622	0	1
5380	GO:0017176	1	0.989927926	0	5
5381	GO:0017177	1	0.995993502	0	2
5382	GO:0017178	1	0.997990564	0	1
5383	GO:0017183	1	0.986004184	0	7
5384	GO:0017185	1	0.984059941	0	8
5385	GO:0017186	1	0.995958822	0	2
5386	GO:0017187	1	0.993931175	0	3
5387	GO:0017188	1	0.998013437	0	1
5388	GO:0017190	1	0.997990564	0	1
5389	GO:0017196	1	0.986037476	0	7
5390	GO:0017198	1	0.997957984	0	1
5391	GO:0018002	1	0.995952559	0	2
5392	GO:0018008	1	0.9960294	0	2
5393	GO:0018009	1	0.997980838	0	1
5394	GO:0018011	1	0.997990564	0	1
5395	GO:0018012	1	0.997990564	0	1
5396	GO:0018013	1	0.997990564	0	1
5397	GO:0018016	1	0.997990564	0	1
5398	GO:0018021	1	0.9979813	0	1
5399	GO:0018022	1	0.976170855	0	12
5400	GO:0018023	1	0.982058985	0	9
5402	GO:0018025	1	0.997990564	0	1
5403	GO:0018026	1	0.98010918	0	10
5404	GO:0018027	1	0.98604466	0	7
5405	GO:0018032	1	0.998004627	0	1
5406	GO:0018057	1	0.990036017	0	5
5407	GO:0018063	1	0.997983283	0	1
5408	GO:0018064	1	0.995975829	0	2

5409	GO:0018076	1	0.994050929	0	3
5410	GO:0018094	1	0.993984576	0	3
5411	GO:0018095	1	0.974210161	0	13
5412	GO:0018101	1	0.992020464	0	4
5413	GO:0018105	1	0.726418462	0	159
5414	GO:0018106	1	0.99795485	0	1
5415	GO:0018107	1	0.879586794	0	64
5416	GO:0018108	1	0.762622491	0	135
5417	GO:0018114	1	0.997986313	0	1
5418	GO:0018117	1	0.995967109	0	2
5419	GO:0018119	1	0.989951329	0	5
5420	GO:0018120	1	0.995942121	0	2
5421	GO:0018125	1	0.995980869	0	2
5422	GO:0018126	1	0.989971137	0	5
5423	GO:0018142	1	0.997990564	0	1
5424	GO:0018146	1	0.951013542	0	25
5425	GO:0018149	1	0.950807821	0	25
5426	GO:0018153	1	0.995977619	0	2
5427	GO:0018158	1	0.991930147	0	4
5428	GO:0018160	1	0.997969289	0	1
5429	GO:0018166	1	0.998013147	0	1
5430	GO:0018171	1	0.993965354	0	3
5431	GO:0018193	1	0.992002861	0	4
5432	GO:0018200	1	0.998012903	0	1
5433	GO:0018205	1	0.998013437	0	1
5434	GO:0018206	1	0.98994455	0	5
5435	GO:0018208	1	0.995989409	0	2
5437	GO:0018216	1	0.982009	0	9
5438	GO:0018230	1	0.954757013	0	23
5439	GO:0018242	1	0.980188207	0	10
5440	GO:0018243	1	0.984123917	0	8
5441	GO:0018271	1	0.998013437	0	1
5442	GO:0018272	1	0.997974251	0	1
5443	GO:0018276	1	0.997965944	0	1
5444	GO:0018277	1	0.995981329	0	2
5445	GO:0018279	1	0.941193533	0	30
5446	GO:0018283	1	0.996002075	0	2
5447	GO:0018293	1	0.997964313	0	1
5448	GO:0018298	1	0.987996036	0	6
5449	GO:0018312	1	0.993986509	0	3
5450	GO:0018315	1	0.99800978	0	1
5451	GO:0018342	1	0.985969408	0	7
5452	GO:0018343	1	0.991968851	0	4
5453	GO:0018344	1	0.987993187	0	6
5454	GO:0018345	1	0.956766778	0	22

5455	GO:0018350	1	0.997984899	0	1
5456	GO:0018352	1	0.997984584	0	1
5457	GO:0018364	1	0.995968151	0	2
5458	GO:0018393	1	0.986085328	0	7
5459	GO:0018394	1	0.988035625	0	6
5460	GO:0018395	1	0.998003307	0	1
5461	GO:0018400	1	0.998013437	0	1
5462	GO:0018401	1	0.980038243	0	10
5463	GO:0018406	1	0.990035775	0	5
5464	GO:0018423	1	0.997965973	0	1
5465	GO:0018424	1	0.998007582	0	1
5466	GO:0018444	1	0.994021183	0	3
5467	GO:0018455	1	0.987915201	0	6
5468	GO:0018467	1	0.997988883	0	1
5469	GO:0018477	1	0.997991563	0	1
5470	GO:0018478	1	0.997981631	0	1
5471	GO:0018479	1	0.993958696	0	3
5472	GO:0018549	1	0.997973663	0	1
5473	GO:0018601	1	0.997972955	0	1
5474	GO:0018636	1	0.993914222	0	3
5475	GO:0018675	1	0.995949749	0	2
5476	GO:0018676	1	0.995949749	0	2
5477	GO:0018685	1	0.989936086	0	5
5478	GO:0018733	1	0.997964061	0	1
5479	GO:0018738	1	0.997964119	0	1
5480	GO:0018773	1	0.997973115	0	1
5481	GO:0018812	1	0.991985904	0	4
5482	GO:0018858	1	0.993970963	0	3
5483	GO:0018872	1	0.996002523	0	2
5484	GO:0018874	1	0.997979579	0	1
5485	GO:0018879	1	0.995974513	0	2
5486	GO:0018885	1	0.997972955	0	1
5487	GO:0018894	1	0.993985764	0	3
5488	GO:0018910	1	0.997972955	0	1
5489	GO:0018916	1	0.991929692	0	4
5490	GO:0018931	1	0.99594783	0	2
5491	GO:0018958	1	0.993931595	0	3
5492	GO:0018960	1	0.997972955	0	1
5493	GO:0018963	1	0.997989278	0	1
5494	GO:0018964	1	0.99800985	0	1
5495	GO:0018969	1	0.997992786	0	1
5496	GO:0018979	1	0.99797575	0	1
5497	GO:0019001	1	0.968314754	0	16
5498	GO:0019002	1	0.994005266	0	3
5500	GO:0019005	1	0.884359597	0	61

5501	GO:0019008	1	0.995981408	0	2
5503	GO:0019046	1	0.994014726	0	3
5504	GO:0019048	1	0.984082755	0	8
5505	GO:0019050	1	0.995952161	0	2
5506	GO:0019054	1	0.978143777	0	11
5507	GO:0019056	1	0.99799243	0	1
5508	GO:0019058	1	0.829108652	0	93
5510	GO:0019061	1	0.997983076	0	1
5511	GO:0019062	1	0.98999307	0	5
5512	GO:0019064	1	0.985986801	0	7
5513	GO:0019065	1	0.988074439	0	6
5514	GO:0019068	1	0.976061117	0	12
5515	GO:0019074	1	0.996025067	0	2
5516	GO:0019075	1	0.995985075	0	2
5517	GO:0019076	1	0.987972867	0	6
5518	GO:0019079	1	0.972245262	0	14
5519	GO:0019081	1	0.989946383	0	5
5520	GO:0019082	1	0.924500144	0	39
5521	GO:0019083	1	0.797490863	0	111
5522	GO:0019085	1	0.996014321	0	2
5523	GO:0019086	1	0.991981008	0	4
5524	GO:0019087	1	0.993991703	0	3
5525	GO:0019100	1	0.996024048	0	2
5526	GO:0019102	1	0.997995025	0	1
5527	GO:0019103	1	0.995976439	0	2
5528	GO:0019104	1	0.985934388	0	7
5529	GO:0019107	1	0.998012634	0	1
5530	GO:0019115	1	0.997978287	0	1
5531	GO:0019119	1	0.997967328	0	1
5532	GO:0019135	1	0.997976697	0	1
5533	GO:0019136	1	0.993973611	0	3
5534	GO:0019144	1	0.995938883	0	2
5535	GO:0019145	1	0.997986719	0	1
5536	GO:0019150	1	0.997979587	0	1
5537	GO:0019153	1	0.997971648	0	1
5538	GO:0019158	1	0.992034495	0	4
5539	GO:0019166	1	0.993944622	0	3
5540	GO:0019171	1	0.996007903	0	2
5541	GO:0019178	1	0.997990564	0	1
5542	GO:0019185	1	0.994008642	0	3
5543	GO:0019200	1	0.997990564	0	1
5544	GO:0019202	1	0.997990564	0	1
5545	GO:0019203	1	0.997987826	0	1
5546	GO:0019205	1	0.982052452	0	9
5547	GO:0019206	1	0.995949871	0	2

5548	GO:0019207	1	0.991956151	0	4
5549	GO:0019208	1	0.984179929	0	8
5550	GO:0019209	1	0.990017564	0	5
5551	GO:0019210	1	0.989957855	0	5
5552	GO:0019211	1	0.992011668	0	4
5553	GO:0019212	1	0.989968571	0	5
5554	GO:0019213	1	0.972241227	0	14
5555	GO:0019215	1	0.980146145	0	10
5557	GO:0019217	1	0.98406933	0	8
5558	GO:0019218	1	0.994033368	0	3
5560	GO:0019222	1	0.902430483	0	51
5561	GO:0019226	1	0.972256363	0	14
5562	GO:0019227	1	0.986041896	0	7
5563	GO:0019228	1	0.955003088	0	23
5564	GO:0019229	1	0.964459773	0	18
5565	GO:0019230	1	0.994015353	0	3
5566	GO:0019233	1	0.92644505	0	38
5567	GO:0019236	1	0.991938295	0	4
5568	GO:0019237	1	0.988036726	0	6
5569	GO:0019239	1	0.984062753	0	8
5570	GO:0019240	1	0.99403979	0	3
5571	GO:0019242	1	0.997969123	0	1
5572	GO:0019243	1	0.993947628	0	3
5573	GO:0019244	1	0.997965332	0	1
5574	GO:0019249	1	0.995976346	0	2
5575	GO:0019254	1	0.993978425	0	3
5576	GO:0019255	1	0.995963559	0	2
5577	GO:0019262	1	0.987908074	0	6
5578	GO:0019264	1	0.995972487	0	2
5579	GO:0019265	1	0.997971738	0	1
5580	GO:0019276	1	0.993986937	0	3
5581	GO:0019284	1	0.993952141	0	3
5582	GO:0019285	1	0.996010025	0	2
5583	GO:0019287	1	0.993933141	0	3
5584	GO:0019290	1	0.997993203	0	1
5585	GO:0019303	1	0.995932579	0	2
5586	GO:0019310	1	0.997968788	0	1
5587	GO:0019318	1	0.990036547	0	5
5588	GO:0019319	1	0.998011974	0	1
5589	GO:0019321	1	0.997979587	0	1
5590	GO:0019322	1	0.995965769	0	2
5591	GO:0019323	1	0.997983458	0	1
5592	GO:0019341	1	0.997988324	0	1
5593	GO:0019343	1	0.993964935	0	3
5594	GO:0019344	1	0.991961336	0	4

5595	GO:0019346	1	0.98794608	0	6
5596	GO:0019348	1	0.991926075	0	4
5597	GO:0019363	1	0.980083337	0	10
5598	GO:0019367	1	0.987988928	0	6
5599	GO:0019369	1	0.928095788	0	37
5600	GO:0019370	1	0.981924754	0	9
5601	GO:0019371	1	0.980021093	0	10
5602	GO:0019372	1	0.968142733	0	16
5603	GO:0019373	1	0.962347329	0	19
5604	GO:0019377	1	0.995989104	0	2
5605	GO:0019388	1	0.987963822	0	6
5606	GO:0019391	1	0.995976321	0	2
5607	GO:0019395	1	0.968369146	0	16
5608	GO:0019402	1	0.997967309	0	1
5609	GO:0019408	1	0.996005421	0	2
5610	GO:0019413	1	0.996009229	0	2
5611	GO:0019417	1	0.996012841	0	2
5612	GO:0019427	1	0.996009229	0	2
5613	GO:0019430	1	0.977941892	0	11
5614	GO:0019432	1	0.966488456	0	17
5615	GO:0019433	1	0.964354876	0	18
5616	GO:0019438	1	0.997966837	0	1
5617	GO:0019439	1	0.989907247	0	5
5618	GO:0019440	1	0.997979521	0	1
5619	GO:0019441	1	0.989911178	0	5
5620	GO:0019442	1	0.995947103	0	2
5621	GO:0019448	1	0.995963941	0	2
5622	GO:0019449	1	0.997989828	0	1
5623	GO:0019452	1	0.997989828	0	1
5624	GO:0019464	1	0.993967408	0	3
5625	GO:0019470	1	0.991980455	0	4
5626	GO:0019477	1	0.998013437	0	1
5627	GO:0019478	1	0.997972026	0	1
5628	GO:0019482	1	0.995987241	0	2
5629	GO:0019483	1	0.995980026	0	2
5630	GO:0019509	1	0.989965507	0	5
5631	GO:0019510	1	0.997983347	0	1
5632	GO:0019511	1	0.986006993	0	7
5633	GO:0019516	1	0.997965332	0	1
5634	GO:0019518	1	0.991925759	0	4
5635	GO:0019521	1	0.997977668	0	1
5636	GO:0019530	1	0.9900521	0	5
5637	GO:0019531	1	0.982153123	0	9
5638	GO:0019532	1	0.982153123	0	9
5639	GO:0019534	1	0.988052368	0	6

5640	GO:0019538	1	0.984041143	0	8
5641	GO:0019542	1	0.996009229	0	2
5642	GO:0019543	1	0.997978061	0	1
5643	GO:0019544	1	0.997978312	0	1
5644	GO:0019547	1	0.997978287	0	1
5645	GO:0019550	1	0.995970589	0	2
5646	GO:0019551	1	0.995970589	0	2
5647	GO:0019556	1	0.991978108	0	4
5648	GO:0019557	1	0.991978108	0	4
5649	GO:0019563	1	0.990029329	0	5
5650	GO:0019605	1	0.997979579	0	1
5651	GO:0019606	1	0.997972849	0	1
5652	GO:0019626	1	0.989963054	0	5
5653	GO:0019627	1	0.997976314	0	1
5654	GO:0019637	1	0.997992461	0	1
5655	GO:0019640	1	0.989927379	0	5
5656	GO:0019646	1	0.997990564	0	1
5657	GO:0019673	1	0.991909098	0	4
5658	GO:0019674	1	0.970254372	0	15
5659	GO:0019676	1	0.998010419	0	1
5660	GO:0019677	1	0.993988898	0	3
5661	GO:0019682	1	0.995938225	0	2
5662	GO:0019693	1	0.991926624	0	4
5663	GO:0019695	1	0.994004143	0	3
5664	GO:0019705	1	0.988072179	0	6
5665	GO:0019706	1	0.947106722	0	27
5666	GO:0019722	1	0.866876217	0	71
5667	GO:0019724	1	0.987939654	0	6
5668	GO:0019725	1	0.982030858	0	9
5669	GO:0019730	1	0.92751888	0	37
5670	GO:0019731	1	0.944699665	0	28
5671	GO:0019732	1	0.991947394	0	4
5672	GO:0019742	1	0.997985159	0	1
5673	GO:0019747	1	0.997958205	0	1
5674	GO:0019752	1	0.956406905	0	22
5675	GO:0019763	1	0.997971945	0	1
5676	GO:0019766	1	0.997971104	0	1
5677	GO:0019767	1	0.997952005	0	1
5678	GO:0019772	1	0.997971783	0	1
5679	GO:0019773	1	0.985821611	0	7
5680	GO:0019774	1	0.977799278	0	11
5681	GO:0019776	1	0.997965121	0	1
5682	GO:0019777	1	0.995953508	0	2
5683	GO:0019778	1	0.998012836	0	1
5684	GO:0019779	1	0.998012836	0	1

5685	GO:0019780	1	0.998013437	0	1
5686	GO:0019781	1	0.995960083	0	2
5687	GO:0019782	1	0.997999323	0	1
5688	GO:0019783	1	0.994005912	0	3
5689	GO:0019784	1	0.989992181	0	5
5690	GO:0019787	1	0.987979383	0	6
5691	GO:0019788	1	0.995941417	0	2
5692	GO:0019789	1	0.966334253	0	17
5693	GO:0019797	1	0.993983524	0	3
5694	GO:0019799	1	0.997990564	0	1
5695	GO:0019800	1	0.988001814	0	6
5696	GO:0019805	1	0.993960353	0	3
5697	GO:0019806	1	0.998013437	0	1
5698	GO:0019807	1	0.997967723	0	1
5699	GO:0019809	1	0.99592624	0	2
5700	GO:0019810	1	0.997998357	0	1
5701	GO:0019811	1	0.998011379	0	1
5702	GO:0019814	1	0.991985904	0	4
5703	GO:0019815	1	0.993953275	0	3
5704	GO:0019825	1	0.946732808	0	27
5705	GO:0019826	1	0.989982529	0	5
5706	GO:0019827	1	0.902546653	0	51
5707	GO:0019828	1	0.986000994	0	7
5708	GO:0019829	1	0.964743786	0	18
5709	GO:0019834	1	0.989877188	0	5
5711	GO:0019838	1	0.910045978	0	47
5712	GO:0019841	1	0.97990696	0	10
5713	GO:0019842	1	0.992000383	0	4
5714	GO:0019843	1	0.910889167	0	46
5715	GO:0019852	1	0.982031731	0	9
5716	GO:0019853	1	0.995950795	0	2
5717	GO:0019855	1	0.97997489	0	10
5718	GO:0019856	1	0.993998455	0	3
5719	GO:0019858	1	0.995967752	0	2
5720	GO:0019859	1	0.997981631	0	1
5721	GO:0019860	1	0.995956107	0	2
5722	GO:0019862	1	0.997971104	0	1
5723	GO:0019863	1	0.993917507	0	3
5724	GO:0019864	1	0.989875845	0	5
5725	GO:0019865	1	0.995995182	0	2
5726	GO:0019866	1	0.997984252	0	1
5727	GO:0019867	1	0.994013197	0	3
5728	GO:0019869	1	0.980110675	0	10
5729	GO:0019870	1	0.984145001	0	8
5730	GO:0019871	1	0.984058637	0	8

5731	GO:0019878	1	0.99600958	0	2
5732	GO:0019882	1	0.918545883	0	42
5733	GO:0019883	1	0.995968412	0	2
5734	GO:0019884	1	0.995931476	0	2
5735	GO:0019885	1	0.982116627	0	9
5736	GO:0019886	1	0.827305424	0	94
5737	GO:0019887	1	0.97606422	0	12
5738	GO:0019888	1	0.926425697	0	38
5739	GO:0019894	1	0.917447907	0	43
5740	GO:0019896	1	0.980181723	0	10
5741	GO:0019897	1	0.95671252	0	22
5742	GO:0019898	1	0.889814365	0	58
5744	GO:0019900	1	0.817858733	0	100
5745	GO:0019901	1	0.390955433	0	463
5746	GO:0019902	1	0.908254785	0	48
5747	GO:0019903	1	0.83796327	0	88
5748	GO:0019904	1	0.600054867	0	253
5749	GO:0019905	1	0.891759067	0	57
5750	GO:0019907	1	0.993917522	0	3
5751	GO:0019908	1	0.994007473	0	3
5752	GO:0019911	1	0.981984947	0	9
5753	GO:0019912	1	0.997981914	0	1
5754	GO:0019915	1	0.952762263	0	24
5755	GO:0019918	1	0.991975634	0	4
5756	GO:0019919	1	0.989956858	0	5
5757	GO:0019932	1	0.991993556	0	4
5758	GO:0019933	1	0.949327519	0	26
5759	GO:0019934	1	0.970282056	0	15
5760	GO:0019935	1	0.998012414	0	1
5762	GO:0019948	1	0.995979383	0	2
5763	GO:0019953	1	0.984030576	0	8
5765	GO:0019956	1	0.987979278	0	6
5766	GO:0019957	1	0.966232656	0	17
5767	GO:0019958	1	0.993977269	0	3
5768	GO:0019959	1	0.996006612	0	2
5769	GO:0019960	1	0.99008039	0	5
5770	GO:0019962	1	0.995985648	0	2
5771	GO:0019964	1	0.998001265	0	1
5772	GO:0019966	1	0.988006964	0	6
5773	GO:0019969	1	0.99800453	0	1
5774	GO:0019970	1	0.993991629	0	3
5775	GO:0019976	1	0.993988051	0	3
5776	GO:0019981	1	0.996010011	0	2
5778	GO:0019992	1	0.974231685	0	13
5779	GO:0020003	1	0.997990556	0	1

5780	GO:0020005	1	0.997990556	0	1
5781	GO:0020018	1	0.994029085	0	3
5782	GO:0020027	1	0.993975322	0	3
5783	GO:0020037	1	0.76902251	0	130
5784	GO:0021501	1	0.997980022	0	1
5785	GO:0021502	1	0.996019363	0	2
5786	GO:0021503	1	0.998013437	0	1
5787	GO:0021508	1	0.998013437	0	1
5788	GO:0021509	1	0.997978453	0	1
5789	GO:0021510	1	0.941561905	0	30
5790	GO:0021511	1	0.998013437	0	1
5791	GO:0021513	1	0.990047839	0	5
5792	GO:0021514	1	0.996016926	0	2
5793	GO:0021515	1	0.998013437	0	1
5794	GO:0021516	1	0.987959017	0	6
5795	GO:0021517	1	0.982164523	0	9
5796	GO:0021520	1	0.993953926	0	3
5797	GO:0021521	1	0.997981567	0	1
5798	GO:0021522	1	0.972403084	0	14
5799	GO:0021523	1	0.995995931	0	2
5800	GO:0021524	1	0.995969098	0	2
5801	GO:0021526	1	0.997976434	0	1
5802	GO:0021527	1	0.986062928	0	7
5803	GO:0021528	1	0.997990564	0	1
5804	GO:0021529	1	0.994019643	0	3
5805	GO:0021530	1	0.997986219	0	1
5806	GO:0021532	1	0.986078308	0	7
5807	GO:0021533	1	0.997976723	0	1
5808	GO:0021534	1	0.997984077	0	1
5809	GO:0021535	1	0.995993074	0	2
5810	GO:0021536	1	0.998013437	0	1
5811	GO:0021537	1	0.950910854	0	25
5812	GO:0021539	1	0.997971376	0	1
5813	GO:0021540	1	0.989997555	0	5
5814	GO:0021541	1	0.998009474	0	1
5815	GO:0021542	1	0.978180975	0	11
5816	GO:0021543	1	0.9939998	0	3
5817	GO:0021544	1	0.993969971	0	3
5818	GO:0021545	1	0.992051131	0	4
5819	GO:0021546	1	0.995967695	0	2
5820	GO:0021547	1	0.997980022	0	1
5821	GO:0021548	1	0.997971376	0	1
5822	GO:0021549	1	0.9155413	0	44
5823	GO:0021551	1	0.998013437	0	1
5824	GO:0021553	1	0.996030484	0	2

5825	GO:0021554	1	0.985958095	0	7
5826	GO:0021555	1	0.995986194	0	2
5827	GO:0021557	1	0.997968974	0	1
5828	GO:0021558	1	0.997968974	0	1
5829	GO:0021559	1	0.993957158	0	3
5830	GO:0021562	1	0.993998111	0	3
5831	GO:0021563	1	0.998013437	0	1
5832	GO:0021564	1	0.998013437	0	1
5833	GO:0021569	1	0.995967815	0	2
5834	GO:0021570	1	0.99593617	0	2
5835	GO:0021571	1	0.995964138	0	2
5836	GO:0021572	1	0.997999653	0	1
5837	GO:0021575	1	0.995986354	0	2
5838	GO:0021587	1	0.99202037	0	4
5839	GO:0021589	1	0.995999228	0	2
5840	GO:0021591	1	0.968388152	0	16
5841	GO:0021592	1	0.998013276	0	1
5842	GO:0021599	1	0.997999653	0	1
5843	GO:0021602	1	0.995979387	0	2
5844	GO:0021610	1	0.994014354	0	3
5845	GO:0021612	1	0.982094254	0	9
5846	GO:0021615	1	0.994013538	0	3
5847	GO:0021618	1	0.998012768	0	1
5848	GO:0021623	1	0.99799889	0	1
5849	GO:0021626	1	0.995980294	0	2
5850	GO:0021629	1	0.998013437	0	1
5851	GO:0021631	1	0.992074591	0	4
5852	GO:0021633	1	0.997980594	0	1
5853	GO:0021636	1	0.99599073	0	2
5854	GO:0021637	1	0.990054637	0	5
5855	GO:0021644	1	0.99597491	0	2
5856	GO:0021649	1	0.996003648	0	2
5857	GO:0021650	1	0.998013437	0	1
5858	GO:0021659	1	0.997992206	0	1
5859	GO:0021660	1	0.997992206	0	1
5860	GO:0021665	1	0.997992206	0	1
5861	GO:0021666	1	0.997992206	0	1
5862	GO:0021670	1	0.976228922	0	12
5863	GO:0021675	1	0.970269652	0	15
5864	GO:0021678	1	0.991943744	0	4
5865	GO:0021680	1	0.982112714	0	9
5866	GO:0021681	1	0.993995503	0	3
5867	GO:0021682	1	0.998013435	0	1
5868	GO:0021683	1	0.99798286	0	1
5869	GO:0021685	1	0.998013437	0	1

5870	GO:0021687	1	0.997995247	0	1
5871	GO:0021688	1	0.997999078	0	1
5872	GO:0021691	1	0.996023707	0	2
5873	GO:0021692	1	0.996007903	0	2
5874	GO:0021693	1	0.9960164	0	2
5875	GO:0021694	1	0.995979641	0	2
5876	GO:0021695	1	0.98998911	0	5
5877	GO:0021696	1	0.992020795	0	4
5878	GO:0021697	1	0.993976556	0	3
5879	GO:0021700	1	0.998013437	0	1
5880	GO:0021702	1	0.982156676	0	9
5881	GO:0021707	1	0.988074126	0	6
5882	GO:0021722	1	0.998006014	0	1
5883	GO:0021740	1	0.996014215	0	2
5884	GO:0021747	1	0.994042479	0	3
5885	GO:0021750	1	0.997986219	0	1
5886	GO:0021754	1	0.997960497	0	1
5887	GO:0021756	1	0.976236378	0	12
5888	GO:0021757	1	0.998003586	0	1
5889	GO:0021758	1	0.998003586	0	1
5890	GO:0021759	1	0.994016413	0	3
5891	GO:0021761	1	0.996029539	0	2
5892	GO:0021762	1	0.917002225	0	43
5893	GO:0021764	1	0.994036757	0	3
5894	GO:0021766	1	0.886514384	0	60
5895	GO:0021767	1	0.997969048	0	1
5896	GO:0021768	1	0.998001701	0	1
5897	GO:0021769	1	0.993987609	0	3
5898	GO:0021771	1	0.998013436	0	1
5899	GO:0021772	1	0.960709104	0	20
5900	GO:0021773	1	0.992044983	0	4
5901	GO:0021775	1	0.994051114	0	3
5902	GO:0021776	1	0.994051114	0	3
5903	GO:0021778	1	0.998013437	0	1
5904	GO:0021779	1	0.997986219	0	1
5905	GO:0021781	1	0.993949459	0	3
5906	GO:0021782	1	0.991988394	0	4
5907	GO:0021784	1	0.995958192	0	2
5908	GO:0021785	1	0.988087385	0	6
5909	GO:0021793	1	0.998000404	0	1
5910	GO:0021794	1	0.98009659	0	10
5911	GO:0021795	1	0.98410537	0	8
5912	GO:0021796	1	0.990030521	0	5
5913	GO:0021797	1	0.997979076	0	1
5914	GO:0021798	1	0.990026777	0	5

5915	GO:0021799	1	0.980196005	0	10
5916	GO:0021800	1	0.992041618	0	4
5917	GO:0021801	1	0.994001494	0	3
5918	GO:0021812	1	0.998013437	0	1
5919	GO:0021813	1	0.997988059	0	1
5920	GO:0021814	1	0.998011102	0	1
5921	GO:0021816	1	0.996007903	0	2
5922	GO:0021817	1	0.994036454	0	3
5923	GO:0021819	1	0.97627464	0	12
5924	GO:0021822	1	0.997990564	0	1
5925	GO:0021828	1	0.992027139	0	4
5926	GO:0021830	1	0.997999639	0	1
5927	GO:0021831	1	0.997992399	0	1
5928	GO:0021836	1	0.996030144	0	2
5929	GO:0021842	1	0.997984757	0	1
5930	GO:0021846	1	0.982062553	0	9
5931	GO:0021847	1	0.995996638	0	2
5932	GO:0021849	1	0.994030123	0	3
5933	GO:0021852	1	0.994002461	0	3
5934	GO:0021853	1	0.992017394	0	4
5935	GO:0021854	1	0.986017109	0	7
5936	GO:0021855	1	0.997969048	0	1
5937	GO:0021859	1	0.996002469	0	2
5938	GO:0021860	1	0.98414756	0	8
5939	GO:0021861	1	0.985969095	0	7
5940	GO:0021869	1	0.996025616	0	2
5941	GO:0021870	1	0.998013437	0	1
5942	GO:0021871	1	0.989996432	0	5
5943	GO:0021872	1	0.997998306	0	1
5944	GO:0021873	1	0.996019045	0	2
5945	GO:0021874	1	0.997993311	0	1
5946	GO:0021879	1	0.993989128	0	3
5947	GO:0021884	1	0.993985447	0	3
5948	GO:0021885	1	0.996023252	0	2
5949	GO:0021888	1	0.99595028	0	2
5950	GO:0021889	1	0.992001365	0	4
5951	GO:0021891	1	0.994021361	0	3
5952	GO:0021892	1	0.995973678	0	2
5953	GO:0021893	1	0.99798349	0	1
5954	GO:0021894	1	0.997961465	0	1
5955	GO:0021895	1	0.976266296	0	12
5956	GO:0021897	1	0.99603063	0	2
5957	GO:0021902	1	0.992049681	0	4
5958	GO:0021903	1	0.995985811	0	2
5959	GO:0021904	1	0.976174185	0	12

5960	GO:0021905	1	0.998013437	0	1
5961	GO:0021910	1	0.998004776	0	1
5962	GO:0021914	1	0.997987562	0	1
5963	GO:0021915	1	0.917433304	0	43
5964	GO:0021919	1	0.998002238	0	1
5965	GO:0021924	1	0.997971213	0	1
5966	GO:0021930	1	0.988014356	0	6
5967	GO:0021935	1	0.996030724	0	2
5968	GO:0021937	1	0.997983203	0	1
5969	GO:0021938	1	0.990042322	0	5
5970	GO:0021940	1	0.992006744	0	4
5971	GO:0021941	1	0.998011379	0	1
5972	GO:0021942	1	0.992022369	0	4
5973	GO:0021943	1	0.992043161	0	4
5974	GO:0021944	1	0.997999366	0	1
5975	GO:0021952	1	0.982241892	0	9
5976	GO:0021953	1	0.98606051	0	7
5977	GO:0021954	1	0.968354843	0	16
5978	GO:0021955	1	0.986085916	0	7
5979	GO:0021956	1	0.99797857	0	1
5980	GO:0021957	1	0.992067178	0	4
5981	GO:0021960	1	0.988064407	0	6
5982	GO:0021965	1	0.994051208	0	3
5983	GO:0021966	1	0.997965715	0	1
5984	GO:0021972	1	0.998012848	0	1
5985	GO:0021978	1	0.987987617	0	6
5986	GO:0021979	1	0.996018622	0	2
5987	GO:0021983	1	0.960612578	0	20
5988	GO:0021984	1	0.993956544	0	3
5989	GO:0021985	1	0.998008316	0	1
5990	GO:0021986	1	0.996019434	0	2
5991	GO:0021987	1	0.863708146	0	73
5992	GO:0021988	1	0.995986215	0	2
5993	GO:0021989	1	0.998013392	0	1
5994	GO:0021993	1	0.998013276	0	1
5995	GO:0021997	1	0.996030731	0	2
5996	GO:0021998	1	0.998003307	0	1
5997	GO:0021999	1	0.998013437	0	1
5998	GO:0022007	1	0.995992048	0	2
5999	GO:0022008	1	0.900816793	0	52
6000	GO:0022009	1	0.990002528	0	5
6001	GO:0022010	1	0.984078878	0	8
6002	GO:0022011	1	0.964623627	0	18
6003	GO:0022013	1	0.998013437	0	1
6004	GO:0022018	1	0.998013437	0	1

6005	GO:0022027	1	0.990104248	0	5
6006	GO:0022028	1	0.992070198	0	4
6007	GO:0022029	1	0.993969242	0	3
6008	GO:0022037	1	0.99598154	0	2
6009	GO:0022038	1	0.980136421	0	10
6010	GO:0022400	1	0.956729672	0	22
6011	GO:0022405	1	0.988044008	0	6
6012	GO:0022406	1	0.997990564	0	1
6013	GO:0022407	1	0.974399148	0	13
6014	GO:0022408	1	0.974347568	0	13
6015	GO:0022409	1	0.974242845	0	13
6016	GO:0022414	1	0.989975072	0	5
6017	GO:0022417	1	0.983902009	0	8
6018	GO:0022602	1	0.993984369	0	3
6019	GO:0022604	1	0.947274415	0	27
6020	GO:0022605	1	0.997956877	0	1
6021	GO:0022607	1	0.997981946	0	1
6022	GO:0022612	1	0.988050893	0	6
6023	GO:0022613	1	0.998004245	0	1
6024	GO:0022614	1	0.990048178	0	5
6025	GO:0022615	1	0.991937345	0	4
6026	GO:0022616	1	0.99800762	0	1
6027	GO:0022617	1	0.891639847	0	57
6028	GO:0022618	1	0.993928599	0	3
6029	GO:0022624	1	0.96613009	0	17
6030	GO:0022625	1	0.89183194	0	56
6031	GO:0022626	1	0.852516649	0	78
6033	GO:0022824	1	0.993989614	0	3
6034	GO:0022829	1	0.995989331	0	2
6035	GO:0022832	1	0.997990564	0	1
6036	GO:0022839	1	0.995985075	0	2
6037	GO:0022840	1	0.993993419	0	3
6038	GO:0022841	1	0.980024049	0	10
6039	GO:0022843	1	0.992037065	0	4
6040	GO:0022848	1	0.972175658	0	14
6041	GO:0022849	1	0.990098148	0	5
6042	GO:0022850	1	0.993941938	0	3
6043	GO:0022851	1	0.984111259	0	8
6044	GO:0022852	1	0.996006289	0	2
6046	GO:0022858	1	0.992040197	0	4
6047	GO:0022889	1	0.991980154	0	4
6048	GO:0022890	1	0.986097541	0	7
6049	GO:0022894	1	0.997982548	0	1
6050	GO:0022898	1	0.986039305	0	7
6051	GO:0022900	1	0.833106457	0	90

6052	GO:0022904	1	0.964274474	0	18
6053	GO:0023019	1	0.968336386	0	16
6054	GO:0023021	1	0.993972021	0	3
6055	GO:0023024	1	0.991959783	0	4
6056	GO:0023025	1	0.997992275	0	1
6057	GO:0023026	1	0.968134455	0	16
6058	GO:0023029	1	0.99400556	0	3
6059	GO:0023030	1	0.995991842	0	2
6060	GO:0023035	1	0.982030095	0	9
6061	GO:0023041	1	0.984059127	0	8
6062	GO:0023051	1	0.992028076	0	4
6063	GO:0023052	1	0.986102437	0	7
6064	GO:0023061	1	0.998013437	0	1
6065	GO:0030001	1	0.935908363	0	33
6066	GO:0030003	1	0.996007655	0	2
6067	GO:0030007	1	0.974283472	0	13
6068	GO:0030008	1	0.970132184	0	15
6069	GO:0030010	1	0.936018488	0	33
6070	GO:0030011	1	0.984105484	0	8
6071	GO:0030014	1	0.968420004	0	16
6072	GO:0030015	1	0.988023679	0	6
6073	GO:0030016	1	0.939560558	0	31
6074	GO:0030017	1	0.913408644	0	45
6076	GO:0030020	1	0.926993836	0	38
6077	GO:0030021	1	0.978192354	0	11
6078	GO:0030023	1	0.982199659	0	9
6079	GO:0030026	1	0.997997916	0	1
6080	GO:0030027	1	0.693251908	0	182
6081	GO:0030029	1	0.994012397	0	3
6082	GO:0030030	1	0.674646277	0	195
6083	GO:0030031	1	0.954774419	0	23
6084	GO:0030032	1	0.934093829	0	34
6085	GO:0030033	1	0.980153232	0	10
6086	GO:0030034	1	0.998002198	0	1
6087	GO:0030035	1	0.994028912	0	3
6088	GO:0030036	1	0.714568912	0	167
6089	GO:0030037	1	0.998012834	0	1
6090	GO:0030038	1	0.998010126	0	1
6091	GO:0030041	1	0.935861876	0	33
6092	GO:0030042	1	0.976164928	0	12
6093	GO:0030043	1	0.991951881	0	4
6094	GO:0030046	1	0.995985515	0	2
6095	GO:0030047	1	0.993995861	0	3
6096	GO:0030048	1	0.966687029	0	17
6097	GO:0030049	1	0.946857275	0	27

6098	GO:0030050	1	0.966693184	0	17
6100	GO:0030055	1	0.984161638	0	8
6101	GO:0030056	1	0.988115672	0	6
6102	GO:0030057	1	0.951318646	0	25
6103	GO:0030060	1	0.991918872	0	4
6104	GO:0030061	1	0.984044721	0	8
6105	GO:0030070	1	0.998012221	0	1
6106	GO:0030071	1	0.980064542	0	10
6107	GO:0030072	1	0.993995824	0	3
6108	GO:0030073	1	0.952804802	0	24
6109	GO:0030091	1	0.989959135	0	5
6110	GO:0030097	1	0.881243636	0	63
6111	GO:0030098	1	0.988029861	0	6
6112	GO:0030099	1	0.958656695	0	21
6113	GO:0030100	1	0.93388544	0	34
6114	GO:0030101	1	0.968199837	0	16
6115	GO:0030103	1	0.997996769	0	1
6116	GO:0030104	1	0.99400709	0	3
6117	GO:0030107	1	0.997992275	0	1
6118	GO:0030108	1	0.997969391	0	1
6119	GO:0030109	1	0.997992275	0	1
6120	GO:0030111	1	0.952986677	0	24
6121	GO:0030116	1	0.991864948	0	4
6122	GO:0030117	1	0.939575667	0	31
6123	GO:0030118	1	0.98605248	0	7
6124	GO:0030119	1	0.985997759	0	7
6125	GO:0030120	1	0.996025059	0	2
6126	GO:0030121	1	0.986077527	0	7
6127	GO:0030122	1	0.982101782	0	9
6128	GO:0030123	1	0.980196452	0	10
6129	GO:0030124	1	0.989976315	0	5
6130	GO:0030125	1	0.98400935	0	8
6131	GO:0030126	1	0.974139762	0	13
6132	GO:0030127	1	0.970399172	0	15
6133	GO:0030128	1	0.998011928	0	1
6134	GO:0030130	1	0.986054708	0	7
6135	GO:0030131	1	0.976217167	0	12
6136	GO:0030132	1	0.984085379	0	8
6137	GO:0030133	1	0.820607985	0	98
6138	GO:0030134	1	0.918960498	0	42
6139	GO:0030135	1	0.99198877	0	4
6140	GO:0030136	1	0.867297946	0	71
6141	GO:0030137	1	0.970196984	0	15
6142	GO:0030139	1	0.882970258	0	62
6143	GO:0030140	1	0.968435643	0	16

6144	GO:0030141	1	0.842128465	0	85
6145	GO:0030142	1	0.998013437	0	1
6146	GO:0030144	1	0.995998446	0	2
6147	GO:0030145	1	0.882795567	0	62
6148	GO:0030148	1	0.900688049	0	52
6149	GO:0030149	1	0.990015569	0	5
6150	GO:0030150	1	0.958357549	0	21
6151	GO:0030151	1	0.991979123	0	4
6152	GO:0030154	1	0.187475265	0	818
6153	GO:0030155	1	0.901104609	0	52
6154	GO:0030156	1	0.990024919	0	5
6155	GO:0030157	1	0.98796518	0	6
6156	GO:0030158	1	0.996019962	0	2
6157	GO:0030159	1	0.943503391	0	29
6158	GO:0030160	1	0.995994037	0	2
6159	GO:0030162	1	0.976101597	0	12
6160	GO:0030163	1	0.928123673	0	37
6161	GO:0030165	1	0.846563736	0	83
6162	GO:0030166	1	0.960721716	0	20
6163	GO:0030167	1	0.99801283	0	1
6164	GO:0030168	1	0.822873725	0	97
6165	GO:0030169	1	0.970359896	0	15
6166	GO:0030170	1	0.90414476	0	50
6167	GO:0030171	1	0.997973936	0	1
6168	GO:0030172	1	0.993896418	0	3
6169	GO:0030173	1	0.886210798	0	60
6170	GO:0030174	1	0.988000692	0	6
6171	GO:0030175	1	0.858545295	0	76
6173	GO:0030177	1	0.934103148	0	34
6174	GO:0030178	1	0.911612037	0	46
6175	GO:0030182	1	0.748047689	0	144
6176	GO:0030183	1	0.888187484	0	59
6177	GO:0030184	1	0.997990556	0	1
6178	GO:0030185	1	0.993935471	0	3
6179	GO:0030187	1	0.995940161	0	2
6180	GO:0030193	1	0.982017789	0	9
6181	GO:0030194	1	0.974128559	0	13
6182	GO:0030195	1	0.974092996	0	13
6183	GO:0030197	1	0.993965087	0	3
6184	GO:0030198	1	0.625769701	0	233
6185	GO:0030199	1	0.823330349	0	97
6186	GO:0030200	1	0.993981387	0	3
6187	GO:0030201	1	0.99403207	0	3
6188	GO:0030202	1	0.997999867	0	1
6189	GO:0030203	1	0.974204844	0	13

6190	GO:0030204	1	0.990063889	0	5
6191	GO:0030205	1	0.996025067	0	2
6192	GO:0030206	1	0.953039319	0	24
6193	GO:0030207	1	0.974212203	0	13
6194	GO:0030208	1	0.978184596	0	11
6195	GO:0030209	1	0.997981777	0	1
6196	GO:0030210	1	0.978275228	0	11
6197	GO:0030211	1	0.997981777	0	1
6198	GO:0030212	1	0.980078965	0	10
6199	GO:0030213	1	0.987992727	0	6
6200	GO:0030214	1	0.970298855	0	15
6201	GO:0030215	1	0.955096155	0	23
6202	GO:0030216	1	0.893253876	0	56
6203	GO:0030217	1	0.928437658	0	37
6204	GO:0030218	1	0.919036345	0	42
6205	GO:0030219	1	0.984069838	0	8
6206	GO:0030220	1	0.968436424	0	16
6207	GO:0030221	1	0.998012887	0	1
6208	GO:0030223	1	0.989988416	0	5
6209	GO:0030224	1	0.968325037	0	16
6210	GO:0030225	1	0.96443115	0	18
6211	GO:0030226	1	0.998013437	0	1
6212	GO:0030228	1	0.997990564	0	1
6213	GO:0030229	1	0.992039747	0	4
6214	GO:0030233	1	0.997972474	0	1
6215	GO:0030234	1	0.948904657	0	26
6216	GO:0030235	1	0.984041342	0	8
6217	GO:0030237	1	0.998006585	0	1
6218	GO:0030238	1	0.988006663	0	6
6219	GO:0030239	1	0.976213805	0	12
6220	GO:0030240	1	0.989985927	0	5
6221	GO:0030241	1	0.993998111	0	3
6222	GO:0030242	1	0.990057729	0	5
6223	GO:0030246	1	0.720828747	0	162
6224	GO:0030247	1	0.976115272	0	12
6225	GO:0030249	1	0.997976977	0	1
6226	GO:0030251	1	0.997983363	0	1
6227	GO:0030252	1	0.99197511	0	4
6228	GO:0030258	1	0.987992922	0	6
6229	GO:0030259	1	0.982042184	0	9
6230	GO:0030261	1	0.952981471	0	24
6231	GO:0030262	1	0.989961829	0	5
6232	GO:0030263	1	0.990053649	0	5
6233	GO:0030264	1	0.997990564	0	1
6234	GO:0030267	1	0.997964949	0	1

6235	GO:0030272	1	0.995952499	0	2
6236	GO:0030273	1	0.997985356	0	1
6237	GO:0030274	1	0.990001288	0	5
6238	GO:0030275	1	0.988050501	0	6
6239	GO:0030276	1	0.917489181	0	43
6240	GO:0030277	1	0.978061666	0	11
6241	GO:0030278	1	0.972294617	0	14
6242	GO:0030279	1	0.962612954	0	19
6243	GO:0030280	1	0.979964133	0	10
6244	GO:0030282	1	0.922701951	0	40
6245	GO:0030283	1	0.989899229	0	5
6246	GO:0030284	1	0.990029331	0	5
6247	GO:0030285	1	0.937657772	0	32
6248	GO:0030286	1	0.930239755	0	36
6249	GO:0030289	1	0.991957386	0	4
6250	GO:0030291	1	0.980150832	0	10
6251	GO:0030292	1	0.994006307	0	3
6252	GO:0030293	1	0.997980063	0	1
6253	GO:0030294	1	0.99798201	0	1
6254	GO:0030295	1	0.945260272	0	28
6255	GO:0030296	1	0.980132491	0	10
6256	GO:0030297	1	0.988067047	0	6
6257	GO:0030298	1	0.99395774	0	3
6258	GO:0030299	1	0.986035017	0	7
6259	GO:0030300	1	0.995962851	0	2
6260	GO:0030301	1	0.952926531	0	24
6261	GO:0030302	1	0.997972474	0	1
6262	GO:0030305	1	0.995988325	0	2
6263	GO:0030307	1	0.859774346	0	75
6264	GO:0030308	1	0.792778644	0	115
6265	GO:0030311	1	0.978105442	0	11
6266	GO:0030314	1	0.992054434	0	4
6267	GO:0030315	1	0.91545999	0	44
6268	GO:0030316	1	0.941457125	0	30
6269	GO:0030317	1	0.873655639	0	67
6270	GO:0030318	1	0.966568861	0	17
6271	GO:0030321	1	0.989979482	0	5
6272	GO:0030322	1	0.97802543	0	11
6273	GO:0030323	1	0.988024601	0	6
6274	GO:0030324	1	0.841303237	0	86
6275	GO:0030325	1	0.966388585	0	17
6276	GO:0030326	1	0.9156157	0	44
6277	GO:0030327	1	0.994009289	0	3
6278	GO:0030328	1	0.996004691	0	2
6279	GO:0030330	1	0.968426075	0	16

6280	GO:0030331	1	0.917278554	0	43
6281	GO:0030332	1	0.939495008	0	31
6284	GO:0030336	1	0.795236106	0	114
6285	GO:0030337	1	0.993940984	0	3
6286	GO:0030342	1	0.997997008	0	1
6287	GO:0030343	1	0.993958226	0	3
6288	GO:0030345	1	0.993972795	0	3
6289	GO:0030346	1	0.988015752	0	6
6290	GO:0030348	1	0.99599326	0	2
6291	GO:0030350	1	0.996019831	0	2
6292	GO:0030366	1	0.997968453	0	1
6293	GO:0030368	1	0.988031432	0	6
6294	GO:0030369	1	0.993994178	0	3
6295	GO:0030371	1	0.976217766	0	12
6297	GO:0030377	1	0.997968733	0	1
6298	GO:0030378	1	0.997986313	0	1
6299	GO:0030379	1	0.998013437	0	1
6300	GO:0030382	1	0.994006307	0	3
6301	GO:0030387	1	0.99796864	0	1
6302	GO:0030388	1	0.981981987	0	9
6303	GO:0030389	1	0.995926573	0	2
6304	GO:0030393	1	0.99796864	0	1
6305	GO:0030395	1	0.997951264	0	1
6306	GO:0030407	1	0.997976909	0	1
6307	GO:0030409	1	0.997976909	0	1
6308	GO:0030412	1	0.997976909	0	1
6310	GO:0030421	1	0.997958205	0	1
6311	GO:0030422	1	0.980077703	0	10
6312	GO:0030423	1	0.99199202	0	4
6315	GO:0030426	1	0.754798319	0	140
6316	GO:0030427	1	0.988038947	0	6
6317	GO:0030429	1	0.997969613	0	1
6318	GO:0030431	1	0.986057964	0	7
6319	GO:0030432	1	0.989956355	0	5
6320	GO:0030433	1	0.863388279	0	73
6321	GO:0030449	1	0.926257536	0	38
6322	GO:0030473	1	0.998006367	0	1
6323	GO:0030478	1	0.992042271	0	4
6324	GO:0030479	1	0.972172233	0	14
6325	GO:0030485	1	0.995986896	0	2
6326	GO:0030488	1	0.928234665	0	37
6327	GO:0030490	1	0.968203315	0	16
6328	GO:0030492	1	0.993944463	0	3
6329	GO:0030496	1	0.709839883	0	170
6330	GO:0030497	1	0.986011145	0	7

6331	GO:0030500	1	0.964422039	0	18
6333	GO:0030502	1	0.976203854	0	12
6334	GO:0030504	1	0.996010598	0	2
6335	GO:0030505	1	0.994031781	0	3
6336	GO:0030506	1	0.960806332	0	20
6338	GO:0030509	1	0.884781698	0	61
6339	GO:0030510	1	0.978163623	0	11
6340	GO:0030511	1	0.94533556	0	28
6341	GO:0030512	1	0.865239172	0	72
6342	GO:0030513	1	0.934132257	0	34
6343	GO:0030514	1	0.90272161	0	51
6344	GO:0030515	1	0.960396595	0	20
6345	GO:0030516	1	0.97429006	0	13
6346	GO:0030517	1	0.95685651	0	22
6347	GO:0030518	1	0.978192447	0	11
6348	GO:0030520	1	0.968438412	0	16
6349	GO:0030521	1	0.968420378	0	16
6351	GO:0030523	1	0.998010344	0	1
6352	GO:0030526	1	0.998012538	0	1
6353	GO:0030527	1	0.997978212	0	1
6354	GO:0030532	1	0.968104711	0	16
6355	GO:0030534	1	0.947458984	0	27
6356	GO:0030538	1	0.998008429	0	1
6357	GO:0030539	1	0.970323468	0	15
6358	GO:0030540	1	0.992023998	0	4
6359	GO:0030544	1	0.917042383	0	43
6360	GO:0030545	1	0.993940794	0	3
6361	GO:0030546	1	0.996018651	0	2
6362	GO:0030547	1	0.995987565	0	2
6363	GO:0030548	1	0.985998314	0	7
6364	GO:0030549	1	0.995918127	0	2
6365	GO:0030550	1	0.987970008	0	6
6366	GO:0030551	1	0.995976873	0	2
6367	GO:0030552	1	0.956924075	0	22
6368	GO:0030553	1	0.976258247	0	12
6369	GO:0030554	1	0.997983347	0	1
6370	GO:0030573	1	0.995972049	0	2
6371	GO:0030574	1	0.928272472	0	37
6372	GO:0030575	1	0.996011342	0	2
6373	GO:0030576	1	0.991977256	0	4
6374	GO:0030578	1	0.99006172	0	5
6375	GO:0030579	1	0.99001947	0	5
6376	GO:0030586	1	0.997998773	0	1
6377	GO:0030592	1	0.993980418	0	3
6379	GO:0030594	1	0.898644084	0	53

6380	GO:0030595	1	0.974075857	0	13
6381	GO:0030619	1	0.98396403	0	8
6382	GO:0030620	1	0.987986206	0	6
6383	GO:0030621	1	0.988035878	0	6
6384	GO:0030622	1	0.993997458	0	3
6385	GO:0030623	1	0.99602607	0	2
6386	GO:0030624	1	0.998010534	0	1
6387	GO:0030626	1	0.995986543	0	2
6388	GO:0030627	1	0.9979641	0	1
6389	GO:0030628	1	0.987934112	0	6
6390	GO:0030629	1	0.995948698	0	2
6391	GO:0030641	1	0.988008394	0	6
6392	GO:0030643	1	0.98605346	0	7
6393	GO:0030644	1	0.990028098	0	5
6394	GO:0030658	1	0.92269283	0	40
6396	GO:0030660	1	0.968423666	0	16
6397	GO:0030662	1	0.997990564	0	1
6398	GO:0030663	1	0.968203531	0	16
6399	GO:0030665	1	0.96454166	0	18
6400	GO:0030666	1	0.886143567	0	60
6401	GO:0030667	1	0.835817233	0	89
6402	GO:0030669	1	0.88069109	0	63
6403	GO:0030670	1	0.863071139	0	73
6404	GO:0030672	1	0.860062843	0	75
6405	GO:0030673	1	0.97049526	0	15
6406	GO:0030674	1	0.826041924	0	95
6407	GO:0030677	1	0.997986368	0	1
6408	GO:0030678	1	0.993952377	0	3
6409	GO:0030681	1	0.979954118	0	10
6410	GO:0030682	1	0.998013437	0	1
6411	GO:0030683	1	0.996006971	0	2
6412	GO:0030684	1	0.993904876	0	3
6413	GO:0030686	1	0.976240625	0	12
6414	GO:0030687	1	0.956572085	0	22
6415	GO:0030688	1	0.974215696	0	13
6416	GO:0030690	1	0.997991486	0	1
6417	GO:0030691	1	0.997991486	0	1
6418	GO:0030692	1	0.995969726	0	2
6419	GO:0030695	1	0.96255627	0	19
6420	GO:0030697	1	0.997996844	0	1
6421	GO:0030704	1	0.997979743	0	1
6422	GO:0030705	1	0.962689352	0	19
6423	GO:0030718	1	0.995991778	0	2
6424	GO:0030719	1	0.995993742	0	2
6425	GO:0030728	1	0.98205656	0	9

6426	GO:0030729	1	0.997999381	0	1
6427	GO:0030730	1	0.991939471	0	4
6428	GO:0030731	1	0.997960398	0	1
6429	GO:0030735	1	0.997990564	0	1
6430	GO:0030742	1	0.958609858	0	21
6431	GO:0030748	1	0.997987515	0	1
6432	GO:0030791	1	0.997986156	0	1
6433	GO:0030792	1	0.996002523	0	2
6434	GO:0030832	1	0.99602575	0	2
6435	GO:0030833	1	0.928180023	0	37
6436	GO:0030834	1	0.995985401	0	2
6437	GO:0030835	1	0.992058517	0	4
6438	GO:0030836	1	0.984040034	0	8
6439	GO:0030837	1	0.964521417	0	18
6440	GO:0030838	1	0.907865023	0	48
6441	GO:0030844	1	0.998013416	0	1
6442	GO:0030845	1	0.997992136	0	1
6443	GO:0030849	1	0.995991196	0	2
6444	GO:0030850	1	0.978200084	0	11
6445	GO:0030851	1	0.962553834	0	19
6446	GO:0030852	1	0.997999158	0	1
6447	GO:0030853	1	0.988098236	0	6
6448	GO:0030854	1	0.987955409	0	6
6449	GO:0030855	1	0.873351396	0	67
6450	GO:0030856	1	0.982121475	0	9
6451	GO:0030857	1	0.982165323	0	9
6452	GO:0030858	1	0.990032679	0	5
6453	GO:0030859	1	0.990080147	0	5
6454	GO:0030860	1	0.997986579	0	1
6455	GO:0030862	1	0.998013416	0	1
6456	GO:0030863	1	0.960707889	0	20
6457	GO:0030864	1	0.904563581	0	50
6458	GO:0030865	1	0.962396703	0	19
6459	GO:0030866	1	0.941739182	0	30
6460	GO:0030867	1	0.980154993	0	10
6461	GO:0030868	1	0.989953735	0	5
6462	GO:0030870	1	0.986073153	0	7
6463	GO:0030877	1	0.976259977	0	12
6464	GO:0030878	1	0.962527857	0	19
6465	GO:0030879	1	0.947369783	0	27
6466	GO:0030880	1	0.995977536	0	2
6467	GO:0030881	1	0.985916472	0	7
6468	GO:0030882	1	0.998005257	0	1
6469	GO:0030883	1	0.998005257	0	1
6470	GO:0030884	1	0.998005257	0	1

6471	GO:0030886	1	0.995960025	0	2
6472	GO:0030888	1	0.992009637	0	4
6473	GO:0030889	1	0.976153401	0	12
6474	GO:0030890	1	0.931982571	0	35
6475	GO:0030891	1	0.991967868	0	4
6476	GO:0030892	1	0.997990564	0	1
6477	GO:0030893	1	0.990063729	0	5
6478	GO:0030894	1	0.995952292	0	2
6479	GO:0030895	1	0.993958906	0	3
6480	GO:0030896	1	0.989950518	0	5
6481	GO:0030897	1	0.976261125	0	12
6482	GO:0030898	1	0.95898469	0	21
6483	GO:0030899	1	0.996018399	0	2
6484	GO:0030900	1	0.892017048	0	57
6485	GO:0030901	1	0.949100708	0	26
6486	GO:0030902	1	0.974298217	0	13
6487	GO:0030903	1	0.98211114	0	9
6488	GO:0030904	1	0.960595694	0	20
6489	GO:0030905	1	0.991992269	0	4
6490	GO:0030906	1	0.991977164	0	4
6491	GO:0030910	1	0.994010065	0	3
6492	GO:0030911	1	0.98412565	0	8
6493	GO:0030913	1	0.988043756	0	6
6494	GO:0030914	1	0.976099262	0	12
6495	GO:0030915	1	0.985963124	0	7
6496	GO:0030916	1	0.987972256	0	6
6497	GO:0030917	1	0.995978555	0	2
6498	GO:0030942	1	0.993944349	0	3
6499	GO:0030943	1	0.989945163	0	5
6500	GO:0030947	1	0.993964388	0	3
6501	GO:0030948	1	0.976224873	0	12
6502	GO:0030949	1	0.978136124	0	11
6503	GO:0030950	1	0.991970702	0	4
6504	GO:0030951	1	0.992016726	0	4
6505	GO:0030953	1	0.980246728	0	10
6506	GO:0030954	1	0.998013175	0	1
6508	GO:0030956	1	0.993959391	0	3
6509	GO:0030957	1	0.98007397	0	10
6510	GO:0030961	1	0.997990564	0	1
6511	GO:0030968	1	0.904186548	0	50
6512	GO:0030970	1	0.970265291	0	15
6513	GO:0030971	1	0.891643291	0	57
6514	GO:0030974	1	0.995965373	0	2
6515	GO:0030975	1	0.997984426	0	1
6516	GO:0030976	1	0.988028385	0	6

6517	GO:0030977	1	0.997975423	0	1
6518	GO:0030981	1	0.996005899	0	2
6519	GO:0030983	1	0.976221638	0	12
6520	GO:0030984	1	0.995958063	0	2
6521	GO:0030990	1	0.997968023	0	1
6522	GO:0030991	1	0.986138984	0	7
6523	GO:0030992	1	0.964403424	0	18
6524	GO:0030997	1	0.996017989	0	2
6525	GO:0031000	1	0.980175519	0	10
6526	GO:0031004	1	0.996028827	0	2
6527	GO:0031005	1	0.974279689	0	13
6528	GO:0031010	1	0.996023524	0	2
6529	GO:0031011	1	0.968180642	0	16
6530	GO:0031012	1	0.610325718	0	245
6531	GO:0031013	1	0.989891626	0	5
6532	GO:0031014	1	0.991851077	0	4
6533	GO:0031016	1	0.958664647	0	21
6534	GO:0031017	1	0.993992893	0	3
6535	GO:0031018	1	0.966357549	0	17
6536	GO:0031021	1	0.995985075	0	2
6537	GO:0031022	1	0.99602408	0	2
6538	GO:0031023	1	0.980252975	0	10
6539	GO:0031032	1	0.941796625	0	30
6540	GO:0031034	1	0.99800419	0	1
6541	GO:0031045	1	0.972307539	0	14
6542	GO:0031047	1	0.884687465	0	61
6543	GO:0031048	1	0.994026914	0	3
6544	GO:0031052	1	0.996008426	0	2
6545	GO:0031053	1	0.978206187	0	11
6546	GO:0031054	1	0.974176081	0	13
6547	GO:0031056	1	0.990027426	0	5
6548	GO:0031058	1	0.99798739	0	1
6549	GO:0031060	1	0.994013884	0	3
6550	GO:0031061	1	0.996030731	0	2
6551	GO:0031062	1	0.986011227	0	7
6552	GO:0031063	1	0.991955765	0	4
6553	GO:0031064	1	0.987963393	0	6
6554	GO:0031065	1	0.974230594	0	13
6555	GO:0031069	1	0.952958575	0	24
6556	GO:0031071	1	0.997984726	0	1
6557	GO:0031072	1	0.875463544	0	66
6558	GO:0031073	1	0.99798341	0	1
6559	GO:0031076	1	0.978241949	0	11
6560	GO:0031077	1	0.992045445	0	4
6561	GO:0031080	1	0.980134651	0	10

6562	GO:0031083	1	0.97015967	0	15
6563	GO:0031084	1	0.994023683	0	3
6564	GO:0031085	1	0.996003339	0	2
6565	GO:0031086	1	0.995972843	0	2
6566	GO:0031087	1	0.990036181	0	5
6567	GO:0031088	1	0.988016095	0	6
6568	GO:0031089	1	0.975989082	0	12
6569	GO:0031090	1	0.972261843	0	14
6570	GO:0031091	1	0.976199	0	12
6571	GO:0031092	1	0.968426259	0	16
6572	GO:0031093	1	0.889692978	0	58
6573	GO:0031094	1	0.996022831	0	2
6574	GO:0031095	1	0.982189819	0	9
6575	GO:0031098	1	0.96074117	0	20
6576	GO:0031099	1	0.997977819	0	1
6577	GO:0031100	1	0.924528805	0	39
6578	GO:0031102	1	0.989997578	0	5
6579	GO:0031103	1	0.972345822	0	14
6580	GO:0031104	1	0.997970686	0	1
6581	GO:0031105	1	0.972224266	0	14
6582	GO:0031106	1	0.995998138	0	2
6583	GO:0031109	1	0.997954014	0	1
6584	GO:0031110	1	0.972217537	0	14
6585	GO:0031111	1	0.996022111	0	2
6586	GO:0031113	1	0.978192616	0	11
6587	GO:0031114	1	0.99403705	0	3
6588	GO:0031115	1	0.976221485	0	12
6589	GO:0031116	1	0.947280241	0	27
6590	GO:0031117	1	0.994010789	0	3
6591	GO:0031118	1	0.991882148	0	4
6592	GO:0031119	1	0.989960737	0	5
6593	GO:0031120	1	0.993907034	0	3
6594	GO:0031122	1	0.908251234	0	48
6595	GO:0031123	1	0.978139981	0	11
6596	GO:0031124	1	0.889790252	0	58
6597	GO:0031125	1	0.998011721	0	1
6598	GO:0031127	1	0.99600925	0	2
6599	GO:0031129	1	0.99801319	0	1
6600	GO:0031133	1	0.992001068	0	4
6601	GO:0031134	1	0.996006828	0	2
6602	GO:0031143	1	0.968362856	0	16
6603	GO:0031145	1	0.841579998	0	85
6604	GO:0031146	1	0.831790267	0	91
6605	GO:0031151	1	0.998013437	0	1
6606	GO:0031161	1	0.997996596	0	1

6607	GO:0031167	1	0.966206165	0	17
6608	GO:0031175	1	0.776069848	0	126
6609	GO:0031177	1	0.998013437	0	1
6610	GO:0031179	1	0.995979035	0	2
6611	GO:0031201	1	0.907795384	0	48
6612	GO:0031204	1	0.986005761	0	7
6613	GO:0031205	1	0.997951367	0	1
6614	GO:0031207	1	0.998013437	0	1
6615	GO:0031208	1	0.994003424	0	3
6616	GO:0031209	1	0.978254334	0	11
6617	GO:0031210	1	0.95289322	0	24
6618	GO:0031213	1	0.996023218	0	2
6619	GO:0031214	1	0.948847285	0	26
6620	GO:0031223	1	0.994013606	0	3
6621	GO:0031224	1	0.974277414	0	13
6622	GO:0031225	1	0.805526053	0	107
6623	GO:0031226	1	0.930165302	0	36
6624	GO:0031227	1	0.98210111	0	9
6625	GO:0031228	1	0.98998366	0	5
6626	GO:0031231	1	0.997961504	0	1
6627	GO:0031232	1	0.983997294	0	8
6628	GO:0031233	1	0.995995671	0	2
6629	GO:0031234	1	0.879348746	0	64
6630	GO:0031235	1	0.992028279	0	4
6631	GO:0031247	1	0.995973781	0	2
6632	GO:0031249	1	0.997985419	0	1
6633	GO:0031251	1	0.996030595	0	2
6634	GO:0031252	1	0.89196191	0	57
6635	GO:0031253	1	0.974286598	0	13
6636	GO:0031256	1	0.982123977	0	9
6637	GO:0031258	1	0.956847106	0	22
6638	GO:0031259	1	0.997990564	0	1
6639	GO:0031260	1	0.997961917	0	1
6640	GO:0031261	1	0.995971851	0	2
6641	GO:0031262	1	0.99190339	0	4
6642	GO:0031264	1	0.985979401	0	7
6643	GO:0031265	1	0.987993302	0	6
6644	GO:0031267	1	0.5899767	0	262
6645	GO:0031268	1	0.997961917	0	1
6646	GO:0031274	1	0.976138092	0	12
6647	GO:0031275	1	0.997990564	0	1
6648	GO:0031279	1	0.998009271	0	1
6649	GO:0031281	1	0.993966961	0	3
6650	GO:0031282	1	0.997976977	0	1
6651	GO:0031283	1	0.993973055	0	3

6652	GO:0031284	1	0.990008201	0	5
6653	GO:0031290	1	0.968467809	0	16
6654	GO:0031291	1	0.994002755	0	3
6655	GO:0031293	1	0.964502021	0	18
6656	GO:0031295	1	0.911461086	0	46
6657	GO:0031296	1	0.993898761	0	3
6658	GO:0031297	1	0.937707161	0	32
6659	GO:0031298	1	0.994002035	0	3
6660	GO:0031300	1	0.997997582	0	1
6661	GO:0031301	1	0.980028787	0	10
6662	GO:0031302	1	0.998013105	0	1
6663	GO:0031303	1	0.995969937	0	2
6664	GO:0031304	1	0.995940827	0	2
6665	GO:0031305	1	0.920484379	0	41
6666	GO:0031306	1	0.996017614	0	2
6667	GO:0031307	1	0.954724982	0	23
6668	GO:0031309	1	0.997990564	0	1
6669	GO:0031312	1	0.995959969	0	2
6670	GO:0031313	1	0.984039608	0	8
6671	GO:0031314	1	0.972154353	0	14
6672	GO:0031315	1	0.998013437	0	1
6673	GO:0031323	1	0.991958387	0	4
6674	GO:0031324	1	0.998000158	0	1
6675	GO:0031325	1	0.988102652	0	6
6676	GO:0031328	1	0.99193711	0	4
6677	GO:0031333	1	0.932096681	0	35
6678	GO:0031334	1	0.900509322	0	52
6679	GO:0031338	1	0.996001226	0	2
6680	GO:0031339	1	0.995989508	0	2
6681	GO:0031340	1	0.986014561	0	7
6682	GO:0031343	1	0.981985375	0	9
6683	GO:0031344	1	0.994018312	0	3
6684	GO:0031345	1	0.99599709	0	2
6685	GO:0031346	1	0.988013108	0	6
6686	GO:0031362	1	0.972157314	0	14
6687	GO:0031365	1	0.99197032	0	4
6688	GO:0031369	1	0.952824708	0	24
6689	GO:0031370	1	0.998012228	0	1
6690	GO:0031371	1	0.995959495	0	2
6691	GO:0031372	1	0.99596163	0	2
6692	GO:0031379	1	0.99800762	0	1
6693	GO:0031380	1	0.996007903	0	2
6695	GO:0031389	1	0.997992802	0	1
6696	GO:0031390	1	0.983989659	0	8
6697	GO:0031391	1	0.994006309	0	3

6698	GO:0031392	1	0.9979534	0	1
6699	GO:0031393	1	0.996006111	0	2
6700	GO:0031394	1	0.987971576	0	6
6701	GO:0031396	1	0.962476812	0	19
6703	GO:0031398	1	0.863430906	0	73
6704	GO:0031401	1	0.991955097	0	4
6705	GO:0031402	1	0.986131598	0	7
6706	GO:0031403	1	0.996017625	0	2
6707	GO:0031404	1	0.980094602	0	10
6708	GO:0031405	1	0.998013437	0	1
6709	GO:0031406	1	0.983936624	0	8
6710	GO:0031408	1	0.995988731	0	2
6712	GO:0031415	1	0.991973572	0	4
6713	GO:0031416	1	0.995978958	0	2
6714	GO:0031417	1	0.994025488	0	3
6715	GO:0031418	1	0.96247411	0	19
6716	GO:0031419	1	0.982056909	0	9
6717	GO:0031422	1	0.998001511	0	1
6718	GO:0031424	1	0.835074644	0	89
6719	GO:0031427	1	0.994015393	0	3
6720	GO:0031428	1	0.985934198	0	7
6721	GO:0031429	1	0.98990497	0	5
6722	GO:0031430	1	0.954828999	0	23
6723	GO:0031431	1	0.996002076	0	2
6724	GO:0031432	1	0.978135963	0	11
6725	GO:0031433	1	0.989991641	0	5
6726	GO:0031434	1	0.974295499	0	13
6727	GO:0031435	1	0.964575525	0	18
6728	GO:0031436	1	0.993971583	0	3
6729	GO:0031439	1	0.998010676	0	1
6730	GO:0031440	1	0.994025502	0	3
6731	GO:0031441	1	0.997988184	0	1
6732	GO:0031442	1	0.986034482	0	7
6733	GO:0031443	1	0.998013437	0	1
6734	GO:0031444	1	0.995927744	0	2
6735	GO:0031448	1	0.998002124	0	1
6736	GO:0031453	1	0.998013437	0	1
6737	GO:0031460	1	0.998012647	0	1
6738	GO:0031461	1	0.970318303	0	15
6739	GO:0031462	1	0.970309763	0	15
6740	GO:0031463	1	0.926624694	0	38
6741	GO:0031464	1	0.976151264	0	12
6742	GO:0031465	1	0.990000376	0	5
6743	GO:0031466	1	0.985957287	0	7
6744	GO:0031467	1	0.991984189	0	4

6745	GO:0031468	1	0.984028514	0	8
6746	GO:0031475	1	0.997970229	0	1
6747	GO:0031477	1	0.998013437	0	1
6748	GO:0031489	1	0.97024288	0	15
6749	GO:0031490	1	0.884838835	0	61
6750	GO:0031491	1	0.947285218	0	27
6751	GO:0031492	1	0.928221008	0	37
6752	GO:0031493	1	0.987999803	0	6
6753	GO:0031497	1	0.984088586	0	8
6754	GO:0031499	1	0.991987728	0	4
6755	GO:0031501	1	0.995937514	0	2
6756	GO:0031503	1	0.99599783	0	2
6757	GO:0031507	1	0.990043839	0	5
6758	GO:0031508	1	0.995963596	0	2
6759	GO:0031510	1	0.995979383	0	2
6760	GO:0031511	1	0.997972187	0	1
6761	GO:0031514	1	0.758337173	0	137
6762	GO:0031515	1	0.993969553	0	3
6763	GO:0031519	1	0.947159515	0	27
6764	GO:0031523	1	0.993973728	0	3
6765	GO:0031526	1	0.900849207	0	52
6766	GO:0031527	1	0.968456498	0	16
6767	GO:0031528	1	0.95303788	0	24
6768	GO:0031529	1	0.974218432	0	13
6769	GO:0031530	1	0.995925821	0	2
6770	GO:0031531	1	0.995925808	0	2
6771	GO:0031532	1	0.899132937	0	53
6772	GO:0031533	1	0.994006307	0	3
6773	GO:0031536	1	0.991918282	0	4
6774	GO:0031543	1	0.992002349	0	4
6775	GO:0031544	1	0.997994788	0	1
6776	GO:0031545	1	0.993995793	0	3
6777	GO:0031547	1	0.99009845	0	5
6778	GO:0031549	1	0.998013356	0	1
6779	GO:0031550	1	0.996008341	0	2
6780	GO:0031571	1	0.982088262	0	9
6781	GO:0031573	1	0.968253996	0	16
6782	GO:0031579	1	0.992029136	0	4
6783	GO:0031580	1	0.995954532	0	2
6784	GO:0031581	1	0.97629696	0	12
6785	GO:0031583	1	0.99798021	0	1
6786	GO:0031584	1	0.987957715	0	6
6787	GO:0031585	1	0.998003428	0	1
6788	GO:0031587	1	0.992030745	0	4
6789	GO:0031588	1	0.982100603	0	9

6790	GO:0031589	1	0.964659641	0	18
6791	GO:0031591	1	0.99199151	0	4
6792	GO:0031592	1	0.998013437	0	1
6793	GO:0031593	1	0.935752536	0	33
6794	GO:0031594	1	0.874254469	0	67
6795	GO:0031595	1	0.993955124	0	3
6796	GO:0031597	1	0.985952063	0	7
6797	GO:0031616	1	0.968279786	0	16
6798	GO:0031620	1	0.995988776	0	2
6799	GO:0031622	1	0.989985336	0	5
6801	GO:0031624	1	0.935989311	0	33
6803	GO:0031626	1	0.998013198	0	1
6804	GO:0031627	1	0.996000357	0	2
6805	GO:0031628	1	0.997979463	0	1
6806	GO:0031629	1	0.976114722	0	12
6807	GO:0031630	1	0.990011036	0	5
6808	GO:0031635	1	0.995993059	0	2
6809	GO:0031638	1	0.96821263	0	16
6810	GO:0031639	1	0.985986283	0	7
6811	GO:0031640	1	0.962035221	0	19
6812	GO:0031641	1	0.958705974	0	21
6813	GO:0031642	1	0.980143751	0	10
6814	GO:0031643	1	0.97238574	0	14
6815	GO:0031644	1	0.996003425	0	2
6816	GO:0031647	1	0.847793344	0	82
6817	GO:0031648	1	0.909823919	0	47
6818	GO:0031649	1	0.995975333	0	2
6819	GO:0031651	1	0.998009002	0	1
6820	GO:0031652	1	0.991992975	0	4
6821	GO:0031663	1	0.939445144	0	31
6822	GO:0031664	1	0.991994337	0	4
6823	GO:0031665	1	0.98397574	0	8
6824	GO:0031666	1	0.986024951	0	7
6825	GO:0031667	1	0.880725529	0	63
6826	GO:0031668	1	0.970309123	0	15
6827	GO:0031669	1	0.972261681	0	14
6828	GO:0031670	1	0.989989064	0	5
6829	GO:0031672	1	0.976201485	0	12
6830	GO:0031673	1	0.99601944	0	2
6831	GO:0031674	1	0.962408584	0	19
6832	GO:0031680	1	0.981973502	0	9
6833	GO:0031681	1	0.968225167	0	16
6834	GO:0031682	1	0.98802735	0	6
6835	GO:0031683	1	0.962451738	0	19
6836	GO:0031685	1	0.99600265	0	2

6837	GO:0031686	1	0.997995931	0	1
6838	GO:0031687	1	0.995964815	0	2
6839	GO:0031690	1	0.997963499	0	1
6840	GO:0031691	1	0.995962914	0	2
6841	GO:0031692	1	0.993976981	0	3
6842	GO:0031694	1	0.989949016	0	5
6843	GO:0031695	1	0.997985992	0	1
6844	GO:0031696	1	0.995996116	0	2
6845	GO:0031697	1	0.990052955	0	5
6846	GO:0031698	1	0.99003483	0	5
6847	GO:0031699	1	0.998009002	0	1
6848	GO:0031700	1	0.9979689	0	1
6849	GO:0031701	1	0.995962914	0	2
6850	GO:0031702	1	0.986101514	0	7
6851	GO:0031703	1	0.997987857	0	1
6852	GO:0031704	1	0.995992197	0	2
6853	GO:0031707	1	0.99798021	0	1
6854	GO:0031708	1	0.995949195	0	2
6855	GO:0031710	1	0.997960745	0	1
6856	GO:0031711	1	0.997999789	0	1
6857	GO:0031715	1	0.998013114	0	1
6858	GO:0031720	1	0.9898011	0	5
6859	GO:0031721	1	0.993868591	0	3
6860	GO:0031724	1	0.997964216	0	1
6861	GO:0031726	1	0.995944849	0	2
6862	GO:0031727	1	0.997955114	0	1
6863	GO:0031728	1	0.99390125	0	3
6864	GO:0031729	1	0.99592123	0	2
6866	GO:0031731	1	0.991867925	0	4
6867	GO:0031732	1	0.99795385	0	1
6868	GO:0031735	1	0.99388836	0	3
6869	GO:0031737	1	0.997998423	0	1
6870	GO:0031748	1	0.984004787	0	8
6871	GO:0031749	1	0.990027268	0	5
6872	GO:0031750	1	0.993981711	0	3
6873	GO:0031751	1	0.995982362	0	2
6874	GO:0031752	1	0.994008632	0	3
6875	GO:0031755	1	0.99598567	0	2
6876	GO:0031762	1	0.995962914	0	2
6877	GO:0031768	1	0.997954504	0	1
6878	GO:0031773	1	0.997954463	0	1
6879	GO:0031775	1	0.998013437	0	1
6880	GO:0031780	1	0.997982034	0	1
6881	GO:0031781	1	0.995951561	0	2
6882	GO:0031782	1	0.995951561	0	2

6883	GO:0031783	1	0.997982034	0	1
6884	GO:0031798	1	0.998013437	0	1
6885	GO:0031799	1	0.995963877	0	2
6886	GO:0031800	1	0.991976016	0	4
6887	GO:0031802	1	0.989982498	0	5
6888	GO:0031812	1	0.998006527	0	1
6889	GO:0031821	1	0.992044448	0	4
6890	GO:0031826	1	0.989937065	0	5
6891	GO:0031838	1	0.987788345	0	6
6892	GO:0031839	1	0.997956554	0	1
6893	GO:0031840	1	0.997956554	0	1
6894	GO:0031848	1	0.986047508	0	7
6895	GO:0031849	1	0.991938291	0	4
6896	GO:0031852	1	0.992011446	0	4
6897	GO:0031859	1	0.997977011	0	1
6898	GO:0031860	1	0.992042721	0	4
6899	GO:0031867	1	0.998005881	0	1
6900	GO:0031870	1	0.998013437	0	1
6901	GO:0031871	1	0.994001757	0	3
6902	GO:0031883	1	0.997980267	0	1
6903	GO:0031894	1	0.998008897	0	1
6904	GO:0031896	1	0.997981914	0	1
6905	GO:0031901	1	0.728909342	0	157
6907	GO:0031904	1	0.976152838	0	12
6908	GO:0031905	1	0.993964834	0	3
6909	GO:0031906	1	0.997968714	0	1
6910	GO:0031914	1	0.993973816	0	3
6911	GO:0031915	1	0.986061133	0	7
6912	GO:0031929	1	0.96253391	0	19
6913	GO:0031930	1	0.995927051	0	2
6914	GO:0031931	1	0.98410424	0	8
6915	GO:0031932	1	0.976209374	0	12
6916	GO:0031936	1	0.974211816	0	13
6917	GO:0031937	1	0.996029156	0	2
6918	GO:0031938	1	0.997977315	0	1
6919	GO:0031940	1	0.997972367	0	1
6920	GO:0031941	1	0.937716743	0	32
6921	GO:0031943	1	0.997971738	0	1
6922	GO:0031947	1	0.998004954	0	1
6923	GO:0031952	1	0.984115906	0	8
6924	GO:0031953	1	0.980177824	0	10
6925	GO:0031954	1	0.95497276	0	23
6926	GO:0031956	1	0.989957631	0	5
6927	GO:0031957	1	0.984047939	0	8
6928	GO:0031959	1	0.998013295	0	1

6929	GO:0031960	1	0.986047671	0	7
6930	GO:0031962	1	0.998013437	0	1
6933	GO:0031981	1	0.992014289	0	4
6935	GO:0031983	1	0.998013437	0	1
6936	GO:0031985	1	0.980092219	0	10
6937	GO:0031987	1	0.990010728	0	5
6938	GO:0031989	1	0.997967083	0	1
6939	GO:0031990	1	0.996001191	0	2
6940	GO:0031994	1	0.976261908	0	12
6941	GO:0031995	1	0.986021781	0	7
6942	GO:0031996	1	0.978138988	0	11
6943	GO:0031997	1	0.993970477	0	3
6944	GO:0031998	1	0.988083561	0	6
6945	GO:0031999	1	0.990025709	0	5
6946	GO:0032000	1	0.984171823	0	8
6947	GO:0032002	1	0.995972086	0	2
6948	GO:0032006	1	0.956730939	0	22
6949	GO:0032007	1	0.951092046	0	25
6950	GO:0032008	1	0.93575149	0	33
6951	GO:0032009	1	0.98018211	0	10
6952	GO:0032010	1	0.99194295	0	4
6953	GO:0032012	1	0.966644339	0	17
6954	GO:0032014	1	0.99601423	0	2
6956	GO:0032021	1	0.993983524	0	3
6957	GO:0032023	1	0.99798314	0	1
6958	GO:0032024	1	0.920824602	0	41
6959	GO:0032025	1	0.987983499	0	6
6960	GO:0032026	1	0.978178102	0	11
6961	GO:0032027	1	0.991969132	0	4
6962	GO:0032028	1	0.997980365	0	1
6963	GO:0032029	1	0.998013437	0	1
6964	GO:0032034	1	0.998013437	0	1
6965	GO:0032036	1	0.983944717	0	8
6966	GO:0032038	1	0.997958906	0	1
6967	GO:0032039	1	0.964552106	0	18
6968	GO:0032040	1	0.926265334	0	38
6969	GO:0032041	1	0.980170358	0	10
6970	GO:0032042	1	0.990024817	0	5
6971	GO:0032044	1	0.995978233	0	2
6972	GO:0032045	1	0.97625496	0	12
6973	GO:0032048	1	0.99599368	0	2
6974	GO:0032049	1	0.984025742	0	8
6975	GO:0032050	1	0.98209913	0	9
6976	GO:0032051	1	0.988060432	0	6
6977	GO:0032052	1	0.985920911	0	7

6978	GO:0032053	1	0.992029571	0	4
6979	GO:0032055	1	0.995988284	0	2
6980	GO:0032056	1	0.997989038	0	1
6981	GO:0032057	1	0.992031484	0	4
6982	GO:0032058	1	0.997984742	0	1
6983	GO:0032059	1	0.982134829	0	9
6984	GO:0032060	1	0.982070187	0	9
6985	GO:0032071	1	0.995977269	0	2
6986	GO:0032072	1	0.998000642	0	1
6987	GO:0032074	1	0.997976841	0	1
6988	GO:0032075	1	0.997969252	0	1
6989	GO:0032076	1	0.997989952	0	1
6990	GO:0032077	1	0.997966828	0	1
6991	GO:0032079	1	0.99197125	0	4
6992	GO:0032088	1	0.861420519	0	74
6993	GO:0032089	1	0.996010026	0	2
6994	GO:0032090	1	0.99596427	0	2
6996	GO:0032092	1	0.870411784	0	69
6997	GO:0032093	1	0.996003809	0	2
6998	GO:0032094	1	0.974263099	0	13
6999	GO:0032095	1	0.991940137	0	4
7000	GO:0032097	1	0.997954504	0	1
7001	GO:0032098	1	0.99194948	0	4
7002	GO:0032099	1	0.985933896	0	7
7003	GO:0032100	1	0.995934881	0	2
7004	GO:0032116	1	0.996007903	0	2
7005	GO:0032119	1	0.99195047	0	4
7006	GO:0032127	1	0.996013982	0	2
7007	GO:0032133	1	0.987950637	0	6
7008	GO:0032135	1	0.998011808	0	1
7009	GO:0032137	1	0.994004799	0	3
7010	GO:0032138	1	0.997991943	0	1
7011	GO:0032139	1	0.993987198	0	3
7012	GO:0032142	1	0.994030367	0	3
7013	GO:0032143	1	0.996010807	0	2
7014	GO:0032144	1	0.998012706	0	1
7015	GO:0032145	1	0.998012706	0	1
7016	GO:0032147	1	0.879533819	0	64
7017	GO:0032148	1	0.935715116	0	33
7018	GO:0032153	1	0.972224266	0	14
7019	GO:0032154	1	0.900799706	0	52
7020	GO:0032156	1	0.99800947	0	1
7021	GO:0032173	1	0.997990564	0	1
7022	GO:0032181	1	0.996012447	0	2
7023	GO:0032182	1	0.99004557	0	5

7024	GO:0032183	1	0.980183589	0	10
7025	GO:0032184	1	0.992034335	0	4
7026	GO:0032185	1	0.99396736	0	3
7027	GO:0032190	1	0.995940565	0	2
7028	GO:0032194	1	0.998003649	0	1
7029	GO:0032196	1	0.998000962	0	1
7030	GO:0032197	1	0.995983434	0	2
7031	GO:0032200	1	0.945217032	0	28
7032	GO:0032201	1	0.933803397	0	34
7033	GO:0032202	1	0.993978758	0	3
7034	GO:0032203	1	0.997976203	0	1
7035	GO:0032204	1	0.974270933	0	13
7036	GO:0032205	1	0.99200932	0	4
7037	GO:0032206	1	0.978207067	0	11
7038	GO:0032208	1	0.997992817	0	1
7039	GO:0032210	1	0.988111082	0	6
7040	GO:0032211	1	0.958708109	0	21
7041	GO:0032212	1	0.933839783	0	34
7042	GO:0032214	1	0.995990119	0	2
7043	GO:0032216	1	0.997982708	0	1
7044	GO:0032217	1	0.989985927	0	5
7045	GO:0032218	1	0.989985927	0	5
7046	GO:0032223	1	0.997987865	0	1
7047	GO:0032224	1	0.998013437	0	1
7048	GO:0032225	1	0.99601155	0	2
7049	GO:0032226	1	0.993953941	0	3
7050	GO:0032227	1	0.996026406	0	2
7051	GO:0032228	1	0.976306806	0	12
7052	GO:0032229	1	0.992008077	0	4
7053	GO:0032230	1	0.980188693	0	10
7054	GO:0032231	1	0.99602846	0	2
7055	GO:0032232	1	0.993972208	0	3
7056	GO:0032233	1	0.976198207	0	12
7057	GO:0032237	1	0.988073838	0	6
7058	GO:0032238	1	0.993969058	0	3
7059	GO:0032243	1	0.997976058	0	1
7060	GO:0032244	1	0.997991904	0	1
7061	GO:0032252	1	0.994043763	0	3
7063	GO:0032261	1	0.997970558	0	1
7064	GO:0032263	1	0.997968322	0	1
7065	GO:0032264	1	0.991992221	0	4
7066	GO:0032266	1	0.915491727	0	44
7067	GO:0032268	1	0.990003007	0	5
7068	GO:0032269	1	0.989986912	0	5
7069	GO:0032270	1	0.974133928	0	13

7070	GO:0032272	1	0.997969972	0	1
7071	GO:0032273	1	0.989987764	0	5
7072	GO:0032274	1	0.997990564	0	1
7073	GO:0032275	1	0.994020235	0	3
7074	GO:0032277	1	0.997996294	0	1
7075	GO:0032279	1	0.974241737	0	13
7076	GO:0032280	1	0.99602906	0	2
7077	GO:0032281	1	0.964364759	0	18
7078	GO:0032286	1	0.992004992	0	4
7079	GO:0032287	1	0.984098458	0	8
7080	GO:0032288	1	0.984112224	0	8
7081	GO:0032289	1	0.990017267	0	5
7082	GO:0032290	1	0.99402909	0	3
7083	GO:0032298	1	0.997992461	0	1
7084	GO:0032299	1	0.99393738	0	3
7085	GO:0032300	1	0.986092395	0	7
7086	GO:0032301	1	0.994009206	0	3
7087	GO:0032302	1	0.996012447	0	2
7088	GO:0032304	1	0.997984552	0	1
7089	GO:0032305	1	0.995976031	0	2
7090	GO:0032307	1	0.99600765	0	2
7091	GO:0032308	1	0.983933335	0	8
7092	GO:0032310	1	0.994032818	0	3
7093	GO:0032311	1	0.995940732	0	2
7094	GO:0032324	1	0.988007155	0	6
7095	GO:0032328	1	0.990102869	0	5
7096	GO:0032329	1	0.997978603	0	1
7097	GO:0032330	1	0.978141564	0	11
7098	GO:0032331	1	0.962587332	0	19
7099	GO:0032332	1	0.966483085	0	17
7100	GO:0032341	1	0.997988246	0	1
7101	GO:0032342	1	0.998013437	0	1
7102	GO:0032347	1	0.997997218	0	1
7103	GO:0032348	1	0.994002632	0	3
7104	GO:0032349	1	0.996006481	0	2
7105	GO:0032350	1	0.993993374	0	3
7106	GO:0032354	1	0.992017124	0	4
7107	GO:0032355	1	0.815785735	0	101
7108	GO:0032356	1	0.99798048	0	1
7109	GO:0032357	1	0.987982193	0	6
7110	GO:0032358	1	0.997957079	0	1
7111	GO:0032361	1	0.997978636	0	1
7112	GO:0032364	1	0.986042051	0	7
7113	GO:0032365	1	0.997962172	0	1
7114	GO:0032366	1	0.995930973	0	2

7116	GO:0032368	1	0.994006307	0	3
7117	GO:0032369	1	0.992000402	0	4
7118	GO:0032370	1	0.996029922	0	2
7119	GO:0032374	1	0.989970781	0	5
7120	GO:0032375	1	0.993924476	0	3
7121	GO:0032376	1	0.988013374	0	6
7122	GO:0032379	1	0.998013437	0	1
7123	GO:0032383	1	0.994006292	0	3
7124	GO:0032384	1	0.998013437	0	1
7125	GO:0032385	1	0.997968397	0	1
7126	GO:0032386	1	0.991981002	0	4
7127	GO:0032387	1	0.995971423	0	2
7128	GO:0032388	1	0.988061013	0	6
7129	GO:0032389	1	0.995982035	0	2
7130	GO:0032391	1	0.926572734	0	38
7131	GO:0032392	1	0.991986512	0	4
7132	GO:0032393	1	0.991917683	0	4
7133	GO:0032394	1	0.995967216	0	2
7134	GO:0032395	1	0.981876303	0	9
7135	GO:0032397	1	0.997970959	0	1
7136	GO:0032398	1	0.995942956	0	2
7137	GO:0032400	1	0.989979238	0	5
7138	GO:0032401	1	0.995994687	0	2
7139	GO:0032402	1	0.960483344	0	20
7140	GO:0032405	1	0.98803197	0	6
7141	GO:0032406	1	0.995994401	0	2
7142	GO:0032407	1	0.986013211	0	7
7143	GO:0032408	1	0.995994401	0	2
7144	GO:0032410	1	0.985994715	0	7
7145	GO:0032411	1	0.987979132	0	6
7146	GO:0032412	1	0.996002188	0	2
7147	GO:0032414	1	0.990025906	0	5
7148	GO:0032415	1	0.99797867	0	1
7149	GO:0032416	1	0.99797867	0	1
7150	GO:0032417	1	0.991948987	0	4
7151	GO:0032418	1	0.941455519	0	30
7152	GO:0032419	1	0.997982404	0	1
7153	GO:0032420	1	0.920891566	0	41
7154	GO:0032421	1	0.980230247	0	10
7155	GO:0032422	1	0.993994629	0	3
7156	GO:0032423	1	0.998009551	0	1
7157	GO:0032425	1	0.993998492	0	3
7158	GO:0032426	1	0.964530793	0	18
7159	GO:0032427	1	0.995996654	0	2
7160	GO:0032428	1	0.998002752	0	1

7161	GO:0032429	1	0.99600165	0	2
7162	GO:0032430	1	0.998012806	0	1
7163	GO:0032431	1	0.997959823	0	1
7164	GO:0032432	1	0.980130332	0	10
7165	GO:0032433	1	0.96658335	0	17
7166	GO:0032434	1	0.972251755	0	14
7167	GO:0032435	1	0.94317023	0	29
7168	GO:0032436	1	0.861617799	0	74
7169	GO:0032437	1	0.986026784	0	7
7170	GO:0032438	1	0.956671898	0	22
7171	GO:0032440	1	0.997965198	0	1
7172	GO:0032444	1	0.994020096	0	3
7173	GO:0032446	1	0.978113619	0	11
7174	GO:0032447	1	0.991934393	0	4
7175	GO:0032449	1	0.992049392	0	4
7176	GO:0032450	1	0.994039789	0	3
7177	GO:0032451	1	0.988014127	0	6
7178	GO:0032452	1	0.968591668	0	16
7179	GO:0032453	1	0.988075418	0	6
7180	GO:0032454	1	0.976348115	0	12
7181	GO:0032455	1	0.991992771	0	4
7182	GO:0032456	1	0.900826608	0	52
7183	GO:0032458	1	0.998006287	0	1
7184	GO:0032461	1	0.997978835	0	1
7185	GO:0032463	1	0.99800714	0	1
7186	GO:0032464	1	0.995990505	0	2
7187	GO:0032465	1	0.91908354	0	42
7188	GO:0032466	1	0.986018798	0	7
7189	GO:0032467	1	0.941576246	0	30
7190	GO:0032468	1	0.995983673	0	2
7191	GO:0032469	1	0.96646189	0	17
7192	GO:0032470	1	0.994031	0	3
7193	GO:0032471	1	0.981991897	0	9
7194	GO:0032472	1	0.995983673	0	2
7195	GO:0032473	1	0.995997152	0	2
7196	GO:0032474	1	0.994042624	0	3
7197	GO:0032475	1	0.997988914	0	1
7201	GO:0032482	1	0.974093461	0	13
7202	GO:0032483	1	0.986145731	0	7
7203	GO:0032485	1	0.994036882	0	3
7204	GO:0032486	1	0.980188055	0	10
7205	GO:0032487	1	0.99206488	0	4
7206	GO:0032488	1	0.986004542	0	7
7207	GO:0032489	1	0.98795682	0	6
7208	GO:0032490	1	0.998013388	0	1

7209	GO:0032491	1	0.997985434	0	1
7210	GO:0032493	1	0.99401744	0	3
7211	GO:0032494	1	0.984033114	0	8
7212	GO:0032495	1	0.976239214	0	12
7214	GO:0032497	1	0.991940108	0	4
7215	GO:0032498	1	0.998011136	0	1
7216	GO:0032499	1	0.997961288	0	1
7217	GO:0032500	1	0.998011136	0	1
7218	GO:0032502	1	0.970216111	0	15
7219	GO:0032506	1	0.994025596	0	3
7220	GO:0032507	1	0.991965696	0	4
7221	GO:0032508	1	0.850009099	0	81
7222	GO:0032509	1	0.978066441	0	11
7223	GO:0032510	1	0.990025817	0	5
7224	GO:0032511	1	0.978031104	0	11
7225	GO:0032515	1	0.905885456	0	49
7226	GO:0032516	1	0.974282984	0	13
7227	GO:0032525	1	0.980033927	0	10
7228	GO:0032526	1	0.915250874	0	44
7229	GO:0032527	1	0.985964656	0	7
7230	GO:0032528	1	0.996030334	0	2
7231	GO:0032532	1	0.988022899	0	6
7232	GO:0032534	1	0.990069549	0	5
7233	GO:0032535	1	0.998013415	0	1
7234	GO:0032536	1	0.998006669	0	1
7235	GO:0032541	1	0.984066584	0	8
7236	GO:0032542	1	0.99599303	0	2
7237	GO:0032543	1	0.927590249	0	37
7238	GO:0032545	1	0.99597746	0	2
7239	GO:0032549	1	0.994039428	0	3
7240	GO:0032556	1	0.997962564	0	1
7241	GO:0032557	1	0.997980968	0	1
7242	GO:0032558	1	0.997972608	0	1
7243	GO:0032559	1	0.993972913	0	3
7244	GO:0032561	1	0.997981381	0	1
7245	GO:0032564	1	0.993987098	0	3
7246	GO:0032567	1	0.997997404	0	1
7247	GO:0032570	1	0.933790632	0	34
7248	GO:0032571	1	0.99795081	0	1
7249	GO:0032574	1	0.996014027	0	2
7250	GO:0032580	1	0.844603718	0	84
7251	GO:0032581	1	0.997975998	0	1
7252	GO:0032584	1	0.988044146	0	6
7253	GO:0032585	1	0.972210212	0	14
7255	GO:0032588	1	0.82417074	0	96

7256	GO:0032589	1	0.998012802	0	1
7257	GO:0032590	1	0.949124542	0	26
7258	GO:0032591	1	0.9723884	0	14
7259	GO:0032592	1	0.980083652	0	10
7260	GO:0032593	1	0.982085902	0	9
7261	GO:0032596	1	0.995978692	0	2
7262	GO:0032597	1	0.997981486	0	1
7263	GO:0032600	1	0.997981486	0	1
7264	GO:0032609	1	0.998013437	0	1
7265	GO:0032613	1	0.998000278	0	1
7266	GO:0032640	1	0.998006178	0	1
7267	GO:0032642	1	0.993962874	0	3
7268	GO:0032648	1	0.993986786	0	3
7270	GO:0032651	1	0.984073203	0	8
7271	GO:0032652	1	0.997968136	0	1
7272	GO:0032653	1	0.995980274	0	2
7273	GO:0032655	1	0.992023909	0	4
7274	GO:0032661	1	0.997997982	0	1
7275	GO:0032663	1	0.997978212	0	1
7276	GO:0032667	1	0.997981889	0	1
7277	GO:0032673	1	0.997963013	0	1
7278	GO:0032674	1	0.997978968	0	1
7279	GO:0032675	1	0.97811145	0	11
7280	GO:0032677	1	0.994027892	0	3
7281	GO:0032680	1	0.97619851	0	12
7282	GO:0032682	1	0.983991715	0	8
7283	GO:0032683	1	0.998009529	0	1
7284	GO:0032685	1	0.997998379	0	1
7285	GO:0032686	1	0.997997404	0	1
7286	GO:0032687	1	0.989991822	0	5
7287	GO:0032688	1	0.974178897	0	13
7288	GO:0032689	1	0.924195271	0	39
7289	GO:0032690	1	0.995970999	0	2
7290	GO:0032691	1	0.94882964	0	26
7291	GO:0032692	1	0.989990934	0	5
7292	GO:0032693	1	0.970220941	0	15
7293	GO:0032695	1	0.972233089	0	14
7294	GO:0032696	1	0.989933175	0	5
7295	GO:0032700	1	0.974142546	0	13
7296	GO:0032701	1	0.993993512	0	3
7298	GO:0032707	1	0.996001927	0	2
7299	GO:0032712	1	0.997983824	0	1
7300	GO:0032713	1	0.985963223	0	7
7301	GO:0032714	1	0.989957441	0	5
7302	GO:0032715	1	0.922671961	0	40

7303	GO:0032717	1	0.96440349	0	18
7304	GO:0032720	1	0.90411894	0	50
7305	GO:0032722	1	0.928205675	0	37
7306	GO:0032723	1	0.998013437	0	1
7307	GO:0032724	1	0.995935437	0	2
7312	GO:0032730	1	0.987946591	0	6
7313	GO:0032731	1	0.907867487	0	48
7314	GO:0032732	1	0.982079918	0	9
7316	GO:0032735	1	0.933808595	0	34
7317	GO:0032736	1	0.976133387	0	12
7318	GO:0032738	1	0.997974939	0	1
7319	GO:0032740	1	0.968235349	0	16
7320	GO:0032741	1	0.990032732	0	5
7321	GO:0032743	1	0.941495139	0	30
7322	GO:0032745	1	0.995970372	0	2
7323	GO:0032747	1	0.993958254	0	3
7324	GO:0032752	1	0.998012951	0	1
7325	GO:0032753	1	0.956672592	0	22
7326	GO:0032754	1	0.982049329	0	9
7329	GO:0032759	1	0.997973097	0	1
7331	GO:0032761	1	0.995989829	0	2
7332	GO:0032764	1	0.995976883	0	2
7333	GO:0032765	1	0.994034025	0	3
7334	GO:0032767	1	0.993979941	0	3
7335	GO:0032769	1	0.995942612	0	2
7336	GO:0032770	1	0.995944581	0	2
7337	GO:0032773	1	0.996027212	0	2
7338	GO:0032774	1	0.993934012	0	3
7339	GO:0032775	1	0.996020318	0	2
7340	GO:0032776	1	0.996024394	0	2
7341	GO:0032777	1	0.991976452	0	4
7342	GO:0032780	1	0.978022085	0	11
7343	GO:0032781	1	0.927879541	0	37
7344	GO:0032782	1	0.988022992	0	6
7345	GO:0032783	1	0.990051025	0	5
7346	GO:0032784	1	0.982069443	0	9
7347	GO:0032785	1	0.995978233	0	2
7348	GO:0032786	1	0.970287116	0	15
7349	GO:0032787	1	0.995976617	0	2
7350	GO:0032788	1	0.997972187	0	1
7351	GO:0032789	1	0.997972187	0	1
7352	GO:0032790	1	0.984024245	0	8
7353	GO:0032791	1	0.995960407	0	2
7354	GO:0032792	1	0.989997888	0	5
7355	GO:0032793	1	0.964537594	0	18

7356	GO:0032794	1	0.970428031	0	15
7357	GO:0032795	1	0.9939718	0	3
7358	GO:0032796	1	0.995989508	0	2
7359	GO:0032797	1	0.97807851	0	11
7360	GO:0032798	1	0.997990564	0	1
7361	GO:0032799	1	0.998003561	0	1
7362	GO:0032801	1	0.976243661	0	12
7363	GO:0032802	1	0.980116257	0	10
7364	GO:0032803	1	0.996002932	0	2
7365	GO:0032804	1	0.995985516	0	2
7366	GO:0032805	1	0.993992564	0	3
7367	GO:0032807	1	0.993972939	0	3
7368	GO:0032808	1	0.990081723	0	5
7369	GO:0032809	1	0.958758341	0	21
7370	GO:0032810	1	0.99598772	0	2
7371	GO:0032811	1	0.993994068	0	3
7372	GO:0032813	1	0.989947812	0	5
7373	GO:0032814	1	0.995987498	0	2
7374	GO:0032815	1	0.987940461	0	6
7375	GO:0032816	1	0.9879704	0	6
7376	GO:0032817	1	0.995999064	0	2
7377	GO:0032819	1	0.987935286	0	6
7378	GO:0032823	1	0.99598927	0	2
7379	GO:0032825	1	0.984097702	0	8
7380	GO:0032826	1	0.997972626	0	1
7381	GO:0032827	1	0.995944309	0	2
7382	GO:0032831	1	0.993924268	0	3
7383	GO:0032834	1	0.997972973	0	1
7384	GO:0032835	1	0.988119586	0	6
7385	GO:0032836	1	0.982188162	0	9
7386	GO:0032838	1	0.9959301	0	2
7387	GO:0032839	1	0.937817263	0	32
7388	GO:0032841	1	0.998003243	0	1
7389	GO:0032848	1	0.998005976	0	1
7390	GO:0032849	1	0.995985744	0	2
7391	GO:0032868	1	0.87747164	0	65
7393	GO:0032870	1	0.949078443	0	26
7394	GO:0032872	1	0.983977497	0	8
7395	GO:0032873	1	0.982006578	0	9
7396	GO:0032874	1	0.95878298	0	21
7397	GO:0032876	1	0.997981405	0	1
7398	GO:0032877	1	0.996021835	0	2
7399	GO:0032878	1	0.994035528	0	3
7400	GO:0032880	1	0.880993411	0	63
7401	GO:0032886	1	0.980221556	0	10

7402	GO:0032889	1	0.991945513	0	4
7404	GO:0032897	1	0.968367804	0	16
7405	GO:0032900	1	0.997991904	0	1
7406	GO:0032901	1	0.998006426	0	1
7407	GO:0032902	1	0.996012026	0	2
7408	GO:0032909	1	0.992070824	0	4
7409	GO:0032911	1	0.990035589	0	5
7410	GO:0032912	1	0.996015431	0	2
7411	GO:0032913	1	0.997981486	0	1
7412	GO:0032914	1	0.987995058	0	6
7413	GO:0032915	1	0.993973089	0	3
7414	GO:0032916	1	0.996030731	0	2
7415	GO:0032918	1	0.99592624	0	2
7416	GO:0032919	1	0.997959564	0	1
7417	GO:0032920	1	0.997959564	0	1
7418	GO:0032922	1	0.881213972	0	63
7419	GO:0032924	1	0.974329761	0	13
7420	GO:0032925	1	0.993963857	0	3
7421	GO:0032926	1	0.986078063	0	7
7422	GO:0032927	1	0.990002425	0	5
7423	GO:0032928	1	0.998012951	0	1
7424	GO:0032929	1	0.99193269	0	4
7425	GO:0032930	1	0.966313182	0	17
7426	GO:0032933	1	0.98214134	0	9
7427	GO:0032934	1	0.970429766	0	15
7428	GO:0032937	1	0.993999758	0	3
7429	GO:0032938	1	0.997993296	0	1
7430	GO:0032940	1	0.980093091	0	10
7431	GO:0032943	1	0.995985623	0	2
7432	GO:0032945	1	0.997992275	0	1
7433	GO:0032946	1	0.995958226	0	2
7434	GO:0032956	1	0.861745959	0	74
7435	GO:0032957	1	0.993998553	0	3
7436	GO:0032958	1	0.978195742	0	11
7437	GO:0032959	1	0.993998768	0	3
7438	GO:0032960	1	0.998010325	0	1
7439	GO:0032962	1	0.991994997	0	4
7440	GO:0032963	1	0.978181201	0	11
7441	GO:0032964	1	0.984102081	0	8
7442	GO:0032966	1	0.978064955	0	11
7443	GO:0032967	1	0.95283162	0	24
7444	GO:0032968	1	0.976287626	0	12
7445	GO:0032970	1	0.995979578	0	2
7446	GO:0032971	1	0.998009617	0	1
7447	GO:0032972	1	0.995921204	0	2

7448	GO:0032976	1	0.997956473	0	1
7449	GO:0032977	1	0.972176955	0	14
7450	GO:0032979	1	0.991970468	0	4
7451	GO:0032980	1	0.997985324	0	1
7452	GO:0032981	1	0.874305649	0	66
7453	GO:0032982	1	0.980250113	0	10
7454	GO:0032983	1	0.994018557	0	3
7455	GO:0032984	1	0.993970936	0	3
7456	GO:0032986	1	0.995940209	0	2
7457	GO:0032990	1	0.997990564	0	1
7459	GO:0032993	1	0.926376003	0	38
7460	GO:0032994	1	0.995949936	0	2
7461	GO:0032996	1	0.997976366	0	1
7462	GO:0032998	1	0.995941331	0	2
7463	GO:0033002	1	0.998013437	0	1
7464	GO:0033003	1	0.995981397	0	2
7465	GO:0033004	1	0.989991011	0	5
7466	GO:0033005	1	0.989930468	0	5
7467	GO:0033007	1	0.995994928	0	2
7468	GO:0033008	1	0.99799382	0	1
7469	GO:0033010	1	0.984061451	0	8
7470	GO:0033011	1	0.997983713	0	1
7471	GO:0033013	1	0.997994159	0	1
7472	GO:0033014	1	0.991941746	0	4
7474	GO:0033018	1	0.982034761	0	9
7475	GO:0033023	1	0.99799116	0	1
7476	GO:0033025	1	0.998002555	0	1
7477	GO:0033026	1	0.998013356	0	1
7478	GO:0033028	1	0.998006917	0	1
7479	GO:0033029	1	0.998013437	0	1
7480	GO:0033030	1	0.996008057	0	2
7481	GO:0033031	1	0.989980476	0	5
7482	GO:0033032	1	0.996002706	0	2
7483	GO:0033033	1	0.993962883	0	3
7484	GO:0033034	1	0.998011533	0	1
7485	GO:0033037	1	0.998013436	0	1
7486	GO:0033038	1	0.971841649	0	14
7487	GO:0033040	1	0.998013216	0	1
7488	GO:0033041	1	0.997987546	0	1
7489	GO:0033043	1	0.995985075	0	2
7490	GO:0033044	1	0.993978178	0	3
7491	GO:0033045	1	0.997990564	0	1
7492	GO:0033058	1	0.994025476	0	3
7493	GO:0033059	1	0.997965514	0	1
7494	GO:0033063	1	0.991959307	0	4

7495	GO:0033065	1	0.995951296	0	2
7496	GO:0033076	1	0.995954363	0	2
7497	GO:0033077	1	0.937694584	0	32
7498	GO:0033080	1	0.998006585	0	1
7499	GO:0033081	1	0.986038464	0	7
7500	GO:0033082	1	0.99598927	0	2
7501	GO:0033085	1	0.99193916	0	4
7502	GO:0033087	1	0.997973681	0	1
7503	GO:0033088	1	0.989960429	0	5
7504	GO:0033089	1	0.980102796	0	10
7505	GO:0033091	1	0.997988028	0	1
7506	GO:0033092	1	0.989960655	0	5
7507	GO:0033093	1	0.988057857	0	6
7508	GO:0033106	1	0.985946013	0	7
7509	GO:0033108	1	0.987911674	0	6
7510	GO:0033116	1	0.866718895	0	71
7511	GO:0033119	1	0.98794972	0	6
7512	GO:0033120	1	0.972345096	0	14
7513	GO:0033127	1	0.995986713	0	2
7514	GO:0033128	1	0.99598318	0	2
7515	GO:0033129	1	0.984038996	0	8
7516	GO:0033130	1	0.976137141	0	12
7517	GO:0033132	1	0.991967479	0	4
7518	GO:0033133	1	0.989967807	0	5
7519	GO:0033135	1	0.986140788	0	7
7520	GO:0033137	1	0.951004694	0	25
7521	GO:0033138	1	0.847774176	0	82
7522	GO:0033140	1	0.993946466	0	3
7523	GO:0033141	1	0.989998417	0	5
7524	GO:0033142	1	0.994020289	0	3
7525	GO:0033144	1	0.993958084	0	3
7526	GO:0033146	1	0.970345979	0	15
7527	GO:0033147	1	0.97037127	0	15
7528	GO:0033148	1	0.978161	0	11
7529	GO:0033149	1	0.993992156	0	3
7530	GO:0033150	1	0.99797755	0	1
7531	GO:0033151	1	0.984122571	0	8
7532	GO:0033152	1	0.992027456	0	4
7533	GO:0033153	1	0.990067392	0	5
7534	GO:0033157	1	0.988059546	0	6
7535	GO:0033162	1	0.966313232	0	17
7536	GO:0033165	1	0.992025181	0	4
7537	GO:0033167	1	0.998013437	0	1
7538	GO:0033168	1	0.998013437	0	1
7539	GO:0033169	1	0.974382344	0	13

7540	GO:0033173	1	0.97821516	0	11
7541	GO:0033176	1	0.997972973	0	1
7542	GO:0033177	1	0.989933855	0	5
7543	GO:0033178	1	0.9959491	0	2
7544	GO:0033179	1	0.980010936	0	10
7545	GO:0033180	1	0.986011714	0	7
7546	GO:0033181	1	0.993949902	0	3
7547	GO:0033182	1	0.995950351	0	2
7548	GO:0033184	1	0.997995688	0	1
7549	GO:0033185	1	0.993893164	0	3
7550	GO:0033186	1	0.994008063	0	3
7551	GO:0033188	1	0.994000085	0	3
7552	GO:0033189	1	0.968365094	0	16
7553	GO:0033192	1	0.990055857	0	5
7554	GO:0033193	1	0.998012887	0	1
7555	GO:0033194	1	0.984038706	0	8
7556	GO:0033197	1	0.982036511	0	9
7557	GO:0033198	1	0.970304081	0	15
7558	GO:0033204	1	0.979954118	0	10
7559	GO:0033206	1	0.996000801	0	2
7561	GO:0033210	1	0.982026881	0	9
7562	GO:0033211	1	0.986052659	0	7
7563	GO:0033212	1	0.994043598	0	3
7564	GO:0033214	1	0.997984615	0	1
7565	GO:0033215	1	0.996001671	0	2
7566	GO:0033218	1	0.995955484	0	2
7567	GO:0033227	1	0.993978297	0	3
7568	GO:0033229	1	0.994020309	0	3
7569	GO:0033233	1	0.996013727	0	2
7570	GO:0033234	1	0.984039858	0	8
7571	GO:0033235	1	0.97414758	0	13
7572	GO:0033256	1	0.99598352	0	2
7573	GO:0033257	1	0.995975714	0	2
7574	GO:0033260	1	0.995958052	0	2
7575	GO:0033262	1	0.989988749	0	5
7576	GO:0033263	1	0.988047305	0	6
7577	GO:0033265	1	0.99798965	0	1
7578	GO:0033268	1	0.972426603	0	14
7579	GO:0033269	1	0.992010073	0	4
7580	GO:0033270	1	0.978225833	0	11
7581	GO:0033273	1	0.992003851	0	4
7582	GO:0033274	1	0.998013437	0	1
7583	GO:0033276	1	0.972199048	0	14
7584	GO:0033277	1	0.996000142	0	2
7585	GO:0033278	1	0.992022053	0	4

7587	GO:0033290	1	0.970123052	0	15
7588	GO:0033291	1	0.997947485	0	1
7589	GO:0033292	1	0.984129241	0	8
7590	GO:0033299	1	0.989999352	0	5
7591	GO:0033300	1	0.99000367	0	5
7592	GO:0033301	1	0.998004524	0	1
7593	GO:0033306	1	0.995985558	0	2
7594	GO:0033313	1	0.997995399	0	1
7595	GO:0033314	1	0.98006022	0	10
7596	GO:0033316	1	0.997994857	0	1
7597	GO:0033320	1	0.997980136	0	1
7598	GO:0033326	1	0.994028362	0	3
7599	GO:0033327	1	0.982120418	0	9
7600	GO:0033328	1	0.996004253	0	2
7601	GO:0033342	1	0.997987016	0	1
7602	GO:0033343	1	0.998013437	0	1
7603	GO:0033344	1	0.962451491	0	19
7604	GO:0033345	1	0.997984749	0	1
7605	GO:0033353	1	0.992026402	0	4
7606	GO:0033363	1	0.993973328	0	3
7607	GO:0033364	1	0.995982547	0	2
7608	GO:0033365	1	0.954980108	0	23
7609	GO:0033366	1	0.995981999	0	2
7610	GO:0033371	1	0.99796516	0	1
7611	GO:0033373	1	0.99796516	0	1
7612	GO:0033382	1	0.99796516	0	1
7613	GO:0033384	1	0.995965062	0	2
7614	GO:0033386	1	0.997991455	0	1
7615	GO:0033387	1	0.993991979	0	3
7616	GO:0033388	1	0.997981841	0	1
7617	GO:0033389	1	0.997981841	0	1
7618	GO:0033391	1	0.974316601	0	13
7619	GO:0033396	1	0.997979776	0	1
7620	GO:0033477	1	0.997978503	0	1
7621	GO:0033484	1	0.996004955	0	2
7622	GO:0033488	1	0.997995521	0	1
7625	GO:0033499	1	0.993937145	0	3
7626	GO:0033500	1	0.995981907	0	2
7627	GO:0033503	1	0.992004061	0	4
7628	GO:0033504	1	0.988012144	0	6
7629	GO:0033512	1	0.993999562	0	3
7630	GO:0033514	1	0.997985293	0	1
7631	GO:0033522	1	0.988046278	0	6
7632	GO:0033523	1	0.98213801	0	9
7633	GO:0033539	1	0.980013742	0	10

7634	GO:0033540	1	0.970223887	0	15
7635	GO:0033549	1	0.995995108	0	2
7636	GO:0033553	1	0.990012349	0	5
7637	GO:0033555	1	0.988114108	0	6
7638	GO:0033557	1	0.993983524	0	3
7639	GO:0033558	1	0.976228808	0	12
7640	GO:0033559	1	0.99798452	0	1
7641	GO:0033561	1	0.995999164	0	2
7642	GO:0033563	1	0.9939998	0	3
7643	GO:0033564	1	0.992066688	0	4
7644	GO:0033565	1	0.994012362	0	3
7645	GO:0033566	1	0.995979877	0	2
7646	GO:0033567	1	0.998010808	0	1
7647	GO:0033572	1	0.929927727	0	36
7648	GO:0033574	1	0.943134738	0	29
7649	GO:0033577	1	0.995965679	0	2
7650	GO:0033578	1	0.99799873	0	1
7651	GO:0033580	1	0.99797447	0	1
7652	GO:0033588	1	0.985983759	0	7
7653	GO:0033590	1	0.991961275	0	4
7654	GO:0033591	1	0.989953345	0	5
7655	GO:0033592	1	0.990048385	0	5
7656	GO:0033593	1	0.998013437	0	1
7657	GO:0033594	1	0.995942433	0	2
7658	GO:0033596	1	0.996030719	0	2
7659	GO:0033597	1	0.997981122	0	1
7660	GO:0033598	1	0.982122367	0	9
7661	GO:0033599	1	0.991928644	0	4
7662	GO:0033600	1	0.992014371	0	4
7663	GO:0033601	1	0.984121264	0	8
7664	GO:0033602	1	0.992051716	0	4
7665	GO:0033603	1	0.990020759	0	5
7666	GO:0033604	1	0.997979332	0	1
7667	GO:0033605	1	0.991988613	0	4
7668	GO:0033609	1	0.997990564	0	1
7669	GO:0033612	1	0.9920006	0	4
7670	GO:0033613	1	0.935962844	0	33
7671	GO:0033615	1	0.987992638	0	6
7672	GO:0033617	1	0.95651756	0	22
7673	GO:0033619	1	0.97806315	0	11
7674	GO:0033622	1	0.984101464	0	8
7675	GO:0033623	1	0.994007559	0	3
7676	GO:0033624	1	0.994029449	0	3
7677	GO:0033625	1	0.982059778	0	9
7678	GO:0033626	1	0.998004409	0	1

7679	GO:0033627	1	0.958875097	0	21
7680	GO:0033628	1	0.976166369	0	12
7681	GO:0033629	1	0.980117512	0	10
7682	GO:0033630	1	0.974214395	0	13
7683	GO:0033631	1	0.990066899	0	5
7684	GO:0033632	1	0.993976667	0	3
7685	GO:0033633	1	0.996029831	0	2
7686	GO:0033634	1	0.987934137	0	6
7687	GO:0033668	1	0.988051248	0	6
7688	GO:0033673	1	0.972214478	0	14
7689	GO:0033674	1	0.872736461	0	68
7690	GO:0033677	1	0.995985747	0	2
7691	GO:0033678	1	0.997989177	0	1
7692	GO:0033679	1	0.998011759	0	1
7693	GO:0033683	1	0.92622203	0	38
7694	GO:0033685	1	0.996002019	0	2
7695	GO:0033686	1	0.993980398	0	3
7696	GO:0033687	1	0.989987771	0	5
7697	GO:0033688	1	0.993986404	0	3
7698	GO:0033689	1	0.982198553	0	9
7699	GO:0033690	1	0.980111828	0	10
7700	GO:0033691	1	0.974213711	0	13
7701	GO:0033692	1	0.994029461	0	3
7702	GO:0033693	1	0.994011684	0	3
7703	GO:0033699	1	0.997978951	0	1
7704	GO:0033700	1	0.983971313	0	8
7705	GO:0033703	1	0.997967667	0	1
7706	GO:0033721	1	0.997991563	0	1
7707	GO:0033737	1	0.997986719	0	1
7708	GO:0033743	1	0.991966379	0	4
7709	GO:0033744	1	0.997969548	0	1
7710	GO:0033745	1	0.993987652	0	3
7711	GO:0033746	1	0.998003307	0	1
7712	GO:0033749	1	0.998003307	0	1
7713	GO:0033754	1	0.995958649	0	2
7714	GO:0033762	1	0.980120147	0	10
7715	GO:0033779	1	0.998007019	0	1
7716	GO:0033781	1	0.997980022	0	1
7717	GO:0033783	1	0.997984994	0	1
7718	GO:0033787	1	0.997992012	0	1
7719	GO:0033791	1	0.99798376	0	1
7720	GO:0033798	1	0.998013437	0	1
7721	GO:0033814	1	0.997968397	0	1
7722	GO:0033819	1	0.997959405	0	1
7723	GO:0033823	1	0.997994221	0	1

7724	GO:0033829	1	0.993948981	0	3
7725	GO:0033842	1	0.9960062	0	2
7726	GO:0033857	1	0.996030582	0	2
7727	GO:0033858	1	0.997996949	0	1
7728	GO:0033861	1	0.997984599	0	1
7730	GO:0033864	1	0.987943894	0	6
7731	GO:0033867	1	0.997972492	0	1
7732	GO:0033871	1	0.997977718	0	1
7733	GO:0033872	1	0.99597006	0	2
7734	GO:0033878	1	0.996022876	0	2
7735	GO:0033882	1	0.995968025	0	2
7736	GO:0033883	1	0.995945342	0	2
7737	GO:0033885	1	0.997982002	0	1
7738	GO:0033897	1	0.99796516	0	1
7739	GO:0033906	1	0.995963616	0	2
7740	GO:0033919	1	0.998006617	0	1
7741	GO:0033925	1	0.998011162	0	1
7742	GO:0033961	1	0.997974321	0	1
7743	GO:0033962	1	0.97231302	0	14
7744	GO:0033979	1	0.995998778	0	2
7745	GO:0033981	1	0.997953073	0	1
7746	GO:0033989	1	0.997989596	0	1
7747	GO:0033993	1	0.974207818	0	13
7748	GO:0034012	1	0.997990564	0	1
7749	GO:0034021	1	0.993877346	0	3
7750	GO:0034038	1	0.997963945	0	1
7751	GO:0034039	1	0.995957137	0	2
7752	GO:0034040	1	0.996030731	0	2
7753	GO:0034041	1	0.997994151	0	1
7754	GO:0034045	1	0.970391347	0	15
7755	GO:0034046	1	0.984115877	0	8
7756	GO:0034054	1	0.997982404	0	1
7757	GO:0034056	1	0.99401832	0	3
7758	GO:0034057	1	0.995998729	0	2
7759	GO:0034058	1	0.984146266	0	8
7760	GO:0034059	1	0.996004841	0	2
7761	GO:0034061	1	0.993954146	0	3
7762	GO:0034062	1	0.997990564	0	1
7763	GO:0034063	1	0.955039594	0	23
7764	GO:0034066	1	0.995960674	0	2
7765	GO:0034067	1	0.962671488	0	19
7766	GO:0034080	1	0.916903493	0	43
7767	GO:0034085	1	0.998007009	0	1
7768	GO:0034087	1	0.996020014	0	2
7769	GO:0034088	1	0.992022818	0	4

7771	GO:0034098	1	0.98410742	0	8
7772	GO:0034101	1	0.979989948	0	10
7773	GO:0034103	1	0.989969299	0	5
7774	GO:0034104	1	0.997987857	0	1
7775	GO:0034105	1	0.99390997	0	3
7776	GO:0034109	1	0.984084425	0	8
7777	GO:0034111	1	0.99599649	0	2
7778	GO:0034112	1	0.989982477	0	5
7779	GO:0034113	1	0.951094329	0	25
7780	GO:0034115	1	0.980056913	0	10
7781	GO:0034116	1	0.97214105	0	14
7782	GO:0034120	1	0.997951264	0	1
7783	GO:0034121	1	0.988066504	0	6
7784	GO:0034122	1	0.972302798	0	14
7785	GO:0034123	1	0.992034568	0	4
7787	GO:0034125	1	0.997974522	0	1
7789	GO:0034128	1	0.986011849	0	7
7790	GO:0034130	1	0.997992028	0	1
7791	GO:0034134	1	0.990017261	0	5
7792	GO:0034136	1	0.990082673	0	5
7793	GO:0034137	1	0.986022488	0	7
7794	GO:0034138	1	0.976113105	0	12
7795	GO:0034140	1	0.996007223	0	2
7796	GO:0034141	1	0.986026331	0	7
7797	GO:0034142	1	0.966328789	0	17
7798	GO:0034143	1	0.996015409	0	2
7799	GO:0034144	1	0.980037316	0	10
7800	GO:0034145	1	0.98007063	0	10
7801	GO:0034146	1	0.99800978	0	1
7802	GO:0034148	1	0.998010813	0	1
7803	GO:0034150	1	0.998013437	0	1
7804	GO:0034154	1	0.990022653	0	5
7806	GO:0034161	1	0.996008423	0	2
7807	GO:0034162	1	0.972317293	0	14
7808	GO:0034163	1	0.998006178	0	1
7809	GO:0034164	1	0.994025851	0	3
7811	GO:0034166	1	0.99799902	0	1
7812	GO:0034184	1	0.989989027	0	5
7813	GO:0034185	1	0.968473642	0	16
7814	GO:0034186	1	0.989954781	0	5
7815	GO:0034188	1	0.996030731	0	2
7816	GO:0034189	1	0.991964799	0	4
7817	GO:0034190	1	0.99596575	0	2
7818	GO:0034191	1	0.991956595	0	4
7819	GO:0034197	1	0.989954299	0	5

7820	GO:0034198	1	0.850838748	0	80
7821	GO:0034199	1	0.968489724	0	16
7822	GO:0034201	1	0.99200419	0	4
7823	GO:0034203	1	0.998013067	0	1
7824	GO:0034204	1	0.974441965	0	13
7825	GO:0034205	1	0.98015415	0	10
7826	GO:0034211	1	0.998013437	0	1
7827	GO:0034213	1	0.99797074	0	1
7828	GO:0034214	1	0.982181589	0	9
7829	GO:0034219	1	0.9960023	0	2
7831	GO:0034224	1	0.997982652	0	1
7832	GO:0034227	1	0.991934393	0	4
7833	GO:0034230	1	0.996006689	0	2
7834	GO:0034231	1	0.998012221	0	1
7835	GO:0034235	1	0.988005907	0	6
7836	GO:0034236	1	0.974270561	0	13
7837	GO:0034237	1	0.953098686	0	24
7838	GO:0034238	1	0.997969861	0	1
7839	GO:0034241	1	0.995972704	0	2
7840	GO:0034242	1	0.998013437	0	1
7841	GO:0034243	1	0.987994439	0	6
7842	GO:0034244	1	0.972242848	0	14
7843	GO:0034245	1	0.998005635	0	1
7844	GO:0034246	1	0.991955601	0	4
7845	GO:0034247	1	0.997965944	0	1
7846	GO:0034260	1	0.913599276	0	45
7847	GO:0034263	1	0.995998485	0	2
7848	GO:0034271	1	0.994001482	0	3
7849	GO:0034272	1	0.994001482	0	3
7850	GO:0034274	1	0.994032474	0	3
7851	GO:0034276	1	0.998012931	0	1
7852	GO:0034314	1	0.968248661	0	16
7853	GO:0034315	1	0.987969676	0	6
7854	GO:0034316	1	0.986011027	0	7
7855	GO:0034329	1	0.964444545	0	18
7856	GO:0034331	1	0.995970058	0	2
7857	GO:0034332	1	0.921178925	0	41
7858	GO:0034333	1	0.978191355	0	11
7859	GO:0034334	1	0.994015218	0	3
7860	GO:0034337	1	0.997976203	0	1
7861	GO:0034338	1	0.99198531	0	4
7863	GO:0034341	1	0.950803923	0	25
7864	GO:0034342	1	0.995985075	0	2
7866	GO:0034346	1	0.995958037	0	2
7867	GO:0034349	1	0.991980516	0	4

7868	GO:0034351	1	0.986038799	0	7
7869	GO:0034352	1	0.997963217	0	1
7870	GO:0034353	1	0.997990564	0	1
7871	GO:0034354	1	0.987936196	0	6
7872	GO:0034355	1	0.997990564	0	1
7873	GO:0034356	1	0.972292328	0	14
7874	GO:0034358	1	0.998013437	0	1
7875	GO:0034359	1	0.998013437	0	1
7876	GO:0034360	1	0.998013437	0	1
7877	GO:0034361	1	0.972053587	0	14
7878	GO:0034362	1	0.976022864	0	12
7879	GO:0034363	1	0.991914112	0	4
7880	GO:0034364	1	0.960171899	0	20
7881	GO:0034365	1	0.99388966	0	3
7882	GO:0034366	1	0.989875042	0	5
7883	GO:0034369	1	0.997949301	0	1
7884	GO:0034370	1	0.997954626	0	1
7885	GO:0034371	1	0.991914112	0	4
7886	GO:0034372	1	0.987850089	0	6
7887	GO:0034373	1	0.99797181	0	1
7888	GO:0034374	1	0.976069749	0	12
7889	GO:0034375	1	0.970060643	0	15
7890	GO:0034377	1	0.998008498	0	1
7891	GO:0034378	1	0.983994073	0	8
7892	GO:0034379	1	0.980094057	0	10
7893	GO:0034380	1	0.982041646	0	9
7894	GO:0034381	1	0.992008749	0	4
7895	GO:0034382	1	0.987926013	0	6
7896	GO:0034383	1	0.954857806	0	23
7897	GO:0034384	1	0.981975444	0	9
7898	GO:0034386	1	0.998012706	0	1
7899	GO:0034388	1	0.990014366	0	5
7900	GO:0034389	1	0.962435481	0	19
7901	GO:0034391	1	0.993998557	0	3
7902	GO:0034392	1	0.987997999	0	6
7903	GO:0034393	1	0.987980035	0	6
7904	GO:0034394	1	0.947373968	0	27
7905	GO:0034395	1	0.997970777	0	1
7906	GO:0034397	1	0.997990564	0	1
7907	GO:0034399	1	0.970430927	0	15
7908	GO:0034401	1	0.997970668	0	1
7909	GO:0034402	1	0.996001191	0	2
7910	GO:0034405	1	0.984021805	0	8
7911	GO:0034414	1	0.997982372	0	1
7912	GO:0034417	1	0.997992012	0	1

7913	GO:0034418	1	0.995970431	0	2
7914	GO:0034421	1	0.994005746	0	3
7915	GO:0034423	1	0.997988712	0	1
7916	GO:0034427	1	0.979934528	0	10
7917	GO:0034431	1	0.98997282	0	5
7918	GO:0034432	1	0.98997282	0	5
7919	GO:0034435	1	0.993960315	0	3
7920	GO:0034436	1	0.99197229	0	4
7921	GO:0034440	1	0.987971556	0	6
7922	GO:0034441	1	0.997975207	0	1
7923	GO:0034445	1	0.99795548	0	1
7924	GO:0034446	1	0.895678043	0	55
7925	GO:0034447	1	0.989959823	0	5
7926	GO:0034450	1	0.97813785	0	11
7927	GO:0034451	1	0.814664602	0	102
7928	GO:0034452	1	0.976302844	0	12
7929	GO:0034453	1	0.970375116	0	15
7930	GO:0034454	1	0.980241418	0	10
7931	GO:0034455	1	0.994001869	0	3
7932	GO:0034456	1	0.99597746	0	2
7933	GO:0034457	1	0.993924716	0	3
7934	GO:0034458	1	0.992047927	0	4
7935	GO:0034462	1	0.994008765	0	3
7936	GO:0034463	1	0.997947485	0	1
7937	GO:0034464	1	0.984061297	0	8
7938	GO:0034465	1	0.993957121	0	3
7939	GO:0034470	1	0.996003435	0	2
7940	GO:0034472	1	0.982106743	0	9
7941	GO:0034473	1	0.993915619	0	3
7942	GO:0034474	1	0.998013437	0	1
7943	GO:0034475	1	0.983852711	0	8
7944	GO:0034476	1	0.993915619	0	3
7945	GO:0034477	1	0.995986257	0	2
7946	GO:0034478	1	0.997993342	0	1
7947	GO:0034485	1	0.988076399	0	6
7948	GO:0034486	1	0.997995468	0	1
7949	GO:0034497	1	0.974288692	0	13
7950	GO:0034498	1	0.984059012	0	8
7951	GO:0034499	1	0.989977227	0	5
7952	GO:0034501	1	0.976097315	0	12
7953	GO:0034502	1	0.992012146	0	4
7954	GO:0034504	1	0.933871695	0	34
7955	GO:0034505	1	0.998013437	0	1
7956	GO:0034506	1	0.998007827	0	1
7957	GO:0034508	1	0.993935514	0	3

7958	GO:0034511	1	0.980127487	0	10
7959	GO:0034512	1	0.996001487	0	2
7960	GO:0034513	1	0.98990497	0	5
7961	GO:0034514	1	0.997983728	0	1
7962	GO:0034515	1	0.995995445	0	2
7963	GO:0034516	1	0.997969613	0	1
7964	GO:0034518	1	0.993995783	0	3
7965	GO:0034545	1	0.997973115	0	1
7966	GO:0034551	1	0.981996672	0	9
7967	GO:0034553	1	0.991932205	0	4
7968	GO:0034584	1	0.994015744	0	3
7969	GO:0034587	1	0.970297809	0	15
7970	GO:0034589	1	0.99801003	0	1
7971	GO:0034590	1	0.99801003	0	1
7972	GO:0034593	1	0.996004693	0	2
7973	GO:0034594	1	0.997997701	0	1
7974	GO:0034595	1	0.992044473	0	4
7975	GO:0034596	1	0.997996596	0	1
7976	GO:0034597	1	0.992026385	0	4
7977	GO:0034599	1	0.815489114	0	101
7978	GO:0034602	1	0.998010047	0	1
7979	GO:0034604	1	0.990012139	0	5
7981	GO:0034612	1	0.937665576	0	32
7982	GO:0034613	1	0.868753578	0	70
7983	GO:0034614	1	0.930041688	0	36
7984	GO:0034616	1	0.984083094	0	8
7985	GO:0034617	1	0.994020685	0	3
7986	GO:0034618	1	0.986035411	0	7
7987	GO:0034620	1	0.949033353	0	26
7988	GO:0034622	1	0.958591598	0	21
7989	GO:0034625	1	0.987988928	0	6
7990	GO:0034626	1	0.987988928	0	6
7991	GO:0034627	1	0.997985749	0	1
7992	GO:0034629	1	0.982077249	0	9
7993	GO:0034632	1	0.997992028	0	1
7994	GO:0034633	1	0.997992028	0	1
7995	GO:0034634	1	0.99007443	0	5
7996	GO:0034635	1	0.995941288	0	2
7997	GO:0034638	1	0.980039229	0	10
7998	GO:0034640	1	0.997997805	0	1
7999	GO:0034642	1	0.998005657	0	1
8000	GO:0034644	1	0.895334553	0	55
8001	GO:0034645	1	0.996023937	0	2
8002	GO:0034647	1	0.992076828	0	4
8003	GO:0034648	1	0.992052245	0	4

8004	GO:0034649	1	0.998006426	0	1
8005	GO:0034650	1	0.997978128	0	1
8006	GO:0034651	1	0.998013437	0	1
8007	GO:0034653	1	0.991971393	0	4
8008	GO:0034656	1	0.970197872	0	15
8009	GO:0034657	1	0.991990994	0	4
8010	GO:0034663	1	0.980061723	0	10
8011	GO:0034665	1	0.996021365	0	2
8012	GO:0034666	1	0.994025512	0	3
8013	GO:0034667	1	0.996021757	0	2
8014	GO:0034668	1	0.994037003	0	3
8015	GO:0034669	1	0.996008328	0	2
8016	GO:0034670	1	0.998013437	0	1
8017	GO:0034673	1	0.994006801	0	3
8018	GO:0034674	1	0.996018743	0	2
8019	GO:0034676	1	0.998013437	0	1
8020	GO:0034677	1	0.998005048	0	1
8021	GO:0034678	1	0.994026984	0	3
8022	GO:0034679	1	0.998007398	0	1
8023	GO:0034680	1	0.996022109	0	2
8024	GO:0034681	1	0.996021857	0	2
8025	GO:0034683	1	0.996029964	0	2
8026	GO:0034684	1	0.996017357	0	2
8027	GO:0034685	1	0.996002516	0	2
8028	GO:0034686	1	0.996030731	0	2
8029	GO:0034687	1	0.996010518	0	2
8030	GO:0034688	1	0.996009728	0	2
8031	GO:0034689	1	0.9960094	0	2
8032	GO:0034693	1	0.997986047	0	1
8033	GO:0034695	1	0.987959148	0	6
8034	GO:0034696	1	0.995972241	0	2
8035	GO:0034698	1	0.988014001	0	6
8036	GO:0034699	1	0.998013437	0	1
8037	GO:0034702	1	0.988100005	0	6
8038	GO:0034703	1	0.988130841	0	6
8040	GO:0034705	1	0.986032287	0	7
8041	GO:0034706	1	0.9840878	0	8
8042	GO:0034707	1	0.919050589	0	42
8043	GO:0034708	1	0.997980878	0	1
8044	GO:0034709	1	0.975864283	0	12
8045	GO:0034711	1	0.989988904	0	5
8046	GO:0034713	1	0.982107575	0	9
8047	GO:0034714	1	0.994033926	0	3
8048	GO:0034715	1	0.987850625	0	6
8049	GO:0034718	1	0.998013373	0	1

8050	GO:0034719	1	0.966127081	0	17
8051	GO:0034720	1	0.984145498	0	8
8052	GO:0034721	1	0.990105648	0	5
8053	GO:0034722	1	0.997969714	0	1
8054	GO:0034724	1	0.998012067	0	1
8055	GO:0034727	1	0.990082389	0	5
8056	GO:0034728	1	0.994051875	0	3
8057	GO:0034729	1	0.998013437	0	1
8058	GO:0034736	1	0.998000348	0	1
8059	GO:0034739	1	0.998009617	0	1
8060	GO:0034750	1	0.99601776	0	2
8061	GO:0034751	1	0.982105104	0	9
8062	GO:0034752	1	0.998013437	0	1
8063	GO:0034755	1	0.988067737	0	6
8064	GO:0034756	1	0.993940997	0	3
8065	GO:0034760	1	0.995965933	0	2
8066	GO:0034763	1	0.998013437	0	1
8067	GO:0034765	1	0.776309528	0	126
8068	GO:0034766	1	0.99197177	0	4
8069	GO:0034767	1	0.992023786	0	4
8070	GO:0034769	1	0.995992352	0	2
8071	GO:0034770	1	0.996007903	0	2
8072	GO:0034772	1	0.995985075	0	2
8073	GO:0034773	1	0.988022606	0	6
8074	GO:0034774	1	0.805100336	0	107
8075	GO:0034775	1	0.988107139	0	6
8076	GO:0034776	1	0.993958795	0	3
8077	GO:0034777	1	0.997982444	0	1
8078	GO:0034875	1	0.991964721	0	4
8079	GO:0034899	1	0.997979932	0	1
8080	GO:0034959	1	0.998013105	0	1
8081	GO:0034968	1	0.956781733	0	22
8082	GO:0034969	1	0.991960512	0	4
8083	GO:0034970	1	0.995986737	0	2
8084	GO:0034971	1	0.997994036	0	1
8085	GO:0034972	1	0.997982002	0	1
8086	GO:0034975	1	0.980150962	0	10
8087	GO:0034976	1	0.841107981	0	86
8088	GO:0034979	1	0.989985308	0	5
8089	GO:0034982	1	0.987983446	0	6
8090	GO:0034983	1	0.984069866	0	8
8091	GO:0034986	1	0.996008652	0	2
8092	GO:0034987	1	0.991945193	0	4
8093	GO:0034988	1	0.996003159	0	2
8094	GO:0034990	1	0.995992276	0	2

8095	GO:0034991	1	0.990055323	0	5
8096	GO:0034993	1	0.980136173	0	10
8097	GO:0034998	1	0.995980988	0	2
8098	GO:0035000	1	0.997991532	0	1
8099	GO:0035004	1	0.986132025	0	7
8100	GO:0035005	1	0.986149583	0	7
8101	GO:0035014	1	0.990070854	0	5
8102	GO:0035019	1	0.917369313	0	43
8103	GO:0035020	1	0.974263611	0	13
8104	GO:0035021	1	0.993996637	0	3
8105	GO:0035022	1	0.986072456	0	7
8106	GO:0035023	1	0.947384899	0	27
8107	GO:0035024	1	0.956899779	0	22
8108	GO:0035025	1	0.945268754	0	28
8109	GO:0035026	1	0.998011678	0	1
8110	GO:0035032	1	0.989996463	0	5
8111	GO:0035033	1	0.995960377	0	2
8112	GO:0035034	1	0.995981053	0	2
8114	GO:0035036	1	0.975959961	0	12
8115	GO:0035037	1	0.998011282	0	1
8116	GO:0035038	1	0.995983077	0	2
8117	GO:0035039	1	0.997983713	0	1
8118	GO:0035050	1	0.978148	0	11
8119	GO:0035051	1	0.997996626	0	1
8120	GO:0035054	1	0.995982112	0	2
8121	GO:0035060	1	0.99401193	0	3
8122	GO:0035061	1	0.99007879	0	5
8123	GO:0035063	1	0.991991162	0	4
8124	GO:0035064	1	0.877501505	0	65
8125	GO:0035065	1	0.984037355	0	8
8126	GO:0035066	1	0.962510724	0	19
8127	GO:0035067	1	0.982046567	0	9
8128	GO:0035082	1	0.956676761	0	22
8129	GO:0035087	1	0.99197095	0	4
8130	GO:0035088	1	0.991966135	0	4
8131	GO:0035089	1	0.99003513	0	5
8132	GO:0035091	1	0.824648077	0	96
8133	GO:0035092	1	0.993992835	0	3
8134	GO:0035093	1	0.990042613	0	5
8135	GO:0035094	1	0.951024488	0	25
8136	GO:0035095	1	0.986071569	0	7
8137	GO:0035097	1	0.951175274	0	25
8138	GO:0035098	1	0.970417826	0	15
8139	GO:0035101	1	0.992008458	0	4
8140	GO:0035102	1	0.970322672	0	15

8141	GO:0035106	1	0.995977128	0	2
8142	GO:0035108	1	0.956899611	0	22
8143	GO:0035112	1	0.99800067	0	1
8144	GO:0035115	1	0.956786468	0	22
8145	GO:0035116	1	0.954811458	0	23
8146	GO:0035128	1	0.998013437	0	1
8147	GO:0035136	1	0.986115912	0	7
8148	GO:0035137	1	0.98607888	0	7
8149	GO:0035145	1	0.960590138	0	20
8150	GO:0035148	1	0.982154617	0	9
8151	GO:0035150	1	0.997969178	0	1
8152	GO:0035162	1	0.968456133	0	16
8153	GO:0035166	1	0.992008449	0	4
8154	GO:0035173	1	0.993982124	0	3
8155	GO:0035174	1	0.989975973	0	5
8156	GO:0035175	1	0.997973646	0	1
8157	GO:0035176	1	0.921282607	0	41
8158	GO:0035189	1	0.996006016	0	2
8159	GO:0035194	1	0.98020007	0	10
8160	GO:0035195	1	0.952475792	0	24
8161	GO:0035196	1	0.970361593	0	15
8162	GO:0035197	1	0.980191727	0	10
8163	GO:0035198	1	0.939649637	0	31
8164	GO:0035212	1	0.997990564	0	1
8165	GO:0035226	1	0.997995536	0	1
8166	GO:0035229	1	0.997995536	0	1
8167	GO:0035234	1	0.988056012	0	6
8168	GO:0035235	1	0.968561806	0	16
8169	GO:0035238	1	0.997990564	0	1
8170	GO:0035239	1	0.98807371	0	6
8171	GO:0035240	1	0.991938283	0	4
8172	GO:0035241	1	0.989956779	0	5
8173	GO:0035242	1	0.989956858	0	5
8174	GO:0035243	1	0.991975634	0	4
8175	GO:0035246	1	0.982012597	0	9
8176	GO:0035247	1	0.993956104	0	3
8177	GO:0035248	1	0.997992353	0	1
8178	GO:0035249	1	0.95686084	0	22
8179	GO:0035250	1	0.996004259	0	2
8180	GO:0035251	1	0.993983524	0	3
8181	GO:0035252	1	0.988005374	0	6
8182	GO:0035253	1	0.97626049	0	12
8183	GO:0035254	1	0.984117357	0	8
8184	GO:0035255	1	0.956869114	0	22
8185	GO:0035256	1	0.993969328	0	3

8187	GO:0035259	1	0.972408665	0	14
8188	GO:0035261	1	0.996030731	0	2
8189	GO:0035262	1	0.997983426	0	1
8190	GO:0035264	1	0.86735457	0	71
8191	GO:0035265	1	0.978202362	0	11
8192	GO:0035267	1	0.962385524	0	19
8193	GO:0035268	1	0.993934711	0	3
8194	GO:0035269	1	0.96444514	0	18
8195	GO:0035270	1	0.997991625	0	1
8196	GO:0035272	1	0.997972169	0	1
8197	GO:0035276	1	0.99398537	0	3
8198	GO:0035278	1	0.964224751	0	18
8199	GO:0035279	1	0.993995838	0	3
8200	GO:0035280	1	0.986040373	0	7
8201	GO:0035281	1	0.995978993	0	2
8202	GO:0035282	1	0.993999622	0	3
8203	GO:0035284	1	0.995995784	0	2
8204	GO:0035295	1	0.99405111	0	3
8205	GO:0035299	1	0.998010682	0	1
8206	GO:0035303	1	0.990026223	0	5
8207	GO:0035304	1	0.996000476	0	2
8208	GO:0035305	1	0.995989465	0	2
8209	GO:0035306	1	0.99800808	0	1
8210	GO:0035307	1	0.951005071	0	25
8211	GO:0035308	1	0.984073284	0	8
8212	GO:0035312	1	0.992040272	0	4
8213	GO:0035313	1	0.9821124	0	9
8214	GO:0035315	1	0.988031105	0	6
8215	GO:0035325	1	0.988028581	0	6
8216	GO:0035327	1	0.95480533	0	23
8217	GO:0035329	1	0.94551332	0	28
8218	GO:0035330	1	0.992033113	0	4
8219	GO:0035331	1	0.97436377	0	13
8220	GO:0035332	1	0.992001574	0	4
8221	GO:0035333	1	0.98602122	0	7
8222	GO:0035335	1	0.817902488	0	100
8223	GO:0035336	1	0.986052571	0	7
8224	GO:0035338	1	0.96639204	0	17
8225	GO:0035339	1	0.991928358	0	4
8226	GO:0035340	1	0.99798625	0	1
8227	GO:0035344	1	0.997986954	0	1
8228	GO:0035349	1	0.995983296	0	2
8229	GO:0035350	1	0.997982916	0	1
8230	GO:0035351	1	0.996004829	0	2
8231	GO:0035352	1	0.997982916	0	1

8232	GO:0035354	1	0.99599623	0	2
8233	GO:0035355	1	0.998013437	0	1
8234	GO:0035356	1	0.987983387	0	6
8235	GO:0035357	1	0.986102023	0	7
8236	GO:0035358	1	0.995999379	0	2
8237	GO:0035359	1	0.995997723	0	2
8238	GO:0035360	1	0.984021134	0	8
8239	GO:0035363	1	0.991984476	0	4
8240	GO:0035364	1	0.997986954	0	1
8241	GO:0035368	1	0.989982127	0	5
8242	GO:0035370	1	0.995975116	0	2
8243	GO:0035371	1	0.956963879	0	22
8244	GO:0035372	1	0.98411242	0	8
8245	GO:0035373	1	0.994009289	0	3
8246	GO:0035374	1	0.98206777	0	9
8247	GO:0035375	1	0.998013437	0	1
8248	GO:0035377	1	0.994028365	0	3
8249	GO:0035378	1	0.997990556	0	1
8250	GO:0035379	1	0.997990556	0	1
8251	GO:0035385	1	0.990069116	0	5
8253	GO:0035397	1	0.997996022	0	1
8254	GO:0035401	1	0.998013295	0	1
8255	GO:0035402	1	0.995988269	0	2
8256	GO:0035403	1	0.996030638	0	2
8257	GO:0035404	1	0.991995103	0	4
8258	GO:0035407	1	0.995988269	0	2
8259	GO:0035408	1	0.996030638	0	2
8260	GO:0035409	1	0.998013295	0	1
8261	GO:0035410	1	0.991902885	0	4
8262	GO:0035418	1	0.976291634	0	12
8263	GO:0035425	1	0.98395765	0	8
8264	GO:0035426	1	0.994032035	0	3
8265	GO:0035434	1	0.992032923	0	4
8266	GO:0035435	1	0.9722573	0	14
8267	GO:0035437	1	0.99402684	0	3
8268	GO:0035438	1	0.997990564	0	1
8269	GO:0035441	1	0.994024277	0	3
8270	GO:0035444	1	0.998009073	0	1
8271	GO:0035445	1	0.997996143	0	1
8276	GO:0035459	1	0.97621855	0	12
8277	GO:0035461	1	0.992013391	0	4
8278	GO:0035469	1	0.992043484	0	4
8279	GO:0035470	1	0.9899223	0	5
8280	GO:0035473	1	0.992005159	0	4
8281	GO:0035481	1	0.994001995	0	3

8282	GO:0035483	1	0.995911034	0	2
8283	GO:0035485	1	0.997975964	0	1
8284	GO:0035491	1	0.997996836	0	1
8285	GO:0035493	1	0.976082735	0	12
8286	GO:0035494	1	0.993985307	0	3
8287	GO:0035497	1	0.970361858	0	15
8288	GO:0035498	1	0.997990564	0	1
8289	GO:0035499	1	0.99800715	0	1
8290	GO:0035500	1	0.996000712	0	2
8291	GO:0035501	1	0.997991726	0	1
8292	GO:0035502	1	0.991995743	0	4
8293	GO:0035505	1	0.995973341	0	2
8294	GO:0035507	1	0.996030719	0	2
8295	GO:0035508	1	0.998013425	0	1
8296	GO:0035509	1	0.990052293	0	5
8297	GO:0035511	1	0.993948757	0	3
8298	GO:0035513	1	0.995995199	0	2
8299	GO:0035515	1	0.992025379	0	4
8300	GO:0035516	1	0.99398927	0	3
8301	GO:0035517	1	0.992061097	0	4
8302	GO:0035518	1	0.976206115	0	12
8303	GO:0035519	1	0.989934319	0	5
8304	GO:0035520	1	0.988114723	0	6
8305	GO:0035522	1	0.992016857	0	4
8306	GO:0035523	1	0.990062493	0	5
8307	GO:0035524	1	0.990060168	0	5
8308	GO:0035525	1	0.997987849	0	1
8309	GO:0035526	1	0.996026547	0	2
8310	GO:0035529	1	0.98198101	0	9
8311	GO:0035538	1	0.997998269	0	1
8312	GO:0035539	1	0.995937479	0	2
8313	GO:0035542	1	0.990018712	0	5
8314	GO:0035543	1	0.995991431	0	2
8315	GO:0035544	1	0.994022197	0	3
8316	GO:0035545	1	0.998013437	0	1
8317	GO:0035552	1	0.993980986	0	3
8318	GO:0035553	1	0.993993696	0	3
8319	GO:0035556	1	0.417197764	0	432
8320	GO:0035562	1	0.986102455	0	7
8321	GO:0035563	1	0.972271942	0	14
8322	GO:0035564	1	0.996025389	0	2
8323	GO:0035565	1	0.997991284	0	1
8324	GO:0035567	1	0.976259985	0	12
8325	GO:0035568	1	0.997990564	0	1
8326	GO:0035570	1	0.997990564	0	1

8327	GO:0035572	1	0.997990564	0	1
8328	GO:0035573	1	0.997990564	0	1
8329	GO:0035574	1	0.996007903	0	2
8330	GO:0035575	1	0.996007903	0	2
8331	GO:0035577	1	0.89152985	0	57
8332	GO:0035578	1	0.859532654	0	75
8333	GO:0035579	1	0.849579342	0	81
8334	GO:0035580	1	0.900308148	0	52
8335	GO:0035581	1	0.997998041	0	1
8336	GO:0035582	1	0.993978475	0	3
8337	GO:0035583	1	0.992053866	0	4
8338	GO:0035584	1	0.958542715	0	21
8339	GO:0035585	1	0.99401227	0	3
8340	GO:0035589	1	0.980033129	0	10
8341	GO:0035590	1	0.954655065	0	23
8342	GO:0035591	1	0.947246118	0	27
8343	GO:0035594	1	0.99799971	0	1
8344	GO:0035596	1	0.995981555	0	2
8345	GO:0035597	1	0.997979018	0	1
8346	GO:0035598	1	0.997998584	0	1
8347	GO:0035600	1	0.995981555	0	2
8348	GO:0035602	1	0.998008455	0	1
8349	GO:0035603	1	0.998008455	0	1
8350	GO:0035604	1	0.998008455	0	1
8351	GO:0035605	1	0.997966382	0	1
8352	GO:0035606	1	0.995922108	0	2
8353	GO:0035607	1	0.998008455	0	1
8354	GO:0035608	1	0.99400887	0	3
8355	GO:0035609	1	0.992018683	0	4
8356	GO:0035610	1	0.990048544	0	5
8357	GO:0035611	1	0.997991958	0	1
8358	GO:0035612	1	0.982141779	0	9
8359	GO:0035613	1	0.972337277	0	14
8360	GO:0035615	1	0.968411317	0	16
8361	GO:0035616	1	0.992036132	0	4
8362	GO:0035617	1	0.990009107	0	5
8363	GO:0035621	1	0.993979647	0	3
8364	GO:0035622	1	0.996024111	0	2
8365	GO:0035623	1	0.997998011	0	1
8366	GO:0035624	1	0.990037302	0	5
8367	GO:0035627	1	0.982032406	0	9
8368	GO:0035630	1	0.988048941	0	6
8369	GO:0035631	1	0.978134039	0	11
8370	GO:0035632	1	0.995948329	0	2
8371	GO:0035633	1	0.939628165	0	31

8372	GO:0035634	1	0.995947244	0	2
8373	GO:0035635	1	0.98417766	0	8
8374	GO:0035639	1	0.997960537	0	1
8375	GO:0035640	1	0.984146617	0	8
8376	GO:0035641	1	0.978219475	0	11
8377	GO:0035642	1	0.997994036	0	1
8378	GO:0035643	1	0.997973451	0	1
8379	GO:0035644	1	0.997994489	0	1
8380	GO:0035645	1	0.998009898	0	1
8381	GO:0035646	1	0.980060944	0	10
8382	GO:0035650	1	0.989964539	0	5
8383	GO:0035651	1	0.99395348	0	3
8384	GO:0035655	1	0.987965266	0	6
8385	GO:0035657	1	0.998012455	0	1
8386	GO:0035658	1	0.989988522	0	5
8387	GO:0035662	1	0.99185119	0	4
8388	GO:0035663	1	0.9939922	0	3
8389	GO:0035665	1	0.997974939	0	1
8391	GO:0035669	1	0.994032645	0	3
8392	GO:0035672	1	0.998004409	0	1
8393	GO:0035673	1	0.99601384	0	2
8394	GO:0035674	1	0.997997664	0	1
8395	GO:0035675	1	0.997988914	0	1
8396	GO:0035683	1	0.996029416	0	2
8397	GO:0035684	1	0.997955114	0	1
8398	GO:0035685	1	0.996000827	0	2
8399	GO:0035686	1	0.989958957	0	5
8400	GO:0035690	1	0.886338206	0	60
8401	GO:0035691	1	0.997966363	0	1
8402	GO:0035692	1	0.995983175	0	2
8403	GO:0035693	1	0.997966363	0	1
8404	GO:0035694	1	0.988028185	0	6
8405	GO:0035695	1	0.998010047	0	1
8406	GO:0035696	1	0.99200282	0	4
8407	GO:0035701	1	0.994021601	0	3
8408	GO:0035702	1	0.996020901	0	2
8409	GO:0035709	1	0.995993393	0	2
8411	GO:0035711	1	0.995992485	0	2
8412	GO:0035712	1	0.998001552	0	1
8413	GO:0035713	1	0.998001552	0	1
8414	GO:0035718	1	0.995940057	0	2
8415	GO:0035720	1	0.993955434	0	3
8416	GO:0035721	1	0.976267118	0	12
8417	GO:0035722	1	0.913083405	0	45
8418	GO:0035723	1	0.974298202	0	13

8419	GO:0035725	1	0.84176339	0	86
8420	GO:0035726	1	0.988024415	0	6
8421	GO:0035727	1	0.988006355	0	6
8422	GO:0035728	1	0.996000953	0	2
8423	GO:0035729	1	0.968368939	0	16
8424	GO:0035730	1	0.997959464	0	1
8425	GO:0035731	1	0.997959464	0	1
8426	GO:0035732	1	0.997959464	0	1
8427	GO:0035733	1	0.996012865	0	2
8428	GO:0035735	1	0.922720732	0	40
8429	GO:0035747	1	0.996030578	0	2
8430	GO:0035748	1	0.988076242	0	6
8431	GO:0035749	1	0.990019338	0	5
8432	GO:0035750	1	0.99801334	0	1
8433	GO:0035751	1	0.991978289	0	4
8434	GO:0035752	1	0.997977002	0	1
8435	GO:0035754	1	0.987957943	0	6
8436	GO:0035755	1	0.997987748	0	1
8437	GO:0035757	1	0.997981373	0	1
8438	GO:0035758	1	0.997981373	0	1
8439	GO:0035759	1	0.997989991	0	1
8440	GO:0035767	1	0.982071717	0	9
8441	GO:0035768	1	0.997964216	0	1
8442	GO:0035770	1	0.974240654	0	13
8443	GO:0035771	1	0.991999856	0	4
8444	GO:0035772	1	0.997974522	0	1
8445	GO:0035773	1	0.982122575	0	9
8446	GO:0035774	1	0.937594331	0	32
8447	GO:0035780	1	0.997991269	0	1
8448	GO:0035781	1	0.995967857	0	2
8449	GO:0035783	1	0.997969861	0	1
8450	GO:0035787	1	0.998013328	0	1
8451	GO:0035789	1	0.998013436	0	1
8452	GO:0035790	1	0.998013437	0	1
8453	GO:0035791	1	0.98807432	0	6
8454	GO:0035793	1	0.994008519	0	3
8455	GO:0035794	1	0.991990636	0	4
8456	GO:0035795	1	0.991954524	0	4
8457	GO:0035798	1	0.997965198	0	1
8458	GO:0035799	1	0.994018275	0	3
8459	GO:0035800	1	0.995977851	0	2
8460	GO:0035801	1	0.998008832	0	1
8461	GO:0035802	1	0.997975026	0	1
8462	GO:0035803	1	0.995940565	0	2
8463	GO:0035804	1	0.995940565	0	2

8464	GO:0035805	1	0.995954471	0	2
8465	GO:0035809	1	0.995940994	0	2
8466	GO:0035810	1	0.976152556	0	12
8467	GO:0035811	1	0.992035127	0	4
8468	GO:0035812	1	0.995995081	0	2
8469	GO:0035813	1	0.99600456	0	2
8470	GO:0035814	1	0.991961328	0	4
8471	GO:0035815	1	0.984035515	0	8
8472	GO:0035821	1	0.995907884	0	2
8473	GO:0035822	1	0.998013437	0	1
8474	GO:0035825	1	0.997989557	0	1
8475	GO:0035838	1	0.998009198	0	1
8476	GO:0035841	1	0.998013437	0	1
8477	GO:0035844	1	0.998013416	0	1
8478	GO:0035845	1	0.97429291	0	13
8479	GO:0035846	1	0.994021436	0	3
8480	GO:0035847	1	0.997983203	0	1
8481	GO:0035849	1	0.997983203	0	1
8482	GO:0035851	1	0.995978139	0	2
8483	GO:0035852	1	0.997983203	0	1
8484	GO:0035853	1	0.9980111	0	1
8485	GO:0035855	1	0.968531203	0	16
8486	GO:0035859	1	0.997989673	0	1
8487	GO:0035860	1	0.98801342	0	6
8488	GO:0035861	1	0.870348845	0	69
8489	GO:0035863	1	0.998013437	0	1
8490	GO:0035864	1	0.990007571	0	5
8491	GO:0035865	1	0.98410075	0	8
8492	GO:0035866	1	0.99405111	0	3
8493	GO:0035867	1	0.992076097	0	4
8494	GO:0035868	1	0.994038364	0	3
8495	GO:0035869	1	0.933828642	0	34
8496	GO:0035870	1	0.995979654	0	2
8497	GO:0035871	1	0.980231456	0	10
8498	GO:0035872	1	0.997979323	0	1
8499	GO:0035873	1	0.988037983	0	6
8500	GO:0035874	1	0.997990564	0	1
8501	GO:0035877	1	0.991971572	0	4
8502	GO:0035878	1	0.990029155	0	5
8503	GO:0035879	1	0.994009787	0	3
8504	GO:0035880	1	0.998005083	0	1
8505	GO:0035881	1	0.996006687	0	2
8506	GO:0035883	1	0.997990564	0	1
8507	GO:0035886	1	0.997993188	0	1
8508	GO:0035898	1	0.995992233	0	2

8509	GO:0035900	1	0.985986064	0	7
8510	GO:0035902	1	0.962374066	0	19
8511	GO:0035904	1	0.955070707	0	23
8512	GO:0035905	1	0.998006617	0	1
8513	GO:0035906	1	0.998006617	0	1
8514	GO:0035907	1	0.996002813	0	2
8515	GO:0035909	1	0.968420381	0	16
8516	GO:0035910	1	0.99200756	0	4
8517	GO:0035912	1	0.984085797	0	8
8518	GO:0035914	1	0.932060704	0	35
8519	GO:0035915	1	0.995994438	0	2
8520	GO:0035921	1	0.997980186	0	1
8521	GO:0035924	1	0.941397092	0	30
8522	GO:0035925	1	0.951038526	0	25
8523	GO:0035927	1	0.998011657	0	1
8524	GO:0035928	1	0.993946157	0	3
8525	GO:0035934	1	0.997990564	0	1
8526	GO:0035945	1	0.997986375	0	1
8527	GO:0035946	1	0.997986375	0	1
8528	GO:0035963	1	0.99599934	0	2
8529	GO:0035964	1	0.998008841	0	1
8530	GO:0035965	1	0.986002085	0	7
8531	GO:0035967	1	0.998008667	0	1
8532	GO:0035970	1	0.956715764	0	22
8533	GO:0035971	1	0.997964583	0	1
8534	GO:0035973	1	0.947086255	0	27
8535	GO:0035976	1	0.991955861	0	4
8536	GO:0035978	1	0.997991726	0	1
8537	GO:0035984	1	0.994020682	0	3
8538	GO:0035985	1	0.993945283	0	3
8539	GO:0035986	1	0.993945283	0	3
8540	GO:0035987	1	0.949288496	0	26
8541	GO:0035988	1	0.974234961	0	13
8542	GO:0035989	1	0.994001838	0	3
8543	GO:0035990	1	0.995969463	0	2
8544	GO:0035992	1	0.997990564	0	1
8545	GO:0035993	1	0.993959846	0	3
8546	GO:0035994	1	0.968369806	0	16
8547	GO:0035995	1	0.988020327	0	6
8548	GO:0035998	1	0.997977827	0	1
8549	GO:0035999	1	0.980037145	0	10
8550	GO:0036002	1	0.970327947	0	15
8551	GO:0036003	1	0.985951399	0	7
8552	GO:0036004	1	0.997964833	0	1
8553	GO:0036006	1	0.992033299	0	4

8554	GO:0036009	1	0.998012455	0	1
8555	GO:0036010	1	0.980168933	0	10
8556	GO:0036016	1	0.988002154	0	6
8557	GO:0036017	1	0.997948513	0	1
8558	GO:0036018	1	0.993880183	0	3
8559	GO:0036019	1	0.991982687	0	4
8560	GO:0036020	1	0.970375629	0	15
8561	GO:0036021	1	0.989998222	0	5
8562	GO:0036022	1	0.997999867	0	1
8563	GO:0036023	1	0.993965029	0	3
8564	GO:0036031	1	0.995985075	0	2
8565	GO:0036033	1	0.992072209	0	4
8566	GO:0036035	1	0.988051555	0	6
8567	GO:0036037	1	0.991944893	0	4
8568	GO:0036038	1	0.974107329	0	13
8569	GO:0036041	1	0.991927196	0	4
8570	GO:0036042	1	0.987910585	0	6
8571	GO:0036046	1	0.997996671	0	1
8572	GO:0036047	1	0.997996671	0	1
8573	GO:0036048	1	0.997996671	0	1
8574	GO:0036049	1	0.995974424	0	2
8575	GO:0036053	1	0.997989991	0	1
8576	GO:0036054	1	0.997996671	0	1
8577	GO:0036055	1	0.995974424	0	2
8578	GO:0036057	1	0.984187597	0	8
8579	GO:0036060	1	0.998007851	0	1
8580	GO:0036064	1	0.727187171	0	158
8581	GO:0036065	1	0.978175186	0	11
8582	GO:0036066	1	0.990005771	0	5
8583	GO:0036071	1	0.995998103	0	2
8584	GO:0036085	1	0.997997568	0	1
8585	GO:0036089	1	0.986056311	0	7
8586	GO:0036090	1	0.997979529	0	1
8587	GO:0036091	1	0.989970409	0	5
8588	GO:0036092	1	0.9744094	0	13
8589	GO:0036094	1	0.983895689	0	8
8590	GO:0036101	1	0.989977197	0	5
8591	GO:0036102	1	0.997965198	0	1
8592	GO:0036105	1	0.998003224	0	1
8593	GO:0036109	1	0.976116693	0	12
8594	GO:0036111	1	0.997989596	0	1
8595	GO:0036112	1	0.995987481	0	2
8596	GO:0036113	1	0.998003887	0	1
8597	GO:0036114	1	0.995934964	0	2
8598	GO:0036115	1	0.997959385	0	1

8599	GO:0036116	1	0.997970421	0	1
8600	GO:0036117	1	0.995971877	0	2
8601	GO:0036119	1	0.997993836	0	1
8602	GO:0036120	1	0.962614954	0	19
8603	GO:0036121	1	0.994006547	0	3
8604	GO:0036122	1	0.978145401	0	11
8605	GO:0036123	1	0.994009726	0	3
8606	GO:0036124	1	0.992035908	0	4
8607	GO:0036126	1	0.893264953	0	56
8608	GO:0036128	1	0.984025203	0	8
8609	GO:0036130	1	0.995936801	0	2
8610	GO:0036131	1	0.997964698	0	1
8611	GO:0036132	1	0.993977933	0	3
8612	GO:0036134	1	0.993952432	0	3
8613	GO:0036137	1	0.997990564	0	1
8614	GO:0036138	1	0.998013437	0	1
8615	GO:0036139	1	0.998013437	0	1
8616	GO:0036140	1	0.998013437	0	1
8617	GO:0036143	1	0.99795726	0	1
8618	GO:0036145	1	0.997974887	0	1
8619	GO:0036146	1	0.997990564	0	1
8620	GO:0036148	1	0.97030747	0	15
8621	GO:0036149	1	0.970273791	0	15
8622	GO:0036150	1	0.96250947	0	19
8623	GO:0036151	1	0.947059734	0	27
8624	GO:0036152	1	0.954747168	0	23
8625	GO:0036153	1	0.997991424	0	1
8626	GO:0036155	1	0.990011557	0	5
8627	GO:0036156	1	0.992054121	0	4
8628	GO:0036157	1	0.980158967	0	10
8629	GO:0036158	1	0.966390947	0	17
8630	GO:0036159	1	0.968318625	0	16
8631	GO:0036164	1	0.997962231	0	1
8632	GO:0036166	1	0.99800641	0	1
8633	GO:0036185	1	0.997965198	0	1
8634	GO:0036186	1	0.996009507	0	2
8635	GO:0036195	1	0.995944467	0	2
8636	GO:0036211	1	0.998007582	0	1
8637	GO:0036218	1	0.989971468	0	5
8638	GO:0036221	1	0.99798012	0	1
8639	GO:0036228	1	0.995998396	0	2
8640	GO:0036245	1	0.995985942	0	2
8641	GO:0036250	1	0.997977399	0	1
8642	GO:0036255	1	0.998005759	0	1
8643	GO:0036257	1	0.997991323	0	1

8644	GO:0036258	1	0.941264893	0	30
8645	GO:0036261	1	0.998004245	0	1
8646	GO:0036265	1	0.995944175	0	2
8647	GO:0036268	1	0.998013437	0	1
8648	GO:0036269	1	0.998013437	0	1
8649	GO:0036284	1	0.997969916	0	1
8650	GO:0036289	1	0.982157737	0	9
8651	GO:0036292	1	0.998004379	0	1
8652	GO:0036293	1	0.998004621	0	1
8653	GO:0036294	1	0.992023083	0	4
8654	GO:0036295	1	0.985974524	0	7
8655	GO:0036297	1	0.897109065	0	54
8656	GO:0036298	1	0.995994401	0	2
8657	GO:0036302	1	0.982176327	0	9
8658	GO:0036303	1	0.994040446	0	3
8659	GO:0036304	1	0.995976691	0	2
8660	GO:0036305	1	0.998013433	0	1
8661	GO:0036309	1	0.996030731	0	2
8662	GO:0036310	1	0.986105085	0	7
8663	GO:0036312	1	0.980230942	0	10
8664	GO:0036313	1	0.993984554	0	3
8665	GO:0036315	1	0.994010339	0	3
8666	GO:0036316	1	0.995981825	0	2
8667	GO:0036317	1	0.997990564	0	1
8668	GO:0036321	1	0.997990564	0	1
8669	GO:0036323	1	0.998012199	0	1
8670	GO:0036324	1	0.992012944	0	4
8671	GO:0036332	1	0.998012199	0	1
8672	GO:0036333	1	0.997988704	0	1
8673	GO:0036335	1	0.99396788	0	3
8674	GO:0036336	1	0.988047235	0	6
8675	GO:0036337	1	0.997987904	0	1
8676	GO:0036339	1	0.997999867	0	1
8677	GO:0036342	1	0.97233757	0	14
8678	GO:0036343	1	0.995961699	0	2
8679	GO:0036344	1	0.998004752	0	1
8680	GO:0036345	1	0.997992539	0	1
8681	GO:0036351	1	0.997983728	0	1
8682	GO:0036352	1	0.997983728	0	1
8683	GO:0036353	1	0.984076969	0	8
8684	GO:0036359	1	0.995994367	0	2
8685	GO:0036363	1	0.998011569	0	1
8686	GO:0036364	1	0.997990564	0	1
8687	GO:0036369	1	0.996030731	0	2
8688	GO:0036371	1	0.995997837	0	2

8689	GO:0036372	1	0.997959783	0	1
8690	GO:0036373	1	0.997990564	0	1
8691	GO:0036374	1	0.985969467	0	7
8692	GO:0036376	1	0.974224894	0	13
8693	GO:0036378	1	0.9919566	0	4
8694	GO:0036379	1	0.997967554	0	1
8695	GO:0036384	1	0.995987577	0	2
8696	GO:0036388	1	0.878280117	0	64
8697	GO:0036393	1	0.997992786	0	1
8698	GO:0036396	1	0.984070044	0	8
8699	GO:0036402	1	0.987879853	0	6
8700	GO:0036403	1	0.995980105	0	2
8701	GO:0036404	1	0.998013437	0	1
8702	GO:0036409	1	0.996006177	0	2
8703	GO:0036413	1	0.995997481	0	2
8704	GO:0036414	1	0.992020464	0	4
8705	GO:0036416	1	0.996012728	0	2
8706	GO:0036424	1	0.995963187	0	2
8707	GO:0036425	1	0.995963187	0	2
8710	GO:0036435	1	0.984020523	0	8
8711	GO:0036438	1	0.992006905	0	4
8712	GO:0036440	1	0.997994098	0	1
8713	GO:0036444	1	0.97814823	0	11
8714	GO:0036449	1	0.986106978	0	7
8715	GO:0036456	1	0.997969548	0	1
8716	GO:0036457	1	0.992022467	0	4
8717	GO:0036458	1	0.997981777	0	1
8718	GO:0036461	1	0.995936376	0	2
8719	GO:0036462	1	0.98798971	0	6
8720	GO:0036463	1	0.99800821	0	1
8721	GO:0036464	1	0.852953268	0	79
8722	GO:0036465	1	0.986012591	0	7
8723	GO:0036466	1	0.995984204	0	2
8724	GO:0036467	1	0.997978943	0	1
8725	GO:0036468	1	0.997978943	0	1
8726	GO:0036470	1	0.997958946	0	1
8727	GO:0036471	1	0.997958946	0	1
8728	GO:0036473	1	0.997997923	0	1
8729	GO:0036474	1	0.995973701	0	2
8730	GO:0036475	1	0.991982207	0	4
8731	GO:0036477	1	0.978205422	0	11
8732	GO:0036478	1	0.997958946	0	1
8733	GO:0036479	1	0.998013437	0	1
8734	GO:0036481	1	0.995950936	0	2
8735	GO:0036486	1	0.992038585	0	4

8736	GO:0036487	1	0.998013437	0	1
8737	GO:0036488	1	0.995938269	0	2
8738	GO:0036492	1	0.996029163	0	2
8739	GO:0036493	1	0.992010713	0	4
8740	GO:0036494	1	0.998013423	0	1
8741	GO:0036496	1	0.995958086	0	2
8742	GO:0036498	1	0.897149327	0	54
8743	GO:0036499	1	0.978073703	0	11
8744	GO:0036500	1	0.9820478	0	9
8745	GO:0036501	1	0.996005047	0	2
8746	GO:0036502	1	0.995990914	0	2
8747	GO:0036503	1	0.968386042	0	16
8748	GO:0036505	1	0.995983521	0	2
8749	GO:0036508	1	0.99799034	0	1
8750	GO:0036509	1	0.99597156	0	2
8751	GO:0036510	1	0.993992818	0	3
8752	GO:0036511	1	0.99597156	0	2
8753	GO:0036512	1	0.99597156	0	2
8754	GO:0036513	1	0.984088106	0	8
8755	GO:0036514	1	0.99800669	0	1
8756	GO:0036515	1	0.99800669	0	1
8757	GO:0036517	1	0.998013437	0	1
8758	GO:0036518	1	0.996010868	0	2
8759	GO:0036520	1	0.99800067	0	1
8760	GO:0036524	1	0.997958946	0	1
8761	GO:0036525	1	0.99796864	0	1
8762	GO:0036526	1	0.997958946	0	1
8763	GO:0036527	1	0.997958946	0	1
8764	GO:0036528	1	0.997958946	0	1
8765	GO:0036529	1	0.997958946	0	1
8766	GO:0036530	1	0.997958946	0	1
8767	GO:0036531	1	0.997958946	0	1
8768	GO:0038001	1	0.986040237	0	7
8769	GO:0038002	1	0.998013335	0	1
8770	GO:0038003	1	0.983964747	0	8
8771	GO:0038007	1	0.984121851	0	8
8772	GO:0038008	1	0.998003011	0	1
8773	GO:0038016	1	0.993986194	0	3
8774	GO:0038018	1	0.996028766	0	2
8775	GO:0038020	1	0.998013437	0	1
8776	GO:0038021	1	0.998009745	0	1
8777	GO:0038023	1	0.691628513	0	183
8778	GO:0038024	1	0.972424204	0	14
8779	GO:0038025	1	0.996016467	0	2
8780	GO:0038026	1	0.982209992	0	9

8781	GO:0038027	1	0.99006901	0	5
8782	GO:0038028	1	0.992025478	0	4
8783	GO:0038029	1	0.998012649	0	1
8784	GO:0038030	1	0.998006585	0	1
8785	GO:0038031	1	0.992053809	0	4
8786	GO:0038033	1	0.990015286	0	5
8787	GO:0038036	1	0.98601586	0	7
8788	GO:0038037	1	0.996027731	0	2
8789	GO:0038038	1	0.998013437	0	1
8790	GO:0038039	1	0.994044673	0	3
8791	GO:0038041	1	0.998003243	0	1
8792	GO:0038043	1	0.995995432	0	2
8793	GO:0038046	1	0.997974731	0	1
8794	GO:0038047	1	0.997976331	0	1
8795	GO:0038048	1	0.998012797	0	1
8796	GO:0038060	1	0.995994003	0	2
8797	GO:0038061	1	0.880148135	0	63
8798	GO:0038062	1	0.994001822	0	3
8799	GO:0038063	1	0.978308978	0	11
8800	GO:0038064	1	0.994006388	0	3
8801	GO:0038065	1	0.992031463	0	4
8802	GO:0038066	1	0.987992137	0	6
8803	GO:0038083	1	0.968497611	0	16
8804	GO:0038084	1	0.970392575	0	15
8805	GO:0038085	1	0.990036425	0	5
8806	GO:0038089	1	0.992064222	0	4
8807	GO:0038091	1	0.994040445	0	3
8808	GO:0038092	1	0.986066222	0	7
8809	GO:0038093	1	0.991989706	0	4
8810	GO:0038094	1	0.995924348	0	2
8811	GO:0038095	1	0.802459481	0	109
8812	GO:0038096	1	0.872275383	0	68
8813	GO:0038097	1	0.998013301	0	1
8814	GO:0038098	1	0.988035081	0	6
8815	GO:0038100	1	0.993993845	0	3
8816	GO:0038101	1	0.99797398	0	1
8817	GO:0038102	1	0.995980313	0	2
8818	GO:0038108	1	0.991990786	0	4
8819	GO:0038109	1	0.996010907	0	2
8820	GO:0038110	1	0.980222562	0	10
8821	GO:0038111	1	0.943369099	0	29
8822	GO:0038112	1	0.997990719	0	1
8825	GO:0038115	1	0.997981373	0	1
8826	GO:0038116	1	0.994017856	0	3
8827	GO:0038117	1	0.997981373	0	1

8828	GO:0038121	1	0.997981373	0	1
8829	GO:0038123	1	0.991978048	0	4
8830	GO:0038124	1	0.991988622	0	4
8831	GO:0038127	1	0.992017677	0	4
8832	GO:0038128	1	0.939708738	0	31
8833	GO:0038129	1	0.997984757	0	1
8834	GO:0038130	1	0.997984757	0	1
8835	GO:0038131	1	0.995979849	0	2
8836	GO:0038132	1	0.990090325	0	5
8837	GO:0038143	1	0.996014817	0	2
8838	GO:0038145	1	0.992005728	0	4
8839	GO:0038146	1	0.996013911	0	2
8840	GO:0038147	1	0.99797487	0	1
8841	GO:0038154	1	0.993991629	0	3
8842	GO:0038155	1	0.984055587	0	8
8843	GO:0038156	1	0.991974956	0	4
8844	GO:0038158	1	0.995977914	0	2
8845	GO:0038160	1	0.99797487	0	1
8846	GO:0038161	1	0.998013437	0	1
8847	GO:0038162	1	0.995993198	0	2
8848	GO:0038163	1	0.992001136	0	4
8849	GO:0038164	1	0.998003662	0	1
8850	GO:0038165	1	0.988048751	0	6
8851	GO:0038166	1	0.989959729	0	5
8852	GO:0038168	1	0.998013336	0	1
8853	GO:0038170	1	0.997993743	0	1
8854	GO:0038171	1	0.994005619	0	3
8855	GO:0038172	1	0.993989518	0	3
8856	GO:0038173	1	0.997989208	0	1
8857	GO:0038177	1	0.997963878	0	1
8858	GO:0038178	1	0.995978601	0	2
8859	GO:0038179	1	0.996009765	0	2
8860	GO:0038180	1	0.978125299	0	11
8861	GO:0038181	1	0.997969714	0	1
8862	GO:0038182	1	0.997969714	0	1
8863	GO:0038183	1	0.980172605	0	10
8864	GO:0038184	1	0.997969714	0	1
8865	GO:0038186	1	0.998012112	0	1
8866	GO:0038187	1	0.978198401	0	11
8867	GO:0038189	1	0.993992971	0	3
8868	GO:0038190	1	0.995992287	0	2
8869	GO:0038191	1	0.990085038	0	5
8870	GO:0038192	1	0.99797915	0	1
8871	GO:0038193	1	0.997982988	0	1
8872	GO:0038194	1	0.995975291	0	2

8873	GO:0038195	1	0.995923632	0	2
8874	GO:0038202	1	0.984147296	0	8
8875	GO:0038203	1	0.986043591	0	7
8876	GO:0039003	1	0.998006243	0	1
8877	GO:0039008	1	0.998013416	0	1
8878	GO:0039019	1	0.998003548	0	1
8879	GO:0039020	1	0.997991284	0	1
8880	GO:0039023	1	0.998013416	0	1
8881	GO:0039520	1	0.99598597	0	2
8882	GO:0039521	1	0.998012836	0	1
8886	GO:0039532	1	0.997979792	0	1
8888	GO:0039535	1	0.994014122	0	3
8890	GO:0039552	1	0.994018276	0	3
8891	GO:0039563	1	0.998013208	0	1
8892	GO:0039564	1	0.998013208	0	1
8893	GO:0039650	1	0.99799068	0	1
8894	GO:0039654	1	0.997990564	0	1
8895	GO:0039689	1	0.99399117	0	3
8896	GO:0039692	1	0.997973337	0	1
8897	GO:0039694	1	0.982122625	0	9
8898	GO:0039695	1	0.998011759	0	1
8899	GO:0039702	1	0.960434256	0	20
8900	GO:0039706	1	0.974125132	0	13
8901	GO:0040001	1	0.990073877	0	5
8902	GO:0040008	1	0.866703132	0	71
8903	GO:0040009	1	0.996000683	0	2
8904	GO:0040010	1	0.993946828	0	3
8905	GO:0040011	1	0.982153768	0	9
8906	GO:0040012	1	0.985988824	0	7
8907	GO:0040013	1	0.995946135	0	2
8908	GO:0040014	1	0.954861729	0	23
8909	GO:0040015	1	0.978090382	0	11
8910	GO:0040016	1	0.988028636	0	6
8911	GO:0040017	1	0.996030048	0	2
8912	GO:0040018	1	0.953007563	0	24
8913	GO:0040019	1	0.972272565	0	14
8914	GO:0040020	1	0.987981075	0	6
8915	GO:0040029	1	0.968401375	0	16
8916	GO:0040030	1	0.998005373	0	1
8917	GO:0040031	1	0.997990433	0	1
8918	GO:0040034	1	0.997966097	0	1
8919	GO:0040036	1	0.990051975	0	5
8920	GO:0040037	1	0.968492098	0	16
8921	GO:0040038	1	0.992038904	0	4
8922	GO:0040040	1	0.998013437	0	1

8923	GO:0042007	1	0.995974338	0	2
8924	GO:0042008	1	0.998002011	0	1
8925	GO:0042010	1	0.993964411	0	3
8926	GO:0042011	1	0.997996022	0	1
8927	GO:0042012	1	0.997996022	0	1
8928	GO:0042015	1	0.993986463	0	3
8929	GO:0042019	1	0.993980165	0	3
8930	GO:0042020	1	0.99597391	0	2
8931	GO:0042022	1	0.995980712	0	2
8932	GO:0042023	1	0.997990564	0	1
8933	GO:0042026	1	0.962397196	0	19
8934	GO:0042030	1	0.99205412	0	4
8935	GO:0042038	1	0.997990564	0	1
8936	GO:0042043	1	0.970430104	0	15
8937	GO:0042044	1	0.994012235	0	3
8938	GO:0042045	1	0.986027037	0	7
8939	GO:0042048	1	0.988031385	0	6
8940	GO:0042053	1	0.990006843	0	5
8944	GO:0042059	1	0.930184777	0	36
8945	GO:0042060	1	0.818045053	0	100
8946	GO:0042063	1	0.984013043	0	8
8947	GO:0042073	1	0.941478058	0	30
8948	GO:0042074	1	0.988026101	0	6
8949	GO:0042088	1	0.981924061	0	9
8950	GO:0042092	1	0.991963357	0	4
8951	GO:0042093	1	0.99402082	0	3
8952	GO:0042098	1	0.960609595	0	20
8953	GO:0042100	1	0.964410944	0	18
8954	GO:0042101	1	0.974099456	0	13
8955	GO:0042102	1	0.905846144	0	49
8956	GO:0042104	1	0.958500885	0	21
8957	GO:0042105	1	0.991897198	0	4
8958	GO:0042110	1	0.913521084	0	45
8959	GO:0042113	1	0.937717224	0	32
8960	GO:0042116	1	0.978158816	0	11
8961	GO:0042117	1	0.983958703	0	8
8963	GO:0042119	1	0.976063984	0	12
8964	GO:0042126	1	0.993963012	0	3
8966	GO:0042129	1	0.980145594	0	10
8967	GO:0042130	1	0.928083011	0	37
8968	GO:0042131	1	0.997990564	0	1
8969	GO:0042132	1	0.99594082	0	2
8970	GO:0042133	1	0.991974348	0	4
8971	GO:0042134	1	0.993942123	0	3
8972	GO:0042135	1	0.984100686	0	8

8973	GO:0042136	1	0.986069515	0	7
8974	GO:0042138	1	0.989972008	0	5
8975	GO:0042144	1	0.997987032	0	1
8976	GO:0042147	1	0.863551242	0	73
8977	GO:0042148	1	0.991975291	0	4
8978	GO:0042149	1	0.909855416	0	47
8979	GO:0042157	1	0.962559252	0	19
8980	GO:0042158	1	0.989955683	0	5
8981	GO:0042159	1	0.990023696	0	5
8982	GO:0042162	1	0.951086844	0	25
8983	GO:0042163	1	0.997968714	0	1
8984	GO:0042165	1	0.99198985	0	4
8985	GO:0042166	1	0.980094397	0	10
8986	GO:0042167	1	0.972165818	0	14
8987	GO:0042168	1	0.989937922	0	5
8988	GO:0042169	1	0.928525587	0	37
8989	GO:0042171	1	0.988056878	0	6
8990	GO:0042175	1	0.994031808	0	3
8991	GO:0042176	1	0.964424972	0	18
8992	GO:0042177	1	0.931958665	0	35
8993	GO:0042178	1	0.983973346	0	8
8994	GO:0042180	1	0.993985404	0	3
8995	GO:0042182	1	0.998002608	0	1
8996	GO:0042197	1	0.997972955	0	1
8997	GO:0042218	1	0.997979183	0	1
8998	GO:0042219	1	0.997990564	0	1
8999	GO:0042220	1	0.928468757	0	37
9000	GO:0042245	1	0.996001845	0	2
9001	GO:0042246	1	0.960378616	0	20
9002	GO:0042247	1	0.997993458	0	1
9003	GO:0042249	1	0.994020151	0	3
9004	GO:0042254	1	0.818824862	0	99
9005	GO:0042255	1	0.986046955	0	7
9006	GO:0042256	1	0.989940967	0	5
9007	GO:0042262	1	0.989882957	0	5
9008	GO:0042264	1	0.996020741	0	2
9009	GO:0042265	1	0.998013437	0	1
9010	GO:0042267	1	0.960382353	0	20
9011	GO:0042269	1	0.995966947	0	2
9012	GO:0042270	1	0.987928869	0	6
9013	GO:0042271	1	0.994025451	0	3
9014	GO:0042272	1	0.993992316	0	3
9015	GO:0042273	1	0.940897874	0	30
9016	GO:0042274	1	0.956303787	0	22
9017	GO:0042275	1	0.997970247	0	1

9018	GO:0042276	1	0.958541698	0	21
9019	GO:0042277	1	0.896930446	0	54
9020	GO:0042278	1	0.991976795	0	4
9021	GO:0042281	1	0.998000023	0	1
9022	GO:0042282	1	0.998011252	0	1
9023	GO:0042283	1	0.997973716	0	1
9024	GO:0042284	1	0.995948206	0	2
9025	GO:0042285	1	0.995985075	0	2
9026	GO:0042287	1	0.99000737	0	5
9027	GO:0042288	1	0.966372252	0	17
9028	GO:0042289	1	0.989963292	0	5
9029	GO:0042292	1	0.997985905	0	1
9031	GO:0042297	1	0.992055576	0	4
9032	GO:0042300	1	0.998009713	0	1
9033	GO:0042301	1	0.978183275	0	11
9034	GO:0042304	1	0.982139156	0	9
9035	GO:0042306	1	0.986008482	0	7
9036	GO:0042307	1	0.922832898	0	40
9037	GO:0042308	1	0.976132036	0	12
9038	GO:0042310	1	0.974231079	0	13
9039	GO:0042311	1	0.956746414	0	22
9040	GO:0042313	1	0.99798021	0	1
9041	GO:0042320	1	0.998013436	0	1
9042	GO:0042321	1	0.995984919	0	2
9043	GO:0042322	1	0.997954504	0	1
9044	GO:0042323	1	0.995966445	0	2
9045	GO:0042325	1	0.964409383	0	18
9046	GO:0042326	1	0.962390156	0	19
9047	GO:0042327	1	0.941470237	0	30
9048	GO:0042328	1	0.996008274	0	2
9049	GO:0042335	1	0.994020639	0	3
9050	GO:0042339	1	0.997989999	0	1
9051	GO:0042340	1	0.982089689	0	9
9052	GO:0042351	1	0.995967607	0	2
9053	GO:0042352	1	0.997990564	0	1
9054	GO:0042355	1	0.986110197	0	7
9055	GO:0042356	1	0.997990564	0	1
9056	GO:0042357	1	0.997975129	0	1
9057	GO:0042359	1	0.978121626	0	11
9058	GO:0042360	1	0.99395921	0	3
9059	GO:0042361	1	0.991973895	0	4
9060	GO:0042368	1	0.995962448	0	2
9061	GO:0042369	1	0.993986226	0	3
9062	GO:0042371	1	0.998003649	0	1
9063	GO:0042373	1	0.991914909	0	4

9064	GO:0042376	1	0.989952286	0	5
9065	GO:0042377	1	0.991973895	0	4
9066	GO:0042379	1	0.989850882	0	5
9067	GO:0042382	1	0.990040596	0	5
9069	GO:0042391	1	0.837931505	0	88
9070	GO:0042392	1	0.985998749	0	7
9072	GO:0042403	1	0.980126344	0	10
9073	GO:0042404	1	0.998013437	0	1
9074	GO:0042405	1	0.976316876	0	12
9075	GO:0042406	1	0.986003257	0	7
9076	GO:0042407	1	0.939446191	0	31
9077	GO:0042412	1	0.997989828	0	1
9078	GO:0042413	1	0.997986532	0	1
9079	GO:0042414	1	0.998013437	0	1
9080	GO:0042415	1	0.988005288	0	6
9081	GO:0042416	1	0.988021518	0	6
9082	GO:0042417	1	0.978146522	0	11
9083	GO:0042418	1	0.997963324	0	1
9084	GO:0042420	1	0.989984045	0	5
9085	GO:0042421	1	0.993993886	0	3
9086	GO:0042423	1	0.993939503	0	3
9087	GO:0042424	1	0.997982988	0	1
9088	GO:0042426	1	0.988057537	0	6
9089	GO:0042427	1	0.993950984	0	3
9090	GO:0042428	1	0.990059418	0	5
9091	GO:0042431	1	0.997982764	0	1
9092	GO:0042438	1	0.978099499	0	11
9093	GO:0042445	1	0.978153098	0	11
9094	GO:0042446	1	0.980130959	0	10
9095	GO:0042447	1	0.994037711	0	3
9096	GO:0042448	1	0.981937725	0	9
9097	GO:0042450	1	0.997978487	0	1
9098	GO:0042461	1	0.992069687	0	4
9099	GO:0042462	1	0.974279783	0	13
9100	GO:0042470	1	0.817389907	0	100
9101	GO:0042471	1	0.988053649	0	6
9102	GO:0042472	1	0.909908767	0	47
9103	GO:0042473	1	0.989973574	0	5
9104	GO:0042474	1	0.970183394	0	15
9105	GO:0042475	1	0.911677956	0	46
9106	GO:0042476	1	0.937548934	0	32
9107	GO:0042481	1	0.993984352	0	3
9108	GO:0042482	1	0.991972443	0	4
9109	GO:0042487	1	0.987971683	0	6
9110	GO:0042488	1	0.995989812	0	2

9111	GO:0042489	1	0.995984776	0	2
9112	GO:0042490	1	0.993998805	0	3
9113	GO:0042491	1	0.986081365	0	7
9114	GO:0042492	1	0.994024846	0	3
9116	GO:0042494	1	0.996030001	0	2
9117	GO:0042495	1	0.99599623	0	2
9118	GO:0042496	1	0.996017597	0	2
9119	GO:0042497	1	0.998000278	0	1
9120	GO:0042500	1	0.988015275	0	6
9121	GO:0042501	1	0.993983314	0	3
9122	GO:0042509	1	0.991924496	0	4
9123	GO:0042531	1	0.905909744	0	49
9124	GO:0042532	1	0.974230802	0	13
9125	GO:0042538	1	0.993999659	0	3
9126	GO:0042541	1	0.991965256	0	4
9128	GO:0042543	1	0.997951079	0	1
9129	GO:0042551	1	0.978240081	0	11
9130	GO:0042552	1	0.907977785	0	48
9131	GO:0042554	1	0.972153002	0	14
9132	GO:0042555	1	0.976193912	0	12
9133	GO:0042558	1	0.998013437	0	1
9134	GO:0042559	1	0.997977827	0	1
9135	GO:0042562	1	0.982020566	0	9
9136	GO:0042564	1	0.998010369	0	1
9137	GO:0042565	1	0.995978993	0	2
9138	GO:0042567	1	0.9920187	0	4
9139	GO:0042568	1	0.997959584	0	1
9140	GO:0042571	1	0.997990564	0	1
9141	GO:0042572	1	0.916889355	0	43
9142	GO:0042573	1	0.950802426	0	25
9143	GO:0042574	1	0.970267891	0	15
9144	GO:0042575	1	0.997973354	0	1
9145	GO:0042577	1	0.976144169	0	12
9146	GO:0042578	1	0.98604333	0	7
9147	GO:0042581	1	0.974090053	0	13
9148	GO:0042582	1	0.983982092	0	8
9149	GO:0042583	1	0.993973762	0	3
9150	GO:0042584	1	0.984089064	0	8
9151	GO:0042585	1	0.993994708	0	3
9152	GO:0042586	1	0.997963324	0	1
9153	GO:0042587	1	0.986074429	0	7
9154	GO:0042588	1	0.993984279	0	3
9155	GO:0042589	1	0.980053778	0	10
9156	GO:0042590	1	0.989899929	0	5
9157	GO:0042592	1	0.980112781	0	10

9158	GO:0042593	1	0.829156498	0	93
9159	GO:0042594	1	0.935738786	0	33
9160	GO:0042595	1	0.998005942	0	1
9161	GO:0042596	1	0.99198908	0	4
9162	GO:0042599	1	0.989978294	0	5
9163	GO:0042602	1	0.997957401	0	1
9164	GO:0042605	1	0.954577456	0	23
9165	GO:0042608	1	0.97795195	0	11
9166	GO:0042609	1	0.981999777	0	9
9167	GO:0042610	1	0.991926513	0	4
9168	GO:0042612	1	0.983860323	0	8
9169	GO:0042613	1	0.971925776	0	14
9170	GO:0042622	1	0.972259674	0	14
9171	GO:0042625	1	0.993912875	0	3
9172	GO:0042626	1	0.899345214	0	53
9173	GO:0042627	1	0.983888506	0	8
9174	GO:0042628	1	0.99798286	0	1
9175	GO:0042629	1	0.989905442	0	5
9176	GO:0042631	1	0.99403234	0	3
9177	GO:0042632	1	0.871889143	0	68
9178	GO:0042633	1	0.983989688	0	8
9179	GO:0042634	1	0.988057117	0	6
9180	GO:0042635	1	0.996001362	0	2
9181	GO:0042636	1	0.998000767	0	1
9182	GO:0042641	1	0.976339984	0	12
9183	GO:0042645	1	0.913293298	0	45
9184	GO:0042658	1	0.997966363	0	1
9185	GO:0042659	1	0.99801336	0	1
9186	GO:0042660	1	0.998013437	0	1
9187	GO:0042662	1	0.993929081	0	3
9188	GO:0042663	1	0.997975268	0	1
9189	GO:0042664	1	0.997963363	0	1
9190	GO:0042666	1	0.998005981	0	1
9191	GO:0042669	1	0.998004171	0	1
9192	GO:0042692	1	0.974260874	0	13
9193	GO:0042693	1	0.99797054	0	1
9194	GO:0042698	1	0.984029968	0	8
9195	GO:0042699	1	0.995962914	0	2
9196	GO:0042700	1	0.998011767	0	1
9197	GO:0042701	1	0.995998761	0	2
9198	GO:0042704	1	0.996014261	0	2
9199	GO:0042709	1	0.995969712	0	2
9200	GO:0042711	1	0.984115936	0	8
9201	GO:0042713	1	0.992067062	0	4
9202	GO:0042719	1	0.989883796	0	5

9203	GO:0042720	1	0.995930493	0	2
9204	GO:0042721	1	0.991971189	0	4
9205	GO:0042723	1	0.989980042	0	5
9206	GO:0042730	1	0.968197574	0	16
9207	GO:0042731	1	0.99199505	0	4
9208	GO:0042732	1	0.993953217	0	3
9209	GO:0042733	1	0.90810811	0	48
9210	GO:0042734	1	0.888592927	0	59
9211	GO:0042737	1	0.987949216	0	6
9212	GO:0042738	1	0.956573314	0	22
9214	GO:0042743	1	0.98588031	0	7
9215	GO:0042744	1	0.962216987	0	19
9216	GO:0042745	1	0.997983347	0	1
9217	GO:0042747	1	0.99597611	0	2
9218	GO:0042748	1	0.998013437	0	1
9219	GO:0042749	1	0.997990804	0	1
9220	GO:0042752	1	0.881226913	0	63
9221	GO:0042753	1	0.986095046	0	7
9222	GO:0042754	1	0.98215161	0	9
9223	GO:0042755	1	0.964415267	0	18
9224	GO:0042756	1	0.991932478	0	4
9225	GO:0042758	1	0.989955933	0	5
9226	GO:0042759	1	0.954622872	0	23
9227	GO:0042760	1	0.988032847	0	6
9228	GO:0042761	1	0.976106753	0	12
9229	GO:0042765	1	0.989981877	0	5
9230	GO:0042766	1	0.992047569	0	4
9231	GO:0042769	1	0.926172871	0	38
9232	GO:0042770	1	0.980112749	0	10
9233	GO:0042771	1	0.941415829	0	30
9234	GO:0042772	1	0.997963003	0	1
9235	GO:0042773	1	0.987973145	0	6
9236	GO:0042774	1	0.997990564	0	1
9237	GO:0042775	1	0.981891816	0	9
9238	GO:0042776	1	0.962464244	0	19
9239	GO:0042779	1	0.995942551	0	2
9240	GO:0042780	1	0.99198045	0	4
9241	GO:0042781	1	0.995991973	0	2
9242	GO:0042788	1	0.935083807	0	33
9243	GO:0042789	1	0.976258981	0	12
9244	GO:0042790	1	0.993984444	0	3
9245	GO:0042791	1	0.98802336	0	6
9246	GO:0042795	1	0.870041927	0	69
9247	GO:0042796	1	0.984023897	0	8
9248	GO:0042797	1	0.986004991	0	7

9249	GO:0042799	1	0.991997615	0	4
9253	GO:0042805	1	0.976223938	0	12
9254	GO:0042806	1	0.989940487	0	5
9255	GO:0042809	1	0.970370487	0	15
9256	GO:0042813	1	0.970443924	0	15
9257	GO:0042816	1	0.994039078	0	3
9258	GO:0042823	1	0.998001224	0	1
9259	GO:0042824	1	0.984019003	0	8
9260	GO:0042825	1	0.996005446	0	2
9261	GO:0042826	1	0.804768101	0	108
9262	GO:0042827	1	0.998010073	0	1
9263	GO:0042832	1	0.966361054	0	17
9264	GO:0042834	1	0.978056109	0	11
9265	GO:0042835	1	0.996024049	0	2
9266	GO:0042840	1	0.997969391	0	1
9267	GO:0042843	1	0.997961917	0	1
9268	GO:0042851	1	0.998006784	0	1
9269	GO:0042853	1	0.99396719	0	3
9270	GO:0042866	1	0.989955044	0	5
9271	GO:0042883	1	0.998004621	0	1
9272	GO:0042886	1	0.997998744	0	1
9273	GO:0042887	1	0.997998744	0	1
9274	GO:0042903	1	0.995990321	0	2
9275	GO:0042904	1	0.993974477	0	3
9276	GO:0042908	1	0.947365657	0	27
9277	GO:0042910	1	0.9665238	0	17
9278	GO:0042921	1	0.988028514	0	6
9279	GO:0042922	1	0.997956554	0	1
9280	GO:0042923	1	0.974145117	0	13
9281	GO:0042924	1	0.997998452	0	1
9282	GO:0042930	1	0.997990564	0	1
9283	GO:0042931	1	0.997990564	0	1
9284	GO:0042937	1	0.997996551	0	1
9285	GO:0042938	1	0.998013399	0	1
9286	GO:0042942	1	0.995986176	0	2
9287	GO:0042945	1	0.997994328	0	1
9288	GO:0042946	1	0.997990564	0	1
9289	GO:0042947	1	0.997990564	0	1
9290	GO:0042953	1	0.972355877	0	14
9291	GO:0042974	1	0.968455931	0	16
9292	GO:0042975	1	0.976263729	0	12
9293	GO:0042976	1	0.990082412	0	5
9294	GO:0042978	1	0.996004346	0	2
9296	GO:0042982	1	0.978082038	0	11
9297	GO:0042983	1	0.997993543	0	1

9298	GO:0042984	1	0.993975403	0	3
9299	GO:0042985	1	0.987990982	0	6
9300	GO:0042986	1	0.992008626	0	4
9301	GO:0042987	1	0.972310759	0	14
9302	GO:0042988	1	0.998013182	0	1
9303	GO:0042989	1	0.979869051	0	10
9304	GO:0042994	1	0.982054206	0	9
9306	GO:0042997	1	0.989947261	0	5
9307	GO:0042998	1	0.988015307	0	6
9308	GO:0043000	1	0.995986234	0	2
9309	GO:0043001	1	0.945302769	0	28
9310	GO:0043004	1	0.998011615	0	1
9311	GO:0043005	1	0.456955758	0	387
9312	GO:0043006	1	0.997987593	0	1
9313	GO:0043007	1	0.995991274	0	2
9314	GO:0043008	1	0.984030529	0	8
9315	GO:0043009	1	0.990044193	0	5
9316	GO:0043010	1	0.900754854	0	52
9317	GO:0043011	1	0.96821055	0	16
9318	GO:0043012	1	0.997988091	0	1
9319	GO:0043014	1	0.926566037	0	38
9320	GO:0043015	1	0.939723767	0	31
9321	GO:0043020	1	0.978062434	0	11
9322	GO:0043021	1	0.931983839	0	35
9323	GO:0043022	1	0.880967979	0	63
9324	GO:0043023	1	0.972203394	0	14
9325	GO:0043024	1	0.966351109	0	17
9326	GO:0043025	1	0.477926277	0	365
9327	GO:0043027	1	0.966368423	0	17
9328	GO:0043028	1	0.997993018	0	1
9329	GO:0043029	1	0.947129203	0	27
9330	GO:0043030	1	0.980089287	0	10
9331	GO:0043031	1	0.985950622	0	7
9332	GO:0043032	1	0.966313103	0	17
9333	GO:0043033	1	0.998013437	0	1
9334	GO:0043034	1	0.962674373	0	19
9335	GO:0043035	1	0.990065891	0	5
9336	GO:0043039	1	0.972214025	0	14
9337	GO:0043041	1	0.998002608	0	1
9338	GO:0043043	1	0.988050812	0	6
9339	GO:0043044	1	0.936069779	0	33
9340	GO:0043045	1	0.990010573	0	5
9341	GO:0043046	1	0.964481375	0	18
9342	GO:0043047	1	0.990065748	0	5
9343	GO:0043048	1	0.997981857	0	1

9344	GO:0043049	1	0.99799543	0	1
9345	GO:0043060	1	0.997986141	0	1
9346	GO:0043062	1	0.998013437	0	1
9347	GO:0043063	1	0.998012648	0	1
9350	GO:0043067	1	0.990002939	0	5
9351	GO:0043068	1	0.98205214	0	9
9352	GO:0043069	1	0.974264818	0	13
9353	GO:0043083	1	0.968456683	0	16
9354	GO:0043084	1	0.99602601	0	2
9355	GO:0043085	1	0.937598072	0	32
9356	GO:0043086	1	0.902112824	0	51
9357	GO:0043087	1	0.835057989	0	90
9358	GO:0043097	1	0.977996111	0	11
9360	GO:0043103	1	0.99594291	0	2
9361	GO:0043105	1	0.997954463	0	1
9362	GO:0043111	1	0.992027234	0	4
9363	GO:0043112	1	0.99799873	0	1
9364	GO:0043113	1	0.949257614	0	26
9365	GO:0043114	1	0.984123206	0	8
9366	GO:0043116	1	0.968450385	0	16
9367	GO:0043117	1	0.980071702	0	10
9368	GO:0043120	1	0.990031072	0	5
9369	GO:0043121	1	0.994017925	0	3
9370	GO:0043122	1	0.945220219	0	28
9371	GO:0043123	1	0.69672595	0	179
9373	GO:0043125	1	0.990011574	0	5
9374	GO:0043128	1	0.997969861	0	1
9375	GO:0043129	1	0.974163172	0	13
9376	GO:0043130	1	0.84609926	0	83
9377	GO:0043136	1	0.995978994	0	2
9378	GO:0043137	1	0.991952676	0	4
9379	GO:0043138	1	0.97242533	0	14
9380	GO:0043139	1	0.98403363	0	8
9381	GO:0043149	1	0.968415404	0	16
9382	GO:0043152	1	0.991956397	0	4
9383	GO:0043153	1	0.945239553	0	28
9385	GO:0043159	1	0.993938927	0	3
9387	GO:0043162	1	0.962484472	0	19
9388	GO:0043163	1	0.99400572	0	3
9389	GO:0043167	1	0.995985075	0	2
9390	GO:0043169	1	0.966422093	0	17
9391	GO:0043170	1	0.997964698	0	1
9392	GO:0043171	1	0.970461414	0	15
9393	GO:0043175	1	0.995990703	0	2
9394	GO:0043177	1	0.987822505	0	6

9395	GO:0043178	1	0.995941804	0	2
9396	GO:0043179	1	0.99798021	0	1
9397	GO:0043181	1	0.998003076	0	1
9398	GO:0043183	1	0.993958068	0	3
9399	GO:0043184	1	0.982149765	0	9
9400	GO:0043185	1	0.995974471	0	2
9401	GO:0043186	1	0.970362208	0	15
9402	GO:0043189	1	0.997990564	0	1
9403	GO:0043190	1	0.990043896	0	5
9404	GO:0043194	1	0.96851659	0	16
9405	GO:0043195	1	0.917345354	0	43
9406	GO:0043196	1	0.98599024	0	7
9407	GO:0043197	1	0.739718652	0	150
9408	GO:0043198	1	0.937903784	0	32
9409	GO:0043199	1	0.991983632	0	4
9410	GO:0043200	1	0.945164785	0	28
9411	GO:0043201	1	0.996013002	0	2
9412	GO:0043202	1	0.844340005	0	84
9413	GO:0043203	1	0.98402222	0	8
9414	GO:0043204	1	0.760536804	0	136
9415	GO:0043208	1	0.992013307	0	4
9416	GO:0043209	1	0.943442944	0	29
9417	GO:0043217	1	0.990018378	0	5
9418	GO:0043218	1	0.993939839	0	3
9419	GO:0043219	1	0.991995409	0	4
9420	GO:0043220	1	0.980088466	0	10
9421	GO:0043221	1	0.997990564	0	1
9422	GO:0043227	1	0.990042575	0	5
9423	GO:0043229	1	0.968334019	0	16
9425	GO:0043235	1	0.694891919	0	181
9426	GO:0043236	1	0.952986291	0	24
9427	GO:0043237	1	0.990053096	0	5
9428	GO:0043240	1	0.972238514	0	14
9429	GO:0043242	1	0.99400671	0	3
9430	GO:0043243	1	0.989994729	0	5
9431	GO:0043244	1	0.998013437	0	1
9432	GO:0043247	1	0.996028851	0	2
9433	GO:0043248	1	0.975970981	0	12
9434	GO:0043249	1	0.978159966	0	11
9435	GO:0043250	1	0.997969916	0	1
9436	GO:0043251	1	0.997969916	0	1
9437	GO:0043252	1	0.974289298	0	13
9438	GO:0043254	1	0.958658821	0	21
9439	GO:0043256	1	0.98416634	0	8
9440	GO:0043257	1	0.998013437	0	1

9441	GO:0043259	1	0.994051875	0	3
9442	GO:0043260	1	0.994051875	0	3
9443	GO:0043262	1	0.996001745	0	2
9444	GO:0043265	1	0.99799652	0	1
9445	GO:0043266	1	0.992009024	0	4
9446	GO:0043267	1	0.992010837	0	4
9447	GO:0043268	1	0.984143778	0	8
9448	GO:0043269	1	0.987858704	0	6
9449	GO:0043270	1	0.991997056	0	4
9450	GO:0043271	1	0.994001954	0	3
9451	GO:0043273	1	0.994031768	0	3
9452	GO:0043274	1	0.956810095	0	22
9453	GO:0043276	1	0.978185781	0	11
9454	GO:0043277	1	0.960630819	0	20
9455	GO:0043278	1	0.950966868	0	25
9456	GO:0043279	1	0.986029895	0	7
9457	GO:0043280	1	0.902355588	0	51
9458	GO:0043281	1	0.976055485	0	12
9459	GO:0043291	1	0.994007787	0	3
9460	GO:0043292	1	0.978049899	0	11
9461	GO:0043293	1	0.995991961	0	2
9462	GO:0043295	1	0.97796596	0	11
9463	GO:0043296	1	0.956848544	0	22
9464	GO:0043297	1	0.984082945	0	8
9465	GO:0043299	1	0.997981211	0	1
9466	GO:0043303	1	0.970330849	0	15
9467	GO:0043304	1	0.982083456	0	9
9468	GO:0043305	1	0.991933659	0	4
9469	GO:0043306	1	0.988056163	0	6
9470	GO:0043308	1	0.995973222	0	2
9471	GO:0043311	1	0.99596433	0	2
9472	GO:0043312	1	0.408439315	0	440
9473	GO:0043313	1	0.995998211	0	2
9474	GO:0043315	1	0.992022616	0	4
9475	GO:0043316	1	0.997991323	0	1
9476	GO:0043318	1	0.995977064	0	2
9477	GO:0043320	1	0.991985932	0	4
9478	GO:0043322	1	0.99796583	0	1
9479	GO:0043323	1	0.99196133	0	4
9480	GO:0043325	1	0.949144603	0	26
9481	GO:0043328	1	0.989931821	0	5
9483	GO:0043331	1	0.993989334	0	3
9484	GO:0043333	1	0.997970045	0	1
9485	GO:0043335	1	0.993959347	0	3
9486	GO:0043337	1	0.99800547	0	1

9487	GO:0043353	1	0.990000275	0	5
9488	GO:0043366	1	0.993979695	0	3
9490	GO:0043368	1	0.994025007	0	3
9491	GO:0043369	1	0.995968904	0	2
9492	GO:0043370	1	0.994023541	0	3
9493	GO:0043371	1	0.992043567	0	4
9494	GO:0043372	1	0.990001457	0	5
9495	GO:0043373	1	0.996003241	0	2
9496	GO:0043374	1	0.993982939	0	3
9497	GO:0043375	1	0.996018623	0	2
9498	GO:0043376	1	0.998003192	0	1
9499	GO:0043377	1	0.995971612	0	2
9500	GO:0043378	1	0.99004539	0	5
9501	GO:0043379	1	0.998013437	0	1
9502	GO:0043380	1	0.998002144	0	1
9503	GO:0043381	1	0.998009752	0	1
9504	GO:0043382	1	0.985942267	0	7
9505	GO:0043383	1	0.998006189	0	1
9506	GO:0043388	1	0.939401035	0	31
9507	GO:0043392	1	0.945145021	0	28
9508	GO:0043393	1	0.94909287	0	26
9509	GO:0043394	1	0.972371733	0	14
9510	GO:0043395	1	0.970315629	0	15
9511	GO:0043398	1	0.991975843	0	4
9512	GO:0043400	1	0.997954504	0	1
9513	GO:0043401	1	0.9549687	0	23
9514	GO:0043402	1	0.998013437	0	1
9515	GO:0043403	1	0.96443032	0	18
9516	GO:0043405	1	0.980134676	0	10
9517	GO:0043406	1	0.90082854	0	52
9518	GO:0043407	1	0.92641332	0	38
9519	GO:0043408	1	0.964479482	0	18
9520	GO:0043409	1	0.954886392	0	23
9521	GO:0043410	1	0.801568209	0	110
9522	GO:0043413	1	0.997982083	0	1
9523	GO:0043415	1	0.993993917	0	3
9524	GO:0043416	1	0.997990564	0	1
9525	GO:0043417	1	0.997970576	0	1
9526	GO:0043418	1	0.993984251	0	3
9527	GO:0043420	1	0.993960353	0	3
9528	GO:0043422	1	0.976158477	0	12
9529	GO:0043423	1	0.998010325	0	1
9530	GO:0043425	1	0.960646747	0	20
9531	GO:0043426	1	0.990047707	0	5
9532	GO:0043430	1	0.997970045	0	1

9533	GO:0043433	1	0.882681333	0	62
9535	GO:0043438	1	0.997992492	0	1
9536	GO:0043456	1	0.99596833	0	2
9537	GO:0043457	1	0.984092571	0	8
9538	GO:0043461	1	0.997970868	0	1
9539	GO:0043462	1	0.984009707	0	8
9540	GO:0043471	1	0.998012241	0	1
9541	GO:0043473	1	0.937821207	0	32
9542	GO:0043474	1	0.997990564	0	1
9543	GO:0043482	1	0.996030731	0	2
9544	GO:0043484	1	0.911851352	0	46
9545	GO:0043486	1	0.982055527	0	9
9546	GO:0043488	1	0.790847141	0	116
9547	GO:0043489	1	0.99193538	0	4
9548	GO:0043490	1	0.99199941	0	4
9549	GO:0043491	1	0.933985587	0	34
9550	GO:0043495	1	0.970231344	0	15
9551	GO:0043503	1	0.995944317	0	2
9552	GO:0043504	1	0.988046361	0	6
9553	GO:0043506	1	0.99397767	0	3
9554	GO:0043507	1	0.937913095	0	32
9555	GO:0043508	1	0.974177549	0	13
9556	GO:0043509	1	0.997981405	0	1
9557	GO:0043512	1	0.995953792	0	2
9558	GO:0043513	1	0.997968378	0	1
9559	GO:0043514	1	0.997968714	0	1
9560	GO:0043515	1	0.988028898	0	6
9561	GO:0043516	1	0.989955722	0	5
9562	GO:0043517	1	0.976157744	0	12
9563	GO:0043518	1	0.972192948	0	14
9564	GO:0043519	1	0.997980382	0	1
9565	GO:0043522	1	0.978038163	0	11
9566	GO:0043523	1	0.960586555	0	20
9567	GO:0043524	1	0.772586758	0	128
9568	GO:0043525	1	0.902292596	0	51
9569	GO:0043527	1	0.995944175	0	2
9570	GO:0043529	1	0.993974803	0	3
9571	GO:0043530	1	0.995960388	0	2
9572	GO:0043531	1	0.930320579	0	36
9573	GO:0043532	1	0.992008642	0	4
9574	GO:0043533	1	0.990052218	0	5
9575	GO:0043534	1	0.980080262	0	10
9576	GO:0043535	1	0.988028575	0	6
9577	GO:0043536	1	0.928374907	0	37
9578	GO:0043537	1	0.960574324	0	20

9579	GO:0043538	1	0.997995094	0	1
9580	GO:0043539	1	0.941536209	0	30
9581	GO:0043540	1	0.997974207	0	1
9582	GO:0043541	1	0.993971966	0	3
9583	GO:0043542	1	0.941496872	0	30
9584	GO:0043543	1	0.997981454	0	1
9585	GO:0043544	1	0.998002843	0	1
9586	GO:0043545	1	0.997990293	0	1
9587	GO:0043546	1	0.988059641	0	6
9588	GO:0043547	1	0.526432357	0	318
9589	GO:0043548	1	0.958785928	0	21
9590	GO:0043549	1	0.990064207	0	5
9591	GO:0043550	1	0.992030371	0	4
9592	GO:0043551	1	0.968441861	0	16
9593	GO:0043552	1	0.945503437	0	28
9594	GO:0043553	1	0.992001362	0	4
9595	GO:0043555	1	0.996014312	0	2
9596	GO:0043558	1	0.998009026	0	1
9597	GO:0043559	1	0.990091739	0	5
9598	GO:0043560	1	0.980225215	0	10
9599	GO:0043564	1	0.993984232	0	3
9601	GO:0043567	1	0.987937988	0	6
9602	GO:0043568	1	0.984115616	0	8
9603	GO:0043569	1	0.990039776	0	5
9604	GO:0043570	1	0.99204879	0	4
9605	GO:0043576	1	0.990039084	0	5
9606	GO:0043583	1	0.978161171	0	11
9607	GO:0043584	1	0.988066113	0	6
9608	GO:0043585	1	0.994030546	0	3
9609	GO:0043586	1	0.984125738	0	8
9610	GO:0043587	1	0.991994152	0	4
9611	GO:0043588	1	0.915531635	0	44
9612	GO:0043589	1	0.978283637	0	11
9613	GO:0043596	1	0.976235475	0	12
9614	GO:0043602	1	0.997986766	0	1
9615	GO:0043603	1	0.997976314	0	1
9616	GO:0043605	1	0.997967328	0	1
9617	GO:0043610	1	0.998013437	0	1
9618	GO:0043614	1	0.9960262	0	2
9619	GO:0043615	1	0.991971367	0	4
9620	GO:0043616	1	0.97813852	0	11
9621	GO:0043618	1	0.993943931	0	3
9622	GO:0043619	1	0.988044052	0	6
9623	GO:0043620	1	0.988046177	0	6
9625	GO:0043622	1	0.992042931	0	4

9626	GO:0043624	1	0.997984757	0	1
9627	GO:0043625	1	0.991958522	0	4
9628	GO:0043626	1	0.997966828	0	1
9629	GO:0043627	1	0.895119485	0	55
9630	GO:0043629	1	0.997990564	0	1
9631	GO:0043630	1	0.997990564	0	1
9632	GO:0043631	1	0.988062817	0	6
9633	GO:0043647	1	0.911868282	0	46
9634	GO:0043648	1	0.991927743	0	4
9635	GO:0043649	1	0.995939606	0	2
9636	GO:0043651	1	0.960458553	0	20
9637	GO:0043652	1	0.968384115	0	16
9638	GO:0043653	1	0.981982758	0	9
9639	GO:0043654	1	0.988004244	0	6
9641	GO:0043666	1	0.913468173	0	45
9642	GO:0043679	1	0.904397076	0	50
9643	GO:0043682	1	0.996030731	0	2
9644	GO:0043686	1	0.993953276	0	3
9645	GO:0043687	1	0.519393623	0	323
9646	GO:0043691	1	0.970147578	0	15
9647	GO:0043697	1	0.992024022	0	4
9648	GO:0043715	1	0.997978835	0	1
9649	GO:0043716	1	0.997978835	0	1
9650	GO:0043727	1	0.99799951	0	1
9651	GO:0043734	1	0.993952279	0	3
9652	GO:0043739	1	0.997998115	0	1
9653	GO:0043754	1	0.998013437	0	1
9654	GO:0043759	1	0.997976212	0	1
9655	GO:0043783	1	0.997984331	0	1
9656	GO:0043795	1	0.997968061	0	1
9657	GO:0043812	1	0.992034181	0	4
9658	GO:0043813	1	0.997996249	0	1
9659	GO:0043843	1	0.997987546	0	1
9660	GO:0043849	1	0.997993118	0	1
9661	GO:0043855	1	0.998003731	0	1
9662	GO:0043874	1	0.997978835	0	1
9663	GO:0043878	1	0.988020826	0	6
9664	GO:0043890	1	0.997984599	0	1
9665	GO:0043914	1	0.998006825	0	1
9666	GO:0043915	1	0.997973557	0	1
9667	GO:0043916	1	0.997961976	0	1
9668	GO:0043921	1	0.997971449	0	1
9669	GO:0043922	1	0.97814406	0	11
9670	GO:0043923	1	0.968419088	0	16
9671	GO:0043924	1	0.998013437	0	1

9672	GO:0043928	1	0.935489994	0	33
9673	GO:0043931	1	0.982191763	0	9
9674	GO:0043932	1	0.991941046	0	4
9675	GO:0043933	1	0.997990564	0	1
9676	GO:0043947	1	0.997974286	0	1
9677	GO:0043949	1	0.98014953	0	10
9678	GO:0043950	1	0.978036559	0	11
9679	GO:0043951	1	0.970413488	0	15
9680	GO:0043956	1	0.997966704	0	1
9681	GO:0043966	1	0.922745289	0	40
9682	GO:0043967	1	0.935858731	0	33
9683	GO:0043968	1	0.970184086	0	15
9684	GO:0043969	1	0.994002514	0	3
9685	GO:0043970	1	0.996004456	0	2
9686	GO:0043972	1	0.99006314	0	5
9687	GO:0043973	1	0.997981946	0	1
9688	GO:0043977	1	0.99799382	0	1
9689	GO:0043979	1	0.997996776	0	1
9690	GO:0043980	1	0.99799382	0	1
9691	GO:0043981	1	0.970297087	0	15
9692	GO:0043982	1	0.970297087	0	15
9693	GO:0043983	1	0.98599125	0	7
9694	GO:0043984	1	0.974268504	0	13
9695	GO:0043985	1	0.987987025	0	6
9696	GO:0043987	1	0.993977814	0	3
9697	GO:0043988	1	0.993969151	0	3
9698	GO:0043990	1	0.997995825	0	1
9699	GO:0043994	1	0.99006314	0	5
9700	GO:0043995	1	0.997990564	0	1
9701	GO:0043996	1	0.997990564	0	1
9702	GO:0043997	1	0.997996279	0	1
9703	GO:0043998	1	0.99799941	0	1
9704	GO:0044003	1	0.993966144	0	3
9705	GO:0044013	1	0.997980545	0	1
9706	GO:0044020	1	0.991951886	0	4
9707	GO:0044027	1	0.997981631	0	1
9708	GO:0044029	1	0.994003641	0	3
9709	GO:0044030	1	0.970385254	0	15
9710	GO:0044053	1	0.997985301	0	1
9711	GO:0044058	1	0.998010369	0	1
9712	GO:0044062	1	0.99599557	0	2
9713	GO:0044065	1	0.993984383	0	3
9714	GO:0044070	1	0.989979161	0	5
9715	GO:0044088	1	0.99800092	0	1
9716	GO:0044090	1	0.995966904	0	2

9717	GO:0044091	1	0.996000027	0	2
9718	GO:0044093	1	0.99800067	0	1
9719	GO:0044105	1	0.997956897	0	1
9720	GO:0044111	1	0.998013437	0	1
9721	GO:0044147	1	0.997972358	0	1
9722	GO:0044154	1	0.984133055	0	8
9723	GO:0044183	1	0.931716108	0	35
9724	GO:0044194	1	0.991933659	0	4
9725	GO:0044195	1	0.997984457	0	1
9726	GO:0044205	1	0.993994038	0	3
9727	GO:0044206	1	0.989922325	0	5
9728	GO:0044207	1	0.998009026	0	1
9729	GO:0044208	1	0.993962535	0	3
9730	GO:0044209	1	0.995938564	0	2
9731	GO:0044210	1	0.996001528	0	2
9732	GO:0044211	1	0.993933754	0	3
9733	GO:0044214	1	0.976293529	0	12
9734	GO:0044216	1	0.998013388	0	1
9735	GO:0044218	1	0.997989689	0	1
9736	GO:0044224	1	0.980206797	0	10
9737	GO:0044225	1	0.9979829	0	1
9738	GO:0044228	1	0.99801218	0	1
9739	GO:0044232	1	0.982101029	0	9
9740	GO:0044233	1	0.962495595	0	19
9741	GO:0044237	1	0.97616266	0	12
9742	GO:0044241	1	0.993973878	0	3
9743	GO:0044242	1	0.989949031	0	5
9744	GO:0044245	1	0.99398529	0	3
9745	GO:0044249	1	0.993976249	0	3
9746	GO:0044255	1	0.960702706	0	20
9747	GO:0044257	1	0.974252312	0	13
9748	GO:0044258	1	0.997984899	0	1
9749	GO:0044260	1	0.995972537	0	2
9750	GO:0044262	1	0.993972694	0	3
9751	GO:0044265	1	0.993992097	0	3
9752	GO:0044267	1	0.771012722	0	129
9753	GO:0044275	1	0.99797628	0	1
9754	GO:0044284	1	0.997990564	0	1
9755	GO:0044291	1	0.954923588	0	23
9756	GO:0044292	1	0.989971211	0	5
9757	GO:0044294	1	0.982185291	0	9
9758	GO:0044295	1	0.939886736	0	31
9759	GO:0044297	1	0.847672379	0	82
9760	GO:0044298	1	0.996016688	0	2
9761	GO:0044299	1	0.998013437	0	1

9762	GO:0044300	1	0.994042666	0	3
9763	GO:0044301	1	0.995984836	0	2
9764	GO:0044302	1	0.997995231	0	1
9765	GO:0044304	1	0.988059451	0	6
9766	GO:0044305	1	0.964518818	0	18
9767	GO:0044306	1	0.960624177	0	20
9768	GO:0044307	1	0.99405187	0	3
9769	GO:0044308	1	0.994050447	0	3
9770	GO:0044309	1	0.988096682	0	6
9771	GO:0044313	1	0.994043354	0	3
9772	GO:0044314	1	0.989921347	0	5
9773	GO:0044316	1	0.995996185	0	2
9774	GO:0044319	1	0.97023347	0	15
9775	GO:0044320	1	0.982124608	0	9
9776	GO:0044321	1	0.982140423	0	9
9777	GO:0044322	1	0.939513276	0	31
9778	GO:0044323	1	0.992025697	0	4
9779	GO:0044325	1	0.772953738	0	128
9780	GO:0044326	1	0.986050927	0	7
9782	GO:0044328	1	0.997990564	0	1
9783	GO:0044329	1	0.997990564	0	1
9784	GO:0044330	1	0.997990564	0	1
9785	GO:0044331	1	0.984157714	0	8
9786	GO:0044334	1	0.996011971	0	2
9787	GO:0044335	1	0.998013437	0	1
9788	GO:0044336	1	0.996016173	0	2
9789	GO:0044338	1	0.993992546	0	3
9790	GO:0044339	1	0.995984029	0	2
9791	GO:0044340	1	0.996012103	0	2
9792	GO:0044341	1	0.993984311	0	3
9793	GO:0044342	1	0.982001537	0	9
9794	GO:0044344	1	0.935843235	0	33
9795	GO:0044345	1	0.998010994	0	1
9796	GO:0044346	1	0.993991454	0	3
9797	GO:0044351	1	0.986031927	0	7
9798	GO:0044354	1	0.984102595	0	8
9799	GO:0044355	1	0.997968826	0	1
9800	GO:0044357	1	0.997975698	0	1
9801	GO:0044375	1	0.993908821	0	3
9802	GO:0044377	1	0.998004657	0	1
9803	GO:0044378	1	0.995986632	0	2
9804	GO:0044380	1	0.998013424	0	1
9805	GO:0044381	1	0.992014005	0	4
9806	GO:0044387	1	0.986053857	0	7
9807	GO:0044388	1	0.9899076	0	5

9809	GO:0044390	1	0.985947368	0	7
9810	GO:0044393	1	0.992029794	0	4
9811	GO:0044406	1	0.993989972	0	3
9812	GO:0044409	1	0.993965868	0	3
9813	GO:0044458	1	0.95280613	0	24
9814	GO:0044466	1	0.995939606	0	2
9815	GO:0044524	1	0.997974251	0	1
9816	GO:0044528	1	0.986025009	0	7
9817	GO:0044530	1	0.994018212	0	3
9818	GO:0044539	1	0.984088608	0	8
9819	GO:0044540	1	0.997974251	0	1
9820	GO:0044545	1	0.986032246	0	7
9821	GO:0044546	1	0.997969585	0	1
9822	GO:0044547	1	0.99002105	0	5
9823	GO:0044548	1	0.974134928	0	13
9824	GO:0044549	1	0.997954463	0	1
9825	GO:0044557	1	0.998013437	0	1
9826	GO:0044565	1	0.995967206	0	2
9827	GO:0044571	1	0.987856746	0	6
9828	GO:0044580	1	0.997960497	0	1
9829	GO:0044594	1	0.971959892	0	14
9830	GO:0044595	1	0.997965466	0	1
9831	GO:0044596	1	0.997965466	0	1
9832	GO:0044597	1	0.98188786	0	9
9833	GO:0044598	1	0.98188786	0	9
9834	GO:0044599	1	0.997990564	0	1
9835	GO:0044602	1	0.997972563	0	1
9836	GO:0044603	1	0.997972563	0	1
9837	GO:0044609	1	0.995975367	0	2
9838	GO:0044610	1	0.997982916	0	1
9839	GO:0044611	1	0.9940485	0	3
9840	GO:0044613	1	0.985924333	0	7
9841	GO:0044614	1	0.994011416	0	3
9842	GO:0044615	1	0.976104559	0	12
9843	GO:0044648	1	0.988036742	0	6
9844	GO:0044650	1	0.997997404	0	1
9845	GO:0044666	1	0.974197644	0	13
9846	GO:0044691	1	0.98605715	0	7
9847	GO:0044715	1	0.9959386	0	2
9848	GO:0044716	1	0.9959386	0	2
9849	GO:0044717	1	0.99797021	0	1
9850	GO:0044721	1	0.997977399	0	1
9851	GO:0044726	1	0.997960952	0	1
9852	GO:0044727	1	0.996007903	0	2
9853	GO:0044729	1	0.996007792	0	2

9854	GO:0044730	1	0.997972358	0	1
9855	GO:0044736	1	0.995985075	0	2
9856	GO:0044752	1	0.991992104	0	4
9857	GO:0044753	1	0.996010213	0	2
9858	GO:0044754	1	0.978088189	0	11
9859	GO:0044770	1	0.988054472	0	6
9860	GO:0044772	1	0.956693329	0	22
9861	GO:0044773	1	0.993950925	0	3
9862	GO:0044774	1	0.997981138	0	1
9863	GO:0044778	1	0.995945719	0	2
9864	GO:0044779	1	0.997998262	0	1
9865	GO:0044782	1	0.950933138	0	25
9866	GO:0044783	1	0.998000642	0	1
9867	GO:0044788	1	0.997979166	0	1
9868	GO:0044790	1	0.998003949	0	1
9869	GO:0044791	1	0.990059143	0	5
9870	GO:0044793	1	0.997984615	0	1
9871	GO:0044794	1	0.984023983	0	8
9872	GO:0044795	1	0.997963344	0	1
9873	GO:0044803	1	0.994045699	0	3
9874	GO:0044804	1	0.986010264	0	7
9875	GO:0044805	1	0.990094806	0	5
9876	GO:0044806	1	0.986142785	0	7
9877	GO:0044818	1	0.988030843	0	6
9878	GO:0044819	1	0.995979059	0	2
9880	GO:0044828	1	0.986087365	0	7
9881	GO:0044829	1	0.984096068	0	8
9882	GO:0044830	1	0.992006562	0	4
9883	GO:0044839	1	0.995986713	0	2
9884	GO:0044843	1	0.995997918	0	2
9885	GO:0044849	1	0.968314318	0	16
9886	GO:0044853	1	0.947265566	0	27
9887	GO:0044854	1	0.997975836	0	1
9888	GO:0044855	1	0.998012965	0	1
9889	GO:0044857	1	0.993987735	0	3
9890	GO:0044860	1	0.995982004	0	2
9891	GO:0044861	1	0.997976578	0	1
9892	GO:0044873	1	0.998013437	0	1
9893	GO:0044877	1	0.457935459	0	385
9894	GO:0044878	1	0.991929592	0	4
9895	GO:0045002	1	0.997989425	0	1
9896	GO:0045003	1	0.994005687	0	3
9897	GO:0045004	1	0.996018569	0	2
9898	GO:0045006	1	0.991879028	0	4
9899	GO:0045007	1	0.952778916	0	24

9900	GO:0045008	1	0.983942644	0	8
9901	GO:0045010	1	0.976223359	0	12
9902	GO:0045016	1	0.997999553	0	1
9903	GO:0045019	1	0.968276472	0	16
9904	GO:0045022	1	0.956841185	0	22
9905	GO:0045023	1	0.995959667	0	2
9906	GO:0045025	1	0.996001945	0	2
9907	GO:0045026	1	0.994000018	0	3
9908	GO:0045027	1	0.993973975	0	3
9909	GO:0045028	1	0.984003033	0	8
9910	GO:0045029	1	0.997974496	0	1
9911	GO:0045030	1	0.995950822	0	2
9912	GO:0045031	1	0.993970863	0	3
9913	GO:0045039	1	0.975915262	0	12
9914	GO:0045040	1	0.991969549	0	4
9915	GO:0045041	1	0.993947915	0	3
9916	GO:0045046	1	0.956749448	0	22
9917	GO:0045047	1	0.974120604	0	13
9918	GO:0045048	1	0.954637433	0	23
9919	GO:0045050	1	0.980095136	0	10
9920	GO:0045053	1	0.990081779	0	5
9921	GO:0045054	1	0.989974165	0	5
9922	GO:0045055	1	0.958697666	0	21
9923	GO:0045056	1	0.978193617	0	11
9924	GO:0045058	1	0.993979489	0	3
9925	GO:0045059	1	0.978070645	0	11
9926	GO:0045060	1	0.978106568	0	11
9927	GO:0045061	1	0.990048976	0	5
9928	GO:0045062	1	0.997973628	0	1
9929	GO:0045063	1	0.986032846	0	7
9930	GO:0045064	1	0.99394267	0	3
9931	GO:0045065	1	0.997992152	0	1
9932	GO:0045066	1	0.995991333	0	2
9933	GO:0045069	1	0.994005866	0	3
9938	GO:0045089	1	0.948954905	0	26
9939	GO:0045091	1	0.997990564	0	1
9940	GO:0045092	1	0.998002011	0	1
9941	GO:0045095	1	0.924215836	0	39
9942	GO:0045098	1	0.993945918	0	3
9943	GO:0045103	1	0.993979685	0	3
9944	GO:0045104	1	0.954935194	0	23
9945	GO:0045105	1	0.99800283	0	1
9946	GO:0045109	1	0.970279979	0	15
9947	GO:0045110	1	0.98807528	0	6
9949	GO:0045112	1	0.998013437	0	1

9950	GO:0045113	1	0.997951655	0	1
9951	GO:0045116	1	0.976036021	0	12
9952	GO:0045117	1	0.995969534	0	2
9953	GO:0045120	1	0.989997245	0	5
9954	GO:0045121	1	0.629932922	0	229
9955	GO:0045123	1	0.989950534	0	5
9956	GO:0045124	1	0.982106638	0	9
9957	GO:0045125	1	0.995927962	0	2
9958	GO:0045127	1	0.997971268	0	1
9959	GO:0045130	1	0.997989999	0	1
9960	GO:0045131	1	0.997994113	0	1
9961	GO:0045132	1	0.990019873	0	5
9962	GO:0045134	1	0.982084832	0	9
9963	GO:0045136	1	0.995969732	0	2
9964	GO:0045137	1	0.997989278	0	1
9965	GO:0045141	1	0.986035681	0	7
9966	GO:0045142	1	0.996022641	0	2
9967	GO:0045143	1	0.985951614	0	7
9968	GO:0045145	1	0.991972782	0	4
9969	GO:0045148	1	0.997979825	0	1
9970	GO:0045159	1	0.980202388	0	10
9971	GO:0045161	1	0.995961228	0	2
9972	GO:0045162	1	0.990087401	0	5
9973	GO:0045163	1	0.994023871	0	3
9974	GO:0045165	1	0.893453667	0	56
9975	GO:0045167	1	0.997973698	0	1
9976	GO:0045171	1	0.863270729	0	73
9977	GO:0045174	1	0.995929731	0	2
9978	GO:0045175	1	0.997990564	0	1
9979	GO:0045176	1	0.970404841	0	15
9980	GO:0045177	1	0.843089842	0	85
9981	GO:0045178	1	0.976204131	0	12
9982	GO:0045179	1	0.984000372	0	8
9983	GO:0045180	1	0.990105214	0	5
9984	GO:0045182	1	0.956782222	0	22
9985	GO:0045183	1	0.997990564	0	1
9986	GO:0045184	1	0.921076746	0	41
9987	GO:0045185	1	0.996017108	0	2
9988	GO:0045186	1	0.998013437	0	1
9989	GO:0045187	1	0.989928437	0	5
9990	GO:0045188	1	0.997980594	0	1
9991	GO:0045190	1	0.97224965	0	14
9992	GO:0045191	1	0.995989579	0	2
9993	GO:0045197	1	0.966609475	0	17
9994	GO:0045198	1	0.972272584	0	14

9995	GO:0045199	1	0.980084727	0	10
9996	GO:0045200	1	0.994012749	0	3
9997	GO:0045202	1	0.239651666	0	701
9998	GO:0045204	1	0.996018677	0	2
9999	GO:0045209	1	0.998002594	0	1
10000	GO:0045210	1	0.998013437	0	1
10001	GO:0045211	1	0.716107347	0	166
10002	GO:0045213	1	0.99398177	0	3
10003	GO:0045214	1	0.937622321	0	32
10004	GO:0045216	1	0.949213939	0	26
10005	GO:0045217	1	0.988026139	0	6
10006	GO:0045218	1	0.994014715	0	3
10007	GO:0045221	1	0.998013384	0	1
10008	GO:0045225	1	0.997963383	0	1
10009	GO:0045226	1	0.995991955	0	2
10010	GO:0045234	1	0.997974974	0	1
10012	GO:0045237	1	0.997998423	0	1
10013	GO:0045239	1	0.997975078	0	1
10014	GO:0045244	1	0.997984362	0	1
10015	GO:0045252	1	0.990043344	0	5
10016	GO:0045254	1	0.990012139	0	5
10017	GO:0045259	1	0.995985075	0	2
10018	GO:0045261	1	0.988002428	0	6
10019	GO:0045263	1	0.972224266	0	14
10020	GO:0045271	1	0.997970759	0	1
10021	GO:0045273	1	0.997963149	0	1
10022	GO:0045275	1	0.997990564	0	1
10023	GO:0045277	1	0.981881203	0	9
10024	GO:0045281	1	0.99799003	0	1
10025	GO:0045292	1	0.97420841	0	13
10026	GO:0045294	1	0.978229193	0	11
10027	GO:0045295	1	0.976384805	0	12
10029	GO:0045298	1	0.99601819	0	2
10030	GO:0045309	1	0.993990754	0	3
10031	GO:0045321	1	0.989925921	0	5
10032	GO:0045322	1	0.984118225	0	8
10033	GO:0045324	1	0.984021757	0	8
10034	GO:0045329	1	0.989969609	0	5
10035	GO:0045332	1	0.953297227	0	24
10036	GO:0045333	1	0.970368854	0	15
10037	GO:0045334	1	0.960610803	0	20
10038	GO:0045335	1	0.861714281	0	74
10039	GO:0045337	1	0.995965062	0	2
10040	GO:0045338	1	0.997980382	0	1
10041	GO:0045340	1	0.993927411	0	3

10042	GO:0045342	1	0.998006135	0	1
10043	GO:0045343	1	0.997956978	0	1
10044	GO:0045344	1	0.997983315	0	1
10045	GO:0045345	1	0.990055135	0	5
10046	GO:0045347	1	0.991994974	0	4
10047	GO:0045348	1	0.982072006	0	9
10048	GO:0045352	1	0.997974904	0	1
10049	GO:0045353	1	0.997974904	0	1
10050	GO:0045428	1	0.986119586	0	7
10051	GO:0045429	1	0.92825124	0	37
10052	GO:0045444	1	0.882893066	0	62
10053	GO:0045445	1	0.97036206	0	15
10054	GO:0045446	1	0.976175206	0	12
10055	GO:0045453	1	0.954748998	0	23
10056	GO:0045454	1	0.918641839	0	42
10057	GO:0045471	1	0.810645404	0	104
10058	GO:0045472	1	0.998013437	0	1
10059	GO:0045475	1	0.97819063	0	11
10060	GO:0045494	1	0.921125999	0	41
10061	GO:0045495	1	0.997985246	0	1
10062	GO:0045499	1	0.949389215	0	26
10063	GO:0045503	1	0.980115212	0	10
10064	GO:0045504	1	0.974187962	0	13
10065	GO:0045505	1	0.94544286	0	28
10066	GO:0045509	1	0.995999656	0	2
10067	GO:0045513	1	0.997968714	0	1
10068	GO:0045515	1	0.997963149	0	1
10069	GO:0045519	1	0.997961071	0	1
10071	GO:0045541	1	0.989946461	0	5
10072	GO:0045542	1	0.988080555	0	6
10073	GO:0045545	1	0.988003101	0	6
10074	GO:0045547	1	0.996015155	0	2
10075	GO:0045550	1	0.995940417	0	2
10076	GO:0045569	1	0.989966597	0	5
10077	GO:0045576	1	0.991929984	0	4
10078	GO:0045577	1	0.984084053	0	8
10079	GO:0045578	1	0.991951879	0	4
10080	GO:0045579	1	0.976174695	0	12
10081	GO:0045580	1	0.974210971	0	13
10082	GO:0045581	1	0.989908043	0	5
10083	GO:0045582	1	0.970199916	0	15
10084	GO:0045584	1	0.997980063	0	1
10085	GO:0045585	1	0.995979974	0	2
10086	GO:0045586	1	0.994022101	0	3
10087	GO:0045588	1	0.986149383	0	7

10088	GO:0045589	1	0.974259474	0	13
10089	GO:0045590	1	0.991964493	0	4
10090	GO:0045591	1	0.976119143	0	12
10091	GO:0045595	1	0.917279308	0	43
10092	GO:0045596	1	0.94336386	0	29
10094	GO:0045598	1	0.9682767	0	16
10095	GO:0045599	1	0.915418549	0	44
10096	GO:0045600	1	0.917110542	0	43
10097	GO:0045601	1	0.9960172	0	2
10098	GO:0045602	1	0.988083489	0	6
10099	GO:0045603	1	0.978100543	0	11
10100	GO:0045604	1	0.991981906	0	4
10101	GO:0045605	1	0.995991715	0	2
10102	GO:0045606	1	0.987977312	0	6
10103	GO:0045607	1	0.992005036	0	4
10104	GO:0045608	1	0.991971394	0	4
10105	GO:0045616	1	0.968424226	0	16
10106	GO:0045617	1	0.992009965	0	4
10107	GO:0045618	1	0.966429297	0	17
10108	GO:0045619	1	0.995990292	0	2
10109	GO:0045620	1	0.997977911	0	1
10110	GO:0045621	1	0.990065514	0	5
10111	GO:0045622	1	0.993953427	0	3
10112	GO:0045623	1	0.998013437	0	1
10113	GO:0045624	1	0.997999172	0	1
10114	GO:0045626	1	0.99401154	0	3
10115	GO:0045627	1	0.991944882	0	4
10116	GO:0045629	1	0.993993051	0	3
10117	GO:0045630	1	0.9860119	0	7
10118	GO:0045634	1	0.997971666	0	1
10119	GO:0045636	1	0.994044362	0	3
10120	GO:0045637	1	0.978186317	0	11
10121	GO:0045638	1	0.970325469	0	15
10122	GO:0045639	1	0.991994552	0	4
10123	GO:0045645	1	0.998003371	0	1
10124	GO:0045646	1	0.989982226	0	5
10125	GO:0045647	1	0.986051403	0	7
10127	GO:0045650	1	0.989984787	0	5
10128	GO:0045651	1	0.974210375	0	13
10129	GO:0045652	1	0.875679743	0	66
10130	GO:0045653	1	0.966341908	0	17
10131	GO:0045654	1	0.982028253	0	9
10133	GO:0045656	1	0.990051275	0	5
10134	GO:0045657	1	0.980050014	0	10
10135	GO:0045659	1	0.996019783	0	2

10136	GO:0045660	1	0.99797847	0	1
10137	GO:0045661	1	0.989990624	0	5
10139	GO:0045663	1	0.964565184	0	18
10140	GO:0045664	1	0.947209732	0	27
10141	GO:0045665	1	0.9063864	0	49
10142	GO:0045666	1	0.868738299	0	70
10143	GO:0045667	1	0.97036283	0	15
10144	GO:0045668	1	0.928544312	0	37
10145	GO:0045669	1	0.886419419	0	60
10146	GO:0045670	1	0.974193992	0	13
10147	GO:0045671	1	0.958746672	0	21
10148	GO:0045672	1	0.972201805	0	14
10149	GO:0045682	1	0.991978432	0	4
10150	GO:0045684	1	0.989980299	0	5
10151	GO:0045685	1	0.998013437	0	1
10152	GO:0045686	1	0.99595709	0	2
10153	GO:0045687	1	0.991983092	0	4
10154	GO:0045703	1	0.997964698	0	1
10155	GO:0045717	1	0.978099311	0	11
10156	GO:0045719	1	0.986101999	0	7
10157	GO:0045720	1	0.995974415	0	2
10158	GO:0045721	1	0.97035522	0	15
10159	GO:0045722	1	0.974281969	0	13
10160	GO:0045723	1	0.980003352	0	10
10161	GO:0045724	1	0.970372311	0	15
10162	GO:0045725	1	0.972431303	0	14
10163	GO:0045726	1	0.994005991	0	3
10164	GO:0045727	1	0.858052846	0	76
10165	GO:0045728	1	0.993974207	0	3
10166	GO:0045730	1	0.976018283	0	12
10167	GO:0045732	1	0.863526189	0	73
10168	GO:0045736	1	0.943325333	0	29
10169	GO:0045737	1	0.943284011	0	29
10170	GO:0045738	1	0.997957079	0	1
10171	GO:0045739	1	0.937627151	0	32
10172	GO:0045740	1	0.943307736	0	29
10173	GO:0045741	1	0.974177008	0	13
10174	GO:0045742	1	0.954853942	0	23
10175	GO:0045743	1	0.984066473	0	8
10176	GO:0045744	1	0.974168564	0	13
10177	GO:0045745	1	0.976070217	0	12
10178	GO:0045746	1	0.933874238	0	34
10179	GO:0045747	1	0.908157697	0	48
10180	GO:0045751	1	0.997997982	0	1
10181	GO:0045759	1	0.991994139	0	4

10182	GO:0045760	1	0.994018273	0	3
10183	GO:0045761	1	0.993977082	0	3
10184	GO:0045762	1	0.982128211	0	9
10185	GO:0045763	1	0.997980773	0	1
10186	GO:0045764	1	0.998004561	0	1
10187	GO:0045765	1	0.939676101	0	31
10188	GO:0045766	1	0.772680965	0	128
10189	GO:0045769	1	0.998013437	0	1
10190	GO:0045773	1	0.936032118	0	33
10191	GO:0045776	1	0.954825661	0	23
10192	GO:0045777	1	0.968297479	0	16
10193	GO:0045778	1	0.976253998	0	12
10194	GO:0045779	1	0.97216697	0	14
10195	GO:0045780	1	0.966450445	0	17
10196	GO:0045785	1	0.90968035	0	47
10197	GO:0045786	1	0.909691037	0	47
10198	GO:0045787	1	0.924596021	0	39
10199	GO:0045792	1	0.982083854	0	9
10200	GO:0045793	1	0.978150447	0	11
10201	GO:0045794	1	0.990082203	0	5
10202	GO:0045795	1	0.998013437	0	1
10203	GO:0045796	1	0.997989301	0	1
10204	GO:0045797	1	0.995994485	0	2
10205	GO:0045799	1	0.998012887	0	1
10206	GO:0045806	1	0.970377042	0	15
10207	GO:0045807	1	0.954829347	0	23
10208	GO:0045813	1	0.997975268	0	1
10209	GO:0045814	1	0.898942885	0	53
10210	GO:0045815	1	0.935704139	0	33
10211	GO:0045819	1	0.996011444	0	2
10212	GO:0045820	1	0.976205652	0	12
10213	GO:0045821	1	0.95884956	0	21
10214	GO:0045822	1	0.988108419	0	6
10215	GO:0045823	1	0.984087711	0	8
10217	GO:0045829	1	0.997984449	0	1
10218	GO:0045830	1	0.974192925	0	13
10219	GO:0045833	1	0.991888309	0	4
10220	GO:0045834	1	0.997990564	0	1
10221	GO:0045835	1	0.98804393	0	6
10222	GO:0045836	1	0.989953429	0	5
10223	GO:0045837	1	0.995945214	0	2
10224	GO:0045838	1	0.989950553	0	5
10225	GO:0045839	1	0.983998153	0	8
10226	GO:0045840	1	0.951027516	0	25
10227	GO:0045841	1	0.995990307	0	2

10228	GO:0045842	1	0.976172954	0	12
10229	GO:0045843	1	0.991946086	0	4
10230	GO:0045844	1	0.997990564	0	1
10231	GO:0045851	1	0.991939472	0	4
10232	GO:0045852	1	0.998012817	0	1
10233	GO:0045859	1	0.948970771	0	26
10234	GO:0045860	1	0.893534871	0	56
10235	GO:0045861	1	0.956558702	0	22
10236	GO:0045862	1	0.960588819	0	20
10237	GO:0045869	1	0.968273927	0	16
10238	GO:0045870	1	0.99603051	0	2
10239	GO:0045875	1	0.996007903	0	2
10240	GO:0045876	1	0.992013559	0	4
10241	GO:0045879	1	0.947384822	0	27
10242	GO:0045880	1	0.932218895	0	35
10243	GO:0045887	1	0.998013437	0	1
10244	GO:0045892	1	0.342075804	0	528
10246	GO:0045898	1	0.998011936	0	1
10247	GO:0045899	1	0.980036896	0	10
10248	GO:0045900	1	0.997970841	0	1
10249	GO:0045901	1	0.992003202	0	4
10250	GO:0045903	1	0.991949187	0	4
10251	GO:0045905	1	0.991996844	0	4
10252	GO:0045906	1	0.992004232	0	4
10253	GO:0045907	1	0.949062266	0	26
10254	GO:0045910	1	0.970369265	0	15
10255	GO:0045911	1	0.997984252	0	1
10256	GO:0045916	1	0.992014571	0	4
10257	GO:0045917	1	0.995949576	0	2
10258	GO:0045918	1	0.998013437	0	1
10259	GO:0045920	1	0.989948234	0	5
10260	GO:0045921	1	0.954890303	0	23
10261	GO:0045922	1	0.987998961	0	6
10262	GO:0045923	1	0.992026484	0	4
10263	GO:0045924	1	0.997984599	0	1
10264	GO:0045925	1	0.996002728	0	2
10265	GO:0045926	1	0.9680637	0	16
10266	GO:0045927	1	0.988012486	0	6
10267	GO:0045930	1	0.945305806	0	28
10268	GO:0045931	1	0.939765257	0	31
10269	GO:0045932	1	0.997960438	0	1
10270	GO:0045933	1	0.997975544	0	1
10271	GO:0045938	1	0.997987608	0	1
10272	GO:0045939	1	0.995973341	0	2
10273	GO:0045940	1	0.997968397	0	1

10274	GO:0045943	1	0.974295322	0	13
10276	GO:0045945	1	0.978195306	0	11
10277	GO:0045947	1	0.972236296	0	14
10278	GO:0045948	1	0.970289142	0	15
10279	GO:0045950	1	0.993978304	0	3
10280	GO:0045951	1	0.997981276	0	1
10281	GO:0045953	1	0.98194413	0	9
10282	GO:0045954	1	0.964413907	0	18
10283	GO:0045955	1	0.984083817	0	8
10284	GO:0045956	1	0.972296861	0	14
10285	GO:0045957	1	0.995997004	0	2
10286	GO:0045959	1	0.991958746	0	4
10287	GO:0045963	1	0.99200926	0	4
10288	GO:0045964	1	0.991986568	0	4
10289	GO:0045967	1	0.994040553	0	3
10290	GO:0045975	1	0.997990564	0	1
10291	GO:0045976	1	0.996011255	0	2
10292	GO:0045977	1	0.997968974	0	1
10293	GO:0045980	1	0.997959405	0	1
10294	GO:0045986	1	0.992032335	0	4
10295	GO:0045987	1	0.968308853	0	16
10296	GO:0045988	1	0.994017777	0	3
10297	GO:0045989	1	0.996005628	0	2
10298	GO:0045990	1	0.997974982	0	1
10299	GO:0045992	1	0.998013437	0	1
10300	GO:0045993	1	0.998011029	0	1
10301	GO:0045994	1	0.997972866	0	1
10302	GO:0045995	1	0.960718693	0	20
10303	GO:0046006	1	0.997977348	0	1
10304	GO:0046007	1	0.974202057	0	13
10305	GO:0046010	1	0.997954504	0	1
10306	GO:0046013	1	0.995967268	0	2
10307	GO:0046015	1	0.997973009	0	1
10308	GO:0046016	1	0.998010126	0	1
10309	GO:0046030	1	0.997997701	0	1
10310	GO:0046031	1	0.997962143	0	1
10311	GO:0046032	1	0.995982556	0	2
10312	GO:0046033	1	0.986028324	0	7
10313	GO:0046034	1	0.935815214	0	33
10314	GO:0046036	1	0.997993327	0	1
10315	GO:0046037	1	0.980156085	0	10
10316	GO:0046038	1	0.994006876	0	3
10317	GO:0046039	1	0.974282264	0	13
10318	GO:0046040	1	0.991986407	0	4
10319	GO:0046041	1	0.997988836	0	1

10320	GO:0046050	1	0.989931123	0	5
10321	GO:0046051	1	0.997988836	0	1
10322	GO:0046052	1	0.998013437	0	1
10323	GO:0046054	1	0.997960081	0	1
10324	GO:0046055	1	0.993997675	0	3
10325	GO:0046057	1	0.99797021	0	1
10326	GO:0046058	1	0.995982613	0	2
10327	GO:0046059	1	0.995959831	0	2
10328	GO:0046060	1	0.997960081	0	1
10329	GO:0046061	1	0.995971934	0	2
10330	GO:0046067	1	0.99797021	0	1
10331	GO:0046068	1	0.995992868	0	2
10332	GO:0046069	1	0.990032932	0	5
10333	GO:0046070	1	0.997960497	0	1
10334	GO:0046074	1	0.993919354	0	3
10335	GO:0046079	1	0.987922518	0	6
10336	GO:0046081	1	0.997976468	0	1
10337	GO:0046083	1	0.995927396	0	2
10338	GO:0046084	1	0.998000955	0	1
10339	GO:0046085	1	0.989995266	0	5
10340	GO:0046086	1	0.998008111	0	1
10341	GO:0046092	1	0.998012983	0	1
10342	GO:0046098	1	0.996012958	0	2
10343	GO:0046100	1	0.997968322	0	1
10344	GO:0046101	1	0.995955741	0	2
10345	GO:0046103	1	0.99396076	0	3
10346	GO:0046104	1	0.995988551	0	2
10347	GO:0046105	1	0.995957538	0	2
10348	GO:0046108	1	0.995964646	0	2
10349	GO:0046111	1	0.997970558	0	1
10350	GO:0046121	1	0.997973945	0	1
10351	GO:0046122	1	0.997960497	0	1
10352	GO:0046130	1	0.998011966	0	1
10353	GO:0046134	1	0.993994038	0	3
10354	GO:0046135	1	0.979997581	0	10
10355	GO:0046146	1	0.981993553	0	9
10356	GO:0046164	1	0.997978287	0	1
10357	GO:0046166	1	0.995953545	0	2
10358	GO:0046167	1	0.996020987	0	2
10359	GO:0046168	1	0.996003077	0	2
10360	GO:0046177	1	0.995972204	0	2
10361	GO:0046185	1	0.99596494	0	2
10362	GO:0046203	1	0.997973416	0	1
10363	GO:0046204	1	0.997959564	0	1
10364	GO:0046208	1	0.995957234	0	2

10365	GO:0046209	1	0.994038766	0	3
10366	GO:0046210	1	0.997986766	0	1
10367	GO:0046219	1	0.989915148	0	5
10368	GO:0046222	1	0.97394987	0	13
10369	GO:0046223	1	0.995959362	0	2
10370	GO:0046226	1	0.995942735	0	2
10371	GO:0046272	1	0.997982002	0	1
10372	GO:0046293	1	0.998012308	0	1
10373	GO:0046294	1	0.993943279	0	3
10374	GO:0046295	1	0.997958946	0	1
10375	GO:0046314	1	0.989899152	0	5
10376	GO:0046316	1	0.997990564	0	1
10377	GO:0046318	1	0.998013077	0	1
10378	GO:0046320	1	0.989991341	0	5
10379	GO:0046321	1	0.988058531	0	6
10380	GO:0046322	1	0.986019914	0	7
10381	GO:0046323	1	0.988044854	0	6
10382	GO:0046324	1	0.986045934	0	7
10383	GO:0046325	1	0.976165657	0	12
10384	GO:0046326	1	0.936106258	0	33
10385	GO:0046327	1	0.997978061	0	1
10386	GO:0046328	1	0.964650729	0	18
10387	GO:0046329	1	0.947338124	0	27
10388	GO:0046330	1	0.84619044	0	83
10389	GO:0046331	1	0.993947345	0	3
10390	GO:0046332	1	0.902828303	0	51
10391	GO:0046337	1	0.991919701	0	4
10392	GO:0046338	1	0.994000733	0	3
10393	GO:0046339	1	0.974345161	0	13
10394	GO:0046340	1	0.996022876	0	2
10395	GO:0046341	1	0.997976485	0	1
10396	GO:0046356	1	0.995945408	0	2
10397	GO:0046359	1	0.997977281	0	1
10398	GO:0046360	1	0.997980496	0	1
10399	GO:0046368	1	0.99799873	0	1
10400	GO:0046370	1	0.995959136	0	2
10401	GO:0046373	1	0.995985075	0	2
10402	GO:0046380	1	0.998005384	0	1
10403	GO:0046386	1	0.995976008	0	2
10404	GO:0046390	1	0.99596851	0	2
10405	GO:0046395	1	0.995942509	0	2
10406	GO:0046398	1	0.99800712	0	1
10407	GO:0046403	1	0.997973945	0	1
10408	GO:0046404	1	0.993973547	0	3
10409	GO:0046415	1	0.990011367	0	5

10410	GO:0046416	1	0.997972026	0	1
10411	GO:0046425	1	0.980121943	0	10
10412	GO:0046426	1	0.97225632	0	14
10413	GO:0046427	1	0.956660651	0	22
10414	GO:0046434	1	0.997974417	0	1
10415	GO:0046449	1	0.998013433	0	1
10416	GO:0046452	1	0.993989333	0	3
10417	GO:0046456	1	0.987977618	0	6
10418	GO:0046458	1	0.998005077	0	1
10419	GO:0046459	1	0.995967808	0	2
10420	GO:0046464	1	0.989995493	0	5
10421	GO:0046466	1	0.991949531	0	4
10422	GO:0046469	1	0.993997121	0	3
10423	GO:0046470	1	0.970164986	0	15
10424	GO:0046471	1	0.989979466	0	5
10425	GO:0046473	1	0.987985758	0	6
10426	GO:0046474	1	0.97815045	0	11
10427	GO:0046475	1	0.970294852	0	15
10428	GO:0046477	1	0.995976961	0	2
10429	GO:0046479	1	0.997968378	0	1
10430	GO:0046483	1	0.985957055	0	7
10431	GO:0046485	1	0.990010151	0	5
10432	GO:0046486	1	0.97632584	0	12
10433	GO:0046487	1	0.987933436	0	6
10434	GO:0046488	1	0.945411062	0	28
10435	GO:0046491	1	0.997957643	0	1
10436	GO:0046496	1	0.995985075	0	2
10437	GO:0046498	1	0.995925827	0	2
10438	GO:0046500	1	0.989951766	0	5
10439	GO:0046501	1	0.994010459	0	3
10440	GO:0046511	1	0.998013437	0	1
10441	GO:0046512	1	0.972204843	0	14
10442	GO:0046513	1	0.939493636	0	31
10443	GO:0046514	1	0.985958027	0	7
10444	GO:0046520	1	0.998006036	0	1
10445	GO:0046521	1	0.997976331	0	1
10446	GO:0046523	1	0.997995999	0	1
10447	GO:0046525	1	0.997973927	0	1
10448	GO:0046526	1	0.997987078	0	1
10449	GO:0046527	1	0.988019009	0	6
10450	GO:0046533	1	0.990014741	0	5
10451	GO:0046538	1	0.997957361	0	1
10452	GO:0046539	1	0.997958366	0	1
10453	GO:0046540	1	0.939108573	0	31
10454	GO:0046541	1	0.989931845	0	5

10455	GO:0046543	1	0.996007538	0	2
10456	GO:0046544	1	0.998013071	0	1
10457	GO:0046545	1	0.992009244	0	4
10458	GO:0046546	1	0.992039134	0	4
10459	GO:0046548	1	0.9821551	0	9
10460	GO:0046549	1	0.980191099	0	10
10461	GO:0046554	1	0.997966249	0	1
10462	GO:0046556	1	0.995985075	0	2
10463	GO:0046570	1	0.997965352	0	1
10464	GO:0046577	1	0.998005077	0	1
10465	GO:0046578	1	0.982142566	0	9
10466	GO:0046579	1	0.943535317	0	29
10467	GO:0046580	1	0.95117786	0	25
10468	GO:0046581	1	0.986126227	0	7
10469	GO:0046587	1	0.997994528	0	1
10470	GO:0046588	1	0.997958406	0	1
10471	GO:0046592	1	0.993944738	0	3
10472	GO:0046596	1	0.974216335	0	13
10473	GO:0046597	1	0.966252795	0	17
10474	GO:0046598	1	0.976036902	0	12
10475	GO:0046599	1	0.982102913	0	9
10476	GO:0046600	1	0.986127289	0	7
10477	GO:0046601	1	0.996008757	0	2
10478	GO:0046602	1	0.99002646	0	5
10479	GO:0046604	1	0.993985204	0	3
10480	GO:0046605	1	0.992004921	0	4
10481	GO:0046611	1	0.997990564	0	1
10482	GO:0046618	1	0.994050411	0	3
10483	GO:0046619	1	0.996012759	0	2
10484	GO:0046620	1	0.98208266	0	9
10485	GO:0046621	1	0.98419165	0	8
10486	GO:0046622	1	0.982085399	0	9
10487	GO:0046623	1	0.998013437	0	1
10488	GO:0046624	1	0.995984435	0	2
10489	GO:0046625	1	0.991967336	0	4
10490	GO:0046626	1	0.97623629	0	12
10491	GO:0046627	1	0.93210158	0	35
10492	GO:0046628	1	0.958805141	0	21
10493	GO:0046629	1	0.989980483	0	5
10494	GO:0046631	1	0.998013437	0	1
10495	GO:0046632	1	0.986020806	0	7
10496	GO:0046633	1	0.998013437	0	1
10497	GO:0046635	1	0.99796466	0	1
10498	GO:0046636	1	0.995983832	0	2
10499	GO:0046638	1	0.980050051	0	10

10500	GO:0046639	1	0.993976253	0	3
10501	GO:0046641	1	0.984041622	0	8
10502	GO:0046642	1	0.994006227	0	3
10503	GO:0046645	1	0.998011136	0	1
10504	GO:0046649	1	0.993989059	0	3
10505	GO:0046651	1	0.989971826	0	5
10506	GO:0046653	1	0.982018399	0	9
10507	GO:0046654	1	0.989995666	0	5
10508	GO:0046655	1	0.964461654	0	18
10509	GO:0046657	1	0.997957924	0	1
10510	GO:0046658	1	0.943230883	0	29
10511	GO:0046661	1	0.993989008	0	3
10512	GO:0046666	1	0.997956473	0	1
10513	GO:0046668	1	0.995994088	0	2
10514	GO:0046671	1	0.998005976	0	1
10515	GO:0046676	1	0.945345851	0	28
10516	GO:0046677	1	0.939488054	0	31
10517	GO:0046680	1	0.997980104	0	1
10518	GO:0046683	1	0.989957876	0	5
10519	GO:0046684	1	0.993969258	0	3
10520	GO:0046685	1	0.976185433	0	12
10521	GO:0046686	1	0.948883305	0	26
10522	GO:0046688	1	0.968273977	0	16
10523	GO:0046689	1	0.98795095	0	6
10524	GO:0046690	1	0.998001484	0	1
10525	GO:0046691	1	0.996029264	0	2
10526	GO:0046695	1	0.993944427	0	3
10527	GO:0046696	1	0.989973143	0	5
10528	GO:0046697	1	0.950864789	0	25
10529	GO:0046703	1	0.981922339	0	9
10530	GO:0046705	1	0.997992152	0	1
10531	GO:0046709	1	0.995987871	0	2
10532	GO:0046710	1	0.982144148	0	9
10533	GO:0046711	1	0.997960081	0	1
10534	GO:0046712	1	0.995967518	0	2
10535	GO:0046713	1	0.997996143	0	1
10536	GO:0046715	1	0.997996143	0	1
10537	GO:0046716	1	0.964485114	0	18
10538	GO:0046717	1	0.995971196	0	2
10539	GO:0046718	1	0.842853888	0	85
10540	GO:0046722	1	0.997984457	0	1
10541	GO:0046724	1	0.997989046	0	1
10543	GO:0046726	1	0.992037265	0	4
10544	GO:0046745	1	0.997990564	0	1
10545	GO:0046754	1	0.997994144	0	1

10546	GO:0046755	1	0.982057253	0	9
10547	GO:0046777	1	0.710640933	0	170
10548	GO:0046778	1	0.995974553	0	2
10549	GO:0046782	1	0.997968918	0	1
10550	GO:0046784	1	0.985959185	0	7
10551	GO:0046785	1	0.976207181	0	12
10552	GO:0046786	1	0.998012691	0	1
10553	GO:0046789	1	0.997997404	0	1
10554	GO:0046790	1	0.984001522	0	8
10555	GO:0046811	1	0.995969847	0	2
10556	GO:0046813	1	0.990009369	0	5
10557	GO:0046814	1	0.997990564	0	1
10558	GO:0046817	1	0.997964679	0	1
10560	GO:0046824	1	0.998013437	0	1
10562	GO:0046826	1	0.987926851	0	6
10563	GO:0046827	1	0.962505379	0	19
10564	GO:0046831	1	0.997989549	0	1
10565	GO:0046832	1	0.996030729	0	2
10566	GO:0046833	1	0.99002452	0	5
10567	GO:0046834	1	0.972403522	0	14
10568	GO:0046835	1	0.95481183	0	23
10569	GO:0046836	1	0.991910676	0	4
10570	GO:0046838	1	0.997987826	0	1
10571	GO:0046839	1	0.974157027	0	13
10572	GO:0046847	1	0.964625939	0	18
10573	GO:0046848	1	0.993882564	0	3
10574	GO:0046849	1	0.968408767	0	16
10575	GO:0046850	1	0.990009758	0	5
10576	GO:0046851	1	0.996023599	0	2
10577	GO:0046854	1	0.90098591	0	52
10578	GO:0046855	1	0.966525742	0	17
10579	GO:0046856	1	0.939860845	0	31
10580	GO:0046865	1	0.998012652	0	1
10581	GO:0046870	1	0.991984004	0	4
10583	GO:0046873	1	0.966418937	0	17
10584	GO:0046874	1	0.995940661	0	2
10585	GO:0046875	1	0.945313986	0	28
10586	GO:0046877	1	0.998012797	0	1
10587	GO:0046878	1	0.997990556	0	1
10588	GO:0046879	1	0.991976529	0	4
10589	GO:0046880	1	0.997981405	0	1
10590	GO:0046881	1	0.990000601	0	5
10591	GO:0046882	1	0.993959115	0	3
10592	GO:0046883	1	0.993943172	0	3
10593	GO:0046884	1	0.998012243	0	1

10594	GO:0046885	1	0.998013324	0	1
10595	GO:0046886	1	0.994028036	0	3
10596	GO:0046887	1	0.987979581	0	6
10597	GO:0046888	1	0.984011715	0	8
10598	GO:0046889	1	0.972252007	0	14
10599	GO:0046890	1	0.987986659	0	6
10600	GO:0046898	1	0.996000336	0	2
10601	GO:0046899	1	0.99598335	0	2
10602	GO:0046900	1	0.997969714	0	1
10603	GO:0046901	1	0.997986485	0	1
10604	GO:0046902	1	0.976118586	0	12
10605	GO:0046903	1	0.988023782	0	6
10606	GO:0046906	1	0.997994159	0	1
10607	GO:0046907	1	0.937852955	0	32
10608	GO:0046911	1	0.997963771	0	1
10609	GO:0046912	1	0.996008383	0	2
10610	GO:0046914	1	0.964314173	0	18
10611	GO:0046915	1	0.996019087	0	2
10612	GO:0046920	1	0.988044771	0	6
10613	GO:0046921	1	0.99799873	0	1
10614	GO:0046922	1	0.995999282	0	2
10615	GO:0046923	1	0.98994319	0	5
10616	GO:0046928	1	0.966490149	0	17
10617	GO:0046929	1	0.992000443	0	4
10618	GO:0046930	1	0.974095025	0	13
10619	GO:0046931	1	0.978089909	0	11
10620	GO:0046933	1	0.968310965	0	16
10621	GO:0046934	1	0.988075956	0	6
10622	GO:0046935	1	0.974271964	0	13
10623	GO:0046939	1	0.995938065	0	2
10625	GO:0046943	1	0.994046493	0	3
10626	GO:0046947	1	0.996006082	0	2
10628	GO:0046950	1	0.99598212	0	2
10629	GO:0046951	1	0.984018714	0	8
10630	GO:0046952	1	0.991982955	0	4
10631	GO:0046958	1	0.997998423	0	1
10632	GO:0046959	1	0.994025339	0	3
10633	GO:0046960	1	0.997984599	0	1
10634	GO:0046961	1	0.954581517	0	23
10635	GO:0046963	1	0.997979233	0	1
10636	GO:0046964	1	0.995962965	0	2
10637	GO:0046965	1	0.966486552	0	17
10639	GO:0046967	1	0.996005446	0	2
10640	GO:0046968	1	0.994004808	0	3
10641	GO:0046969	1	0.99598226	0	2

10642	GO:0046970	1	0.997977718	0	1
10643	GO:0046972	1	0.995978355	0	2
10644	GO:0046974	1	0.980249973	0	10
10646	GO:0046976	1	0.990034008	0	5
10647	GO:0046977	1	0.993924211	0	3
10648	GO:0046978	1	0.990010524	0	5
10649	GO:0046979	1	0.993973155	0	3
10650	GO:0046980	1	0.998007827	0	1
10651	GO:0046982	1	0.534822824	0	309
10652	GO:0046983	1	0.749453683	0	143
10653	GO:0046984	1	0.998011029	0	1
10654	GO:0046985	1	0.991965209	0	4
10655	GO:0046986	1	0.998011029	0	1
10656	GO:0047006	1	0.995940322	0	2
10657	GO:0047012	1	0.997971684	0	1
10658	GO:0047015	1	0.997959295	0	1
10659	GO:0047016	1	0.997981664	0	1
10660	GO:0047017	1	0.99595926	0	2
10661	GO:0047020	1	0.995933772	0	2
10662	GO:0047021	1	0.997965025	0	1
10663	GO:0047023	1	0.983865146	0	8
10664	GO:0047024	1	0.989877132	0	5
10665	GO:0047025	1	0.99596517	0	2
10666	GO:0047035	1	0.98385753	0	8
10667	GO:0047042	1	0.997967667	0	1
10668	GO:0047044	1	0.981856626	0	9
10669	GO:0047045	1	0.983861551	0	8
10670	GO:0047057	1	0.995926229	0	2
10671	GO:0047066	1	0.995924184	0	2
10672	GO:0047086	1	0.989896058	0	5
10673	GO:0047092	1	0.997984994	0	1
10674	GO:0047102	1	0.997986023	0	1
10675	GO:0047105	1	0.997986719	0	1
10676	GO:0047115	1	0.993911452	0	3
10677	GO:0047117	1	0.998013437	0	1
10678	GO:0047127	1	0.997967507	0	1
10679	GO:0047130	1	0.998013437	0	1
10680	GO:0047131	1	0.998013437	0	1
10681	GO:0047134	1	0.991934005	0	4
10682	GO:0047150	1	0.995969138	0	2
10683	GO:0047159	1	0.996024327	0	2
10684	GO:0047166	1	0.997977196	0	1
10685	GO:0047173	1	0.998012626	0	1
10686	GO:0047179	1	0.991919836	0	4
10687	GO:0047184	1	0.984044593	0	8

10688	GO:0047186	1	0.998006447	0	1
10689	GO:0047191	1	0.9980071	0	1
10690	GO:0047192	1	0.994009383	0	3
10691	GO:0047220	1	0.997991067	0	1
10692	GO:0047223	1	0.993980471	0	3
10693	GO:0047224	1	0.997986516	0	1
10694	GO:0047225	1	0.997982179	0	1
10695	GO:0047237	1	0.994015738	0	3
10696	GO:0047238	1	0.988084328	0	6
10697	GO:0047256	1	0.998008633	0	1
10698	GO:0047263	1	0.997992871	0	1
10699	GO:0047273	1	0.997999107	0	1
10700	GO:0047275	1	0.997981308	0	1
10701	GO:0047277	1	0.997977752	0	1
10702	GO:0047280	1	0.998011582	0	1
10703	GO:0047288	1	0.994010381	0	3
10704	GO:0047290	1	0.995958432	0	2
10705	GO:0047291	1	0.997983633	0	1
10706	GO:0047293	1	0.99797242	0	1
10707	GO:0047298	1	0.998012706	0	1
10708	GO:0047315	1	0.997990564	0	1
10709	GO:0047316	1	0.997990564	0	1
10710	GO:0047322	1	0.996030372	0	2
10711	GO:0047323	1	0.997980773	0	1
10712	GO:0047325	1	0.997998232	0	1
10713	GO:0047326	1	0.997975112	0	1
10714	GO:0047341	1	0.998000656	0	1
10715	GO:0047349	1	0.997990564	0	1
10716	GO:0047369	1	0.997990564	0	1
10717	GO:0047372	1	0.978095265	0	11
10718	GO:0047374	1	0.993961658	0	3
10719	GO:0047376	1	0.996000056	0	2
10720	GO:0047381	1	0.997974487	0	1
10721	GO:0047389	1	0.996014461	0	2
10722	GO:0047390	1	0.998006948	0	1
10723	GO:0047391	1	0.995997963	0	2
10724	GO:0047394	1	0.997982492	0	1
10725	GO:0047395	1	0.997993389	0	1
10726	GO:0047402	1	0.997990564	0	1
10727	GO:0047408	1	0.995981018	0	2
10728	GO:0047409	1	0.995981018	0	2
10729	GO:0047412	1	0.997975242	0	1
10730	GO:0047419	1	0.997975827	0	1
10731	GO:0047429	1	0.985989171	0	7
10732	GO:0047442	1	0.997976434	0	1

10733	GO:0047444	1	0.997964968	0	1
10734	GO:0047451	1	0.996007903	0	2
10735	GO:0047453	1	0.997990564	0	1
10736	GO:0047464	1	0.998012966	0	1
10737	GO:0047484	1	0.994026381	0	3
10738	GO:0047485	1	0.807484223	0	106
10739	GO:0047493	1	0.994000085	0	3
10740	GO:0047496	1	0.966519808	0	17
10741	GO:0047497	1	0.986005335	0	7
10742	GO:0047498	1	0.974117676	0	13
10743	GO:0047499	1	0.986000426	0	7
10744	GO:0047522	1	0.993977933	0	3
10745	GO:0047536	1	0.997982267	0	1
10746	GO:0047545	1	0.998013437	0	1
10747	GO:0047555	1	0.978206823	0	11
10748	GO:0047560	1	0.998013197	0	1
10750	GO:0047603	1	0.997963383	0	1
10751	GO:0047609	1	0.997989557	0	1
10752	GO:0047611	1	0.997989557	0	1
10753	GO:0047617	1	0.977997465	0	11
10754	GO:0047620	1	0.997993389	0	1
10755	GO:0047621	1	0.997973115	0	1
10756	GO:0047631	1	0.989885651	0	5
10757	GO:0047655	1	0.991905484	0	4
10758	GO:0047676	1	0.982129602	0	9
10759	GO:0047677	1	0.997986813	0	1
10760	GO:0047685	1	0.997969981	0	1
10761	GO:0047690	1	0.994002995	0	3
10762	GO:0047693	1	0.997954504	0	1
10763	GO:0047696	1	0.991986203	0	4
10764	GO:0047704	1	0.997978403	0	1
10765	GO:0047708	1	0.995947262	0	2
10766	GO:0047710	1	0.995977932	0	2
10767	GO:0047718	1	0.993921179	0	3
10768	GO:0047726	1	0.997986766	0	1
10769	GO:0047730	1	0.99800715	0	1
10770	GO:0047734	1	0.997990564	0	1
10771	GO:0047743	1	0.997964119	0	1
10772	GO:0047747	1	0.995972268	0	2
10773	GO:0047748	1	0.99798341	0	1
10774	GO:0047749	1	0.99798341	0	1
10775	GO:0047750	1	0.995927188	0	2
10776	GO:0047751	1	0.993969634	0	3
10777	GO:0047756	1	0.993979048	0	3
10778	GO:0047757	1	0.996025067	0	2

10779	GO:0047760	1	0.985995259	0	7
10780	GO:0047777	1	0.998013437	0	1
10781	GO:0047780	1	0.995996995	0	2
10782	GO:0047787	1	0.995958371	0	2
10783	GO:0047800	1	0.9980045	0	1
10784	GO:0047801	1	0.99798074	0	1
10785	GO:0047804	1	0.995985075	0	2
10786	GO:0047805	1	0.997990564	0	1
10787	GO:0047820	1	0.997990564	0	1
10788	GO:0047834	1	0.96419022	0	18
10789	GO:0047837	1	0.997961917	0	1
10790	GO:0047840	1	0.997962564	0	1
10791	GO:0047844	1	0.979945743	0	10
10792	GO:0047847	1	0.995964646	0	2
10793	GO:0047860	1	0.997965581	0	1
10794	GO:0047865	1	0.997995795	0	1
10795	GO:0047874	1	0.997980463	0	1
10796	GO:0047886	1	0.997972223	0	1
10797	GO:0047888	1	0.997977768	0	1
10798	GO:0047894	1	0.993916251	0	3
10799	GO:0047915	1	0.997973151	0	1
10800	GO:0047918	1	0.997990564	0	1
10801	GO:0047931	1	0.998005792	0	1
10802	GO:0047933	1	0.998013437	0	1
10803	GO:0047934	1	0.998013437	0	1
10804	GO:0047935	1	0.998013437	0	1
10805	GO:0047936	1	0.998013437	0	1
10806	GO:0047939	1	0.997969391	0	1
10807	GO:0047941	1	0.997969391	0	1
10808	GO:0047946	1	0.997979759	0	1
10809	GO:0047956	1	0.995941484	0	2
10810	GO:0047961	1	0.995951345	0	2
10811	GO:0047963	1	0.998000663	0	1
10812	GO:0047975	1	0.995979798	0	2
10813	GO:0047977	1	0.995977999	0	2
10814	GO:0047988	1	0.99797696	0	1
10815	GO:0047992	1	0.997990564	0	1
10816	GO:0047994	1	0.997963383	0	1
10817	GO:0048002	1	0.991993433	0	4
10818	GO:0048006	1	0.998005257	0	1
10819	GO:0048007	1	0.994036973	0	3
10820	GO:0048008	1	0.941833888	0	30
10821	GO:0048009	1	0.972510184	0	14
10822	GO:0048010	1	0.875862867	0	66
10823	GO:0048011	1	0.962580608	0	19

10824	GO:0048012	1	0.978133556	0	11
10825	GO:0048013	1	0.844613018	0	84
10826	GO:0048014	1	0.992072416	0	4
10827	GO:0048015	1	0.904837571	0	50
10828	GO:0048016	1	0.980158332	0	10
10829	GO:0048017	1	0.992011772	0	4
10830	GO:0048018	1	0.935437993	0	33
10831	GO:0048019	1	0.974052171	0	13
10832	GO:0048020	1	0.971851414	0	14
10833	GO:0048021	1	0.99402527	0	3
10834	GO:0048022	1	0.992029478	0	4
10835	GO:0048023	1	0.986086602	0	7
10836	GO:0048024	1	0.962596406	0	19
10837	GO:0048025	1	0.956682612	0	22
10838	GO:0048026	1	0.962509361	0	19
10839	GO:0048027	1	0.948926662	0	26
10840	GO:0048029	1	0.980062659	0	10
10841	GO:0048030	1	0.997972973	0	1
10842	GO:0048034	1	0.997994374	0	1
10843	GO:0048038	1	0.974038321	0	13
10844	GO:0048039	1	0.985917465	0	7
10845	GO:0048040	1	0.997980136	0	1
10846	GO:0048041	1	0.949306896	0	26
10847	GO:0048048	1	0.982062758	0	9
10848	GO:0048050	1	0.993977589	0	3
10849	GO:0048066	1	0.978190261	0	11
10850	GO:0048069	1	0.997979233	0	1
10851	GO:0048070	1	0.9940187	0	3
10852	GO:0048073	1	0.998010369	0	1
10853	GO:0048074	1	0.997979743	0	1
10854	GO:0048075	1	0.998010073	0	1
10855	GO:0048087	1	0.995966431	0	2
10856	GO:0048096	1	0.976211395	0	12
10857	GO:0048101	1	0.993982997	0	3
10858	GO:0048102	1	0.986008689	0	7
10859	GO:0048103	1	0.9821233	0	9
10860	GO:0048105	1	0.994042092	0	3
10861	GO:0048132	1	0.997990564	0	1
10862	GO:0048133	1	0.993966853	0	3
10863	GO:0048137	1	0.997976314	0	1
10864	GO:0048143	1	0.972178098	0	14
10865	GO:0048144	1	0.995998446	0	2
10866	GO:0048145	1	0.994022561	0	3
10867	GO:0048146	1	0.91712523	0	43
10868	GO:0048147	1	0.950914933	0	25

10869	GO:0048148	1	0.976155483	0	12
10870	GO:0048149	1	0.988026756	0	6
10871	GO:0048156	1	0.91545935	0	44
10872	GO:0048160	1	0.996029064	0	2
10873	GO:0048167	1	0.901044654	0	52
10874	GO:0048168	1	0.96844526	0	16
10875	GO:0048169	1	0.968379355	0	16
10876	GO:0048170	1	0.990051798	0	5
10877	GO:0048172	1	0.978181139	0	11
10878	GO:0048179	1	0.988080553	0	6
10879	GO:0048185	1	0.972319979	0	14
10880	GO:0048188	1	0.976187581	0	12
10881	GO:0048193	1	0.960582748	0	20
10882	GO:0048194	1	0.992035763	0	4
10883	GO:0048199	1	0.998008841	0	1
10884	GO:0048203	1	0.995973647	0	2
10885	GO:0048205	1	0.988011383	0	6
10886	GO:0048208	1	0.880930515	0	63
10887	GO:0048209	1	0.998013215	0	1
10888	GO:0048210	1	0.991956551	0	4
10889	GO:0048211	1	0.99600103	0	2
10890	GO:0048213	1	0.995970263	0	2
10891	GO:0048219	1	0.998012654	0	1
10892	GO:0048227	1	0.984034092	0	8
10893	GO:0048232	1	0.995998862	0	2
10894	GO:0048237	1	0.99595541	0	2
10895	GO:0048239	1	0.935543421	0	33
10896	GO:0048240	1	0.964292786	0	18
10897	GO:0048241	1	0.997976127	0	1
10898	GO:0048242	1	0.997963664	0	1
10899	GO:0048243	1	0.998013251	0	1
10900	GO:0048244	1	0.997972849	0	1
10901	GO:0048245	1	0.983893481	0	8
10902	GO:0048246	1	0.975895488	0	12
10903	GO:0048247	1	0.963960864	0	18
10905	GO:0048250	1	0.995951143	0	2
10906	GO:0048251	1	0.978231162	0	11
10907	GO:0048252	1	0.993949669	0	3
10908	GO:0048254	1	0.989919633	0	5
10909	GO:0048255	1	0.941505071	0	30
10910	GO:0048256	1	0.995983931	0	2
10911	GO:0048257	1	0.99597972	0	2
10912	GO:0048259	1	0.995988504	0	2
10913	GO:0048260	1	0.964507868	0	18
10914	GO:0048261	1	0.98790281	0	6

10915	GO:0048263	1	0.993978313	0	3
10916	GO:0048264	1	0.997994512	0	1
10917	GO:0048265	1	0.976138846	0	12
10918	GO:0048266	1	0.980113419	0	10
10919	GO:0048268	1	0.966479685	0	17
10920	GO:0048269	1	0.995975588	0	2
10921	GO:0048270	1	0.997980014	0	1
10922	GO:0048273	1	0.989998903	0	5
10923	GO:0048278	1	0.958671232	0	21
10924	GO:0048280	1	0.984009969	0	8
10925	GO:0048284	1	0.995942988	0	2
10926	GO:0048286	1	0.937818043	0	32
10927	GO:0048290	1	0.997999503	0	1
10928	GO:0048291	1	0.997983728	0	1
10929	GO:0048294	1	0.994000922	0	3
10930	GO:0048295	1	0.995986601	0	2
10931	GO:0048297	1	0.997980805	0	1
10932	GO:0048298	1	0.99197946	0	4
10933	GO:0048302	1	0.992000118	0	4
10934	GO:0048304	1	0.988036144	0	6
10935	GO:0048306	1	0.860902142	0	74
10936	GO:0048311	1	0.984166508	0	8
10937	GO:0048312	1	0.984090987	0	8
10938	GO:0048318	1	0.989949467	0	5
10939	GO:0048319	1	0.998013437	0	1
10940	GO:0048320	1	0.99800715	0	1
10941	GO:0048332	1	0.996029539	0	2
10942	GO:0048333	1	0.976295013	0	12
10943	GO:0048337	1	0.997993311	0	1
10944	GO:0048339	1	0.988069509	0	6
10945	GO:0048340	1	0.996030731	0	2
10946	GO:0048341	1	0.986039723	0	7
10947	GO:0048343	1	0.995970313	0	2
10948	GO:0048352	1	0.998003307	0	1
10949	GO:0048368	1	0.991984936	0	4
10950	GO:0048378	1	0.998003307	0	1
10951	GO:0048382	1	0.988015494	0	6
10952	GO:0048384	1	0.966452494	0	17
10953	GO:0048385	1	0.991955936	0	4
10954	GO:0048386	1	0.989994365	0	5
10955	GO:0048387	1	0.986001484	0	7
10956	GO:0048388	1	0.993959031	0	3
10957	GO:0048389	1	0.997976774	0	1
10958	GO:0048392	1	0.997974922	0	1
10959	GO:0048403	1	0.99404481	0	3

10960	GO:0048406	1	0.992045229	0	4
10961	GO:0048407	1	0.978311815	0	11
10962	GO:0048408	1	0.991952676	0	4
10963	GO:0048468	1	0.952881591	0	24
10964	GO:0048469	1	0.945124679	0	28
10966	GO:0048476	1	0.989925586	0	5
10967	GO:0048477	1	0.935792379	0	33
10968	GO:0048478	1	0.982090857	0	9
10969	GO:0048484	1	0.980102407	0	10
10970	GO:0048485	1	0.978178854	0	11
10971	GO:0048487	1	0.920811925	0	41
10972	GO:0048488	1	0.928456182	0	37
10973	GO:0048489	1	0.968408689	0	16
10974	GO:0048490	1	0.968306645	0	16
10975	GO:0048495	1	0.98808637	0	6
10976	GO:0048496	1	0.990051884	0	5
10977	GO:0048499	1	0.991961986	0	4
10978	GO:0048500	1	0.98792741	0	6
10979	GO:0048505	1	0.991973394	0	4
10980	GO:0048511	1	0.75790605	0	138
10981	GO:0048512	1	0.986064744	0	7
10982	GO:0048513	1	0.976121221	0	12
10983	GO:0048514	1	0.949279331	0	26
10984	GO:0048515	1	0.984040557	0	8
10985	GO:0048520	1	0.997992028	0	1
10986	GO:0048523	1	0.998009727	0	1
10987	GO:0048524	1	0.991988308	0	4
10988	GO:0048525	1	0.991935765	0	4
10989	GO:0048534	1	0.99599243	0	2
10990	GO:0048535	1	0.970259933	0	15
10991	GO:0048536	1	0.951028775	0	25
10992	GO:0048538	1	0.913593789	0	45
10993	GO:0048539	1	0.986049272	0	7
10994	GO:0048541	1	0.992011118	0	4
10995	GO:0048545	1	0.945245287	0	28
10996	GO:0048546	1	0.976195736	0	12
10997	GO:0048549	1	0.990006877	0	5
10998	GO:0048550	1	0.991969675	0	4
10999	GO:0048557	1	0.978109888	0	11
11000	GO:0048560	1	0.997981567	0	1
11001	GO:0048561	1	0.997982211	0	1
11002	GO:0048562	1	0.996013294	0	2
11003	GO:0048563	1	0.996030003	0	2
11004	GO:0048565	1	0.95503678	0	23
11005	GO:0048566	1	0.97224967	0	14

11006	GO:0048568	1	0.921030791	0	41
11007	GO:0048569	1	0.997980071	0	1
11008	GO:0048570	1	0.990027222	0	5
11009	GO:0048588	1	0.995993876	0	2
11010	GO:0048589	1	0.951124696	0	25
11011	GO:0048592	1	0.994051875	0	3
11012	GO:0048593	1	0.958784419	0	21
11013	GO:0048596	1	0.97241637	0	14
11014	GO:0048597	1	0.993916427	0	3
11015	GO:0048598	1	0.960639463	0	20
11016	GO:0048599	1	0.974208804	0	13
11017	GO:0048608	1	0.98804332	0	6
11018	GO:0048609	1	0.994021149	0	3
11019	GO:0048611	1	0.997993836	0	1
11020	GO:0048617	1	0.990051117	0	5
11021	GO:0048619	1	0.998005482	0	1
11022	GO:0048625	1	0.994017016	0	3
11023	GO:0048627	1	0.993991181	0	3
11024	GO:0048630	1	0.995982015	0	2
11025	GO:0048631	1	0.997999078	0	1
11026	GO:0048632	1	0.996008869	0	2
11027	GO:0048633	1	0.993986226	0	3
11028	GO:0048635	1	0.998003396	0	1
11029	GO:0048638	1	0.996012355	0	2
11030	GO:0048639	1	0.990039939	0	5
11031	GO:0048640	1	0.99801	0	1
11032	GO:0048641	1	0.996000349	0	2
11033	GO:0048642	1	0.996005227	0	2
11034	GO:0048643	1	0.984101381	0	8
11035	GO:0048644	1	0.990003668	0	5
11036	GO:0048645	1	0.991989699	0	4
11037	GO:0048646	1	0.968271749	0	16
11038	GO:0048659	1	0.992004727	0	4
11039	GO:0048660	1	0.980098594	0	10
11041	GO:0048662	1	0.954737276	0	23
11042	GO:0048663	1	0.968395689	0	16
11043	GO:0048664	1	0.993988918	0	3
11044	GO:0048665	1	0.976216426	0	12
11045	GO:0048666	1	0.89547368	0	55
11046	GO:0048667	1	0.982171945	0	9
11047	GO:0048668	1	0.990075334	0	5
11048	GO:0048669	1	0.996018547	0	2
11049	GO:0048670	1	0.998013356	0	1
11050	GO:0048671	1	0.986120761	0	7
11051	GO:0048672	1	0.984030292	0	8

11052	GO:0048675	1	0.949295732	0	26
11053	GO:0048677	1	0.998013437	0	1
11054	GO:0048678	1	0.948864506	0	26
11055	GO:0048679	1	0.986102925	0	7
11056	GO:0048680	1	0.983993894	0	8
11057	GO:0048681	1	0.978187022	0	11
11058	GO:0048685	1	0.997971376	0	1
11059	GO:0048691	1	0.998013437	0	1
11060	GO:0048692	1	0.998013437	0	1
11061	GO:0048694	1	0.998013437	0	1
11062	GO:0048697	1	0.995967081	0	2
11063	GO:0048698	1	0.997990564	0	1
11064	GO:0048699	1	0.978237932	0	11
11065	GO:0048701	1	0.943512417	0	29
11066	GO:0048702	1	0.984099942	0	8
11067	GO:0048703	1	0.982114249	0	9
11068	GO:0048704	1	0.941504876	0	30
11069	GO:0048705	1	0.9341837	0	34
11070	GO:0048706	1	0.953028617	0	24
11071	GO:0048708	1	0.988070627	0	6
11072	GO:0048709	1	0.954837175	0	23
11073	GO:0048710	1	0.996030731	0	2
11074	GO:0048711	1	0.980053675	0	10
11075	GO:0048712	1	0.982044677	0	9
11076	GO:0048713	1	0.991985075	0	4
11077	GO:0048714	1	0.962443535	0	19
11078	GO:0048715	1	0.982058049	0	9
11079	GO:0048729	1	0.990061546	0	5
11080	GO:0048730	1	0.988015914	0	6
11081	GO:0048731	1	0.997991563	0	1
11082	GO:0048732	1	0.997991811	0	1
11083	GO:0048733	1	0.988039147	0	6
11084	GO:0048738	1	0.972304063	0	14
11085	GO:0048739	1	0.984107206	0	8
11086	GO:0048741	1	0.96058418	0	20
11087	GO:0048742	1	0.991992671	0	4
11088	GO:0048743	1	0.992025058	0	4
11089	GO:0048745	1	0.978192389	0	11
11090	GO:0048747	1	0.974273728	0	13
11091	GO:0048752	1	0.99202768	0	4
11092	GO:0048753	1	0.998005976	0	1
11093	GO:0048754	1	0.949229685	0	26
11094	GO:0048755	1	0.990046844	0	5
11095	GO:0048757	1	0.997960596	0	1
11096	GO:0048762	1	0.982138745	0	9

11098	GO:0048769	1	0.993998111	0	3
11099	GO:0048771	1	0.982109865	0	9
11100	GO:0048786	1	0.921106566	0	41
11101	GO:0048787	1	0.978191126	0	11
11102	GO:0048788	1	0.986174576	0	7
11103	GO:0048789	1	0.99801271	0	1
11104	GO:0048790	1	0.988082826	0	6
11105	GO:0048791	1	0.98016979	0	10
11106	GO:0048792	1	0.998000781	0	1
11107	GO:0048793	1	0.990010186	0	5
11108	GO:0048806	1	0.986076846	0	7
11109	GO:0048807	1	0.99801181	0	1
11110	GO:0048808	1	0.996012325	0	2
11111	GO:0048812	1	0.868961173	0	70
11112	GO:0048813	1	0.928603471	0	37
11113	GO:0048814	1	0.962765125	0	19
11114	GO:0048820	1	0.990010621	0	5
11115	GO:0048821	1	0.96253407	0	19
11116	GO:0048822	1	0.995998098	0	2
11117	GO:0048839	1	0.920925603	0	41
11118	GO:0048840	1	0.99799807	0	1
11119	GO:0048841	1	0.994011947	0	3
11120	GO:0048842	1	0.99006746	0	5
11121	GO:0048843	1	0.949376883	0	26
11122	GO:0048844	1	0.95679133	0	22
11123	GO:0048845	1	0.988066406	0	6
11124	GO:0048846	1	0.984175257	0	8
11125	GO:0048850	1	0.998013437	0	1
11126	GO:0048852	1	0.997981308	0	1
11127	GO:0048853	1	0.984031991	0	8
11128	GO:0048854	1	0.956973038	0	22
11129	GO:0048855	1	0.998013437	0	1
11130	GO:0048856	1	0.900827121	0	52
11131	GO:0048857	1	0.997971629	0	1
11132	GO:0048858	1	0.993986876	0	3
11133	GO:0048859	1	0.995988589	0	2
11134	GO:0048861	1	0.98207439	0	9
11135	GO:0048863	1	0.949100579	0	26
11136	GO:0048864	1	0.98802001	0	6
11137	GO:0048866	1	0.997973469	0	1
11138	GO:0048869	1	0.998012707	0	1
11139	GO:0048870	1	0.928348733	0	37
11140	GO:0048871	1	0.993975423	0	3
11141	GO:0048872	1	0.956690558	0	22
11142	GO:0048873	1	0.956733832	0	22

11143	GO:0048874	1	0.996020098	0	2
11144	GO:0048875	1	0.995990019	0	2
11145	GO:0048880	1	0.996007256	0	2
11146	GO:0048935	1	0.986110394	0	7
11147	GO:0048936	1	0.995998306	0	2
11148	GO:0050011	1	0.997971521	0	1
11149	GO:0050023	1	0.99797628	0	1
11150	GO:0050031	1	0.997985293	0	1
11151	GO:0050038	1	0.997956897	0	1
11152	GO:0050046	1	0.997990564	0	1
11153	GO:0050048	1	0.995989427	0	2
11154	GO:0050051	1	0.989977197	0	5
11155	GO:0050061	1	0.998005077	0	1
11156	GO:0050062	1	0.997987281	0	1
11157	GO:0050072	1	0.98403963	0	8
11158	GO:0050080	1	0.997981761	0	1
11159	GO:0050104	1	0.99796988	0	1
11160	GO:0050113	1	0.997968788	0	1
11161	GO:0050115	1	0.998012626	0	1
11162	GO:0050121	1	0.997969419	0	1
11163	GO:0050124	1	0.998005384	0	1
11164	GO:0050135	1	0.972296546	0	14
11165	GO:0050136	1	0.997986438	0	1
11167	GO:0050152	1	0.997965639	0	1
11168	GO:0050153	1	0.997981849	0	1
11169	GO:0050155	1	0.997978312	0	1
11170	GO:0050178	1	0.995908518	0	2
11171	GO:0050185	1	0.998013437	0	1
11172	GO:0050196	1	0.996028514	0	2
11173	GO:0050197	1	0.995993334	0	2
11174	GO:0050200	1	0.996024327	0	2
11175	GO:0050201	1	0.997990564	0	1
11176	GO:0050207	1	0.997990564	0	1
11177	GO:0050211	1	0.993987166	0	3
11178	GO:0050213	1	0.997982868	0	1
11179	GO:0050218	1	0.992000153	0	4
11180	GO:0050220	1	0.99393564	0	3
11181	GO:0050221	1	0.997965025	0	1
11182	GO:0050251	1	0.995968553	0	2
11183	GO:0050252	1	0.995994325	0	2
11184	GO:0050253	1	0.992012658	0	4
11185	GO:0050254	1	0.995960543	0	2
11186	GO:0050255	1	0.997987078	0	1
11187	GO:0050262	1	0.997990564	0	1
11188	GO:0050265	1	0.993984704	0	3

11189	GO:0050277	1	0.998005904	0	1
11190	GO:0050290	1	0.998012218	0	1
11191	GO:0050291	1	0.986016556	0	7
11192	GO:0050294	1	0.991907414	0	4
11193	GO:0050295	1	0.995992346	0	2
11194	GO:0050313	1	0.997959564	0	1
11195	GO:0050321	1	0.956936403	0	22
11196	GO:0050333	1	0.997975129	0	1
11197	GO:0050337	1	0.997951841	0	1
11198	GO:0050346	1	0.997990564	0	1
11199	GO:0050347	1	0.99597848	0	2
11200	GO:0050353	1	0.997992183	0	1
11201	GO:0050354	1	0.997990564	0	1
11202	GO:0050355	1	0.998011042	0	1
11203	GO:0050405	1	0.996030372	0	2
11204	GO:0050421	1	0.992006384	0	4
11205	GO:0050427	1	0.966171293	0	17
11206	GO:0050428	1	0.988024992	0	6
11207	GO:0050429	1	0.996030522	0	2
11208	GO:0050431	1	0.956920364	0	22
11209	GO:0050434	1	0.952685635	0	24
11210	GO:0050435	1	0.976233422	0	12
11211	GO:0050436	1	0.994044867	0	3
11212	GO:0050459	1	0.997990564	0	1
11213	GO:0050462	1	0.997964968	0	1
11214	GO:0050473	1	0.993974509	0	3
11215	GO:0050479	1	0.997990564	0	1
11216	GO:0050480	1	0.997980886	0	1
11217	GO:0050482	1	0.966239916	0	17
11218	GO:0050483	1	0.997959086	0	1
11219	GO:0050484	1	0.998013312	0	1
11220	GO:0050486	1	0.997999237	0	1
11221	GO:0050501	1	0.993978253	0	3
11222	GO:0050508	1	0.99402378	0	3
11223	GO:0050509	1	0.996008274	0	2
11224	GO:0050510	1	0.992037752	0	4
11225	GO:0050512	1	0.997980071	0	1
11226	GO:0050528	1	0.997984931	0	1
11227	GO:0050543	1	0.997962172	0	1
11228	GO:0050544	1	0.989867665	0	5
11229	GO:0050560	1	0.997999739	0	1
11230	GO:0050561	1	0.998006752	0	1
11231	GO:0050566	1	0.997990564	0	1
11232	GO:0050567	1	0.993959391	0	3
11233	GO:0050568	1	0.997992902	0	1

11234	GO:0050571	1	0.997971864	0	1
11235	GO:0050577	1	0.997990564	0	1
11236	GO:0050591	1	0.997990913	0	1
11237	GO:0050610	1	0.995929731	0	2
11238	GO:0050613	1	0.995980813	0	2
11239	GO:0050614	1	0.998009932	0	1
11240	GO:0050632	1	0.997968397	0	1
11241	GO:0050633	1	0.993937897	0	3
11242	GO:0050646	1	0.99797447	0	1
11243	GO:0050647	1	0.99797447	0	1
11244	GO:0050648	1	0.99797447	0	1
11245	GO:0050649	1	0.9919499	0	4
11246	GO:0050650	1	0.984076776	0	8
11247	GO:0050651	1	0.993992247	0	3
11248	GO:0050652	1	0.998004561	0	1
11249	GO:0050653	1	0.996015567	0	2
11250	GO:0050655	1	0.997981744	0	1
11251	GO:0050656	1	0.987995083	0	6
11252	GO:0050658	1	0.980179826	0	10
11253	GO:0050659	1	0.99602927	0	2
11254	GO:0050660	1	0.875671842	0	66
11256	GO:0050664	1	0.998010552	0	1
11257	GO:0050665	1	0.982029342	0	9
11258	GO:0050666	1	0.997986657	0	1
11259	GO:0050667	1	0.988073322	0	6
11260	GO:0050668	1	0.997982988	0	1
11261	GO:0050671	1	0.990025875	0	5
11262	GO:0050672	1	0.995985661	0	2
11263	GO:0050673	1	0.980191397	0	10
11264	GO:0050678	1	0.974280757	0	13
11265	GO:0050679	1	0.889903064	0	58
11266	GO:0050680	1	0.884566994	0	61
11267	GO:0050681	1	0.947262051	0	27
11268	GO:0050682	1	0.994020989	0	3
11269	GO:0050683	1	0.998008583	0	1
11270	GO:0050684	1	0.974287158	0	13
11271	GO:0050687	1	0.980102978	0	10
11273	GO:0050689	1	0.993953104	0	3
11274	GO:0050690	1	0.950931509	0	25
11275	GO:0050691	1	0.986030708	0	7
11276	GO:0050692	1	0.991994521	0	4
11277	GO:0050693	1	0.988045474	0	6
11278	GO:0050694	1	0.995960114	0	2
11279	GO:0050698	1	0.997987219	0	1
11280	GO:0050699	1	0.941355502	0	30

11281	GO:0050700	1	0.970275252	0	15
11282	GO:0050708	1	0.968355593	0	16
11284	GO:0050714	1	0.919054808	0	42
11285	GO:0050727	1	0.847682277	0	82
11286	GO:0050728	1	0.842376537	0	85
11287	GO:0050729	1	0.859504495	0	75
11288	GO:0050730	1	0.960511012	0	20
11289	GO:0050731	1	0.846260436	0	83
11290	GO:0050732	1	0.974170007	0	13
11291	GO:0050733	1	0.993984411	0	3
11292	GO:0050746	1	0.995998681	0	2
11293	GO:0050747	1	0.997960141	0	1
11294	GO:0050748	1	0.996029964	0	2
11295	GO:0050750	1	0.958646609	0	21
11296	GO:0050764	1	0.982120473	0	9
11297	GO:0050765	1	0.962444168	0	19
11298	GO:0050766	1	0.926083487	0	38
11299	GO:0050767	1	0.933961856	0	34
11300	GO:0050768	1	0.954817041	0	23
11301	GO:0050769	1	0.939648795	0	31
11302	GO:0050770	1	0.955034272	0	23
11303	GO:0050771	1	0.962602709	0	19
11304	GO:0050772	1	0.932293011	0	35
11305	GO:0050773	1	0.962617288	0	19
11306	GO:0050774	1	0.98406687	0	8
11307	GO:0050775	1	0.960720134	0	20
11309	GO:0050777	1	0.982050399	0	9
11311	GO:0050779	1	0.991997104	0	4
11312	GO:0050780	1	0.99797867	0	1
11313	GO:0050785	1	0.997966894	0	1
11314	GO:0050786	1	0.981783261	0	9
11315	GO:0050787	1	0.995975935	0	2
11316	GO:0050790	1	0.588420199	0	263
11317	GO:0050792	1	0.987932073	0	6
11318	GO:0050793	1	0.99207686	0	4
11319	GO:0050794	1	0.996026485	0	2
11320	GO:0050795	1	0.98803134	0	6
11321	GO:0050796	1	0.89175723	0	57
11322	GO:0050798	1	0.992027593	0	4
11323	GO:0050801	1	0.970542552	0	15
11325	GO:0050804	1	0.863832082	0	73
11326	GO:0050805	1	0.986058421	0	7
11327	GO:0050806	1	0.966456775	0	17
11328	GO:0050807	1	0.952914086	0	24
11330	GO:0050810	1	0.982019605	0	9

11331	GO:0050811	1	0.97814786	0	11
11332	GO:0050812	1	0.997996934	0	1
11333	GO:0050815	1	0.991968603	0	4
11334	GO:0050816	1	0.993990193	0	3
11335	GO:0050819	1	0.991920681	0	4
11336	GO:0050820	1	0.998013437	0	1
11337	GO:0050821	1	0.690987809	0	183
11338	GO:0050823	1	0.998001741	0	1
11339	GO:0050828	1	0.997990564	0	1
11340	GO:0050829	1	0.905580393	0	49
11341	GO:0050830	1	0.873178719	0	67
11342	GO:0050832	1	0.963991879	0	18
11343	GO:0050833	1	0.991982272	0	4
11344	GO:0050839	1	0.881388015	0	63
11345	GO:0050840	1	0.945448477	0	28
11346	GO:0050847	1	0.982179288	0	9
11348	GO:0050849	1	0.992013985	0	4
11349	GO:0050850	1	0.978012544	0	11
11350	GO:0050851	1	0.987968985	0	6
11351	GO:0050852	1	0.722013644	0	161
11352	GO:0050853	1	0.937792132	0	32
11353	GO:0050855	1	0.980138653	0	10
11354	GO:0050856	1	0.992040101	0	4
11355	GO:0050857	1	0.998012965	0	1
11356	GO:0050859	1	0.989958416	0	5
11358	GO:0050861	1	0.984073313	0	8
11359	GO:0050862	1	0.972131373	0	14
11360	GO:0050863	1	0.978113621	0	11
11361	GO:0050864	1	0.993956326	0	3
11362	GO:0050865	1	0.99801336	0	1
11363	GO:0050866	1	0.998006897	0	1
11364	GO:0050867	1	0.991962986	0	4
11365	GO:0050868	1	0.972289419	0	14
11366	GO:0050869	1	0.986020386	0	7
11367	GO:0050870	1	0.958535545	0	21
11368	GO:0050871	1	0.980101765	0	10
11369	GO:0050872	1	0.980139836	0	10
11370	GO:0050873	1	0.945114875	0	28
11371	GO:0050877	1	0.920771586	0	41
11372	GO:0050878	1	0.992049438	0	4
11373	GO:0050881	1	0.990038453	0	5
11374	GO:0050882	1	0.988102864	0	6
11375	GO:0050883	1	0.997986219	0	1
11376	GO:0050884	1	0.976225491	0	12
11377	GO:0050885	1	0.936039643	0	33

11378	GO:0050890	1	0.920955606	0	41
11379	GO:0050891	1	0.980173308	0	10
11380	GO:0050892	1	0.974259768	0	13
11381	GO:0050893	1	0.993961369	0	3
11382	GO:0050896	1	0.614919338	0	239
11383	GO:0050897	1	0.99199882	0	4
11384	GO:0050900	1	0.788662254	0	118
11385	GO:0050901	1	0.960643804	0	20
11386	GO:0050902	1	0.995989455	0	2
11387	GO:0050904	1	0.991986272	0	4
11388	GO:0050905	1	0.958650664	0	21
11389	GO:0050906	1	0.995995838	0	2
11390	GO:0050907	1	0.991893585	0	4
11391	GO:0050908	1	0.962622137	0	19
11392	GO:0050909	1	0.942870917	0	29
11393	GO:0050910	1	0.970312361	0	15
11394	GO:0050911	1	0.805118962	0	106
11395	GO:0050912	1	0.995975586	0	2
11396	GO:0050913	1	0.990001843	0	5
11397	GO:0050915	1	0.992008422	0	4
11398	GO:0050916	1	0.989977521	0	5
11399	GO:0050917	1	0.989978305	0	5
11401	GO:0050919	1	0.926883575	0	38
11402	GO:0050920	1	0.986049505	0	7
11403	GO:0050921	1	0.970140507	0	15
11404	GO:0050922	1	0.995986117	0	2
11405	GO:0050923	1	0.998000404	0	1
11406	GO:0050925	1	0.996030731	0	2
11407	GO:0050927	1	0.97821161	0	11
11408	GO:0050928	1	0.998013263	0	1
11409	GO:0050929	1	0.998012848	0	1
11410	GO:0050930	1	0.97404984	0	13
11411	GO:0050942	1	0.995985075	0	2
11412	GO:0050951	1	0.990104979	0	5
11413	GO:0050953	1	0.980172962	0	10
11414	GO:0050954	1	0.995969177	0	2
11415	GO:0050955	1	0.996026083	0	2
11416	GO:0050957	1	0.988071315	0	6
11417	GO:0050960	1	0.998008787	0	1
11418	GO:0050961	1	0.997990564	0	1
11419	GO:0050965	1	0.98008916	0	10
11420	GO:0050966	1	0.980124802	0	10
11421	GO:0050968	1	0.996003262	0	2
11422	GO:0050973	1	0.998013437	0	1
11423	GO:0050974	1	0.989975075	0	5

11424	GO:0050975	1	0.995953039	0	2
11425	GO:0050976	1	0.998013437	0	1
11426	GO:0050982	1	0.976272182	0	12
11427	GO:0050992	1	0.997980805	0	1
11428	GO:0050994	1	0.993921356	0	3
11429	GO:0050995	1	0.970276627	0	15
11430	GO:0050996	1	0.992012	0	4
11431	GO:0050998	1	0.972338852	0	14
11432	GO:0050999	1	0.958634633	0	21
11433	GO:0051000	1	0.960621396	0	20
11434	GO:0051001	1	0.98397059	0	8
11435	GO:0051004	1	0.990007388	0	5
11436	GO:0051005	1	0.991972115	0	4
11437	GO:0051006	1	0.987901198	0	6
11438	GO:0051008	1	0.997977743	0	1
11439	GO:0051010	1	0.960836946	0	20
11440	GO:0051011	1	0.974303593	0	13
11441	GO:0051012	1	0.996030431	0	2
11442	GO:0051013	1	0.976233094	0	12
11443	GO:0051014	1	0.974207237	0	13
11444	GO:0051015	1	0.676686042	0	194
11445	GO:0051016	1	0.958694009	0	21
11446	GO:0051017	1	0.932208742	0	35
11447	GO:0051018	1	0.956909711	0	22
11448	GO:0051019	1	0.945338077	0	28
11449	GO:0051020	1	0.932036727	0	35
11450	GO:0051021	1	0.99796988	0	1
11451	GO:0051022	1	0.993987085	0	3
11452	GO:0051026	1	0.988016013	0	6
11454	GO:0051029	1	0.99796274	0	1
11455	GO:0051031	1	0.997970704	0	1
11456	GO:0051032	1	0.998006968	0	1
11457	GO:0051033	1	0.994044922	0	3
11458	GO:0051036	1	0.994002074	0	3
11459	GO:0051039	1	0.99799824	0	1
11460	GO:0051041	1	0.997998423	0	1
11461	GO:0051042	1	0.997996626	0	1
11462	GO:0051044	1	0.970306365	0	15
11463	GO:0051045	1	0.984060732	0	8
11464	GO:0051046	1	0.996012911	0	2
11465	GO:0051047	1	0.992022827	0	4
11466	GO:0051048	1	0.994007871	0	3
11467	GO:0051049	1	0.994015448	0	3
11468	GO:0051050	1	0.997980169	0	1
11469	GO:0051051	1	0.991929914	0	4

11470	GO:0051053	1	0.998001782	0	1
11471	GO:0051054	1	0.995938446	0	2
11472	GO:0051055	1	0.992019065	0	4
11473	GO:0051056	1	0.762762759	0	135
11474	GO:0051057	1	0.988112568	0	6
11475	GO:0051058	1	0.986102219	0	7
11476	GO:0051059	1	0.939457462	0	31
11477	GO:0051066	1	0.995954433	0	2
11478	GO:0051068	1	0.998002843	0	1
11479	GO:0051081	1	0.990009688	0	5
11480	GO:0051082	1	0.807092813	0	106
11481	GO:0051083	1	0.995960379	0	2
11482	GO:0051084	1	0.991977408	0	4
11483	GO:0051085	1	0.939393502	0	31
11484	GO:0051086	1	0.99797802	0	1
11485	GO:0051087	1	0.810679762	0	104
11486	GO:0051089	1	0.998006948	0	1
11487	GO:0051090	1	0.951081999	0	25
11489	GO:0051092	1	0.734463457	0	153
11490	GO:0051096	1	0.988029295	0	6
11492	GO:0051098	1	0.992045496	0	4
11493	GO:0051099	1	0.987963955	0	6
11494	GO:0051100	1	0.995971317	0	2
11495	GO:0051101	1	0.987898743	0	6
11496	GO:0051102	1	0.998008075	0	1
11497	GO:0051103	1	0.992000572	0	4
11498	GO:0051106	1	0.991997243	0	4
11499	GO:0051117	1	0.844315442	0	84
11500	GO:0051119	1	0.997990564	0	1
11501	GO:0051120	1	0.995992687	0	2
11502	GO:0051121	1	0.99798452	0	1
11503	GO:0051122	1	0.981968304	0	9
11504	GO:0051123	1	0.980023832	0	10
11505	GO:0051124	1	0.994039845	0	3
11506	GO:0051125	1	0.995985036	0	2
11507	GO:0051126	1	0.993965165	0	3
11508	GO:0051127	1	0.992029934	0	4
11509	GO:0051128	1	0.994036432	0	3
11510	GO:0051130	1	0.998013428	0	1
11511	GO:0051131	1	0.958440063	0	21
11512	GO:0051135	1	0.991949211	0	4
11513	GO:0051136	1	0.99598927	0	2
11514	GO:0051138	1	0.992039258	0	4
11515	GO:0051139	1	0.998006135	0	1
11516	GO:0051141	1	0.998003192	0	1

11517	GO:0051142	1	0.993935161	0	3
11518	GO:0051145	1	0.972308518	0	14
11519	GO:0051146	1	0.966532567	0	17
11520	GO:0051147	1	0.988065666	0	6
11521	GO:0051148	1	0.982073141	0	9
11522	GO:0051149	1	0.956882625	0	22
11523	GO:0051150	1	0.992030324	0	4
11524	GO:0051151	1	0.98605816	0	7
11525	GO:0051152	1	0.989960293	0	5
11526	GO:0051153	1	0.996026918	0	2
11527	GO:0051154	1	0.995963397	0	2
11528	GO:0051155	1	0.985946626	0	7
11529	GO:0051156	1	0.982096591	0	9
11530	GO:0051160	1	0.997987078	0	1
11531	GO:0051164	1	0.997987078	0	1
11532	GO:0051168	1	0.970406134	0	15
11533	GO:0051169	1	0.986059211	0	7
11534	GO:0051170	1	0.95834549	0	21
11535	GO:0051173	1	0.997972866	0	1
11536	GO:0051177	1	0.989957781	0	5
11537	GO:0051180	1	0.989986735	0	5
11538	GO:0051204	1	0.993981236	0	3
11539	GO:0051205	1	0.983996011	0	8
11540	GO:0051208	1	0.990024161	0	5
11542	GO:0051213	1	0.846204563	0	83
11543	GO:0051216	1	0.875782869	0	66
11544	GO:0051219	1	0.9302599	0	36
11545	GO:0051220	1	0.98608143	0	7
11546	GO:0051222	1	0.988015039	0	6
11547	GO:0051223	1	0.98601801	0	7
11548	GO:0051224	1	0.988012058	0	6
11549	GO:0051225	1	0.93027793	0	36
11550	GO:0051228	1	0.99602328	0	2
11551	GO:0051233	1	0.950955158	0	25
11552	GO:0051238	1	0.997948824	0	1
11553	GO:0051246	1	0.982076705	0	9
11554	GO:0051247	1	0.980065988	0	10
11555	GO:0051248	1	0.996004507	0	2
11556	GO:0051249	1	0.995980971	0	2
11557	GO:0051250	1	0.994034535	0	3
11558	GO:0051251	1	0.997985434	0	1
11559	GO:0051252	1	0.980119728	0	10
11560	GO:0051255	1	0.993965121	0	3
11561	GO:0051256	1	0.986008209	0	7
11562	GO:0051257	1	0.995998977	0	2

11563	GO:0051258	1	0.976020043	0	12
11564	GO:0051259	1	0.964474652	0	18
11565	GO:0051260	1	0.822790708	0	97
11566	GO:0051261	1	0.998000052	0	1
11567	GO:0051262	1	0.960719915	0	20
11568	GO:0051264	1	0.997991424	0	1
11569	GO:0051265	1	0.997991424	0	1
11570	GO:0051267	1	0.99799807	0	1
11571	GO:0051270	1	0.998012834	0	1
11572	GO:0051271	1	0.988051834	0	6
11573	GO:0051272	1	0.98013035	0	10
11574	GO:0051276	1	0.945251899	0	28
11575	GO:0051279	1	0.972257952	0	14
11576	GO:0051280	1	0.995945607	0	2
11578	GO:0051282	1	0.995983129	0	2
11579	GO:0051283	1	0.997993242	0	1
11580	GO:0051284	1	0.993975557	0	3
11581	GO:0051286	1	0.992031387	0	4
11582	GO:0051287	1	0.926232929	0	38
11584	GO:0051290	1	0.970369265	0	15
11585	GO:0051291	1	0.984062521	0	8
11586	GO:0051292	1	0.982118748	0	9
11587	GO:0051293	1	0.989918928	0	5
11588	GO:0051294	1	0.990082587	0	5
11589	GO:0051295	1	0.992061644	0	4
11590	GO:0051298	1	0.968538892	0	16
11591	GO:0051299	1	0.995989609	0	2
11592	GO:0051301	1	0.43712011	0	408
11593	GO:0051302	1	0.97807923	0	11
11594	GO:0051303	1	0.995991531	0	2
11595	GO:0051304	1	0.990073085	0	5
11596	GO:0051306	1	0.994000588	0	3
11597	GO:0051307	1	0.998013437	0	1
11598	GO:0051308	1	0.997990564	0	1
11599	GO:0051309	1	0.99797426	0	1
11600	GO:0051310	1	0.976160749	0	12
11601	GO:0051311	1	0.997990564	0	1
11602	GO:0051315	1	0.978108319	0	11
11603	GO:0051321	1	0.79959837	0	111
11604	GO:0051341	1	0.997970913	0	1
11605	GO:0051343	1	0.991972883	0	4
11606	GO:0051344	1	0.989999784	0	5
11607	GO:0051345	1	0.992011327	0	4
11608	GO:0051346	1	0.988024053	0	6
11609	GO:0051349	1	0.998013437	0	1

11610	GO:0051350	1	0.997964583	0	1
11611	GO:0051351	1	0.99193297	0	4
11612	GO:0051353	1	0.988048329	0	6
11613	GO:0051354	1	0.9879657	0	6
11614	GO:0051365	1	0.995989121	0	2
11615	GO:0051371	1	0.97229502	0	14
11616	GO:0051373	1	0.993945783	0	3
11617	GO:0051377	1	0.991994386	0	4
11618	GO:0051378	1	0.987995072	0	6
11619	GO:0051379	1	0.989972512	0	5
11620	GO:0051380	1	0.993964555	0	3
11621	GO:0051382	1	0.970262924	0	15
11622	GO:0051383	1	0.98800191	0	6
11623	GO:0051384	1	0.889569386	0	58
11624	GO:0051385	1	0.994023653	0	3
11625	GO:0051386	1	0.996028198	0	2
11626	GO:0051387	1	0.993965346	0	3
11627	GO:0051388	1	0.986050971	0	7
11628	GO:0051389	1	0.9960299	0	2
11629	GO:0051391	1	0.998007125	0	1
11630	GO:0051393	1	0.972362831	0	14
11631	GO:0051400	1	0.991954778	0	4
11632	GO:0051402	1	0.922690492	0	40
11633	GO:0051403	1	0.947071953	0	27
11634	GO:0051409	1	0.992000705	0	4
11635	GO:0051410	1	0.995985075	0	2
11636	GO:0051412	1	0.970238617	0	15
11637	GO:0051413	1	0.997986797	0	1
11638	GO:0051414	1	0.99202547	0	4
11639	GO:0051415	1	0.997990564	0	1
11640	GO:0051418	1	0.998013437	0	1
11641	GO:0051425	1	0.994025607	0	3
11642	GO:0051427	1	0.99392822	0	3
11643	GO:0051428	1	0.988000419	0	6
11644	GO:0051429	1	0.99592797	0	2
11645	GO:0051430	1	0.993953488	0	3
11646	GO:0051431	1	0.993888556	0	3
11647	GO:0051433	1	0.998011412	0	1
11648	GO:0051434	1	0.987977202	0	6
11649	GO:0051438	1	0.994017576	0	3
11650	GO:0051443	1	0.962534061	0	19
11651	GO:0051444	1	0.982092558	0	9
11652	GO:0051445	1	0.995995299	0	2
11653	GO:0051447	1	0.991962388	0	4
11654	GO:0051450	1	0.991990278	0	4

11655	GO:0051451	1	0.982120232	0	9
11656	GO:0051452	1	0.991959312	0	4
11657	GO:0051453	1	0.947345804	0	27
11658	GO:0051454	1	0.992005034	0	4
11659	GO:0051455	1	0.997990564	0	1
11660	GO:0051457	1	0.97222639	0	14
11661	GO:0051461	1	0.991941153	0	4
11662	GO:0051463	1	0.998013437	0	1
11663	GO:0051464	1	0.99594951	0	2
11664	GO:0051466	1	0.995984795	0	2
11665	GO:0051480	1	0.947254569	0	27
11667	GO:0051482	1	0.950832415	0	25
11668	GO:0051489	1	0.974250748	0	13
11669	GO:0051490	1	0.989998753	0	5
11670	GO:0051491	1	0.945491379	0	28
11671	GO:0051492	1	0.96846114	0	16
11672	GO:0051493	1	0.951032224	0	25
11673	GO:0051494	1	0.997990564	0	1
11674	GO:0051495	1	0.989981081	0	5
11675	GO:0051496	1	0.900881434	0	52
11676	GO:0051497	1	0.951211001	0	25
11677	GO:0051499	1	0.995961922	0	2
11678	GO:0051500	1	0.995961922	0	2
11679	GO:0051503	1	0.995972207	0	2
11680	GO:0051525	1	0.994019283	0	3
11682	GO:0051537	1	0.956513931	0	22
11683	GO:0051538	1	0.993968113	0	3
11685	GO:0051541	1	0.996012159	0	2
11686	GO:0051542	1	0.998013437	0	1
11687	GO:0051545	1	0.997984394	0	1
11688	GO:0051546	1	0.989983495	0	5
11689	GO:0051548	1	0.996030709	0	2
11690	GO:0051549	1	0.978250618	0	11
11691	GO:0051552	1	0.987995355	0	6
11692	GO:0051560	1	0.968406235	0	16
11693	GO:0051561	1	0.978075139	0	11
11694	GO:0051562	1	0.994009458	0	3
11695	GO:0051563	1	0.996018678	0	2
11696	GO:0051567	1	0.982215971	0	9
11697	GO:0051568	1	0.958679368	0	21
11698	GO:0051569	1	0.984085578	0	8
11699	GO:0051570	1	0.994044875	0	3
11700	GO:0051571	1	0.968468728	0	16
11701	GO:0051572	1	0.988056597	0	6
11702	GO:0051573	1	0.988106404	0	6

11703	GO:0051574	1	0.988094129	0	6
11704	GO:0051575	1	0.988012295	0	6
11705	GO:0051580	1	0.9939627	0	3
11706	GO:0051582	1	0.997990564	0	1
11707	GO:0051583	1	0.991969924	0	4
11708	GO:0051584	1	0.993935213	0	3
11709	GO:0051585	1	0.997996934	0	1
11710	GO:0051586	1	0.993939032	0	3
11712	GO:0051592	1	0.898818311	0	53
11713	GO:0051593	1	0.985983877	0	7
11714	GO:0051594	1	0.995954156	0	2
11715	GO:0051595	1	0.998013437	0	1
11716	GO:0051597	1	0.990048716	0	5
11717	GO:0051598	1	0.993977886	0	3
11718	GO:0051599	1	0.994013785	0	3
11719	GO:0051601	1	0.99200459	0	4
11720	GO:0051602	1	0.95282132	0	24
11721	GO:0051603	1	0.911164133	0	46
11722	GO:0051604	1	0.964365784	0	18
11723	GO:0051606	1	0.995999152	0	2
11725	GO:0051608	1	0.998013429	0	1
11726	GO:0051610	1	0.990024885	0	5
11727	GO:0051611	1	0.998012669	0	1
11728	GO:0051612	1	0.993996	0	3
11729	GO:0051615	1	0.996010058	0	2
11730	GO:0051620	1	0.992005557	0	4
11731	GO:0051621	1	0.995976268	0	2
11732	GO:0051622	1	0.997996934	0	1
11733	GO:0051623	1	0.997975371	0	1
11734	GO:0051625	1	0.996010058	0	2
11735	GO:0051638	1	0.997990564	0	1
11736	GO:0051639	1	0.976273508	0	12
11737	GO:0051640	1	0.996004302	0	2
11738	GO:0051641	1	0.998013193	0	1
11739	GO:0051642	1	0.964586754	0	18
11740	GO:0051643	1	0.995950615	0	2
11741	GO:0051645	1	0.988059428	0	6
11742	GO:0051646	1	0.980131772	0	10
11743	GO:0051647	1	0.990012154	0	5
11744	GO:0051648	1	0.995997945	0	2
11745	GO:0051649	1	0.986020482	0	7
11746	GO:0051650	1	0.988016409	0	6
11747	GO:0051651	1	0.997963771	0	1
11748	GO:0051653	1	0.990050582	0	5
11749	GO:0051654	1	0.993989159	0	3

11750	GO:0051656	1	0.995997675	0	2
11751	GO:0051659	1	0.995988819	0	2
11752	GO:0051660	1	0.980179111	0	10
11753	GO:0051661	1	0.988076863	0	6
11754	GO:0051664	1	0.997990564	0	1
11755	GO:0051665	1	0.99799116	0	1
11756	GO:0051666	1	0.997976058	0	1
11757	GO:0051668	1	0.993969002	0	3
11758	GO:0051673	1	0.995958347	0	2
11759	GO:0051683	1	0.98400654	0	8
11760	GO:0051684	1	0.994030917	0	3
11761	GO:0051685	1	0.997990564	0	1
11762	GO:0051693	1	0.966483078	0	17
11763	GO:0051694	1	0.988033923	0	6
11764	GO:0051697	1	0.997992647	0	1
11765	GO:0051701	1	0.995944309	0	2
11766	GO:0051702	1	0.989992592	0	5
11767	GO:0051707	1	0.9899979	0	5
11768	GO:0051709	1	0.997996551	0	1
11769	GO:0051712	1	0.99401956	0	3
11770	GO:0051717	1	0.998013415	0	1
11772	GO:0051722	1	0.997986015	0	1
11773	GO:0051723	1	0.997986015	0	1
11774	GO:0051724	1	0.993975906	0	3
11775	GO:0051725	1	0.989983146	0	5
11777	GO:0051729	1	0.994009949	0	3
11778	GO:0051731	1	0.995991665	0	2
11779	GO:0051733	1	0.997974295	0	1
11780	GO:0051734	1	0.995952251	0	2
11781	GO:0051736	1	0.997974295	0	1
11782	GO:0051747	1	0.997959145	0	1
11783	GO:0051750	1	0.997965466	0	1
11784	GO:0051751	1	0.998010119	0	1
11785	GO:0051754	1	0.994009217	0	3
11786	GO:0051758	1	0.998013437	0	1
11787	GO:0051764	1	0.974247754	0	13
11788	GO:0051765	1	0.997975112	0	1
11790	GO:0051771	1	0.991944332	0	4
11791	GO:0051775	1	0.978102353	0	11
11792	GO:0051780	1	0.99800882	0	1
11793	GO:0051781	1	0.918921485	0	42
11794	GO:0051782	1	0.984055104	0	8
11795	GO:0051786	1	0.997999107	0	1
11796	GO:0051787	1	0.945116378	0	28
11797	GO:0051788	1	0.993992023	0	3

11798	GO:0051790	1	0.997965658	0	1
11799	GO:0051791	1	0.989984513	0	5
11800	GO:0051792	1	0.987932365	0	6
11801	GO:0051793	1	0.989957697	0	5
11802	GO:0051794	1	0.998013192	0	1
11803	GO:0051795	1	0.998013192	0	1
11804	GO:0051796	1	0.998009912	0	1
11805	GO:0051797	1	0.995991978	0	2
11806	GO:0051798	1	0.985939786	0	7
11807	GO:0051799	1	0.993998316	0	3
11808	GO:0051800	1	0.998013415	0	1
11809	GO:0051823	1	0.992041448	0	4
11810	GO:0051835	1	0.992001426	0	4
11811	GO:0051838	1	0.987892673	0	6
11812	GO:0051861	1	0.991954199	0	4
11813	GO:0051864	1	0.98021105	0	10
11814	GO:0051865	1	0.870430665	0	69
11815	GO:0051866	1	0.998002118	0	1
11816	GO:0051870	1	0.995971903	0	2
11817	GO:0051873	1	0.995924951	0	2
11818	GO:0051875	1	0.99600866	0	2
11819	GO:0051877	1	0.995988995	0	2
11820	GO:0051878	1	0.99800189	0	1
11822	GO:0051880	1	0.980176975	0	10
11823	GO:0051881	1	0.94492835	0	28
11824	GO:0051882	1	0.99397753	0	3
11825	GO:0051884	1	0.996008433	0	2
11826	GO:0051885	1	0.996000349	0	2
11827	GO:0051886	1	0.998013146	0	1
11828	GO:0051891	1	0.994029603	0	3
11829	GO:0051893	1	0.953099998	0	24
11830	GO:0051894	1	0.94928523	0	26
11831	GO:0051895	1	0.964614602	0	18
11832	GO:0051896	1	0.972255622	0	14
11833	GO:0051897	1	0.725845701	0	159
11834	GO:0051898	1	0.915382649	0	44
11835	GO:0051899	1	0.960649418	0	20
11836	GO:0051900	1	0.990032448	0	5
11837	GO:0051901	1	0.983975882	0	8
11839	GO:0051903	1	0.995971143	0	2
11840	GO:0051904	1	0.99600866	0	2
11841	GO:0051908	1	0.997997419	0	1
11842	GO:0051915	1	0.998013437	0	1
11843	GO:0051916	1	0.995982913	0	2
11844	GO:0051917	1	0.995959536	0	2

11845	GO:0051918	1	0.98201805	0	9
11846	GO:0051919	1	0.993958894	0	3
11847	GO:0051920	1	0.985858639	0	7
11848	GO:0051923	1	0.972079455	0	14
11849	GO:0051924	1	0.954890539	0	23
11850	GO:0051926	1	0.98015446	0	10
11851	GO:0051928	1	0.952871329	0	24
11852	GO:0051930	1	0.958650281	0	21
11853	GO:0051932	1	0.978176606	0	11
11854	GO:0051935	1	0.998013437	0	1
11855	GO:0051936	1	0.99199354	0	4
11856	GO:0051938	1	0.996012226	0	2
11857	GO:0051939	1	0.992008609	0	4
11858	GO:0051946	1	0.996030686	0	2
11859	GO:0051951	1	0.998005048	0	1
11860	GO:0051956	1	0.995985193	0	2
11861	GO:0051957	1	0.995996458	0	2
11862	GO:0051958	1	0.996009224	0	2
11863	GO:0051959	1	0.947454845	0	27
11864	GO:0051960	1	0.997985733	0	1
11865	GO:0051961	1	0.997990564	0	1
11866	GO:0051963	1	0.982200269	0	9
11867	GO:0051964	1	0.990092624	0	5
11868	GO:0051965	1	0.915670362	0	44
11869	GO:0051966	1	0.949259331	0	26
11870	GO:0051967	1	0.990023802	0	5
11871	GO:0051968	1	0.962665947	0	19
11872	GO:0051969	1	0.993927192	0	3
11873	GO:0051970	1	0.99598826	0	2
11874	GO:0051971	1	0.998013437	0	1
11875	GO:0051972	1	0.992009109	0	4
11876	GO:0051973	1	0.93387658	0	34
11877	GO:0051974	1	0.974184982	0	13
11878	GO:0051977	1	0.995945888	0	2
11879	GO:0051978	1	0.997981704	0	1
11880	GO:0051983	1	0.982095531	0	9
11881	GO:0051984	1	0.990087476	0	5
11882	GO:0051987	1	0.993977504	0	3
11883	GO:0051988	1	0.986060123	0	7
11884	GO:0051990	1	0.997988044	0	1
11885	GO:0051996	1	0.997980382	0	1
11886	GO:0051998	1	0.997990564	0	1
11887	GO:0052031	1	0.985950064	0	7
11888	GO:0052314	1	0.997978943	0	1
11889	GO:0052362	1	0.997972358	0	1

11890	GO:0052373	1	0.997953748	0	1
11891	GO:0052381	1	0.997980773	0	1
11892	GO:0052405	1	0.993912082	0	3
11893	GO:0052547	1	0.981906398	0	9
11894	GO:0052548	1	0.984049826	0	8
11895	GO:0052590	1	0.998013437	0	1
11896	GO:0052591	1	0.998013437	0	1
11897	GO:0052593	1	0.996000206	0	2
11898	GO:0052594	1	0.996000206	0	2
11899	GO:0052595	1	0.996000206	0	2
11900	GO:0052596	1	0.996000206	0	2
11901	GO:0052597	1	0.997990564	0	1
11902	GO:0052598	1	0.997990564	0	1
11903	GO:0052599	1	0.997990564	0	1
11904	GO:0052600	1	0.997990564	0	1
11905	GO:0052629	1	0.986114284	0	7
11906	GO:0052630	1	0.997983966	0	1
11907	GO:0052642	1	0.993959931	0	3
11908	GO:0052650	1	0.979961341	0	10
11909	GO:0052651	1	0.987971258	0	6
11910	GO:0052652	1	0.998004812	0	1
11911	GO:0052654	1	0.995989427	0	2
11912	GO:0052655	1	0.995989427	0	2
11913	GO:0052656	1	0.995989427	0	2
11914	GO:0052657	1	0.997968322	0	1
11915	GO:0052658	1	0.986105529	0	7
11916	GO:0052659	1	0.990038523	0	5
11917	GO:0052666	1	0.997977919	0	1
11918	GO:0052689	1	0.92613559	0	38
11919	GO:0052692	1	0.997968378	0	1
11920	GO:0052695	1	0.96820517	0	16
11921	GO:0052696	1	0.981998224	0	9
11922	GO:0052697	1	0.98000744	0	10
11923	GO:0052717	1	0.998013437	0	1
11924	GO:0052722	1	0.997977163	0	1
11925	GO:0052723	1	0.992050608	0	4
11926	GO:0052724	1	0.992050608	0	4
11927	GO:0052725	1	0.997998232	0	1
11928	GO:0052726	1	0.997998232	0	1
11929	GO:0052735	1	0.991992288	0	4
11930	GO:0052739	1	0.982044889	0	9
11931	GO:0052740	1	0.982044889	0	9
11932	GO:0052741	1	0.995949749	0	2
11933	GO:0052742	1	0.980251959	0	10
11934	GO:0052745	1	0.99402785	0	3

11935	GO:0052746	1	0.996012806	0	2
11936	GO:0052751	1	0.997960636	0	1
11937	GO:0052794	1	0.993966343	0	3
11938	GO:0052795	1	0.993966343	0	3
11939	GO:0052796	1	0.993966343	0	3
11940	GO:0052798	1	0.997980218	0	1
11941	GO:0052810	1	0.998013437	0	1
11942	GO:0052812	1	0.988075956	0	6
11943	GO:0052814	1	0.998005077	0	1
11944	GO:0052815	1	0.995968025	0	2
11945	GO:0052816	1	0.995991425	0	2
11946	GO:0052817	1	0.998000663	0	1
11947	GO:0052821	1	0.997961976	0	1
11948	GO:0052822	1	0.997961976	0	1
11949	GO:0052824	1	0.997984678	0	1
11950	GO:0052826	1	0.997992012	0	1
11951	GO:0052828	1	0.996025715	0	2
11952	GO:0052832	1	0.993978668	0	3
11953	GO:0052833	1	0.993978668	0	3
11954	GO:0052834	1	0.993978668	0	3
11955	GO:0052840	1	0.98997282	0	5
11956	GO:0052842	1	0.98997282	0	5
11957	GO:0052851	1	0.994042865	0	3
11958	GO:0052855	1	0.997990564	0	1
11959	GO:0052856	1	0.995954532	0	2
11960	GO:0052857	1	0.995954532	0	2
11961	GO:0052858	1	0.998002699	0	1
11962	GO:0052866	1	0.992052327	0	4
11963	GO:0052869	1	0.996000692	0	2
11964	GO:0052871	1	0.997984552	0	1
11965	GO:0052872	1	0.997984552	0	1
11966	GO:0052884	1	0.997988448	0	1
11967	GO:0052885	1	0.997988448	0	1
11968	GO:0052894	1	0.997979817	0	1
11969	GO:0052895	1	0.997979817	0	1
11970	GO:0052899	1	0.997973416	0	1
11971	GO:0052901	1	0.995957234	0	2
11972	GO:0052902	1	0.997973416	0	1
11973	GO:0052903	1	0.997973416	0	1
11974	GO:0052904	1	0.997973416	0	1
11975	GO:0052905	1	0.993983524	0	3
11976	GO:0052906	1	0.998013274	0	1
11977	GO:0052907	1	0.997990564	0	1
11978	GO:0052909	1	0.997990564	0	1
11979	GO:0052917	1	0.997984678	0	1

11980	GO:0052918	1	0.998013437	0	1
11981	GO:0052925	1	0.997970201	0	1
11982	GO:0052926	1	0.998013437	0	1
11983	GO:0052927	1	0.997983092	0	1
11984	GO:0052928	1	0.997983092	0	1
11985	GO:0052929	1	0.997983092	0	1
11986	GO:0055001	1	0.994034053	0	3
11987	GO:0055002	1	0.997998107	0	1
11988	GO:0055003	1	0.980148288	0	10
11989	GO:0055005	1	0.996012712	0	2
11990	GO:0055006	1	0.998013437	0	1
11991	GO:0055007	1	0.966416123	0	17
11992	GO:0055008	1	0.98605486	0	7
11993	GO:0055009	1	0.992005573	0	4
11994	GO:0055010	1	0.958503651	0	21
11995	GO:0055012	1	0.992013875	0	4
11996	GO:0055013	1	0.988134791	0	6
11997	GO:0055014	1	0.997973451	0	1
11998	GO:0055015	1	0.987971079	0	6
11999	GO:0055017	1	0.998013437	0	1
12000	GO:0055020	1	0.997974922	0	1
12001	GO:0055022	1	0.997941377	0	1
12002	GO:0055024	1	0.998002947	0	1
12003	GO:0055025	1	0.998013437	0	1
12004	GO:0055026	1	0.99796116	0	1
12005	GO:0055028	1	0.998013437	0	1
12006	GO:0055037	1	0.771406707	0	129
12007	GO:0055038	1	0.84627837	0	83
12008	GO:0055048	1	0.998013437	0	1
12009	GO:0055056	1	0.990045097	0	5
12010	GO:0055059	1	0.994025412	0	3
12011	GO:0055062	1	0.994002882	0	3
12012	GO:0055064	1	0.978289232	0	11
12013	GO:0055065	1	0.993990655	0	3
12014	GO:0055069	1	0.990009872	0	5
12015	GO:0055070	1	0.991919777	0	4
12016	GO:0055071	1	0.998012027	0	1
12017	GO:0055072	1	0.935794716	0	33
12018	GO:0055073	1	0.997951758	0	1
12019	GO:0055074	1	0.952922631	0	24
12020	GO:0055075	1	0.968501751	0	16
12021	GO:0055077	1	0.994018045	0	3
12022	GO:0055078	1	0.972317297	0	14
12023	GO:0055080	1	0.998013437	0	1
12024	GO:0055081	1	0.998013437	0	1

12025	GO:0055082	1	0.998012418	0	1
12027	GO:0055087	1	0.993998266	0	3
12028	GO:0055088	1	0.913353546	0	45
12029	GO:0055089	1	0.974255366	0	13
12030	GO:0055091	1	0.970247178	0	15
12031	GO:0055092	1	0.989991269	0	5
12032	GO:0055093	1	0.972217883	0	14
12033	GO:0055096	1	0.995989555	0	2
12034	GO:0055100	1	0.994005591	0	3
12035	GO:0055102	1	0.99189758	0	4
12036	GO:0055103	1	0.99799807	0	1
12037	GO:0055105	1	0.995946717	0	2
12038	GO:0055106	1	0.994004197	0	3
12039	GO:0055107	1	0.995985075	0	2
12040	GO:0055108	1	0.997978237	0	1
12041	GO:0055111	1	0.998013431	0	1
12042	GO:0055113	1	0.998013437	0	1
12044	GO:0055117	1	0.964513917	0	18
12045	GO:0055118	1	0.993993775	0	3
12046	GO:0055119	1	0.976209301	0	12
12047	GO:0055120	1	0.997961189	0	1
12048	GO:0055123	1	0.994024836	0	3
12049	GO:0055129	1	0.987970226	0	6
12050	GO:0055131	1	0.987967937	0	6
12051	GO:0060001	1	0.998013437	0	1
12052	GO:0060002	1	0.994044125	0	3
12053	GO:0060003	1	0.993988544	0	3
12054	GO:0060005	1	0.994015684	0	3
12055	GO:0060007	1	0.998009113	0	1
12056	GO:0060008	1	0.990008736	0	5
12057	GO:0060009	1	0.986080505	0	7
12058	GO:0060010	1	0.997999158	0	1
12059	GO:0060011	1	0.99398405	0	3
12060	GO:0060012	1	0.991996471	0	4
12061	GO:0060013	1	0.986079548	0	7
12062	GO:0060016	1	0.997965313	0	1
12063	GO:0060017	1	0.988041427	0	6
12064	GO:0060018	1	0.992028875	0	4
12065	GO:0060019	1	0.99199184	0	4
12066	GO:0060020	1	0.982087508	0	9
12067	GO:0060021	1	0.888206773	0	59
12068	GO:0060022	1	0.988051697	0	6
12069	GO:0060023	1	0.992021171	0	4
12070	GO:0060024	1	0.9960241	0	2
12071	GO:0060025	1	0.994027078	0	3

12072	GO:0060026	1	0.99404709	0	3
12073	GO:0060027	1	0.996005429	0	2
12074	GO:0060028	1	0.991986775	0	4
12075	GO:0060029	1	0.99200429	0	4
12076	GO:0060032	1	0.996018773	0	2
12077	GO:0060033	1	0.994014029	0	3
12078	GO:0060035	1	0.99800715	0	1
12079	GO:0060037	1	0.970378843	0	15
12080	GO:0060038	1	0.978220055	0	11
12081	GO:0060039	1	0.986120984	0	7
12082	GO:0060040	1	0.993998455	0	3
12083	GO:0060041	1	0.879410615	0	64
12084	GO:0060042	1	0.978204958	0	11
12085	GO:0060043	1	0.99203848	0	4
12086	GO:0060044	1	0.974204569	0	13
12087	GO:0060045	1	0.960757079	0	20
12088	GO:0060046	1	0.99596216	0	2
12089	GO:0060047	1	0.974167077	0	13
12090	GO:0060048	1	0.928222952	0	37
12091	GO:0060049	1	0.993990435	0	3
12092	GO:0060050	1	0.990002122	0	5
12093	GO:0060051	1	0.997990564	0	1
12094	GO:0060052	1	0.98409265	0	8
12095	GO:0060053	1	0.996002201	0	2
12096	GO:0060054	1	0.986005392	0	7
12097	GO:0060055	1	0.980144802	0	10
12098	GO:0060056	1	0.99201811	0	4
12099	GO:0060058	1	0.99397375	0	3
12100	GO:0060059	1	0.988090556	0	6
12101	GO:0060060	1	0.998012752	0	1
12102	GO:0060061	1	0.995994645	0	2
12103	GO:0060064	1	0.997969788	0	1
12104	GO:0060065	1	0.980186867	0	10
12105	GO:0060066	1	0.993969458	0	3
12106	GO:0060067	1	0.996000556	0	2
12107	GO:0060068	1	0.982124997	0	9
12108	GO:0060070	1	0.879286551	0	64
12109	GO:0060071	1	0.835380494	0	89
12110	GO:0060072	1	0.998013437	0	1
12111	GO:0060073	1	0.995998947	0	2
12112	GO:0060074	1	0.982114415	0	9
12113	GO:0060075	1	0.984088783	0	8
12114	GO:0060076	1	0.951317281	0	25
12115	GO:0060077	1	0.978118081	0	11
12116	GO:0060078	1	0.922782712	0	40

12117	GO:0060079	1	0.897139193	0	54
12118	GO:0060080	1	0.984053427	0	8
12119	GO:0060081	1	0.984028691	0	8
12120	GO:0060083	1	0.99602609	0	2
12121	GO:0060084	1	0.994000352	0	3
12122	GO:0060086	1	0.993995328	0	3
12123	GO:0060087	1	0.987987376	0	6
12124	GO:0060088	1	0.970312734	0	15
12125	GO:0060090	1	0.849809051	0	81
12126	GO:0060091	1	0.988002306	0	6
12127	GO:0060100	1	0.978146306	0	11
12128	GO:0060112	1	0.997954463	0	1
12129	GO:0060113	1	0.988017519	0	6
12130	GO:0060116	1	0.998013437	0	1
12131	GO:0060117	1	0.992027004	0	4
12132	GO:0060119	1	0.990000521	0	5
12133	GO:0060121	1	0.998004633	0	1
12134	GO:0060122	1	0.95475801	0	23
12135	GO:0060123	1	0.998013437	0	1
12136	GO:0060124	1	0.985995189	0	7
12137	GO:0060125	1	0.996030731	0	2
12138	GO:0060126	1	0.995992952	0	2
12139	GO:0060127	1	0.997982436	0	1
12140	GO:0060128	1	0.993983055	0	3
12141	GO:0060129	1	0.992001363	0	4
12142	GO:0060134	1	0.984139247	0	8
12143	GO:0060135	1	0.962518675	0	19
12144	GO:0060136	1	0.989994697	0	5
12145	GO:0060137	1	0.99201581	0	4
12146	GO:0060139	1	0.995978809	0	2
12147	GO:0060143	1	0.993985611	0	3
12148	GO:0060152	1	0.996025738	0	2
12149	GO:0060155	1	0.982013897	0	9
12150	GO:0060157	1	0.995974955	0	2
12151	GO:0060158	1	0.987950862	0	6
12152	GO:0060159	1	0.991976624	0	4
12153	GO:0060160	1	0.99398992	0	3
12154	GO:0060161	1	0.994021378	0	3
12155	GO:0060163	1	0.997986219	0	1
12156	GO:0060164	1	0.995971253	0	2
12157	GO:0060165	1	0.997986219	0	1
12158	GO:0060166	1	0.99396819	0	3
12159	GO:0060168	1	0.993987715	0	3
12160	GO:0060169	1	0.997970558	0	1
12161	GO:0060170	1	0.904348552	0	50

12162	GO:0060171	1	0.990057211	0	5
12163	GO:0060173	1	0.917321122	0	43
12164	GO:0060174	1	0.982142029	0	9
12165	GO:0060175	1	0.998013437	0	1
12166	GO:0060177	1	0.995976298	0	2
12167	GO:0060178	1	0.997982804	0	1
12168	GO:0060179	1	0.990043978	0	5
12169	GO:0060183	1	0.995992197	0	2
12170	GO:0060187	1	0.99801197	0	1
12171	GO:0060192	1	0.993942681	0	3
12172	GO:0060193	1	0.993941609	0	3
12173	GO:0060197	1	0.993975958	0	3
12174	GO:0060201	1	0.995986253	0	2
12175	GO:0060203	1	0.991981228	0	4
12176	GO:0060205	1	0.991880492	0	4
12177	GO:0060212	1	0.993968596	0	3
12178	GO:0060213	1	0.974238642	0	13
12179	GO:0060214	1	0.993951705	0	3
12180	GO:0060215	1	0.994004635	0	3
12181	GO:0060216	1	0.976297427	0	12
12182	GO:0060217	1	0.998012887	0	1
12183	GO:0060218	1	0.972287956	0	14
12184	GO:0060219	1	0.992001897	0	4
12185	GO:0060220	1	0.997979743	0	1
12186	GO:0060221	1	0.993984396	0	3
12187	GO:0060228	1	0.993883505	0	3
12188	GO:0060230	1	0.99592295	0	2
12189	GO:0060231	1	0.990015231	0	5
12190	GO:0060232	1	0.998013437	0	1
12191	GO:0060234	1	0.998013437	0	1
12192	GO:0060235	1	0.994013509	0	3
12193	GO:0060236	1	0.96647463	0	17
12194	GO:0060242	1	0.992010394	0	4
12195	GO:0060243	1	0.998009559	0	1
12196	GO:0060244	1	0.992003874	0	4
12197	GO:0060251	1	0.996008915	0	2
12198	GO:0060252	1	0.966362921	0	17
12199	GO:0060253	1	0.987955018	0	6
12200	GO:0060259	1	0.990044938	0	5
12201	GO:0060260	1	0.897003568	0	54
12202	GO:0060261	1	0.966539741	0	17
12203	GO:0060262	1	0.997975457	0	1
12204	GO:0060263	1	0.991913579	0	4
12205	GO:0060265	1	0.99795724	0	1
12206	GO:0060266	1	0.99393549	0	3

12207	GO:0060267	1	0.993989026	0	3
12208	GO:0060271	1	0.640239645	0	221
12209	GO:0060272	1	0.984010358	0	8
12210	GO:0060279	1	0.99598276	0	2
12211	GO:0060282	1	0.998008767	0	1
12212	GO:0060283	1	0.998013437	0	1
12213	GO:0060285	1	0.968498099	0	16
12214	GO:0060287	1	0.98013675	0	10
12215	GO:0060290	1	0.991999361	0	4
12216	GO:0060291	1	0.919299881	0	42
12217	GO:0060292	1	0.964561116	0	18
12218	GO:0060294	1	0.976021185	0	12
12219	GO:0060296	1	0.988001192	0	6
12220	GO:0060297	1	0.998013437	0	1
12221	GO:0060298	1	0.99400084	0	3
12222	GO:0060300	1	0.998009113	0	1
12223	GO:0060301	1	0.997986391	0	1
12224	GO:0060302	1	0.995952938	0	2
12225	GO:0060304	1	0.988095941	0	6
12226	GO:0060305	1	0.997992136	0	1
12227	GO:0060306	1	0.980162869	0	10
12228	GO:0060307	1	0.964560474	0	18
12229	GO:0060309	1	0.995971125	0	2
12230	GO:0060311	1	0.997955724	0	1
12231	GO:0060312	1	0.9960184	0	2
12232	GO:0060313	1	0.997955724	0	1
12233	GO:0060314	1	0.964489327	0	18
12234	GO:0060315	1	0.9799904	0	10
12235	GO:0060316	1	0.984011614	0	8
12236	GO:0060317	1	0.974298608	0	13
12237	GO:0060318	1	0.994000064	0	3
12238	GO:0060319	1	0.996003943	0	2
12239	GO:0060322	1	0.964473	0	18
12240	GO:0060323	1	0.985978394	0	7
12241	GO:0060324	1	0.968472325	0	16
12242	GO:0060325	1	0.943563898	0	29
12243	GO:0060326	1	0.868192736	0	70
12244	GO:0060327	1	0.994006268	0	3
12245	GO:0060330	1	0.997998019	0	1
12246	GO:0060332	1	0.997972187	0	1
12249	GO:0060335	1	0.988021756	0	6
12250	GO:0060336	1	0.987962406	0	6
12252	GO:0060338	1	0.982062756	0	9
12255	GO:0060341	1	0.986103961	0	7
12256	GO:0060342	1	0.993995921	0	3

12257	GO:0060346	1	0.98611373	0	7
12258	GO:0060347	1	0.980097153	0	10
12259	GO:0060348	1	0.888157331	0	59
12260	GO:0060349	1	0.947204763	0	27
12261	GO:0060350	1	0.990076754	0	5
12262	GO:0060351	1	0.988088283	0	6
12263	GO:0060352	1	0.993997623	0	3
12264	GO:0060354	1	0.989956956	0	5
12265	GO:0060355	1	0.991955294	0	4
12266	GO:0060358	1	0.995985193	0	2
12267	GO:0060359	1	0.997990564	0	1
12268	GO:0060363	1	0.991958541	0	4
12269	GO:0060364	1	0.994004256	0	3
12270	GO:0060366	1	0.998013437	0	1
12271	GO:0060367	1	0.998013437	0	1
12272	GO:0060368	1	0.995972507	0	2
12273	GO:0060369	1	0.99598806	0	2
12274	GO:0060370	1	0.996007902	0	2
12275	GO:0060371	1	0.990041161	0	5
12276	GO:0060372	1	0.99203728	0	4
12277	GO:0060373	1	0.990064911	0	5
12278	GO:0060374	1	0.994033591	0	3
12279	GO:0060375	1	0.998012887	0	1
12280	GO:0060376	1	0.997987811	0	1
12281	GO:0060377	1	0.99799807	0	1
12282	GO:0060378	1	0.997974974	0	1
12283	GO:0060379	1	0.99399027	0	3
12284	GO:0060381	1	0.997979644	0	1
12285	GO:0060382	1	0.998013437	0	1
12286	GO:0060383	1	0.998008429	0	1
12287	GO:0060384	1	0.968517986	0	16
12288	GO:0060385	1	0.992049908	0	4
12289	GO:0060386	1	0.998013436	0	1
12290	GO:0060389	1	0.980185003	0	10
12291	GO:0060390	1	0.989996903	0	5
12292	GO:0060391	1	0.972346145	0	14
12293	GO:0060392	1	0.988007771	0	6
12294	GO:0060393	1	0.991967171	0	4
12295	GO:0060394	1	0.974186786	0	13
12296	GO:0060395	1	0.904474468	0	50
12297	GO:0060396	1	0.986129509	0	7
12298	GO:0060397	1	0.980261982	0	10
12299	GO:0060398	1	0.99796964	0	1
12300	GO:0060399	1	0.993993029	0	3
12301	GO:0060400	1	0.995979915	0	2

12302	GO:0060401	1	0.996007903	0	2
12303	GO:0060402	1	0.974389802	0	13
12304	GO:0060404	1	0.998003737	0	1
12305	GO:0060405	1	0.998013437	0	1
12306	GO:0060406	1	0.994027855	0	3
12307	GO:0060407	1	0.997970558	0	1
12308	GO:0060411	1	0.976228276	0	12
12309	GO:0060412	1	0.939785674	0	31
12310	GO:0060413	1	0.984121554	0	8
12311	GO:0060414	1	0.993999371	0	3
12312	GO:0060415	1	0.993991762	0	3
12313	GO:0060416	1	0.976174787	0	12
12314	GO:0060420	1	0.99399805	0	3
12315	GO:0060421	1	0.993995624	0	3
12316	GO:0060422	1	0.997983394	0	1
12317	GO:0060425	1	0.968346118	0	16
12318	GO:0060426	1	0.994006808	0	3
12319	GO:0060428	1	0.9860482	0	7
12320	GO:0060429	1	0.986014796	0	7
12321	GO:0060430	1	0.990050861	0	5
12322	GO:0060431	1	0.998002091	0	1
12323	GO:0060433	1	0.995990777	0	2
12324	GO:0060434	1	0.996003392	0	2
12325	GO:0060435	1	0.993955585	0	3
12326	GO:0060437	1	0.994002167	0	3
12327	GO:0060438	1	0.99397145	0	3
12328	GO:0060439	1	0.993995587	0	3
12329	GO:0060440	1	0.988015406	0	6
12330	GO:0060441	1	0.972333221	0	14
12331	GO:0060442	1	0.989980095	0	5
12332	GO:0060443	1	0.996023277	0	2
12333	GO:0060444	1	0.980239455	0	10
12334	GO:0060445	1	0.980171756	0	10
12335	GO:0060447	1	0.995988589	0	2
12336	GO:0060448	1	0.997969391	0	1
12337	GO:0060449	1	0.98802318	0	6
12338	GO:0060452	1	0.985952801	0	7
12339	GO:0060453	1	0.994006781	0	3
12340	GO:0060454	1	0.996011237	0	2
12341	GO:0060455	1	0.991921956	0	4
12342	GO:0060458	1	0.997971213	0	1
12343	GO:0060459	1	0.997971213	0	1
12344	GO:0060463	1	0.988081514	0	6
12345	GO:0060465	1	0.994025946	0	3
12346	GO:0060466	1	0.998013437	0	1

12347	GO:0060468	1	0.99596617	0	2
12348	GO:0060471	1	0.998013437	0	1
12349	GO:0060473	1	0.991957589	0	4
12350	GO:0060474	1	0.995952813	0	2
12351	GO:0060478	1	0.994008328	0	3
12352	GO:0060479	1	0.99800067	0	1
12353	GO:0060480	1	0.995940837	0	2
12354	GO:0060481	1	0.998006659	0	1
12355	GO:0060482	1	0.998006669	0	1
12356	GO:0060484	1	0.9880168	0	6
12357	GO:0060485	1	0.992041125	0	4
12358	GO:0060486	1	0.994011194	0	3
12359	GO:0060487	1	0.984094055	0	8
12360	GO:0060488	1	0.998013437	0	1
12361	GO:0060489	1	0.998013437	0	1
12362	GO:0060490	1	0.998013437	0	1
12363	GO:0060491	1	0.993980921	0	3
12364	GO:0060492	1	0.995987168	0	2
12365	GO:0060501	1	0.992010783	0	4
12366	GO:0060502	1	0.995966948	0	2
12367	GO:0060503	1	0.997974922	0	1
12368	GO:0060506	1	0.997999867	0	1
12369	GO:0060509	1	0.990037214	0	5
12370	GO:0060510	1	0.993991395	0	3
12371	GO:0060512	1	0.992002442	0	4
12372	GO:0060513	1	0.994013682	0	3
12373	GO:0060516	1	0.997971213	0	1
12374	GO:0060517	1	0.998006804	0	1
12375	GO:0060520	1	0.997995025	0	1
12376	GO:0060523	1	0.994004852	0	3
12377	GO:0060527	1	0.992069478	0	4
12378	GO:0060528	1	0.9940064	0	3
12379	GO:0060529	1	0.996024135	0	2
12380	GO:0060532	1	0.996020806	0	2
12381	GO:0060534	1	0.992031278	0	4
12382	GO:0060535	1	0.994028402	0	3
12383	GO:0060536	1	0.997973257	0	1
12384	GO:0060537	1	0.989911896	0	5
12385	GO:0060538	1	0.993983747	0	3
12386	GO:0060539	1	0.986018811	0	7
12387	GO:0060541	1	0.992017807	0	4
12388	GO:0060544	1	0.962535558	0	19
12389	GO:0060545	1	0.991979421	0	4
12390	GO:0060546	1	0.974255512	0	13
12391	GO:0060547	1	0.993884069	0	3

12392	GO:0060548	1	0.86844624	0	70
12393	GO:0060557	1	0.997972866	0	1
12394	GO:0060558	1	0.998012112	0	1
12395	GO:0060559	1	0.99594675	0	2
12396	GO:0060560	1	0.994018254	0	3
12397	GO:0060561	1	0.990027891	0	5
12398	GO:0060562	1	0.996030731	0	2
12399	GO:0060563	1	0.986014471	0	7
12400	GO:0060566	1	0.998013437	0	1
12401	GO:0060571	1	0.99597744	0	2
12402	GO:0060574	1	0.99397281	0	3
12403	GO:0060575	1	0.992020146	0	4
12404	GO:0060576	1	0.98407206	0	8
12405	GO:0060579	1	0.993997826	0	3
12406	GO:0060585	1	0.993968205	0	3
12407	GO:0060586	1	0.982051484	0	9
12408	GO:0060587	1	0.998000656	0	1
12409	GO:0060588	1	0.997962837	0	1
12410	GO:0060590	1	0.995994947	0	2
12411	GO:0060591	1	0.992011397	0	4
12412	GO:0060592	1	0.997974922	0	1
12413	GO:0060594	1	0.998013437	0	1
12414	GO:0060595	1	0.998008455	0	1
12415	GO:0060596	1	0.996016151	0	2
12416	GO:0060598	1	0.997964891	0	1
12417	GO:0060599	1	0.996012355	0	2
12418	GO:0060601	1	0.998008455	0	1
12419	GO:0060603	1	0.988132021	0	6
12420	GO:0060606	1	0.998013437	0	1
12421	GO:0060611	1	0.997985513	0	1
12422	GO:0060612	1	0.945375461	0	28
12423	GO:0060613	1	0.990019852	0	5
12424	GO:0060615	1	0.998008455	0	1
12425	GO:0060620	1	0.997990564	0	1
12426	GO:0060623	1	0.993985886	0	3
12427	GO:0060627	1	0.98209223	0	9
12428	GO:0060628	1	0.988036988	0	6
12429	GO:0060629	1	0.995971277	0	2
12430	GO:0060631	1	0.997995414	0	1
12431	GO:0060632	1	0.995985372	0	2
12432	GO:0060633	1	0.994008233	0	3
12433	GO:0060638	1	0.99599991	0	2
12434	GO:0060644	1	0.976217595	0	12
12435	GO:0060648	1	0.997976314	0	1
12436	GO:0060661	1	0.997981357	0	1

12437	GO:0060662	1	0.989990242	0	5
12438	GO:0060664	1	0.991974787	0	4
12439	GO:0060665	1	0.995989597	0	2
12440	GO:0060666	1	0.990078583	0	5
12441	GO:0060667	1	0.998008455	0	1
12442	GO:0060668	1	0.998003256	0	1
12443	GO:0060669	1	0.990059014	0	5
12444	GO:0060670	1	0.984037518	0	8
12445	GO:0060672	1	0.993997843	0	3
12446	GO:0060674	1	0.974313757	0	13
12447	GO:0060675	1	0.995950354	0	2
12448	GO:0060676	1	0.991958461	0	4
12449	GO:0060678	1	0.997982868	0	1
12450	GO:0060681	1	0.998005652	0	1
12451	GO:0060682	1	0.99799034	0	1
12452	GO:0060683	1	0.997990851	0	1
12453	GO:0060684	1	0.991948233	0	4
12454	GO:0060685	1	0.995970214	0	2
12455	GO:0060686	1	0.992033032	0	4
12456	GO:0060687	1	0.99203061	0	4
12457	GO:0060688	1	0.993949132	0	3
12458	GO:0060689	1	0.996014543	0	2
12459	GO:0060693	1	0.995990206	0	2
12460	GO:0060694	1	0.997976203	0	1
12461	GO:0060696	1	0.997983998	0	1
12462	GO:0060697	1	0.997954626	0	1
12463	GO:0060698	1	0.996008722	0	2
12464	GO:0060699	1	0.997967366	0	1
12466	GO:0060702	1	0.996008722	0	2
12467	GO:0060703	1	0.997989952	0	1
12468	GO:0060706	1	0.989979921	0	5
12469	GO:0060707	1	0.97619004	0	12
12470	GO:0060708	1	0.989991481	0	5
12471	GO:0060709	1	0.997992291	0	1
12472	GO:0060710	1	0.988039124	0	6
12473	GO:0060711	1	0.99005443	0	5
12474	GO:0060712	1	0.988035708	0	6
12475	GO:0060713	1	0.995983102	0	2
12476	GO:0060715	1	0.993994847	0	3
12477	GO:0060716	1	0.966384236	0	17
12478	GO:0060717	1	0.995987481	0	2
12479	GO:0060718	1	0.990064234	0	5
12480	GO:0060720	1	0.998013116	0	1
12481	GO:0060721	1	0.997958626	0	1
12482	GO:0060729	1	0.992006921	0	4

12483	GO:0060731	1	0.997998598	0	1
12484	GO:0060732	1	0.993980395	0	3
12485	GO:0060734	1	0.998011889	0	1
12486	GO:0060735	1	0.997967366	0	1
12487	GO:0060736	1	0.988095087	0	6
12488	GO:0060738	1	0.995971163	0	2
12489	GO:0060739	1	0.998013437	0	1
12490	GO:0060740	1	0.988053222	0	6
12491	GO:0060741	1	0.995949645	0	2
12492	GO:0060742	1	0.992022921	0	4
12493	GO:0060743	1	0.995987402	0	2
12494	GO:0060744	1	0.989981734	0	5
12495	GO:0060745	1	0.992075543	0	4
12496	GO:0060746	1	0.998013437	0	1
12497	GO:0060748	1	0.994026709	0	3
12498	GO:0060749	1	0.968378231	0	16
12499	GO:0060750	1	0.992028596	0	4
12500	GO:0060751	1	0.997981889	0	1
12501	GO:0060753	1	0.997969271	0	1
12502	GO:0060754	1	0.987980878	0	6
12503	GO:0060755	1	0.998006014	0	1
12505	GO:0060761	1	0.987991858	0	6
12506	GO:0060762	1	0.993975799	0	3
12507	GO:0060763	1	0.993994893	0	3
12508	GO:0060764	1	0.998013122	0	1
12509	GO:0060765	1	0.915334722	0	44
12510	GO:0060766	1	0.974311745	0	13
12511	GO:0060768	1	0.995988589	0	2
12512	GO:0060769	1	0.993978758	0	3
12513	GO:0060770	1	0.987958435	0	6
12514	GO:0060775	1	0.995994729	0	2
12515	GO:0060782	1	0.997971213	0	1
12516	GO:0060783	1	0.997971213	0	1
12517	GO:0060784	1	0.998006804	0	1
12518	GO:0060789	1	0.98997456	0	5
12519	GO:0060795	1	0.997968004	0	1
12520	GO:0060800	1	0.994007649	0	3
12521	GO:0060803	1	0.997969659	0	1
12522	GO:0060804	1	0.997996626	0	1
12523	GO:0060806	1	0.997973257	0	1
12524	GO:0060809	1	0.996007903	0	2
12525	GO:0060816	1	0.996017131	0	2
12526	GO:0060817	1	0.997982002	0	1
12527	GO:0060819	1	0.996030727	0	2
12528	GO:0060820	1	0.998013437	0	1

12529	GO:0060821	1	0.998013437	0	1
12530	GO:0060825	1	0.997976723	0	1
12532	GO:0060829	1	0.998008236	0	1
12533	GO:0060830	1	0.995974458	0	2
12534	GO:0060831	1	0.988088062	0	6
12535	GO:0060836	1	0.98806996	0	6
12536	GO:0060837	1	0.997975801	0	1
12537	GO:0060838	1	0.9940039	0	3
12538	GO:0060840	1	0.984141637	0	8
12539	GO:0060841	1	0.994047721	0	3
12540	GO:0060842	1	0.993997939	0	3
12541	GO:0060843	1	0.998013437	0	1
12542	GO:0060844	1	0.997984402	0	1
12543	GO:0060853	1	0.997999078	0	1
12544	GO:0060854	1	0.998006543	0	1
12545	GO:0060856	1	0.995967927	0	2
12546	GO:0060857	1	0.99600046	0	2
12547	GO:0060873	1	0.998013437	0	1
12548	GO:0060875	1	0.998013437	0	1
12549	GO:0060887	1	0.997990564	0	1
12550	GO:0060896	1	0.998003307	0	1
12551	GO:0060900	1	0.997992028	0	1
12552	GO:0060901	1	0.998009912	0	1
12553	GO:0060903	1	0.99599197	0	2
12554	GO:0060904	1	0.99800725	0	1
12555	GO:0060907	1	0.97613679	0	12
12556	GO:0060912	1	0.997990564	0	1
12557	GO:0060913	1	0.993933319	0	3
12558	GO:0060914	1	0.995996222	0	2
12559	GO:0060915	1	0.998008455	0	1
12560	GO:0060916	1	0.993992135	0	3
12561	GO:0060920	1	0.997996656	0	1
12562	GO:0060921	1	0.997963363	0	1
12563	GO:0060923	1	0.994011044	0	3
12564	GO:0060928	1	0.996020619	0	2
12565	GO:0060930	1	0.998008236	0	1
12566	GO:0060931	1	0.990059307	0	5
12567	GO:0060932	1	0.997983585	0	1
12568	GO:0060939	1	0.998013437	0	1
12569	GO:0060940	1	0.995992447	0	2
12570	GO:0060945	1	0.997993844	0	1
12571	GO:0060947	1	0.993986939	0	3
12572	GO:0060948	1	0.994016389	0	3
12573	GO:0060956	1	0.992008613	0	4
12574	GO:0060964	1	0.851233876	0	80

12575	GO:0060965	1	0.991953187	0	4
12576	GO:0060968	1	0.978104153	0	11
12577	GO:0060971	1	0.991985114	0	4
12578	GO:0060972	1	0.985977866	0	7
12579	GO:0060973	1	0.996017622	0	2
12580	GO:0060975	1	0.997963363	0	1
12581	GO:0060976	1	0.951113057	0	25
12582	GO:0060977	1	0.996006627	0	2
12583	GO:0060978	1	0.994024933	0	3
12584	GO:0060979	1	0.994051875	0	3
12585	GO:0060981	1	0.998013436	0	1
12586	GO:0060982	1	0.984133049	0	8
12587	GO:0060987	1	0.997983808	0	1
12588	GO:0060988	1	0.993995174	0	3
12589	GO:0060989	1	0.998002277	0	1
12590	GO:0060992	1	0.986089644	0	7
12591	GO:0060993	1	0.980195865	0	10
12592	GO:0060994	1	0.998004343	0	1
12593	GO:0060996	1	0.972432772	0	14
12594	GO:0060997	1	0.966556864	0	17
12595	GO:0060998	1	0.98026446	0	10
12596	GO:0060999	1	0.956916988	0	22
12597	GO:0061000	1	0.988093537	0	6
12598	GO:0061001	1	0.958901387	0	21
12599	GO:0061002	1	0.988074083	0	6
12600	GO:0061003	1	0.960828773	0	20
12601	GO:0061005	1	0.994042815	0	3
12602	GO:0061009	1	0.995958117	0	2
12603	GO:0061010	1	0.998013437	0	1
12604	GO:0061014	1	0.972360213	0	14
12605	GO:0061015	1	0.995939188	0	2
12606	GO:0061024	1	0.77607738	0	126
12608	GO:0061026	1	0.994051727	0	3
12609	GO:0061028	1	0.964555662	0	18
12610	GO:0061029	1	0.98209595	0	9
12611	GO:0061030	1	0.993955851	0	3
12612	GO:0061031	1	0.997967253	0	1
12613	GO:0061033	1	0.995990716	0	2
12614	GO:0061034	1	0.996027101	0	2
12615	GO:0061035	1	0.989996276	0	5
12616	GO:0061036	1	0.970374749	0	15
12617	GO:0061037	1	0.984002033	0	8
12618	GO:0061038	1	0.988033946	0	6
12619	GO:0061040	1	0.998013437	0	1
12620	GO:0061041	1	0.989950198	0	5

12621	GO:0061042	1	0.986059385	0	7
12622	GO:0061043	1	0.998010813	0	1
12623	GO:0061044	1	0.989951996	0	5
12624	GO:0061045	1	0.968445255	0	16
12625	GO:0061046	1	0.998006804	0	1
12626	GO:0061047	1	0.995979549	0	2
12627	GO:0061048	1	0.997972866	0	1
12628	GO:0061049	1	0.9880723	0	6
12629	GO:0061051	1	0.984006798	0	8
12630	GO:0061052	1	0.982060127	0	9
12631	GO:0061053	1	0.968373622	0	16
12632	GO:0061055	1	0.994026	0	3
12633	GO:0061056	1	0.991965801	0	4
12634	GO:0061061	1	0.980049036	0	10
12635	GO:0061072	1	0.984065705	0	8
12636	GO:0061073	1	0.992018361	0	4
12637	GO:0061074	1	0.995991007	0	2
12638	GO:0061077	1	0.94502289	0	28
12639	GO:0061084	1	0.992001227	0	4
12640	GO:0061085	1	0.997995407	0	1
12641	GO:0061086	1	0.992018481	0	4
12642	GO:0061087	1	0.992016723	0	4
12643	GO:0061088	1	0.990039986	0	5
12644	GO:0061090	1	0.997985088	0	1
12645	GO:0061092	1	0.992068155	0	4
12646	GO:0061097	1	0.994044869	0	3
12647	GO:0061098	1	0.92653019	0	38
12648	GO:0061099	1	0.970298197	0	15
12649	GO:0061100	1	0.997986219	0	1
12650	GO:0061101	1	0.995950243	0	2
12651	GO:0061102	1	0.997986219	0	1
12652	GO:0061103	1	0.997986219	0	1
12653	GO:0061104	1	0.995981797	0	2
12654	GO:0061106	1	0.997968974	0	1
12655	GO:0061108	1	0.99798286	0	1
12656	GO:0061113	1	0.996021486	0	2
12657	GO:0061114	1	0.99799543	0	1
12658	GO:0061133	1	0.979947532	0	10
12659	GO:0061135	1	0.998013437	0	1
12660	GO:0061136	1	0.962387548	0	19
12661	GO:0061138	1	0.994026948	0	3
12662	GO:0061141	1	0.998013437	0	1
12663	GO:0061143	1	0.997992028	0	1
12664	GO:0061144	1	0.997995977	0	1
12665	GO:0061145	1	0.996020806	0	2

12666	GO:0061146	1	0.993996717	0	3
12667	GO:0061149	1	0.997974922	0	1
12668	GO:0061151	1	0.997974922	0	1
12669	GO:0061153	1	0.995969582	0	2
12670	GO:0061154	1	0.983989732	0	8
12671	GO:0061155	1	0.997974922	0	1
12672	GO:0061156	1	0.988053448	0	6
12673	GO:0061157	1	0.966523732	0	17
12674	GO:0061158	1	0.966477321	0	17
12675	GO:0061159	1	0.998012752	0	1
12676	GO:0061162	1	0.996030731	0	2
12677	GO:0061163	1	0.997990564	0	1
12678	GO:0061170	1	0.997956595	0	1
12679	GO:0061178	1	0.964594831	0	18
12680	GO:0061179	1	0.979977539	0	10
12681	GO:0061180	1	0.988025385	0	6
12682	GO:0061181	1	0.994002436	0	3
12683	GO:0061182	1	0.993964546	0	3
12684	GO:0061184	1	0.996003806	0	2
12685	GO:0061185	1	0.997978403	0	1
12686	GO:0061188	1	0.996030632	0	2
12687	GO:0061189	1	0.997971213	0	1
12688	GO:0061193	1	0.99796016	0	1
12689	GO:0061196	1	0.997982996	0	1
12690	GO:0061197	1	0.996006812	0	2
12691	GO:0061198	1	0.9940061	0	3
12692	GO:0061202	1	0.985995804	0	7
12693	GO:0061205	1	0.992018706	0	4
12694	GO:0061290	1	0.996012554	0	2
12695	GO:0061298	1	0.984168467	0	8
12696	GO:0061299	1	0.988068273	0	6
12697	GO:0061300	1	0.997970229	0	1
12698	GO:0061301	1	0.998013437	0	1
12699	GO:0061302	1	0.998005859	0	1
12700	GO:0061303	1	0.985969333	0	7
12701	GO:0061304	1	0.990105068	0	5
12702	GO:0061308	1	0.993967855	0	3
12703	GO:0061309	1	0.995986354	0	2
12704	GO:0061312	1	0.989984789	0	5
12705	GO:0061314	1	0.978164558	0	11
12706	GO:0061317	1	0.997993311	0	1
12707	GO:0061324	1	0.99800067	0	1
12708	GO:0061325	1	0.997982436	0	1
12710	GO:0061333	1	0.992075912	0	4
12711	GO:0061337	1	0.935967625	0	33

12712	GO:0061339	1	0.997990564	0	1
12713	GO:0061343	1	0.998013437	0	1
12714	GO:0061347	1	0.998013437	0	1
12715	GO:0061348	1	0.998013437	0	1
12716	GO:0061349	1	0.998013437	0	1
12717	GO:0061350	1	0.998013437	0	1
12718	GO:0061351	1	0.976094188	0	12
12719	GO:0061354	1	0.998013437	0	1
12720	GO:0061355	1	0.995964372	0	2
12721	GO:0061357	1	0.996002601	0	2
12722	GO:0061358	1	0.997976331	0	1
12723	GO:0061364	1	0.994051216	0	3
12724	GO:0061368	1	0.997967158	0	1
12725	GO:0061369	1	0.998006585	0	1
12726	GO:0061370	1	0.989924952	0	5
12727	GO:0061371	1	0.991963402	0	4
12728	GO:0061374	1	0.997969048	0	1
12729	GO:0061377	1	0.997969048	0	1
12730	GO:0061379	1	0.997969048	0	1
12731	GO:0061381	1	0.997969048	0	1
12732	GO:0061383	1	0.997977247	0	1
12733	GO:0061384	1	0.986064253	0	7
12734	GO:0061386	1	0.998013437	0	1
12735	GO:0061394	1	0.997979348	0	1
12736	GO:0061395	1	0.997973778	0	1
12737	GO:0061400	1	0.997990564	0	1
12738	GO:0061402	1	0.997986047	0	1
12739	GO:0061408	1	0.997981211	0	1
12740	GO:0061418	1	0.867840557	0	70
12741	GO:0061419	1	0.988044394	0	6
12742	GO:0061428	1	0.991982464	0	4
12743	GO:0061430	1	0.998012334	0	1
12744	GO:0061433	1	0.997977718	0	1
12745	GO:0061436	1	0.950960148	0	25
12746	GO:0061441	1	0.996003306	0	2
12747	GO:0061444	1	0.998013437	0	1
12748	GO:0061445	1	0.997993767	0	1
12749	GO:0061448	1	0.989999964	0	5
12750	GO:0061450	1	0.998013436	0	1
12751	GO:0061458	1	0.990021793	0	5
12752	GO:0061459	1	0.99203628	0	4
12753	GO:0061462	1	0.972221092	0	14
12754	GO:0061463	1	0.995985075	0	2
12755	GO:0061469	1	0.984032865	0	8
12756	GO:0061470	1	0.989999411	0	5

12757	GO:0061474	1	0.997987312	0	1
12758	GO:0061481	1	0.997957079	0	1
12759	GO:0061484	1	0.964503364	0	18
12760	GO:0061485	1	0.998013437	0	1
12761	GO:0061501	1	0.997990564	0	1
12762	GO:0061502	1	0.995989265	0	2
12763	GO:0061507	1	0.995986396	0	2
12764	GO:0061508	1	0.997990564	0	1
12765	GO:0061511	1	0.996030469	0	2
12766	GO:0061512	1	0.935781478	0	33
12767	GO:0061513	1	0.993997307	0	3
12768	GO:0061514	1	0.997974835	0	1
12769	GO:0061515	1	0.995979339	0	2
12770	GO:0061518	1	0.984004177	0	8
12771	GO:0061519	1	0.998002934	0	1
12772	GO:0061520	1	0.996002516	0	2
12773	GO:0061525	1	0.99801336	0	1
12774	GO:0061526	1	0.997988914	0	1
12775	GO:0061547	1	0.998000934	0	1
12776	GO:0061548	1	0.997987562	0	1
12777	GO:0061549	1	0.984109488	0	8
12778	GO:0061550	1	0.99800067	0	1
12779	GO:0061551	1	0.990054972	0	5
12780	GO:0061564	1	0.970422129	0	15
12781	GO:0061565	1	0.997990564	0	1
12782	GO:0061566	1	0.997990564	0	1
12783	GO:0061567	1	0.997990564	0	1
12784	GO:0061568	1	0.997990564	0	1
12785	GO:0061569	1	0.997990564	0	1
12786	GO:0061570	1	0.997990564	0	1
12787	GO:0061571	1	0.997990564	0	1
12788	GO:0061572	1	0.995985075	0	2
12789	GO:0061573	1	0.997990564	0	1
12790	GO:0061574	1	0.99200822	0	4
12791	GO:0061575	1	0.98395698	0	8
12792	GO:0061577	1	0.994021029	0	3
12793	GO:0061578	1	0.978183048	0	11
12794	GO:0061580	1	0.998008667	0	1
12795	GO:0061582	1	0.998013437	0	1
12796	GO:0061586	1	0.997999567	0	1
12797	GO:0061588	1	0.988039015	0	6
12798	GO:0061589	1	0.992024241	0	4
12799	GO:0061590	1	0.990053164	0	5
12800	GO:0061591	1	0.99202533	0	4
12801	GO:0061598	1	0.99800978	0	1

12802	GO:0061599	1	0.99800978	0	1
12803	GO:0061604	1	0.997985905	0	1
12804	GO:0061605	1	0.997985905	0	1
12805	GO:0061608	1	0.960694611	0	20
12806	GO:0061609	1	0.997985529	0	1
12807	GO:0061611	1	0.997974765	0	1
12808	GO:0061614	1	0.998005071	0	1
12809	GO:0061615	1	0.993995087	0	3
12810	GO:0061617	1	0.981959573	0	9
12811	GO:0061620	1	0.997987546	0	1
12812	GO:0061621	1	0.956598342	0	22
12813	GO:0061623	1	0.997967309	0	1
12814	GO:0061624	1	0.98997919	0	5
12815	GO:0061626	1	0.984012682	0	8
12816	GO:0061627	1	0.997978503	0	1
12817	GO:0061628	1	0.994021869	0	3
12818	GO:0061629	1	0.987954865	0	6
12820	GO:0061631	1	0.935646055	0	33
12821	GO:0061632	1	0.997988836	0	1
12822	GO:0061635	1	0.978035159	0	11
12823	GO:0061640	1	0.956650657	0	22
12824	GO:0061642	1	0.993990575	0	3
12825	GO:0061643	1	0.994022234	0	3
12826	GO:0061646	1	0.998012768	0	1
12827	GO:0061647	1	0.99598226	0	2
12828	GO:0061649	1	0.994017839	0	3
12829	GO:0061654	1	0.997967083	0	1
12830	GO:0061656	1	0.997964216	0	1
12831	GO:0061657	1	0.997962681	0	1
12832	GO:0061663	1	0.993947479	0	3
12833	GO:0061665	1	0.980070951	0	10
12834	GO:0061666	1	0.997990564	0	1
12835	GO:0061668	1	0.993939662	0	3
12836	GO:0061669	1	0.990067422	0	5
12837	GO:0061670	1	0.99796988	0	1
12838	GO:0061673	1	0.990016071	0	5
12839	GO:0061676	1	0.984129081	0	8
12840	GO:0061684	1	0.986058595	0	7
12841	GO:0061685	1	0.997990564	0	1
12842	GO:0061689	1	0.991989648	0	4
12843	GO:0061690	1	0.997963596	0	1
12844	GO:0061697	1	0.995974424	0	2
12845	GO:0061698	1	0.995974424	0	2
12846	GO:0061699	1	0.995974424	0	2
12847	GO:0061700	1	0.978117189	0	11

12848	GO:0061702	1	0.986085905	0	7
12849	GO:0061708	1	0.997978086	0	1
12850	GO:0061709	1	0.976138513	0	12
12851	GO:0061711	1	0.995958604	0	2
12852	GO:0061712	1	0.997998584	0	1
12853	GO:0061713	1	0.99397432	0	3
12854	GO:0061714	1	0.997961189	0	1
12855	GO:0061715	1	0.99799807	0	1
12856	GO:0061723	1	0.996003175	0	2
12857	GO:0061724	1	0.995958249	0	2
12858	GO:0061727	1	0.997958946	0	1
12859	GO:0061731	1	0.997997463	0	1
12860	GO:0061732	1	0.989992514	0	5
12861	GO:0061733	1	0.988087298	0	6
12862	GO:0061734	1	0.992036861	0	4
12863	GO:0061736	1	0.998013435	0	1
12864	GO:0061737	1	0.991933729	0	4
12865	GO:0061738	1	0.993972659	0	3
12866	GO:0061739	1	0.992005255	0	4
12867	GO:0061740	1	0.993991236	0	3
12868	GO:0061741	1	0.995975129	0	2
12869	GO:0061742	1	0.998008272	0	1
12870	GO:0061743	1	0.99395601	0	3
12871	GO:0061744	1	0.980151816	0	10
12872	GO:0061749	1	0.996028686	0	2
12873	GO:0061750	1	0.997983887	0	1
12874	GO:0061751	1	0.998013267	0	1
12875	GO:0061752	1	0.995975737	0	2
12876	GO:0061753	1	0.998013435	0	1
12877	GO:0061757	1	0.997997916	0	1
12878	GO:0061760	1	0.997955074	0	1
12879	GO:0061762	1	0.992019334	0	4
12880	GO:0061763	1	0.995983133	0	2
12881	GO:0061766	1	0.997941649	0	1
12882	GO:0061767	1	0.998013437	0	1
12883	GO:0061768	1	0.998012558	0	1
12884	GO:0061769	1	0.997990564	0	1
12885	GO:0061770	1	0.997981211	0	1
12886	GO:0061771	1	0.993944102	0	3
12887	GO:0061772	1	0.998000558	0	1
12888	GO:0061789	1	0.994051875	0	3
12889	GO:0061797	1	0.997990564	0	1
12890	GO:0061798	1	0.998009002	0	1
12891	GO:0061799	1	0.998009002	0	1
12892	GO:0061809	1	0.972296546	0	14

12893	GO:0061811	1	0.997969548	0	1
12894	GO:0061812	1	0.997969548	0	1
12895	GO:0061817	1	0.988049748	0	6
12896	GO:0061819	1	0.994004742	0	3
12897	GO:0061820	1	0.982137111	0	9
12898	GO:0061821	1	0.992062278	0	4
12899	GO:0061825	1	0.998006856	0	1
12900	GO:0061827	1	0.987992086	0	6
12901	GO:0061828	1	0.998013437	0	1
12902	GO:0061829	1	0.998013437	0	1
12903	GO:0061830	1	0.998013437	0	1
12904	GO:0061831	1	0.998013437	0	1
12905	GO:0061832	1	0.998013437	0	1
12906	GO:0061833	1	0.995996429	0	2
12907	GO:0061837	1	0.998013433	0	1
12908	GO:0061842	1	0.99797827	0	1
12910	GO:0061845	1	0.997998232	0	1
12911	GO:0061846	1	0.997998232	0	1
12912	GO:0061847	1	0.995989749	0	2
12913	GO:0061849	1	0.996028686	0	2
12914	GO:0061850	1	0.997957984	0	1
12915	GO:0061851	1	0.995963563	0	2
12916	GO:0061855	1	0.998012907	0	1
12917	GO:0061857	1	0.996000723	0	2
12918	GO:0061860	1	0.996029964	0	2
12919	GO:0061863	1	0.998013437	0	1
12920	GO:0061870	1	0.998013437	0	1
12921	GO:0061871	1	0.99800724	0	1
12922	GO:0061881	1	0.997973504	0	1
12923	GO:0061885	1	0.995984816	0	2
12924	GO:0061888	1	0.997963848	0	1
12925	GO:0061889	1	0.991911545	0	4
12926	GO:0061890	1	0.998013437	0	1
12927	GO:0061891	1	0.993991527	0	3
12928	GO:0061896	1	0.998012004	0	1
12929	GO:0061897	1	0.998012004	0	1
12930	GO:0061898	1	0.998012004	0	1
12931	GO:0061899	1	0.998012004	0	1
12932	GO:0061903	1	0.995992683	0	2
12933	GO:0061909	1	0.984122998	0	8
12934	GO:0061910	1	0.997990564	0	1
12935	GO:0061912	1	0.996010213	0	2
12936	GO:0061920	1	0.998013437	0	1
12937	GO:0061921	1	0.998013437	0	1
12938	GO:0061928	1	0.995939333	0	2

12939	GO:0061929	1	0.997990564	0	1
12940	GO:0061944	1	0.99599724	0	2
12941	GO:0061951	1	0.991986751	0	4
12942	GO:0061952	1	0.966257097	0	17
12943	GO:0061953	1	0.993976346	0	3
12944	GO:0061956	1	0.997990564	0	1
12945	GO:0061966	1	0.994007662	0	3
12946	GO:0061974	1	0.997999867	0	1
12947	GO:0061975	1	0.99599976	0	2
12948	GO:0061981	1	0.997969613	0	1
12949	GO:0061987	1	0.997985262	0	1
12950	GO:0062003	1	0.997993682	0	1
12951	GO:0062009	1	0.974347026	0	13
12952	GO:0062023	1	0.538612615	0	306
12953	GO:0062026	1	0.998012203	0	1
12954	GO:0062028	1	0.996030372	0	2
12955	GO:0062029	1	0.996005669	0	2
12956	GO:0062030	1	0.99796836	0	1
12957	GO:0062037	1	0.997982628	0	1
12958	GO:0062043	1	0.996030731	0	2
12959	GO:0062050	1	0.997991408	0	1
12960	GO:0062061	1	0.995976499	0	2
12961	GO:0062064	1	0.998007009	0	1
12962	GO:0062065	1	0.998007009	0	1
12963	GO:0062069	1	0.998003237	0	1
12964	GO:0062072	1	0.997985277	0	1
12965	GO:0062076	1	0.998010751	0	1
12966	GO:0062078	1	0.997990564	0	1
12967	GO:0062082	1	0.997997344	0	1
12968	GO:0062094	1	0.997999867	0	1
12969	GO:0062098	1	0.997987562	0	1
12970	GO:0062099	1	0.993953442	0	3
12971	GO:0062100	1	0.997988013	0	1
12972	GO:0062101	1	0.998003428	0	1
12973	GO:0062103	1	0.99800762	0	1
12974	GO:0062105	1	0.99800266	0	1
12975	GO:0062112	1	0.998004627	0	1
12976	GO:0062124	1	0.997990564	0	1
12977	GO:0062152	1	0.997995399	0	1
12978	GO:0062153	1	0.995966374	0	2
12979	GO:0062154	1	0.997987717	0	1
12980	GO:0062156	1	0.997969916	0	1
12981	GO:0062157	1	0.99598588	0	2
12982	GO:0062173	1	0.997959295	0	1
12983	GO:0062175	1	0.997970082	0	1

12984	GO:0062176	1	0.990015281	0	5
12985	GO:0062177	1	0.995953453	0	2
12986	GO:0062180	1	0.997997008	0	1
12987	GO:0062181	1	0.995991854	0	2
12988	GO:0062182	1	0.997981615	0	1
12989	GO:0062183	1	0.995965589	0	2
12990	GO:0062184	1	0.995979211	0	2
12991	GO:0062185	1	0.997986485	0	1
12992	GO:0062187	1	0.993969443	0	3
12993	GO:0062188	1	0.993969443	0	3
12994	GO:0062189	1	0.991965796	0	4
12995	GO:0062208	1	0.998011501	0	1
12996	GO:0062213	1	0.997969972	0	1
12997	GO:0062234	1	0.995939404	0	2
12998	GO:0062237	1	0.998013437	0	1
12999	GO:0065001	1	0.99603071	0	2
13000	GO:0065002	1	0.993995359	0	3
13001	GO:0065003	1	0.778889072	0	124
13002	GO:0065004	1	0.992000833	0	4
13003	GO:0065010	1	0.997987919	0	1
13004	GO:0070001	1	0.995950753	0	2
13005	GO:0070002	1	0.997966097	0	1
13006	GO:0070004	1	0.993984707	0	3
13007	GO:0070006	1	0.949172102	0	26
13008	GO:0070008	1	0.997979948	0	1
13009	GO:0070012	1	0.994002225	0	3
13010	GO:0070016	1	0.98020611	0	10
13011	GO:0070025	1	0.997988215	0	1
13012	GO:0070026	1	0.993929695	0	3
13013	GO:0070032	1	0.993954328	0	3
13014	GO:0070033	1	0.993985386	0	3
13015	GO:0070034	1	0.956677756	0	22
13016	GO:0070037	1	0.997961268	0	1
13017	GO:0070039	1	0.995970306	0	2
13018	GO:0070042	1	0.997994926	0	1
13019	GO:0070044	1	0.991967605	0	4
13020	GO:0070050	1	0.964499018	0	18
13021	GO:0070051	1	0.992033215	0	4
13022	GO:0070052	1	0.996014252	0	2
13023	GO:0070053	1	0.993993231	0	3
13024	GO:0070054	1	0.998007771	0	1
13025	GO:0070059	1	0.937648944	0	32
13026	GO:0070060	1	0.998013437	0	1
13027	GO:0070061	1	0.989974027	0	5
13028	GO:0070062	1	0.016467427	0	1947

13029	GO:0070063	1	0.964484602	0	18
13030	GO:0070064	1	0.962662662	0	19
13031	GO:0070069	1	0.99400394	0	3
13032	GO:0070070	1	0.995965126	0	2
13033	GO:0070072	1	0.988002203	0	6
13034	GO:0070076	1	0.997988665	0	1
13035	GO:0070078	1	0.998003307	0	1
13036	GO:0070079	1	0.998003307	0	1
13037	GO:0070080	1	0.997959464	0	1
13038	GO:0070083	1	0.991979232	0	4
13039	GO:0070084	1	0.993995043	0	3
13040	GO:0070085	1	0.980086317	0	10
13041	GO:0070086	1	0.994026107	0	3
13042	GO:0070087	1	0.98809548	0	6
13043	GO:0070089	1	0.998013437	0	1
13044	GO:0070091	1	0.997963848	0	1
13045	GO:0070092	1	0.99399835	0	3
13046	GO:0070093	1	0.995970974	0	2
13047	GO:0070094	1	0.995982451	0	2
13048	GO:0070095	1	0.991981184	0	4
13049	GO:0070097	1	0.982117924	0	9
13051	GO:0070100	1	0.986076006	0	7
13052	GO:0070101	1	0.995991753	0	2
13054	GO:0070104	1	0.995974865	0	2
13055	GO:0070105	1	0.993976037	0	3
13057	GO:0070110	1	0.996010011	0	2
13058	GO:0070119	1	0.998008754	0	1
13059	GO:0070120	1	0.99201972	0	4
13060	GO:0070121	1	0.998013416	0	1
13061	GO:0070122	1	0.976001413	0	12
13062	GO:0070123	1	0.998013437	0	1
13063	GO:0070124	1	0.99595581	0	2
13064	GO:0070125	1	0.835801476	0	88
13065	GO:0070126	1	0.834120631	0	89
13066	GO:0070127	1	0.998006752	0	1
13067	GO:0070129	1	0.983973915	0	8
13068	GO:0070130	1	0.997990564	0	1
13069	GO:0070131	1	0.970143267	0	15
13070	GO:0070139	1	0.994035065	0	3
13071	GO:0070140	1	0.994021069	0	3
13072	GO:0070141	1	0.994001562	0	3
13073	GO:0070142	1	0.997978237	0	1
13074	GO:0070143	1	0.998012382	0	1
13075	GO:0070145	1	0.997999739	0	1
13076	GO:0070150	1	0.997990564	0	1

13077	GO:0070158	1	0.997979817	0	1
13078	GO:0070159	1	0.997984805	0	1
13079	GO:0070160	1	0.968307488	0	16
13080	GO:0070164	1	0.992021706	0	4
13081	GO:0070165	1	0.99599829	0	2
13082	GO:0070166	1	0.978206883	0	11
13083	GO:0070168	1	0.982115049	0	9
13084	GO:0070169	1	0.989921755	0	5
13085	GO:0070171	1	0.998013437	0	1
13086	GO:0070172	1	0.995976304	0	2
13087	GO:0070175	1	0.993976137	0	3
13088	GO:0070176	1	0.997975199	0	1
13089	GO:0070178	1	0.997986313	0	1
13090	GO:0070179	1	0.997986313	0	1
13091	GO:0070180	1	0.989836419	0	5
13092	GO:0070181	1	0.98185433	0	9
13093	GO:0070182	1	0.964483631	0	18
13094	GO:0070183	1	0.997991586	0	1
13095	GO:0070184	1	0.997981211	0	1
13096	GO:0070187	1	0.986006857	0	7
13097	GO:0070189	1	0.991999891	0	4
13098	GO:0070191	1	0.997990564	0	1
13099	GO:0070192	1	0.984072692	0	8
13100	GO:0070193	1	0.995950624	0	2
13101	GO:0070194	1	0.995976129	0	2
13102	GO:0070195	1	0.998010412	0	1
13103	GO:0070197	1	0.991999878	0	4
13104	GO:0070198	1	0.978164683	0	11
13105	GO:0070200	1	0.990038715	0	5
13106	GO:0070201	1	0.984096513	0	8
13107	GO:0070202	1	0.997990564	0	1
13108	GO:0070206	1	0.997966363	0	1
13109	GO:0070207	1	0.972152903	0	14
13110	GO:0070208	1	0.998013387	0	1
13111	GO:0070209	1	0.99399242	0	3
13112	GO:0070212	1	0.984103931	0	8
13113	GO:0070213	1	0.976246357	0	12
13114	GO:0070221	1	0.989902835	0	5
13115	GO:0070224	1	0.997990564	0	1
13116	GO:0070228	1	0.997966363	0	1
13117	GO:0070231	1	0.991981027	0	4
13118	GO:0070232	1	0.994000254	0	3
13119	GO:0070233	1	0.985986599	0	7
13120	GO:0070234	1	0.993911974	0	3
13121	GO:0070235	1	0.997977348	0	1

13122	GO:0070236	1	0.993978995	0	3
13123	GO:0070241	1	0.997972973	0	1
13124	GO:0070242	1	0.989993705	0	5
13125	GO:0070243	1	0.995999541	0	2
13126	GO:0070244	1	0.986028025	0	7
13127	GO:0070245	1	0.989982529	0	5
13128	GO:0070251	1	0.995993334	0	2
13129	GO:0070252	1	0.994011052	0	3
13130	GO:0070254	1	0.991916508	0	4
13131	GO:0070256	1	0.995966445	0	2
13132	GO:0070257	1	0.987987039	0	6
13133	GO:0070260	1	0.997990564	0	1
13134	GO:0070262	1	0.972220675	0	14
13135	GO:0070265	1	0.997990564	0	1
13136	GO:0070266	1	0.960598637	0	20
13137	GO:0070267	1	0.998002856	0	1
13138	GO:0070268	1	0.830150937	0	92
13139	GO:0070269	1	0.972145431	0	14
13140	GO:0070273	1	0.94151004	0	30
13141	GO:0070274	1	0.997978736	0	1
13142	GO:0070278	1	0.991979937	0	4
13143	GO:0070279	1	0.997986094	0	1
13144	GO:0070280	1	0.998005759	0	1
13145	GO:0070286	1	0.970246179	0	15
13146	GO:0070290	1	0.994039801	0	3
13147	GO:0070291	1	0.98995872	0	5
13148	GO:0070292	1	0.982056531	0	9
13149	GO:0070293	1	0.987941123	0	6
13150	GO:0070294	1	0.992013199	0	4
13151	GO:0070295	1	0.993967977	0	3
13152	GO:0070296	1	0.992029073	0	4
13153	GO:0070300	1	0.958709817	0	21
13154	GO:0070301	1	0.87736554	0	65
13155	GO:0070302	1	0.997996776	0	1
13156	GO:0070304	1	0.995992381	0	2
13157	GO:0070305	1	0.997969308	0	1
13158	GO:0070306	1	0.980215191	0	10
13159	GO:0070307	1	0.98601898	0	7
13160	GO:0070309	1	0.990039758	0	5
13161	GO:0070314	1	0.988007528	0	6
13162	GO:0070315	1	0.993985286	0	3
13163	GO:0070316	1	0.998006716	0	1
13164	GO:0070317	1	0.924621225	0	39
13165	GO:0070318	1	0.991986047	0	4
13166	GO:0070319	1	0.996000636	0	2

13167	GO:0070320	1	0.997989294	0	1
13168	GO:0070324	1	0.989976922	0	5
13169	GO:0070325	1	0.996030731	0	2
13170	GO:0070326	1	0.991975671	0	4
13171	GO:0070327	1	0.986071999	0	7
13172	GO:0070328	1	0.964295613	0	18
13173	GO:0070330	1	0.948855408	0	26
13174	GO:0070335	1	0.998013437	0	1
13175	GO:0070336	1	0.995992654	0	2
13176	GO:0070337	1	0.998013437	0	1
13177	GO:0070345	1	0.990003864	0	5
13178	GO:0070346	1	0.997989952	0	1
13179	GO:0070349	1	0.997946933	0	1
13180	GO:0070350	1	0.998009966	0	1
13181	GO:0070358	1	0.986041022	0	7
13182	GO:0070365	1	0.976119427	0	12
13183	GO:0070367	1	0.997979199	0	1
13184	GO:0070368	1	0.997963363	0	1
13185	GO:0070369	1	0.994033151	0	3
13186	GO:0070370	1	0.989923197	0	5
13187	GO:0070371	1	0.945220516	0	28
13188	GO:0070372	1	0.945398384	0	28
13190	GO:0070374	1	0.722259799	0	161
13191	GO:0070375	1	0.996001801	0	2
13192	GO:0070378	1	0.995985075	0	2
13193	GO:0070381	1	0.996009713	0	2
13194	GO:0070382	1	0.949210198	0	26
13195	GO:0070383	1	0.981950335	0	9
13196	GO:0070384	1	0.994012035	0	3
13197	GO:0070390	1	0.989900462	0	5
13198	GO:0070392	1	0.997961288	0	1
13199	GO:0070401	1	0.993949913	0	3
13200	GO:0070402	1	0.970242795	0	15
13201	GO:0070403	1	0.968259686	0	16
13202	GO:0070404	1	0.993928247	0	3
13203	GO:0070407	1	0.997996249	0	1
13204	GO:0070409	1	0.998013437	0	1
13205	GO:0070410	1	0.980192505	0	10
13206	GO:0070411	1	0.972413365	0	14
13207	GO:0070412	1	0.958822682	0	21
13208	GO:0070413	1	0.997993713	0	1
13209	GO:0070417	1	0.98210872	0	9
13210	GO:0070418	1	0.996027868	0	2
13211	GO:0070419	1	0.984061477	0	8
13212	GO:0070421	1	0.998000221	0	1

13213	GO:0070423	1	0.954811194	0	23
13214	GO:0070424	1	0.99006863	0	5
13215	GO:0070427	1	0.995963104	0	2
13216	GO:0070429	1	0.998010813	0	1
13217	GO:0070430	1	0.994002971	0	3
13218	GO:0070431	1	0.984012818	0	8
13219	GO:0070433	1	0.994007603	0	3
13220	GO:0070434	1	0.989995429	0	5
13221	GO:0070435	1	0.995990836	0	2
13222	GO:0070436	1	0.996012293	0	2
13223	GO:0070445	1	0.994017281	0	3
13224	GO:0070446	1	0.997977718	0	1
13225	GO:0070447	1	0.994003036	0	3
13226	GO:0070449	1	0.991985904	0	4
13227	GO:0070453	1	0.995933801	0	2
13228	GO:0070455	1	0.997997701	0	1
13229	GO:0070458	1	0.989958954	0	5
13230	GO:0070459	1	0.993963219	0	3
13231	GO:0070460	1	0.997992678	0	1
13232	GO:0070461	1	0.986055036	0	7
13233	GO:0070462	1	0.998003737	0	1
13234	GO:0070463	1	0.998001347	0	1
13235	GO:0070469	1	0.877807086	0	64
13236	GO:0070471	1	0.997987857	0	1
13237	GO:0070472	1	0.99397667	0	3
13238	GO:0070473	1	0.997977802	0	1
13239	GO:0070474	1	0.990036263	0	5
13240	GO:0070475	1	0.987971219	0	6
13241	GO:0070476	1	0.995946304	0	2
13242	GO:0070478	1	0.998009229	0	1
13243	GO:0070481	1	0.99799308	0	1
13244	GO:0070483	1	0.995997555	0	2
13245	GO:0070486	1	0.998011319	0	1
13246	GO:0070487	1	0.994002355	0	3
13247	GO:0070488	1	0.995903923	0	2
13248	GO:0070489	1	0.995988928	0	2
13250	GO:0070492	1	0.986006988	0	7
13251	GO:0070493	1	0.984105737	0	8
13252	GO:0070495	1	0.993994167	0	3
13253	GO:0070498	1	0.825163446	0	95
13254	GO:0070500	1	0.997969714	0	1
13255	GO:0070506	1	0.995982554	0	2
13256	GO:0070507	1	0.934193015	0	34
13258	GO:0070509	1	0.960768888	0	20
13259	GO:0070512	1	0.998013437	0	1

13260	GO:0070513	1	0.989914681	0	5
13261	GO:0070516	1	0.991910798	0	4
13262	GO:0070522	1	0.992007081	0	4
13263	GO:0070524	1	0.997968201	0	1
13264	GO:0070525	1	0.995979282	0	2
13265	GO:0070527	1	0.926407199	0	38
13266	GO:0070528	1	0.974067913	0	13
13267	GO:0070530	1	0.956871603	0	22
13268	GO:0070531	1	0.984022591	0	8
13269	GO:0070534	1	0.91701394	0	43
13270	GO:0070535	1	0.996001053	0	2
13271	GO:0070536	1	0.939738775	0	31
13272	GO:0070537	1	0.993986902	0	3
13273	GO:0070538	1	0.995934616	0	2
13274	GO:0070539	1	0.998001903	0	1
13275	GO:0070541	1	0.993989698	0	3
13277	GO:0070543	1	0.995995251	0	2
13278	GO:0070544	1	0.98021105	0	10
13279	GO:0070545	1	0.993963043	0	3
13280	GO:0070548	1	0.997990564	0	1
13281	GO:0070551	1	0.997990564	0	1
13282	GO:0070552	1	0.989986213	0	5
13283	GO:0070553	1	0.997990564	0	1
13284	GO:0070554	1	0.995996844	0	2
13285	GO:0070555	1	0.935816735	0	33
13286	GO:0070557	1	0.995952751	0	2
13287	GO:0070560	1	0.995986643	0	2
13288	GO:0070561	1	0.994010704	0	3
13289	GO:0070562	1	0.994013144	0	3
13290	GO:0070563	1	0.995997145	0	2
13291	GO:0070564	1	0.99399249	0	3
13292	GO:0070566	1	0.997990564	0	1
13293	GO:0070567	1	0.997990564	0	1
13294	GO:0070568	1	0.995985075	0	2
13295	GO:0070569	1	0.991979039	0	4
13296	GO:0070570	1	0.998007878	0	1
13297	GO:0070571	1	0.996002327	0	2
13298	GO:0070572	1	0.997978753	0	1
13299	GO:0070573	1	0.989998224	0	5
13300	GO:0070574	1	0.990042989	0	5
13301	GO:0070576	1	0.993988058	0	3
13302	GO:0070577	1	0.958833635	0	21
13303	GO:0070578	1	0.982047844	0	9
13304	GO:0070579	1	0.992051538	0	4
13305	GO:0070584	1	0.960482285	0	20

13306	GO:0070585	1	0.988024163	0	6
13307	GO:0070586	1	0.997989766	0	1
13309	GO:0070593	1	0.970436334	0	15
13310	GO:0070602	1	0.994027253	0	3
13311	GO:0070611	1	0.997988758	0	1
13312	GO:0070612	1	0.997988758	0	1
13313	GO:0070613	1	0.98407597	0	8
13314	GO:0070615	1	0.938172259	0	32
13315	GO:0070618	1	0.997989549	0	1
13316	GO:0070625	1	0.99797994	0	1
13317	GO:0070626	1	0.997969493	0	1
13318	GO:0070628	1	0.964389098	0	18
13319	GO:0070633	1	0.980204783	0	10
13320	GO:0070634	1	0.993979097	0	3
13321	GO:0070640	1	0.995972806	0	2
13322	GO:0070643	1	0.997997008	0	1
13323	GO:0070644	1	0.994046923	0	3
13324	GO:0070646	1	0.994006307	0	3
13325	GO:0070649	1	0.994036628	0	3
13326	GO:0070651	1	0.99799308	0	1
13327	GO:0070652	1	0.984034791	0	8
13328	GO:0070653	1	0.997958205	0	1
13329	GO:0070661	1	0.997992508	0	1
13330	GO:0070662	1	0.998013176	0	1
13331	GO:0070663	1	0.997974452	0	1
13332	GO:0070664	1	0.997959464	0	1
13333	GO:0070665	1	0.995942045	0	2
13334	GO:0070667	1	0.99599645	0	2
13335	GO:0070668	1	0.99601261	0	2
13336	GO:0070669	1	0.996030313	0	2
13337	GO:0070670	1	0.994050224	0	3
13338	GO:0070671	1	0.99600523	0	2
13339	GO:0070672	1	0.994051205	0	3
13340	GO:0070673	1	0.993989216	0	3
13341	GO:0070674	1	0.998013436	0	1
13342	GO:0070675	1	0.998013436	0	1
13343	GO:0070676	1	0.995947355	0	2
13344	GO:0070678	1	0.997989441	0	1
13345	GO:0070679	1	0.982243595	0	9
13346	GO:0070681	1	0.993959391	0	3
13347	GO:0070682	1	0.993966187	0	3
13348	GO:0070684	1	0.997962935	0	1
13349	GO:0070685	1	0.997980439	0	1
13350	GO:0070691	1	0.995977927	0	2
13351	GO:0070694	1	0.997990564	0	1

13352	GO:0070695	1	0.990037898	0	5
13353	GO:0070697	1	0.995972588	0	2
13354	GO:0070698	1	0.993948613	0	3
13355	GO:0070699	1	0.994001452	0	3
13356	GO:0070700	1	0.976194863	0	12
13357	GO:0070701	1	0.993969635	0	3
13358	GO:0070715	1	0.997998598	0	1
13359	GO:0070716	1	0.997986094	0	1
13360	GO:0070723	1	0.984146588	0	8
13361	GO:0070724	1	0.994011381	0	3
13362	GO:0070728	1	0.986064878	0	7
13363	GO:0070730	1	0.996021677	0	2
13364	GO:0070731	1	0.998006763	0	1
13365	GO:0070733	1	0.995967109	0	2
13366	GO:0070734	1	0.990034787	0	5
13367	GO:0070735	1	0.995994923	0	2
13368	GO:0070736	1	0.998000432	0	1
13369	GO:0070739	1	0.996011241	0	2
13370	GO:0070740	1	0.980109652	0	10
13371	GO:0070741	1	0.995965185	0	2
13372	GO:0070742	1	0.97816352	0	11
13373	GO:0070743	1	0.997961071	0	1
13375	GO:0070761	1	0.985944522	0	7
13376	GO:0070762	1	0.997990564	0	1
13377	GO:0070765	1	0.98602309	0	7
13378	GO:0070772	1	0.997996022	0	1
13379	GO:0070773	1	0.997990564	0	1
13380	GO:0070774	1	0.998010588	0	1
13381	GO:0070776	1	0.982127674	0	9
13382	GO:0070777	1	0.998004621	0	1
13383	GO:0070778	1	0.984068353	0	8
13384	GO:0070779	1	0.992031033	0	4
13385	GO:0070780	1	0.995967092	0	2
13386	GO:0070781	1	0.998013437	0	1
13387	GO:0070782	1	0.986059471	0	7
13388	GO:0070813	1	0.995954135	0	2
13389	GO:0070814	1	0.991946303	0	4
13390	GO:0070815	1	0.998003307	0	1
13391	GO:0070816	1	0.982050591	0	9
13392	GO:0070820	1	0.991993737	0	4
13393	GO:0070821	1	0.882761128	0	62
13394	GO:0070822	1	0.990020676	0	5
13395	GO:0070826	1	0.998009073	0	1
13396	GO:0070827	1	0.996003722	0	2
13397	GO:0070828	1	0.984158105	0	8

13398	GO:0070829	1	0.992044782	0	4
13399	GO:0070830	1	0.896930056	0	54
13400	GO:0070831	1	0.980183093	0	10
13401	GO:0070836	1	0.993979062	0	3
13402	GO:0070837	1	0.99000367	0	5
13403	GO:0070839	1	0.998006135	0	1
13404	GO:0070840	1	0.953076466	0	24
13405	GO:0070842	1	0.988020338	0	6
13406	GO:0070845	1	0.998008667	0	1
13407	GO:0070846	1	0.998008667	0	1
13408	GO:0070847	1	0.983984286	0	8
13409	GO:0070848	1	0.968299242	0	16
13410	GO:0070849	1	0.992004132	0	4
13411	GO:0070851	1	0.980135948	0	10
13412	GO:0070852	1	0.99002013	0	5
13413	GO:0070853	1	0.998013437	0	1
13414	GO:0070856	1	0.998013437	0	1
13415	GO:0070857	1	0.993991536	0	3
13416	GO:0070858	1	0.99799543	0	1
13418	GO:0070860	1	0.997983585	0	1
13419	GO:0070861	1	0.998013437	0	1
13420	GO:0070862	1	0.997989952	0	1
13421	GO:0070863	1	0.980207485	0	10
13422	GO:0070868	1	0.998013437	0	1
13423	GO:0070873	1	0.989919601	0	5
13424	GO:0070876	1	0.992006441	0	4
13425	GO:0070877	1	0.996005372	0	2
13426	GO:0070878	1	0.984126513	0	8
13427	GO:0070883	1	0.987995328	0	6
13428	GO:0070884	1	0.996009564	0	2
13429	GO:0070885	1	0.974183721	0	13
13430	GO:0070886	1	0.974230002	0	13
13431	GO:0070888	1	0.93588257	0	33
13432	GO:0070889	1	0.997989069	0	1
13433	GO:0070891	1	0.993943549	0	3
13434	GO:0070892	1	0.997979792	0	1
13435	GO:0070895	1	0.996008432	0	2
13436	GO:0070897	1	0.993965935	0	3
13437	GO:0070898	1	0.998013437	0	1
13438	GO:0070899	1	0.997994451	0	1
13439	GO:0070900	1	0.995963779	0	2
13440	GO:0070901	1	0.989969221	0	5
13441	GO:0070902	1	0.995962975	0	2
13442	GO:0070904	1	0.99600176	0	2
13443	GO:0070905	1	0.991981953	0	4

13444	GO:0070911	1	0.949007092	0	26
13445	GO:0070914	1	0.978180078	0	11
13446	GO:0070915	1	0.991955896	0	4
13447	GO:0070922	1	0.998011759	0	1
13448	GO:0070925	1	0.988071271	0	6
13449	GO:0070926	1	0.9980062	0	1
13450	GO:0070931	1	0.998013436	0	1
13451	GO:0070932	1	0.962616609	0	19
13452	GO:0070933	1	0.978167678	0	11
13453	GO:0070934	1	0.990061614	0	5
13454	GO:0070935	1	0.972280804	0	14
13456	GO:0070937	1	0.988089682	0	6
13457	GO:0070938	1	0.988052528	0	6
13458	GO:0070939	1	0.994012039	0	3
13459	GO:0070940	1	0.990021514	0	5
13460	GO:0070944	1	0.993948646	0	3
13461	GO:0070945	1	0.997973115	0	1
13462	GO:0070948	1	0.995969164	0	2
13463	GO:0070951	1	0.997972706	0	1
13464	GO:0070963	1	0.997992508	0	1
13465	GO:0070966	1	0.994026903	0	3
13466	GO:0070971	1	0.939768065	0	31
13467	GO:0070972	1	0.987985802	0	6
13468	GO:0070973	1	0.982159127	0	9
13469	GO:0070974	1	0.997995025	0	1
13470	GO:0070975	1	0.998013437	0	1
13471	GO:0070976	1	0.997989906	0	1
13472	GO:0070977	1	0.996025193	0	2
13473	GO:0070979	1	0.94308321	0	29
13474	GO:0070980	1	0.997984378	0	1
13475	GO:0070981	1	0.997983919	0	1
13476	GO:0070982	1	0.997973778	0	1
13477	GO:0070985	1	0.993917522	0	3
13478	GO:0070986	1	0.972339559	0	14
13479	GO:0070987	1	0.956655538	0	22
13480	GO:0070988	1	0.989981623	0	5
13481	GO:0070989	1	0.970236259	0	15
13482	GO:0070990	1	0.995950592	0	2
13483	GO:0070991	1	0.993990793	0	3
13484	GO:0070994	1	0.995974445	0	2
13485	GO:0070995	1	0.990003079	0	5
13486	GO:0070996	1	0.995951561	0	2
13487	GO:0070997	1	0.982184013	0	9
13488	GO:0071000	1	0.995978131	0	2
13489	GO:0071001	1	0.993969159	0	3

13490	GO:0071004	1	0.968180017	0	16
13491	GO:0071005	1	0.903756724	0	50
13492	GO:0071006	1	0.976164104	0	12
13493	GO:0071007	1	0.941131178	0	30
13494	GO:0071008	1	0.993989899	0	3
13495	GO:0071011	1	0.962232358	0	19
13496	GO:0071013	1	0.843957664	0	84
13497	GO:0071014	1	0.980043133	0	10
13498	GO:0071020	1	0.997974592	0	1
13499	GO:0071021	1	0.997973628	0	1
13500	GO:0071025	1	0.99799308	0	1
13501	GO:0071028	1	0.981933632	0	9
13502	GO:0071033	1	0.997973698	0	1
13503	GO:0071034	1	0.991973183	0	4
13504	GO:0071035	1	0.987899606	0	6
13505	GO:0071036	1	0.997991083	0	1
13506	GO:0071037	1	0.997991083	0	1
13507	GO:0071038	1	0.987899606	0	6
13508	GO:0071039	1	0.997991083	0	1
13509	GO:0071040	1	0.997991083	0	1
13510	GO:0071042	1	0.9919391	0	4
13511	GO:0071044	1	0.98013181	0	10
13512	GO:0071045	1	0.997972474	0	1
13513	GO:0071048	1	0.995968689	0	2
13514	GO:0071049	1	0.995948959	0	2
13515	GO:0071050	1	0.997990564	0	1
13516	GO:0071051	1	0.985906761	0	7
13517	GO:0071062	1	0.99602635	0	2
13518	GO:0071065	1	0.995994427	0	2
13519	GO:0071071	1	0.995956398	0	2
13520	GO:0071072	1	0.996019725	0	2
13521	GO:0071073	1	0.991942533	0	4
13522	GO:0071074	1	0.993986841	0	3
13523	GO:0071076	1	0.989992208	0	5
13524	GO:0071103	1	0.995991815	0	2
13525	GO:0071104	1	0.998013384	0	1
13526	GO:0071107	1	0.993984826	0	3
13527	GO:0071108	1	0.937984851	0	32
13528	GO:0071109	1	0.996030731	0	2
13529	GO:0071110	1	0.998013437	0	1
13530	GO:0071133	1	0.997996656	0	1
13531	GO:0071139	1	0.998013437	0	1
13532	GO:0071140	1	0.99600562	0	2
13533	GO:0071141	1	0.984142081	0	8
13534	GO:0071144	1	0.98415749	0	8

13535	GO:0071156	1	0.987981158	0	6
13536	GO:0071157	1	0.95854072	0	21
13537	GO:0071158	1	0.94519472	0	28
13538	GO:0071159	1	0.995948946	0	2
13539	GO:0071162	1	0.980111079	0	10
13540	GO:0071163	1	0.995957896	0	2
13541	GO:0071164	1	0.998004245	0	1
13542	GO:0071166	1	0.997981744	0	1
13543	GO:0071168	1	0.974221594	0	13
13544	GO:0071169	1	0.984003155	0	8
13545	GO:0071171	1	0.997990564	0	1
13546	GO:0071203	1	0.976188407	0	12
13547	GO:0071204	1	0.987978561	0	6
13548	GO:0071205	1	0.992074382	0	4
13549	GO:0071206	1	0.998013437	0	1
13550	GO:0071207	1	0.99598962	0	2
13551	GO:0071208	1	0.991885032	0	4
13552	GO:0071209	1	0.99392455	0	3
13553	GO:0071211	1	0.998013435	0	1
13554	GO:0071218	1	0.984067198	0	8
13555	GO:0071219	1	0.987997514	0	6
13556	GO:0071221	1	0.9899998	0	5
13558	GO:0071223	1	0.982029081	0	9
13559	GO:0071224	1	0.987906952	0	6
13560	GO:0071225	1	0.982070528	0	9
13561	GO:0071226	1	0.992001079	0	4
13562	GO:0071228	1	0.995959356	0	2
13563	GO:0071230	1	0.90084669	0	52
13564	GO:0071231	1	0.997961189	0	1
13565	GO:0071233	1	0.980190076	0	10
13566	GO:0071236	1	0.982105116	0	9
13567	GO:0071241	1	0.996007675	0	2
13568	GO:0071242	1	0.990045642	0	5
13569	GO:0071243	1	0.981964422	0	9
13570	GO:0071245	1	0.998003555	0	1
13571	GO:0071247	1	0.997962143	0	1
13572	GO:0071248	1	0.989967122	0	5
13573	GO:0071250	1	0.998013437	0	1
13574	GO:0071253	1	0.988065755	0	6
13575	GO:0071257	1	0.97818809	0	11
13576	GO:0071258	1	0.99800882	0	1
13577	GO:0071259	1	0.997986219	0	1
13578	GO:0071260	1	0.861604762	0	74
13579	GO:0071261	1	0.997949342	0	1
13580	GO:0071264	1	0.9960223	0	2

13581	GO:0071267	1	0.995969138	0	2
13582	GO:0071275	1	0.997981065	0	1
13583	GO:0071276	1	0.9334008	0	34
13585	GO:0071279	1	0.991968919	0	4
13586	GO:0071280	1	0.954479818	0	23
13587	GO:0071281	1	0.986012815	0	7
13588	GO:0071283	1	0.995944113	0	2
13589	GO:0071284	1	0.992045692	0	4
13590	GO:0071285	1	0.978123292	0	11
13592	GO:0071287	1	0.986107725	0	7
13593	GO:0071288	1	0.992010086	0	4
13594	GO:0071294	1	0.962209761	0	19
13595	GO:0071295	1	0.998011551	0	1
13596	GO:0071298	1	0.998000656	0	1
13597	GO:0071300	1	0.888127614	0	59
13598	GO:0071301	1	0.998013437	0	1
13599	GO:0071305	1	0.980106613	0	10
13600	GO:0071306	1	0.99599357	0	2
13601	GO:0071307	1	0.995988479	0	2
13602	GO:0071310	1	0.943322265	0	29
13603	GO:0071312	1	0.986039427	0	7
13605	GO:0071314	1	0.988070736	0	6
13606	GO:0071315	1	0.994008946	0	3
13607	GO:0071316	1	0.987946487	0	6
13609	GO:0071320	1	0.908321731	0	48
13610	GO:0071321	1	0.988114711	0	6
13611	GO:0071322	1	0.994022914	0	3
13612	GO:0071329	1	0.998012893	0	1
13613	GO:0071332	1	0.991985269	0	4
13614	GO:0071333	1	0.900813728	0	52
13615	GO:0071339	1	0.941428833	0	30
13616	GO:0071340	1	0.986027288	0	7
13617	GO:0071344	1	0.995941821	0	2
13621	GO:0071348	1	0.998012334	0	1
13622	GO:0071349	1	0.996025739	0	2
13623	GO:0071352	1	0.997994528	0	1
13624	GO:0071353	1	0.95667345	0	22
13625	GO:0071354	1	0.968314688	0	16
13628	GO:0071359	1	0.994035507	0	3
13630	GO:0071361	1	0.972353813	0	14
13631	GO:0071363	1	0.886367768	0	60
13632	GO:0071364	1	0.913617096	0	45
13633	GO:0071371	1	0.988036013	0	6
13634	GO:0071372	1	0.984095174	0	8
13635	GO:0071373	1	0.99598451	0	2

13636	GO:0071374	1	0.986051401	0	7
13637	GO:0071375	1	0.964346043	0	18
13638	GO:0071376	1	0.994015239	0	3
13639	GO:0071377	1	0.960699047	0	20
13640	GO:0071378	1	0.995977696	0	2
13641	GO:0071379	1	0.993966238	0	3
13642	GO:0071380	1	0.966490528	0	17
13643	GO:0071383	1	0.974308283	0	13
13644	GO:0071384	1	0.997964698	0	1
13645	GO:0071385	1	0.962537804	0	19
13646	GO:0071386	1	0.996030731	0	2
13647	GO:0071387	1	0.998013198	0	1
13648	GO:0071389	1	0.995974741	0	2
13649	GO:0071391	1	0.970378379	0	15
13650	GO:0071392	1	0.941522304	0	30
13651	GO:0071393	1	0.996025384	0	2
13652	GO:0071394	1	0.982089847	0	9
13653	GO:0071395	1	0.991890451	0	4
13654	GO:0071396	1	0.98994511	0	5
13655	GO:0071397	1	0.968469631	0	16
13656	GO:0071398	1	0.958753608	0	21
13657	GO:0071400	1	0.993986848	0	3
13658	GO:0071401	1	0.997983307	0	1
13659	GO:0071403	1	0.996000622	0	2
13660	GO:0071404	1	0.968375236	0	16
13662	GO:0071409	1	0.991987336	0	4
13663	GO:0071415	1	0.995974394	0	2
13664	GO:0071417	1	0.993964393	0	3
13665	GO:0071418	1	0.995946909	0	2
13666	GO:0071420	1	0.988106487	0	6
13667	GO:0071421	1	0.984106432	0	8
13668	GO:0071422	1	0.986001323	0	7
13669	GO:0071423	1	0.991950994	0	4
13670	GO:0071424	1	0.997990564	0	1
13671	GO:0071425	1	0.972287746	0	14
13672	GO:0071439	1	0.98604989	0	7
13673	GO:0071440	1	0.997990564	0	1
13674	GO:0071441	1	0.998005942	0	1
13675	GO:0071442	1	0.991989607	0	4
13676	GO:0071447	1	0.988071499	0	6
13677	GO:0071449	1	0.997958586	0	1
13678	GO:0071451	1	0.995953059	0	2
13679	GO:0071453	1	0.994001104	0	3
13680	GO:0071454	1	0.998013437	0	1
13681	GO:0071455	1	0.986057696	0	7

13682	GO:0071456	1	0.798199021	0	112
13683	GO:0071458	1	0.990011614	0	5
13684	GO:0071459	1	0.986052442	0	7
13685	GO:0071460	1	0.995933787	0	2
13686	GO:0071461	1	0.991969149	0	4
13687	GO:0071464	1	0.994013722	0	3
13688	GO:0071466	1	0.987973835	0	6
13689	GO:0071467	1	0.985953001	0	7
13690	GO:0071468	1	0.984095834	0	8
13691	GO:0071470	1	0.982137216	0	9
13692	GO:0071471	1	0.998011162	0	1
13693	GO:0071472	1	0.993995078	0	3
13694	GO:0071474	1	0.986074036	0	7
13695	GO:0071475	1	0.993961816	0	3
13696	GO:0071476	1	0.984079902	0	8
13697	GO:0071477	1	0.996014095	0	2
13698	GO:0071478	1	0.993974207	0	3
13699	GO:0071479	1	0.93196246	0	35
13700	GO:0071480	1	0.941428046	0	30
13701	GO:0071481	1	0.978204454	0	11
13702	GO:0071482	1	0.984048469	0	8
13703	GO:0071486	1	0.998013267	0	1
13704	GO:0071492	1	0.980044868	0	10
13705	GO:0071493	1	0.983968022	0	8
13706	GO:0071494	1	0.987989826	0	6
13707	GO:0071498	1	0.974224607	0	13
13708	GO:0071499	1	0.984005012	0	8
13709	GO:0071500	1	0.994019251	0	3
13710	GO:0071501	1	0.998012924	0	1
13711	GO:0071502	1	0.995995053	0	2
13712	GO:0071503	1	0.997999867	0	1
13713	GO:0071504	1	0.990056492	0	5
13714	GO:0071506	1	0.995992831	0	2
13715	GO:0071513	1	0.997982179	0	1
13716	GO:0071514	1	0.995985989	0	2
13717	GO:0071517	1	0.998013437	0	1
13718	GO:0071526	1	0.930633943	0	36
13719	GO:0071528	1	0.998006394	0	1
13720	GO:0071529	1	0.997987919	0	1
13721	GO:0071532	1	0.992031467	0	4
13722	GO:0071535	1	0.991992408	0	4
13723	GO:0071539	1	0.958792206	0	21
13724	GO:0071540	1	0.995987387	0	2
13725	GO:0071541	1	0.985935145	0	7
13726	GO:0071542	1	0.962493808	0	19

13727	GO:0071543	1	0.98997282	0	5
13728	GO:0071544	1	0.997967045	0	1
13729	GO:0071546	1	0.986072177	0	7
13730	GO:0071547	1	0.990002801	0	5
13731	GO:0071548	1	0.982081858	0	9
13732	GO:0071549	1	0.949026452	0	26
13733	GO:0071550	1	0.983989889	0	8
13734	GO:0071556	1	0.950602218	0	25
13735	GO:0071557	1	0.990082313	0	5
13736	GO:0071558	1	0.990082313	0	5
13737	GO:0071559	1	0.980192431	0	10
13738	GO:0071560	1	0.897310428	0	54
13739	GO:0071561	1	0.998012984	0	1
13740	GO:0071564	1	0.976206045	0	12
13741	GO:0071565	1	0.972329561	0	14
13742	GO:0071566	1	0.997993574	0	1
13743	GO:0071567	1	0.997984639	0	1
13744	GO:0071568	1	0.995957246	0	2
13745	GO:0071569	1	0.9879367	0	6
13746	GO:0071575	1	0.998003063	0	1
13747	GO:0071577	1	0.960558049	0	20
13748	GO:0071578	1	0.988031926	0	6
13749	GO:0071579	1	0.99799209	0	1
13750	GO:0071584	1	0.997978852	0	1
13751	GO:0071585	1	0.997978852	0	1
13752	GO:0071586	1	0.993949571	0	3
13753	GO:0071593	1	0.993968033	0	3
13754	GO:0071595	1	0.995985075	0	2
13755	GO:0071596	1	0.994051737	0	3
13756	GO:0071598	1	0.991980347	0	4
13757	GO:0071599	1	0.986112623	0	7
13758	GO:0071600	1	0.998006866	0	1
13759	GO:0071602	1	0.998010588	0	1
13760	GO:0071603	1	0.992024905	0	4
13761	GO:0071604	1	0.996002516	0	2
13762	GO:0071614	1	0.997976246	0	1
13763	GO:0071617	1	0.991983409	0	4
13764	GO:0071621	1	0.99596269	0	2
13765	GO:0071622	1	0.997978345	0	1
13766	GO:0071624	1	0.995913963	0	2
13767	GO:0071625	1	0.972455675	0	14
13768	GO:0071629	1	0.997972474	0	1
13769	GO:0071630	1	0.996030731	0	2
13770	GO:0071633	1	0.993976586	0	3
13771	GO:0071635	1	0.994005385	0	3

13772	GO:0071636	1	0.982104252	0	9
13773	GO:0071638	1	0.989967233	0	5
13775	GO:0071640	1	0.997961288	0	1
13776	GO:0071641	1	0.994000359	0	3
13777	GO:0071642	1	0.995995864	0	2
13778	GO:0071644	1	0.997978287	0	1
13780	GO:0071651	1	0.988015964	0	6
13781	GO:0071654	1	0.997986797	0	1
13782	GO:0071656	1	0.997983824	0	1
13783	GO:0071657	1	0.991964481	0	4
13785	GO:0071660	1	0.998013437	0	1
13786	GO:0071663	1	0.997994489	0	1
13787	GO:0071664	1	0.998007407	0	1
13788	GO:0071665	1	0.997999867	0	1
13789	GO:0071670	1	0.996013439	0	2
13790	GO:0071672	1	0.993961377	0	3
13791	GO:0071673	1	0.993973793	0	3
13792	GO:0071674	1	0.997968117	0	1
13793	GO:0071676	1	0.998012848	0	1
13794	GO:0071677	1	0.993934896	0	3
13795	GO:0071678	1	0.998013437	0	1
13796	GO:0071679	1	0.976280883	0	12
13797	GO:0071681	1	0.99007035	0	5
13798	GO:0071682	1	0.966147597	0	17
13799	GO:0071691	1	0.994051836	0	3
13800	GO:0071701	1	0.998012544	0	1
13801	GO:0071702	1	0.987996057	0	6
13802	GO:0071704	1	0.990015092	0	5
13803	GO:0071705	1	0.994009224	0	3
13804	GO:0071707	1	0.995957565	0	2
13805	GO:0071709	1	0.992023053	0	4
13806	GO:0071711	1	0.972352529	0	14
13807	GO:0071712	1	0.974184206	0	13
13808	GO:0071714	1	0.998002231	0	1
13809	GO:0071716	1	0.986138878	0	7
13810	GO:0071718	1	0.998008003	0	1
13811	GO:0071723	1	0.990021639	0	5
13812	GO:0071726	1	0.991988622	0	4
13813	GO:0071727	1	0.993975652	0	3
13814	GO:0071731	1	0.991922974	0	4
13815	GO:0071732	1	0.970232374	0	15
13816	GO:0071733	1	0.998013437	0	1
13817	GO:0071751	1	0.998009857	0	1
13818	GO:0071763	1	0.987992857	0	6
13819	GO:0071765	1	0.998009411	0	1

13820	GO:0071773	1	0.943585622	0	29
13821	GO:0071774	1	0.986054483	0	7
13822	GO:0071782	1	0.966425865	0	17
13823	GO:0071786	1	0.978093922	0	11
13824	GO:0071787	1	0.991972918	0	4
13825	GO:0071788	1	0.997990564	0	1
13826	GO:0071795	1	0.997990564	0	1
13827	GO:0071796	1	0.995960503	0	2
13828	GO:0071797	1	0.99198205	0	4
13829	GO:0071799	1	0.991984894	0	4
13830	GO:0071800	1	0.992008728	0	4
13831	GO:0071801	1	0.993983238	0	3
13832	GO:0071803	1	0.98208246	0	9
13833	GO:0071805	1	0.791995845	0	116
13834	GO:0071806	1	0.983982633	0	8
13835	GO:0071812	1	0.995983436	0	2
13836	GO:0071813	1	0.987941959	0	6
13837	GO:0071816	1	0.964408502	0	18
13838	GO:0071817	1	0.989990503	0	5
13839	GO:0071818	1	0.99396609	0	3
13840	GO:0071819	1	0.995958664	0	2
13841	GO:0071820	1	0.997968974	0	1
13842	GO:0071821	1	0.989983471	0	5
13843	GO:0071830	1	0.997963771	0	1
13844	GO:0071831	1	0.997963771	0	1
13845	GO:0071837	1	0.976245972	0	12
13846	GO:0071838	1	0.997999789	0	1
13847	GO:0071839	1	0.997986007	0	1
13848	GO:0071846	1	0.995966063	0	2
13849	GO:0071847	1	0.990011254	0	5
13850	GO:0071848	1	0.995983436	0	2
13851	GO:0071850	1	0.96636871	0	17
13852	GO:0071855	1	0.997964756	0	1
13853	GO:0071863	1	0.997979472	0	1
13854	GO:0071864	1	0.989986198	0	5
13855	GO:0071866	1	0.993988429	0	3
13856	GO:0071869	1	0.997983458	0	1
13857	GO:0071870	1	0.988049212	0	6
13858	GO:0071871	1	0.99600081	0	2
13859	GO:0071872	1	0.974302091	0	13
13860	GO:0071873	1	0.998013437	0	1
13861	GO:0071874	1	0.995991286	0	2
13862	GO:0071875	1	0.980124143	0	10
13863	GO:0071878	1	0.998009002	0	1
13864	GO:0071879	1	0.997981631	0	1

13865	GO:0071880	1	0.976153881	0	12
13866	GO:0071881	1	0.9980062	0	1
13867	GO:0071882	1	0.9980062	0	1
13868	GO:0071885	1	0.997990564	0	1
13869	GO:0071887	1	0.993963127	0	3
13870	GO:0071888	1	0.995966519	0	2
13871	GO:0071889	1	0.937793294	0	32
13872	GO:0071890	1	0.998013217	0	1
13873	GO:0071893	1	0.997974922	0	1
13874	GO:0071894	1	0.998000642	0	1
13875	GO:0071895	1	0.995982629	0	2
13876	GO:0071896	1	0.990067465	0	5
13877	GO:0071897	1	0.932120623	0	35
13878	GO:0071899	1	0.99398193	0	3
13879	GO:0071900	1	0.99201307	0	4
13880	GO:0071901	1	0.962563478	0	19
13881	GO:0071902	1	0.90614272	0	49
13882	GO:0071907	1	0.990024607	0	5
13883	GO:0071908	1	0.998013355	0	1
13884	GO:0071909	1	0.998013355	0	1
13885	GO:0071910	1	0.990071941	0	5
13886	GO:0071911	1	0.99801243	0	1
13887	GO:0071913	1	0.997971738	0	1
13888	GO:0071914	1	0.996016644	0	2
13889	GO:0071916	1	0.992003818	0	4
13890	GO:0071918	1	0.989972982	0	5
13891	GO:0071919	1	0.997999874	0	1
13892	GO:0071920	1	0.995973093	0	2
13893	GO:0071921	1	0.996007903	0	2
13894	GO:0071922	1	0.99199098	0	4
13895	GO:0071926	1	0.994003906	0	3
13896	GO:0071929	1	0.995990778	0	2
13897	GO:0071930	1	0.994027207	0	3
13898	GO:0071931	1	0.993964374	0	3
13899	GO:0071932	1	0.994026471	0	3
13900	GO:0071933	1	0.974268318	0	13
13901	GO:0071934	1	0.993992799	0	3
13902	GO:0071936	1	0.996030416	0	2
13903	GO:0071939	1	0.997992028	0	1
13904	GO:0071942	1	0.993985643	0	3
13905	GO:0071944	1	0.873842526	0	67
13906	GO:0071947	1	0.990084213	0	5
13907	GO:0071949	1	0.935883372	0	33
13908	GO:0071951	1	0.997990495	0	1
13909	GO:0071953	1	0.989971277	0	5

13910	GO:0071954	1	0.997974207	0	1
13911	GO:0071955	1	0.996029945	0	2
13912	GO:0071962	1	0.993980906	0	3
13913	GO:0071963	1	0.995989555	0	2
13914	GO:0071966	1	0.997984457	0	1
13915	GO:0071971	1	0.997989991	0	1
13916	GO:0071976	1	0.997998817	0	1
13917	GO:0071985	1	0.978083422	0	11
13918	GO:0071986	1	0.980052559	0	10
13919	GO:0071987	1	0.992029995	0	4
13920	GO:0072003	1	0.997969178	0	1
13921	GO:0072006	1	0.976311107	0	12
13922	GO:0072010	1	0.998000906	0	1
13923	GO:0072011	1	0.99394185	0	3
13924	GO:0072012	1	0.992028212	0	4
13925	GO:0072014	1	0.992013352	0	4
13926	GO:0072015	1	0.982214286	0	9
13927	GO:0072016	1	0.997988743	0	1
13928	GO:0072017	1	0.994051874	0	3
13929	GO:0072028	1	0.997981065	0	1
13930	GO:0072033	1	0.99601115	0	2
13931	GO:0072034	1	0.994020155	0	3
13932	GO:0072038	1	0.99595425	0	2
13933	GO:0072040	1	0.998007874	0	1
13934	GO:0072044	1	0.994007787	0	3
13935	GO:0072046	1	0.997969178	0	1
13936	GO:0072047	1	0.997983585	0	1
13937	GO:0072049	1	0.993930391	0	3
13938	GO:0072050	1	0.991948462	0	4
13939	GO:0072053	1	0.996011234	0	2
13940	GO:0072054	1	0.996011234	0	2
13941	GO:0072060	1	0.998006669	0	1
13942	GO:0072061	1	0.993980009	0	3
13943	GO:0072070	1	0.9960164	0	2
13944	GO:0072073	1	0.994044064	0	3
13945	GO:0072075	1	0.98803977	0	6
13946	GO:0072076	1	0.997982868	0	1
13947	GO:0072077	1	0.997983203	0	1
13948	GO:0072078	1	0.997969178	0	1
13949	GO:0072079	1	0.995978081	0	2
13950	GO:0072080	1	0.997973451	0	1
13951	GO:0072086	1	0.993951104	0	3
13952	GO:0072089	1	0.982166987	0	9
13953	GO:0072091	1	0.988013608	0	6
13954	GO:0072092	1	0.994051628	0	3

13955	GO:0072095	1	0.996006812	0	2
13956	GO:0072097	1	0.997974922	0	1
13957	GO:0072101	1	0.997974922	0	1
13958	GO:0072102	1	0.994034871	0	3
13959	GO:0072104	1	0.99599229	0	2
13960	GO:0072105	1	0.996016151	0	2
13961	GO:0072107	1	0.991975811	0	4
13962	GO:0072108	1	0.993978614	0	3
13963	GO:0072110	1	0.9979964	0	1
13964	GO:0072111	1	0.995964155	0	2
13965	GO:0072112	1	0.986013684	0	7
13966	GO:0072125	1	0.995986738	0	2
13967	GO:0072126	1	0.99000624	0	5
13968	GO:0072133	1	0.994009812	0	3
13969	GO:0072134	1	0.996025179	0	2
13971	GO:0072137	1	0.99204425	0	4
13972	GO:0072138	1	0.993966839	0	3
13973	GO:0072139	1	0.995992274	0	2
13974	GO:0072141	1	0.997968974	0	1
13975	GO:0072143	1	0.997976774	0	1
13976	GO:0072144	1	0.991976532	0	4
13977	GO:0072148	1	0.997992399	0	1
13978	GO:0072156	1	0.996026485	0	2
13979	GO:0072160	1	0.996030211	0	2
13980	GO:0072161	1	0.993958481	0	3
13982	GO:0072163	1	0.998005652	0	1
13983	GO:0072164	1	0.988050222	0	6
13984	GO:0072166	1	0.997976774	0	1
13985	GO:0072168	1	0.997976774	0	1
13986	GO:0072169	1	0.997976774	0	1
13987	GO:0072170	1	0.995979938	0	2
13988	GO:0072172	1	0.997989472	0	1
13989	GO:0072173	1	0.997990719	0	1
13990	GO:0072174	1	0.99597972	0	2
13991	GO:0072175	1	0.998013437	0	1
13992	GO:0072177	1	0.990039461	0	5
13993	GO:0072178	1	0.992028878	0	4
13994	GO:0072179	1	0.997995407	0	1
13995	GO:0072180	1	0.995964155	0	2
13996	GO:0072181	1	0.997969178	0	1
13997	GO:0072182	1	0.995999994	0	2
13998	GO:0072183	1	0.997976774	0	1
13999	GO:0072184	1	0.997976774	0	1
14000	GO:0072189	1	0.992065014	0	4
14001	GO:0072190	1	0.995987519	0	2

14002	GO:0072192	1	0.997974922	0	1
14003	GO:0072193	1	0.993983031	0	3
14004	GO:0072197	1	0.990010079	0	5
14005	GO:0072198	1	0.997974922	0	1
14006	GO:0072200	1	0.997974922	0	1
14007	GO:0072201	1	0.98801691	0	6
14008	GO:0072202	1	0.998013255	0	1
14009	GO:0072203	1	0.996000747	0	2
14010	GO:0072205	1	0.982098244	0	9
14011	GO:0072207	1	0.994001446	0	3
14012	GO:0072208	1	0.995993707	0	2
14013	GO:0072210	1	0.994014599	0	3
14014	GO:0072213	1	0.997982868	0	1
14015	GO:0072214	1	0.998013004	0	1
14016	GO:0072218	1	0.996030299	0	2
14017	GO:0072219	1	0.998013004	0	1
14018	GO:0072221	1	0.995997368	0	2
14019	GO:0072224	1	0.996000375	0	2
14020	GO:0072229	1	0.997980886	0	1
14021	GO:0072234	1	0.997976774	0	1
14022	GO:0072235	1	0.998013004	0	1
14023	GO:0072236	1	0.998006669	0	1
14024	GO:0072237	1	0.998013437	0	1
14025	GO:0072239	1	0.997976774	0	1
14026	GO:0072249	1	0.998013437	0	1
14027	GO:0072254	1	0.997989991	0	1
14028	GO:0072255	1	0.9979925	0	1
14029	GO:0072259	1	0.997976774	0	1
14030	GO:0072262	1	0.998013436	0	1
14031	GO:0072267	1	0.997982868	0	1
14032	GO:0072268	1	0.99596363	0	2
14033	GO:0072272	1	0.997989387	0	1
14034	GO:0072273	1	0.998006585	0	1
14035	GO:0072274	1	0.998013437	0	1
14036	GO:0072275	1	0.998013436	0	1
14037	GO:0072277	1	0.99603073	0	2
14038	GO:0072278	1	0.994014592	0	3
14039	GO:0072282	1	0.993960324	0	3
14040	GO:0072283	1	0.997983203	0	1
14041	GO:0072284	1	0.99203922	0	4
14042	GO:0072285	1	0.998004776	0	1
14043	GO:0072286	1	0.997987203	0	1
14044	GO:0072287	1	0.998013437	0	1
14045	GO:0072289	1	0.994019815	0	3
14046	GO:0072300	1	0.998012663	0	1

14047	GO:0072303	1	0.9979964	0	1
14048	GO:0072305	1	0.998006243	0	1
14049	GO:0072307	1	0.990091258	0	5
14051	GO:0072318	1	0.994015743	0	3
14052	GO:0072319	1	0.997981567	0	1
14053	GO:0072320	1	0.992022105	0	4
14054	GO:0072321	1	0.987928634	0	6
14055	GO:0072331	1	0.995973895	0	2
14056	GO:0072332	1	0.954715767	0	23
14057	GO:0072334	1	0.991918498	0	4
14058	GO:0072341	1	0.990055096	0	5
14059	GO:0072344	1	0.987990902	0	6
14060	GO:0072345	1	0.990032204	0	5
14061	GO:0072347	1	0.998004621	0	1
14062	GO:0072350	1	0.993959755	0	3
14063	GO:0072354	1	0.995968189	0	2
14064	GO:0072355	1	0.997973646	0	1
14065	GO:0072356	1	0.998007827	0	1
14066	GO:0072357	1	0.986096072	0	7
14067	GO:0072359	1	0.970385353	0	15
14068	GO:0072378	1	0.993990968	0	3
14069	GO:0072380	1	0.996001124	0	2
14070	GO:0072382	1	0.998013437	0	1
14071	GO:0072383	1	0.988119603	0	6
14072	GO:0072384	1	0.988022351	0	6
14073	GO:0072385	1	0.992035579	0	4
14074	GO:0072386	1	0.998013437	0	1
14075	GO:0072389	1	0.998006243	0	1
14076	GO:0072393	1	0.996018162	0	2
14077	GO:0072394	1	0.997972608	0	1
14078	GO:0072413	1	0.995994612	0	2
14079	GO:0072421	1	0.997972608	0	1
14080	GO:0072422	1	0.993963314	0	3
14081	GO:0072423	1	0.996014102	0	2
14082	GO:0072425	1	0.97817676	0	11
14083	GO:0072428	1	0.997976383	0	1
14084	GO:0072429	1	0.991974129	0	4
14085	GO:0072431	1	0.998013437	0	1
14086	GO:0072432	1	0.998013437	0	1
14087	GO:0072434	1	0.998013437	0	1
14088	GO:0072487	1	0.992013509	0	4
14089	GO:0072488	1	0.985975108	0	7
14090	GO:0072497	1	0.997968853	0	1
14091	GO:0072498	1	0.987965945	0	6
14092	GO:0072507	1	0.998013437	0	1

14093	GO:0072513	1	0.996000254	0	2
14094	GO:0072518	1	0.996030731	0	2
14095	GO:0072520	1	0.97621189	0	12
14096	GO:0072530	1	0.995993485	0	2
14097	GO:0072531	1	0.987954365	0	6
14098	GO:0072534	1	0.998013437	0	1
14099	GO:0072536	1	0.993980165	0	3
14100	GO:0072537	1	0.993991833	0	3
14101	GO:0072538	1	0.995992447	0	2
14102	GO:0072539	1	0.99200454	0	4
14103	GO:0072540	1	0.986008096	0	7
14104	GO:0072542	1	0.978130494	0	11
14105	GO:0072544	1	0.997973451	0	1
14106	GO:0072545	1	0.995958661	0	2
14107	GO:0072546	1	0.980095136	0	10
14108	GO:0072553	1	0.993997252	0	3
14109	GO:0072554	1	0.995988492	0	2
14110	GO:0072555	1	0.997971648	0	1
14111	GO:0072557	1	0.993953799	0	3
14112	GO:0072558	1	0.991921922	0	4
14113	GO:0072559	1	0.984020828	0	8
14114	GO:0072560	1	0.995962824	0	2
14115	GO:0072562	1	0.845580376	0	83
14116	GO:0072563	1	0.997996453	0	1
14117	GO:0072570	1	0.997998019	0	1
14118	GO:0072572	1	0.995975432	0	2
14119	GO:0072573	1	0.998010813	0	1
14120	GO:0072574	1	0.988014138	0	6
14121	GO:0072576	1	0.996030731	0	2
14122	GO:0072577	1	0.98609142	0	7
14123	GO:0072579	1	0.99800978	0	1
14124	GO:0072582	1	0.971951748	0	14
14125	GO:0072583	1	0.951164254	0	25
14126	GO:0072584	1	0.989995353	0	5
14127	GO:0072587	1	0.998011217	0	1
14128	GO:0072589	1	0.991882148	0	4
14129	GO:0072590	1	0.996030731	0	2
14130	GO:0072591	1	0.998013437	0	1
14131	GO:0072592	1	0.993993628	0	3
14134	GO:0072599	1	0.998013437	0	1
14135	GO:0072655	1	0.987973855	0	6
14136	GO:0072656	1	0.989995446	0	5
14137	GO:0072657	1	0.956762754	0	22
14138	GO:0072659	1	0.745350957	0	146
14139	GO:0072660	1	0.996030731	0	2

14140	GO:0072663	1	0.998003224	0	1
14142	GO:0072666	1	0.998010813	0	1
14143	GO:0072669	1	0.983918223	0	8
14144	GO:0072672	1	0.99000792	0	5
14145	GO:0072673	1	0.988032417	0	6
14146	GO:0072674	1	0.993988759	0	3
14147	GO:0072675	1	0.990029132	0	5
14148	GO:0072676	1	0.99201388	0	4
14149	GO:0072678	1	0.986009156	0	7
14150	GO:0072679	1	0.995960566	0	2
14151	GO:0072683	1	0.993967215	0	3
14152	GO:0072684	1	0.998005669	0	1
14153	GO:0072686	1	0.794995365	0	114
14154	GO:0072687	1	0.974143098	0	13
14155	GO:0072697	1	0.995965412	0	2
14156	GO:0072703	1	0.998013429	0	1
14157	GO:0072707	1	0.997987709	0	1
14158	GO:0072708	1	0.995983923	0	2
14159	GO:0072709	1	0.993969939	0	3
14160	GO:0072710	1	0.997990564	0	1
14161	GO:0072711	1	0.978171497	0	11
14162	GO:0072715	1	0.997974172	0	1
14163	GO:0072716	1	0.997990564	0	1
14164	GO:0072717	1	0.99396507	0	3
14165	GO:0072718	1	0.994012343	0	3
14166	GO:0072719	1	0.988035055	0	6
14167	GO:0072720	1	0.997990564	0	1
14168	GO:0072721	1	0.997990564	0	1
14169	GO:0072734	1	0.989959969	0	5
14170	GO:0072738	1	0.995973077	0	2
14171	GO:0072739	1	0.997990564	0	1
14172	GO:0072740	1	0.997976824	0	1
14173	GO:0072749	1	0.997975371	0	1
14174	GO:0072750	1	0.995927874	0	2
14175	GO:0072752	1	0.996006689	0	2
14176	GO:0072755	1	0.998005013	0	1
14177	GO:0072757	1	0.992012213	0	4
14178	GO:0075044	1	0.990049175	0	5
14179	GO:0075506	1	0.995995716	0	2
14180	GO:0075521	1	0.997954136	0	1
14181	GO:0075522	1	0.978142356	0	11
14182	GO:0075523	1	0.997990564	0	1
14183	GO:0075525	1	0.989966767	0	5
14184	GO:0075606	1	0.997954136	0	1
14185	GO:0075713	1	0.983997233	0	8

14186	GO:0075732	1	0.996028401	0	2
14187	GO:0075733	1	0.904371606	0	50
14188	GO:0080008	1	0.95305235	0	24
14189	GO:0080009	1	0.970284853	0	15
14190	GO:0080019	1	0.995997464	0	2
14191	GO:0080025	1	0.951040258	0	25
14192	GO:0080030	1	0.994003883	0	3
14193	GO:0080048	1	0.997990564	0	1
14194	GO:0080058	1	0.997967253	0	1
14195	GO:0080090	1	0.996002372	0	2
14196	GO:0080101	1	0.997960141	0	1
14197	GO:0080111	1	0.964324964	0	18
14198	GO:0080121	1	0.995983296	0	2
14199	GO:0080122	1	0.995983296	0	2
14200	GO:0080129	1	0.997962094	0	1
14201	GO:0080132	1	0.997985482	0	1
14202	GO:0080135	1	0.990022407	0	5
14203	GO:0080144	1	0.997990564	0	1
14204	GO:0080146	1	0.997974251	0	1
14205	GO:0080154	1	0.991976253	0	4
14206	GO:0080163	1	0.997984954	0	1
14207	GO:0080164	1	0.997990564	0	1
14208	GO:0080170	1	0.99796877	0	1
14210	GO:0080184	1	0.997983585	0	1
14211	GO:0085017	1	0.998013437	0	1
14212	GO:0085018	1	0.997990556	0	1
14213	GO:0085020	1	0.981977351	0	9
14214	GO:0085029	1	0.982084464	0	9
14215	GO:0085032	1	0.997998569	0	1
14216	GO:0086001	1	0.994047999	0	3
14217	GO:0086002	1	0.97243901	0	14
14218	GO:0086003	1	0.992038533	0	4
14219	GO:0086004	1	0.980232689	0	10
14220	GO:0086005	1	0.970436935	0	15
14221	GO:0086006	1	0.990055029	0	5
14222	GO:0086007	1	0.992065385	0	4
14223	GO:0086008	1	0.986052561	0	7
14224	GO:0086009	1	0.980113046	0	10
14225	GO:0086010	1	0.974420147	0	13
14226	GO:0086011	1	0.984084854	0	8
14227	GO:0086012	1	0.976351371	0	12
14228	GO:0086013	1	0.980104154	0	10
14229	GO:0086014	1	0.984155556	0	8
14230	GO:0086015	1	0.99207686	0	4
14231	GO:0086016	1	0.996028135	0	2

14232	GO:0086019	1	0.996021711	0	2
14233	GO:0086020	1	0.996014185	0	2
14234	GO:0086021	1	0.996014185	0	2
14235	GO:0086023	1	0.998013437	0	1
14236	GO:0086024	1	0.998013437	0	1
14237	GO:0086029	1	0.996007137	0	2
14238	GO:0086030	1	0.997979332	0	1
14239	GO:0086036	1	0.990012186	0	5
14240	GO:0086038	1	0.997990564	0	1
14241	GO:0086039	1	0.998013437	0	1
14242	GO:0086040	1	0.998011551	0	1
14243	GO:0086041	1	0.998013437	0	1
14244	GO:0086042	1	0.998013229	0	1
14245	GO:0086043	1	0.998013437	0	1
14246	GO:0086044	1	0.997996859	0	1
14247	GO:0086045	1	0.992067529	0	4
14248	GO:0086046	1	0.99207686	0	4
14249	GO:0086047	1	0.993999157	0	3
14250	GO:0086048	1	0.994039366	0	3
14251	GO:0086053	1	0.992004231	0	4
14252	GO:0086054	1	0.997996859	0	1
14253	GO:0086055	1	0.997996859	0	1
14254	GO:0086056	1	0.996026554	0	2
14255	GO:0086057	1	0.99800608	0	1
14256	GO:0086059	1	0.998013437	0	1
14257	GO:0086060	1	0.998013437	0	1
14258	GO:0086061	1	0.998013437	0	1
14259	GO:0086062	1	0.995983102	0	2
14260	GO:0086063	1	0.998013437	0	1
14261	GO:0086064	1	0.97234938	0	14
14262	GO:0086066	1	0.998013437	0	1
14263	GO:0086067	1	0.996003534	0	2
14264	GO:0086069	1	0.997996746	0	1
14265	GO:0086070	1	0.998013437	0	1
14266	GO:0086072	1	0.997986188	0	1
14267	GO:0086073	1	0.988106254	0	6
14268	GO:0086075	1	0.995997075	0	2
14269	GO:0086076	1	0.997996859	0	1
14270	GO:0086077	1	0.994029837	0	3
14271	GO:0086078	1	0.997996859	0	1
14272	GO:0086079	1	0.997996859	0	1
14273	GO:0086080	1	0.990028832	0	5
14274	GO:0086082	1	0.997986188	0	1
14275	GO:0086083	1	0.990089098	0	5
14276	GO:0086089	1	0.993993691	0	3

14277	GO:0086091	1	0.930457701	0	36
14278	GO:0086092	1	0.998011551	0	1
14279	GO:0086098	1	0.996001288	0	2
14280	GO:0086100	1	0.986096538	0	7
14281	GO:0089700	1	0.994027611	0	3
14282	GO:0089701	1	0.989911797	0	5
14283	GO:0089708	1	0.99799116	0	1
14284	GO:0089709	1	0.991963938	0	4
14285	GO:0089717	1	0.982228376	0	9
14286	GO:0089718	1	0.976321596	0	12
14287	GO:0089720	1	0.983956683	0	8
14288	GO:0090009	1	0.986072094	0	7
14289	GO:0090022	1	0.991987285	0	4
14290	GO:0090023	1	0.958461913	0	21
14291	GO:0090024	1	0.994021846	0	3
14292	GO:0090025	1	0.997995361	0	1
14294	GO:0090027	1	0.990009555	0	5
14295	GO:0090031	1	0.997986766	0	1
14296	GO:0090035	1	0.997971738	0	1
14297	GO:0090036	1	0.992055997	0	4
14298	GO:0090037	1	0.980112079	0	10
14299	GO:0090038	1	0.992052853	0	4
14300	GO:0090042	1	0.994011543	0	3
14301	GO:0090043	1	0.991979287	0	4
14302	GO:0090044	1	0.997971738	0	1
14303	GO:0090045	1	0.995944134	0	2
14304	GO:0090049	1	0.994028151	0	3
14305	GO:0090050	1	0.958610703	0	21
14306	GO:0090051	1	0.970356051	0	15
14307	GO:0090063	1	0.984136642	0	8
14308	GO:0090065	1	0.997968918	0	1
14309	GO:0090067	1	0.998011379	0	1
14310	GO:0090068	1	0.994037541	0	3
14311	GO:0090071	1	0.996007881	0	2
14312	GO:0090074	1	0.998013437	0	1
14313	GO:0090075	1	0.997995931	0	1
14314	GO:0090076	1	0.996019352	0	2
14315	GO:0090080	1	0.99603019	0	2
14316	GO:0090081	1	0.997968004	0	1
14317	GO:0090082	1	0.991953273	0	4
14318	GO:0090083	1	0.996019079	0	2
14319	GO:0090084	1	0.982043188	0	9
14320	GO:0090085	1	0.998013437	0	1
14321	GO:0090086	1	0.995968895	0	2
14322	GO:0090090	1	0.713562849	0	167

14323	GO:0090091	1	0.984060043	0	8
14324	GO:0090094	1	0.997976774	0	1
14325	GO:0090096	1	0.997984457	0	1
14326	GO:0090101	1	0.997986797	0	1
14327	GO:0090102	1	0.954976442	0	23
14328	GO:0090103	1	0.962567426	0	19
14329	GO:0090107	1	0.995984542	0	2
14330	GO:0090108	1	0.995997135	0	2
14331	GO:0090110	1	0.972408818	0	14
14332	GO:0090114	1	0.990031723	0	5
14333	GO:0090116	1	0.992044266	0	4
14334	GO:0090117	1	0.997994144	0	1
14335	GO:0090118	1	0.994031754	0	3
14336	GO:0090119	1	0.996021086	0	2
14337	GO:0090122	1	0.997978869	0	1
14338	GO:0090126	1	0.998013437	0	1
14339	GO:0090128	1	0.994008874	0	3
14340	GO:0090129	1	0.984099957	0	8
14341	GO:0090131	1	0.993919908	0	3
14342	GO:0090132	1	0.998013236	0	1
14343	GO:0090133	1	0.997990564	0	1
14344	GO:0090134	1	0.995955728	0	2
14345	GO:0090135	1	0.993983762	0	3
14346	GO:0090136	1	0.974344621	0	13
14347	GO:0090138	1	0.998003887	0	1
14348	GO:0090140	1	0.982169887	0	9
14349	GO:0090141	1	0.966394666	0	17
14350	GO:0090144	1	0.99795383	0	1
14351	GO:0090148	1	0.990057081	0	5
14352	GO:0090149	1	0.995999233	0	2
14353	GO:0090150	1	0.974230455	0	13
14354	GO:0090151	1	0.995996346	0	2
14355	GO:0090152	1	0.998007629	0	1
14356	GO:0090153	1	0.997980382	0	1
14357	GO:0090155	1	0.993972719	0	3
14358	GO:0090156	1	0.98996655	0	5
14359	GO:0090158	1	0.989951954	0	5
14360	GO:0090160	1	0.978170437	0	11
14361	GO:0090161	1	0.974371173	0	13
14362	GO:0090162	1	0.976289394	0	12
14363	GO:0090164	1	0.996006689	0	2
14364	GO:0090166	1	0.986004292	0	7
14365	GO:0090168	1	0.991978615	0	4
14366	GO:0090169	1	0.996010891	0	2
14367	GO:0090170	1	0.991966738	0	4

14368	GO:0090172	1	0.997990564	0	1
14369	GO:0090175	1	0.990023106	0	5
14370	GO:0090176	1	0.994022789	0	3
14371	GO:0090177	1	0.997964525	0	1
14372	GO:0090178	1	0.998011696	0	1
14373	GO:0090179	1	0.980169304	0	10
14374	GO:0090181	1	0.976103494	0	12
14375	GO:0090184	1	0.991983335	0	4
14376	GO:0090187	1	0.997979776	0	1
14377	GO:0090188	1	0.992015721	0	4
14378	GO:0090189	1	0.997981065	0	1
14379	GO:0090190	1	0.966394285	0	17
14380	GO:0090191	1	0.995958614	0	2
14381	GO:0090193	1	0.997976723	0	1
14382	GO:0090194	1	0.997974922	0	1
14383	GO:0090199	1	0.99797433	0	1
14384	GO:0090200	1	0.94491603	0	28
14385	GO:0090201	1	0.962410966	0	19
14386	GO:0090204	1	0.995975386	0	2
14387	GO:0090205	1	0.995976236	0	2
14388	GO:0090207	1	0.991951979	0	4
14389	GO:0090209	1	0.997963771	0	1
14390	GO:0090210	1	0.996005256	0	2
14391	GO:0090212	1	0.998008787	0	1
14392	GO:0090216	1	0.998003237	0	1
14393	GO:0090217	1	0.994027222	0	3
14394	GO:0090218	1	0.997976007	0	1
14395	GO:0090219	1	0.99799643	0	1
14396	GO:0090220	1	0.997990564	0	1
14397	GO:0090222	1	0.996030731	0	2
14398	GO:0090226	1	0.997956837	0	1
14399	GO:0090230	1	0.99801236	0	1
14400	GO:0090234	1	0.994009289	0	3
14401	GO:0090235	1	0.990074465	0	5
14402	GO:0090237	1	0.998012951	0	1
14403	GO:0090238	1	0.993981669	0	3
14404	GO:0090239	1	0.995995215	0	2
14405	GO:0090240	1	0.989911065	0	5
14406	GO:0090241	1	0.995972186	0	2
14407	GO:0090242	1	0.997998511	0	1
14408	GO:0090244	1	0.9920396	0	4
14409	GO:0090245	1	0.994031827	0	3
14410	GO:0090246	1	0.998010994	0	1
14411	GO:0090249	1	0.996005385	0	2
14412	GO:0090251	1	0.996006727	0	2

14413	GO:0090258	1	0.992000572	0	4
14414	GO:0090259	1	0.995992287	0	2
14415	GO:0090260	1	0.996024568	0	2
14416	GO:0090261	1	0.991912432	0	4
14417	GO:0090263	1	0.750823513	0	142
14418	GO:0090264	1	0.997971783	0	1
14419	GO:0090266	1	0.987988458	0	6
14420	GO:0090267	1	0.986008298	0	7
14421	GO:0090271	1	0.994049608	0	3
14422	GO:0090272	1	0.993921904	0	3
14423	GO:0090275	1	0.997990564	0	1
14424	GO:0090276	1	0.997979381	0	1
14425	GO:0090277	1	0.98997428	0	5
14426	GO:0090279	1	0.988097839	0	6
14427	GO:0090280	1	0.974218298	0	13
14428	GO:0090281	1	0.989942574	0	5
14429	GO:0090282	1	0.99799465	0	1
14430	GO:0090284	1	0.998013437	0	1
14431	GO:0090285	1	0.995969498	0	2
14432	GO:0090287	1	0.998013437	0	1
14433	GO:0090288	1	0.996030144	0	2
14434	GO:0090291	1	0.996023418	0	2
14435	GO:0090292	1	0.994036454	0	3
14436	GO:0090296	1	0.998003986	0	1
14437	GO:0090297	1	0.997965696	0	1
14438	GO:0090298	1	0.996016941	0	2
14439	GO:0090301	1	0.997972813	0	1
14440	GO:0090303	1	0.958708455	0	21
14441	GO:0090305	1	0.774247505	0	127
14442	GO:0090306	1	0.994025531	0	3
14443	GO:0090307	1	0.924707255	0	39
14444	GO:0090308	1	0.997968004	0	1
14445	GO:0090309	1	0.978242008	0	11
14446	GO:0090310	1	0.996008529	0	2
14447	GO:0090311	1	0.991996265	0	4
14448	GO:0090312	1	0.993969793	0	3
14449	GO:0090313	1	0.998004621	0	1
14450	GO:0090314	1	0.941368742	0	30
14451	GO:0090315	1	0.987979547	0	6
14452	GO:0090316	1	0.950993657	0	25
14453	GO:0090317	1	0.998011533	0	1
14454	GO:0090322	1	0.993922578	0	3
14455	GO:0090324	1	0.991988423	0	4
14456	GO:0090325	1	0.997989069	0	1
14457	GO:0090327	1	0.998009002	0	1

14458	GO:0090329	1	0.998011388	0	1
14459	GO:0090330	1	0.990018138	0	5
14460	GO:0090331	1	0.978187576	0	11
14461	GO:0090335	1	0.99005147	0	5
14462	GO:0090336	1	0.972360649	0	14
14463	GO:0090340	1	0.997979776	0	1
14464	GO:0090341	1	0.997970959	0	1
14465	GO:0090343	1	0.995988473	0	2
14466	GO:0090344	1	0.989997617	0	5
14467	GO:0090346	1	0.997986766	0	1
14468	GO:0090350	1	0.997971413	0	1
14469	GO:0090361	1	0.997979694	0	1
14470	GO:0090362	1	0.995995444	0	2
14471	GO:0090367	1	0.997981583	0	1
14472	GO:0090370	1	0.987968603	0	6
14473	GO:0090382	1	0.982075053	0	9
14474	GO:0090383	1	0.94877127	0	26
14475	GO:0090384	1	0.997977002	0	1
14476	GO:0090385	1	0.982022712	0	9
14477	GO:0090387	1	0.998010419	0	1
14478	GO:0090389	1	0.998013437	0	1
14479	GO:0090394	1	0.984128818	0	8
14480	GO:0090398	1	0.956700967	0	22
14481	GO:0090399	1	0.968371257	0	16
14482	GO:0090400	1	0.992000954	0	4
14483	GO:0090402	1	0.995973906	0	2
14484	GO:0090403	1	0.992010154	0	4
14485	GO:0090409	1	0.997983299	0	1
14486	GO:0090410	1	0.997983299	0	1
14487	GO:0090416	1	0.997987639	0	1
14488	GO:0090422	1	0.997988914	0	1
14489	GO:0090425	1	0.99799543	0	1
14490	GO:0090433	1	0.997993682	0	1
14491	GO:0090434	1	0.994013496	0	3
14492	GO:0090435	1	0.990014535	0	5
14493	GO:0090443	1	0.991997152	0	4
14494	GO:0090461	1	0.990065948	0	5
14495	GO:0090472	1	0.998009214	0	1
14496	GO:0090481	1	0.98995265	0	5
14497	GO:0090482	1	0.992022789	0	4
14498	GO:0090486	1	0.997990564	0	1
14499	GO:0090494	1	0.991982794	0	4
14500	GO:0090497	1	0.996010764	0	2
14501	GO:0090498	1	0.992013456	0	4
14502	GO:0090500	1	0.997998496	0	1

14504	GO:0090502	1	0.884269287	0	61
14505	GO:0090503	1	0.935828056	0	33
14506	GO:0090520	1	0.995993528	0	2
14507	GO:0090521	1	0.988087668	0	6
14508	GO:0090522	1	0.997992918	0	1
14509	GO:0090527	1	0.982103643	0	9
14510	GO:0090533	1	0.998011551	0	1
14511	GO:0090534	1	0.998013437	0	1
14512	GO:0090537	1	0.996025644	0	2
14513	GO:0090541	1	0.996004999	0	2
14514	GO:0090543	1	0.935910767	0	33
14515	GO:0090554	1	0.990079841	0	5
14516	GO:0090555	1	0.988131162	0	6
14517	GO:0090556	1	0.996030731	0	2
14518	GO:0090557	1	0.976311017	0	12
14519	GO:0090559	1	0.990054928	0	5
14520	GO:0090560	1	0.995967058	0	2
14521	GO:0090571	1	0.993995476	0	3
14522	GO:0090575	1	0.888049058	0	59
14523	GO:0090599	1	0.992058018	0	4
14524	GO:0090611	1	0.989982988	0	5
14525	GO:0090615	1	0.997977264	0	1
14526	GO:0090618	1	0.996029964	0	2
14527	GO:0090619	1	0.995985075	0	2
14528	GO:0090624	1	0.995985075	0	2
14529	GO:0090625	1	0.997990564	0	1
14530	GO:0090630	1	0.805164888	0	108
14531	GO:0090636	1	0.998013359	0	1
14532	GO:0090637	1	0.998013359	0	1
14533	GO:0090646	1	0.99200092	0	4
14534	GO:0090647	1	0.982080532	0	9
14535	GO:0090648	1	0.998013437	0	1
14536	GO:0090649	1	0.993935016	0	3
14537	GO:0090650	1	0.984080538	0	8
14538	GO:0090651	1	0.995963142	0	2
14539	GO:0090656	1	0.976250953	0	12
14540	GO:0090657	1	0.996003739	0	2
14541	GO:0090659	1	0.998013437	0	1
14542	GO:0090660	1	0.98204708	0	9
14543	GO:0090661	1	0.991882148	0	4
14544	GO:0090663	1	0.99796602	0	1
14545	GO:0090666	1	0.989951225	0	5
14546	GO:0090668	1	0.998013437	0	1
14547	GO:0090669	1	0.991984541	0	4
14548	GO:0090671	1	0.997976203	0	1

14549	GO:0090673	1	0.998013437	0	1
14550	GO:0090675	1	0.998009559	0	1
14551	GO:0090676	1	0.997993311	0	1
14552	GO:0090694	1	0.996007903	0	2
14553	GO:0090721	1	0.998013437	0	1
14554	GO:0090722	1	0.996030724	0	2
14555	GO:0090724	1	0.990009423	0	5
14556	GO:0090725	1	0.998006543	0	1
14557	GO:0090730	1	0.997985764	0	1
14558	GO:0090734	1	0.947290691	0	27
14559	GO:0090737	1	0.99600397	0	2
14560	GO:0093001	1	0.997997597	0	1
14561	GO:0095500	1	0.980050381	0	10
14562	GO:0097001	1	0.983968743	0	8
14563	GO:0097003	1	0.995991953	0	2
14564	GO:0097006	1	0.993945212	0	3
14565	GO:0097009	1	0.934085043	0	34
14566	GO:0097010	1	0.995998729	0	2
14567	GO:0097011	1	0.978097066	0	11
14568	GO:0097013	1	0.9939474	0	3
14569	GO:0097014	1	0.993997369	0	3
14570	GO:0097016	1	0.993992598	0	3
14571	GO:0097017	1	0.997990285	0	1
14572	GO:0097020	1	0.997999824	0	1
14573	GO:0097021	1	0.991996658	0	4
14574	GO:0097022	1	0.995998729	0	2
14575	GO:0097023	1	0.997990564	0	1
14576	GO:0097025	1	0.99201748	0	4
14577	GO:0097027	1	0.982026652	0	9
14578	GO:0097028	1	0.981981159	0	9
14579	GO:0097029	1	0.993939735	0	3
14580	GO:0097035	1	0.989943591	0	5
14581	GO:0097036	1	0.997968714	0	1
14582	GO:0097037	1	0.9939713	0	3
14583	GO:0097038	1	0.970387166	0	15
14584	GO:0097039	1	0.993979662	0	3
14585	GO:0097045	1	0.998012341	0	1
14586	GO:0097049	1	0.992004903	0	4
14587	GO:0097050	1	0.995991447	0	2
14588	GO:0097051	1	0.994043084	0	3
14589	GO:0097052	1	0.991995229	0	4
14590	GO:0097053	1	0.991960561	0	4
14591	GO:0097055	1	0.995976369	0	2
14592	GO:0097056	1	0.991981045	0	4
14593	GO:0097057	1	0.995946674	0	2

14594	GO:0097058	1	0.995954499	0	2
14595	GO:0097059	1	0.997975173	0	1
14596	GO:0097060	1	0.930354098	0	36
14597	GO:0097061	1	0.991943886	0	4
14598	GO:0097062	1	0.980163618	0	10
14599	GO:0097065	1	0.992053793	0	4
14600	GO:0097066	1	0.984004845	0	8
14601	GO:0097067	1	0.974313524	0	13
14602	GO:0097068	1	0.99800547	0	1
14603	GO:0097069	1	0.996004042	0	2
14604	GO:0097070	1	0.994007849	0	3
14605	GO:0097079	1	0.997997916	0	1
14606	GO:0097080	1	0.997997916	0	1
14607	GO:0097084	1	0.983965231	0	8
14608	GO:0097089	1	0.993947782	0	3
14609	GO:0097090	1	0.998013437	0	1
14610	GO:0097091	1	0.984114675	0	8
14611	GO:0097094	1	0.986098219	0	7
14612	GO:0097100	1	0.989971425	0	5
14613	GO:0097101	1	0.997984402	0	1
14614	GO:0097102	1	0.995989407	0	2
14615	GO:0097104	1	0.982221242	0	9
14616	GO:0097105	1	0.978298278	0	11
14617	GO:0097106	1	0.998005327	0	1
14618	GO:0097107	1	0.994051853	0	3
14619	GO:0097108	1	0.992056934	0	4
14620	GO:0097109	1	0.992032637	0	4
14621	GO:0097110	1	0.893602802	0	56
14622	GO:0097111	1	0.996030731	0	2
14623	GO:0097112	1	0.994029945	0	3
14624	GO:0097113	1	0.992019891	0	4
14625	GO:0097114	1	0.990055679	0	5
14626	GO:0097115	1	0.99801271	0	1
14627	GO:0097116	1	0.994000126	0	3
14628	GO:0097117	1	0.996030731	0	2
14629	GO:0097118	1	0.996027798	0	2
14630	GO:0097119	1	0.990094296	0	5
14631	GO:0097120	1	0.980275953	0	10
14632	GO:0097123	1	0.995963572	0	2
14633	GO:0097124	1	0.989949683	0	5
14634	GO:0097125	1	0.995958398	0	2
14635	GO:0097129	1	0.993959701	0	3
14636	GO:0097134	1	0.993961101	0	3
14637	GO:0097135	1	0.995976719	0	2
14638	GO:0097136	1	0.986018832	0	7

14639	GO:0097140	1	0.998012708	0	1
14640	GO:0097141	1	0.998012708	0	1
14641	GO:0097144	1	0.997956473	0	1
14642	GO:0097145	1	0.995942265	0	2
14643	GO:0097149	1	0.994019182	0	3
14644	GO:0097150	1	0.958874383	0	21
14645	GO:0097151	1	0.99010314	0	5
14646	GO:0097152	1	0.995987917	0	2
14647	GO:0097153	1	0.982011725	0	9
14648	GO:0097154	1	0.997981405	0	1
14649	GO:0097155	1	0.994048123	0	3
14650	GO:0097156	1	0.996026972	0	2
14651	GO:0097157	1	0.978158769	0	11
14652	GO:0097158	1	0.995954282	0	2
14653	GO:0097159	1	0.995975075	0	2
14654	GO:0097160	1	0.997948513	0	1
14655	GO:0097161	1	0.998013437	0	1
14656	GO:0097162	1	0.99799854	0	1
14657	GO:0097163	1	0.997970731	0	1
14658	GO:0097165	1	0.988032415	0	6
14659	GO:0097167	1	0.992002777	0	4
14660	GO:0097168	1	0.995943379	0	2
14661	GO:0097169	1	0.991880439	0	4
14662	GO:0097176	1	0.993943399	0	3
14663	GO:0097177	1	0.991921742	0	4
14664	GO:0097178	1	0.976209342	0	12
14665	GO:0097179	1	0.997961681	0	1
14666	GO:0097180	1	0.995932878	0	2
14667	GO:0097185	1	0.997990564	0	1
14668	GO:0097186	1	0.984055336	0	8
14669	GO:0097187	1	0.991997091	0	4
14670	GO:0097188	1	0.997987912	0	1
14673	GO:0097192	1	0.941433946	0	30
14675	GO:0097194	1	0.952750812	0	24
14676	GO:0097195	1	0.993984969	0	3
14677	GO:0097196	1	0.995972145	0	2
14678	GO:0097197	1	0.980102046	0	10
14679	GO:0097198	1	0.996030731	0	2
14680	GO:0097199	1	0.981988031	0	9
14681	GO:0097200	1	0.982009422	0	9
14682	GO:0097201	1	0.991984116	0	4
14683	GO:0097202	1	0.972185006	0	14
14684	GO:0097205	1	0.998012835	0	1
14685	GO:0097208	1	0.98997585	0	5
14686	GO:0097209	1	0.99198685	0	4

14687	GO:0097211	1	0.990046018	0	5
14688	GO:0097212	1	0.995995599	0	2
14689	GO:0097213	1	0.9979825	0	1
14690	GO:0097214	1	0.995938361	0	2
14691	GO:0097222	1	0.998011657	0	1
14692	GO:0097224	1	0.984012901	0	8
14693	GO:0097225	1	0.950849945	0	25
14694	GO:0097226	1	0.997983235	0	1
14695	GO:0097227	1	0.988025077	0	6
14696	GO:0097228	1	0.960583798	0	20
14697	GO:0097229	1	0.997990564	0	1
14698	GO:0097232	1	0.998013437	0	1
14700	GO:0097234	1	0.998013437	0	1
14701	GO:0097237	1	0.986013995	0	7
14702	GO:0097238	1	0.997968061	0	1
14703	GO:0097241	1	0.98998666	0	5
14704	GO:0097242	1	0.974362035	0	13
14705	GO:0097243	1	0.995945888	0	2
14706	GO:0097250	1	0.985853735	0	7
14707	GO:0097252	1	0.995995386	0	2
14708	GO:0097255	1	0.989917719	0	5
14709	GO:0097257	1	0.997965198	0	1
14710	GO:0097264	1	0.986060088	0	7
14711	GO:0097267	1	0.982046787	0	9
14712	GO:0097268	1	0.994019684	0	3
14713	GO:0097269	1	0.99597848	0	2
14714	GO:0097274	1	0.995993386	0	2
14715	GO:0097275	1	0.998013437	0	1
14716	GO:0097276	1	0.998013437	0	1
14717	GO:0097277	1	0.998013437	0	1
14718	GO:0097278	1	0.996008337	0	2
14719	GO:0097281	1	0.997972608	0	1
14720	GO:0097284	1	0.978161995	0	11
14721	GO:0097291	1	0.99797867	0	1
14722	GO:0097296	1	0.991997464	0	4
14723	GO:0097298	1	0.998006856	0	1
14724	GO:0097300	1	0.989940187	0	5
14725	GO:0097305	1	0.995992791	0	2
14726	GO:0097309	1	0.995985075	0	2
14727	GO:0097310	1	0.997990564	0	1
14728	GO:0097320	1	0.974224805	0	13
14729	GO:0097322	1	0.982100193	0	9
14730	GO:0097323	1	0.998009467	0	1
14731	GO:0097324	1	0.99603047	0	2
14732	GO:0097325	1	0.994007464	0	3

14733	GO:0097326	1	0.998013176	0	1
14734	GO:0097327	1	0.987980706	0	6
14735	GO:0097332	1	0.995967374	0	2
14736	GO:0097338	1	0.994034633	0	3
14737	GO:0097340	1	0.993988922	0	3
14738	GO:0097342	1	0.988012986	0	6
14739	GO:0097343	1	0.9980062	0	1
14740	GO:0097344	1	0.997970886	0	1
14741	GO:0097345	1	0.982007759	0	9
14742	GO:0097350	1	0.992007997	0	4
14743	GO:0097352	1	0.945080402	0	28
14744	GO:0097355	1	0.995966913	0	2
14745	GO:0097356	1	0.990006861	0	5
14746	GO:0097359	1	0.996030099	0	2
14747	GO:0097361	1	0.990023073	0	5
14748	GO:0097362	1	0.995994401	0	2
14749	GO:0097363	1	0.994006266	0	3
14750	GO:0097367	1	0.991973112	0	4
14751	GO:0097368	1	0.992011817	0	4
14752	GO:0097370	1	0.997990564	0	1
14753	GO:0097371	1	0.976085901	0	12
14754	GO:0097372	1	0.997973786	0	1
14755	GO:0097374	1	0.996003648	0	2
14756	GO:0097376	1	0.997968378	0	1
14757	GO:0097379	1	0.997983203	0	1
14758	GO:0097380	1	0.997983426	0	1
14759	GO:0097381	1	0.974178794	0	13
14760	GO:0097383	1	0.998013437	0	1
14761	GO:0097386	1	0.974317361	0	13
14762	GO:0097400	1	0.980059292	0	10
14763	GO:0097401	1	0.998007757	0	1
14764	GO:0097402	1	0.997986922	0	1
14765	GO:0097403	1	0.997994666	0	1
14766	GO:0097409	1	0.997977911	0	1
14767	GO:0097411	1	0.992065955	0	4
14768	GO:0097413	1	0.98998631	0	5
14769	GO:0097414	1	0.997977911	0	1
14770	GO:0097418	1	0.990046976	0	5
14771	GO:0097421	1	0.929855409	0	36
14772	GO:0097422	1	0.990013112	0	5
14773	GO:0097427	1	0.988039951	0	6
14774	GO:0097428	1	0.9759785	0	12
14775	GO:0097431	1	0.932182266	0	35
14776	GO:0097433	1	0.98801064	0	6
14777	GO:0097435	1	0.974195162	0	13

14778	GO:0097440	1	0.968530284	0	16
14779	GO:0097441	1	0.988041563	0	6
14780	GO:0097442	1	0.998012928	0	1
14781	GO:0097443	1	0.988083474	0	6
14782	GO:0097444	1	0.993976037	0	3
14783	GO:0097447	1	0.993964985	0	3
14784	GO:0097449	1	0.976195049	0	12
14785	GO:0097450	1	0.99000289	0	5
14786	GO:0097451	1	0.994017981	0	3
14787	GO:0097452	1	0.99195698	0	4
14788	GO:0097454	1	0.993989801	0	3
14789	GO:0097457	1	0.994017068	0	3
14790	GO:0097462	1	0.997977911	0	1
14791	GO:0097466	1	0.995994611	0	2
14792	GO:0097468	1	0.997990564	0	1
14793	GO:0097470	1	0.992063947	0	4
14794	GO:0097472	1	0.993943015	0	3
14795	GO:0097473	1	0.998013267	0	1
14796	GO:0097474	1	0.998013437	0	1
14797	GO:0097475	1	0.995992287	0	2
14798	GO:0097477	1	0.99399648	0	3
14799	GO:0097484	1	0.980151945	0	10
14800	GO:0097485	1	0.98809132	0	6
14801	GO:0097486	1	0.991927564	0	4
14802	GO:0097487	1	0.993968423	0	3
14803	GO:0097489	1	0.997987593	0	1
14804	GO:0097490	1	0.992038585	0	4
14805	GO:0097491	1	0.992038585	0	4
14806	GO:0097493	1	0.996030731	0	2
14807	GO:0097494	1	0.988022675	0	6
14808	GO:0097497	1	0.997996769	0	1
14809	GO:0097498	1	0.995994611	0	2
14810	GO:0097499	1	0.998012708	0	1
14811	GO:0097500	1	0.992037032	0	4
14812	GO:0097501	1	0.99800725	0	1
14813	GO:0097502	1	0.972169892	0	14
14814	GO:0097503	1	0.964430273	0	18
14815	GO:0097504	1	0.976127362	0	12
14816	GO:0097505	1	0.998013436	0	1
14817	GO:0097510	1	0.997979455	0	1
14818	GO:0097512	1	0.987913936	0	6
14819	GO:0097513	1	0.994051875	0	3
14820	GO:0097519	1	0.998013437	0	1
14821	GO:0097524	1	0.989997051	0	5
14822	GO:0097525	1	0.997990386	0	1

14823	GO:0097526	1	0.987847006	0	6
14824	GO:0097527	1	0.985980236	0	7
14825	GO:0097528	1	0.995963171	0	2
14826	GO:0097530	1	0.998003887	0	1
14827	GO:0097531	1	0.998013071	0	1
14828	GO:0097533	1	0.995978415	0	2
14829	GO:0097535	1	0.996007045	0	2
14830	GO:0097536	1	0.99597416	0	2
14831	GO:0097539	1	0.982170856	0	9
14832	GO:0097541	1	0.997990564	0	1
14833	GO:0097542	1	0.90982633	0	47
14834	GO:0097543	1	0.996009641	0	2
14835	GO:0097545	1	0.995985075	0	2
14836	GO:0097546	1	0.939532037	0	31
14837	GO:0097550	1	0.980016061	0	10
14838	GO:0097552	1	0.997990564	0	1
14839	GO:0097553	1	0.988130811	0	6
14840	GO:0097573	1	0.997967253	0	1
14841	GO:0097575	1	0.996012347	0	2
14842	GO:0097577	1	0.997956756	0	1
14843	GO:0097581	1	0.993978401	0	3
14844	GO:0097598	1	0.996007826	0	2
14845	GO:0097602	1	0.952884775	0	24
14846	GO:0097603	1	0.998008787	0	1
14847	GO:0097621	1	0.993991818	0	3
14848	GO:0097623	1	0.976193696	0	12
14849	GO:0097629	1	0.994017467	0	3
14850	GO:0097631	1	0.997990564	0	1
14851	GO:0097632	1	0.996007708	0	2
14852	GO:0097635	1	0.996028987	0	2
14853	GO:0097637	1	0.996005505	0	2
14854	GO:0097638	1	0.994037239	0	3
14855	GO:0097643	1	0.993929579	0	3
14856	GO:0097647	1	0.989904668	0	5
14857	GO:0097649	1	0.997990564	0	1
14858	GO:0097655	1	0.997990564	0	1
14859	GO:0097657	1	0.997990564	0	1
14860	GO:0097676	1	0.994019701	0	3
14861	GO:0097677	1	0.984083602	0	8
14862	GO:0097680	1	0.988064316	0	6
14863	GO:0097681	1	0.992040982	0	4
14864	GO:0097682	1	0.997980349	0	1
14865	GO:0097692	1	0.984091326	0	8
14866	GO:0097694	1	0.996030731	0	2
14867	GO:0097695	1	0.996030731	0	2

14868	GO:0097698	1	0.997969696	0	1
14869	GO:0097699	1	0.998010978	0	1
14870	GO:0097708	1	0.974333855	0	13
14871	GO:0097711	1	0.826038121	0	95
14872	GO:0097712	1	0.997990564	0	1
14873	GO:0097718	1	0.93584787	0	33
14874	GO:0097720	1	0.990012439	0	5
14875	GO:0097726	1	0.997962652	0	1
14876	GO:0097728	1	0.996016472	0	2
14877	GO:0097729	1	0.976153035	0	12
14878	GO:0097730	1	0.947268092	0	27
14879	GO:0097731	1	0.978126028	0	11
14880	GO:0097733	1	0.988011002	0	6
14881	GO:0097734	1	0.99800761	0	1
14882	GO:0097744	1	0.998010863	0	1
14883	GO:0097745	1	0.993952377	0	3
14884	GO:0097746	1	0.966410334	0	17
14885	GO:0097749	1	0.996007539	0	2
14886	GO:0097750	1	0.996022947	0	2
14887	GO:0097752	1	0.993967614	0	3
14888	GO:0097753	1	0.995999571	0	2
14889	GO:0098038	1	0.998000962	0	1
14890	GO:0098505	1	0.980097116	0	10
14891	GO:0098506	1	0.997973945	0	1
14892	GO:0098507	1	0.995987421	0	2
14893	GO:0098508	1	0.995973592	0	2
14894	GO:0098519	1	0.993994064	0	3
14895	GO:0098528	1	0.998013437	0	1
14896	GO:0098530	1	0.998013437	0	1
14897	GO:0098531	1	0.996012553	0	2
14898	GO:0098532	1	0.990056141	0	5
14899	GO:0098534	1	0.98999852	0	5
14900	GO:0098535	1	0.990033754	0	5
14901	GO:0098536	1	0.990027128	0	5
14902	GO:0098542	1	0.997985088	0	1
14903	GO:0098547	1	0.997982676	0	1
14904	GO:0098554	1	0.99002362	0	5
14905	GO:0098556	1	0.987823704	0	6
14906	GO:0098559	1	0.993974898	0	3
14907	GO:0098560	1	0.995981884	0	2
14908	GO:0098562	1	0.995968307	0	2
14909	GO:0098574	1	0.987991398	0	6
14910	GO:0098575	1	0.995965795	0	2
14911	GO:0098576	1	0.998009097	0	1
14912	GO:0098577	1	0.998013437	0	1

14913	GO:0098582	1	0.998003586	0	1
14915	GO:0098591	1	0.990097955	0	5
14916	GO:0098592	1	0.992014088	0	4
14917	GO:0098594	1	0.997955033	0	1
14918	GO:0098595	1	0.997982628	0	1
14919	GO:0098599	1	0.995962776	0	2
14920	GO:0098609	1	0.707664754	0	172
14921	GO:0098629	1	0.995996176	0	2
14922	GO:0098632	1	0.960770828	0	20
14923	GO:0098633	1	0.995987482	0	2
14924	GO:0098636	1	0.993994618	0	3
14925	GO:0098639	1	0.990095624	0	5
14926	GO:0098640	1	0.995978953	0	2
14927	GO:0098641	1	0.96429302	0	18
14928	GO:0098655	1	0.863811278	0	73
14929	GO:0098656	1	0.89034461	0	58
14930	GO:0098657	1	0.995978683	0	2
14931	GO:0098658	1	0.996028495	0	2
14932	GO:0098659	1	0.99602295	0	2
14933	GO:0098662	1	0.976190017	0	12
14934	GO:0098664	1	0.974097877	0	13
14935	GO:0098666	1	0.99798234	0	1
14936	GO:0098674	1	0.996030731	0	2
14937	GO:0098680	1	0.99800762	0	1
14938	GO:0098684	1	0.99003611	0	5
14939	GO:0098685	1	0.855041212	0	78
14940	GO:0098686	1	0.935939833	0	33
14941	GO:0098688	1	0.980151382	0	10
14942	GO:0098690	1	0.989999955	0	5
14943	GO:0098691	1	0.986003493	0	7
14944	GO:0098693	1	0.982148787	0	9
14945	GO:0098694	1	0.997996716	0	1
14946	GO:0098695	1	0.998013437	0	1
14947	GO:0098696	1	0.984081107	0	8
14948	GO:0098698	1	0.994031994	0	3
14949	GO:0098700	1	0.994000306	0	3
14950	GO:0098703	1	0.972410616	0	14
14951	GO:0098705	1	0.996029609	0	2
14952	GO:0098706	1	0.996028201	0	2
14953	GO:0098708	1	0.990055289	0	5
14954	GO:0098711	1	0.994000817	0	3
14955	GO:0098712	1	0.984125414	0	8
14956	GO:0098713	1	0.994009977	0	3
14957	GO:0098718	1	0.99801003	0	1
14958	GO:0098719	1	0.962706581	0	19

14959	GO:0098725	1	0.997990564	0	1
14960	GO:0098734	1	0.99194996	0	4
14961	GO:0098735	1	0.990047668	0	5
14962	GO:0098736	1	0.998013437	0	1
14963	GO:0098739	1	0.994015837	0	3
14964	GO:0098742	1	0.932257278	0	35
14965	GO:0098743	1	0.997977835	0	1
14966	GO:0098746	1	0.99801243	0	1
14967	GO:0098749	1	0.994020344	0	3
14968	GO:0098752	1	0.997980063	0	1
14969	GO:0098753	1	0.997984188	0	1
14970	GO:0098759	1	0.9979964	0	1
14971	GO:0098761	1	0.974147311	0	13
14972	GO:0098770	1	0.996022063	0	2
14973	GO:0098773	1	0.992043459	0	4
14974	GO:0098779	1	0.987988728	0	6
14975	GO:0098780	1	0.987990666	0	6
14976	GO:0098781	1	0.998013335	0	1
14977	GO:0098787	1	0.998007771	0	1
14978	GO:0098789	1	0.974242615	0	13
14979	GO:0098792	1	0.992025635	0	4
14981	GO:0098794	1	0.788825149	0	118
14982	GO:0098796	1	0.994002778	0	3
14983	GO:0098797	1	0.978257862	0	11
14984	GO:0098799	1	0.998002011	0	1
14985	GO:0098800	1	0.995940968	0	2
14986	GO:0098808	1	0.995972438	0	2
14987	GO:0098809	1	0.995985075	0	2
14988	GO:0098810	1	0.986067247	0	7
14989	GO:0098813	1	0.997977517	0	1
14990	GO:0098814	1	0.995987246	0	2
14991	GO:0098815	1	0.984101145	0	8
14992	GO:0098821	1	0.988087197	0	6
14993	GO:0098826	1	0.989980423	0	5
14994	GO:0098829	1	0.998013437	0	1
14995	GO:0098830	1	0.998012465	0	1
14996	GO:0098831	1	0.976337939	0	12
14997	GO:0098833	1	0.998009559	0	1
14998	GO:0098835	1	0.989988508	0	5
14999	GO:0098837	1	0.98803319	0	6
15000	GO:0098838	1	0.996002563	0	2
15001	GO:0098839	1	0.932271065	0	35
15002	GO:0098840	1	0.992014843	0	4
15003	GO:0098842	1	0.995971532	0	2
15004	GO:0098843	1	0.995977357	0	2

15006	GO:0098845	1	0.991994129	0	4
15007	GO:0098847	1	0.99595945	0	2
15008	GO:0098849	1	0.997994159	0	1
15009	GO:0098850	1	0.991962461	0	4
15010	GO:0098855	1	0.994038826	0	3
15011	GO:0098856	1	0.993979675	0	3
15012	GO:0098857	1	0.996025105	0	2
15013	GO:0098858	1	0.998013437	0	1
15014	GO:0098868	1	0.988057937	0	6
15015	GO:0098869	1	0.866054072	0	71
15016	GO:0098871	1	0.990028148	0	5
15017	GO:0098872	1	0.998013437	0	1
15018	GO:0098875	1	0.998012194	0	1
15019	GO:0098877	1	0.998004621	0	1
15020	GO:0098880	1	0.996030731	0	2
15021	GO:0098882	1	0.990093289	0	5
15022	GO:0098883	1	0.989986879	0	5
15024	GO:0098885	1	0.989995229	0	5
15025	GO:0098886	1	0.99600758	0	2
15026	GO:0098887	1	0.98408088	0	8
15027	GO:0098888	1	0.996030731	0	2
15028	GO:0098890	1	0.994034717	0	3
15029	GO:0098891	1	0.998012409	0	1
15030	GO:0098892	1	0.997978353	0	1
15031	GO:0098894	1	0.996021026	0	2
15032	GO:0098900	1	0.993993305	0	3
15033	GO:0098901	1	0.992035604	0	4
15034	GO:0098902	1	0.998013437	0	1
15035	GO:0098903	1	0.99399698	0	3
15036	GO:0098904	1	0.994008217	0	3
15037	GO:0098905	1	0.995957695	0	2
15038	GO:0098906	1	0.997996859	0	1
15039	GO:0098907	1	0.994046694	0	3
15040	GO:0098908	1	0.996013093	0	2
15041	GO:0098909	1	0.986059664	0	7
15042	GO:0098910	1	0.99403536	0	3
15043	GO:0098911	1	0.97829769	0	11
15044	GO:0098912	1	0.994047706	0	3
15045	GO:0098914	1	0.991983945	0	4
15046	GO:0098915	1	0.980112885	0	10
15047	GO:0098918	1	0.998013437	0	1
15048	GO:0098919	1	0.992073275	0	4
15049	GO:0098921	1	0.994028643	0	3
15050	GO:0098924	1	0.998013437	0	1
15051	GO:0098925	1	0.997990564	0	1

15052	GO:0098930	1	0.994030265	0	3
15053	GO:0098937	1	0.997988758	0	1
15054	GO:0098939	1	0.996013282	0	2
15055	GO:0098942	1	0.994034117	0	3
15056	GO:0098943	1	0.991962256	0	4
15057	GO:0098957	1	0.990076362	0	5
15058	GO:0098958	1	0.994002731	0	3
15059	GO:0098962	1	0.968467872	0	16
15060	GO:0098963	1	0.994009028	0	3
15061	GO:0098966	1	0.996030731	0	2
15062	GO:0098967	1	0.989990894	0	5
15063	GO:0098968	1	0.998013208	0	1
15064	GO:0098969	1	0.994013629	0	3
15065	GO:0098970	1	0.988014669	0	6
15066	GO:0098971	1	0.992062121	0	4
15067	GO:0098972	1	0.998013437	0	1
15068	GO:0098973	1	0.991965323	0	4
15069	GO:0098974	1	0.976197885	0	12
15070	GO:0098976	1	0.982193798	0	9
15071	GO:0098977	1	0.998013282	0	1
15072	GO:0098978	1	0.530487714	0	314
15073	GO:0098981	1	0.984093302	0	8
15074	GO:0098982	1	0.890229831	0	58
15075	GO:0098983	1	0.992068484	0	4
15076	GO:0098984	1	0.996005853	0	2
15077	GO:0098985	1	0.990098192	0	5
15078	GO:0098989	1	0.995987359	0	2
15079	GO:0098992	1	0.986075885	0	7
15080	GO:0098993	1	0.97998111	0	10
15081	GO:0098998	1	0.997996723	0	1
15082	GO:0098999	1	0.998009244	0	1
15083	GO:0099003	1	0.976232846	0	12
15084	GO:0099004	1	0.997951202	0	1
15085	GO:0099010	1	0.995996512	0	2
15086	GO:0099011	1	0.996030731	0	2
15087	GO:0099012	1	0.996030647	0	2
15088	GO:0099013	1	0.998013437	0	1
15089	GO:0099020	1	0.997991997	0	1
15090	GO:0099023	1	0.998013437	0	1
15091	GO:0099025	1	0.99601803	0	2
15092	GO:0099026	1	0.992042361	0	4
15093	GO:0099029	1	0.993993362	0	3
15094	GO:0099031	1	0.992020937	0	4
15095	GO:0099033	1	0.995985075	0	2
15096	GO:0099038	1	0.996029947	0	2

15097	GO:0099039	1	0.996030731	0	2
15098	GO:0099040	1	0.994045144	0	3
15099	GO:0099041	1	0.990055468	0	5
15100	GO:0099044	1	0.995967989	0	2
15101	GO:0099050	1	0.9979902	0	1
15102	GO:0099053	1	0.993995541	0	3
15103	GO:0099054	1	0.974343621	0	13
15104	GO:0099055	1	0.921033148	0	41
15105	GO:0099056	1	0.913672584	0	45
15106	GO:0099059	1	0.972336772	0	14
15107	GO:0099060	1	0.9606896	0	20
15108	GO:0099061	1	0.926696804	0	38
15109	GO:0099065	1	0.998013182	0	1
15110	GO:0099066	1	0.997975715	0	1
15111	GO:0099068	1	0.99599107	0	2
15112	GO:0099072	1	0.980135073	0	10
15113	GO:0099073	1	0.994012496	0	3
15114	GO:0099074	1	0.993990317	0	3
15115	GO:0099077	1	0.998013437	0	1
15116	GO:0099078	1	0.983954969	0	8
15117	GO:0099087	1	0.997992817	0	1
15118	GO:0099091	1	0.992004151	0	4
15119	GO:0099092	1	0.972335522	0	14
15120	GO:0099093	1	0.996007856	0	2
15121	GO:0099104	1	0.991983917	0	4
15122	GO:0099106	1	0.989850548	0	5
15123	GO:0099111	1	0.998013437	0	1
15124	GO:0099115	1	0.99598974	0	2
15125	GO:0099116	1	0.997966211	0	1
15126	GO:0099122	1	0.992049398	0	4
15127	GO:0099144	1	0.99800978	0	1
15128	GO:0099147	1	0.990104062	0	5
15129	GO:0099149	1	0.976227505	0	12
15130	GO:0099150	1	0.998013437	0	1
15131	GO:0099151	1	0.984143636	0	8
15132	GO:0099152	1	0.993989659	0	3
15133	GO:0099154	1	0.998011379	0	1
15134	GO:0099156	1	0.997999567	0	1
15135	GO:0099158	1	0.997996723	0	1
15136	GO:0099159	1	0.995973944	0	2
15137	GO:0099160	1	0.996011189	0	2
15138	GO:0099170	1	0.984064467	0	8
15139	GO:0099171	1	0.994026539	0	3
15140	GO:0099172	1	0.9959963	0	2
15141	GO:0099173	1	0.980179674	0	10

15142	GO:0099174	1	0.99801271	0	1
15143	GO:0099175	1	0.964437071	0	18
15144	GO:0099178	1	0.995970811	0	2
15145	GO:0099179	1	0.99402204	0	3
15146	GO:0099180	1	0.995961004	0	2
15147	GO:0099181	1	0.998008298	0	1
15148	GO:0099182	1	0.99800283	0	1
15149	GO:0099183	1	0.996013418	0	2
15150	GO:0099184	1	0.994016799	0	3
15151	GO:0099185	1	0.994016799	0	3
15152	GO:0099186	1	0.996022063	0	2
15153	GO:0099189	1	0.998013437	0	1
15154	GO:0099400	1	0.998013437	0	1
15155	GO:0099502	1	0.994018932	0	3
15156	GO:0099503	1	0.991966733	0	4
15157	GO:0099504	1	0.990043789	0	5
15158	GO:0099505	1	0.992045153	0	4
15159	GO:0099507	1	0.9960128	0	2
15160	GO:0099508	1	0.994037369	0	3
15161	GO:0099509	1	0.980135106	0	10
15162	GO:0099512	1	0.995966898	0	2
15163	GO:0099518	1	0.993938456	0	3
15164	GO:0099519	1	0.994046756	0	3
15165	GO:0099520	1	0.998002581	0	1
15166	GO:0099523	1	0.976308945	0	12
15167	GO:0099524	1	0.966481626	0	17
15168	GO:0099525	1	0.99207021	0	4
15169	GO:0099526	1	0.996030731	0	2
15170	GO:0099527	1	0.982105744	0	9
15171	GO:0099529	1	0.997991579	0	1
15172	GO:0099530	1	0.996030731	0	2
15173	GO:0099533	1	0.993997681	0	3
15174	GO:0099534	1	0.995959615	0	2
15175	GO:0099536	1	0.992046613	0	4
15176	GO:0099542	1	0.998013437	0	1
15177	GO:0099544	1	0.996021931	0	2
15178	GO:0099545	1	0.994020188	0	3
15179	GO:0099546	1	0.998013435	0	1
15180	GO:0099547	1	0.995995182	0	2
15181	GO:0099550	1	0.997981179	0	1
15182	GO:0099551	1	0.998013437	0	1
15183	GO:0099553	1	0.99404399	0	3
15184	GO:0099555	1	0.997990564	0	1
15185	GO:0099557	1	0.994005106	0	3
15186	GO:0099558	1	0.991950295	0	4

15187	GO:0099560	1	0.964656967	0	18
15188	GO:0099562	1	0.990095634	0	5
15189	GO:0099563	1	0.992018039	0	4
15190	GO:0099564	1	0.997998232	0	1
15191	GO:0099566	1	0.978303669	0	11
15192	GO:0099567	1	0.997987203	0	1
15193	GO:0099568	1	0.997993551	0	1
15194	GO:0099569	1	0.997990564	0	1
15195	GO:0099572	1	0.990062781	0	5
15196	GO:0099575	1	0.99596144	0	2
15197	GO:0099576	1	0.994047738	0	3
15198	GO:0099577	1	0.997979973	0	1
15199	GO:0099578	1	0.994004691	0	3
15200	GO:0099579	1	0.996006487	0	2
15201	GO:0099580	1	0.996030548	0	2
15202	GO:0099582	1	0.997991904	0	1
15203	GO:0099583	1	0.994051874	0	3
15204	GO:0099590	1	0.991963222	0	4
15205	GO:0099601	1	0.991966433	0	4
15206	GO:0099604	1	0.990027295	0	5
15207	GO:0099606	1	0.998013437	0	1
15208	GO:0099607	1	0.998013437	0	1
15209	GO:0099608	1	0.998013083	0	1
15210	GO:0099609	1	0.996030731	0	2
15211	GO:0099612	1	0.993981847	0	3
15212	GO:0099617	1	0.991996145	0	4
15213	GO:0099625	1	0.993986446	0	3
15214	GO:0099626	1	0.996007197	0	2
15215	GO:0099627	1	0.997990564	0	1
15216	GO:0099629	1	0.996007903	0	2
15217	GO:0099630	1	0.997990564	0	1
15218	GO:0099631	1	0.995930809	0	2
15219	GO:0099634	1	0.993972099	0	3
15220	GO:0099635	1	0.993997681	0	3
15221	GO:0099637	1	0.998013437	0	1
15222	GO:0099638	1	0.997982404	0	1
15223	GO:0099639	1	0.997998802	0	1
15224	GO:0099641	1	0.984107896	0	8
15225	GO:0099642	1	0.996030731	0	2
15226	GO:0099645	1	0.992027119	0	4
15227	GO:0099699	1	0.99399485	0	3
15228	GO:0099703	1	0.993997681	0	3
15229	GO:0099738	1	0.978157399	0	11
15230	GO:0100002	1	0.997992291	0	1
15231	GO:0100026	1	0.996022892	0	2

15232	GO:0101003	1	0.896984131	0	54
15233	GO:0101004	1	0.995985401	0	2
15234	GO:0101005	1	0.980139884	0	10
15235	GO:0101006	1	0.993940988	0	3
15236	GO:0101020	1	0.984011608	0	8
15237	GO:0101021	1	0.989990199	0	5
15238	GO:0101030	1	0.993955499	0	3
15239	GO:0101031	1	0.968095264	0	16
15240	GO:0102007	1	0.993922934	0	3
15241	GO:0102009	1	0.997976841	0	1
15242	GO:0102033	1	0.990021282	0	5
15243	GO:0102035	1	0.998013437	0	1
15244	GO:0102076	1	0.997992183	0	1
15245	GO:0102077	1	0.995963191	0	2
15246	GO:0102084	1	0.997982988	0	1
15247	GO:0102092	1	0.996030582	0	2
15248	GO:0102102	1	0.99800715	0	1
15249	GO:0102113	1	0.998013437	0	1
15250	GO:0102116	1	0.997973026	0	1
15251	GO:0102121	1	0.987971588	0	6
15252	GO:0102131	1	0.998013437	0	1
15253	GO:0102132	1	0.998013437	0	1
15254	GO:0102140	1	0.996030731	0	2
15255	GO:0102148	1	0.993965293	0	3
15256	GO:0102158	1	0.991985904	0	4
15257	GO:0102166	1	0.997990564	0	1
15258	GO:0102167	1	0.997990564	0	1
15259	GO:0102175	1	0.997971684	0	1
15260	GO:0102193	1	0.995948271	0	2
15261	GO:0102194	1	0.99796864	0	1
15262	GO:0102200	1	0.998013209	0	1
15263	GO:0102207	1	0.9959828	0	2
15264	GO:0102250	1	0.994013624	0	3
15265	GO:0102258	1	0.998005567	0	1
15266	GO:0102259	1	0.998005567	0	1
15267	GO:0102264	1	0.997990564	0	1
15268	GO:0102279	1	0.998012626	0	1
15269	GO:0102320	1	0.997990913	0	1
15270	GO:0102336	1	0.987988928	0	6
15271	GO:0102337	1	0.987988928	0	6
15272	GO:0102338	1	0.987988928	0	6
15273	GO:0102339	1	0.997987281	0	1
15274	GO:0102340	1	0.997987281	0	1
15275	GO:0102341	1	0.997987281	0	1
15276	GO:0102342	1	0.997987281	0	1

15277	GO:0102343	1	0.991985904	0	4
15278	GO:0102344	1	0.991985904	0	4
15279	GO:0102345	1	0.991985904	0	4
15280	GO:0102354	1	0.993947824	0	3
15281	GO:0102389	1	0.997989115	0	1
15282	GO:0102390	1	0.997988836	0	1
15283	GO:0102391	1	0.993970963	0	3
15284	GO:0102420	1	0.992009974	0	4
15285	GO:0102485	1	0.993997854	0	3
15286	GO:0102486	1	0.993997854	0	3
15287	GO:0102487	1	0.993997854	0	3
15288	GO:0102488	1	0.993997854	0	3
15289	GO:0102489	1	0.993997854	0	3
15290	GO:0102490	1	0.993997854	0	3
15291	GO:0102491	1	0.993997854	0	3
15292	GO:0102499	1	0.994013624	0	3
15293	GO:0102500	1	0.998013437	0	1
15294	GO:0102521	1	0.995988254	0	2
15295	GO:0102522	1	0.997982083	0	1
15296	GO:0102524	1	0.997990564	0	1
15297	GO:0102545	1	0.978159542	0	11
15298	GO:0102552	1	0.997973035	0	1
15299	GO:0102553	1	0.997973035	0	1
15300	GO:0102555	1	0.997959405	0	1
15301	GO:0102559	1	0.998013437	0	1
15302	GO:0102567	1	0.952783428	0	24
15303	GO:0102568	1	0.952783428	0	24
15304	GO:0102571	1	0.997990564	0	1
15305	GO:0102662	1	0.997981631	0	1
15306	GO:0102704	1	0.997992925	0	1
15307	GO:0102707	1	0.997987515	0	1
15308	GO:0102732	1	0.997975112	0	1
15309	GO:0102751	1	0.995978962	0	2
15310	GO:0102752	1	0.997996052	0	1
15311	GO:0102756	1	0.987988928	0	6
15312	GO:0102757	1	0.997990564	0	1
15313	GO:0102758	1	0.997963616	0	1
15314	GO:0102769	1	0.997972277	0	1
15315	GO:0102773	1	0.996008213	0	2
15316	GO:0102797	1	0.998012804	0	1
15317	GO:0102798	1	0.998012804	0	1
15318	GO:0102867	1	0.997990293	0	1
15319	GO:0102938	1	0.997982988	0	1
15320	GO:0102953	1	0.991971664	0	4
15321	GO:0102965	1	0.995997464	0	2

15322	GO:0102991	1	0.974052058	0	13
15323	GO:0103002	1	0.997973026	0	1
15324	GO:0103025	1	0.991933575	0	4
15325	GO:0103026	1	0.997990564	0	1
15326	GO:0103045	1	0.997979809	0	1
15327	GO:0103046	1	0.998012912	0	1
15328	GO:0103053	1	0.995951125	0	2
15329	GO:0103066	1	0.997971684	0	1
15330	GO:0103067	1	0.997971684	0	1
15331	GO:0103068	1	0.991971664	0	4
15332	GO:0103069	1	0.997979067	0	1
15333	GO:0103073	1	0.995963191	0	2
15334	GO:0106001	1	0.99600044	0	2
15335	GO:0106003	1	0.998013437	0	1
15336	GO:0106004	1	0.995944175	0	2
15337	GO:0106005	1	0.994006307	0	3
15338	GO:0106006	1	0.994035901	0	3
15339	GO:0106008	1	0.997965639	0	1
15340	GO:0106014	1	0.997987546	0	1
15341	GO:0106015	1	0.993969003	0	3
15342	GO:0106016	1	0.995945909	0	2
15343	GO:0106018	1	0.996006377	0	2
15344	GO:0106019	1	0.997999981	0	1
15345	GO:0106022	1	0.998006747	0	1
15346	GO:0106027	1	0.998013437	0	1
15347	GO:0106028	1	0.998012604	0	1
15348	GO:0106029	1	0.991943436	0	4
15349	GO:0106030	1	0.996003456	0	2
15350	GO:0106034	1	0.995956307	0	2
15351	GO:0106035	1	0.987946891	0	6
15352	GO:0106044	1	0.997958946	0	1
15353	GO:0106045	1	0.997958946	0	1
15354	GO:0106046	1	0.997958946	0	1
15355	GO:0106047	1	0.997989557	0	1
15356	GO:0106048	1	0.997989557	0	1
15357	GO:0106049	1	0.997977002	0	1
15358	GO:0106050	1	0.997990564	0	1
15359	GO:0106056	1	0.998013083	0	1
15360	GO:0106064	1	0.998013437	0	1
15361	GO:0106068	1	0.997953871	0	1
15362	GO:0106070	1	0.997982034	0	1
15363	GO:0106071	1	0.993988912	0	3
15364	GO:0106072	1	0.991966673	0	4
15365	GO:0106073	1	0.996013576	0	2
15366	GO:0106074	1	0.974205276	0	13

15367	GO:0106077	1	0.992024677	0	4
15368	GO:0106078	1	0.997996279	0	1
15369	GO:0106089	1	0.998013437	0	1
15370	GO:0106090	1	0.997941649	0	1
15371	GO:0106091	1	0.995943412	0	2
15372	GO:0106101	1	0.997975998	0	1
15373	GO:0106105	1	0.997990564	0	1
15374	GO:0106121	1	0.998013437	0	1
15375	GO:0106134	1	0.998002124	0	1
15376	GO:0106137	1	0.995995495	0	2
15377	GO:0106138	1	0.998013437	0	1
15378	GO:0106140	1	0.992035026	0	4
15379	GO:0106153	1	0.997963315	0	1
15380	GO:0106162	1	0.998007125	0	1
15381	GO:0106177	1	0.998013437	0	1
15382	GO:0106217	1	0.997982628	0	1
15383	GO:0106226	1	0.998013437	0	1
15384	GO:0106227	1	0.997996279	0	1
15385	GO:0106228	1	0.997996279	0	1
15386	GO:0106229	1	0.997996279	0	1
15387	GO:0106230	1	0.995983678	0	2
15388	GO:0106231	1	0.995983678	0	2
15389	GO:0106235	1	0.993983524	0	3
15390	GO:0106245	1	0.995976634	0	2
15391	GO:0106255	1	0.99597944	0	2
15392	GO:0106256	1	0.986061545	0	7
15393	GO:0106258	1	0.997986813	0	1
15394	GO:0106261	1	0.997997805	0	1
15395	GO:0106262	1	0.991988582	0	4
15396	GO:0106263	1	0.991988582	0	4
15397	GO:0106272	1	0.998008841	0	1
15398	GO:0106273	1	0.998008841	0	1
15399	GO:0106274	1	0.995942121	0	2
15400	GO:0106275	1	0.995942121	0	2
15401	GO:0106276	1	0.995923309	0	2
15402	GO:0106277	1	0.995923309	0	2
15403	GO:0106281	1	0.997959295	0	1
15404	GO:0106282	1	0.997959295	0	1
15405	GO:0106283	1	0.997959295	0	1
15406	GO:0106289	1	0.998004147	0	1
15407	GO:0106293	1	0.99603072	0	2
15408	GO:0106294	1	0.994025172	0	3
15409	GO:0106300	1	0.988018587	0	6
15410	GO:0106301	1	0.997976246	0	1
15411	GO:0106306	1	0.868491325	0	70

15412	GO:0106307	1	0.868491325	0	70
15413	GO:0106309	1	0.997979067	0	1
15416	GO:0106312	1	0.998013437	0	1
15417	GO:0106313	1	0.998013437	0	1
15418	GO:0106321	1	0.997988883	0	1
15419	GO:0106322	1	0.997988883	0	1
15420	GO:0106325	1	0.997982179	0	1
15421	GO:0106326	1	0.997982179	0	1
15422	GO:0106327	1	0.997986516	0	1
15423	GO:0106328	1	0.997986516	0	1
15424	GO:0106329	1	0.997979521	0	1
15425	GO:0106330	1	0.99799195	0	1
15426	GO:0106331	1	0.99799195	0	1
15427	GO:0106333	1	0.995936009	0	2
15428	GO:0106335	1	0.998008236	0	1
15429	GO:0106341	1	0.997984584	0	1
15430	GO:0106342	1	0.997984584	0	1
15431	GO:0106347	1	0.998003986	0	1
15432	GO:0106363	1	0.996001638	0	2
15433	GO:0110008	1	0.997995178	0	1
15434	GO:0110015	1	0.995887523	0	2
15435	GO:0110024	1	0.993996942	0	3
15436	GO:0110025	1	0.995991901	0	2
15437	GO:0110026	1	0.997995247	0	1
15438	GO:0110032	1	0.993997847	0	3
15439	GO:0110050	1	0.997967328	0	1
15440	GO:0110051	1	0.997990564	0	1
15441	GO:0110053	1	0.99400122	0	3
15442	GO:0110059	1	0.998013397	0	1
15443	GO:0110061	1	0.996030731	0	2
15444	GO:0110070	1	0.997977517	0	1
15445	GO:0110076	1	0.995961134	0	2
15446	GO:0110077	1	0.997993466	0	1
15447	GO:0110090	1	0.998012279	0	1
15448	GO:0110091	1	0.995999447	0	2
15449	GO:0110095	1	0.989904362	0	5
15450	GO:0110097	1	0.997983546	0	1
15451	GO:0110099	1	0.995971652	0	2
15452	GO:0110104	1	0.990070106	0	5
15453	GO:0110113	1	0.998011533	0	1
15454	GO:0110135	1	0.994016862	0	3
15455	GO:0110151	1	0.997982396	0	1
15456	GO:0110152	1	0.997990564	0	1
15457	GO:0110153	1	0.998001538	0	1
15458	GO:0110155	1	0.995996027	0	2

15459	GO:0120001	1	0.993913895	0	3
15461	GO:0120012	1	0.997990564	0	1
15462	GO:0120013	1	0.987975357	0	6
15463	GO:0120014	1	0.995985514	0	2
15464	GO:0120015	1	0.998013048	0	1
15465	GO:0120017	1	0.995967616	0	2
15466	GO:0120019	1	0.988019189	0	6
15468	GO:0120025	1	0.998013437	0	1
15469	GO:0120034	1	0.997969197	0	1
15470	GO:0120035	1	0.997961288	0	1
15471	GO:0120036	1	0.997999366	0	1
15472	GO:0120041	1	0.985941303	0	7
15473	GO:0120042	1	0.997988898	0	1
15474	GO:0120043	1	0.995970327	0	2
15475	GO:0120044	1	0.989997163	0	5
15476	GO:0120045	1	0.995980964	0	2
15477	GO:0120048	1	0.997990564	0	1
15478	GO:0120049	1	0.99599847	0	2
15479	GO:0120053	1	0.997990564	0	1
15480	GO:0120058	1	0.997954504	0	1
15481	GO:0120061	1	0.995928953	0	2
15482	GO:0120069	1	0.997956554	0	1
15483	GO:0120072	1	0.998013176	0	1
15484	GO:0120078	1	0.997985733	0	1
15485	GO:0120092	1	0.995970489	0	2
15486	GO:0120094	1	0.99799981	0	1
15487	GO:0120095	1	0.998000136	0	1
15488	GO:0120103	1	0.980176198	0	10
15489	GO:0120108	1	0.997978951	0	1
15490	GO:0120114	1	0.997970941	0	1
15491	GO:0120115	1	0.985840464	0	7
15492	GO:0120117	1	0.997998817	0	1
15493	GO:0120134	1	0.998013437	0	1
15494	GO:0120135	1	0.998013437	0	1
15495	GO:0120146	1	0.987925996	0	6
15496	GO:0120147	1	0.997980316	0	1
15497	GO:0120153	1	0.997966932	0	1
15498	GO:0120158	1	0.997990564	0	1
15499	GO:0120160	1	0.994003316	0	3
15500	GO:0120161	1	0.998003396	0	1
15502	GO:0120163	1	0.913533222	0	45
15503	GO:0120169	1	0.998012012	0	1
15504	GO:0120170	1	0.990027472	0	5
15505	GO:0120183	1	0.988110743	0	6
15506	GO:0120188	1	0.992000772	0	4

15507	GO:0120189	1	0.998012379	0	1
15508	GO:0120192	1	0.991960935	0	4
15509	GO:0120193	1	0.990018517	0	5
15510	GO:0120197	1	0.991952966	0	4
15511	GO:0120199	1	0.991983905	0	4
15512	GO:0120200	1	0.990006336	0	5
15513	GO:0120206	1	0.994009559	0	3
15514	GO:0120211	1	0.99799465	0	1
15515	GO:0120212	1	0.997990564	0	1
15516	GO:0120219	1	0.998013437	0	1
15517	GO:0120220	1	0.997977264	0	1
15518	GO:0120222	1	0.991999831	0	4
15519	GO:0120223	1	0.995978555	0	2
15520	GO:0120225	1	0.995996063	0	2
15521	GO:0120229	1	0.997971467	0	1
15522	GO:0120234	1	0.998013437	0	1
15523	GO:0120241	1	0.997990564	0	1
15524	GO:0120242	1	0.997990564	0	1
15525	GO:0120243	1	0.997990564	0	1
15526	GO:0120249	1	0.99798686	0	1
15527	GO:0120259	1	0.993996873	0	3
15528	GO:0120293	1	0.966254265	0	17
15529	GO:0120294	1	0.997992902	0	1
15530	GO:0120295	1	0.997992902	0	1
15531	GO:0120296	1	0.997992902	0	1
15532	GO:0120297	1	0.997992902	0	1
15533	GO:0120298	1	0.997992902	0	1
15534	GO:0120299	1	0.997992902	0	1
15535	GO:0120301	1	0.998013437	0	1
15536	GO:0140007	1	0.989966769	0	5
15537	GO:0140009	1	0.992019195	0	4
15538	GO:0140010	1	0.998004621	0	1
15539	GO:0140013	1	0.998013407	0	1
15540	GO:0140014	1	0.993996691	0	3
15541	GO:0140018	1	0.997990564	0	1
15542	GO:0140021	1	0.989940812	0	5
15543	GO:0140030	1	0.991989467	0	4
15544	GO:0140031	1	0.990033415	0	5
15545	GO:0140036	1	0.984059668	0	8
15546	GO:0140037	1	0.995985138	0	2
15547	GO:0140039	1	0.996011176	0	2
15548	GO:0140041	1	0.997958946	0	1
15549	GO:0140042	1	0.978039941	0	11
15550	GO:0140052	1	0.980146845	0	10
15551	GO:0140056	1	0.99402527	0	3

15552	GO:0140058	1	0.990097961	0	5
15553	GO:0140059	1	0.98799727	0	6
15554	GO:0140065	1	0.998013437	0	1
15555	GO:0140066	1	0.998013437	0	1
15556	GO:0140067	1	0.998013437	0	1
15557	GO:0140068	1	0.998013437	0	1
15558	GO:0140069	1	0.998013437	0	1
15559	GO:0140074	1	0.998013178	0	1
15560	GO:0140078	1	0.985910482	0	7
15561	GO:0140081	1	0.995958651	0	2
15562	GO:0140090	1	0.993963212	0	3
15563	GO:0140105	1	0.997980063	0	1
15564	GO:0140112	1	0.995983833	0	2
15565	GO:0140115	1	0.982198811	0	9
15566	GO:0140157	1	0.994050319	0	3
15567	GO:0140161	1	0.997998802	0	1
15568	GO:0140194	1	0.997967045	0	1
15569	GO:0140199	1	0.994016428	0	3
15570	GO:0140206	1	0.990032781	0	5
15571	GO:0140207	1	0.997996551	0	1
15572	GO:0140212	1	0.997990564	0	1
15573	GO:0140214	1	0.997962172	0	1
15574	GO:0140223	1	0.991929238	0	4
15575	GO:0140253	1	0.998009829	0	1
15576	GO:0140260	1	0.997990564	0	1
15577	GO:0140261	1	0.998013437	0	1
15578	GO:0140262	1	0.99199797	0	4
15579	GO:0140268	1	0.980172173	0	10
15580	GO:0140272	1	0.997982756	0	1
15581	GO:0140275	1	0.974092143	0	13
15582	GO:0140284	1	0.991998104	0	4
15583	GO:0140285	1	0.994021322	0	3
15584	GO:0140289	1	0.976244194	0	12
15585	GO:0140290	1	0.997990564	0	1
15586	GO:0140291	1	0.993979663	0	3
15587	GO:0140292	1	0.997990564	0	1
15588	GO:0140293	1	0.993979663	0	3
15589	GO:0140294	1	0.993980418	0	3
15590	GO:0140296	1	0.997963315	0	1
15591	GO:0140297	1	0.982087845	0	9
15592	GO:0140298	1	0.997992848	0	1
15593	GO:0140300	1	0.993989677	0	3
15594	GO:0140311	1	0.991995987	0	4
15595	GO:0140312	1	0.990071594	0	5
15596	GO:0140318	1	0.987999672	0	6

15597	GO:0140326	1	0.960974124	0	20
15598	GO:0140327	1	0.996030731	0	2
15599	GO:0140328	1	0.992056906	0	4
15600	GO:0140329	1	0.997981704	0	1
15601	GO:0140331	1	0.986169474	0	7
15602	GO:0140337	1	0.997973071	0	1
15603	GO:0140338	1	0.995970357	0	2
15604	GO:0140339	1	0.997973071	0	1
15605	GO:0140340	1	0.997973071	0	1
15606	GO:0140343	1	0.996021569	0	2
15607	GO:0140344	1	0.998008498	0	1
15608	GO:0140345	1	0.990096401	0	5
15609	GO:0140346	1	0.990105203	0	5
15610	GO:0140347	1	0.998013437	0	1
15611	GO:0140348	1	0.997981704	0	1
15612	GO:0140351	1	0.994051684	0	3
15613	GO:0140359	1	0.992057324	0	4
15614	GO:0140360	1	0.996006125	0	2
15615	GO:0140361	1	0.99204201	0	4
15617	GO:0140412	1	0.997997916	0	1
15618	GO:0140416	1	0.970108115	0	15
15619	GO:0140439	1	0.986077166	0	7
15620	GO:0140444	1	0.990067626	0	5
15621	GO:0140447	1	0.99596549	0	2
15622	GO:0140448	1	0.997961681	0	1
15623	GO:0140450	1	0.998013437	0	1
15624	GO:0140467	1	0.991984299	0	4
15625	GO:0140468	1	0.993973804	0	3
15626	GO:0140469	1	0.9960223	0	2
15627	GO:0140493	1	0.998013437	0	1
15628	GO:0140507	1	0.993934998	0	3
15629	GO:0140517	1	0.998010902	0	1
15630	GO:0140536	1	0.99799889	0	1
15631	GO:0140537	1	0.993996216	0	3
15632	GO:0140552	1	0.994019739	0	3
15633	GO:0140554	1	0.997990564	0	1
15634	GO:0140560	1	0.997990564	0	1
15635	GO:0140561	1	0.993983524	0	3
15636	GO:0140562	1	0.993983524	0	3
15637	GO:0140563	1	0.995988045	0	2
15638	GO:0140567	1	0.996005251	0	2
15639	GO:0140569	1	0.998006373	0	1
15640	GO:0140570	1	0.997994972	0	1
15641	GO:0140571	1	0.993976229	0	3
15642	GO:0140575	1	0.993979825	0	3

15643	GO:0140576	1	0.993979825	0	3
15644	GO:0140581	1	0.996030731	0	2
15645	GO:0140585	1	0.997969824	0	1
15646	GO:0140591	1	0.997990564	0	1
15647	GO:0140597	1	0.998003224	0	1
15648	GO:0150001	1	0.996030727	0	2
15649	GO:0150002	1	0.996021927	0	2
15650	GO:0150003	1	0.992010242	0	4
15651	GO:0150007	1	0.994007455	0	3
15652	GO:0150011	1	0.988062764	0	6
15653	GO:0150012	1	0.984158415	0	8
15654	GO:0150013	1	0.998013437	0	1
15655	GO:0150014	1	0.998013433	0	1
15656	GO:0150018	1	0.997986391	0	1
15657	GO:0150019	1	0.998012321	0	1
15658	GO:0150020	1	0.994023817	0	3
15659	GO:0150024	1	0.997979792	0	1
15660	GO:0150025	1	0.997979792	0	1
15661	GO:0150032	1	0.995973848	0	2
15662	GO:0150033	1	0.998013437	0	1
15663	GO:0150034	1	0.994022798	0	3
15664	GO:0150051	1	0.998013437	0	1
15665	GO:0150056	1	0.995965613	0	2
15666	GO:0150057	1	0.995963138	0	2
15667	GO:0150058	1	0.998003243	0	1
15668	GO:0150062	1	0.993998452	0	3
15669	GO:0150064	1	0.996029252	0	2
15670	GO:0150072	1	0.997982708	0	1
15671	GO:0150074	1	0.995977234	0	2
15672	GO:0150076	1	0.995978683	0	2
15673	GO:0150077	1	0.97806155	0	11
15674	GO:0150078	1	0.983885313	0	8
15675	GO:0150079	1	0.990002213	0	5
15676	GO:0150090	1	0.997997106	0	1
15677	GO:0150093	1	0.986030097	0	7
15678	GO:0150094	1	0.98614114	0	7
15679	GO:0150098	1	0.998004011	0	1
15680	GO:0150099	1	0.992011646	0	4
15681	GO:0150101	1	0.998013437	0	1
15682	GO:0150102	1	0.995949756	0	2
15683	GO:0150103	1	0.998005048	0	1
15684	GO:0150104	1	0.855485989	0	78
15685	GO:0150105	1	0.984191648	0	8
15686	GO:0150106	1	0.997997716	0	1
15687	GO:0150107	1	0.997966837	0	1

15688	GO:0150110	1	0.998013437	0	1
15689	GO:0150111	1	0.991939928	0	4
15690	GO:0150115	1	0.9980126	0	1
15691	GO:0150116	1	0.997990564	0	1
15692	GO:0150128	1	0.997941607	0	1
15693	GO:0150129	1	0.997972866	0	1
15694	GO:0150142	1	0.997988898	0	1
15695	GO:0150145	1	0.997988898	0	1
15696	GO:0150172	1	0.99599906	0	2
15697	GO:0150175	1	0.997981704	0	1
15698	GO:0150178	1	0.997981704	0	1
15699	GO:0198738	1	0.996030299	0	2
15700	GO:1900004	1	0.987935161	0	6
15701	GO:1900005	1	0.998013437	0	1
15702	GO:1900006	1	0.968541476	0	16
15703	GO:1900015	1	0.984093224	0	8
15704	GO:1900016	1	0.964479348	0	18
15705	GO:1900017	1	0.964509101	0	18
15706	GO:1900020	1	0.986076485	0	7
15707	GO:1900022	1	0.998001863	0	1
15708	GO:1900024	1	0.992020737	0	4
15710	GO:1900026	1	0.924463562	0	39
15711	GO:1900027	1	0.982056499	0	9
15712	GO:1900028	1	0.987987446	0	6
15713	GO:1900029	1	0.97224368	0	14
15714	GO:1900034	1	0.856591803	0	77
15715	GO:1900035	1	0.997989991	0	1
15716	GO:1900036	1	0.997984046	0	1
15717	GO:1900037	1	0.991994865	0	4
15718	GO:1900038	1	0.996001491	0	2
15719	GO:1900039	1	0.998000292	0	1
15720	GO:1900044	1	0.998013437	0	1
15721	GO:1900045	1	0.993990088	0	3
15722	GO:1900046	1	0.997975561	0	1
15723	GO:1900047	1	0.997986078	0	1
15724	GO:1900051	1	0.952857462	0	24
15725	GO:1900053	1	0.997964698	0	1
15726	GO:1900054	1	0.998002091	0	1
15727	GO:1900060	1	0.991928112	0	4
15728	GO:1900063	1	0.992017724	0	4
15729	GO:1900069	1	0.992003775	0	4
15730	GO:1900075	1	0.997971277	0	1
15731	GO:1900076	1	0.991977222	0	4
15732	GO:1900077	1	0.997998085	0	1
15733	GO:1900078	1	0.992004115	0	4

15734	GO:1900079	1	0.997977002	0	1
15735	GO:1900082	1	0.998013437	0	1
15736	GO:1900085	1	0.997989294	0	1
15737	GO:1900086	1	0.990047631	0	5
15738	GO:1900087	1	0.947202158	0	27
15739	GO:1900095	1	0.998013437	0	1
15740	GO:1900099	1	0.998013437	0	1
15741	GO:1900100	1	0.995951546	0	2
15742	GO:1900101	1	0.997990564	0	1
15743	GO:1900102	1	0.993968348	0	3
15744	GO:1900103	1	0.989974297	0	5
15745	GO:1900106	1	0.993987593	0	3
15746	GO:1900107	1	0.997982195	0	1
15747	GO:1900108	1	0.99398949	0	3
15748	GO:1900110	1	0.995942527	0	2
15749	GO:1900112	1	0.996007756	0	2
15750	GO:1900113	1	0.988021899	0	6
15751	GO:1900114	1	0.994032296	0	3
15752	GO:1900116	1	0.995957759	0	2
15753	GO:1900118	1	0.984002498	0	8
15754	GO:1900119	1	0.982089307	0	9
15755	GO:1900120	1	0.998006617	0	1
15756	GO:1900121	1	0.984087087	0	8
15757	GO:1900122	1	0.98801002	0	6
15758	GO:1900125	1	0.998013267	0	1
15759	GO:1900126	1	0.994039764	0	3
15760	GO:1900127	1	0.992035485	0	4
15761	GO:1900131	1	0.99594889	0	2
15762	GO:1900134	1	0.998005521	0	1
15763	GO:1900135	1	0.995987455	0	2
15764	GO:1900138	1	0.991962169	0	4
15765	GO:1900139	1	0.996007429	0	2
15766	GO:1900142	1	0.997986735	0	1
15767	GO:1900143	1	0.998013437	0	1
15768	GO:1900148	1	0.997990564	0	1
15769	GO:1900149	1	0.998013437	0	1
15770	GO:1900153	1	0.972297148	0	14
15771	GO:1900155	1	0.995986023	0	2
15772	GO:1900158	1	0.994001622	0	3
15773	GO:1900159	1	0.997984615	0	1
15774	GO:1900163	1	0.997991137	0	1
15775	GO:1900164	1	0.995960555	0	2
15776	GO:1900168	1	0.994004246	0	3
15777	GO:1900175	1	0.997971213	0	1
15778	GO:1900176	1	0.99797398	0	1

15779	GO:1900180	1	0.982022656	0	9
15780	GO:1900181	1	0.962658073	0	19
15781	GO:1900182	1	0.937748717	0	32
15782	GO:1900186	1	0.98998707	0	5
15783	GO:1900194	1	0.993955901	0	3
15784	GO:1900195	1	0.9939837	0	3
15785	GO:1900208	1	0.995940968	0	2
15786	GO:1900210	1	0.995946034	0	2
15787	GO:1900212	1	0.998006243	0	1
15788	GO:1900215	1	0.998006243	0	1
15789	GO:1900218	1	0.998006243	0	1
15790	GO:1900220	1	0.996028617	0	2
15791	GO:1900221	1	0.99394799	0	3
15792	GO:1900222	1	0.986012041	0	7
15793	GO:1900223	1	0.987995672	0	6
15794	GO:1900224	1	0.998013437	0	1
15795	GO:1900225	1	0.997989859	0	1
15796	GO:1900226	1	0.99401744	0	3
15797	GO:1900227	1	0.982125102	0	9
15798	GO:1900229	1	0.995979137	0	2
15799	GO:1900235	1	0.998013364	0	1
15800	GO:1900242	1	0.976238649	0	12
15801	GO:1900243	1	0.998013246	0	1
15802	GO:1900244	1	0.982115205	0	9
15805	GO:1900248	1	0.996030717	0	2
15806	GO:1900260	1	0.997976824	0	1
15807	GO:1900264	1	0.981953444	0	9
15808	GO:1900271	1	0.978091045	0	11
15809	GO:1900272	1	0.978138003	0	11
15810	GO:1900273	1	0.960745154	0	20
15811	GO:1900275	1	0.996018183	0	2
15812	GO:1900276	1	0.998000872	0	1
15813	GO:1900280	1	0.997970959	0	1
15814	GO:1900281	1	0.998001552	0	1
15815	GO:1900363	1	0.997998642	0	1
15816	GO:1900364	1	0.984112229	0	8
15817	GO:1900365	1	0.992025237	0	4
15818	GO:1900369	1	0.998013437	0	1
15819	GO:1900370	1	0.998013354	0	1
15820	GO:1900383	1	0.99799835	0	1
15821	GO:1900407	1	0.984087593	0	8
15822	GO:1900408	1	0.996028464	0	2
15823	GO:1900424	1	0.996019673	0	2
15824	GO:1900425	1	0.987896737	0	6
15825	GO:1900426	1	0.991924395	0	4

15826	GO:1900449	1	0.993972529	0	3
15827	GO:1900450	1	0.997998423	0	1
15828	GO:1900451	1	0.995989274	0	2
15829	GO:1900452	1	0.992055983	0	4
15830	GO:1900453	1	0.993981118	0	3
15831	GO:1900454	1	0.996022217	0	2
15832	GO:1900477	1	0.997979891	0	1
15833	GO:1900480	1	0.997990123	0	1
15834	GO:1900535	1	0.997970421	0	1
15835	GO:1900542	1	0.997990564	0	1
15836	GO:1900625	1	0.994036284	0	3
15837	GO:1900721	1	0.998012907	0	1
15838	GO:1900737	1	0.998000872	0	1
15839	GO:1900738	1	0.995946055	0	2
15840	GO:1900740	1	0.950870029	0	25
15841	GO:1900744	1	0.988051456	0	6
15842	GO:1900745	1	0.948962641	0	26
15843	GO:1900746	1	0.99193725	0	4
15844	GO:1900747	1	0.980081229	0	10
15845	GO:1900748	1	0.988097754	0	6
15846	GO:1900753	1	0.998010375	0	1
15847	GO:1900825	1	0.995971644	0	2
15848	GO:1900827	1	0.998013437	0	1
15849	GO:1900924	1	0.995968606	0	2
15850	GO:1901003	1	0.997990564	0	1
15851	GO:1901006	1	0.9979656	0	1
15852	GO:1901016	1	0.988064392	0	6
15853	GO:1901017	1	0.98809066	0	6
15854	GO:1901018	1	0.990018161	0	5
15855	GO:1901019	1	0.998013437	0	1
15856	GO:1901020	1	0.9959861	0	2
15857	GO:1901021	1	0.998013437	0	1
15858	GO:1901028	1	0.995968021	0	2
15859	GO:1901029	1	0.981983537	0	9
15860	GO:1901030	1	0.978009418	0	11
15861	GO:1901031	1	0.993982539	0	3
15862	GO:1901052	1	0.997961622	0	1
15863	GO:1901053	1	0.997999042	0	1
15864	GO:1901074	1	0.997989518	0	1
15865	GO:1901076	1	0.995978683	0	2
15866	GO:1901077	1	0.99797867	0	1
15867	GO:1901081	1	0.997990564	0	1
15868	GO:1901086	1	0.998013026	0	1
15869	GO:1901096	1	0.994007776	0	3
15870	GO:1901097	1	0.990018007	0	5

15871	GO:1901098	1	0.988049203	0	6
15872	GO:1901128	1	0.997982764	0	1
15873	GO:1901135	1	0.996012088	0	2
15874	GO:1901136	1	0.997977617	0	1
15875	GO:1901137	1	0.996012088	0	2
15876	GO:1901142	1	0.996024838	0	2
15877	GO:1901143	1	0.993985068	0	3
15878	GO:1901163	1	0.998013138	0	1
15879	GO:1901164	1	0.989949763	0	5
15880	GO:1901165	1	0.986008833	0	7
15881	GO:1901166	1	0.99006748	0	5
15882	GO:1901184	1	0.998013437	0	1
15883	GO:1901185	1	0.988049136	0	6
15884	GO:1901186	1	0.997984402	0	1
15885	GO:1901187	1	0.998013437	0	1
15886	GO:1901189	1	0.997984402	0	1
15887	GO:1901194	1	0.997962779	0	1
15888	GO:1901201	1	0.992029722	0	4
15889	GO:1901202	1	0.997984299	0	1
15890	GO:1901203	1	0.992041025	0	4
15891	GO:1901207	1	0.998007329	0	1
15892	GO:1901208	1	0.998000023	0	1
15893	GO:1901211	1	0.998000023	0	1
15894	GO:1901214	1	0.960577094	0	20
15895	GO:1901215	1	0.893268815	0	56
15896	GO:1901216	1	0.924696283	0	39
15897	GO:1901222	1	0.970158518	0	15
15898	GO:1901223	1	0.950988817	0	25
15899	GO:1901224	1	0.875374667	0	66
15900	GO:1901227	1	0.997962211	0	1
15901	GO:1901228	1	0.997962211	0	1
15902	GO:1901231	1	0.997988044	0	1
15903	GO:1901233	1	0.997988044	0	1
15904	GO:1901235	1	0.997998598	0	1
15905	GO:1901248	1	0.997988743	0	1
15906	GO:1901253	1	0.997959046	0	1
15907	GO:1901254	1	0.99801048	0	1
15908	GO:1901255	1	0.995988788	0	2
15909	GO:1901256	1	0.998002548	0	1
15910	GO:1901258	1	0.997989921	0	1
15911	GO:1901264	1	0.995960442	0	2
15912	GO:1901291	1	0.997977171	0	1
15913	GO:1901292	1	0.997978935	0	1
15914	GO:1901296	1	0.997975268	0	1
15915	GO:1901297	1	0.997984402	0	1

15916	GO:1901299	1	0.995996114	0	2
15917	GO:1901300	1	0.991986603	0	4
15918	GO:1901301	1	0.998011696	0	1
15919	GO:1901303	1	0.997989952	0	1
15920	GO:1901307	1	0.997973416	0	1
15921	GO:1901310	1	0.997983538	0	1
15922	GO:1901315	1	0.992041496	0	4
15923	GO:1901317	1	0.995950728	0	2
15924	GO:1901318	1	0.993970402	0	3
15925	GO:1901329	1	0.997975784	0	1
15926	GO:1901331	1	0.997996836	0	1
15927	GO:1901339	1	0.997996708	0	1
15928	GO:1901340	1	0.99800873	0	1
15929	GO:1901341	1	0.997977969	0	1
15930	GO:1901342	1	0.995973148	0	2
15931	GO:1901346	1	0.997982468	0	1
15932	GO:1901355	1	0.995969202	0	2
15933	GO:1901360	1	0.997997382	0	1
15934	GO:1901363	1	0.993951656	0	3
15935	GO:1901373	1	0.997968397	0	1
15936	GO:1901374	1	0.997976127	0	1
15937	GO:1901379	1	0.968381542	0	16
15938	GO:1901380	1	0.980150821	0	10
15939	GO:1901381	1	0.935902334	0	33
15940	GO:1901382	1	0.996005511	0	2
15941	GO:1901383	1	0.998013437	0	1
15942	GO:1901385	1	0.98408664	0	8
15943	GO:1901386	1	0.9840273	0	8
15944	GO:1901387	1	0.983981993	0	8
15945	GO:1901388	1	0.990073667	0	5
15946	GO:1901389	1	0.997987328	0	1
15947	GO:1901390	1	0.998001633	0	1
15948	GO:1901394	1	0.998009214	0	1
15949	GO:1901398	1	0.998009699	0	1
15950	GO:1901407	1	0.99600287	0	2
15951	GO:1901423	1	0.997962143	0	1
15952	GO:1901474	1	0.995969534	0	2
15953	GO:1901475	1	0.995981428	0	2
15954	GO:1901480	1	0.991981263	0	4
15955	GO:1901485	1	0.994006633	0	3
15956	GO:1901491	1	0.99403313	0	3
15957	GO:1901492	1	0.995997299	0	2
15958	GO:1901494	1	0.998013437	0	1
15960	GO:1901522	1	0.984109429	0	8
15961	GO:1901525	1	0.992020328	0	4

15962	GO:1901526	1	0.991990206	0	4
15963	GO:1901529	1	0.996007119	0	2
15964	GO:1901532	1	0.995966912	0	2
15965	GO:1901533	1	0.993945719	0	3
15966	GO:1901534	1	0.994029043	0	3
15967	GO:1901536	1	0.993962202	0	3
15968	GO:1901537	1	0.990078293	0	5
15969	GO:1901555	1	0.998013437	0	1
15970	GO:1901557	1	0.998007466	0	1
15971	GO:1901558	1	0.99599357	0	2
15972	GO:1901563	1	0.998013077	0	1
15973	GO:1901565	1	0.997990564	0	1
15974	GO:1901581	1	0.998013437	0	1
15975	GO:1901582	1	0.998013437	0	1
15976	GO:1901585	1	0.997991656	0	1
15977	GO:1901588	1	0.995996758	0	2
15978	GO:1901594	1	0.998008787	0	1
15979	GO:1901605	1	0.997982267	0	1
15980	GO:1901610	1	0.997998598	0	1
15981	GO:1901611	1	0.991950513	0	4
15982	GO:1901612	1	0.980076442	0	10
15983	GO:1901620	1	0.994023691	0	3
15984	GO:1901621	1	0.990027645	0	5
15985	GO:1901622	1	0.997987919	0	1
15986	GO:1901623	1	0.997990564	0	1
15987	GO:1901624	1	0.998010468	0	1
15988	GO:1901625	1	0.997990564	0	1
15989	GO:1901626	1	0.997971277	0	1
15990	GO:1901631	1	0.998013437	0	1
15991	GO:1901640	1	0.998013437	0	1
15992	GO:1901641	1	0.998013437	0	1
15993	GO:1901642	1	0.985976791	0	7
15994	GO:1901647	1	0.995947427	0	2
15995	GO:1901652	1	0.985955775	0	7
15996	GO:1901653	1	0.968371586	0	16
15997	GO:1901660	1	0.994051875	0	3
15998	GO:1901661	1	0.995956042	0	2
15999	GO:1901662	1	0.997963052	0	1
16000	GO:1901666	1	0.995983482	0	2
16001	GO:1901668	1	0.997990564	0	1
16002	GO:1901671	1	0.99192496	0	4
16003	GO:1901673	1	0.962440878	0	19
16004	GO:1901675	1	0.998013437	0	1
16005	GO:1901676	1	0.998006732	0	1
16006	GO:1901679	1	0.997982916	0	1

16007	GO:1901684	1	0.997982764	0	1
16008	GO:1901687	1	0.958121705	0	21
16009	GO:1901696	1	0.997988603	0	1
16010	GO:1901698	1	0.997986438	0	1
16011	GO:1901706	1	0.995991813	0	2
16012	GO:1901707	1	0.99796583	0	1
16013	GO:1901723	1	0.997990014	0	1
16014	GO:1901724	1	0.997990564	0	1
16015	GO:1901725	1	0.998008812	0	1
16016	GO:1901726	1	0.996011152	0	2
16017	GO:1901727	1	0.98807879	0	6
16018	GO:1901731	1	0.994004842	0	3
16020	GO:1901741	1	0.974152103	0	13
16021	GO:1901750	1	0.983971715	0	8
16022	GO:1901751	1	0.99798452	0	1
16023	GO:1901753	1	0.997987546	0	1
16024	GO:1901796	1	0.739060889	0	150
16025	GO:1901797	1	0.990011809	0	5
16026	GO:1901798	1	0.981894668	0	9
16028	GO:1901800	1	0.966262378	0	17
16029	GO:1901805	1	0.99798512	0	1
16030	GO:1901810	1	0.997990564	0	1
16031	GO:1901827	1	0.997988448	0	1
16032	GO:1901835	1	0.997974207	0	1
16033	GO:1901836	1	0.997973786	0	1
16034	GO:1901837	1	0.99197925	0	4
16035	GO:1901838	1	0.980102363	0	10
16036	GO:1901841	1	0.988020121	0	6
16037	GO:1901842	1	0.989954745	0	5
16038	GO:1901843	1	0.990025752	0	5
16039	GO:1901844	1	0.982058578	0	9
16040	GO:1901845	1	0.998007892	0	1
16041	GO:1901856	1	0.997983601	0	1
16042	GO:1901857	1	0.984013512	0	8
16043	GO:1901858	1	0.995960485	0	2
16044	GO:1901860	1	0.998013437	0	1
16045	GO:1901862	1	0.998013437	0	1
16046	GO:1901863	1	0.998013437	0	1
16047	GO:1901873	1	0.998013437	0	1
16048	GO:1901874	1	0.996007903	0	2
16049	GO:1901877	1	0.995987277	0	2
16050	GO:1901888	1	0.986105402	0	7
16051	GO:1901890	1	0.991922179	0	4
16052	GO:1901895	1	0.992026358	0	4
16053	GO:1901896	1	0.990013446	0	5

16054	GO:1901897	1	0.998008812	0	1
16055	GO:1901898	1	0.996027113	0	2
16056	GO:1901899	1	0.9959681	0	2
16057	GO:1901907	1	0.98997282	0	5
16058	GO:1901909	1	0.98997282	0	5
16059	GO:1901911	1	0.98997282	0	5
16060	GO:1901919	1	0.997990495	0	1
16061	GO:1901953	1	0.996013933	0	2
16062	GO:1901962	1	0.997982324	0	1
16063	GO:1901964	1	0.997974922	0	1
16064	GO:1901978	1	0.99799543	0	1
16065	GO:1901979	1	0.998005048	0	1
16066	GO:1901980	1	0.995978683	0	2
16067	GO:1901981	1	0.960548419	0	20
16068	GO:1901984	1	0.993990129	0	3
16069	GO:1901985	1	0.980108787	0	10
16070	GO:1901986	1	0.997968079	0	1
16071	GO:1901987	1	0.993996864	0	3
16072	GO:1901989	1	0.997987709	0	1
16073	GO:1901990	1	0.848518852	0	81
16074	GO:1901991	1	0.991988847	0	4
16075	GO:1901992	1	0.994017866	0	3
16076	GO:1901994	1	0.997994221	0	1
16077	GO:1901998	1	0.924439509	0	39
16078	GO:1902000	1	0.997969048	0	1
16079	GO:1902003	1	0.996026143	0	2
16080	GO:1902004	1	0.96837949	0	16
16081	GO:1902010	1	0.998001538	0	1
16082	GO:1902017	1	0.962448322	0	19
16083	GO:1902018	1	0.978134275	0	11
16084	GO:1902023	1	0.998006135	0	1
16085	GO:1902031	1	0.996015095	0	2
16086	GO:1902033	1	0.993991308	0	3
16087	GO:1902035	1	0.990014476	0	5
16088	GO:1902036	1	0.876690947	0	65
16089	GO:1902037	1	0.993983562	0	3
16090	GO:1902038	1	0.998000906	0	1
16091	GO:1902041	1	0.968278403	0	16
16092	GO:1902042	1	0.935699218	0	33
16093	GO:1902043	1	0.980155852	0	10
16094	GO:1902044	1	0.996013178	0	2
16095	GO:1902045	1	0.997984362	0	1
16096	GO:1902047	1	0.99800761	0	1
16097	GO:1902064	1	0.998003127	0	1
16098	GO:1902065	1	0.98797577	0	6

16099	GO:1902070	1	0.99796964	0	1
16100	GO:1902073	1	0.997963732	0	1
16101	GO:1902074	1	0.989962603	0	5
16102	GO:1902078	1	0.998013437	0	1
16103	GO:1902081	1	0.997990564	0	1
16104	GO:1902082	1	0.998002124	0	1
16105	GO:1902083	1	0.990074374	0	5
16106	GO:1902093	1	0.987941862	0	6
16107	GO:1902098	1	0.998012112	0	1
16108	GO:1902108	1	0.987980473	0	6
16109	GO:1902109	1	0.99193026	0	4
16110	GO:1902110	1	0.991964066	0	4
16111	GO:1902115	1	0.992036213	0	4
16112	GO:1902117	1	0.997981243	0	1
16113	GO:1902121	1	0.998012112	0	1
16114	GO:1902153	1	0.997990564	0	1
16115	GO:1902161	1	0.998013437	0	1
16116	GO:1902164	1	0.993926676	0	3
16117	GO:1902165	1	0.997993666	0	1
16118	GO:1902166	1	0.97012468	0	15
16119	GO:1902167	1	0.9979518	0	1
16120	GO:1902170	1	0.998007485	0	1
16121	GO:1902172	1	0.995972843	0	2
16122	GO:1902173	1	0.997997136	0	1
16123	GO:1902174	1	0.995968454	0	2
16124	GO:1902175	1	0.99600058	0	2
16125	GO:1902176	1	0.974088245	0	13
16126	GO:1902177	1	0.991924071	0	4
16127	GO:1902178	1	0.99801	0	1
16128	GO:1902186	1	0.99401015	0	3
16129	GO:1902187	1	0.974294396	0	13
16130	GO:1902188	1	0.980016834	0	10
16131	GO:1902202	1	0.995976653	0	2
16132	GO:1902203	1	0.99801283	0	1
16133	GO:1902204	1	0.997978777	0	1
16134	GO:1902206	1	0.99797354	0	1
16135	GO:1902215	1	0.99797354	0	1
16136	GO:1902216	1	0.995991891	0	2
16137	GO:1902219	1	0.99204353	0	4
16138	GO:1902220	1	0.997962143	0	1
16139	GO:1902224	1	0.997980886	0	1
16140	GO:1902227	1	0.995947715	0	2
16141	GO:1902228	1	0.997985513	0	1
16142	GO:1902230	1	0.972137253	0	14
16143	GO:1902231	1	0.989941394	0	5

16144	GO:1902232	1	0.996002383	0	2
16145	GO:1902233	1	0.99797354	0	1
16146	GO:1902235	1	0.99397325	0	3
16147	GO:1902236	1	0.966335664	0	17
16148	GO:1902237	1	0.978013393	0	11
16149	GO:1902239	1	0.998009244	0	1
16150	GO:1902253	1	0.997988013	0	1
16151	GO:1902254	1	0.990031968	0	5
16152	GO:1902255	1	0.993879054	0	3
16153	GO:1902256	1	0.998013192	0	1
16154	GO:1902257	1	0.995977893	0	2
16155	GO:1902259	1	0.988034277	0	6
16156	GO:1902260	1	0.980100703	0	10
16157	GO:1902261	1	0.992038971	0	4
16158	GO:1902262	1	0.991976784	0	4
16159	GO:1902263	1	0.993994406	0	3
16160	GO:1902268	1	0.993910101	0	3
16161	GO:1902269	1	0.996004346	0	2
16162	GO:1902270	1	0.997998598	0	1
16163	GO:1902271	1	0.997971972	0	1
16164	GO:1902275	1	0.984138582	0	8
16165	GO:1902282	1	0.980112885	0	10
16166	GO:1902283	1	0.996002248	0	2
16167	GO:1902285	1	0.992038585	0	4
16168	GO:1902287	1	0.97636354	0	12
16169	GO:1902305	1	0.984090216	0	8
16170	GO:1902306	1	0.993988502	0	3
16171	GO:1902307	1	0.995995599	0	2
16172	GO:1902309	1	0.994013641	0	3
16173	GO:1902310	1	0.997984742	0	1
16174	GO:1902311	1	0.997974731	0	1
16175	GO:1902336	1	0.995992287	0	2
16176	GO:1902339	1	0.994011173	0	3
16177	GO:1902356	1	0.991950994	0	4
16178	GO:1902358	1	0.968398426	0	16
16179	GO:1902362	1	0.997991284	0	1
16180	GO:1902365	1	0.998013437	0	1
16181	GO:1902367	1	0.995973741	0	2
16182	GO:1902369	1	0.997990564	0	1
16183	GO:1902373	1	0.994027041	0	3
16184	GO:1902378	1	0.997986391	0	1
16185	GO:1902379	1	0.995971199	0	2
16186	GO:1902380	1	0.997985419	0	1
16187	GO:1902387	1	0.985967185	0	7
16188	GO:1902388	1	0.985982162	0	7

16189	GO:1902389	1	0.985982162	0	7
16190	GO:1902396	1	0.990064202	0	5
16191	GO:1902410	1	0.995986343	0	2
16192	GO:1902412	1	0.991999097	0	4
16193	GO:1902414	1	0.982111868	0	9
16194	GO:1902415	1	0.99597218	0	2
16195	GO:1902416	1	0.989959993	0	5
16196	GO:1902425	1	0.995998422	0	2
16197	GO:1902426	1	0.995961585	0	2
16198	GO:1902430	1	0.962507363	0	19
16199	GO:1902437	1	0.996030731	0	2
16200	GO:1902440	1	0.997990564	0	1
16201	GO:1902443	1	0.998004752	0	1
16202	GO:1902444	1	0.997963936	0	1
16203	GO:1902445	1	0.99391282	0	3
16204	GO:1902455	1	0.993977089	0	3
16205	GO:1902459	1	0.984092923	0	8
16206	GO:1902462	1	0.99199106	0	4
16207	GO:1902463	1	0.995969942	0	2
16208	GO:1902466	1	0.995987932	0	2
16209	GO:1902473	1	0.996007392	0	2
16210	GO:1902474	1	0.990097662	0	5
16211	GO:1902475	1	0.976207438	0	12
16212	GO:1902476	1	0.890305402	0	58
16213	GO:1902480	1	0.997985466	0	1
16214	GO:1902488	1	0.997968853	0	1
16215	GO:1902490	1	0.995972821	0	2
16216	GO:1902491	1	0.997956827	0	1
16217	GO:1902494	1	0.978019197	0	11
16218	GO:1902495	1	0.994031396	0	3
16219	GO:1902498	1	0.998013437	0	1
16220	GO:1902499	1	0.993967863	0	3
16221	GO:1902501	1	0.996029001	0	2
16222	GO:1902504	1	0.997994159	0	1
16223	GO:1902510	1	0.993920924	0	3
16224	GO:1902511	1	0.993997515	0	3
16225	GO:1902512	1	0.989861613	0	5
16226	GO:1902513	1	0.990048384	0	5
16227	GO:1902514	1	0.986036614	0	7
16228	GO:1902523	1	0.992047027	0	4
16229	GO:1902524	1	0.993944352	0	3
16230	GO:1902525	1	0.998004306	0	1
16231	GO:1902527	1	0.989952332	0	5
16232	GO:1902530	1	0.996007903	0	2
16233	GO:1902531	1	0.985989772	0	7

16234	GO:1902532	1	0.995973677	0	2
16235	GO:1902533	1	0.982197915	0	9
16236	GO:1902544	1	0.997998115	0	1
16237	GO:1902546	1	0.997957079	0	1
16238	GO:1902548	1	0.996030125	0	2
16239	GO:1902553	1	0.997991749	0	1
16240	GO:1902554	1	0.998005815	0	1
16241	GO:1902559	1	0.995962965	0	2
16243	GO:1902562	1	0.997980545	0	1
16244	GO:1902563	1	0.997959195	0	1
16245	GO:1902564	1	0.99595946	0	2
16246	GO:1902570	1	0.985953624	0	7
16247	GO:1902572	1	0.998003862	0	1
16248	GO:1902586	1	0.997982404	0	1
16249	GO:1902595	1	0.995961771	0	2
16250	GO:1902600	1	0.802281604	0	109
16251	GO:1902603	1	0.991958786	0	4
16252	GO:1902605	1	0.997990386	0	1
16253	GO:1902607	1	0.997988098	0	1
16254	GO:1902608	1	0.99796602	0	1
16255	GO:1902613	1	0.997990464	0	1
16256	GO:1902616	1	0.991958182	0	4
16257	GO:1902617	1	0.990008377	0	5
16258	GO:1902618	1	0.998013437	0	1
16259	GO:1902622	1	0.989928624	0	5
16260	GO:1902624	1	0.997985733	0	1
16261	GO:1902626	1	0.993894803	0	3
16262	GO:1902629	1	0.997967366	0	1
16263	GO:1902630	1	0.997989828	0	1
16264	GO:1902631	1	0.995978131	0	2
16265	GO:1902635	1	0.994027222	0	3
16266	GO:1902636	1	0.993947315	0	3
16267	GO:1902656	1	0.997996769	0	1
16268	GO:1902661	1	0.993963733	0	3
16269	GO:1902667	1	0.988097401	0	6
16270	GO:1902669	1	0.997978885	0	1
16271	GO:1902679	1	0.995942018	0	2
16272	GO:1902680	1	0.995964188	0	2
16273	GO:1902683	1	0.9979831	0	1
16274	GO:1902684	1	0.997966704	0	1
16275	GO:1902685	1	0.994029437	0	3
16276	GO:1902686	1	0.995975502	0	2
16277	GO:1902691	1	0.997995977	0	1
16278	GO:1902692	1	0.996004268	0	2
16279	GO:1902710	1	0.996030731	0	2

16280	GO:1902711	1	0.974231094	0	13
16281	GO:1902722	1	0.993968741	0	3
16282	GO:1902723	1	0.992015328	0	4
16283	GO:1902725	1	0.992029402	0	4
16284	GO:1902728	1	0.996029971	0	2
16285	GO:1902729	1	0.998009529	0	1
16286	GO:1902730	1	0.995967829	0	2
16287	GO:1902731	1	0.992036441	0	4
16288	GO:1902732	1	0.985978322	0	7
16289	GO:1902737	1	0.992032433	0	4
16290	GO:1902742	1	0.980151166	0	10
16291	GO:1902745	1	0.995985075	0	2
16292	GO:1902746	1	0.99798523	0	1
16293	GO:1902747	1	0.987985118	0	6
16294	GO:1902748	1	0.998013437	0	1
16295	GO:1902749	1	0.989998657	0	5
16296	GO:1902750	1	0.99597733	0	2
16297	GO:1902751	1	0.988007921	0	6
16298	GO:1902766	1	0.995947914	0	2
16299	GO:1902771	1	0.998013437	0	1
16300	GO:1902773	1	0.997994964	0	1
16301	GO:1902774	1	0.988039213	0	6
16302	GO:1902775	1	0.99402174	0	3
16303	GO:1902803	1	0.994004895	0	3
16304	GO:1902806	1	0.982134582	0	9
16305	GO:1902807	1	0.989963485	0	5
16306	GO:1902808	1	0.974202219	0	13
16307	GO:1902809	1	0.99799257	0	1
16308	GO:1902811	1	0.997985764	0	1
16309	GO:1902817	1	0.990037588	0	5
16310	GO:1902823	1	0.996016125	0	2
16311	GO:1902824	1	0.995943198	0	2
16312	GO:1902842	1	0.992020461	0	4
16313	GO:1902846	1	0.998013437	0	1
16314	GO:1902847	1	0.997991997	0	1
16315	GO:1902850	1	0.996008451	0	2
16316	GO:1902856	1	0.994034203	0	3
16317	GO:1902857	1	0.98609919	0	7
16318	GO:1902859	1	0.997960497	0	1
16319	GO:1902860	1	0.997980886	0	1
16320	GO:1902866	1	0.998008098	0	1
16321	GO:1902871	1	0.99798349	0	1
16322	GO:1902882	1	0.995990552	0	2
16323	GO:1902883	1	0.993996488	0	3
16324	GO:1902884	1	0.993976746	0	3

16325	GO:1902889	1	0.998013437	0	1
16326	GO:1902894	1	0.972324687	0	14
16327	GO:1902895	1	0.930361912	0	36
16328	GO:1902896	1	0.994003409	0	3
16329	GO:1902897	1	0.994011016	0	3
16330	GO:1902902	1	0.970200158	0	15
16331	GO:1902903	1	0.997958946	0	1
16332	GO:1902904	1	0.997979166	0	1
16333	GO:1902905	1	0.997961031	0	1
16334	GO:1902910	1	0.998009912	0	1
16335	GO:1902911	1	0.994027396	0	3
16336	GO:1902914	1	0.997981599	0	1
16337	GO:1902915	1	0.997953769	0	1
16338	GO:1902916	1	0.987995272	0	6
16339	GO:1902936	1	0.98210265	0	9
16340	GO:1902937	1	0.994001287	0	3
16341	GO:1902938	1	0.996007555	0	2
16342	GO:1902939	1	0.997992724	0	1
16343	GO:1902943	1	0.996026123	0	2
16344	GO:1902945	1	0.992053635	0	4
16345	GO:1902946	1	0.997986391	0	1
16346	GO:1902947	1	0.995966812	0	2
16347	GO:1902948	1	0.996029656	0	2
16348	GO:1902949	1	0.98800333	0	6
16349	GO:1902950	1	0.991994158	0	4
16350	GO:1902951	1	0.993995312	0	3
16351	GO:1902952	1	0.99597931	0	2
16352	GO:1902953	1	0.990036344	0	5
16353	GO:1902954	1	0.998013437	0	1
16354	GO:1902955	1	0.998013437	0	1
16355	GO:1902957	1	0.995991764	0	2
16356	GO:1902958	1	0.993922874	0	3
16357	GO:1902959	1	0.998005453	0	1
16358	GO:1902960	1	0.994017782	0	3
16359	GO:1902961	1	0.986022305	0	7
16360	GO:1902963	1	0.996022762	0	2
16361	GO:1902966	1	0.982221676	0	9
16362	GO:1902969	1	0.99800493	0	1
16363	GO:1902975	1	0.988044228	0	6
16364	GO:1902977	1	0.997977315	0	1
16365	GO:1902979	1	0.997990564	0	1
16366	GO:1902983	1	0.997998744	0	1
16367	GO:1902988	1	0.998013437	0	1
16368	GO:1902990	1	0.99400095	0	3
16369	GO:1902991	1	0.991947717	0	4

16370	GO:1902992	1	0.994004161	0	3
16371	GO:1902993	1	0.992059657	0	4
16372	GO:1902995	1	0.991972575	0	4
16373	GO:1902997	1	0.998013437	0	1
16374	GO:1902998	1	0.997991997	0	1
16375	GO:1903002	1	0.997963771	0	1
16376	GO:1903003	1	0.991984148	0	4
16377	GO:1903006	1	0.996000723	0	2
16378	GO:1903007	1	0.998006243	0	1
16379	GO:1903008	1	0.995985913	0	2
16380	GO:1903010	1	0.994027989	0	3
16381	GO:1903011	1	0.99797739	0	1
16382	GO:1903012	1	0.993967705	0	3
16383	GO:1903020	1	0.995968876	0	2
16384	GO:1903025	1	0.992045184	0	4
16385	GO:1903026	1	0.989922411	0	5
16386	GO:1903027	1	0.995945881	0	2
16387	GO:1903028	1	0.998013437	0	1
16388	GO:1903033	1	0.995968968	0	2
16389	GO:1903034	1	0.997972205	0	1
16390	GO:1903036	1	0.998013437	0	1
16391	GO:1903037	1	0.996030731	0	2
16392	GO:1903038	1	0.988057339	0	6
16393	GO:1903039	1	0.98997188	0	5
16394	GO:1903043	1	0.997995673	0	1
16395	GO:1903044	1	0.993962239	0	3
16396	GO:1903045	1	0.998013432	0	1
16397	GO:1903049	1	0.997991757	0	1
16398	GO:1903051	1	0.995987798	0	2
16399	GO:1903052	1	0.991990133	0	4
16400	GO:1903053	1	0.994017126	0	3
16401	GO:1903054	1	0.997991284	0	1
16402	GO:1903055	1	0.994015439	0	3
16403	GO:1903056	1	0.998013437	0	1
16404	GO:1903059	1	0.998006383	0	1
16405	GO:1903060	1	0.997954992	0	1
16406	GO:1903061	1	0.992002615	0	4
16407	GO:1903064	1	0.998013437	0	1
16408	GO:1903069	1	0.997981486	0	1
16409	GO:1903070	1	0.993972033	0	3
16410	GO:1903071	1	0.984010327	0	8
16411	GO:1903073	1	0.997958946	0	1
16414	GO:1903078	1	0.897237904	0	54
16415	GO:1903082	1	0.997961288	0	1
16416	GO:1903093	1	0.998013437	0	1

16417	GO:1903094	1	0.995928947	0	2
16418	GO:1903096	1	0.997994857	0	1
16419	GO:1903100	1	0.998013437	0	1
16420	GO:1903108	1	0.99599847	0	2
16421	GO:1903109	1	0.995970881	0	2
16422	GO:1903118	1	0.997984505	0	1
16423	GO:1903119	1	0.998008048	0	1
16424	GO:1903122	1	0.997958946	0	1
16425	GO:1903125	1	0.998013437	0	1
16426	GO:1903126	1	0.997980789	0	1
16427	GO:1903135	1	0.991960883	0	4
16428	GO:1903136	1	0.98589869	0	7
16429	GO:1903140	1	0.982123091	0	9
16430	GO:1903141	1	0.996015149	0	2
16431	GO:1903142	1	0.988043927	0	6
16432	GO:1903143	1	0.995972769	0	2
16433	GO:1903146	1	0.968414356	0	16
16434	GO:1903147	1	0.995948584	0	2
16435	GO:1903168	1	0.997958946	0	1
16436	GO:1903169	1	0.986023536	0	7
16437	GO:1903170	1	0.996007858	0	2
16438	GO:1903178	1	0.997958946	0	1
16439	GO:1903181	1	0.997958946	0	1
16440	GO:1903189	1	0.997958946	0	1
16441	GO:1903190	1	0.997958946	0	1
16442	GO:1903197	1	0.997958946	0	1
16443	GO:1903200	1	0.997958946	0	1
16444	GO:1903202	1	0.991942435	0	4
16445	GO:1903203	1	0.996018929	0	2
16446	GO:1903204	1	0.976216361	0	12
16447	GO:1903205	1	0.997990564	0	1
16448	GO:1903206	1	0.983986237	0	8
16449	GO:1903208	1	0.989952626	0	5
16450	GO:1903209	1	0.995950902	0	2
16451	GO:1903214	1	0.985996829	0	7
16452	GO:1903215	1	0.988022469	0	6
16453	GO:1903217	1	0.995980987	0	2
16454	GO:1903223	1	0.994042256	0	3
16455	GO:1903225	1	0.996030731	0	2
16456	GO:1903226	1	0.997990564	0	1
16457	GO:1903231	1	0.973704448	0	13
16458	GO:1903232	1	0.987941733	0	6
16459	GO:1903233	1	0.996030064	0	2
16460	GO:1903235	1	0.99801243	0	1
16461	GO:1903236	1	0.998013437	0	1

16462	GO:1903237	1	0.995918697	0	2
16463	GO:1903238	1	0.988071841	0	6
16464	GO:1903241	1	0.993989384	0	3
16465	GO:1903243	1	0.986047321	0	7
16466	GO:1903244	1	0.99595725	0	2
16467	GO:1903249	1	0.998013437	0	1
16468	GO:1903251	1	0.98995125	0	5
16469	GO:1903259	1	0.997990564	0	1
16470	GO:1903265	1	0.978073622	0	11
16471	GO:1903278	1	0.991926892	0	4
16472	GO:1903280	1	0.998013392	0	1
16473	GO:1903281	1	0.995992061	0	2
16474	GO:1903284	1	0.997996934	0	1
16475	GO:1903285	1	0.996012788	0	2
16476	GO:1903286	1	0.998012926	0	1
16477	GO:1903288	1	0.986083414	0	7
16478	GO:1903292	1	0.993966932	0	3
16479	GO:1903296	1	0.996023549	0	2
16480	GO:1903298	1	0.989978809	0	5
16481	GO:1903301	1	0.997990564	0	1
16482	GO:1903302	1	0.997979364	0	1
16483	GO:1903305	1	0.996018435	0	2
16484	GO:1903307	1	0.989988103	0	5
16485	GO:1903319	1	0.997992848	0	1
16486	GO:1903334	1	0.995983213	0	2
16487	GO:1903343	1	0.998013437	0	1
16488	GO:1903347	1	0.992030024	0	4
16489	GO:1903348	1	0.98801317	0	6
16490	GO:1903350	1	0.997990564	0	1
16491	GO:1903351	1	0.972299788	0	14
16492	GO:1903352	1	0.996030731	0	2
16493	GO:1903358	1	0.974242853	0	13
16494	GO:1903361	1	0.987963535	0	6
16495	GO:1903362	1	0.998011771	0	1
16496	GO:1903363	1	0.989974662	0	5
16497	GO:1903364	1	0.97230651	0	14
16498	GO:1903371	1	0.996000269	0	2
16499	GO:1903373	1	0.992023097	0	4
16500	GO:1903375	1	0.99402484	0	3
16501	GO:1903376	1	0.998007582	0	1
16502	GO:1903377	1	0.99197642	0	4
16503	GO:1903378	1	0.996014878	0	2
16504	GO:1903382	1	0.996005942	0	2
16505	GO:1903384	1	0.995952103	0	2
16506	GO:1903385	1	0.995995809	0	2

16507	GO:1903387	1	0.998013278	0	1
16508	GO:1903392	1	0.994020802	0	3
16509	GO:1903393	1	0.996024833	0	2
16510	GO:1903394	1	0.997984299	0	1
16511	GO:1903400	1	0.995975813	0	2
16512	GO:1903401	1	0.992025388	0	4
16513	GO:1903403	1	0.998006297	0	1
16514	GO:1903408	1	0.993943878	0	3
16515	GO:1903409	1	0.994026217	0	3
16516	GO:1903413	1	0.991919886	0	4
16517	GO:1903416	1	0.992020751	0	4
16518	GO:1903420	1	0.998013437	0	1
16519	GO:1903421	1	0.991956026	0	4
16520	GO:1903422	1	0.996021834	0	2
16521	GO:1903423	1	0.994036012	0	3
16522	GO:1903426	1	0.987998266	0	6
16523	GO:1903427	1	0.978120004	0	11
16524	GO:1903428	1	0.97425856	0	13
16525	GO:1903429	1	0.998013437	0	1
16526	GO:1903431	1	0.997985434	0	1
16527	GO:1903432	1	0.988012835	0	6
16528	GO:1903434	1	0.998006113	0	1
16529	GO:1903435	1	0.997991323	0	1
16530	GO:1903438	1	0.996016465	0	2
16531	GO:1903441	1	0.990057217	0	5
16532	GO:1903442	1	0.998005759	0	1
16533	GO:1903444	1	0.995990718	0	2
16534	GO:1903445	1	0.99596122	0	2
16535	GO:1903452	1	0.998013437	0	1
16536	GO:1903457	1	0.997979224	0	1
16537	GO:1903461	1	0.997995992	0	1
16538	GO:1903463	1	0.997996776	0	1
16539	GO:1903464	1	0.998002144	0	1
16541	GO:1903489	1	0.997975328	0	1
16542	GO:1903494	1	0.997959843	0	1
16543	GO:1903496	1	0.997959843	0	1
16544	GO:1903506	1	0.995981442	0	2
16545	GO:1903507	1	0.991971399	0	4
16546	GO:1903508	1	0.998013437	0	1
16547	GO:1903512	1	0.997979249	0	1
16548	GO:1903514	1	0.997979874	0	1
16549	GO:1903515	1	0.99602969	0	2
16550	GO:1903518	1	0.995991994	0	2
16551	GO:1903522	1	0.997996776	0	1
16552	GO:1903523	1	0.997966894	0	1

16553	GO:1903526	1	0.996018204	0	2
16554	GO:1903527	1	0.9900778	0	5
16555	GO:1903531	1	0.995941004	0	2
16556	GO:1903533	1	0.997982676	0	1
16557	GO:1903538	1	0.997991687	0	1
16558	GO:1903539	1	0.994042588	0	3
16559	GO:1903540	1	0.997971277	0	1
16560	GO:1903542	1	0.992009961	0	4
16561	GO:1903543	1	0.972203596	0	14
16562	GO:1903545	1	0.998000558	0	1
16563	GO:1903546	1	0.990010855	0	5
16564	GO:1903547	1	0.997958626	0	1
16565	GO:1903551	1	0.993989763	0	3
16566	GO:1903553	1	0.99200475	0	4
16567	GO:1903556	1	0.997970594	0	1
16568	GO:1903561	1	0.900624382	0	52
16569	GO:1903564	1	0.997996324	0	1
16570	GO:1903565	1	0.996004237	0	2
16571	GO:1903566	1	0.992038638	0	4
16572	GO:1903568	1	0.998000348	0	1
16573	GO:1903569	1	0.997984568	0	1
16574	GO:1903572	1	0.998001964	0	1
16575	GO:1903573	1	0.987942223	0	6
16576	GO:1903575	1	0.991933528	0	4
16577	GO:1903576	1	0.997979743	0	1
16578	GO:1903577	1	0.995985075	0	2
16579	GO:1903578	1	0.996005882	0	2
16580	GO:1903579	1	0.995914498	0	2
16581	GO:1903580	1	0.998013437	0	1
16582	GO:1903586	1	0.998010534	0	1
16583	GO:1903587	1	0.998013437	0	1
16584	GO:1903588	1	0.989987989	0	5
16585	GO:1903589	1	0.985956012	0	7
16586	GO:1903595	1	0.991941982	0	4
16587	GO:1903597	1	0.995973619	0	2
16588	GO:1903598	1	0.990015348	0	5
16589	GO:1903599	1	0.986014974	0	7
16590	GO:1903608	1	0.986065726	0	7
16591	GO:1903609	1	0.997989294	0	1
16592	GO:1903611	1	0.99797739	0	1
16593	GO:1903612	1	0.997964148	0	1
16594	GO:1903614	1	0.99394713	0	3
16595	GO:1903615	1	0.994003723	0	3
16596	GO:1903620	1	0.99800762	0	1
16597	GO:1903621	1	0.997977693	0	1

16598	GO:1903625	1	0.99797739	0	1
16599	GO:1903626	1	0.998013437	0	1
16600	GO:1903629	1	0.99797739	0	1
16601	GO:1903632	1	0.997964525	0	1
16602	GO:1903634	1	0.99797739	0	1
16603	GO:1903638	1	0.997951347	0	1
16604	GO:1903644	1	0.997987468	0	1
16605	GO:1903645	1	0.995917207	0	2
16606	GO:1903646	1	0.996001124	0	2
16607	GO:1903659	1	0.991999262	0	4
16608	GO:1903660	1	0.997987328	0	1
16609	GO:1903670	1	0.990031824	0	5
16610	GO:1903671	1	0.976211271	0	12
16611	GO:1903672	1	0.962571406	0	19
16612	GO:1903673	1	0.997977247	0	1
16613	GO:1903676	1	0.997977129	0	1
16614	GO:1903679	1	0.993958681	0	3
16615	GO:1903690	1	0.992076839	0	4
16616	GO:1903691	1	0.992062725	0	4
16617	GO:1903697	1	0.997999639	0	1
16618	GO:1903699	1	0.998013437	0	1
16619	GO:1903701	1	0.998013192	0	1
16620	GO:1903704	1	0.99800762	0	1
16621	GO:1903706	1	0.993944781	0	3
16622	GO:1903707	1	0.995972412	0	2
16623	GO:1903709	1	0.998013437	0	1
16624	GO:1903710	1	0.99800761	0	1
16625	GO:1903712	1	0.994020309	0	3
16626	GO:1903713	1	0.997978603	0	1
16627	GO:1903715	1	0.982109244	0	9
16628	GO:1903720	1	0.995954221	0	2
16629	GO:1903721	1	0.98796845	0	6
16630	GO:1903723	1	0.995952939	0	2
16631	GO:1903724	1	0.992039117	0	4
16632	GO:1903725	1	0.995959632	0	2
16633	GO:1903729	1	0.998013437	0	1
16634	GO:1903730	1	0.998013341	0	1
16635	GO:1903743	1	0.997966704	0	1
16636	GO:1903744	1	0.996007898	0	2
16637	GO:1903748	1	0.995988008	0	2
16638	GO:1903751	1	0.99597671	0	2
16639	GO:1903753	1	0.988037545	0	6
16640	GO:1903754	1	0.996029804	0	2
16641	GO:1903755	1	0.997964216	0	1
16642	GO:1903760	1	0.998012926	0	1

16643	GO:1903762	1	0.996022218	0	2
16644	GO:1903763	1	0.991965853	0	4
16645	GO:1903764	1	0.998012926	0	1
16646	GO:1903765	1	0.993995034	0	3
16647	GO:1903766	1	0.99600158	0	2
16648	GO:1903767	1	0.997987546	0	1
16649	GO:1903770	1	0.997992817	0	1
16650	GO:1903771	1	0.997991842	0	1
16651	GO:1903772	1	0.997964467	0	1
16652	GO:1903774	1	0.993954469	0	3
16653	GO:1903775	1	0.995984674	0	2
16654	GO:1903778	1	0.998013437	0	1
16656	GO:1903780	1	0.998007892	0	1
16657	GO:1903786	1	0.998013437	0	1
16658	GO:1903788	1	0.99799034	0	1
16659	GO:1903790	1	0.99600301	0	2
16660	GO:1903798	1	0.997968918	0	1
16661	GO:1903799	1	0.980163221	0	10
16662	GO:1903800	1	0.98004383	0	10
16663	GO:1903801	1	0.994009977	0	3
16664	GO:1903803	1	0.997977382	0	1
16665	GO:1903804	1	0.997997701	0	1
16666	GO:1903806	1	0.998013349	0	1
16667	GO:1903810	1	0.998013437	0	1
16668	GO:1903812	1	0.99801003	0	1
16669	GO:1903817	1	0.994009559	0	3
16670	GO:1903818	1	0.982118505	0	9
16671	GO:1903824	1	0.997992817	0	1
16672	GO:1903825	1	0.992023032	0	4
16673	GO:1903826	1	0.996007903	0	2
16674	GO:1903827	1	0.970425042	0	15
16675	GO:1903828	1	0.998008675	0	1
16676	GO:1903829	1	0.986139131	0	7
16677	GO:1903830	1	0.970363942	0	15
16678	GO:1903839	1	0.997986219	0	1
16679	GO:1903841	1	0.996001019	0	2
16680	GO:1903843	1	0.994002258	0	3
16681	GO:1903845	1	0.997977936	0	1
16682	GO:1903847	1	0.9979419	0	1
16683	GO:1903849	1	0.998013437	0	1
16684	GO:1903850	1	0.997977129	0	1
16685	GO:1903852	1	0.993935842	0	3
16686	GO:1903860	1	0.996024447	0	2
16687	GO:1903861	1	0.964651954	0	18
16688	GO:1903862	1	0.993960566	0	3

16689	GO:1903883	1	0.998004446	0	1
16690	GO:1903886	1	0.998004446	0	1
16691	GO:1903891	1	0.99800725	0	1
16692	GO:1903892	1	0.995995981	0	2
16693	GO:1903893	1	0.997986594	0	1
16694	GO:1903894	1	0.99396139	0	3
16695	GO:1903895	1	0.989982079	0	5
16696	GO:1903896	1	0.984018026	0	8
16697	GO:1903897	1	0.99800725	0	1
16698	GO:1903898	1	0.988029984	0	6
16699	GO:1903899	1	0.991958913	0	4
16701	GO:1903901	1	0.995946164	0	2
16702	GO:1903902	1	0.986084063	0	7
16703	GO:1903903	1	0.997990285	0	1
16704	GO:1903904	1	0.997990564	0	1
16705	GO:1903905	1	0.9960061	0	2
16706	GO:1903906	1	0.997990285	0	1
16707	GO:1903909	1	0.997990285	0	1
16708	GO:1903911	1	0.995955755	0	2
16709	GO:1903912	1	0.990007121	0	5
16710	GO:1903917	1	0.997984742	0	1
16711	GO:1903919	1	0.998013437	0	1
16712	GO:1903920	1	0.997966353	0	1
16713	GO:1903923	1	0.996007624	0	2
16714	GO:1903924	1	0.997982259	0	1
16715	GO:1903926	1	0.997976383	0	1
16716	GO:1903929	1	0.997996889	0	1
16717	GO:1903935	1	0.99800283	0	1
16718	GO:1903936	1	0.989951691	0	5
16719	GO:1903937	1	0.996019506	0	2
16720	GO:1903944	1	0.995995299	0	2
16721	GO:1903946	1	0.997983808	0	1
16722	GO:1903949	1	0.998008236	0	1
16723	GO:1903954	1	0.998007329	0	1
16724	GO:1903955	1	0.941544555	0	30
16725	GO:1903958	1	0.994006385	0	3
16726	GO:1903965	1	0.997967573	0	1
16728	GO:1903974	1	0.996017591	0	2
16729	GO:1903976	1	0.995977413	0	2
16730	GO:1903977	1	0.99801331	0	1
16731	GO:1903978	1	0.99197265	0	4
16732	GO:1903979	1	0.988070759	0	6
16733	GO:1903980	1	0.988025872	0	6
16734	GO:1903981	1	0.995943541	0	2
16735	GO:1903984	1	0.994017856	0	3

16736	GO:1903988	1	0.997999682	0	1
16737	GO:1903996	1	0.99796953	0	1
16738	GO:1903997	1	0.986056432	0	7
16739	GO:1903999	1	0.991968802	0	4
16740	GO:1904000	1	0.993992533	0	3
16741	GO:1904003	1	0.998001633	0	1
16742	GO:1904009	1	0.997968079	0	1
16743	GO:1904010	1	0.997968079	0	1
16744	GO:1904015	1	0.997992902	0	1
16745	GO:1904016	1	0.995994686	0	2
16746	GO:1904017	1	0.996030731	0	2
16747	GO:1904019	1	0.998013437	0	1
16748	GO:1904021	1	0.995976351	0	2
16749	GO:1904022	1	0.995992197	0	2
16750	GO:1904024	1	0.99399799	0	3
16751	GO:1904026	1	0.995991108	0	2
16752	GO:1904027	1	0.995997959	0	2
16753	GO:1904028	1	0.993993452	0	3
16754	GO:1904030	1	0.991949747	0	4
16755	GO:1904031	1	0.992002281	0	4
16756	GO:1904036	1	0.985959034	0	7
16757	GO:1904037	1	0.992038906	0	4
16758	GO:1904042	1	0.997998773	0	1
16759	GO:1904044	1	0.99194543	0	4
16760	GO:1904045	1	0.993983908	0	3
16761	GO:1904046	1	0.993902601	0	3
16762	GO:1904047	1	0.968250959	0	16
16763	GO:1904049	1	0.996007903	0	2
16764	GO:1904050	1	0.998011417	0	1
16765	GO:1904054	1	0.998013437	0	1
16766	GO:1904056	1	0.995987093	0	2
16767	GO:1904057	1	0.995966752	0	2
16768	GO:1904058	1	0.985980703	0	7
16769	GO:1904059	1	0.99403705	0	3
16770	GO:1904062	1	0.996030731	0	2
16771	GO:1904064	1	0.997980447	0	1
16772	GO:1904071	1	0.994051875	0	3
16773	GO:1904075	1	0.998013437	0	1
16774	GO:1904093	1	0.995947871	0	2
16775	GO:1904100	1	0.993993864	0	3
16776	GO:1904105	1	0.995954828	0	2
16777	GO:1904106	1	0.995987478	0	2
16778	GO:1904109	1	0.998013437	0	1
16779	GO:1904115	1	0.88290281	0	62
16780	GO:1904117	1	0.994015285	0	3

16781	GO:1904120	1	0.997996052	0	1
16782	GO:1904121	1	0.995985514	0	2
16783	GO:1904124	1	0.99797915	0	1
16784	GO:1904139	1	0.997997106	0	1
16785	GO:1904140	1	0.997970155	0	1
16786	GO:1904141	1	0.989961112	0	5
16787	GO:1904151	1	0.995986378	0	2
16788	GO:1904152	1	0.99800189	0	1
16789	GO:1904153	1	0.982060717	0	9
16790	GO:1904154	1	0.993972986	0	3
16791	GO:1904155	1	0.996016353	0	2
16792	GO:1904156	1	0.997999503	0	1
16793	GO:1904158	1	0.989973	0	5
16794	GO:1904161	1	0.995977392	0	2
16795	GO:1904172	1	0.996012787	0	2
16796	GO:1904177	1	0.997990564	0	1
16797	GO:1904178	1	0.997972367	0	1
16798	GO:1904179	1	0.992005649	0	4
16799	GO:1904182	1	0.997963596	0	1
16800	GO:1904184	1	0.996019145	0	2
16801	GO:1904188	1	0.997987927	0	1
16802	GO:1904193	1	0.998013437	0	1
16803	GO:1904197	1	0.998013437	0	1
16804	GO:1904199	1	0.998008236	0	1
16805	GO:1904200	1	0.996005663	0	2
16806	GO:1904205	1	0.998013437	0	1
16807	GO:1904206	1	0.998013437	0	1
16808	GO:1904211	1	0.99599165	0	2
16809	GO:1904213	1	0.998013437	0	1
16810	GO:1904219	1	0.99396456	0	3
16811	GO:1904220	1	0.997990564	0	1
16812	GO:1904221	1	0.997980382	0	1
16813	GO:1904222	1	0.99396456	0	3
16814	GO:1904224	1	0.993965715	0	3
16815	GO:1904227	1	0.997981631	0	1
16816	GO:1904231	1	0.996007903	0	2
16817	GO:1904234	1	0.995980103	0	2
16818	GO:1904237	1	0.994026464	0	3
16819	GO:1904238	1	0.99800715	0	1
16820	GO:1904240	1	0.997967742	0	1
16821	GO:1904246	1	0.997974207	0	1
16822	GO:1904247	1	0.997995551	0	1
16823	GO:1904251	1	0.994011865	0	3
16824	GO:1904256	1	0.997997701	0	1
16825	GO:1904261	1	0.990105641	0	5

16826	GO:1904262	1	0.970280358	0	15
16827	GO:1904263	1	0.964435359	0	18
16828	GO:1904268	1	0.998013437	0	1
16829	GO:1904270	1	0.995975292	0	2
16830	GO:1904271	1	0.996003399	0	2
16831	GO:1904273	1	0.994008605	0	3
16832	GO:1904274	1	0.997982243	0	1
16833	GO:1904283	1	0.997977701	0	1
16834	GO:1904288	1	0.992029929	0	4
16835	GO:1904290	1	0.99801134	0	1
16836	GO:1904291	1	0.997990564	0	1
16837	GO:1904292	1	0.998010757	0	1
16838	GO:1904293	1	0.993959498	0	3
16839	GO:1904294	1	0.986054099	0	7
16840	GO:1904300	1	0.996026123	0	2
16841	GO:1904303	1	0.99800882	0	1
16842	GO:1904306	1	0.993965788	0	3
16843	GO:1904307	1	0.997996934	0	1
16844	GO:1904313	1	0.99800725	0	1
16845	GO:1904315	1	0.951029199	0	25
16846	GO:1904316	1	0.99800882	0	1
16847	GO:1904317	1	0.99800882	0	1
16848	GO:1904322	1	0.982219666	0	9
16849	GO:1904328	1	0.997990045	0	1
16850	GO:1904338	1	0.995992636	0	2
16851	GO:1904339	1	0.993989768	0	3
16852	GO:1904343	1	0.998013176	0	1
16853	GO:1904346	1	0.995944733	0	2
16854	GO:1904349	1	0.995971651	0	2
16855	GO:1904352	1	0.99596449	0	2
16856	GO:1904353	1	0.998013378	0	1
16857	GO:1904354	1	0.99008358	0	5
16858	GO:1904355	1	0.96639501	0	17
16859	GO:1904356	1	0.997978835	0	1
16860	GO:1904357	1	0.978158036	0	11
16861	GO:1904358	1	0.990045952	0	5
16862	GO:1904372	1	0.998008048	0	1
16863	GO:1904373	1	0.995982684	0	2
16864	GO:1904375	1	0.998013437	0	1
16865	GO:1904378	1	0.993995903	0	3
16866	GO:1904379	1	0.996007903	0	2
16867	GO:1904380	1	0.958659438	0	21
16868	GO:1904381	1	0.994031241	0	3
16869	GO:1904382	1	0.990042171	0	5
16870	GO:1904383	1	0.995977617	0	2

16871	GO:1904385	1	0.974216	0	13
16872	GO:1904386	1	0.998008048	0	1
16873	GO:1904389	1	0.997970686	0	1
16874	GO:1904391	1	0.997970686	0	1
16875	GO:1904393	1	0.996001286	0	2
16876	GO:1904394	1	0.997984062	0	1
16877	GO:1904395	1	0.993986506	0	3
16878	GO:1904397	1	0.997970686	0	1
16879	GO:1904398	1	0.99801008	0	1
16880	GO:1904399	1	0.993924033	0	3
16881	GO:1904404	1	0.998013437	0	1
16882	GO:1904407	1	0.997991269	0	1
16883	GO:1904408	1	0.997963052	0	1
16884	GO:1904411	1	0.996011095	0	2
16885	GO:1904414	1	0.998003307	0	1
16886	GO:1904417	1	0.986062375	0	7
16887	GO:1904423	1	0.996015155	0	2
16888	GO:1904424	1	0.996013334	0	2
16889	GO:1904425	1	0.994003847	0	3
16890	GO:1904426	1	0.997991253	0	1
16891	GO:1904427	1	0.997982484	0	1
16892	GO:1904428	1	0.988089544	0	6
16893	GO:1904430	1	0.99401699	0	3
16894	GO:1904431	1	0.991994368	0	4
16895	GO:1904434	1	0.995941715	0	2
16896	GO:1904437	1	0.995941715	0	2
16897	GO:1904439	1	0.99796271	0	1
16898	GO:1904440	1	0.997997701	0	1
16899	GO:1904442	1	0.997999639	0	1
16900	GO:1904447	1	0.992003494	0	4
16901	GO:1904450	1	0.996030001	0	2
16902	GO:1904456	1	0.997976434	0	1
16903	GO:1904457	1	0.99798512	0	1
16904	GO:1904464	1	0.998013437	0	1
16905	GO:1904465	1	0.995960787	0	2
16906	GO:1904466	1	0.993998223	0	3
16907	GO:1904472	1	0.997966894	0	1
16908	GO:1904478	1	0.998013437	0	1
16909	GO:1904479	1	0.997948513	0	1
16910	GO:1904482	1	0.997985984	0	1
16911	GO:1904486	1	0.994010071	0	3
16912	GO:1904491	1	0.98598253	0	7
16913	GO:1904492	1	0.997988199	0	1
16914	GO:1904493	1	0.997988199	0	1
16915	GO:1904504	1	0.990023347	0	5

16916	GO:1904506	1	0.998011553	0	1
16917	GO:1904515	1	0.996016817	0	2
16918	GO:1904526	1	0.994009799	0	3
16919	GO:1904527	1	0.996030719	0	2
16920	GO:1904528	1	0.99198378	0	4
16921	GO:1904530	1	0.997995893	0	1
16922	GO:1904531	1	0.99801343	0	1
16923	GO:1904535	1	0.998011553	0	1
16924	GO:1904539	1	0.998012817	0	1
16925	GO:1904544	1	0.997989146	0	1
16926	GO:1904550	1	0.997979727	0	1
16927	GO:1904556	1	0.996001462	0	2
16928	GO:1904557	1	0.997978603	0	1
16929	GO:1904562	1	0.996030731	0	2
16930	GO:1904566	1	0.997996302	0	1
16931	GO:1904568	1	0.997994528	0	1
16932	GO:1904570	1	0.997974172	0	1
16933	GO:1904574	1	0.997974172	0	1
16934	GO:1904577	1	0.997972635	0	1
16935	GO:1904579	1	0.995970379	0	2
16936	GO:1904582	1	0.99398383	0	3
16937	GO:1904586	1	0.995969629	0	2
16938	GO:1904588	1	0.997965083	0	1
16939	GO:1904589	1	0.997979166	0	1
16940	GO:1904591	1	0.998013401	0	1
16941	GO:1904597	1	0.995982027	0	2
16942	GO:1904602	1	0.995962052	0	2
16943	GO:1904612	1	0.998005815	0	1
16944	GO:1904613	1	0.996026623	0	2
16945	GO:1904616	1	0.997983148	0	1
16946	GO:1904620	1	0.997984457	0	1
16947	GO:1904628	1	0.989970269	0	5
16948	GO:1904631	1	0.997982524	0	1
16949	GO:1904635	1	0.996030257	0	2
16950	GO:1904637	1	0.991968605	0	4
16951	GO:1904639	1	0.998013437	0	1
16952	GO:1904640	1	0.998013437	0	1
16953	GO:1904643	1	0.998013437	0	1
16954	GO:1904645	1	0.974179875	0	13
16955	GO:1904646	1	0.924690802	0	39
16956	GO:1904647	1	0.998013437	0	1
16957	GO:1904649	1	0.997976578	0	1
16958	GO:1904651	1	0.998000656	0	1
16959	GO:1904659	1	0.962506453	0	19
16960	GO:1904666	1	0.996003149	0	2

16961	GO:1904667	1	0.973869782	0	13
16962	GO:1904668	1	0.974191811	0	13
16963	GO:1904669	1	0.994026465	0	3
16964	GO:1904672	1	0.993978552	0	3
16965	GO:1904674	1	0.996009098	0	2
16966	GO:1904677	1	0.997993389	0	1
16967	GO:1904679	1	0.998013437	0	1
16968	GO:1904680	1	0.994025418	0	3
16969	GO:1904681	1	0.99798965	0	1
16970	GO:1904684	1	0.996028044	0	2
16971	GO:1904685	1	0.993949565	0	3
16972	GO:1904690	1	0.998013437	0	1
16973	GO:1904691	1	0.995942698	0	2
16974	GO:1904692	1	0.998003624	0	1
16975	GO:1904693	1	0.994036832	0	3
16976	GO:1904694	1	0.99402909	0	3
16977	GO:1904695	1	0.995971621	0	2
16978	GO:1904702	1	0.998013359	0	1
16979	GO:1904706	1	0.956669514	0	22
16980	GO:1904707	1	0.932182169	0	35
16981	GO:1904708	1	0.997988168	0	1
16982	GO:1904710	1	0.997962143	0	1
16983	GO:1904713	1	0.996007903	0	2
16984	GO:1904714	1	0.989993383	0	5
16985	GO:1904715	1	0.993968849	0	3
16986	GO:1904716	1	0.997984536	0	1
16987	GO:1904717	1	0.994051875	0	3
16988	GO:1904719	1	0.995997445	0	2
16989	GO:1904723	1	0.997975268	0	1
16990	GO:1904724	1	0.903662953	0	50
16991	GO:1904733	1	0.997990564	0	1
16992	GO:1904736	1	0.997990564	0	1
16993	GO:1904738	1	0.998012245	0	1
16994	GO:1904743	1	0.998013437	0	1
16995	GO:1904744	1	0.997961248	0	1
16996	GO:1904749	1	0.993993256	0	3
16997	GO:1904750	1	0.998013437	0	1
16998	GO:1904751	1	0.989942488	0	5
16999	GO:1904753	1	0.984106323	0	8
17000	GO:1904754	1	0.966617978	0	17
17001	GO:1904761	1	0.992042195	0	4
17002	GO:1904762	1	0.998007582	0	1
17003	GO:1904764	1	0.997979166	0	1
17004	GO:1904766	1	0.998013437	0	1
17005	GO:1904768	1	0.993965731	0	3

17006	GO:1904772	1	0.992019165	0	4
17007	GO:1904776	1	0.995975982	0	2
17008	GO:1904777	1	0.997990564	0	1
17009	GO:1904778	1	0.990061193	0	5
17010	GO:1904779	1	0.997985356	0	1
17011	GO:1904780	1	0.996019275	0	2
17012	GO:1904781	1	0.986100001	0	7
17013	GO:1904782	1	0.996014194	0	2
17014	GO:1904783	1	0.989965735	0	5
17015	GO:1904784	1	0.998013399	0	1
17016	GO:1904792	1	0.997993366	0	1
17017	GO:1904796	1	0.99800067	0	1
17018	GO:1904797	1	0.998013437	0	1
17019	GO:1904798	1	0.990020002	0	5
17020	GO:1904800	1	0.99800714	0	1
17021	GO:1904803	1	0.9979518	0	1
17022	GO:1904808	1	0.995985075	0	2
17023	GO:1904812	1	0.998007125	0	1
17024	GO:1904813	1	0.784421758	0	120
17025	GO:1904815	1	0.997990564	0	1
17026	GO:1904816	1	0.993997938	0	3
17027	GO:1904823	1	0.993959725	0	3
17028	GO:1904825	1	0.989982991	0	5
17029	GO:1904828	1	0.998013437	0	1
17030	GO:1904831	1	0.995956989	0	2
17031	GO:1904835	1	0.996003648	0	2
17032	GO:1904837	1	0.943516554	0	29
17033	GO:1904841	1	0.99595282	0	2
17034	GO:1904842	1	0.997973469	0	1
17035	GO:1904843	1	0.997981211	0	1
17036	GO:1904844	1	0.997986438	0	1
17037	GO:1904845	1	0.99395731	0	3
17038	GO:1904850	1	0.997993366	0	1
17039	GO:1904851	1	0.980011287	0	10
17040	GO:1904854	1	0.99799724	0	1
17041	GO:1904855	1	0.997965944	0	1
17042	GO:1904861	1	0.988059159	0	6
17043	GO:1904862	1	0.982195903	0	9
17044	GO:1904864	1	0.996016171	0	2
17045	GO:1904867	1	0.997976203	0	1
17046	GO:1904871	1	0.978047002	0	11
17047	GO:1904872	1	0.992011046	0	4
17048	GO:1904874	1	0.970091492	0	15
17049	GO:1904876	1	0.997992802	0	1
17050	GO:1904877	1	0.99798048	0	1

17051	GO:1904878	1	0.996001159	0	2
17052	GO:1904879	1	0.995997291	0	2
17053	GO:1904880	1	0.997986438	0	1
17054	GO:1904881	1	0.996006487	0	2
17055	GO:1904884	1	0.996030731	0	2
17056	GO:1904885	1	0.990052737	0	5
17057	GO:1904886	1	0.96259597	0	19
17058	GO:1904887	1	0.998013437	0	1
17059	GO:1904888	1	0.980148582	0	10
17060	GO:1904891	1	0.993974113	0	3
17061	GO:1904893	1	0.998013437	0	1
17062	GO:1904894	1	0.989906243	0	5
17063	GO:1904896	1	0.997981567	0	1
17064	GO:1904899	1	0.997999172	0	1
17065	GO:1904902	1	0.997972866	0	1
17066	GO:1904903	1	0.980044724	0	10
17067	GO:1904905	1	0.997976587	0	1
17068	GO:1904906	1	0.989997803	0	5
17069	GO:1904908	1	0.996030731	0	2
17070	GO:1904911	1	0.997993366	0	1
17071	GO:1904914	1	0.997993366	0	1
17072	GO:1904924	1	0.997985159	0	1
17073	GO:1904925	1	0.990003486	0	5
17074	GO:1904928	1	0.994048919	0	3
17075	GO:1904929	1	0.988078633	0	6
17076	GO:1904931	1	0.997985167	0	1
17077	GO:1904934	1	0.998013437	0	1
17078	GO:1904938	1	0.994025329	0	3
17079	GO:1904947	1	0.997994758	0	1
17080	GO:1904948	1	0.986069878	0	7
17081	GO:1904949	1	0.998006243	0	1
17082	GO:1904950	1	0.997998991	0	1
17083	GO:1904951	1	0.988052704	0	6
17084	GO:1904953	1	0.990062845	0	5
17085	GO:1904954	1	0.995974425	0	2
17086	GO:1904955	1	0.998013437	0	1
17087	GO:1904956	1	0.995993327	0	2
17088	GO:1904959	1	0.991968887	0	4
17089	GO:1904960	1	0.993907903	0	3
17090	GO:1904970	1	0.995987478	0	2
17091	GO:1904973	1	0.998011759	0	1
17092	GO:1904976	1	0.996024027	0	2
17093	GO:1904977	1	0.997992508	0	1
17094	GO:1904978	1	0.998012241	0	1
17095	GO:1904980	1	0.998013437	0	1

17096	GO:1904983	1	0.997980675	0	1
17097	GO:1904992	1	0.997985678	0	1
17098	GO:1904996	1	0.978083263	0	11
17099	GO:1904997	1	0.997997419	0	1
17100	GO:1904998	1	0.997993311	0	1
17101	GO:1904999	1	0.995964399	0	2
17102	GO:1905000	1	0.998013437	0	1
17103	GO:1905006	1	0.995993843	0	2
17104	GO:1905007	1	0.990067562	0	5
17105	GO:1905010	1	0.997987857	0	1
17106	GO:1905025	1	0.997995384	0	1
17107	GO:1905030	1	0.996030612	0	2
17108	GO:1905031	1	0.998013437	0	1
17109	GO:1905035	1	0.997993265	0	1
17110	GO:1905037	1	0.994017863	0	3
17111	GO:1905039	1	0.982155229	0	9
17112	GO:1905040	1	0.997986391	0	1
17113	GO:1905041	1	0.998013437	0	1
17114	GO:1905042	1	0.997982548	0	1
17115	GO:1905045	1	0.997990564	0	1
17116	GO:1905048	1	0.997990564	0	1
17117	GO:1905049	1	0.993987519	0	3
17118	GO:1905050	1	0.991914618	0	4
17119	GO:1905053	1	0.995935819	0	2
17120	GO:1905056	1	0.994051875	0	3
17121	GO:1905059	1	0.998013437	0	1
17122	GO:1905060	1	0.994051339	0	3
17123	GO:1905062	1	0.997989859	0	1
17124	GO:1905064	1	0.990048753	0	5
17125	GO:1905065	1	0.996013519	0	2
17126	GO:1905069	1	0.998007874	0	1
17127	GO:1905072	1	0.994020578	0	3
17128	GO:1905075	1	0.994035153	0	3
17129	GO:1905090	1	0.998013437	0	1
17130	GO:1905098	1	0.992000992	0	4
17131	GO:1905103	1	0.980161468	0	10
17132	GO:1905111	1	0.997941649	0	1
17133	GO:1905114	1	0.994033578	0	3
17134	GO:1905116	1	0.997964949	0	1
17135	GO:1905123	1	0.996023722	0	2
17136	GO:1905128	1	0.998013437	0	1
17137	GO:1905135	1	0.996005257	0	2
17138	GO:1905143	1	0.99797085	0	1
17139	GO:1905144	1	0.99399577	0	3
17140	GO:1905145	1	0.996030731	0	2

17141	GO:1905146	1	0.986038128	0	7
17142	GO:1905150	1	0.987997521	0	6
17143	GO:1905152	1	0.998013437	0	1
17144	GO:1905154	1	0.998013246	0	1
17145	GO:1905162	1	0.995994176	0	2
17146	GO:1905165	1	0.994015865	0	3
17147	GO:1905166	1	0.994014748	0	3
17148	GO:1905167	1	0.994051709	0	3
17149	GO:1905168	1	0.97230311	0	14
17150	GO:1905171	1	0.996028607	0	2
17151	GO:1905172	1	0.994038765	0	3
17152	GO:1905174	1	0.997976263	0	1
17153	GO:1905176	1	0.9979925	0	1
17154	GO:1905179	1	0.995971117	0	2
17155	GO:1905183	1	0.997956524	0	1
17156	GO:1905184	1	0.997956524	0	1
17157	GO:1905188	1	0.997976841	0	1
17158	GO:1905198	1	0.991960222	0	4
17159	GO:1905199	1	0.997986375	0	1
17160	GO:1905202	1	0.995995346	0	2
17161	GO:1905203	1	0.998013437	0	1
17162	GO:1905205	1	0.992003664	0	4
17163	GO:1905206	1	0.9980062	0	1
17164	GO:1905208	1	0.997987593	0	1
17165	GO:1905209	1	0.998011182	0	1
17166	GO:1905216	1	0.998013357	0	1
17167	GO:1905221	1	0.996020975	0	2
17168	GO:1905222	1	0.992028739	0	4
17169	GO:1905223	1	0.996030731	0	2
17170	GO:1905224	1	0.996020765	0	2
17171	GO:1905226	1	0.99796581	0	1
17172	GO:1905229	1	0.997965083	0	1
17173	GO:1905232	1	0.99002393	0	5
17174	GO:1905240	1	0.998012928	0	1
17175	GO:1905242	1	0.998000066	0	1
17176	GO:1905243	1	0.995984842	0	2
17177	GO:1905244	1	0.99001703	0	5
17178	GO:1905246	1	0.997999481	0	1
17179	GO:1905247	1	0.997983681	0	1
17180	GO:1905259	1	0.995933625	0	2
17181	GO:1905267	1	0.997963441	0	1
17182	GO:1905273	1	0.993945832	0	3
17183	GO:1905274	1	0.988080606	0	6
17184	GO:1905278	1	0.992064608	0	4
17185	GO:1905279	1	0.992054062	0	4

17186	GO:1905280	1	0.997994297	0	1
17187	GO:1905281	1	0.995985075	0	2
17188	GO:1905285	1	0.998003307	0	1
17189	GO:1905286	1	0.997984299	0	1
17190	GO:1905289	1	0.996030731	0	2
17191	GO:1905291	1	0.997961288	0	1
17192	GO:1905302	1	0.998011417	0	1
17193	GO:1905303	1	0.996015409	0	2
17194	GO:1905305	1	0.998013437	0	1
17195	GO:1905312	1	0.994007709	0	3
17196	GO:1905313	1	0.997981889	0	1
17197	GO:1905314	1	0.998013437	0	1
17198	GO:1905317	1	0.99801192	0	1
17199	GO:1905322	1	0.993958768	0	3
17200	GO:1905323	1	0.991966418	0	4
17201	GO:1905327	1	0.997971213	0	1
17202	GO:1905332	1	0.997996294	0	1
17203	GO:1905333	1	0.995931334	0	2
17204	GO:1905337	1	0.993991478	0	3
17205	GO:1905341	1	0.99798941	0	1
17206	GO:1905342	1	0.99195941	0	4
17207	GO:1905344	1	0.995966716	0	2
17208	GO:1905347	1	0.998013437	0	1
17209	GO:1905349	1	0.997991478	0	1
17210	GO:1905355	1	0.998013114	0	1
17211	GO:1905364	1	0.997976331	0	1
17212	GO:1905366	1	0.99597693	0	2
17213	GO:1905368	1	0.997991284	0	1
17214	GO:1905370	1	0.997985159	0	1
17215	GO:1905373	1	0.995987295	0	2
17216	GO:1905377	1	0.998011582	0	1
17217	GO:1905381	1	0.998003825	0	1
17218	GO:1905382	1	0.993978336	0	3
17219	GO:1905386	1	0.995971015	0	2
17220	GO:1905394	1	0.984085892	0	8
17221	GO:1905395	1	0.993964747	0	3
17222	GO:1905396	1	0.997966752	0	1
17223	GO:1905403	1	0.995995649	0	2
17224	GO:1905404	1	0.997970594	0	1
17225	GO:1905406	1	0.998013437	0	1
17226	GO:1905408	1	0.995979402	0	2
17227	GO:1905413	1	0.998011431	0	1
17228	GO:1905426	1	0.995972907	0	2
17229	GO:1905429	1	0.99800985	0	1
17230	GO:1905430	1	0.99598632	0	2

17231	GO:1905437	1	0.997990564	0	1
17232	GO:1905438	1	0.997969178	0	1
17233	GO:1905445	1	0.995975104	0	2
17234	GO:1905448	1	0.991920836	0	4
17235	GO:1905450	1	0.996030601	0	2
17236	GO:1905451	1	0.990053075	0	5
17237	GO:1905453	1	0.997997716	0	1
17238	GO:1905454	1	0.997985277	0	1
17239	GO:1905456	1	0.997997716	0	1
17240	GO:1905460	1	0.996030659	0	2
17241	GO:1905461	1	0.986034436	0	7
17242	GO:1905463	1	0.998013436	0	1
17243	GO:1905469	1	0.998013246	0	1
17244	GO:1905474	1	0.997969788	0	1
17245	GO:1905475	1	0.990031717	0	5
17246	GO:1905477	1	0.988008639	0	6
17247	GO:1905492	1	0.998002516	0	1
17248	GO:1905502	1	0.993991913	0	3
17249	GO:1905505	1	0.99797487	0	1
17250	GO:1905513	1	0.997987639	0	1
17251	GO:1905515	1	0.911734414	0	46
17252	GO:1905516	1	0.994008251	0	3
17253	GO:1905517	1	0.994003868	0	3
17254	GO:1905520	1	0.996030006	0	2
17255	GO:1905521	1	0.995938752	0	2
17256	GO:1905522	1	0.993973421	0	3
17257	GO:1905523	1	0.995991917	0	2
17258	GO:1905524	1	0.996007903	0	2
17259	GO:1905526	1	0.997982067	0	1
17260	GO:1905528	1	0.998011282	0	1
17261	GO:1905537	1	0.998013232	0	1
17262	GO:1905538	1	0.988065867	0	6
17263	GO:1905549	1	0.997972367	0	1
17264	GO:1905552	1	0.995954937	0	2
17265	GO:1905553	1	0.997966837	0	1
17266	GO:1905555	1	0.993955941	0	3
17267	GO:1905563	1	0.990009639	0	5
17268	GO:1905564	1	0.970388933	0	15
17269	GO:1905572	1	0.997991842	0	1
17270	GO:1905573	1	0.993968968	0	3
17271	GO:1905574	1	0.997991842	0	1
17272	GO:1905575	1	0.997991842	0	1
17273	GO:1905576	1	0.995972975	0	2
17274	GO:1905577	1	0.997991842	0	1
17275	GO:1905580	1	0.997985733	0	1

17276	GO:1905581	1	0.993939056	0	3
17277	GO:1905584	1	0.998003793	0	1
17278	GO:1905589	1	0.997987857	0	1
17279	GO:1905590	1	0.997996355	0	1
17280	GO:1905594	1	0.997963052	0	1
17281	GO:1905596	1	0.995949226	0	2
17282	GO:1905597	1	0.997972358	0	1
17283	GO:1905598	1	0.99009323	0	5
17284	GO:1905599	1	0.991930593	0	4
17285	GO:1905601	1	0.993970528	0	3
17286	GO:1905602	1	0.995969738	0	2
17287	GO:1905604	1	0.994016771	0	3
17288	GO:1905605	1	0.990054994	0	5
17289	GO:1905606	1	0.953079268	0	24
17290	GO:1905609	1	0.997978736	0	1
17291	GO:1905612	1	0.998013232	0	1
17292	GO:1905618	1	0.986089849	0	7
17293	GO:1905634	1	0.994044709	0	3
17294	GO:1905636	1	0.991952112	0	4
17295	GO:1905642	1	0.997990564	0	1
17296	GO:1905643	1	0.996001807	0	2
17297	GO:1905647	1	0.998013349	0	1
17298	GO:1905653	1	0.993944944	0	3
17299	GO:1905662	1	0.997984449	0	1
17300	GO:1905663	1	0.995989273	0	2
17301	GO:1905664	1	0.995974058	0	2
17302	GO:1905665	1	0.998003243	0	1
17303	GO:1905667	1	0.998006721	0	1
17304	GO:1905668	1	0.991974244	0	4
17305	GO:1905671	1	0.992004324	0	4
17306	GO:1905672	1	0.997981065	0	1
17307	GO:1905673	1	0.997983681	0	1
17308	GO:1905675	1	0.99594857	0	2
17309	GO:1905676	1	0.997969861	0	1
17310	GO:1905684	1	0.994001321	0	3
17311	GO:1905689	1	0.998013437	0	1
17312	GO:1905696	1	0.998013232	0	1
17313	GO:1905698	1	0.998011759	0	1
17314	GO:1905700	1	0.995886938	0	2
17315	GO:1905704	1	0.996013192	0	2
17316	GO:1905705	1	0.998013437	0	1
17317	GO:1905706	1	0.993980067	0	3
17318	GO:1905709	1	0.996016313	0	2
17319	GO:1905710	1	0.998003887	0	1
17320	GO:1905711	1	0.99800547	0	1

17321	GO:1905716	1	0.997987297	0	1
17322	GO:1905719	1	0.984103442	0	8
17323	GO:1905720	1	0.998013437	0	1
17324	GO:1905721	1	0.993967449	0	3
17325	GO:1905737	1	0.995996458	0	2
17326	GO:1905751	1	0.996025048	0	2
17327	GO:1905762	1	0.995985075	0	2
17328	GO:1905765	1	0.994004742	0	3
17329	GO:1905768	1	0.998013437	0	1
17330	GO:1905772	1	0.997996294	0	1
17331	GO:1905773	1	0.994044845	0	3
17332	GO:1905774	1	0.998008429	0	1
17333	GO:1905775	1	0.995972347	0	2
17334	GO:1905776	1	0.99395113	0	3
17335	GO:1905778	1	0.995990119	0	2
17336	GO:1905786	1	0.993980034	0	3
17337	GO:1905788	1	0.997989828	0	1
17338	GO:1905793	1	0.996029996	0	2
17339	GO:1905795	1	0.998013437	0	1
17340	GO:1905799	1	0.998012708	0	1
17341	GO:1905802	1	0.99799209	0	1
17342	GO:1905818	1	0.995966913	0	2
17343	GO:1905820	1	0.995999437	0	2
17344	GO:1905828	1	0.997990649	0	1
17345	GO:1905832	1	0.990043452	0	5
17346	GO:1905833	1	0.998013353	0	1
17347	GO:1905835	1	0.997974496	0	1
17348	GO:1905839	1	0.995990119	0	2
17349	GO:1905843	1	0.998013437	0	1
17350	GO:1905855	1	0.997963771	0	1
17351	GO:1905856	1	0.997988013	0	1
17352	GO:1905860	1	0.997963771	0	1
17353	GO:1905867	1	0.989940638	0	5
17354	GO:1905869	1	0.997990564	0	1
17355	GO:1905870	1	0.989997488	0	5
17356	GO:1905872	1	0.997990564	0	1
17357	GO:1905885	1	0.99600089	0	2
17358	GO:1905890	1	0.997963771	0	1
17359	GO:1905892	1	0.997991997	0	1
17360	GO:1905895	1	0.997991997	0	1
17361	GO:1905897	1	0.993940078	0	3
17362	GO:1905898	1	0.997971783	0	1
17363	GO:1905906	1	0.995949016	0	2
17364	GO:1905907	1	0.977884838	0	11
17365	GO:1905908	1	0.991994163	0	4

17366	GO:1905912	1	0.997987047	0	1
17367	GO:1905913	1	0.997975956	0	1
17368	GO:1905920	1	0.995940254	0	2
17369	GO:1905931	1	0.996028542	0	2
17370	GO:1905932	1	0.997960735	0	1
17371	GO:1905934	1	0.997968974	0	1
17372	GO:1905956	1	0.996000388	0	2
17373	GO:1990000	1	0.854388557	0	78
17374	GO:1990001	1	0.993969125	0	3
17375	GO:1990002	1	0.997969391	0	1
17376	GO:1990003	1	0.996007648	0	2
17377	GO:1990005	1	0.997990564	0	1
17378	GO:1990008	1	0.997973681	0	1
17379	GO:1990023	1	0.974327916	0	13
17380	GO:1990026	1	0.996018932	0	2
17381	GO:1990029	1	0.997996859	0	1
17382	GO:1990030	1	0.995996728	0	2
17383	GO:1990031	1	0.997996814	0	1
17384	GO:1990032	1	0.998011102	0	1
17385	GO:1990034	1	0.990082452	0	5
17386	GO:1990036	1	0.99402401	0	3
17387	GO:1990037	1	0.993945157	0	3
17388	GO:1990038	1	0.997977911	0	1
17389	GO:1990044	1	0.997971846	0	1
17390	GO:1990046	1	0.99593595	0	2
17391	GO:1990047	1	0.997983155	0	1
17392	GO:1990048	1	0.990082986	0	5
17393	GO:1990049	1	0.990099069	0	5
17394	GO:1990050	1	0.985926056	0	7
17395	GO:1990051	1	0.989987792	0	5
17396	GO:1990070	1	0.997955602	0	1
17397	GO:1990071	1	0.995973008	0	2
17398	GO:1990072	1	0.993948699	0	3
17399	GO:1990074	1	0.993994832	0	3
17400	GO:1990075	1	0.988058615	0	6
17401	GO:1990079	1	0.99601524	0	2
17402	GO:1990086	1	0.992025196	0	4
17403	GO:1990089	1	0.993994784	0	3
17404	GO:1990090	1	0.930370425	0	36
17405	GO:1990092	1	0.99799807	0	1
17406	GO:1990108	1	0.994024093	0	3
17407	GO:1990111	1	0.991925219	0	4
17408	GO:1990112	1	0.993976017	0	3
17409	GO:1990114	1	0.99797251	0	1
17410	GO:1990116	1	0.995985075	0	2

17411	GO:1990117	1	0.997956473	0	1
17412	GO:1990120	1	0.994038944	0	3
17413	GO:1990124	1	0.978213555	0	11
17414	GO:1990130	1	0.988006603	0	6
17415	GO:1990131	1	0.991959025	0	4
17416	GO:1990134	1	0.99801296	0	1
17417	GO:1990136	1	0.995979374	0	2
17418	GO:1990138	1	0.958849516	0	21
17419	GO:1990144	1	0.995962507	0	2
17420	GO:1990145	1	0.996001532	0	2
17421	GO:1990147	1	0.997998991	0	1
17422	GO:1990164	1	0.997996776	0	1
17423	GO:1990165	1	0.997978951	0	1
17424	GO:1990166	1	0.989967188	0	5
17425	GO:1990167	1	0.994011797	0	3
17426	GO:1990168	1	0.992033588	0	4
17427	GO:1990172	1	0.998013437	0	1
17428	GO:1990173	1	0.996007521	0	2
17429	GO:1990174	1	0.996023388	0	2
17430	GO:1990175	1	0.996024183	0	2
17431	GO:1990180	1	0.993944934	0	3
17432	GO:1990182	1	0.991940369	0	4
17433	GO:1990184	1	0.995998228	0	2
17434	GO:1990189	1	0.995961387	0	2
17435	GO:1990190	1	0.997957984	0	1
17436	GO:1990204	1	0.998001045	0	1
17437	GO:1990221	1	0.995986927	0	2
17438	GO:1990226	1	0.980127763	0	10
17439	GO:1990227	1	0.997990564	0	1
17440	GO:1990234	1	0.989919103	0	5
17441	GO:1990238	1	0.998013437	0	1
17442	GO:1990239	1	0.988048236	0	6
17443	GO:1990244	1	0.997990564	0	1
17444	GO:1990245	1	0.997990564	0	1
17445	GO:1990246	1	0.988002428	0	6
17446	GO:1990247	1	0.986113149	0	7
17447	GO:1990248	1	0.997988013	0	1
17448	GO:1990253	1	0.980153213	0	10
17449	GO:1990254	1	0.988035943	0	6
17450	GO:1990256	1	0.998013437	0	1
17451	GO:1990258	1	0.99193607	0	4
17452	GO:1990259	1	0.995928961	0	2
17453	GO:1990261	1	0.997990564	0	1
17454	GO:1990264	1	0.986110501	0	7
17455	GO:1990266	1	0.984053548	0	8

17456	GO:1990267	1	0.99798074	0	1
17457	GO:1990269	1	0.990004309	0	5
17458	GO:1990275	1	0.997992608	0	1
17459	GO:1990276	1	0.997990433	0	1
17460	GO:1990280	1	0.998013437	0	1
17461	GO:1990298	1	0.997981122	0	1
17462	GO:1990314	1	0.982121865	0	9
17463	GO:1990316	1	0.984129399	0	8
17464	GO:1990332	1	0.998007771	0	1
17465	GO:1990349	1	0.989986431	0	5
17466	GO:1990356	1	0.997964216	0	1
17467	GO:1990357	1	0.990017511	0	5
17468	GO:1990379	1	0.989950703	0	5
17469	GO:1990380	1	0.970442908	0	15
17470	GO:1990381	1	0.958651119	0	21
17471	GO:1990384	1	0.994013983	0	3
17472	GO:1990385	1	0.998008918	0	1
17473	GO:1990386	1	0.997978237	0	1
17474	GO:1990390	1	0.998001265	0	1
17475	GO:1990391	1	0.990088042	0	5
17476	GO:1990393	1	0.99005673	0	5
17477	GO:1990400	1	0.997986101	0	1
17478	GO:1990401	1	0.995980992	0	2
17479	GO:1990402	1	0.995951579	0	2
17480	GO:1990403	1	0.978174866	0	11
17481	GO:1990404	1	0.968463551	0	16
17482	GO:1990405	1	0.98604896	0	7
17483	GO:1990406	1	0.995975243	0	2
17484	GO:1990407	1	0.997958386	0	1
17485	GO:1990408	1	0.995975243	0	2
17486	GO:1990409	1	0.995972769	0	2
17487	GO:1990410	1	0.993949663	0	3
17488	GO:1990414	1	0.986044935	0	7
17489	GO:1990416	1	0.980202136	0	10
17490	GO:1990418	1	0.993971957	0	3
17491	GO:1990422	1	0.997958946	0	1
17492	GO:1990423	1	0.994015825	0	3
17493	GO:1990425	1	0.994004033	0	3
17494	GO:1990426	1	0.995999878	0	2
17495	GO:1990428	1	0.993998536	0	3
17496	GO:1990429	1	0.993989872	0	3
17497	GO:1990430	1	0.994026006	0	3
17498	GO:1990435	1	0.998013437	0	1
17499	GO:1990438	1	0.99798234	0	1
17500	GO:1990439	1	0.997993566	0	1

17501	GO:1990440	1	0.978050779	0	11
17502	GO:1990441	1	0.991978949	0	4
17503	GO:1990442	1	0.997958446	0	1
17504	GO:1990443	1	0.995981853	0	2
17505	GO:1990444	1	0.993998876	0	3
17506	GO:1990446	1	0.989846299	0	5
17507	GO:1990447	1	0.995942904	0	2
17508	GO:1990448	1	0.998013437	0	1
17509	GO:1990450	1	0.997980087	0	1
17510	GO:1990451	1	0.997986031	0	1
17511	GO:1990452	1	0.994004839	0	3
17512	GO:1990454	1	0.980126471	0	10
17513	GO:1990456	1	0.98610588	0	7
17514	GO:1990459	1	0.980081448	0	10
17515	GO:1990460	1	0.992004612	0	4
17516	GO:1990462	1	0.996006202	0	2
17517	GO:1990466	1	0.998011212	0	1
17518	GO:1990467	1	0.997981065	0	1
17519	GO:1990468	1	0.997981065	0	1
17520	GO:1990478	1	0.99797487	0	1
17521	GO:1990481	1	0.985966598	0	7
17522	GO:1990498	1	0.974223172	0	13
17523	GO:1990502	1	0.994040101	0	3
17524	GO:1990504	1	0.996030647	0	2
17525	GO:1990511	1	0.992022905	0	4
17526	GO:1990513	1	0.996025057	0	2
17527	GO:1990518	1	0.992035462	0	4
17528	GO:1990519	1	0.995985008	0	2
17529	GO:1990523	1	0.991995716	0	4
17530	GO:1990529	1	0.998010119	0	1
17531	GO:1990531	1	0.986172333	0	7
17532	GO:1990535	1	0.982200913	0	9
17533	GO:1990539	1	0.997983808	0	1
17534	GO:1990540	1	0.997997916	0	1
17535	GO:1990542	1	0.98599399	0	7
17536	GO:1990544	1	0.989940812	0	5
17537	GO:1990547	1	0.997973168	0	1
17538	GO:1990548	1	0.997994758	0	1
17539	GO:1990549	1	0.995985075	0	2
17540	GO:1990550	1	0.997992275	0	1
17541	GO:1990565	1	0.995964324	0	2
17542	GO:1990569	1	0.992003772	0	4
17543	GO:1990572	1	0.99800762	0	1
17544	GO:1990573	1	0.934104547	0	34
17545	GO:1990575	1	0.993972411	0	3

17546	GO:1990578	1	0.991951721	0	4
17547	GO:1990579	1	0.998007771	0	1
17548	GO:1990583	1	0.997978237	0	1
17549	GO:1990584	1	0.993886467	0	3
17550	GO:1990589	1	0.991974949	0	4
17551	GO:1990590	1	0.995963452	0	2
17552	GO:1990592	1	0.989940016	0	5
17553	GO:1990597	1	0.996022746	0	2
17554	GO:1990599	1	0.994004742	0	3
17555	GO:1990603	1	0.997990564	0	1
17556	GO:1990604	1	0.992031015	0	4
17557	GO:1990613	1	0.995996499	0	2
17558	GO:1990617	1	0.995936266	0	2
17559	GO:1990619	1	0.99598226	0	2
17560	GO:1990622	1	0.995941825	0	2
17561	GO:1990630	1	0.996002248	0	2
17562	GO:1990635	1	0.988057297	0	6
17563	GO:1990637	1	0.995991052	0	2
17564	GO:1990641	1	0.993949209	0	3
17565	GO:1990646	1	0.995982935	0	2
17566	GO:1990654	1	0.998012707	0	1
17567	GO:1990665	1	0.995937705	0	2
17568	GO:1990666	1	0.996020708	0	2
17569	GO:1990667	1	0.995979875	0	2
17570	GO:1990668	1	0.987983108	0	6
17571	GO:1990675	1	0.998013437	0	1
17572	GO:1990676	1	0.998013437	0	1
17573	GO:1990678	1	0.998009617	0	1
17574	GO:1990682	1	0.995997046	0	2
17575	GO:1990683	1	0.995989759	0	2
17576	GO:1990696	1	0.99199718	0	4
17577	GO:1990697	1	0.997982227	0	1
17578	GO:1990698	1	0.995986053	0	2
17579	GO:1990699	1	0.997982227	0	1
17580	GO:1990700	1	0.998007009	0	1
17581	GO:1990701	1	0.997990564	0	1
17582	GO:1990705	1	0.998013437	0	1
17583	GO:1990708	1	0.992052664	0	4
17584	GO:1990709	1	0.998010498	0	1
17585	GO:1990712	1	0.986034122	0	7
17586	GO:1990715	1	0.995997806	0	2
17587	GO:1990716	1	0.991995681	0	4
17588	GO:1990718	1	0.998013437	0	1
17589	GO:1990723	1	0.996012179	0	2
17590	GO:1990726	1	0.989845216	0	5

17591	GO:1990730	1	0.996012519	0	2
17592	GO:1990731	1	0.998003243	0	1
17593	GO:1990737	1	0.994006433	0	3
17594	GO:1990738	1	0.997990564	0	1
17595	GO:1990742	1	0.997949301	0	1
17596	GO:1990743	1	0.992002585	0	4
17597	GO:1990744	1	0.997977936	0	1
17598	GO:1990745	1	0.990018148	0	5
17599	GO:1990748	1	0.996004631	0	2
17600	GO:1990752	1	0.990006575	0	5
17601	GO:1990755	1	0.997990564	0	1
17602	GO:1990756	1	0.970157422	0	15
17603	GO:1990757	1	0.992005159	0	4
17604	GO:1990758	1	0.997975801	0	1
17605	GO:1990760	1	0.994033692	0	3
17606	GO:1990761	1	0.994015301	0	3
17607	GO:1990763	1	0.982038493	0	9
17608	GO:1990764	1	0.997988075	0	1
17609	GO:1990769	1	0.991991854	0	4
17610	GO:1990771	1	0.998013437	0	1
17611	GO:1990776	1	0.990100183	0	5
17612	GO:1990777	1	0.997963771	0	1
17613	GO:1990778	1	0.996025353	0	2
17614	GO:1990780	1	0.998008048	0	1
17615	GO:1990782	1	0.941668939	0	30
17616	GO:1990785	1	0.994036846	0	3
17617	GO:1990786	1	0.996002406	0	2
17618	GO:1990787	1	0.998013437	0	1
17619	GO:1990791	1	0.991999203	0	4
17620	GO:1990792	1	0.997999639	0	1
17621	GO:1990809	1	0.991974112	0	4
17622	GO:1990811	1	0.997990564	0	1
17623	GO:1990812	1	0.992036819	0	4
17624	GO:1990814	1	0.997968136	0	1
17625	GO:1990817	1	0.990016068	0	5
17626	GO:1990822	1	0.988026709	0	6
17627	GO:1990823	1	0.997999867	0	1
17628	GO:1990825	1	0.976149889	0	12
17629	GO:1990828	1	0.997981195	0	1
17630	GO:1990829	1	0.997977894	0	1
17631	GO:1990830	1	0.844625313	0	84
17632	GO:1990832	1	0.997979166	0	1
17633	GO:1990833	1	0.997979166	0	1
17634	GO:1990834	1	0.989989983	0	5
17635	GO:1990836	1	0.998008272	0	1

17637	GO:1990838	1	0.997990564	0	1
17638	GO:1990839	1	0.998009898	0	1
17640	GO:1990845	1	0.982131481	0	9
17641	GO:1990851	1	0.994031513	0	3
17642	GO:1990858	1	0.997984457	0	1
17643	GO:1990859	1	0.995972031	0	2
17644	GO:1990869	1	0.972364652	0	14
17645	GO:1990876	1	0.998013437	0	1
17646	GO:1990879	1	0.993983524	0	3
17647	GO:1990883	1	0.998007125	0	1
17648	GO:1990889	1	0.995980007	0	2
17649	GO:1990890	1	0.997973646	0	1
17651	GO:1990907	1	0.974337979	0	13
17652	GO:1990909	1	0.978255275	0	11
17653	GO:1990910	1	0.991964384	0	4
17654	GO:1990911	1	0.993968836	0	3
17655	GO:1990913	1	0.991984993	0	4
17656	GO:1990917	1	0.997987328	0	1
17657	GO:1990918	1	0.994013408	0	3
17658	GO:1990923	1	0.993983194	0	3
17659	GO:1990926	1	0.996008783	0	2
17660	GO:1990927	1	0.994030377	0	3
17661	GO:1990928	1	0.993989062	0	3
17662	GO:1990930	1	0.997971213	0	1
17663	GO:1990931	1	0.99601458	0	2
17664	GO:1990932	1	0.995913462	0	2
17665	GO:1990935	1	0.993923802	0	3
17666	GO:1990938	1	0.99800761	0	1
17667	GO:1990939	1	0.921390743	0	41
17668	GO:1990948	1	0.981790951	0	9
17669	GO:1990953	1	0.997990564	0	1
17670	GO:1990955	1	0.998008429	0	1
17671	GO:1990959	1	0.995996793	0	2
17672	GO:1990960	1	0.998003662	0	1
17673	GO:1990961	1	0.982116329	0	9
17674	GO:1990962	1	0.990064919	0	5
17675	GO:1990963	1	0.997978678	0	1
17676	GO:1990966	1	0.992010409	0	4
17677	GO:1990968	1	0.998013437	0	1
17678	GO:1990969	1	0.998013437	0	1
17679	GO:1990971	1	0.998006897	0	1
17680	GO:1990983	1	0.997987966	0	1
17681	GO:1990984	1	0.996001845	0	2
17682	GO:2000001	1	0.982068079	0	9
17683	GO:2000002	1	0.989987586	0	5

17684	GO:2000003	1	0.997990564	0	1
17685	GO:2000005	1	0.997974922	0	1
17686	GO:2000007	1	0.997974922	0	1
17687	GO:2000008	1	0.980160466	0	10
17688	GO:2000009	1	0.978144691	0	11
17689	GO:2000010	1	0.968414677	0	16
17690	GO:2000011	1	0.998002634	0	1
17691	GO:2000016	1	0.995938798	0	2
17692	GO:2000017	1	0.995998357	0	2
17693	GO:2000019	1	0.998006585	0	1
17694	GO:2000020	1	0.988058539	0	6
17695	GO:2000027	1	0.99797054	0	1
17696	GO:2000035	1	0.984043858	0	8
17697	GO:2000036	1	0.974319174	0	13
17698	GO:2000041	1	0.995993327	0	2
17699	GO:2000042	1	0.962591251	0	19
17700	GO:2000045	1	0.966371826	0	17
17701	GO:2000048	1	0.980116614	0	10
17702	GO:2000049	1	0.978182597	0	11
17703	GO:2000050	1	0.994021949	0	3
17704	GO:2000051	1	0.994048497	0	3
17705	GO:2000052	1	0.988003223	0	6
17706	GO:2000054	1	0.998010994	0	1
17707	GO:2000058	1	0.992053192	0	4
17708	GO:2000059	1	0.966265077	0	17
17709	GO:2000060	1	0.964587876	0	18
17710	GO:2000062	1	0.997971213	0	1
17711	GO:2000063	1	0.997971213	0	1
17712	GO:2000064	1	0.996029166	0	2
17713	GO:2000065	1	0.994002632	0	3
17714	GO:2000066	1	0.998006585	0	1
17715	GO:2000074	1	0.990029555	0	5
17716	GO:2000077	1	0.995998987	0	2
17717	GO:2000078	1	0.993933008	0	3
17718	GO:2000080	1	0.998010994	0	1
17719	GO:2000081	1	0.997993311	0	1
17720	GO:2000095	1	0.990027988	0	5
17721	GO:2000096	1	0.98408883	0	8
17722	GO:2000097	1	0.997986985	0	1
17723	GO:2000098	1	0.995963662	0	2
17724	GO:2000103	1	0.997993389	0	1
17725	GO:2000104	1	0.997964486	0	1
17726	GO:2000105	1	0.99798941	0	1
17727	GO:2000107	1	0.995986748	0	2
17728	GO:2000109	1	0.997985301	0	1

17730	GO:2000111	1	0.99399453	0	3
17731	GO:2000114	1	0.964643549	0	18
17732	GO:2000116	1	0.993984586	0	3
17733	GO:2000117	1	0.991924408	0	4
17734	GO:2000118	1	0.998008812	0	1
17735	GO:2000120	1	0.993944312	0	3
17736	GO:2000121	1	0.988010313	0	6
17737	GO:2000124	1	0.994019974	0	3
17738	GO:2000134	1	0.920942936	0	41
17739	GO:2000137	1	0.995991099	0	2
17740	GO:2000138	1	0.99396532	0	3
17741	GO:2000143	1	0.997985379	0	1
17742	GO:2000144	1	0.990049447	0	5
17743	GO:2000145	1	0.939701976	0	31
17744	GO:2000146	1	0.974119824	0	13
17745	GO:2000147	1	0.964466504	0	18
17746	GO:2000152	1	0.99800761	0	1
17747	GO:2000156	1	0.99600261	0	2
17748	GO:2000157	1	0.995928947	0	2
17749	GO:2000158	1	0.995977851	0	2
17750	GO:2000167	1	0.998013355	0	1
17751	GO:2000170	1	0.99397297	0	3
17752	GO:2000171	1	0.98611152	0	7
17753	GO:2000172	1	0.994007562	0	3
17754	GO:2000173	1	0.99800714	0	1
17755	GO:2000176	1	0.998007319	0	1
17756	GO:2000177	1	0.980126311	0	10
17757	GO:2000178	1	0.978166757	0	11
17758	GO:2000179	1	0.956737573	0	22
17759	GO:2000180	1	0.998006585	0	1
17760	GO:2000182	1	0.996012162	0	2
17761	GO:2000184	1	0.99598628	0	2
17762	GO:2000187	1	0.997982764	0	1
17763	GO:2000191	1	0.998013437	0	1
17764	GO:2000192	1	0.997954992	0	1
17765	GO:2000193	1	0.997990564	0	1
17766	GO:2000194	1	0.997957944	0	1
17767	GO:2000195	1	0.998011079	0	1
17768	GO:2000208	1	0.997995886	0	1
17769	GO:2000209	1	0.998013433	0	1
17770	GO:2000210	1	0.987972914	0	6
17771	GO:2000211	1	0.998013437	0	1
17772	GO:2000212	1	0.998012964	0	1
17773	GO:2000224	1	0.997964698	0	1
17774	GO:2000225	1	0.995971411	0	2

17775	GO:2000227	1	0.997968974	0	1
17776	GO:2000230	1	0.997976203	0	1
17777	GO:2000232	1	0.996008831	0	2
17778	GO:2000233	1	0.994029665	0	3
17779	GO:2000234	1	0.980069014	0	10
17780	GO:2000249	1	0.96255848	0	19
17781	GO:2000250	1	0.998013437	0	1
17782	GO:2000251	1	0.964315181	0	18
17783	GO:2000252	1	0.99389984	0	3
17784	GO:2000253	1	0.989973053	0	5
17785	GO:2000255	1	0.997958726	0	1
17786	GO:2000256	1	0.997959843	0	1
17787	GO:2000264	1	0.995975413	0	2
17788	GO:2000266	1	0.997971684	0	1
17789	GO:2000267	1	0.990003854	0	5
17790	GO:2000269	1	0.993964724	0	3
17791	GO:2000270	1	0.986081788	0	7
17792	GO:2000271	1	0.980091796	0	10
17793	GO:2000272	1	0.943165685	0	29
17794	GO:2000273	1	0.980142756	0	10
17795	GO:2000276	1	0.997981809	0	1
17796	GO:2000277	1	0.997958946	0	1
17797	GO:2000278	1	0.993976754	0	3
17798	GO:2000279	1	0.974184416	0	13
17799	GO:2000286	1	0.993993755	0	3
17800	GO:2000287	1	0.99798258	0	1
17801	GO:2000288	1	0.991997319	0	4
17802	GO:2000291	1	0.996007903	0	2
17803	GO:2000296	1	0.995922807	0	2
17804	GO:2000297	1	0.99602625	0	2
17805	GO:2000298	1	0.996002327	0	2
17806	GO:2000299	1	0.994023868	0	3
17807	GO:2000300	1	0.915509365	0	44
17808	GO:2000301	1	0.991984509	0	4
17809	GO:2000302	1	0.994047466	0	3
17810	GO:2000303	1	0.993953385	0	3
17811	GO:2000304	1	0.97811415	0	11
17812	GO:2000308	1	0.997984615	0	1
17813	GO:2000309	1	0.997996656	0	1
17814	GO:2000310	1	0.951318012	0	25
17815	GO:2000311	1	0.966487666	0	17
17816	GO:2000312	1	0.99602105	0	2
17817	GO:2000313	1	0.997976723	0	1
17818	GO:2000314	1	0.997972813	0	1
17819	GO:2000317	1	0.995997973	0	2

17820	GO:2000318	1	0.989948114	0	5
17821	GO:2000320	1	0.988050232	0	6
17822	GO:2000321	1	0.994006623	0	3
17823	GO:2000323	1	0.988058353	0	6
17824	GO:2000324	1	0.995986519	0	2
17825	GO:2000329	1	0.997996355	0	1
17826	GO:2000330	1	0.993943003	0	3
17827	GO:2000331	1	0.997998802	0	1
17828	GO:2000334	1	0.995956661	0	2
17829	GO:2000338	1	0.997989906	0	1
17830	GO:2000340	1	0.993984108	0	3
17831	GO:2000341	1	0.992003675	0	4
17833	GO:2000343	1	0.9800746	0	10
17834	GO:2000344	1	0.984015483	0	8
17835	GO:2000345	1	0.996014953	0	2
17836	GO:2000346	1	0.991983642	0	4
17837	GO:2000347	1	0.980048331	0	10
17838	GO:2000348	1	0.994003215	0	3
17839	GO:2000349	1	0.998010813	0	1
17840	GO:2000350	1	0.997961288	0	1
17841	GO:2000351	1	0.995989114	0	2
17842	GO:2000352	1	0.951055602	0	25
17843	GO:2000353	1	0.968328343	0	16
17844	GO:2000354	1	0.995990657	0	2
17845	GO:2000355	1	0.995960514	0	2
17846	GO:2000357	1	0.997971213	0	1
17847	GO:2000358	1	0.997971213	0	1
17848	GO:2000359	1	0.997956827	0	1
17849	GO:2000360	1	0.993928609	0	3
17850	GO:2000363	1	0.99402862	0	3
17851	GO:2000367	1	0.998006747	0	1
17852	GO:2000368	1	0.99594947	0	2
17853	GO:2000369	1	0.980193023	0	10
17854	GO:2000370	1	0.990083729	0	5
17855	GO:2000373	1	0.988065544	0	6
17856	GO:2000374	1	0.997979323	0	1
17857	GO:2000376	1	0.995915248	0	2
17858	GO:2000377	1	0.956666227	0	22
17859	GO:2000378	1	0.956636045	0	22
17860	GO:2000379	1	0.937512737	0	32
17861	GO:2000380	1	0.998001565	0	1
17862	GO:2000381	1	0.996029108	0	2
17863	GO:2000382	1	0.995927855	0	2
17864	GO:2000384	1	0.997956877	0	1
17865	GO:2000386	1	0.995979872	0	2

17866	GO:2000388	1	0.997967836	0	1
17867	GO:2000391	1	0.987974637	0	6
17868	GO:2000392	1	0.99400518	0	3
17869	GO:2000393	1	0.99600761	0	2
17870	GO:2000394	1	0.986087579	0	7
17871	GO:2000397	1	0.995935013	0	2
17872	GO:2000401	1	0.992025319	0	4
17873	GO:2000402	1	0.990036661	0	5
17874	GO:2000403	1	0.995945556	0	2
17875	GO:2000404	1	0.988003593	0	6
17876	GO:2000405	1	0.989961084	0	5
17878	GO:2000408	1	0.997998379	0	1
17879	GO:2000410	1	0.997980488	0	1
17880	GO:2000415	1	0.997996656	0	1
17881	GO:2000418	1	0.995947816	0	2
17882	GO:2000420	1	0.998000474	0	1
17883	GO:2000424	1	0.997988712	0	1
17884	GO:2000425	1	0.997992902	0	1
17885	GO:2000426	1	0.995979122	0	2
17886	GO:2000427	1	0.988017395	0	6
17887	GO:2000431	1	0.998006856	0	1
17888	GO:2000434	1	0.988076293	0	6
17889	GO:2000435	1	0.989899534	0	5
17890	GO:2000436	1	0.99004557	0	5
17891	GO:2000438	1	0.998013437	0	1
17892	GO:2000439	1	0.99597827	0	2
17893	GO:2000446	1	0.998010268	0	1
17894	GO:2000448	1	0.995945257	0	2
17895	GO:2000452	1	0.997977348	0	1
17896	GO:2000454	1	0.997976075	0	1
17897	GO:2000458	1	0.997974887	0	1
17898	GO:2000463	1	0.953118863	0	24
17899	GO:2000465	1	0.995985222	0	2
17900	GO:2000466	1	0.994004518	0	3
17901	GO:2000467	1	0.990032816	0	5
17902	GO:2000469	1	0.998013437	0	1
17903	GO:2000473	1	0.998012965	0	1
17904	GO:2000474	1	0.995997577	0	2
17905	GO:2000478	1	0.998011533	0	1
17907	GO:2000480	1	0.982046944	0	9
17908	GO:2000481	1	0.988072481	0	6
17909	GO:2000486	1	0.998000656	0	1
17910	GO:2000487	1	0.997986047	0	1
17911	GO:2000490	1	0.995986309	0	2
17912	GO:2000491	1	0.992033616	0	4

17913	GO:2000494	1	0.997994159	0	1
17914	GO:2000502	1	0.995930455	0	2
17915	GO:2000503	1	0.995946804	0	2
17916	GO:2000504	1	0.995966165	0	2
17917	GO:2000510	1	0.98593357	0	7
17918	GO:2000514	1	0.997966894	0	1
17919	GO:2000515	1	0.995967136	0	2
17920	GO:2000516	1	0.993932396	0	3
17921	GO:2000521	1	0.995955944	0	2
17922	GO:2000522	1	0.997981373	0	1
17923	GO:2000524	1	0.997980063	0	1
17924	GO:2000525	1	0.995993387	0	2
17925	GO:2000526	1	0.997981373	0	1
17926	GO:2000533	1	0.997986735	0	1
17927	GO:2000534	1	0.998011533	0	1
17928	GO:2000535	1	0.990022625	0	5
17929	GO:2000536	1	0.996003237	0	2
17930	GO:2000538	1	0.998010325	0	1
17931	GO:2000541	1	0.997986688	0	1
17932	GO:2000543	1	0.985980696	0	7
17933	GO:2000544	1	0.996022961	0	2
17934	GO:2000545	1	0.997964216	0	1
17935	GO:2000546	1	0.991967673	0	4
17936	GO:2000547	1	0.997981373	0	1
17937	GO:2000549	1	0.99795385	0	1
17938	GO:2000552	1	0.997956978	0	1
17940	GO:2000556	1	0.991971444	0	4
17941	GO:2000558	1	0.997962035	0	1
17942	GO:2000560	1	0.998013437	0	1
17943	GO:2000562	1	0.981900144	0	9
17944	GO:2000563	1	0.991970682	0	4
17945	GO:2000564	1	0.994022653	0	3
17946	GO:2000565	1	0.997990564	0	1
17947	GO:2000566	1	0.993950344	0	3
17948	GO:2000568	1	0.995965344	0	2
17949	GO:2000570	1	0.997998482	0	1
17950	GO:2000572	1	0.998001552	0	1
17951	GO:2000573	1	0.970344753	0	15
17952	GO:2000574	1	0.988052099	0	6
17953	GO:2000575	1	0.998013433	0	1
17954	GO:2000576	1	0.997990564	0	1
17955	GO:2000582	1	0.99390686	0	3
17956	GO:2000583	1	0.993992471	0	3
17957	GO:2000584	1	0.996021367	0	2
17958	GO:2000586	1	0.998006617	0	1

17959	GO:2000587	1	0.993994966	0	3
17960	GO:2000588	1	0.994044287	0	3
17961	GO:2000590	1	0.998011533	0	1
17962	GO:2000591	1	0.9979925	0	1
17963	GO:2000594	1	0.998006243	0	1
17964	GO:2000601	1	0.980212801	0	10
17965	GO:2000607	1	0.997995407	0	1
17966	GO:2000609	1	0.99594935	0	2
17967	GO:2000611	1	0.99399339	0	3
17968	GO:2000612	1	0.998006243	0	1
17969	GO:2000615	1	0.995984016	0	2
17970	GO:2000616	1	0.998013169	0	1
17971	GO:2000617	1	0.984057229	0	8
17972	GO:2000618	1	0.997996776	0	1
17973	GO:2000619	1	0.993981013	0	3
17974	GO:2000620	1	0.996030731	0	2
17975	GO:2000623	1	0.992041063	0	4
17976	GO:2000626	1	0.997990564	0	1
17977	GO:2000627	1	0.99400394	0	3
17978	GO:2000628	1	0.992031078	0	4
17979	GO:2000630	1	0.993971459	0	3
17980	GO:2000632	1	0.99799807	0	1
17981	GO:2000635	1	0.99598062	0	2
17982	GO:2000637	1	0.978222866	0	11
17983	GO:2000638	1	0.995987523	0	2
17984	GO:2000639	1	0.998004752	0	1
17985	GO:2000640	1	0.995997677	0	2
17986	GO:2000641	1	0.984055406	0	8
17987	GO:2000642	1	0.995925353	0	2
17988	GO:2000643	1	0.984147135	0	8
17989	GO:2000645	1	0.99801218	0	1
17990	GO:2000646	1	0.992031963	0	4
17991	GO:2000647	1	0.989996063	0	5
17992	GO:2000648	1	0.980157635	0	10
17993	GO:2000649	1	0.970190028	0	15
17994	GO:2000650	1	0.982194231	0	9
17995	GO:2000651	1	0.986124945	0	7
17996	GO:2000653	1	0.990022213	0	5
17997	GO:2000655	1	0.998005942	0	1
17998	GO:2000657	1	0.99797657	0	1
17999	GO:2000659	1	0.995963682	0	2
18000	GO:2000660	1	0.989942557	0	5
18001	GO:2000661	1	0.998012691	0	1
18002	GO:2000669	1	0.989966007	0	5
18003	GO:2000670	1	0.993993562	0	3

18004	GO:2000671	1	0.998004907	0	1
18005	GO:2000672	1	0.993982566	0	3
18006	GO:2000675	1	0.992035348	0	4
18007	GO:2000676	1	0.991951055	0	4
18008	GO:2000677	1	0.99602814	0	2
18009	GO:2000678	1	0.982032321	0	9
18010	GO:2000679	1	0.982095853	0	9
18011	GO:2000683	1	0.997995407	0	1
18012	GO:2000685	1	0.997991726	0	1
18013	GO:2000691	1	0.998009692	0	1
18014	GO:2000696	1	0.998013437	0	1
18015	GO:2000697	1	0.997984157	0	1
18016	GO:2000703	1	0.997995407	0	1
18017	GO:2000706	1	0.998003787	0	1
18018	GO:2000707	1	0.995996692	0	2
18019	GO:2000719	1	0.997957984	0	1
18020	GO:2000721	1	0.998011604	0	1
18021	GO:2000723	1	0.997989286	0	1
18022	GO:2000724	1	0.997990564	0	1
18023	GO:2000726	1	0.986027904	0	7
18024	GO:2000727	1	0.988057385	0	6
18025	GO:2000729	1	0.995964672	0	2
18026	GO:2000730	1	0.997996776	0	1
18027	GO:2000734	1	0.997995407	0	1
18028	GO:2000736	1	0.989960436	0	5
18029	GO:2000737	1	0.970408997	0	15
18030	GO:2000738	1	0.989962736	0	5
18031	GO:2000739	1	0.998013437	0	1
18032	GO:2000740	1	0.998003787	0	1
18033	GO:2000741	1	0.992065156	0	4
18034	GO:2000744	1	0.995967207	0	2
18035	GO:2000751	1	0.997990564	0	1
18036	GO:2000753	1	0.997991137	0	1
18037	GO:2000755	1	0.997991137	0	1
18038	GO:2000757	1	0.989979319	0	5
18039	GO:2000758	1	0.992058479	0	4
18040	GO:2000761	1	0.99801259	0	1
18041	GO:2000765	1	0.995999769	0	2
18042	GO:2000766	1	0.986122513	0	7
18043	GO:2000767	1	0.986065392	0	7
18044	GO:2000768	1	0.993983377	0	3
18045	GO:2000771	1	0.998013437	0	1
18046	GO:2000772	1	0.974292224	0	13
18047	GO:2000773	1	0.962627867	0	19
18048	GO:2000774	1	0.976014785	0	12

18049	GO:2000775	1	0.997980104	0	1
18050	GO:2000777	1	0.995966238	0	2
18051	GO:2000779	1	0.992035206	0	4
18052	GO:2000780	1	0.990070386	0	5
18053	GO:2000781	1	0.974203495	0	13
18054	GO:2000785	1	0.972261129	0	14
18055	GO:2000786	1	0.978232763	0	11
18056	GO:2000791	1	0.996023267	0	2
18057	GO:2000794	1	0.998006804	0	1
18058	GO:2000795	1	0.998013437	0	1
18059	GO:2000798	1	0.998003787	0	1
18060	GO:2000805	1	0.995985075	0	2
18061	GO:2000806	1	0.998013437	0	1
18062	GO:2000807	1	0.994022798	0	3
18063	GO:2000808	1	0.998013415	0	1
18064	GO:2000809	1	0.994046625	0	3
18065	GO:2000810	1	0.972292427	0	14
18066	GO:2000811	1	0.964586825	0	18
18067	GO:2000812	1	0.996000831	0	2
18068	GO:2000813	1	0.991985904	0	4
18069	GO:2000815	1	0.99600362	0	2
18070	GO:2000816	1	0.995910172	0	2
18071	GO:2000818	1	0.997991532	0	1
18072	GO:2000819	1	0.992009247	0	4
18073	GO:2000820	1	0.993997939	0	3
18074	GO:2000821	1	0.992013439	0	4
18075	GO:2000822	1	0.994002401	0	3
18076	GO:2000825	1	0.993945493	0	3
18077	GO:2000826	1	0.998004776	0	1
18078	GO:2000827	1	0.997986375	0	1
18079	GO:2000836	1	0.99799854	0	1
18080	GO:2000845	1	0.996016494	0	2
18081	GO:2000850	1	0.996011072	0	2
18082	GO:2000852	1	0.997965514	0	1
18083	GO:2000860	1	0.995989881	0	2
18084	GO:2000864	1	0.998013437	0	1
18085	GO:2000866	1	0.995986309	0	2
18086	GO:2000870	1	0.997978885	0	1
18087	GO:2000969	1	0.988065045	0	6
18088	GO:2000971	1	0.997985262	0	1
18089	GO:2000973	1	0.992053453	0	4
18090	GO:2000974	1	0.993960505	0	3
18091	GO:2000975	1	0.998010676	0	1
18092	GO:2000978	1	0.993907262	0	3
18093	GO:2000979	1	0.99799543	0	1

18094	GO:2000981	1	0.995939184	0	2
18095	GO:2000984	1	0.997964583	0	1
18096	GO:2000986	1	0.997962583	0	1
18097	GO:2000987	1	0.989972049	0	5
18098	GO:2001012	1	0.997974922	0	1
18099	GO:2001013	1	0.992055523	0	4
18100	GO:2001014	1	0.986070823	0	7
18101	GO:2001016	1	0.994020665	0	3
18102	GO:2001019	1	0.997959962	0	1
18103	GO:2001020	1	0.978176447	0	11
18104	GO:2001021	1	0.997981405	0	1
18105	GO:2001022	1	0.974224957	0	13
18106	GO:2001023	1	0.995983334	0	2
18107	GO:2001027	1	0.994016394	0	3
18108	GO:2001028	1	0.980086	0	10
18109	GO:2001030	1	0.993965715	0	3
18110	GO:2001031	1	0.99801341	0	1
18111	GO:2001032	1	0.98603855	0	7
18112	GO:2001033	1	0.986009339	0	7
18113	GO:2001034	1	0.968345724	0	16
18114	GO:2001037	1	0.995985805	0	2
18115	GO:2001038	1	0.996009062	0	2
18116	GO:2001044	1	0.989932685	0	5
18117	GO:2001045	1	0.994039718	0	3
18118	GO:2001046	1	0.986007942	0	7
18119	GO:2001051	1	0.997978453	0	1
18120	GO:2001054	1	0.989980389	0	5
18121	GO:2001056	1	0.980057433	0	10
18122	GO:2001065	1	0.997966932	0	1
18123	GO:2001069	1	0.984064177	0	8
18124	GO:2001070	1	0.993998816	0	3
18125	GO:2001076	1	0.997974974	0	1
18126	GO:2001106	1	0.996014729	0	2
18127	GO:2001107	1	0.995985075	0	2
18128	GO:2001108	1	0.998013437	0	1
18129	GO:2001111	1	0.997978203	0	1
18130	GO:2001113	1	0.99801283	0	1
18131	GO:2001125	1	0.997990564	0	1
18132	GO:2001135	1	0.988042383	0	6
18133	GO:2001136	1	0.997999596	0	1
18134	GO:2001137	1	0.990034118	0	5
18135	GO:2001140	1	0.987937318	0	6
18136	GO:2001141	1	0.990074553	0	5
18137	GO:2001142	1	0.997987639	0	1
18138	GO:2001145	1	0.997981583	0	1

18139	GO:2001150	1	0.995965931	0	2
18140	GO:2001153	1	0.997997701	0	1
18141	GO:2001161	1	0.998013437	0	1
18142	GO:2001162	1	0.995978238	0	2
18143	GO:2001165	1	0.997983052	0	1
18144	GO:2001168	1	0.992036492	0	4
18145	GO:2001169	1	0.995988324	0	2
18146	GO:2001170	1	0.993999317	0	3
18147	GO:2001171	1	0.974179301	0	13
18148	GO:2001173	1	0.997980789	0	1
18149	GO:2001178	1	0.997984204	0	1
18150	GO:2001185	1	0.99798545	0	1
18151	GO:2001186	1	0.993952525	0	3
18152	GO:2001187	1	0.995947912	0	2
18153	GO:2001189	1	0.993956155	0	3
18154	GO:2001190	1	0.993922124	0	3
18155	GO:2001193	1	0.997992275	0	1
18156	GO:2001198	1	0.995950993	0	2
18157	GO:2001199	1	0.987932745	0	6
18158	GO:2001200	1	0.991974708	0	4
18159	GO:2001204	1	0.99598735	0	2
18160	GO:2001205	1	0.990033799	0	5
18161	GO:2001206	1	0.991969213	0	4
18162	GO:2001212	1	0.993997674	0	3
18163	GO:2001213	1	0.99600308	0	2
18164	GO:2001214	1	0.978086178	0	11
18165	GO:2001222	1	0.968495588	0	16
18166	GO:2001223	1	0.980178973	0	10
18167	GO:2001224	1	0.970464208	0	15
18168	GO:2001225	1	0.996029947	0	2
18169	GO:2001226	1	0.996000115	0	2
18170	GO:2001229	1	0.998013437	0	1
18171	GO:2001233	1	0.997983076	0	1
18172	GO:2001234	1	0.939497938	0	31
18173	GO:2001235	1	0.94715184	0	27
18174	GO:2001236	1	0.986062708	0	7
18175	GO:2001237	1	0.928361963	0	37
18176	GO:2001238	1	0.946953784	0	27
18177	GO:2001239	1	0.99801343	0	1
18179	GO:2001241	1	0.98201306	0	9
18180	GO:2001242	1	0.987980868	0	6
18181	GO:2001243	1	0.949004348	0	26
18182	GO:2001244	1	0.927896	0	37
18183	GO:2001245	1	0.997962172	0	1
18184	GO:2001246	1	0.9980071	0	1

18185	GO:2001247	1	0.993990219	0	3
18186	GO:2001251	1	0.997981138	0	1
18187	GO:2001252	1	0.996002551	0	2
18188	GO:2001253	1	0.997994512	0	1
18189	GO:2001255	1	0.996007192	0	2
18190	GO:2001256	1	0.974161585	0	13
18191	GO:2001257	1	0.996003884	0	2
18192	GO:2001258	1	0.995986822	0	2
18193	GO:2001259	1	0.990063635	0	5
18194	GO:2001260	1	0.995971199	0	2
18195	GO:2001268	1	0.987916399	0	6
18196	GO:2001269	1	0.985981536	0	7
18197	GO:2001271	1	0.994013167	0	3
18198	GO:2001272	1	0.997957079	0	1
18199	GO:2001280	1	0.996021089	0	2
18200	GO:2001286	1	0.99800808	0	1
18201	GO:2001287	1	0.995980033	0	2
18202	GO:2001288	1	0.994015581	0	3
18203	GO:2001294	1	0.997981761	0	1
18204	GO:2001295	1	0.996030731	0	2
18205	GO:2001301	1	0.989914009	0	5
18206	GO:2001302	1	0.997965198	0	1
18207	GO:2001303	1	0.993974509	0	3
18208	GO:2001306	1	0.99798452	0	1
18209	GO:2001311	1	0.995964405	0	2
15414	GO:0106310	1	0.492342588	0	351
15415	GO:0106311	1	0.492342588	0	351
128	GO:0000287	1	0.655903966	0	209
299	GO:0000922	1	0.757319875	0	138
3083	GO:0006897	1	0.631820309	0	228
5101	GO:0016477	1	0.605508315	0	249
254	GO:0000781	1	0.731634388	0	155
4882	GO:0015918	1	0.962608511	0	19
4970	GO:0016175	1	0.980074901	0	10

ne bar chart (bold). The bars
ts the gene count hits as a
s.

term	ontology
**immune system ¦**	BP
**response to virus**	BP
**negative regulati**	BP
**innate immune r**	BP
**defense response**	BP
**type I interferon**	BP
**interferon-gamm**	BP
double-stranded	MF
interleukin-27-me	BP
antiviral innate in	BP
positive regulatio	BP
2'-5'-oligoadenyla	MF
**regulation of ribo**	BP
RNA helicase acti	MF
**negative regulati**	BP
**response to bact**	BP
**defense response**	BP
positive regulatio	BP
helicase activity	MF
**positive regulatio**	BP
**viral process**	BP
RNA binding	MF
positive regulatio	BP
MDA-5 signaling ¦	BP
CXCR3 chemokine	MF
postsynaptic endo	CC
**regulation of defe**	BP
**ISG15-protein co**	BP
synaptic vesicle b	BP
RIG-I signaling pa	BP
single-stranded R	MF
dynamin family p	BP
postsynaptic neu	BP
response to type	BP
**T cell chemotaxis**	BP
regulation of syna	BP
response to inter	BP
**immune respons**	BP
dendritic spine he	CC
**cellular response**	BP

CXCR chemokine	MF
mitochondrial me	CC
nucleotide bindin	MF
regulation of lipic	BP
nucleotidyltransfe	MF
mitochondrial fiss	BP
cellular response	BP
positive regulatio	BP
negative regulatic	BP
**positive regulatic**	BP
defense response	BP
positive regulatio	BP
blood circulation	BP
sterol biosyntheti	BP
positive regulatio	BP
chemokine activit	MF
negative regulatic	BP
palmitoyl-CoA 9-c	MF
negative regulatic	BP
**regulation of lact**	BP
regulation of vira	BP
recombination hc	MF
sterol delta7 redu	MF
brassinosteroid b	BP
7-dehydrocholest	MF
cholesterol transp	BP
**regulation of Myl**	BP
**regulation of T ce**	BP
modulation by hc	BP
response to cold	BP
endoplasmic retic	CC
perinuclear regio	CC
cellular response	BP
cholesterol biosy	BP
**cellular response**	BP
receptor internali	BP
membrane fusior	BP
**chemokine-medi**	BP
regulation of type	BP
cytoplasmic patte	BP
alveolar lamellar	CC
negative regulatic	BP
negative regulatic	BP
monounsaturatec	BP
UMP kinase activ	MF

CMP kinase activi	MF
dCMP kinase acti	MF
nucleoside mono	MF
renal tubule deve	BP
**regulation of mo**	BP
**regulation of My**	BP
protein localizatio	BP
thymidylate kinas	MF
**dUDP biosynthet**	BP
**dTDP biosyntheti**	BP
establishment of	BP
cellular response	BP
GMP reductase a	MF
GMP reductase c	CC
negative regulatio	BP
intracellular trans	BP
**antimicrobial hu**	BP
ATPase activity	MF
regulation of cell	BP
nucleobase-conta	BP
GTP binding	MF
positive regulatio	BP
**neutrophil chem**	BP
oxidoreductase a	MF
stearoyl-CoA 9-de	MF
metanephric mes	BP
metanephric mes	BP
negative regulatio	BP
negative regulatio	BP
negative regulatio	BP
positive regulatio	BP
positive regulatio	BP
uridylate kinase a	MF
C-4 methylsterol	MF
CD4-positive, alp	BP
activation of imm	BP
steroid biosynthe	BP
cytoplasm	CC
ATP binding	MF
positive regulatio	BP
negative regulatio	BP
calcium-induced	MF
positive regulatio	BP
ryanodine-sensiti	MF
CD4-positive, alp	BP

regulation of end	BP
cytidylate kinase	MF
negative regulatio	BP
cholesterol biosyı	BP
cholesterol biosyı	BP
negative regulatio	BP
purine nucleobas	BP
ISG15 transferase	MF
dTTP biosynthetic	BP
**negative regulati**	BP
apoptotic signalir	BP
macrophage deriv	BP
positive regulatio	BP
negative regulatio	BP
regulation of inte	BP
positive regulatio	BP
purine nucleotide	BP
cellular response	BP
intracellular mem	CC
negative regulatio	BP
oxidation-reducti	BP
positive regulatio	BP
CCR5 chemokine	MF
oxidoreductase a	MF
detection of virus	BP
regulation of nucl	BP
establishment of	BP
positive regulatio	BP
negative regulatio	BP
cholesterol impoı	BP
adenylate cyclase	BP
cytosol	CC
cellular response	BP
interleukin-21-me	BP
positive regulatio	BP
regulation of prot	BP
cAMP-dependent	MF
positive regulatio	BP
endoplasmic retic	CC
cytoplasmic vesic	CC
cholesterol metal	BP
calcium-release c	MF
interleukin-9-me	BP
negative regulatio	BP
histone H3-K36 m	BP

endothelial cell a	BP
pyrimidine nucle	BP
regulation of cAN	BP
nucleoside tripho	BP
modification-dep	BP
GTPase activity	MF
cellular response	BP
histone methyltr	MF
regulation of epic	BP
regulation of ada	BP
regulation of calc	BP
response to inter	BP
negative regulati	BP
regulation of chol	BP
interleukin-35-me	BP
nucleoside mono	BP
positive regulatio	BP
steroid metabolic	BP
nucleic acid bindi	MF
intracellular chol	BP
fatty-acyl-CoA bic	BP
negative regulati	BP
integral compone	CC
regulation of inte	BP
positive regulatio	BP
immunoglobulin	BP
cytolysis	BP
protein tag	MF
positive regulatio	BP
positive regulatio	BP
regulation of type	BP
unsaturated fatty	BP
positive regulatio	BP
purine-containing	BP
chemotaxis	BP
iron ion binding	MF
oxidoreductase a	MF
positive regulatio	BP
double-stranded	MF
negative regulati	BP
histone methyltr	MF
response to audit	BP
positive regulatio	BP
molecular_functi	MF
heparin binding	MF

histone H3-K4 tri	BP
histone methyltra	MF
negative regulatio	BP
response to vitam	BP
interleukin-6-mec	BP
lamin binding	MF
negative regulatio	BP
cellular response	BP
apoptotic mitoch	BP
negative regulatio	BP
positive regulatio	BP
response to exog	BP
RNA polymerase	MF
cholesterol trans	MF
presynapse	CC
nucleoside dipho	MF
nucleoside dipho	BP
cytokine activity	MF
apoptotic process	BP
regulation of prot	BP
negative regulatio	BP
cytoplasmic vesic	CC
axon	CC
cellular response	BP
positive regulatio	BP
histone methylati	BP
negative regulatio	BP
lipid biosynthetic	BP
regulation of inna	BP
response to fatty	BP
ribonuclease activ	MF
release of cytoch	BP
cell-cell signaling	BP
calcium channel c	CC
negative regulatio	BP
ubiquitin-like pro	MF
RNA catabolic pro	BP
positive regulatio	BP
nuclear hormone	MF
histone-lysine N-r	MF
smooth endoplas	CC
histone acetyltra	MF
nuclear outer me	CC
response to gamr	BP
microtubule cyto	CC

fatty acid metabo	BP
spectrin binding	MF
mitochondrial ou	CC
tumor necrosis fa	MF
chemoattractant	MF
protein phosphat	MF
positive regulatio	BP
receptor signaling	BP
oxidoreductase a	MF
negative regulatio	BP
thyroid hormone	MF
TRIF-dependent t	BP
nucleotide metab	BP
release of seques	BP
intrinsic apoptoti	BP
RNA phosphodies	BP
sarcoplasmic retic	CC
vesicle membrane	CC
negative regulatio	BP
positive regulatio	BP
positive regulatio	BP
reactive oxygen s	BP
protein binding	MF
translesion synth	BP
NADP binding	MF
positive chemota	BP
pattern specificat	BP
repressing transc	MF
response to cAM	BP
cytokine binding	MF
regulation of apo	BP
identical protein	MF
RNA polymerase	MF
extrinsic apoptoti	BP
Hsp90 protein bin	MF
cholesterol bindin	MF
intermembrane li	BP
negative regulatio	BP
cellular response	BP
4 iron, 4 sulfur cl	MF
protein deubiquit	BP
cytosolic small rib	CC
hydrolase activity	MF
regulation of carc	BP
actin cytoskeleton	CC

microtubule bind	MF
cytokine-mediate	BP
response to pepti	BP
protein homotetr	BP
lipid metabolic pr	BP
intermediate filar	CC
postsynaptic den:	CC
nuclear inner mei	CC
protein self-assoc	MF
positive regulatio	BP
negative regulatic	BP
promoter-specific	MF
cellular response	BP
response to mech	BP
regulation of gen	BP
host cell	CC
response to cytok	BP
response to hydr	BP
nuclear receptor	MF
positive regulatio	BP
regulation of cycl	BP
sarcoplasmic retic	CC
protein K48-linke	BP
ubiquitin protein	MF
cis-regulatory reg	MF
fatty acid biosynt	BP
cellular response	BP
iron-sulfur cluster	MF
cellular response	BP
negative regulatic	BP
GDP binding	MF
regulation of auto	BP
cellular_compone	CC
inflammatory res	BP
response to nutri	BP
calcium channel a	MF
cellular response	BP
serine-type endo	MF
positive regulatio	BP
nuclear pore	CC
intracellular recep	BP
positive regulatio	BP
cellular calcium ic	BP
regulation of cell	BP
muscle organ dev	BP

lipid droplet	CC
negative regulatio	BP
peptidase inhibito	MF
cellular response	BP
integrin-mediated	BP
thiol-dependent u	MF
ruffle membrane	CC
enzyme binding	MF
mitochondrion	CC
positive regulatio	BP
sarcolemma	CC
mRNA transport	BP
regulation of imm	BP
catalytic activity	MF
tumor necrosis fa	BP
DNA binding	MF
cellular response	BP
calcium ion transı	BP
positive regulatio	BP
Z disc	CC
bicellular tight jur	CC
calcium ion trans	BP
negative regulatio	BP
negative regulatio	BP
response to orgaı	BP
nuclear-transcrib	BP
positive regulatio	BP
extracellular spac	CC
mitochondrial inr	CC
late endosome m	CC
lipid transport	BP
nucleolus	CC
integrin binding	MF
membrane	CC
negative regulatio	BP
fibrillar center	CC
ion channel activi	MF
regulation of cell	BP
histone binding	MF
ribonucleoprotei	CC
response to lipop	BP
lyase activity	MF
transferase activi	MF
vesicle	CC
extracellular regio	CC

adaptive immune	BP
plasma membran	CC
DNA-binding tran	MF
calmodulin bindir	MF
transmembrane t	MF
methyltransferas	MF
proteasome-med	BP
nucleoplasm	CC
methylation	BP
transmembrane t	BP
nuclear envelope	CC
ion transport	BP
protein phosphor	BP
cell surface recep	BP
signal transductic	BP
transcription regu	MF
protein homodim	MF
positive regulatio	BP
G protein-couple	BP
nuclear membrar	CC
positive regulatio	BP
external side of p	CC
ubiquitin-protein	MF
metal ion binding	MF
ion transmembra	BP
protein polyubiqu	BP
early endosome	CC
ubiquitin protein	MF
response to drug	BP
endosome memb	CC
ubiquitin-depend	BP
regulation of tran	BP
zinc ion binding	MF
lipid binding	MF
chromatin	CC
cadherin binding	MF
signaling recepto	MF
lysosomal memb	CC
chromatin organi	BP
positive regulatio	BP
actin binding	MF
negative regulatic	BP
sequence-specific	MF
lysosome	CC
dendrite	CC

chromatin bindin	MF
sequence-specific	MF
RNA polymerase	MF
DNA-binding tran	MF
cellular response	BP
chromosome	CC
protein ubiquitin	BP
nucleus	CC
Golgi membrane	CC
integral compone	CC
calcium ion bindi	MF
Golgi apparatus	CC
protein transport	BP
kinase activity	MF
phosphorylation	BP
NA	NA
protein-containin	CC
cell junction	CC
cell projection	CC
cytoskeleton	CC
regulation of tran	BP
positive regulatio	BP
potassium ion bir	MF
positive regulatio	BP
motor activity	MF
cell cycle	BP
synapse organiza	BP
nuclear speck	CC
mRNA processing	BP
GTPase activator	MF
cell surface	CC
integral compone	CC
regulation of can	BP
mitochondrial ge	BP
reproduction	BP
alpha-1,6-manno	MF
trans-hexaprenyl	MF
single strand bre	BP
single-stranded D	MF
phosphopyruvate	CC
alpha-glucoside t	BP
regulation of DN	BP
regulation of mit	BP
mitotic spindle el	BP
maltose metaboli	BP

alpha-1,2-manno:	MF
ribosomal large s	BP
ribosomal small s	BP
mannosyltransfer	MF
cell wall mannopr	BP
alpha-1,3-manno:	MF
acyl binding	MF
acyl carrier activit	MF
very long-chain fa	BP
autophagosome a	BP
peptidyltransfera	MF
tRNA binding	MF
urea cycle	BP
citrulline metabo	BP
argininosuccinate	BP
ribosomal subuni	BP
ribosomal large s	BP
ribosomal small s	BP
fatty-acyl-CoA bir	MF
L-ornithine transr	MF
mitotic sister chr	BP
DNA replication c	BP
DNA damage che	BP
G1/S transition of	BP
regulation of tran	BP
G2/M transition c	BP
S-adenosyl-L-met	MF
sulfur amino acid	BP
sulfur amino acid	BP
sulfate assimilatic	BP
succinate dehydr	MF
histidine biosynth	BP
nucleotide-excisic	CC
nucleotide-excisic	CC
nucleotide-excisic	CC
nucleotide-excisic	CC
regulation of tran	BP
histone deacetyla	CC
RNA polymerase	CC
glycerol-1-phospl	MF
histone acetyltra	CC
SAGA complex	CC
PCAF complex	CC
transcription fact	CC
transcription fact	CC

establishment of	BP
Golgi cis cisterna	CC
Golgi trans cistern	CC
exocyst	CC
microfilament mc	MF
SNARE binding	MF
recombinase acti	MF
ubiquitin ligase cc	CC
nuclear ubiquitin	CC
cytoplasmic ubiqu	CC
rRNA modificatio	BP
protein phosphat	CC
phosphorelay sigi	BP
protein phosphat	CC
MAPK cascade	BP
sphingosine hydr	MF
ribonuclease MRI	MF
ribonuclease MRI	CC
3'-5'-exoribonucle	MF
nuclear exosome	CC
cytoplasmic exos	CC
exosome (RNase	CC
rRNA (adenine-N6	MF
rDNA binding	MF
rDNA heterochro	BP
activation of MAI	BP
activation of MAI	BP
activation of MAI	BP
inactivation of M.	BP
NAD+ diphosphat	MF
meiotic spindle o	BP
tRNA-intron endc	MF
tRNA-intron endc	CC
tRNA 2'-phospho	MF
DNA secondary st	MF
vacuolar proton-t	CC
vacuolar proton-t	CC
peptide-N4-(N-ac	MF
N-acetylglucosam	MF
microtubule cyto	BP
nuclear chromosc	CC
astral microtubul	CC
pericentriolar ma	CC
commitment com	CC
spliceosomal tri-s	BP

spliceosomal com	BP
delta24(24-1) ste	MF
C-8 sterol isomer	MF
C-5 sterol desatu	MF
lanosterol syntha	MF
C-3 sterol dehydr	MF
3-keto sterol redu	MF
peroxisome targe	MF
polysaccharide bi	BP
polysaccharide ca	BP
mitochondrial pro	CC
mitochondrial pro	CC
mitochondrial pro	CC
mitotic cell cycle	BP
mitotic cytokines	BP
1-phosphatidylin	MF
nuclear-transcrib	BP
nuclear-transcrib	BP
deadenylation-de	BP
nuclear-transcrib	BP
ferric-chelate red	MF
nuclear-transcrib	BP
adenine nucleotid	MF
spermine transpo	BP
endopolyphospha	MF
retrograde transp	BP
response to react	BP
response to supe	BP
response to singl	BP
response to oxyg	BP
extrinsic compon	CC
cyclin-dependent	CC
cytoplasmic cyclin	CC
nicotinamide-nuc	MF
re-entry into mito	BP
storage vacuole	CC
lytic vacuole	CC
telomerase catal	CC
3-hydroxyanthran	MF
protein deneddyl	BP
RNA cap binding	MF
RNA 7-methylgua	MF
transcription exp	CC
THO complex	CC
generation of cat	BP

generation of cat	BP
cis assembly of pı	BP
RNA splicing, via t	BP
tRNA-type intron	BP
alternative mRNA	BP
regulation of alte	BP
second spliceosoı	MF
spliceosomal snR	BP
spliceosome conf	BP
mRNA 3'-splice si	BP
spliceosomal com	BP
mRNA 5'-splice si	BP
mRNA splicing, vi	BP
four-way junction	MF
Y-form DNA bindi	MF
heteroduplex DN	MF
bubble DNA bindi	MF
double-strand/sir	MF
phagophore asseı	CC
EKC/KEOPS comp	CC
protein peptidyl-ı	BP
regulation of hist	BP
negative regulati	BP
positive regulatio	BP
HIR complex	CC
autophagosome ı	CC
autophagy of mit	BP
mitophagy	BP
pexophagy	BP
regulation of tran	BP
positive regulatio	BP
positive regulatio	BP
core TFIIH comple	CC
transcription fact	CC
MIS12/MIND typ	CC
THO complex par	CC
endonucleolytic c	BP
cleavage in ITS2 b	BP
rRNA 2'-O-methyl	BP
enzyme-directed	BP
snoRNA guided rI	BP
enzyme-directed	BP
maturation of 5.8	BP
endonucleolytic c	BP
maturation of SSU	BP

maturation of LSU	BP
maturation of 5.8	BP
exonucleolytic tri	BP
cleavage involvec	BP
maturation of LSU	BP
endonucleolytic c	BP
endonucleolytic c	BP
endonucleolytic c	BP
maturation of 5S	BP
box C/D snoRNP a	BP
box H/ACA snoRN	BP
box C/D snoRNA :	BP
box H/ACA snoRN	BP
proteasome comp	CC
glycosylphosphat	CC
embryonic axis sp	BP
mismatch base pa	MF
purine-specific m	MF
oxidized pyrimidi	MF
meiotic DNA doul	BP
meiotic DNA reco	BP
meiotic mismatch	BP
meiotic DNA repa	BP
resolution of mei	BP
nucleotide-excisio	BP
nucleotide-excisio	BP
nucleotide-excisio	BP
pyrimidine dimer	BP
(R,R)-butanediol c	MF
telomere mainter	BP
telomere mainter	BP
double-strand bre	BP
recombinational r	BP
non-recombinatio	BP
double-strand bre	BP
DNA double-strar	BP
DNA recombinase	BP
DNA synthesis inv	BP
strand displacem	BP
DNA strand renat	BP
removal of nonho	BP
DNA catabolic pro	BP
DNA catabolic pro	BP
syncytium format	BP
phosphatidyl-N-m	MF

adenyl-nucleotide	MF
chromosome, cer	CC
kinetochore	CC
condensed chrom	CC
condensed nucle	CC
condensed chrom	CC
nuclear telomere	CC
nucleosome	CC
euchromatin	CC
heterochromatin	CC
condensed chrom	CC
condensed nucle	CC
synaptonemal co	CC
condensin compl	CC
lateral element	CC
central element	CC
transverse filame	CC
sex chromosome	CC
X chromosome	CC
Y chromosome	CC
origin recognition	CC
diacylglycerol dip	MF
GINS complex	CC
Swr1 complex	CC
ESCRT I complex	CC
ESCRT II complex	CC
ESCRT III complex	CC
nuclear MIS12/M	CC
sister chromatid s	BP
regulation of glut	BP
regulation of argi	BP
inositol hexakisph	MF
inositol-1,4,5-tris	MF
inositol tetrakisph	MF
inositol-1,3,4,5,6-	MF
inositol hexakisph	MF
inositol heptakisp	MF
inositol hexakisph	MF
inositol hexakisph	MF
ER ubiquitin ligase	CC
Hrd1p ubiquitin li	CC
Hrd1p ubiquitin li	CC
translation repres	MF
cell morphogenes	BP
cell morphogenes	BP

actomyosin contr	BP
actomyosin contr	BP
division septum a	BP
septin ring assem	BP
equatorial microt	CC
gamma-tubulin co	CC
P-body	CC
GARP complex	CC
condensed chrom	CC
condensed chrom	CC
condensed nuclea	CC
condensed nuclea	CC
nuclear-transcrib	BP
mitochondrial RN	BP
mitochondrial mF	BP
mitochondrial RN	BP
negative regulatio	BP
positive regulatio	BP
mitochondrial RN	BP
mitochondrial RN	BP
mitochondrial RN	BP
RNA 5'-end proce	BP
tRNA exon ligatio	BP
transcription-dep	BP
posttranscription	BP
Prp19 complex	CC
RNA polymerase	MF
RNA polymerase	MF
RNA polymerase	MF
RNA polymerase	MF
RNA polymerase	MF
RNA polymerase	MF
RNA polymerase	MF
RNA polymerase	MF
RNA polymerase	MF
mitochondrial pro	MF
RNA polymerase	MF
core promoter se	MF
RNA polymerase	MF
RNA polymerase	MF
RNA polymerase	MF
regulatory region	MF
regulatory region	MF
RNA polymerase	MF
RNA polymerase	MF

TFIIB-class transc	MF
TFIID-class transc	MF
TFIIF-class transc	MF
TFIIH-class transc	MF
basal RNA polyme	MF
negative regulatio	BP
RNA polymerase	MF
DNA-templated t	BP
transcription ope	BP
transcription tern	MF
TFIIIC-class transc	MF
intronic transcrip	MF
RNA polymerase	MF
RNA polymerase	MF
RNA polymerase	MF
transcription, RN	BP
transcriptional st	BP
RNA polymerase	MF
RNA polymerase	MF
RNA polymerase	BP
maintenance of t	BP
histone displacem	BP
DNA-binding tran	MF
DNA-binding tran	MF
transcription cofa	MF
transcription core	MF
transcription coa	MF
RNA polymerase	MF
RNA polymerase	MF
DNA-binding tran	MF
DNA-binding tran	MF
age-dependent re	BP
age-dependent re	BP
SAM complex	CC
PAM complex, Tir	CC
guanine nucleotid	MF
skeletal system d	BP
cartilage condens	BP
ossification	BP
neurotransmitter	BP
regulation of neu	BP
acetylcholine cat	BP
action potential	BP
RNA methylation	BP
dihydronicotinam	MF

selenocysteine in	BP
opioid peptide ac	MF
prostaglandin bio	BP
N-acetylglucosam	MF
voltage-gated soc	CC
peptide amidatio	BP
outer dense fiber	CC
pseudouridine sy	BP
retinoid metaboli	BP
angiogenesis	BP
microfibril	CC
lipopolysaccharid	MF
interleukin-21 rec	MF
cornified envelop	CC
radial spoke	CC
radial spoke stalk	CC
N-acetylgalactosa	MF
amyloid-beta bin	MF
ovarian follicle de	BP
ovulation from ov	BP
ovarian follicle ru	BP
initiation of prim	BP
primary ovarian f	BP
preantral ovarian	BP
antral ovarian fol	BP
ovarian cumulus	BP
ovarian follicle at	BP
luteinization	BP
luteolysis	BP
oocyte growth	BP
oocyte maturatio	BP
regulation of cell	BP
regulation of cell	BP
fatty acid alpha-o	BP
response to prot	BP
cholesterol 25-hy	MF
blood vessel deve	BP
branching involve	BP
vasculogenesis	BP
lactosylceramide	BP
ganglioside metal	BP
ganglioside biosy	BP
globoside biosynt	BP
microtubule bunc	BP
medium-chain fat	BP

detection of chen	BP
detection of chen	BP
detection of chen	BP
Gq/11-coupled se	MF
dopamine neurot	MF
dopamine neurot	MF
angiotensin recep	MF
pancreatic polype	MF
adrenomedullin r	MF
neuromedin U re	MF
G protein-couple	MF
purinergic nucleo	MF
virus receptor act	MF
G protein-couple	MF
nociceptin recept	MF
cysteinyl leukotri	MF
leukotriene B4 re	MF
calcitonin gene-re	MF
adenylate cyclase	MF
group II metabot	MF
group III metabot	MF
osteoblast differe	BP
dense fibrillar cor	CC
granular compon	CC
peptide receptor	MF
eye development	BP
urogenital system	BP
metanephros dev	BP
ureteric bud deve	BP
branching involve	BP
temperature hom	BP
fever generation	BP
conditioned taste	BP
behavioral fear re	BP
G protein-couple	MF
alpha-N-acetylgal	MF
response to hypo	BP
ameboidal-type c	BP
acrosomal vesicle	CC
ATPase activator	MF
regulation of chro	BP
male germ cell nu	CC
female germ cell	CC
acrosome assemb	BP
long-chain fatty a	BP

cellular glucose h	BP
tRNA 3'-terminal	BP
sialate O-acetyles	MF
tRNA 5'-leader re	BP
pseudophosphata	MF
histamine metab	BP
histamine biosyn	BP
histamine catabo	BP
gastric acid secre	BP
in utero embryon	BP
gastrulation with	BP
formation of prim	BP
ectoderm format	BP
endoderm forma	BP
mesoderm forma	BP
cell fate specifica	BP
cell fate determir	BP
mesodermal cell	BP
endodermal cell f	BP
ectodermal cell fa	BP
endodermal cell f	BP
L-amino-acid oxic	MF
stress fiber	CC
ruffle	CC
lipid kinase activi	MF
ceramide kinase a	MF
formation of tran	BP
formation of cyto	BP
galactosylceramic	MF
mRNA (N6-adeno	MF
prenylcysteine ox	MF
establishment of	BP
morphogenesis o	BP
sex chromatin	CC
Barr body	CC
XY body	CC
photoreceptor ou	CC
eye photorecepto	BP
neural crest cell n	BP
somitogenesis	BP
somite specificati	BP
retinal dehydroge	MF
organ induction	BP
morphogenesis o	BP
neuron migration	BP

membrane raft as	BP
membrane raft p	BP
establishment of	BP
establishment of	BP
immunological sy	BP
immunological sy	CC
myeloid dendritic	BP
microglial cell act	BP
cell activation	BP
leukocyte homeo	BP
T cell homeostati	BP
plasma membran	BP
natural killer cell	BP
neutrophil home	BP
neutrophil apopt	BP
B cell homeostasi	BP
B cell apoptotic p	BP
phosphotyrosine	MF
phosphatidylserir	MF
natural killer cell	BP
IgM binding	MF
polymeric immun	MF
positive regulatio	BP
positive regulatio	BP
type IV hypersens	BP
negative regulatio	BP
positive regulatio	BP
negative regulatio	BP
positive regulatio	BP
negative regulatio	BP
positive regulatio	BP
regulation of cyto	BP
negative regulatio	BP
positive regulatio	BP
serotonin secretio	BP
histamine secreti	BP
kidney developm	BP
mesonephros dev	BP
blastocyst develo	BP
blastocyst format	BP
inner cell mass ce	BP
inner cell mass ce	BP
inner cell mass ce	BP
trophectodermal	BP
trophectodermal	BP

trophectodermal	BP
blastocyst growth	BP
inner cell mass ce	BP
trophectodermal	BP
blastocyst hatchi	BP
epithelial to mese	BP
embryonic epithe	BP
neural plate mor	BP
neural plate deve	BP
neural tube form	BP
neural fold forma	BP
neural tube closu	BP
protein insertion	BP
phagolysosome a	BP
opsonin binding	MF
opsonin receptor	MF
complement binc	MF
complement com	MF
complement com	MF
complement com	MF
complement com	MF
complement com	MF
complement com	MF
NK T cell differen	BP
NK T cell prolifera	BP
complement acti	BP
negative regulati	BP
(1->3)-beta-D-glu	MF
lipopolysaccharid	MF
response to yeas	BP
Mullerian duct re	BP
receptor recyclin	BP
nucleoside bindin	MF
purine nucleoside	MF
endothelial cell d	BP
endothelial cell m	BP
selenium compou	BP
glucuronyl-galact	MF
liver developmen	BP
placenta develop	BP
phagocytic cup	CC
embryonic placer	BP
maternal placent	BP
tissue homeostas	BP
retina homeostas	BP

negative regulati	BP
cell killing	BP
leukocyte mediat	BP
regulation of leuk	BP
positive regulatio	BP
T cell mediated cy	BP
regulation of T ce	BP
negative regulati	BP
positive regulatio	BP
photoreceptor in	CC
farnesylated prot	MF
regulation of rece	BP
negative regulati	BP
positive regulatio	BP
B-1 B cell homeos	BP
B-1 B cell differer	BP
regulation of exo	BP
uropod	CC
regulation of prot	BP
negative regulati	BP
positive regulatio	BP
endothelial cell p	BP
regulation of end	BP
positive regulatio	BP
female pronucleu	CC
male pronucleus	CC
postsynaptic mer	BP
hair follicle devel	BP
vasculature devel	BP
lymph vessel dev	BP
lymphangiogenes	BP
heart looping	BP
sebaceous gland	BP
intestinal D-gluco	BP
regulation of cell-	BP
negative regulati	BP
positive regulatio	BP
blood vessel matu	BP
positive regulatio	BP
intramembranous	BP
endochondral oss	BP
regulation of cyto	BP
negative regulati	BP
positive regulatio	BP
synaptic transmis	BP

startle response	BP
G-protein alpha-s	MF
thigmotaxis	BP
suckling behavior	BP
fibronectin bindir	MF
positive regulatio	BP
negative regulatio	BP
retinoic acid bind	MF
G protein-couplec	BP
blood vessel remo	BP
response to ampl	BP
nervous system p	BP
regulation of syst	BP
negative regulatio	BP
regulation of syst	BP
regulation of syst	BP
norepinephrine-e	BP
positive regulatio	BP
positive regulatio	BP
angiotensin-medi	BP
renin secretion in	BP
angiotensin matu	BP
morphogenesis o	BP
morphogenesis o	BP
regulation of bloc	BP
regulation of bloc	BP
renin-angiotensin	BP
regulation of rena	BP
protease binding	MF
response to dieta	BP
reduction of food	BP
diet induced ther	BP
norepinephrine-e	BP
regulation of the	BP
regulation of hea	BP
regulation of sodi	BP
desensitization of	BP
G protein-couplec	BP
desensitization of	BP
regulation of bloc	BP
brain renin-angio	BP
negative regulatio	BP
positive regulatio	BP
p53 binding	MF
sprouting angioge	BP

intussusceptive a	BP
cell migration inv	BP
blood vessel endo	BP
opsin binding	MF
osteoblast fate co	BP
positive regulatio	BP
adenine binding	MF
uracil binding	MF
thymine binding	MF
purine nucleobas	MF
chondrocyte diffe	BP
chondrocyte deve	BP
epithelial cell dev	BP
columnar/cuboid	BP
glandular epitheli	BP
glandular epitheli	BP
epithelial cell mat	BP
glandular epitheli	BP
optic cup morpho	BP
extraocular skelet	BP
osteoblast develo	BP
acrosome matrix	BP
inner acrosomal r	CC
acrosomal memb	CC
outer acrosomal r	CC
regulation of oxid	BP
4-hydroxybenzoa	MF
protein depalmito	BP
diaphragm contra	BP
regulation of resp	BP
lens developmen	BP
lens morphogene	BP
regulation of rece	BP
negative regulatio	BP
positive regulatio	BP
auditory receptor	BP
polyprenyltransfe	MF
caveolar macrom	CC
polkadots	CC
tRNA wobble uric	BP
tRNA wobble ade	BP
tRNA wobble cyto	BP
podosome	CC
interleukin-33 rec	MF
interleukin-33 bir	MF

interleukin-33 rec	MF
store-operated ca	BP
semaphorin recep	CC
aggressive behavi	BP
inter-male aggres	BP
maternal aggress	BP
tRNA wobble bas	BP
tRNA nucleoside	BP
polycystin comple	CC
UTP binding	MF
CTP binding	MF
retinoic acid bios	BP
stereocilia ankle l	CC
stereocilia ankle l	CC
tRNA wobble pos	BP
cytosolic tRNA wo	CC
G-quadruplex RN	MF
bile acid conjugat	BP
steroid receptor	MF
thyroid hormone	BP
regulation of thyr	BP
osteoclast prolife	BP
desmosome asse	BP
aminoacyl-tRNA e	MF
dystroglycan binc	MF
3-methylcrotonyl	CC
protein localizatic	BP
male germ cell pr	BP
manchette	CC
palmitoyltransfer	CC
cytoplasmic trans	BP
cytoplasmic trans	BP
cytoplasmic trans	BP
cytoplasmic trans	BP
translation reiniti	BP
ribose phosphate	CC
cap-independent	BP
cap-dependent tr	BP
IRES-dependent t	BP
MAML1-RBP-Jkap	CC
hepatocyte cell m	BP
Ser-tRNA(Ala) hyc	MF
zona pellucida rec	CC
somatic recombir	BP
behavioral defens	BP

defense response	BP
activation of inna	BP
innate immune re	BP
pattern recogniti	BP
stimulatory C-typ	BP
toll-like receptor	BP
innate immune re	BP
natural killer cell	BP
leukocyte chemo	BP
response to mole	BP
response to mole	BP
hematopoietic pr	BP
wound healing in	BP
connective tissue	BP
organ or tissue sp	BP
lymphocyte home	BP
myeloid cell hom	BP
cell activation inv	BP
endothelial cell a	BP
astrocyte activati	BP
follicular dendriti	BP
follicular dendriti	BP
leukocyte activati	BP
plasmacytoid den	BP
basophil activatio	BP
myeloid dendritic	BP
monocyte activat	BP
macrophage activ	BP
microglial cell act	BP
neutrophil activat	BP
T cell activation ir	BP
T cell activation v	BP
T cell differentiati	BP
T-helper cell linea	BP
T-helper 1 cell lin	BP
CD8-positive, gan	BP
T cell proliferatio	BP
B cell activation ir	BP
mature B cell diff	BP
germinal center B	BP
marginal zone B c	BP
follicular B cell di	BP
plasma cell differ	BP
myeloid progenit	BP
memory B cell dif	BP

lymphoid progeni	BP
B cell proliferatio	BP
natural killer cell .	BP
B cell lineage com	BP
immature B cell d	BP
pro-B cell differer	BP
pre-B cell differer	BP
pre-B cell allelic e	BP
transitional one s	BP
transitional two s	BP
mature B cell diff	BP
B-1a B cell differe	BP
B cell affinity mat	BP
response to tumc	BP
B cell negative se	BP
plasma kallikrein-	BP
detection of tum	BP
defense response	BP
B cell homeostati	BP
T cell lineage com	BP
CD4-positive, CD2	BP
CD4-positive, CD2	BP
alpha-beta T cell l	BP
NK T cell lineage (	BP
leukocyte activati	BP
immune response	BP
hepatic immune r	BP
mucosal immune	BP
immune response	BP
MHC class I prote	BP
MHC class Ib prot	BP
MHC class II prote	BP
dendritic cell che	BP
myeloid dendritic	BP
antigen transcyto	BP
immunoglobulin t	BP
IgG immunoglobu	BP
immune response	BP
T cell mediated cy	BP
immune response	BP
complement rece	BP
Fc receptor medi	BP
granuloma forma	BP
immune complex	BP
immune complex	BP

immune complex	BP
inflammatory res	BP
acute inflammatc	BP
chronic inflamma	BP
neutrophil media	BP
eosinophil media	BP
mast cell mediate	BP
peripheral B cell t	BP
humoral immune	BP
T cell mediated ir	BP
T cell antigen pro	BP
peripheral T cell t	BP
adaptive immune	BP
germinal center f	BP
myeloid dendritic	BP
plasmacytoid den	BP
antigen processin	BP
antigen processin	BP
antigen processin	BP
antigen processin	BP
antigen processin	BP
antigen processin	BP
antigen processin	BP
antigen processin	BP
antigen processin	BP
antigen processin	BP
antigen processin	BP
peptide antigen a	BP
peptide antigen a	BP
antigen processin	BP
antigen processin	BP
tolerance inducti	BP
central tolerance	BP
central tolerance	BP
tolerance inducti	BP
T cell tolerance ir	BP
lymphocyte chem	BP
natural killer cell	BP
immune system c	BP
leukocyte differe	BP
leukocyte migrati	BP
leukocyte migrati	BP
hypersensitivity	BP
acute inflammatc	BP
regulation of vasc	BP

production of mo	BP
respiratory burst	BP
nitric oxide produ	BP
leukotriene produ	BP
activation of plas	BP
Factor XII activati	BP
activation of bloo	BP
chronic inflamma	BP
monocyte chemo	BP
mast cell chemot	BP
histamine secreti	BP
serotonin secretio	BP
basophil degranu	BP
somatic diversific	BP
pro-T cell differen	BP
myeloid leukocyt	BP
basophil chemota	BP
platelet degranul	BP
regulation of anti	BP
positive regulatio	BP
negative regulatio	BP
negative regulatio	BP
positive regulatio	BP
negative regulatio	BP
negative regulatio	BP
positive regulatio	BP
negative regulatio	BP
regulation of B ce	BP
regulation of T ce	BP
negative regulatio	BP
regulation of gerr	BP
negative regulatio	BP
positive regulatio	BP
regulation of imm	BP
negative regulatio	BP
positive regulatio	BP
regulation of tole	BP
positive regulatio	BP
positive regulatio	BP
regulation of tole	BP
positive regulatio	BP
regulation of T ce	BP
positive regulatio	BP
regulation of T ce	BP
positive regulatio	BP

regulation of acut	BP
negative regulatic	BP
positive regulatio	BP
regulation of chrc	BP
negative regulatic	BP
respiratory burst	BP
regulation of imm	BP
negative regulatic	BP
positive regulatio	BP
regulation of leuk	BP
negative regulatic	BP
positive regulatio	BP
regulation of leuk	BP
negative regulatic	BP
regulation of cellu	BP
negative regulatic	BP
positive regulatio	BP
negative regulatic	BP
negative regulatic	BP
positive regulatio	BP
regulation of natu	BP
positive regulatio	BP
regulation of cytc	BP
negative regulatic	BP
positive regulatio	BP
regulation of B ce	BP
regulation of T ce	BP
negative regulatic	BP
positive regulatio	BP
negative regulatic	BP
positive regulatio	BP
regulation of den	BP
negative regulatic	BP
positive regulatio	BP
negative regulatic	BP
antigen processin	BP
cell surface patte	BP
cytoplasmic patte	BP
MyD88-depender	BP
MyD88-independ	BP
immune response	BP
innate immune re	BP
regulation of anti	BP
regulation of mye	BP
negative regulatic	BP

positive regulatio	BP
immune response	BP
immune response	BP
immune response	BP
immune response	BP
Fc receptor media	BP
negative regulatio	BP
peptide secretion	BP
negative regulatio	BP
positive regulatio	BP
positive regulatio	BP
negative regulatio	BP
positive regulatio	BP
negative regulatio	BP
positive regulatio	BP
regulation of type	BP
negative regulatio	BP
positive regulatio	BP
negative regulatio	BP
positive regulatio	BP
regulation of T ce	BP
negative regulatio	BP
positive regulatio	BP
positive regulatio	BP
positive regulatio	BP
positive regulatio	BP
positive regulatio	BP
negative regulatio	BP
positive regulatio	BP
negative regulatio	BP
positive regulatio	BP
regulation of acut	BP
negative regulatio	BP
positive regulatio	BP
negative regulatio	BP
positive regulatio	BP
positive regulatio	BP
positive regulatio	BP
positive regulatio	BP
positive regulatio	BP
regulation of B ce	BP
negative regulatio	BP
positive regulatio	BP
negative regulatio	BP
regulation of hum	BP

positive regulatio	BP
regulation of hum	BP
negative regulatio	BP
positive regulatio	BP
tRNA wobble bas	BP
trabecular meshw	BP
response to ische	BP
lipid hydroxylatio	BP
desmosome orga	BP
tRNA N1-guanine	BP
tRNA N2-guanine	BP
tRNA dihydrourid	BP
cyclin K-CDK12 co	CC
cyclin K-CDK13 co	CC
tRNA C5-cytosine	BP
tumor necrosis fa	CC
tRNA threonylcar	BP
ceramide phosph	MF
leukotriene-C(4) l	MF
5'-deoxynucleotic	MF
regionalization	BP
developmental pr	BP
heart morphogen	BP
skeletal muscle co	BP
involuntary skelet	BP
muscle system pr	BP
renal system proc	BP
heart process	BP
respiratory systen	BP
lymph circulation	BP
regulation of syst	BP
regulation of syst	BP
detection of oxyg	BP
regulation of syst	BP
regulation of syst	BP
angiotensin-medi	BP
regulation of vasc	BP
regulation of the	BP
hormonal regulat	BP
regulation of hea	BP
negative regulatio	BP
regulation of hea	BP
positive regulatio	BP
acetylcholine-me	BP
regulation of syst	BP

regulation of syst	BP
positive regulatio	BP
negative regulati	BP
renal water hom	BP
regulation of glor	BP
glomerular filtrati	BP
pressure natriure	BP
renal sodium ion	BP
renal water trans	BP
positive regulatio	BP
regulation of syst	BP
positive regulatio	BP
negative regulati	BP
negative regulati	BP
negative regulati	BP
regulation of vas	BP
detection of nod	BP
heart field specifi	BP
BMP signaling pa	BP
mesodermal-end	BP
negative regulati	BP
primary heart fiel	BP
secondary heart f	BP
determination of	BP
embryonic heart	BP
neural crest cell n	BP
outflow tract sep	BP
membranous sep	BP
muscular septum	BP
outflow tract mo	BP
regulation of anir	BP
endocardium dev	BP
endothelium dev	BP
endocardium mo	BP
cardiac conducti	BP
atrioventricular n	BP
sinoatrial node d	BP
Purkinje myocyte	BP
bundle of His dev	BP
atrioventricular b	BP
coronary vein mo	BP
heart valve devel	BP
atrioventricular v	BP
mitral valve deve	BP
aortic valve devel	BP

pulmonary valve	BP
heart valve morp	BP
aortic valve morp	BP
atrioventricular v	BP
coronary sinus va	BP
mitral valve morp	BP
pulmonary valve	BP
sinoatrial valve m	BP
tricuspid valve mo	BP
heart valve forma	BP
atrioventricular v	BP
mitral valve form	BP
pulmonary valve	BP
tricuspid valve fo	BP
endocardial cushi	BP
epithelial to mese	BP
endocardial cushi	BP
endocardial cushi	BP
cardiac chamber	BP
cardiac chamber	BP
cardiac ventricle	BP
cardiac atrium mo	BP
cardiac atrium fo	BP
cardiac ventricle	BP
cardiac right atriu	BP
cardiac left ventri	BP
cardiac right vent	BP
cardiac right vent	BP
left ventricular ca	BP
right ventricular c	BP
ventricular trabec	BP
ventricular compa	BP
right ventricular c	BP
atrial cardiac mus	BP
ventricular cardia	BP
cardiac ventricle	BP
sinus venosus mo	BP
growth involved i	BP
cardiac muscle tis	BP
post-embryonic c	BP
positive regulatio	BP
negative regulatio	BP
cardiac neural cre	BP
regulation of mer	BP
regulation of tran	BP

positive regulatio	BP
cardioblast anteri	BP
cardioblast migra	BP
regulation of carc	BP
regulation of secc	BP
Notch signaling p	BP
smoothened sign	BP
endocardial cushi	BP
cell migration inv	BP
endocardial cushi	BP
apoptotic process	BP
cardiac septum d	BP
ventricular septu	BP
atrial septum dev	BP
atrial septum prir	BP
atrial septum sec	BP
cardiac muscle hy	BP
negative regulatic	BP
type B pancreatic	BP
pancreatic A cell	BP
pancreatic A cell	BP
type B pancreatic	BP
regulation of extr	BP
positive regulatio	BP
negative regulatic	BP
amino acid transr	BP
keratinocyte deve	BP
corneocyte devel	BP
corneocyte desqu	BP
mesenchymal to	BP
metanephros mo	BP
regulation of mes	BP
cilium movement	BP
proepicardium de	BP
pericardium mor	BP
proepicardium ce	BP
pulmonary myoca	BP
epithelial cilium n	BP
regulation of ciliu	BP
positive regulatio	BP
regulation of ciliu	BP
noradrenergic ne	BP
noradrenergic ne	BP
noradrenergic ne	BP
brainstem develo	BP

lamellipodium as:	BP
establishment of	BP
cell-matrix adhesi	BP
sphingosine-1-ph	BP
epithelial cell mo	BP
apical constrictio	BP
regulation of COP	BP
axis elongation	BP
planar cell polarit	BP
optic vesicle mor	BP
retinal pigment e	BP
neural retina dev	BP
optic cup formati	BP
optic cup structu	BP
establishment of	BP
chondrocyte diffe	BP
chondrocyte hype	BP
endochondral bo	BP
growth plate cart	BP
growth plate cart	BP
growth plate cart	BP
regulation of grov	BP
growth plate cart	BP
growth plate cart	BP
growth plate cart	BP
growth plate cart	BP
chondrocyte deve	BP
DNA helicase acti	MF
AT DNA binding	MF
bent DNA binding	MF
damaged DNA bir	MF
DNA replication c	MF
DNA clamp loade	MF
double-stranded	MF
left-handed Z-DN	MF
satellite DNA binc	MF
single-stranded D	MF
steroid hormone	MF
transcription elor	MF
transcription core	MF
transcription coa	MF
transcription core	MF
telomerase activi	MF
telomerase RNA r	MF
double-stranded	MF

mRNA binding	MF
mRNA 3'-UTR bin	MF
structural constitu	MF
translation initiat	MF
translation elonga	MF
translation releas	MF
peptidyl-prolyl cis	MF
protein disulfide i	MF
microtubule moto	MF
actin monomer b	MF
lysozyme activity	MF
protein-glutamine	MF
antigen binding	MF
alpha-ketoacid de	MF
alpha-1,3-manno:	MF
alpha-N-acetylne	MF
beta-1,3-galactos	MF
beta-1,4-mannos	MF
beta-N-acetylgluc	MF
beta-carotene 15	MF
beta-galactoside	MF
beta-galactoside	MF
beta-ureidopropi	MF
gamma-glutamyl	MF
1-acylglycerol-3-p	MF
1-pyrroline-5-carl	MF
1,4-alpha-glucan	MF
11-beta-hydroxys	MF
2-acylglycerol O-a	MF
1-alkyl-2-acetylgl	MF
2-hydroxyacylsph	MF
2-methylacyl-CoA	MF
3-beta-hydroxy-d	MF
3-hydroxyacyl-Co	MF
3-hydroxybutyrat	MF
3-hydroxyisobuty	MF
3-methyl-2-oxobu	MF
3-oxo-5-alpha-ste	MF
4-aminobutyrate	MF
4-hydroxyphenyl	MF
5-aminolevulinat	MF
6-phosphofructol	MF
6-phosphofructo-	MF
6-pyruvoyltetrahy	MF
ADP-ribosylargini	MF

AMP deaminase a	MF
ATP adenylyltrans	MF
ATP citrate synth	MF
protein C-termina	MF
CDP-diacylglycerc	MF
CDP-diacylglycerc	MF
CTP synthase acti	MF
D-amino-acid oxi	MF
DNA (cytosine-5-)	MF
DNA-directed DN	MF
DNA primase acti	MF
DNA-directed 5'-3	MF
alkylbase DNA N-	MF
DNA-(apurinic or	MF
methylated-DNA-	MF
DNA ligase activit	MF
DNA ligase (ATP)	MF
DNA topoisomera	MF
DNA topoisomera	MF
DNA topoisomera	MF
FMN adenylyltrar	MF
GMP synthase ac	MF
GMP synthase (gl	MF
GPI-anchor transa	MF
NA	NA
GTP cyclohydrola	MF
IMP cyclohydrola	MF
IMP dehydrogena	MF
L-iditol 2-dehydrc	MF
L-iduronidase act	MF
L-serine ammonia	MF
N-acetylgalactosa	MF
N-acetylglucosam	MF
N-acetyllactosam	MF
(N-acetylneurami	MF
N4-(beta-N-acety	MF
NAD+ ADP-ribosy	MF
NAD+ kinase acti	MF
NAD+ synthase (g	MF
NAD+ nucleosida	MF
NADH dehydroge	MF
NAD(P)H dehydrc	MF
NAD(P)+-protein-	MF
NAD(P)+ transhyc	MF
NADPH-hemopro	MF

NADPH:quinone r	MF
RNA-3'-phosphat	MF
RNA-directed DN	MF
RNA-directed 5'-3	MF
RNA ligase (ATP)	MF
(S)-2-hydroxy-aci	MF
UDP-N-acetylgluc	MF
UDP-N-acetylgluc	MF
UDP-N-acetylgluc	MF
UDP-N-acetylgluc	MF
UDP-glucose 4-ep	MF
UDP-glucose 6-de	MF
UDP-glucose:glyc	MF
UTP:glucose-1-ph	MF
acetolactate synt	MF
acetyl-CoA C-acet	MF
acetyl-CoA hydro	MF
acetate-CoA ligas	MF
acetyl-CoA C-acyl	MF
acetyl-CoA carbo	MF
acetylcholinester	MF
acid phosphatase	MF
aconitate hydrat	MF
acyl-CoA dehydro	MF
acyl-CoA oxidase	MF
acylphosphatase	MF
adenine phospho	MF
adenosine deami	MF
adenosine kinase	MF
adenosylhomocy	MF
adenosylmethion	MF
adenylate cyclase	MF
adenylate kinase	MF
N6-(1,2-dicarbox	MF
adenylosuccinate	MF
adenylylsulfate ki	MF
L-alanine:2-oxogl	MF
alcohol dehydrog	MF
alcohol dehydrog	MF
alcohol sulfotran	MF
3-chloroallyl alde	MF
aldehyde dehydr	MF
aldehyde dehydr	MF
aldehyde oxidase	MF
alditol:NADP+ 1-	MF

aldo-keto reducta	MF
aldose 1-epimera	MF
alkaline phosphat	MF
amidase activity	MF
acetyl-CoA:L-glut	MF
L-aminoadipate-s	MF
amidophosphorib	MF
aminoacyl-tRNA h	MF
aminoacylase act	MF
aminomethyltran	MF
arachidonate 5-li	MF
arachidonate 12(	MF
arginase activity	MF
argininosuccinate	MF
argininosuccinate	MF
arginyltransferase	MF
aromatic-L-aminc	MF
aralkylamine N-ac	MF
arylamine N-acet	MF
arylformamidase	MF
aryl sulfotransfer	MF
aryldialkylphosph	MF
arylesterase activ	MF
arylsulfatase acti	MF
asparagine synth	MF
asparaginase acti	MF
aspartate 1-decar	MF
L-aspartate:2-oxc	MF
aspartate carbam	MF
biliverdin reducta	MF
biotin carboxylase	MF
biotin-[acetyl-Co/	MF
biotin-[methylcro	MF
biotin-[methylma	MF
biotin-[propionyl-	MF
bis(5'-nucleosyl)-	MF
bisphosphoglycer	MF
bisphosphoglycer	MF
branched-chain-a	MF
butyryl-CoA dehy	MF
carbamoyl-phosp	MF
carbamoyl-phosp	MF
carbonate dehydı	MF
carbonyl reducta	MF
carnitine O-acety	MF

carnitine O-palmi	MF
catalase activity	MF
cerebroside-sulfa	MF
choline kinase act	MF
cholinesterase ac	MF
choline-phosphat	MF
citrate (Si)-syntha	MF
coproporphyrinog	MF
creatine kinase ac	MF
cyclic-nucleotide	MF
2',3'-cyclic-nucleo	MF
3',5'-cyclic-nucleo	MF
3',5'-cyclic-AMP p	MF
calmodulin-depen	MF
cGMP-stimulated	MF
cGMP-inhibited c	MF
cystathionine bet	MF
cystathionine gan	MF
cytidine deamina	MF
cytochrome-b5 re	MF
cytochrome-c oxi	MF
dCMP deaminase	MF
glycogen debranc	MF
4-alpha-glucanotr	MF
amylo-alpha-1,6-g	MF
deoxyadenosine k	MF
deoxycytidine kin	MF
deoxyguanosine k	MF
deoxyribose-phos	MF
dephospho-CoA k	MF
diacylglycerol cho	MF
diacylglycerol kin	MF
diacylglycerol O-a	MF
diamine N-acetylt	MF
dihydrofolate red	MF
dihydrolipoyl deh	MF
dihydrolipoyllysin	MF
dihydroorotase a	MF
dihydroorotate d	MF
6,7-dihydropterid	MF
dihydropyrimidin	MF
dimethylallyltrans	MF
diphosphomevalo	MF
diphthine synthas	MF
dodecenoyl-CoA d	MF

dopachrome ison	MF
dolichol kinase ac	MF
dolichyl-phosphat	MF
dUTP diphosphat	MF
electron-transfer	MF
endopeptidase ac	MF
ATP-dependent p	MF
aminopeptidase a	MF
carboxypeptidase	MF
metallocarboxype	MF
serine-type carbo	MF
aspartic-type end	MF
cysteine-type enc	MF
calcium-depende	MF
metalloendopept	MF
serine-type endo	MF
threonine-type er	MF
enoyl-CoA hydrat	MF
epoxide hydrolas	MF
estradiol 17-beta	MF
estrone sulfotran	MF
ethanolamine kin	MF
ethanolamine-ph	MF
ethanolaminepho	MF
exo-alpha-sialidas	MF
exopolyphosphat	MF
farnesyl-diphosph	MF
farnesyltranstran	MF
fatty acid synthas	MF
[acyl-carrier-prot	MF
[acyl-carrier-prot	MF
3-oxoacyl-[acyl-ca	MF
3-oxoacyl-[acyl-ca	MF
3-hydroxypalmito	MF
oleoyl-[acyl-carrie	MF
fatty-acyl-CoA syr	MF
ferroxidase activi	MF
ferredoxin-NADP-	MF
ferrochelatase ac	MF
tetrahydrofolylpo	MF
formate-tetrahyd	MF
fructose-2,6-bispl	MF
fructose-bisphosp	MF
fumarate hydrata	MF
fumarylacetoacet	MF

galactokinase act	MF
galactosylceramic	MF
geranyltranstrans	MF
glucan 1,4-alpha-;	MF
glucokinase activi	MF
gluconolactonase	MF
glucosamine-6-pł	MF
glucosamine 6-ph	MF
glucose-6-phosph	MF
glucose-6-phosph	MF
glucose-6-phosph	MF
glucosylceramida	MF
glutamate 5-kinas	MF
glutamate-5-sem	MF
glutamate decarb	MF
glutamate dehydı	MF
glutamate dehydı	MF
glutamate dehydı	MF
glutamate-ammo	MF
glutamate-cystei	MF
glutaminase activ	MF
glutamine-fructos	MF
glutaryl-CoA dehy	MF
glutathione-disul	MF
glutathione synth	MF
glutathione trans	MF
glyceraldehyde-3-	MF
glycerol-3-phosph	MF
glycerol-3-phosph	MF
glycerol-3-phosph	MF
glycerol kinase ac	MF
glycerone kinase	MF
glycine hydroxym	MF
glycogen (starch)	MF
glycine dehydrog	MF
glycolipid mannos	MF
GDP-Man:Man3G	MF
GDP-Man:Man1G	MF
glycylpeptide N-t	MF
guanosine-diphos	MF
guanylate cyclase	MF
guanylate kinase	MF
heme oxygenase	MF
heparan sulfate 2	MF
hexaprenyldihydr	MF

hexokinase activi	MF
histidine ammoni	MF
histidine decarbo	MF
histone acetyltra	MF
histone deacetyla	MF
holocytochrome-	MF
homogentisate 1,	MF
hyaluronongluco	MF
hydroxyacylgluta	MF
hydroxymethylbil	MF
hydroxymethylglu	MF
hydroxymethylglu	MF
hydroxymethylglu	MF
hypoxanthine ph	MF
iduronate-2-sulfa	MF
inorganic diphosp	MF
1-phosphatidylin	MF
phosphatidylinosi	MF
phosphatidylinosi	MF
phosphatidylinosi	MF
inositol-1,4-bisph	MF
inositol-polyphos	MF
inositol-hexakisp	MF
iodide peroxidase	MF
isocitrate dehydr	MF
isocitrate dehydr	MF
isocitrate dehydr	MF
isopentenyl-diph	MF
ketohexokinase a	MF
lactate dehydrog	MF
D-lactate dehydr	MF
L-lactate dehydro	MF
lactose synthase	MF
lactoylglutathion	MF
leukotriene-A4 h	MF
leukotriene-C4 sy	MF
lipoprotein lipase	MF
long-chain-acyl-C	MF
long-chain fatty a	MF
lysine N-acetyltra	MF
malic enzyme act	MF
malate dehydrog	MF
malate dehydrog	MF
malate synthase	MF
mannose-1-phos	MF

mannose-6-phos|	MF
methenyltetrahy(	MF
methionine aden(	MF
methionyl-tRNA f	MF
mRNA (guanine-N	MF
mRNA (nucleosid(	MF
mRNA guanylyltra	MF
methylcrotonoyl-	MF
methylenetetrah\	MF
methylenetetrah\	MF
methylenetetrah\	MF
methylenetetrah\	MF
methylglutaconyl	MF
methylmalonate-	MF
methylmalonyl-C(	MF
methylmalonyl-C(	MF
mevalonate kinas	MF
monooxygenase a	MF
calcidiol 1-monoc	MF
N,N-dimethylanili	MF
dopamine beta-m	MF
kynurenine 3-mo	MF
monophenol mor	MF
peptidylglycine m	MF
phenylalanine 4-r	MF
squalene monoo›	MF
steroid 17-alpha-I	MF
steroid 21-monoc	MF
tryptophan 5-mo	MF
inositol-3-phosph	MF
neolactotetraosyl	MF
nicotinate-nucleo	MF
nicotinate-nucleo	MF
nicotinate phospl	MF
nitric-oxide synth	MF
nuclease activity	MF
endonuclease act	MF
endodeoxyribonu	MF
endoribonuclease	MF
ribonuclease A ac	MF
RNA-DNA hybrid	MF
ribonuclease III a(	MF
ribonuclease P ac	MF
exonuclease activ	MF
phosphodiesteras	MF

deoxyribonucleas	MF
deoxyribonucleas	MF
exoribonuclease a	MF
5'-3' exoribonucle	MF
poly(A)-specific ri	MF
deoxyribonucleas	MF
tRNA-specific ribo	MF
nucleotide diphos	MF
hydrolase activity	MF
alpha,alpha-treha	MF
alpha-amylase ac	MF
alpha-galactosida	MF
alpha-1,4-glucosi	MF
alpha-mannosida	MF
alpha-L-fucosidas	MF
alpha-N-acetylglu	MF
beta-N-acetylhex	MF
beta-galactosidas	MF
beta-glucuronida	MF
beta-mannosidas	MF
chitinase activity	MF
glycoprotein endo	MF
mannosyl-oligosa	MF
mannosyl-oligosa	MF
mannosyl-oligosa	MF
oligosaccharyl tra	MF
N-acetylglucosam	MF
chitobiosyldiphos	MF
dolichyl-diphosph	MF
dolichyl-phospha	MF
dolichyl-phospha	MF
ornithine decarbo	MF
ornithine-oxo-aci	MF
orotate phosphor	MF
orotidine-5'-phos	MF
oxoglutarate deh	MF
pantothenate kin	MF
pantetheine-phos	MF
peptide alpha-N-a	MF
peptide-aspartate	MF
peptidylamidogly	MF
peroxidase activit	MF
glutathione perox	MF
phenylethanolam	MF
phosphatidate cy	MF

phosphatidylchol	MF
phosphatidyletha	MF
phosphatidylserir	MF
phosphoacetylglu	MF
phosphoenolpyru	MF
phosphoenolpyru	MF
phosphoglucomu	MF
phosphomannom	MF
phosphogluconat	MF
phosphoglycerate	MF
phosphoglycerate	MF
phosphoglycerate	MF
phospholipase ac	MF
glycosylphosphat	MF
lysophospholipas	MF
phospholipase A2	MF
phospholipase C a	MF
phospholipase D	MF
phosphomevalon	MF
phosphopantothe	MF
phosphopantothe	MF
phosphopyruvate	MF
phosphoribosylar	MF
phosphoribosylar	MF
phosphoribosylar	MF
phosphoribosylfo	MF
phosphoribosylfo	MF
phosphoribosylar	MF
phosphoribosylgly	MF
1,4-alpha-oligoglu	MF
phosphoserine ph	MF
O-phospho-L-seri	MF
poly(ADP-ribose)	MF
polynucleotide 5'	MF
polynucleotide ac	MF
polypeptide N-ac	MF
polyribonucleotic	MF
porphobilinogen s	MF
procollagen-proli	MF
proline dehydrog	MF
propionyl-CoA ca	MF
prenyltransferase	MF
protein farnesyltr	MF
protein geranylge	MF
CAAX-protein ger	MF

Rab geranylgeran	MF
prostaglandin-en	MF
prostaglandin-D s	MF
protein-arginine	MF
protein C-termina	MF
protein kinase ac	MF
protein histidine	MF
protein serine/th	MF
transmembrane r	MF
3-phosphoinositi	MF
DNA-dependent	MF
AMP-activated pr	MF
calmodulin-deper	MF
elongation factor	MF
myosin light chair	MF
phosphorylase kir	MF
cAMP-dependent	MF
cGMP-dependent	MF
cyclin-dependent	MF
eukaryotic transla	MF
protein kinase C a	MF
calcium-depende	MF
calcium-indepenc	MF
G protein-couple	MF
NF-kappaB-induci	MF
JUN kinase activit	MF
JUN kinase kinase	MF
MAP kinase activi	MF
MAP kinase kinas	MF
MAP kinase kinas	MF
ribosomal proteir	MF
protein serine/th	MF
protein tyrosine k	MF
transmembrane r	MF
non-membrane s	MF
protein-L-isoaspa	MF
protein-lysine 6-c	MF
phosphoprotein p	MF
protein serine/th	MF
calcium-depende	MF
protein tyrosine p	MF
non-membrane s	MF
prenylated protei	MF
oxygen-depender	MF
purine-nucleoside	MF

pyridoxamine-ph	MF
pyrroline-5-carbo	MF
pyruvate carboxy	MF
pyruvate dehydrc	MF
pyruvate dehydrc	MF
pyruvate dehydrc	MF
[pyruvate dehydr	MF
dihydrolipoyllysin	MF
pyruvate kinase a	MF
retinal isomerase	MF
retinol dehydroge	MF
ribokinase activit	MF
ribonucleoside-di	MF
ribose phosphate	MF
ribulose-phospha	MF
ribose-5-phospha	MF
saccharopine deh	MF
saccharopine deh	MF
selenide, water d	MF
sepiapterin reduc	MF
serine C-palmitoy	MF
serine-pyruvate t	MF
spermidine synth	MF
sphingomyelin ph	MF
steroid delta-ison	MF
sterol esterase ac	MF
sterol O-acyltrans	MF
steryl-sulfatase a	MF
succinate-CoA lig	MF
succinate-CoA lig	MF
succinate-semialc	MF
succinyl-CoA hydr	MF
sulfate adenylyltr	MF
sulfinoalanine de	MF
superoxide dismu	MF
thiamine diphosp	MF
thioether S-meth	MF
thioredoxin-disul	MF
thiosulfate sulfur	MF
L-threonine amm	MF
thromboxane-A s	MF
thymidine kinase	MF
thymidylate synth	MF
thyroxine 5'-deio	MF
sedoheptulose-7-	MF

transketolase act	MF
transposase activ	MF
triglyceride lipase	MF
triose-phosphate	MF
tRNA (guanine-N2	MF
tRNA adenylyltrar	MF
aminoacyl-tRNA l	MF
alanine-tRNA liga	MF
arginine-tRNA liga	MF
aspartate-tRNA li	MF
asparagine-tRNA	MF
cysteine-tRNA liga	MF
glutamate-tRNA l	MF
glutamine-tRNA li	MF
glycine-tRNA ligas	MF
histidine-tRNA lig	MF
isoleucine-tRNA li	MF
leucine-tRNA liga:	MF
lysine-tRNA ligase	MF
methionine-tRNA	MF
phenylalanine-tRI	MF
proline-tRNA ligas	MF
serine-tRNA ligas	MF
threonine-tRNA li	MF
tryptophan-tRNA	MF
tyrosine-tRNA liga	MF
valine-tRNA ligase	MF
tryptophan 2,3-di	MF
tubulin-tyrosine l	MF
L-tyrosine:2-oxog	MF
ubiquitin activatir	MF
uracil DNA N-glyc	MF
uridine kinase act	MF
uridine phosphor	MF
uroporphyrinoger	MF
uroporphyrinoger	MF
xanthine dehydrc	MF
xanthine oxidase	MF
xylulokinase activ	MF
enzyme inhibitor	MF
phospholipase inl	MF
protein kinase inl	MF
cyclin-dependent	MF
cAMP-dependent	MF
protein phosphat	MF

protein serine/th	MF
endopeptidase in	MF
cysteine-type end	MF
asialoglycoprotei	MF
complement rece	MF
complement com	MF
complement com	MF
complement com	MF
nuclear receptor	MF
glucocorticoid re	MF
transmembrane s	MF
GABA-A receptor	MF
cytokine recepto	MF
ciliary neurotropl	MF
erythropoietin re	MF
growth hormone	MF
interferon recept	MF
type I interferon	MF
interferon-gamm	MF
interleukin-1 rece	MF
interleukin-1, typ	MF
interleukin-1, typ	MF
interleukin-2 rece	MF
interleukin-3 rece	MF
interleukin-4 rece	MF
interleukin-5 rece	MF
interleukin-6 rece	MF
interleukin-7 rece	MF
interleukin-8 rece	MF
interleukin-9 rece	MF
interleukin-10 re	MF
interleukin-11 re	MF
leukemia inhibito	MF
oncostatin-M rec	MF
prolactin recepto	MF
G protein-couple	MF
extracellularly AT	MF
adrenergic recept	MF
alpha1-adrenergi	MF
alpha2-adrenergi	MF
beta-adrenergic r	MF
beta1-adrenergic	MF
beta2-adrenergic	MF
angiotensin type	MF
bombesin recept	MF

bradykinin recept	MF
calcitonin recepto	MF
cannabinoid rece	MF
chemokine recep	MF
dopamine neurot	MF
prostaglandin rec	MF
prostaglandin D r	MF
prostaglandin E re	MF
prostaglandin F re	MF
thromboxane rec	MF
thromboxane A2	MF
endothelin recept	MF
G protein-couple	MF
galanin receptor a	MF
glucagon recepto	MF
gonadotropin-rel	MF
histamine recepto	MF
ionotropic glutam	MF
AMPA glutamate	MF
NMDA glutamate	MF
leukotriene recep	MF
melanocortin rec	MF
beta-endorphin re	MF
melanocyte-stimu	MF
N-formyl peptide	MF
neuropeptide Y re	MF
olfactory recepto	MF
opioid receptor a	MF
oxytocin receptor	MF
parathyroid horm	MF
platelet activating	MF
G protein-couple	MF
somatostatin rece	MF
tachykinin recept	MF
thyroid-stimulatin	MF
transferrin recept	MF
vasoactive intesti	MF
vasopressin recep	MF
transmembrane r	MF
ephrin receptor a	MF
GPI-linked ephrin	MF
transmembrane-e	MF
epidermal growth	MF
fibroblast growth	MF
hepatocyte growt	MF

insulin-activated	MF
insulin-like growt	MF
macrophage colo	MF
platelet-derived	MF
platelet-derived	MF
platelet-derived	MF
stem cell factor r	MF
vascular endothe	MF
transforming gro	MF
transforming gro	MF
transforming gro	MF
neurotrophin rec	MF
tumor necrosis fa	MF
osmosensor activ	MF
death receptor a	MF
low-density lipop	MF
netrin receptor a	MF
netrin receptor a	MF
scavenger recept	MF
KDEL sequence bi	MF
signal recognitior	MF
signal sequence b	MF
nuclear export sig	MF
peroxisome matr	MF
peroxisome matr	MF
laminin receptor	MF
transmembrane r	MF
MAP-kinase scaff	MF
protein kinase C b	MF
guanyl-nucleotide	MF
GDP-dissociation	MF
Rab GDP-dissocia	MF
Rho GDP-dissocia	MF
GTPase inhibitor	MF
fibroblast growth	MF
type 1 fibroblast	MF
frizzled binding	MF
type 2 fibroblast	MF
Notch binding	MF
patched binding	MF
type II transformi	MF
receptor tyrosine	MF
smoothened binc	MF
Toll binding	MF
death receptor bi	MF

scavenger recept	MF
cytokine receptor	MF
ciliary neurotropl	MF
granulocyte macr	MF
granulocyte color	MF
growth hormone	MF
type I interferon	MF
interferon-gamm	MF
interleukin-2 rece	MF
interleukin-4 rece	MF
interleukin-5 rece	MF
interleukin-6 rece	MF
interleukin-7 rece	MF
interleukin-10 rec	MF
interleukin-11 rec	MF
interleukin-12 rec	MF
leukemia inhibito	MF
interleukin-1 rece	MF
interleukin-1, typ	MF
interleukin-1, typ	MF
interleukin-1 rece	MF
interleukin-8 rece	MF
epidermal growtl	MF
macrophage colo	MF
insulin receptor b	MF
insulin-like growt	MF
transforming grov	MF
platelet-derived g	MF
nerve growth fact	MF
neurotrophin rec	MF
neurotrophin TRk	MF
neurotrophin TRk	MF
neurotrophin TRk	MF
hepatocyte growt	MF
vascular endothe	MF
stem cell factor r	MF
CD40 receptor bi	MF
CD27 receptor bi	MF
ErbB-2 class recep	MF
hormone activity	MF
gonadotropin hor	MF
neuropeptide hor	MF
structural molecu	MF
structural constit	MF
extracellular mat	MF

structural constit	MF
transporter activi	MF
inositol 1,4,5-tris	MF
intracellular cycli	MF
intracellular cAM	MF
intracellular cGM	MF
volume-sensitive	MF
calcium activated	MF
intracellular sodi	MF
intracellular calci	MF
extracellular ligar	MF
excitatory extrac	MF
extracellularly glu	MF
inhibitory extrace	MF
inward rectifier p	MF
gap junction char	MF
voltage-gated ion	MF
voltage-gated cal	MF
calcium channel r	MF
voltage-gated chl	MF
voltage-gated soc	MF
voltage-gated pot	MF
A-type (transient	MF
delayed rectifier	MF
open rectifier pot	MF
anion channel act	MF
chloride channel	MF
intracellularly ATI	MF
cation channel ac	MF
potassium chann	MF
sodium channel a	MF
amine transmem	MF
acetylcholine trar	MF
amino acid:proto	MF
amino acid:sodiu	MF
high-affinity argin	MF
L-histidine transn	MF
high-affinity lysin	MF
neutral amino aci	MF
proline:sodium sy	MF
L-tyrosine transm	MF
creatine transme	MF
creatine:sodium	MF
dicarboxylic acid	MF
L-glutamate trans	MF

high-affinity gluta	MF
inorganic phosph	MF
high-affinity inorg	MF
lipid transporter a	MF
long-chain fatty a	MF
neurotransmitter	MF
neurotransmitter	MF
dopamine:sodium	MF
gamma-aminobu	MF
norepinephrine:s	MF
serotonin:sodium	MF
nucleoside transr	MF
nucleotide-sugar	MF
organic acid trans	MF
organic acid:sodiu	MF
oxygen carrier ac	MF
purine nucleobas	MF
ATP transmembra	MF
carbohydrate:pro	MF
fructose transme	MF
galactose transm	MF
glucose transmer	MF
low-affinity gluco	MF
myo-inositol tran	MF
myo-inositol:prot	MF
myo-inositol:sodi	MF
taurine transmem	MF
taurine:sodium sy	MF
water transmemb	MF
copper ion transn	MF
iron ion transmer	MF
manganese ion tr	MF
zinc ion transmer	MF
calcium transmer	MF
sodium:potassiur	MF
glucose:sodium s	MF
nucleoside:sodiur	MF
proton-depender	MF
calcium:sodium a	MF
sodium:phosphat	MF
inorganic anion e	MF
CMP-N-acetylneu	MF
GDP-fucose trans	MF
UDP-galactose tra	MF
UDP-glucose tran	MF

UDP-glucuronic a	MF
UDP-N-acetylgluc	MF
UDP-N-acetylgala	MF
UDP-xylose trans	MF
ATP:ADP antiport	MF
pyruvate seconda	MF
soluble NSF attac	MF
SNAP receptor ac	MF
steroid binding	MF
androgen binding	MF
vitamin D binding	MF
retinoid binding	MF
11-cis retinal binc	MF
all-trans retinal bi	MF
fatty acid binding	MF
copper ion bindin	MF
detection of calci	BP
collagen binding	MF
cytoskeletal regul	MF
insulin-like growt	MF
profilin binding	MF
tropomyosin binc	MF
macrolide binding	MF
FK506 binding	MF
galactose binding	MF
glucose binding	MF
mannose binding	MF
glycosaminoglyca	MF
hyaluronic acid bi	MF
folic acid binding	MF
phospholipid binc	MF
calcium-depende	MF
1-phosphatidylin	MF
phosphatidylinosi	MF
phosphatidylinosi	MF
phospholipid trar	MF
odorant binding	MF
fibrinogen compl	CC
membrane attack	CC
collagen trimer	CC
collagen type XV	CC
fibrillar collagen t	CC
collagen type I tri	CC
collagen type II tr	CC
collagen type III t	CC

collagen type IV t	CC
collagen type V tr	CC
collagen type VI t	CC
collagen type VII	CC
collagen type VIII	CC
collagen type XI t	CC
collagen type IX t	CC
collagen type XII	CC
collagen type XIV	CC
collagen type XVI	CC
collagen type XIII	CC
basement membr	CC
laminin-1 comple	CC
laminin-2 comple	CC
laminin-3 comple	CC
laminin-5 comple	CC
interstitial matrix	CC
intracellular	CC
lamin filament	CC
integral compone	CC
nuclear envelope	CC
annulate lamellae	CC
nuclear lamina	CC
nucleolar ribonuc	CC
nuclear pre-replic	CC
replication fork	CC
alpha DNA polym	CC
DNA replication fa	CC
DNA replication fa	CC
nuclear origin of r	CC
RNA polymerase	CC
RNA polymerase	CC
transcription regu	CC
RNA polymerase	CC
transcription fact	CC
Ada2/Gcn5/Ada3	CC
transcription fact	CC
transcription fact	CC
transcription fact	CC
transcription fact	CC
chromatin silenci	CC
anaphase-promot	CC
spliceosomal com	CC
U5 snRNP	CC
U7 snRNP	CC

U2-type spliceoso	CC
U1 snRNP	CC
U2 snRNP	CC
U4 snRNP	CC
U6 snRNP	CC
U12-type spliceos	CC
U4atac snRNP	CC
U6atac snRNP	CC
telomerase holoe	CC
chiasma	CC
recombination no	CC
late recombinatio	CC
pericentric hetero	CC
perichromatin fib	CC
nucleolus organiz	CC
small nucleolar ri	CC
RNA polymerase	CC
mitochondrial en	CC
mitochondrial ou	CC
TIM23 mitochond	CC
m-AAA complex	CC
mitochondrial res	CC
mitochondrial res	CC
mitochondrial res	CC
mitochondrial res	CC
mitochondrial res	CC
mitochondrial pro	CC
mitochondrial pro	CC
mitochondrial pe	CC
mitochondrial int	CC
mitochondrial ma	CC
gamma DNA poly	CC
mitochondrial rib	CC
mitochondrial lar	CC
mitochondrial sm	CC
endosome	CC
late endosome	CC
multivesicular bo	CC
vacuole	CC
vacuolar membra	CC
autophagosome	CC
peroxisome	CC
peroxisomal mem	CC
integral compone	CC
peroxisomal matr	CC

Sec61 translocon	CC
signal recognitior	CC
signal recognitior	CC
signal peptidase c	CC
endoplasmic retic	CC
rough endoplasm	CC
endoplasmic retic	CC
Golgi stack	CC
Golgi lumen	CC
Golgi medial ciste	CC
Golgi-associated v	CC
cis-Golgi network	CC
trans-Golgi netwo	CC
centrosome	CC
centriole	CC
microtubule orga	CC
aster	CC
spindle	CC
actomyosin contr	CC
polar microtubule	CC
kinetochore micro	CC
chaperonin-conta	CC
hemoglobin comp	CC
heterotrimeric G-	CC
proteasome regu	CC
proteasome core	CC
ribosome	CC
polysome	CC
mRNA cap bindin	CC
nuclear cap bindi	CC
mRNA cleavage a	CC
mRNA cleavage s	CC
mRNA cleavage fa	CC
eukaryotic transla	CC
eukaryotic transla	CC
eukaryotic transla	CC
eukaryotic transla	CC
nascent polypept	CC
axonemal dynein	CC
muscle myosin co	CC
troponin complex	CC
muscle thin filam	CC
striated muscle m	CC
striated muscle th	CC
cytoplasmic dyne	CC

dynactin complex	CC
kinesin complex	CC
plus-end kinesin c	CC
microtubule	CC
microtubule asso	CC
spindle microtubu	CC
axonemal microtu	CC
nuclear microtub	CC
cytoplasmic micro	CC
intermediate filar	CC
neurofilament	CC
actin filament	CC
Arp2/3 protein co	CC
potassium:protor	CC
sodium:potassiur	CC
voltage-gated cal	CC
acetylcholine-gat	CC
interleukin-2 rece	CC
interleukin-5 rece	CC
interleukin-6 rece	CC
interleukin-13 rec	CC
insulin receptor c	CC
oncostatin-M rec	CC
caveola	CC
microvillus	CC
brush border	CC
clathrin-coated pi	CC
cell-cell junction	CC
adherens junctior	CC
spot adherens jur	CC
zonula adherens	CC
fascia adherens	CC
septate junction	CC
gap junction	CC
connexin comple:	CC
focal adhesion	CC
muscle tendon ju	CC
cilium	CC
axoneme	CC
cell cortex	CC
septin ring	CC
phosphatidylinosi	CC
phosphatidylinosi	CC
phosphatidylinosi	CC
6-phosphofructok	CC

mitochondrial alp	CC
acetolactate synt	CC
cAMP-dependent	CC
CAAX-protein ger	CC
calcium- and calm	CC
calcineurin compl	CC
protein kinase CK	CC
DNA-dependent p	CC
glycine cleavage c	CC
phosphorylase kin	CC
protein farnesyltr	CC
mitochondrial pyr	CC
Rab-protein gerar	CC
serine-pyruvate a	CC
ribonucleoside-di	CC
carbohydrate met	BP
polysaccharide m	BP
glycogen metabo	BP
glycogen biosynth	BP
regulation of glyc	BP
glycogen cataboli	BP
regulation of glyc	BP
starch catabolic p	BP
disaccharide meta	BP
sucrose metaboli	BP
sucrose biosynthe	BP
lactose biosynthe	BP
trehalose metabo	BP
trehalose catabol	BP
monosaccharide m	BP
xylulose metaboli	BP
xylulose catabolic	BP
xylulose biosynth	BP
fructose metabol	BP
fructose 6-phosph	BP
fructose 2,6-bisph	BP
fucose metabolic	BP
glucose metaboli	BP
glucose catabolic	BP
UDP-glucose met	BP
galactose metabo	BP
mannose metabo	BP
D-ribose metabol	BP
5-phosphoribose	BP
inositol metaboli	BP

inositol biosynthe	BP
glycosaminoglyca	BP
glycosaminoglyca	BP
proteoglycan met	BP
chitin catabolic pı	BP
glucosamine met	BP
glucosamine cata	BP
N-acetylglucosam	BP
N-acetylglucosam	BP
N-acetylglucosam	BP
UDP-N-acetylgluc	BP
UDP-N-acetylgluc	BP
UDP-N-acetylgluc	BP
N-acetylmannosa	BP
N-acetylneuramir	BP
CMP-N-acetylneu	BP
sorbitol metaboli	BP
sorbitol biosynthe	BP
sorbitol catabolic	BP
UDP-glucuronate	BP
alcohol metabolic	BP
ethanol metaboli	BP
ethanol catabolic	BP
ethanol oxidation	BP
glycerol metaboli	BP
glycerol-3-phosph	BP
cellular aldehyde	BP
organic acid meta	BP
acetyl-CoA metab	BP
acetyl-CoA biosyr	BP
acetyl-CoA biosyr	BP
lactate metabolic	BP
pyruvate metabo	BP
generation of pre	BP
gluconeogenesis	BP
glycolytic process	BP
glyoxylate cycle	BP
pentose-phospha	BP
tricarboxylic acid	BP
citrate metabolic	BP
isocitrate metabc	BP
2-oxoglutarate m	BP
succinyl-CoA met	BP
succinate metabc	BP
fumarate metabc	BP

oxaloacetate met	BP
malate metabolic	BP
regulation of carb	BP
regulation of glyc	BP
regulation of gluc	BP
energy reserve m	BP
glycerol biosynth	BP
NADH oxidation	BP
acetaldehyde me	BP
oxidative phosph	BP
mitochondrial ele	BP
mitochondrial ele	BP
mitochondrial ele	BP
mitochondrial ele	BP
ferredoxin metab	BP
glycerophosphate	BP
purine nucleobas	BP
guanine catabolic	BP
inosine catabolic	BP
adenosine catabo	BP
deoxyadenosine (	BP
purine nucleotide	BP
purine ribonucleo	BP
AMP biosynthetic	BP
adenine salvage	BP
dAMP biosynthet	BP
cAMP biosynthet	BP
ADP biosynthetic	BP
dADP biosyntheti	BP
dADP phosphoryl	BP
dATP biosyntheti	BP
GMP biosynthetic	BP
guanine salvage	BP
cGMP biosynthet	BP
GTP biosynthetic	BP
dGDP biosyntheti	BP
dGDP phosphoryl	BP
IMP biosynthetic	BP
'de novo' IMP bio	BP
ITP catabolic proc	BP
purine nucleotide	BP
AMP catabolic pr	BP
cAMP catabolic p	BP
dGTP catabolic pr	BP
IMP catabolic pro	BP

pyrimidine nucle	BP
'de novo' pyrimid	BP
pyrimidine nucle	BP
thymine catabolic	BP
5-methylcytosine	BP
uracil catabolic p	BP
pyrimidine nucle	BP
thymidine catabo	BP
uridine catabolic	BP
pyrimidine nucle	BP
UMP biosynthetic	BP
UDP biosynthetic	BP
dUMP biosynthet	BP
UTP biosynthetic	BP
dTMP biosynthet	BP
CTP biosynthetic	BP
CMP catabolic pr	BP
dCMP catabolic p	BP
dCTP catabolic pr	BP
CTP catabolic pro	BP
UDP catabolic pr	BP
UDP-glucose cata	BP
DNA metabolic p	BP
DNA replication	BP
DNA-dependent I	BP
mitochondrial DN	BP
DNA topological	BP
DNA ligation	BP
pre-replicative co	BP
DNA unwinding ir	BP
DNA replication,	BP
DNA replication i	BP
DNA strand elong	BP
leading strand el	BP
lagging strand el	BP
regulation of DN	BP
RNA-dependent I	BP
DNA repair	BP
regulation of DN	BP
transcription-cou	BP
base-excision rep	BP
base-excision rep	BP
base-excision rep	BP
base-excision rep	BP
base-excision rep	BP

nucleotide-excisio	BP
pyrimidine dimer	BP
nucleotide-excisio	BP
nucleotide-excisio	BP
nucleotide-excisio	BP
nucleotide-excisio	BP
nucleotide-excisio	BP
mismatch repair	BP
postreplication re	BP
double-strand bre	BP
double-strand bre	BP
DNA modification	BP
DNA methylation	BP
DNA dealkylation	BP
DNA catabolic pro	BP
apoptotic DNA fra	BP
DNA recombinati	BP
meiotic gene con	BP
mitotic recombin	BP
transposition, DN	BP
DNA packaging	BP
chromatin assem	BP
nucleosome asse	BP
DNA replication-c	BP
DNA replication-i	BP
nucleosome disas	BP
chromatin remod	BP
chromatin silenci	BP
DNA methylation	BP
chromatin silenci	BP
regulation of gen	BP
transcription, DN	BP
DNA-templated tr	BP
DNA-templated tr	BP
DNA-templated tr	BP
regulation of tran	BP
regulation of tran	BP
transcription by R	BP
transcription initi	BP
transcription elon	BP
termination of RN	BP
rRNA processing	BP
transcription by R	BP
transcription initi	BP
transcription elon	BP

termination of RN	BP
7-methylguanosir	BP
mRNA splice site	BP
mRNA polyadeny	BP
mRNA cleavage	BP
adenosine to inos	BP
transcription by F	BP
transcription initi	BP
termination of RN	BP
tRNA splicing, via	BP
mitochondrial tra	BP
transcription initi	BP
transcription elor	BP
termination of mi	BP
RNA processing	BP
mRNA 3'-end pro	BP
tRNA metabolic p	BP
tRNA modificatio	BP
mRNA catabolic p	BP
RNA localization	BP
RNA import into r	BP
RNA export from	BP
mRNA export fro	BP
rRNA export from	BP
snRNA export fro	BP
tRNA export from	BP
translation	BP
translational initi	BP
translational elon	BP
translational term	BP
regulation of tran	BP
tRNA aminoacyla	BP
alanyl-tRNA amin	BP
arginyl-tRNA amir	BP
asparaginyl-tRNA	BP
aspartyl-tRNA am	BP
cysteinyl-tRNA an	BP
glutamyl-tRNA an	BP
glutaminyl-tRNA a	BP
glycyl-tRNA amin	BP
histidyl-tRNA ami	BP
isoleucyl-tRNA an	BP
leucyl-tRNA amin	BP
lysyl-tRNA amino	BP
methionyl-tRNA a	BP

phenylalanyl-tRN	BP
prolyl-tRNA amin	BP
seryl-tRNA amino	BP
threonyl-tRNA an	BP
tryptophanyl-tRN	BP
tyrosyl-tRNA ami	BP
valyl-tRNA amino	BP
regulation of tran	BP
regulation of tran	BP
regulation of tran	BP
regulation of tran	BP
protein folding	BP
'de novo' protein	BP
cellular protein m	BP
signal peptide pro	BP
protein phosphor	BP
negative regulatio	BP
protein dephosph	BP
protein ADP-ribos	BP
protein acetylatio	BP
N-terminal protei	BP
internal protein a	BP
protein deacetyla	BP
protein sulfation	BP
peptidyl-tyrosine	BP
protein methylati	BP
N-terminal protei	BP
C-terminal protei	BP
protein demethyl	BP
protein glycosylat	BP
protein N-linked g	BP
dolichol-linked ol	BP
dolichyl diphosph	BP
oligosaccharide-li	BP
N-glycan processi	BP
protein O-linked g	BP
protein lipidation	BP
N-terminal protei	BP
C-terminal protei	BP
GPI anchor metab	BP
GPI anchor biosyn	BP
GPI anchor releas	BP
proteolysis	BP
membrane protei	BP
protein monoubic	BP

protein quality cc	BP
glycoprotein cata	BP
protein deglycosy	BP
peptide metaboli	BP
cellular amino aci	BP
regulation of cellu	BP
arginine metaboli	BP
arginine biosynth	BP
arginine catabolic	BP
asparagine metak	BP
asparagine biosyr	BP
aspartate metabc	BP
aspartate biosynt	BP
aspartate catabol	BP
cysteine metabol	BP
cysteine biosynth	BP
glutamate metab	BP
glutamate biosyn	BP
glutamate catabc	BP
glutamate catabc	BP
glutamate decark	BP
glutamine metab	BP
glutamine biosyn	BP
glutamine catabo	BP
glycine metabolic	BP
glycine biosynthe	BP
glycine catabolic	BP
histidine metabol	BP
histidine catabol	BP
isoleucine metab	BP
isoleucine catabo	BP
leucine metabolic	BP
leucine catabolic	BP
lysine catabolic p	BP
methionine meta	BP
S-adenosylmethic	BP
S-adenosylmethic	BP
L-phenylalanine n	BP
L-phenylalanine c	BP
proline metabolic	BP
proline biosynthe	BP
proline catabolic	BP
L-serine metaboli	BP
L-serine biosynth	BP
L-serine catabolic	BP

threonine metab	BP
threonine catabo	BP
tryptophan metal	BP
tryptophan catab	BP
tyrosine metaboli	BP
tyrosine catabolic	BP
valine metabolic	BP
valine catabolic p	BP
cellular modified	BP
cellular biogenic	BP
amino-acid betair	BP
amino-acid betair	BP
ethanolamine me	BP
acetylcholine cat	BP
melanin metaboli	BP
melanin biosynth	BP
catecholamine m	BP
octopamine bios	BP
thyroid hormone	BP
ornithine metabo	BP
ornithine biosynt	BP
polyamine metab	BP
polyamine biosyn	BP
spermine biosynt	BP
polyamine catabo	BP
creatine metabol	BP
creatine biosynth	BP
protein targeting	BP
protein import in	BP
NLS-bearing prot	BP
ribosomal proteir	BP
protein export fr	BP
protein targeting	BP
cotranslational pr	BP
SRP-dependent c	BP
SRP-dependent c	BP
SRP-dependent c	BP
posttranslational	BP
protein retention	BP
protein targeting	BP
protein targeting	BP
vacuolar protein	BP
protein targeting	BP
protein targeting	BP
protein processin	BP

fatty acid beta-ox	BP
acyl-CoA metabol	BP
neutral lipid meta	BP
acylglycerol meta	BP
monoacylglycerol	BP
triglyceride metal	BP
triglyceride mobil	BP
membrane lipid n	BP
phospholipid met	BP
phosphatidyletha	BP
phospholipid trar	BP
glycerophospholi	BP
diacylglycerol bio	BP
phosphatidic acid	BP
phosphatidylglyce	BP
phosphatidylchol	BP
CDP-choline path	BP
phosphatidylserir	BP
phosphatidylserir	BP
phosphatidylserir	BP
phosphatidylinosi	BP
glycerol ether me	BP
platelet activating	BP
glycolipid metabo	BP
sphingolipid meta	BP
3-keto-sphinganir	BP
sphinganine meta	BP
sphinganine-1-ph	BP
sphinganine-1-ph	BP
sphingosine meta	BP
ceramide metabo	BP
glycosylceramide	BP
glucosylceramide	BP
glucosylceramide	BP
glucosylceramide	BP
galactosylceramic	BP
galactosylceramic	BP
sphingomyelin m	BP
sphingomyelin ca	BP
sphingomyelin bi	BP
glycosphingolipid	BP
glycosphingolipid	BP
ganglioside catab	BP
icosanoid metabo	BP
leukotriene meta	BP

prostanoid metak	BP
prostaglandin me	BP
bile acid biosynth	BP
C21-steroid horm	BP
progesterone bio	BP
androgen biosynt	BP
estrogen biosynth	BP
glucocorticoid bic	BP
mineralocorticoic	BP
steroid catabolic	BP
cholesterol catab	BP
androgen catabol	BP
estrogen cataboli	BP
glucocorticoid cat	BP
sesquiterpenoid r	BP
isoprenoid metak	BP
cellular aromatic	BP
eye pigment bios	BP
tetrahydrobiopte	BP
one-carbon meta	BP
NADH metabolic	BP
nicotinamide ribc	BP
NADP metabolic p	BP
NADPH regenerat	BP
NADP biosyntheti	BP
NADP catabolic p	BP
ubiquinone meta	BP
ubiquinone biosy	BP
FAD biosynthetic	BP
glutathione meta	BP
glutathione biosy	BP
glutathione catab	BP
nucleoside phosp	BP
ATP biosynthetic	BP
AMP phosphoryla	BP
ATP generation fr	BP
folic acid-contain	BP
vitamin metaboli	BP
biotin metabolic p	BP
riboflavin metabc	BP
thiamine metabo	BP
vitamin A metabc	BP
Mo-molybdopter	BP
porphyrin-contair	BP
porphyrin-contair	BP

uroporphyrinoge	BP
succinyl-CoA path	BP
protoporphyrinog	BP
heme biosyntheti	BP
heme A biosynthe	BP
heme oxidation	BP
bilirubin conjugat	BP
sulfur compound	BP
phosphate-contai	BP
polyphosphate m	BP
polyphosphate ca	BP
superoxide metal	BP
xenobiotic metab	BP
nitrogen compou	BP
regulation of nitr	BP
nitric oxide biosy	BP
cation transport	BP
potassium ion tra	BP
sodium ion transp	BP
phosphate ion tra	BP
anion transport	BP
chloride transpor	BP
cobalt ion transpo	BP
copper ion transp	BP
iron ion transport	BP
manganese ion tr	BP
zinc ion transport	BP
water transport	BP
dicarboxylic acid	BP
neurotransmitter	BP
serotonin transpo	BP
mitochondrial tra	BP
tricarboxylic acid	BP
mitochondrial citr	BP
acyl carnitine tran	BP
pyruvate transpo	BP
mitochondrial pyr	BP
mitochondrial cal	BP
carnitine shuttle	BP
drug transmembr	BP
oligopeptide tran	BP
extracellular tran	BP
nucleotide transp	BP
pyrimidine nucleo	BP
amino acid transp	BP

asparagine transp	BP
glutamine transp	BP
cellular ion home	BP
cellular metal ion	BP
cellular cadmium	BP
cellular copper io	BP
cellular iron ion h	BP
intracellular sequ	BP
cellular zinc ion h	BP
cellular sodium io	BP
cell volume home	BP
regulation of pH	BP
intracellular prot	BP
exocytosis	BP
endoplasmic retic	BP
retrograde vesicl	BP
intra-Golgi vesicle	BP
post-Golgi vesicle	BP
Golgi to plasma m	BP
Golgi to endosom	BP
Golgi to vacuole t	BP
receptor-mediate	BP
vesicle budding fr	BP
vesicle targeting	BP
vesicle docking in	BP
vesicle fusion	BP
pinocytosis	BP
phagocytosis	BP
phagocytosis, rec	BP
phagocytosis, eng	BP
nucleocytoplasmi	BP
autophagy	BP
activation of cyst	BP
cellular compone	BP
activation-induce	BP
inflammatory cell	BP
substrate-depenc	BP
substrate-depenc	BP
substrate-depenc	BP
negative regulatic	BP
muscle contractic	BP
regulation of mus	BP
smooth muscle c	BP
regulation of smc	BP
striated muscle c	BP

regulation of stria	BP
induction by virus	BP
syncytium format	BP
acute-phase resp	BP
complement acti	BP
complement acti	BP
complement acti	BP
humoral immune	BP
positive regulatio	BP
positive regulatio	BP
cellular defense r	BP
response to osmo	BP
hypotonic respon	BP
hyperosmotic res	BP
DNA damage indu	BP
DNA damage resp	BP
DNA damage resp	BP
response to oxida	BP
response to lipid	BP
ER overload respo	BP
ER-nucleus signal	BP
response to unfol	BP
positive regulatio	BP
response to stero	BP
cellular response	BP
organelle organiz	BP
nucleus organizat	BP
nuclear envelope	BP
nuclear pore orga	BP
nucleolus organiz	BP
telomere mainter	BP
mitochondrion or	BP
mitochondrial me	BP
inner mitochondr	BP
plasma membran	BP
cytoskeleton orga	BP
actin ubiquitinati	BP
actin filament org	BP
microtubule-base	BP
microtubule-base	BP
microtubule depo	BP
microtubule nucl	BP
tubulin complex a	BP
post-chaperonin t	BP
negative regulatio	BP

cytoplasm organi	BP
endoplasmic retic	BP
Golgi organizatior	BP
peroxisome orgar	BP
endosome organi	BP
vacuole organizat	BP
vacuolar transpor	BP
vacuolar acidifica	BP
protein catabolic	BP
lysosome organiz	BP
lysosomal transp	BP
lysosomal lumen	BP
cell-cell junction a	BP
cell-substrate jun	BP
cell cycle arrest	BP
spindle organizati	BP
mitotic spindle or	BP
spindle assembly	BP
spindle assembly	BP
chromosome seg	BP
male meiosis chr	BP
sister chromatid c	BP
regulation of siste	BP
mitotic sister chr	BP
female meiosis si	BP
positive regulatio	BP
mitotic chromosc	BP
mitotic nuclear er	BP
mitotic chromosc	BP
mitotic metaphas	BP
mitotic nuclear er	BP
regulation of mit	BP
traversing start c	BP
metaphase/anapl	BP
mitotic cell cycle	BP
mitotic spindle as	BP
mitotic G2 DNA d	BP
regulation of exit	BP
nuclear migratior	BP
centrosome cycle	BP
centriole replicati	BP
mitotic centrosor	BP
male meiosis cyt	BP
endomitotic cell c	BP
meiosis I	BP

synapsis	BP
synaptonemal co	BP
reciprocal meioti	BP
male meiotic nuc	BP
male meiosis I	BP
female meiotic nu	BP
female meiosis I	BP
cell communicati	BP
cell adhesion	BP
homophilic cell a	BP
heterophilic cell-c	BP
neuron cell-cell a	BP
leukocyte cell-cel	BP
cell-matrix adhesi	BP
calcium-indepenc	BP
negative regulatio	BP
establishment or	BP
enzyme linked re	BP
receptor guanylyl	BP
transmembrane r	BP
activation of tran	BP
signal complex as	BP
epidermal growth	BP
epidermal growth	BP
negative regulatio	BP
regulation of epic	BP
transmembrane r	BP
transforming gro	BP
transforming gro	BP
common-partner	BP
SMAD protein co	BP
transmembrane r	BP
G protein-couple	BP
adenylate cyclase	BP
activation of ade	BP
adenylate cyclase	BP
adenylate cyclase	BP
negative regulatio	BP
adenylate cyclase	BP
adenylate cyclase	BP
adenylate cyclase	BP
adenylate cyclase	BP
G protein-couple	BP
phospholipase C-	BP
activation of pho	BP

positive regulatio	BP
protein kinase C-a	BP
phospholipase C-a	BP
phospholipase C-a	BP
phospholipase C-a	BP
serotonin recepto	BP
dopamine recept	BP
G protein-couple	BP
gamma-aminobu	BP
glutamate recept	BP
G protein-couple	BP
tachykinin recept	BP
neuropeptide sigi	BP
Notch signaling p	BP
Notch receptor pi	BP
Wnt signaling pat	BP
smoothened sign	BP
patched ligand m	BP
positive regulatio	BP
osmosensory sigr	BP
I-kappaB kinase/N	BP
activation of NF-k	BP
I-kappaB phospho	BP
cytoplasmic sequ	BP
JNK cascade	BP
activation of JNKK	BP
activation of JUN	BP
JUN phosphorylat	BP
tyrosine phospho	BP
nitric oxide media	BP
small GTPase me	BP
Ras protein signal	BP
Rho protein signa	BP
chemical synaptic	BP
neurotransmitter	BP
neuron-neuron sy	BP
synaptic transmis	BP
neuromuscular sy	BP
multicellular orga	BP
gamete generatic	BP
germ cell develop	BP
spermatogenesis	BP
spermatogonial c	BP
spermatid develo	BP
sperm axoneme a	BP

spermatid nucleu	BP
spermatid nucleu	BP
female gamete ge	BP
vitellogenesis	BP
pole plasm assem	BP
insemination	BP
single fertilizatior	BP
binding of sperm	BP
acrosome reactio	BP
penetration of zo	BP
fusion of sperm t	BP
egg activation	BP
pronuclear fusion	BP
regulation of mit	BP
zygotic specificati	BP
zygotic determina	BP
thorax and anteri	BP
determination of	BP
gastrulation	BP
ventral midline de	BP
segment specifica	BP
compartment pat	BP
anterior compart	BP
posterior compar	BP
ectoderm develo	BP
nervous system d	BP
neuroblast fate d	BP
ganglion mother	BP
glial cell fate dete	BP
neuroblast prolife	BP
negative regulatio	BP
axonogenesis	BP
axon guidance	BP
axon target recog	BP
axonal fasciculati	BP
synapse assembly	BP
central nervous s	BP
ventral midline de	BP
brain developme	BP
peripheral nervou	BP
sensory organ de	BP
salivary gland dev	BP
salivary gland mo	BP
foregut morphog	BP
hindgut morphog	BP

regulation of rhod	BP
endoderm develo	BP
endodermal cell f	BP
midgut developm	BP
visceral mesoder	BP
posterior midgut	BP
mesoderm develo	BP
ectoderm and me	BP
mesodermal cell	BP
mesodermal cell	BP
gonadal mesoder	BP
heart developme	BP
mesoderm migra	BP
adult heart devel	BP
myoblast fate det	BP
skeletal muscle ti	BP
myoblast fusion	BP
muscle cell fate d	BP
visceral muscle de	BP
somatic muscle d	BP
neuromuscular ju	BP
establishment of	BP
sex determinatio	BP
sex differentiatio	BP
dosage compensa	BP
female pregnancy	BP
embryo implanta	BP
parturition	BP
aging	BP
cell aging	BP
respiratory gaseo	BP
digestion	BP
excretion	BP
body fluid secreti	BP
lactation	BP
blood coagulatio	BP
blood coagulatio	BP
blood coagulatio	BP
hemostasis	BP
sensory perceptio	BP
visual perception	BP
phototransductio	BP
phototransductio	BP
phototransductio	BP
sensory perceptio	BP

sensory perceptio	BP
sensory perceptio	BP
behavior	BP
learning or memo	BP
learning	BP
memory	BP
short-term memo	BP
long-term memoı	BP
mating behavior	BP
mating	BP
copulation	BP
negative regulati	BP
rhythmic behavio	BP
circadian rhythm	BP
ultradian rhythm	BP
grooming behavic	BP
locomotory beha	BP
adult walking beł	BP
feeding behavior	BP
visual behavior	BP
chemosensory be	BP
mechanosensory	BP
beta-catenin bind	MF
regulation of hea	BP
G protein-couple	MF
synaptic vesicle	CC
protein C-termin	MF
transcription elor	CC
cyclin/CDK positi	CC
monocarboxylic a	MF
tRNA processing	BP
high-density lipo	MF
cell recognition	BP
neuron recognitic	BP
synaptic target re	BP
intracellular ferrit	CC
motor neuron ax	BP
axon guidance re	MF
enzyme activator	MF
calcium sensitive	MF
male courtship be	BP
female courtship	BP
sensory organ bo	BP
mitochondrial fus	BP
eye pigment gran	BP

chitin binding	MF
Toll signaling patl	BP
regulation of acti	BP
establishment of	BP
glutamate recept	MF
ornithine decarbo	MF
guanylate cyclase	CC
voltage-gated pot	CC
mesodermal cell	BP
translation termi	MF
N-acetyltransfera	MF
phosphoric dieste	MF
growth factor act	MF
axo-dendritic trar	BP
anterograde axor	BP
retrograde axona	BP
spectrin	CC
cytoskeletal prote	MF
cytoskeletal anch	MF
DNA-dependent /	MF
5S rRNA binding	MF
protein localizatic	BP
alcohol dehydrog	MF
galactoside 2-alpl	MF
UDP-glucose:hex	MF
N-acetyllactosam	MF
alpha-methylacyl-	MF
nicotinamide N-m	MF
peptide-methioni	MF
sarcosine oxidase	MF
prostaglandin-I sy	MF
sphinganine-1-ph	MF
N-acetyllactosam	MF
thiopurine S-metl	MF
ceramide glucosy	MF
ubiquinol-cytochr	MF
4-alpha-hydroxyt	MF
acetylesterase ac	MF
quercetin 2,3-dio	MF
primary amine ox	MF
transcription fact	MF
translation factor	MF
NADH dehydroge	MF
protein tyrosine/	MF
nuclear localizatic	MF

cAMP response e	MF
oxysterol binding	MF
poly(A) binding	MF
drug binding	MF
sulfotransferase a	MF
structural constit	MF
biological_proces	BP
metabolic proces	BP
actin polymerizat	BP
negative regulatic	BP
protein phosphat	MF
hedgehog recept	MF
protein tyrosine p	MF
N-methyltransfer	MF
O-methyltransfer	MF
RNA methyltransf	MF
mRNA methyltrar	MF
tRNA methyltrans	MF
tRNA (guanine-N7	MF
succinate dehydr	MF
adenylate cyclase	MF
COP9 signalosom	CC
glycogen phosph	MF
RNA-dependent A	MF
poly-pyrimidine t	MF
neuropeptide rec	MF
eukaryotic initiati	MF
metalloendopept	MF
RNA guanylyltran	MF
tRNA guanylyltrar	MF
UDP-glycosyltran	MF
phosphatidate ph	MF
ferrous iron bindi	MF
ferric iron binding	MF
ion channel inhibi	MF
bile acid metabol	BP
C21-steroid horm	BP
androgen metabo	BP
estrogen metabo	BP
glucocorticoid me	BP
spermine metabo	BP
spermidine metal	BP
regulation of bloc	BP
cell death	BP
opsonization	BP

peptidase activity	MF
cysteine-type pep	MF
metalloexopeptic	MF
serine-type pepti	MF
metallopeptidase	MF
exopeptidase acti	MF
dipeptidyl-peptid	MF
tripeptidyl-peptic	MF
peptidyl-dipeptid	MF
omega peptidase	MF
1-alkyl-2-acetylgl	CC
oligosaccharyltra	CC
tRNA-specific ade	MF
nucleotidase acti	MF
5'-nucleotidase a	MF
3'-nucleotidase a	MF
3-oxoacid CoA-tra	MF
pyrimidine-specif	MF
Mo-molybdopter	MF
poly(U) RNA bind	MF
JAK pathway sign	MF
secondary active	MF
sulfate transport	BP
calcium, potassiu	MF
gamma-tubulin ri	CC
gamma-tubulin sı	CC
protein methyltra	MF
regulation of G pı	BP
cohesin complex	CC
sulfonylurea rece	MF
inward rectifying	CC
cell population pı	BP
insulin receptor s	BP
protein serine/th	CC
F-actin capping pı	CC
acetylcholine bio	BP
calcium- and caln	MF
spermidine biosy	BP
3'-5'-exodeoxyrib	MF
single-stranded D	MF
intracellular mRN	BP
isoprenoid biosyr	BP
DNA binding, ben	MF
integrin complex	CC
associative learni	BP

structural constit	MF
voltage-gated ani	MF
double-stranded	MF
single-stranded D	MF
double-stranded	MF
7S RNA binding	MF
G2/MI transition	BP
protein prenyltra	MF
protein transmen	MF
cation transmem	MF
methyl-CpG bindi	MF
ionotropic glutam	CC
protein tyrosine/	MF
high voltage-gate	MF
low voltage-gatec	MF
endosome to lysc	BP
histone mRNA me	BP
gamma-butyrobe	MF
determination of	BP
adult feeding beh	BP
adult locomotory	BP
glial cell migratio	BP
MAP kinase kinas	MF
katanin complex	CC
RNA polymerase	MF
germ cell migratio	BP
olfactory learning	BP
asymmetric cell d	BP
regulation of cell	BP
regulation of cell	BP
axon ensheathme	BP
sialyltransferase a	MF
O-acyltransferase	MF
acetylglucosamin	MF
acetylgalactosam	MF
galactosyltransfe	MF
thioredoxin pero	MF
RNA splicing	BP
mechanosensitive	MF
IkappaB kinase ac	MF
IkappaB kinase cc	CC
cholesterol monc	MF
steroid 7-alpha-h	MF
coumarin 7-hydrc	MF
testosterone 16-a	MF

arachidonic acid	MF
arachidonic acid	MF
steroid hydroxyla	MF
oxysterol 7-alpha	MF
sterol 12-alpha-h	MF
sterol 14-demeth	MF
retinoic acid 4-hy	MF
25-hydroxychole	MF
arachidonic acid	MF
arachidonic acid	MF
gonad developm	BP
3'-5' exonuclease	MF
5'-3' exonuclease	MF
CoA-transferase	MF
8-oxo-7,8-dihydr	MF
fucosyltransfera	MF
protein-N-termin	MF
RNA lariat debrar	MF
RNA polymerase	MF
beta-glucosidase	MF
glycoprotein 6-al	MF
2-polyprenyl-6-m	MF
protein kinase C i	MF
calcium-depende	MF
ribonuclease inhi	MF
phosphatidyletha	MF
selenium binding	MF
vitamin E binding	MF
JUN kinase bindin	MF
thyrotropin-relea	MF
inositol-1,4,5-tris	MF
3'(2'),5'-bisphosp	MF
3-hydroxyisobuty	MF
phosphofructokir	MF
CDP-diacylglycer	MF
D-aspartate oxida	MF
GDP-mannose 4,6	MF
N-acetylglucosam	MF
N-acetylglucosam	MF
RNA ligase activit	MF
alanine-glyoxylat	MF
alpha-1,3-manno	MF
alpha-1,6-manno	MF
alpha-N-acetylgal	MF
beta-galactosyl-N	MF

carnitine O-octan	MF
chondroitin 6-sul	MF
dTDP-glucose 4,6	MF
glycerate dehydr	MF
glycogenin glucos	MF
[heparan sulfate]	MF
histone-arginine	MF
isovaleryl-CoA de	MF
palmitoyl-(protei	MF
procollagen-lysin	MF
protein-tyrosine	MF
pyridoxal kinase	MF
queuine tRNA-rib	MF
sarcosine dehydr	MF
sphinganine kina	MF
sulfite oxidase ac	MF
transaminase acti	MF
sulfuric ester hyd	MF
diphosphoinosito	MF
gamma-glutamyl	MF
UDP-galactose:gl	MF
tetracycline trans	MF
translation activa	MF
protoheme IX far	MF
UDP-galactose:be	MF
melatonin recept	MF
benzodiazepine r	MF
monoamine trans	MF
sucrose:proton s	MF
sodium:iodide sy	MF
bile acid:sodium	MF
anion transmemb	MF
sodium:bicarbon	MF
sodium:potassiu	MF
secondary active	MF
organic anion tra	MF
folic acid transme	MF
folate:anion anti	MF
ammonium trans	MF
L-ascorbate:sodiu	MF
acetyl-CoA transr	MF
sodium-depende	MF
phosphatidylchol	MF
phosphatidylinosi	MF
taste receptor ac	MF

G protein-couple	MF
riboflavin kinase a	MF
N-acetyllactosam	MF
oxidized purine n	MF
respiratory chain	BP
proteasome activ	CC
proteasome regu	CC
proteasome regu	CC
visual learning	BP
fibroblast growth	BP
epidermis develo	BP
JUN kinase kinase	MF
proton-exporting	MF
potassium transm	MF
ATPase-coupled x	MF
microtubule-seve	MF
ATP-dependent m	MF
ATP-dependent m	MF
JUN kinase phosp	MF
regulation of syna	BP
male gonad deve	BP
female gonad dev	BP
regulation of smo	BP
regulation of Toll	BP
regulation of Not	BP
photoreceptor ce	BP
anterior/posterio	BP
calcium-depende	MF
phosphorylase kin	MF
attachment of spi	BP
alkylglycerone-ph	MF
ether lipid biosyn	BP
peptidyl-lysine m	BP
pyridoxine biosyn	BP
guanosine metab	BP
epsilon DNA poly	CC
CHRAC	CC
extrinsic apoptoti	BP
granzyme-mediat	BP
intrinsic apoptoti	BP
intrinsic apoptoti	BP
intrinsic apoptoti	BP
activation of cyst	BP
ubiquitin-like mo	MF
carbohydrate tra	BP

hexose transmem	BP
rRNA methyltrans	MF
rRNA (uridine-2'-(	MF
cellular amino aci	BP
lipopolysaccharid	BP
phospholipid bios	BP
pyrimidine-contai	BP
cysteine-type enc	MF
(3R)-hydroxymyri	MF
2',3'-cyclic-nuclec	MF
2,3-dihydro-2,3-d	MF
2,4-dienoyl-CoA r	MF
2-octoprenyl-3-m	MF
3-demethylubiqu	MF
3-hydroxydecano	MF
4-hydroxy-2-oxog	MF
methionine synth	MF
cholate 7-alpha-d	MF
D-lactate dehydrc	MF
D-serine ammoni	MF
DNA-3-methylade	MF
L-allo-threonine a	MF
N-acetylmuramoy	MF
NAD(P)+ transhyc	MF
N-acetylneuramir	MF
NADPH dehydrog	MF
S-adenosylmethic	MF
UDP-N-acetylgluc	MF
UDP-sugar diphos	MF
acetate CoA-trans	MF
N-acylneuraminat	MF
agmatinase activi	MF
alkyl hydroperoxi	MF
arsenate reducta:	MF
bis(5'-nucleosyl)-t	MF
beta-aspartyl-pep	MF
betaine-aldehyde	MF
bis(5'-nucleosyl)-t	MF
cardiolipin syntha	MF
chloramphenicol	MF
choline dehydrog	MF
cob(I)yrinic acid a	MF
crossover junctio	MF
cupric reductase a	MF
dATP pyrophosph	MF

dGTPase activity	MF
endochitinase act	MF
exodeoxyribonuc	MF
exodeoxyribonuc	MF
exoribonuclease I	MF
fructokinase activ	MF
glycerate kinase a	MF
glycerophosphod	MF
glycine C-acetyltr	MF
guanine deamina	MF
guanosine-3',5'-b	MF
guanosine-5'-tripl	MF
holo-[acyl-carrier	MF
potassium:protor	MF
hydroxypyruvate	MF
methylglyoxal syr	MF
inositol monopho	MF
nitrate reductase	MF
nitric oxide dioxy	MF
oxaloacetate deca	MF
peptidoglycan gly	MF
phosphatidylglyce	MF
phospho-N-acety	MF
phosphoglycolate	MF
phospholipase A1	MF
phosphopentomu	MF
protein-glutamat	MF
rRNA (adenine-N6	MF
ribonuclease E ac	MF
selenocysteine ly	MF
site-specific DNA-	MF
DNA-methyltrans	MF
succinate-semialc	MF
tRNA (guanine-N1	MF
tRNA (guanosine-	MF
thymidine phosph	MF
dosage compensa	BP
pentose-phospha	BP
pentose-phospha	BP
electron transfer	MF
catabolic process	BP
biosynthetic proc	BP
aerobic respiratic	BP
fatty acid catabol	BP
cellular amino aci	BP

glutamine family	BP
aspartate family a	BP
serine family ami	BP
serine family ami	BP
serine family ami	BP
aromatic amino a	BP
aromatic amino a	BP
pyruvate family a	BP
branched-chain a	BP
branched-chain a	BP
branched-chain a	BP
methionine biosy	BP
methionine catab	BP
isoleucine biosyn	BP
leucine biosynthe	BP
valine biosynthet	BP
glycoprotein met	BP
glycoprotein bios	BP
lipopolysaccharid	BP
lipoate metabolic	BP
lipoate biosynthe	BP
purine nucleobas	BP
xanthine cataboli	BP
nucleoside metab	BP
nucleoside mono	BP
nucleoside diphos	BP
nucleoside diphos	BP
nucleoside diphos	BP
nucleoside tripho	BP
purine ribonucleo	BP
purine ribonucleo	BP
ribonucleoside m	BP
deoxyribonucleos	BP
deoxyribonucleos	BP
nucleotide biosyn	BP
nucleotide catabo	BP
purine ribonucleo	BP
purine ribonucleo	BP
cyclic nucleotide	BP
ribonucleoside di	BP
deoxyribonucleos	BP
deoxyribonucleos	BP
cyclic nucleotide	BP
purine deoxyribo	BP
pyrimidine ribonu	BP

pyrimidine deoxy	BP
nucleotide-sugar	BP
nucleotide-sugar	BP
thiamine diphosp	BP
riboflavin biosynt	BP
menaquinone bic	BP
cobalamin metab	BP
cobalamin biosyn	BP
isopentenyl diphc	BP
glycolipid biosynt	BP
protein lipoylatio	BP
peptidoglycan cat	BP
10-formyltetrahy	BP
10-formyltetrahy	BP
10-formyltetrahy	BP
ribonucleotide m	BP
deoxyribonucleot	BP
deoxyribonucleot	BP
deoxyribonucleot	BP
response to temp	BP
cellular response	BP
response to pH	BP
nucleoid	CC
GDP-mannose bic	BP
mRNA transcripti	BP
snRNA transcripti	BP
rRNA transcriptio	BP
tRNA transcriptio	BP
protein biotinylat	BP
protein secretion	BP
amine metabolic	BP
amine biosynthet	BP
oligosaccharide m	BP
oligosaccharide b	BP
oligosaccharide c	BP
response to radia	BP
acetyl-CoA carbo:	CC
phenylalanine-tRI	CC
DNA topoisomera	CC
glycerol-3-phospl	CC
mitochondrial oxi	CC
DNA polymerase	CC
succinate-CoA lig	CC
endopeptidase Cl	CC
biotin binding	MF

four-way junctior	MF
rRNA (cytosine-C!	MF
N-acylmannosam	MF
translational atte	BP
phospholipid cata	BP
folic acid-containi	BP
FMN biosynthetic	BP
toxin biosyntheti	BP
toxin metabolic p	BP
pathogenesis	BP
response to heat	BP
response to xeno	BP
response to UV	BP
response to wate	BP
response to light	BP
NAD biosynthetic	BP
glyoxylate catabo	BP
carnitine metabo	BP
methylglyoxal me	BP
cyanate catabolic	BP
glycolate metabo	BP
pyridoxal 5'-phos	BP
pyruvate oxidatio	BP
putrescine metab	BP
putrescine biosyn	BP
putrescine catabo	BP
gamma-aminobu	BP
gamma-aminobu	BP
gamma-aminobu	BP
RNA modification	BP
7-methylguanosir	BP
fertilization	BP
detection of abio	BP
detection of light	BP
detection of visib	BP
detection of chen	BP
detection of bioti	BP
response to symb	BP
response to wour	BP
response to fung	BP
response to nema	BP
response to gravi	BP
cold acclimation	BP
response to herbi	BP
response to toxic	BP

response to blue	BP
response to light	BP
response to high	BP
response to low l	BP
photoperiodism	BP
entrainment of ci	BP
UV protection	BP
response to salt s	BP
anatomical struct	BP
low-affinity phos	MF
induced systemic	BP
ethylene metabo	BP
response to horm	BP
gibberellic acid m	BP
response to carbo	BP
response to sucro	BP
response to gluco	BP
response to fruct	BP
response to salicy	BP
hormone-mediat	BP
carbohydrate me	BP
blue light signalin	BP
regulation of asyr	BP
positive regulatio	BP
post-embryonic d	BP
embryo developn	BP
regulation of mito	BP
axis specification	BP
coumarin metabo	BP
flavonoid metabo	BP
alkaloid metaboli	BP
alkaloid catabolic	BP
unidimensional ce	BP
abscission	BP
mitochondrial en	CC
determination of	BP
embryonic patter	BP
photoreceptor ac	MF
blue light photore	MF
animal organ mor	BP
tissue developme	BP
negative regulatio	BP
negative regulatio	BP
positive regulatio	BP
negative regulatio	BP

cytoplasmic side (	CC
auditory receptor	BP
epidermal cell dif	BP
hormone transpo	BP
sterol 5-alpha rec	MF
fatty acid elongas	MF
fatty acid elongas	CC
basal plasma mer	CC
calcium-depende	MF
proximal/distal a	BP
anterior/posterio	BP
polarity specificat	BP
dorsal/ventral axi	BP
anterior/posterio	BP
dorsal/ventral pa	BP
proximal/distal p	BP
radial pattern for	BP
epidermal cell fat	BP
regulation of sign	BP
positive regulatio	BP
negative regulatio	BP
cytidine deamina	BP
pseudouridine sy	MF
cellular process	BP
cell-cell recogniti	BP
response to extra	BP
cellular water ho	BP
oocyte differentia	BP
glial cell different	BP
cardioblast differ	BP
amylopectin bios	BP
wax biosynthetic	BP
meiotic chromos	BP
response to orga	BP
response to aceta	BP
response to inorg	BP
response to meta	BP
response to iron i	BP
response to iron(	BP
response to iron(	BP
response to mang	BP
response to zinc i	BP
response to alum	BP
response to myco	BP
zygote asymmetr	BP

cellular response	BP
arginine catabolic	BP
phenylacetate cat	BP
proline catabolic	BP
farnesyl diphosph	BP
regulation of prot	BP
response to chlor	BP
specification of ar	BP
response to cesiu	BP
response to X-ray	BP
body morphogen	BP
embryonic body r	BP
FMN binding	MF
positive regulatio	BP
vitamin E biosynt	BP
response to ozon	BP
response to ionizi	BP
maintenance of D	BP
response to UV-B	BP
response to UV-C	BP
response to lithiu	BP
vascular transpor	BP
response to orgar	BP
establishment or	BP
glucose mediatec	BP
endomembrane s	BP
NADH dehydroge	BP
multicellular orga	BP
animal organ sen	BP
SCF complex asse	BP
response to vitam	BP
response to selen	BP
response to silver	BP
detoxification of	BP
response to lead	BP
acireductone diox	MF
regulation of hyd	BP
detoxification of	BP
phosphatidylinosi	MF
membrane invagi	BP
lithium:proton an	MF
regulation of anic	BP
negative regulatic	BP
chromocenter	CC
perinucleolar chr	CC

double-stranded	MF
COP9 signalosom	BP
regulation of G2/	BP
histone monoubi	BP
3,4-dihydroxy-5-p	MF
DNA methylation	BP
methyl-CpNpG bi	MF
fatty acid omega-	BP
carotenoid dioxyg	MF
response to alkali	BP
response to acidi	BP
regulation of cell	BP
negative regulatio	BP
positive regulatio	BP
centriole-centriol	BP
exit from mitosis	BP
negative regulatio	BP
positive regulatio	BP
mesenchymal cel	BP
regulation of mes	BP
nerve growth fact	MF
gene expression	BP
regulation of sign	BP
regulation of gast	BP
response to sulfu	BP
epidermal cell div	BP
regulation of epic	BP
H3 histone acetyl	MF
H4 histone acetyl	MF
cytoplasmic stres	CC
proteasomal prot	BP
proteasomal ubiq	BP
RNA secondary st	BP
negative regulatio	BP
positive regulatio	BP
polyamine home	BP
regulation of acet	BP
negative regulatio	BP
positive regulatio	BP
regulation of pho	BP
positive regulatio	BP
negative regulatio	BP
telomerase inhibi	MF
regulation of calc	BP
negative regulatio	BP

positive regulatio	BP
negative regulatio	BP
negative regulatio	BP
regulation of acti	BP
positive regulatio	BP
regulation of plat	BP
negative regulatio	BP
positive regulatio	BP
regulation of glyc	BP
positive regulatio	BP
regulation of cell	BP
regulation of cellu	BP
regulation of keto	BP
regulation of dou	BP
positive regulatio	BP
positive regulatio	BP
vascular endothe	BP
regulation of vasc	BP
positive regulatio	BP
glutamine secreti	BP
miRNA metabolic	BP
miRNA catabolic p	BP
regulation of lam	BP
positive regulatio	BP
negative regulatio	BP
regulation of end	BP
positive regulatio	BP
negative regulatio	BP
regulation of cyto	BP
positive regulatio	BP
negative regulatio	BP
posttranscription	BP
mRNA localization	BP
regulation of mRN	BP
regulation of carc	BP
positive regulatio	BP
negative regulatio	BP
negative regulatio	BP
programmed cell	BP
positive regulatio	BP
negative regulatio	BP
negative regulatio	BP
epithelial cell mig	BP
regulation of epit	BP
negative regulatio	BP

regulation of mit	BP
positive regulatio	BP
negative regulati	BP
positive regulatio	BP
regulation of plat	BP
positive regulatio	BP
negative regulati	BP
cell communicati	BP
cell communicati	BP
regulation of cell	BP
positive regulatio	BP
positive regulatio	BP
negative regulati	BP
muscle cell apopt	BP
cardiac muscle ce	BP
negative regulati	BP
positive regulatio	BP
negative regulati	BP
ectodermal cell d	BP
epithelial structu	BP
negative regulati	BP
positive regulatio	BP
acetyltransferase	MF
negative regulati	BP
positive regulatio	BP
meiotic DNA doul	BP
ganglioside biosy	BP
globoside biosynt	BP
regulation of coll	BP
negative regulati	BP
regulation of coll	BP
regulation of extr	BP
negative regulati	BP
regulation of epit	BP
positive regulatio	BP
negative regulati	BP
negative regulati	BP
regulation of ferr	BP
regulation of defi	BP
negative regulati	BP
positive regulatio	BP
negative regulati	BP
negative regulati	BP
positive regulatio	BP
serum response e	MF

protein kinase A s	BP
regulation of prot	BP
positive regulatio	BP
regulation of mac	BP
positive regulatio	BP
negative regulatio	BP
positive regulatio	BP
negative regulatio	BP
regulation of nitri	BP
positive regulatio	BP
negative regulatio	BP
regulation of cGM	BP
positive regulatio	BP
negative regulatio	BP
regulation of plas	BP
positive regulatio	BP
negative regulatio	BP
regulation of mac	BP
positive regulatio	BP
negative regulatio	BP
fibroblast migrati	BP
regulation of fibro	BP
positive regulatio	BP
negative regulatio	BP
positive regulatio	BP
negative regulatio	BP
regulation of tran	BP
negative regulatio	BP
regulation of cell	BP
meiotic DNA doul	BP
DNA double-strar	BP
regulation of mRI	BP
regulation of ubic	BP
regulation of mul	BP
positive regulatio	BP
negative regulatio	BP
regulation of tum	BP
negative regulatio	BP
regulation of syna	BP
positive regulatio	BP
regulation of cell-	BP
positive regulatio	BP
negative regulatio	BP
neuropeptide cat	BP
substance P catal	BP

bradykinin catabo	BP
calcitonin catabol	BP
regulation of hor	BP
positive regulatio	BP
regulation of mit	BP
positive regulatio	BP
negative regulati	BP
regulation of cen	BP
positive regulatio	BP
negative regulati	BP
regulation of gluc	BP
positive regulatio	BP
negative regulati	BP
regulation of myc	BP
positive regulatio	BP
negative regulati	BP
telomere mainte	BP
regulation of pro	BP
negative regulati	BP
regulation of ker	BP
positive regulatio	BP
negative regulati	BP
positive regulatio	BP
retina layer form	BP
regulation of chr	BP
regulation of chr	BP
regulation of pro	BP
adenylate cyclase	MF
adenylate cyclase	MF
calcium-depende	MF
calcium-depende	MF
calcium-depende	MF
positive regulatio	BP
positive regulatio	BP
regulation of trig	BP
positive regulatio	BP
negative regulati	BP
regulation of cho	BP
regulation of cho	BP
positive regulatio	BP
lipid localization	BP
lipid transport in	BP
cholesterol stora	BP
regulation of rele	BP
regulation of card	BP

regulation of carc	BP
regulation of lipic	BP
positive regulatio	BP
positive regulatio	BP
negative regulatic	BP
negative regulatic	BP
positive regulatio	BP
negative regulatic	BP
negative regulatic	BP
negative regulatic	BP
positive regulatio	BP
regulation of pho	BP
negative regulatic	BP
positive regulatio	BP
negative regulatic	BP
negative regulatic	BP
regulation of gluc	BP
positive regulatio	BP
positive regulatio	BP
negative regulatic	BP
negative regulatic	BP
positive regulatio	BP
negative regulatic	BP
regulation of pho	BP
positive regulatio	BP
negative regulatic	BP
positive regulatio	BP
positive regulatio	BP
negative regulatic	BP
regulation of necı	BP
positive regulatio	BP
regulation of cell	BP
positive regulatio	BP
negative regulatic	BP
CoA pyrophospha	MF
negative regulatic	BP
negative regulatic	BP
positive regulatio	BP
positive regulatio	BP
positive regulatio	BP
negative regulatic	BP
negative regulatic	BP
negative regulatic	BP
magnesium ion h	BP
cellular magnesiu	BP

regulation of mit	BP
regulation of pho	BP
regulation of mic	BP
transport along m	BP
positive regulatio	BP
negative regulati	BP
regulation of neu	BP
positive regulatio	BP
negative regulati	BP
positive regulatio	BP
positive regulatio	BP
regulation of low	BP
negative regulati	BP
regulation of SM/	BP
negative regulati	BP
ubiquitin recyclin	BP
free ubiquitin cha	BP
anaphase-promo	MF
regulation of tran	BP
regulation of eIF2	BP
programmed cell	BP
induction of prog	BP
endomembrane s	CC
ER to Golgi transp	CC
trans-Golgi netwo	CC
astrocyte develop	BP
oligodendrocyte	BP
microglia differen	BP
microglia develop	BP
positive regulatio	BP
glial cell prolifera	BP
Schwann cell prol	BP
peripheral nervou	BP
regulation of glio	BP
positive regulatio	BP
neuroblast differe	BP
notochord forma	BP
neural crest form	BP
mesenchymal cel	BP
neural crest cell c	BP
neural crest cell c	BP
neural crest cell f	BP
neural crest cell f	BP
Schwann cell diffe	BP
regulation of Sch	BP

positive regulatio	BP
regulation of neu	BP
positive regulatio	BP
negative regulatio	BP
Schwann cell dev	BP
establishment of	BP
glutamate secreti	BP
regulation of glut	BP
positive regulatio	BP
negative regulatio	BP
gamma-aminobu	BP
regulation of gam	BP
negative regulatio	BP
positive regulatio	BP
regulation of acet	BP
positive regulatio	BP
regulation of dop	BP
regulation of nore	BP
negative regulatio	BP
positive regulatio	BP
phosphatidylinosi	BP
regulation of pho	BP
negative regulatio	BP
positive regulatio	BP
response to purin	BP
response to amin	BP
junctional sarcopl	CC
intercalated disc	CC
C zone	CC
striated muscle ti	BP
branchiomeric sk	BP
positive regulatio	BP
skeletal muscle sa	BP
regulation of skel	BP
spectrin-associate	CC
skeletal muscle a	BP
regulation of skel	BP
negative regulatio	BP
positive regulatio	BP
negative regulatio	BP
negative regulatio	BP
longitudinal sarco	CC
terminal cisterna	CC
terminal cisterna	CC
smooth muscle a	BP

smooth muscle h	BP
regulation of som	BP
release of seques	BP
regulation of skel	BP
positive regulatio	BP
skeletal muscle sa	BP
regulation of skel	BP
tonic smooth mus	BP
response to activi	BP
artery smooth mu	BP
vein smooth mus	BP
intestine smooth	BP
vascular smooth	BP
urinary bladder sı	BP
skeletal muscle sa	BP
myoblast differer	BP
myoblast migratic	BP
skeletal muscle sa	BP
regulation of skel	BP
growth factor dep	BP
esophagus smoot	BP
response to musc	BP
response to inact	BP
regulation of skel	BP
positive regulatio	BP
regulation of skel	BP
skeletal myofibril	BP
response to musc	BP
response to musc	BP
response to injury	BP
response to elect	BP
transition betwee	BP
transition betwee	BP
muscle atrophy	BP
striated muscle a	BP
response to dene	BP
smooth muscle h	BP
muscle hypertrop	BP
cardiac muscle hy	BP
satellite cell activ	BP
myotube differen	BP
myotube cell dev	BP
myoblast fusion i	BP
myotube differen	BP
smooth muscle ce	BP

regulation of smc	BP
positive regulatio	BP
negative regulatic	BP
regulation of lung	BP
heme-copper terı	MF
heparan sulfate p	BP
heparan sulfate p	BP
heparan sulfate p	BP
[heparan sulfate]-	MF
galactosylgalacto:	MF
heparan-alpha-glı	MF
glucuronosyltrans	MF
coreceptor activit	MF
Cajal body	CC
protein disulfide (	MF
disulfide oxidorec	MF
peptide disulfide	MF
glutathione disulf	MF
NADPH-adrenodc	MF
secretin receptor	MF
thrombin-activate	MF
amidinotransfera	MF
glycine amidinotr	MF
DNA integration	BP
ion transmembra	MF
proton transmem	MF
potassium ion tra	MF
sodium ion transı	MF
calcium ion transı	MF
cadmium ion trar	MF
cobalt ion transm	MF
ferrous iron trans	MF
lead ion transmeı	MF
magnesium ion tı	MF
molybdate ion trა	MF
nickel cation tran	MF
vanadium ion traı	MF
organic cation tra	MF
arsenite transmeı	MF
bicarbonate trans	MF
chloride transmeı	MF
iodide transmeml	MF
nitrate transmem	MF
phosphate ion trა	MF
sulfate transmem	MF

thiosulfate transr	MF
bile acid transme	MF
canalicular bile ac	MF
bilirubin transme	MF
lactate transmem	MF
mevalonate trans	MF
oxaloacetate tran	MF
prostaglandin tra	MF
sialic acid transm	MF
citrate transmem	MF
alpha-ketoglutara	MF
malate transmem	MF
succinate transm	MF
tricarboxylic acid	MF
urate transmemb	MF
carbohydrate tra	MF
pentose transme	MF
fucose transmem	MF
alpha-glucoside t	MF
glucose-6-phosph	MF
glucuronoside tra	MF
pyrimidine nucle	MF
polyol transmem	MF
glycerol transmer	MF
amino acid transr	MF
acidic amino acid	MF
aromatic amino a	MF
basic amino acid	MF
neutral amino aci	MF
L-amino acid tran	MF
L-alanine transme	MF
arginine transme	MF
L-asparagine tran	MF
L-aspartate trans	MF
L-cystine transme	MF
gamma-aminobu	MF
L-glutamine trans	MF
glycine transmem	MF
L-isoleucine trans	MF
L-lysine transmen	MF
L-leucine transme	MF
L-methionine tra	MF
L-proline transme	MF
L-serine transmer	MF
L-threonine trans	MF

L-tryptophan trar	MF
amino-acid betair	MF
urea transmembr	MF
nucleobase transı	MF
adenine transmeı	MF
purine nucleoside	MF
cytidine transmer	MF
uridine transmem	MF
pyrimidine nuclec	MF
purine nucleotide	MF
ADP transmembr	MF
pyrimidine nuclec	MF
choline transmen	MF
biotin transmemb	MF
carnitine transme	MF
acyl carnitine trar	MF
coenzyme A trans	MF
L-ascorbic acid tra	MF
FAD transmembra	MF
heme transporter	MF
pantothenate tra	MF
thiamine transme	MF
fatty acid transm	MF
aminophospholip	MF
sterol transporter	MF
water channel act	MF
proton channel a	MF
glycerol channel a	MF
urea channel acti	MF
channel activity	MF
calcium-activated	MF
outward rectifier	MF
ATP-activated inv	MF
stretch-activated,	MF
ligand-gated ion c	MF
kainate selective	MF
store-operated ca	MF
ligand-gated sodi	MF
porin activity	MF
uniporter activity	MF
symporter activit	MF
solute:proton syn	MF
anion:cation sym	MF
antiporter activity	MF
solute:proton ant	MF

anion:anion antip	MF
phosphate:protoı	MF
sodium:inorganic	MF
cystine:glutamate	MF
peptide:proton sy	MF
sodium-independ	MF
thyroid hormone	MF
methotrexate tra	MF
low-affinity sodiu	MF
high-affinity sodiu	MF
oxoglutarate:mal	MF
calcium:cation an	MF
calcium:proton aı	MF
glycine:sodium sy	MF
cation:chloride sy	MF
sodium:chloride s	MF
potassium:chloric	MF
sodium:sulfate sy	MF
sodium:proton ar	MF
potassium:protor	MF
pyrimidine- and a	MF
purine-specific nu	MF
manganese trans	MF
ATPase-coupled p	MF
ATPase-coupled g	MF
ATPase-coupled b	MF
ATPase-coupled p	MF
ATPase-coupled h	MF
ATPase-coupled p	MF
P-P-bond-hydroly	MF
oxidoreduction-d	MF
potassium chann	MF
acetylcholine rec	MF
G-protein activat	MF
cation:cation anti	MF
gamma-aminobu	MF
formate transmeı	MF
glutamate:sodiun	MF
propionate transı	MF
efflux transmemb	MF
ATPase-coupled f	MF
DNA translocase a	MF
tubulin binding	MF
short-chain fatty	MF
toxic substance b	MF

fatty acid ligase a	MF
peptidoglycan tra	MF
quaternary ammo	MF
branched-chain a	MF
formate efflux tra	MF
carbon dioxide tr	BP
oxygen transport	BP
monovalent inorg	BP
nickel cation tran	BP
vanadium ion tra	BP
copper ion impor	BP
protein maturatio	BP
molybdate ion tra	BP
lead ion transpor	BP
magnesium ion tr	BP
mercury ion trans	BP
organic cation tra	BP
ammonium trans	BP
quaternary ammo	BP
inorganic anion tr	BP
arsenite transpor	BP
bicarbonate trans	BP
iodide transport	BP
nitrate transport	BP
nitrite transport	BP
thiosulfate transp	BP
organic anion tra	BP
hexose phosphat	BP
monocarboxylic a	BP
bile acid and bile	BP
canalicular bile ac	BP
bilirubin transpor	BP
formate transpor	BP
lactate transport	BP
mevalonate trans	BP
oxaloacetate tran	BP
propanoate trans	BP
prostaglandin tra	BP
taurine transport	BP
sialic acid transpo	BP
alpha-ketoglutara	BP
succinate transpo	BP
citrate transport	BP
urate transport	BP
pentose transme	BP

fructose transme	BP
fucose transmem	BP
galactose transm	BP
glucose-6-phosph	BP
sucrose transport	BP
polysaccharide tr	BP
glucuronoside tra	BP
CMP-N-acetylneu	BP
UDP-glucose tran	BP
UDP-glucuronic a	BP
UDP-N-acetylgala	BP
UDP-xylose trans	BP
polyol transport	BP
glycerol transport	BP
mannitol transpo	BP
myo-inositol tran	BP
aromatic amino a	BP
basic amino acid	BP
branched-chain a	BP
neutral amino aci	BP
S-adenosyl-L-met	BP
L-amino acid tran	BP
L-alanine transpo	BP
arginine transpor	BP
aspartate transm	BP
L-cystine transpo	BP
gamma-aminobu	BP
L-glutamate trans	BP
glycine transport	BP
histidine transpor	BP
lysine transport	BP
leucine transport	BP
methionine trans	BP
ornithine transpo	BP
phenylalanine tra	BP
proline transport	BP
L-serine transport	BP
threonine transpo	BP
tryptophan trans	BP
tyrosine transpor	BP
valine transport	BP
peptide transport	BP
peptidoglycan tra	BP
amine transport	BP
amino-acid betair	BP

urea transport	BP
aminergic neurot	BP
monoamine trans	BP
organic hydroxy c	BP
nucleobase trans	BP
adenine transpor	BP
guanine transpor	BP
pyrimidine nuclec	BP
nucleoside transp	BP
purine nucleoside	BP
cytidine transport	BP
uridine transport	BP
pyrimidine nuclec	BP
purine nucleotide	BP
ADP transport	BP
ATP transport	BP
purine ribonuclec	BP
choline transport	BP
dopamine transp	BP
norepinephrine ti	BP
acetyl-CoA transp	BP
biotin transport	BP
carnitine transpo	BP
creatine transme	BP
L-ascorbic acid tr	BP
folic acid transpo	BP
heme transport	BP
pantothenate tra	BP
thiamine transpo	BP
cobalamin transp	BP
siderophore trans	BP
tetracycline trans	BP
fatty acid transpo	BP
long-chain fatty a	BP
long-chain fatty a	BP
long-chain fatty a	BP
short-chain fatty	BP
short-chain fatty	BP
phospholipid trar	BP
fatty-acyl-CoA tra	BP
aminophospholip	BP
peroxisomal mem	BP
lipopolysaccharid	BP
mannosidase acti	MF
glucosidase activi	MF

trehalase activity	MF
hexosaminidase a	MF
nucleobase-conta	BP
large ribosomal s	CC
small ribosomal s	CC
coenzyme A meta	BP
coenzyme A biosy	BP
coenzyme A catal	BP
pantothenate me	BP
formate metaboli	BP
nucleobase-conta	BP
diadenosine poly	BP
diadenosine triph	BP
diadenosine tetra	BP
energy derivation	BP
ATP synthesis cou	BP
electron transpor	BP
chlorophyll biosy	BP
phospholipase ac	MF
phospholipase A2	MF
dystrophin-associ	CC
dystroglycan com	CC
sarcoglycan comp	CC
syntrophin compl	CC
morphogen activi	MF
cyclosporin A bin	MF
peptidoglycan im	MF
CDP-diacylglycerc	BP
rhabdomere	CC
tRNA import into	BP
maleylacetoaceta	MF
zeta DNA polyme	CC
cellular response	BP
lipid catabolic prc	BP
cellular compone	BP
detection of bact	BP
detection of fung	BP
detection of temp	BP
vesicle organizati	BP
carbohydrate bio	BP
carbohydrate cat	BP
Wnt signaling pat	BP
rhodopsin mediat	BP
RNA metabolic pr	BP
mRNA metabolic	BP

rRNA metabolic p	BP
snRNA metabolic	BP
snoRNA metaboli	BP
rRNA catabolic pr	BP
snRNA catabolic p	BP
snoRNA catabolic	BP
tRNA catabolic pr	BP
synaptic vesicle e	BP
synaptic vesicle t	BP
synaptic vesicle d	BP
synaptic vesicle p	BP
polyprenol biosyn	BP
polyprenol catabo	BP
monoterpenoid m	BP
diterpenoid meta	BP
triterpenoid biosy	BP
carotenoid metab	BP
carotene metabo	BP
carotene cataboli	BP
xanthophyll meta	BP
sterol metabolic p	BP
glycoside catabol	BP
translation releas	MF
translation releas	MF
nickel cation bind	MF
urocanate hydrat	MF
pyrimidine-nucleo	MF
formyltetrahydro	MF
linoleate 13S-lipo	MF
glial cell-derived n	MF
interleukin-15 rec	MF
NAD(P)H oxidase	MF
superoxide-gener	MF
snRNA processing	BP
synaptic vesicle b	BP
synaptic vesicle m	BP
synaptic vesicle to	BP
synaptic vesicle u	BP
vesicle-mediated	BP
endosomal transp	BP
axon choice point	BP
axon midline choi	BP
regulation of stria	BP
muscle attachme	BP
catechol O-methy	MF

AMP binding	MF
antioxidant activi	MF
kynurenine-oxogl	MF
linoleoyl-CoA des	MF
procollagen-proli	CC
iron-sulfur cluste	BP
steroid dehydrog	MF
sphingomyelin ph	MF
beta-N-acetylgluc	MF
telomere capping	BP
inclusion body	CC
aggresome	CC
macroautophagy	BP
positive regulatio	BP
autophagosome r	BP
regulation of mac	BP
negative regulatio	BP
regulation of auto	BP
RNA interference	BP
channel regulator	MF
channel inhibitor	MF
N-sulfoglucosami	MF
RNA polymerase	MF
preassembly of G	BP
attachment of GP	BP
N-glycan processi	BP
selenocysteine m	BP
selenocysteine bi	BP
selenocysteine ca	BP
protein N-acetylg	MF
glycoprotein-N-ac	MF
gap junction asse	BP
O-glycan processi	BP
O-glycan processi	BP
O-glycan processi	BP
prefoldin comple	CC
protein-arginine N	MF
[myelin basic pro	MF
lysine N-methyltr	MF
protein-lysine N-r	MF
eukaryotic transla	CC
eukaryotic 43S pr	CC
small conductanc	MF
glycerone-phosph	MF
palmitoyl-CoA hy	MF

myristoyl-[acyl-ca	MF
palmitoyl-[acyl-ca	MF
acyl-[acyl-carrier-	MF
lipase activity	MF
tRNA (uracil) met	MF
1-phosphatidylin	MF
phosphatidylinosi	MF
1-phosphatidylin	MF
1-phosphatidylin	MF
dephosphorylatic	BP
inositol bisphosp	MF
phosphatidylinosi	MF
phosphatidylinosi	MF
endoplasmic retic	BP
female meiosis cl	BP
neuron remodelin	BP
basolateral plasm	CC
apical plasma me	CC
apicolateral plasn	CC
lateral plasma me	CC
morphogenesis o	BP
establishment or	BP
calcium-indepenc	BP
calcium-depende	BP
calcium-depende	BP
catenin complex	CC
meiotic chromos	BP
dendrite develop	BP
activin receptor a	MF
activin receptor a	MF
nuclear matrix	CC
palmitoyl-CoA ox	MF
pristanoyl-CoA ox	MF
dimethylarginina	MF
15-hydroxyprosta	MF
CoA-ligase activit	MF
acetyltransferase	MF
palmitoyltransfer	MF
N-acyltransferase	MF
acylglycerol O-ac	MF
octanoyltransfera	MF
S-acetyltransfera	MF
S-malonyltransfe	MF
CoA carboxylase	MF
mRNA (2'-O-meth	MF

tRNA (guanine) m	MF
tRNA (cytosine) n	MF
tRNA (cytosine-5-	MF
tRNA (adenine-N1	MF
tRNA (adenine-N6	MF
tRNA-uridine ami	MF
rRNA (adenine) m	MF
rRNA (guanine) m	MF
tRNA cytidylyltrar	MF
posttranscription	BP
RISC complex	CC
somatic cell DNA	BP
somatic diversific	BP
somatic hypermu	BP
somatic recombir	BP
C-acetyltransfera:	MF
gene silencing	BP
myosin complex	CC
myosin II comple:	CC
unconventional m	CC
pyrophosphatase	MF
proton-transporti	CC
vacuolar proton-t	CC
regulation of emb	BP
negative regulatic	BP
negative regulatic	BP
cytosolic transpo	BP
protein processin	BP
peptide hormone	BP
farnesol catabolic	BP
C-C chemokine re	MF
C-X-C chemokine	MF
C-X3-C chemokin	MF
substance P recep	MF
substance K recep	MF
protein-hormone	MF
prostacyclin recep	MF
pheromone recep	MF
peptidase activat	MF
peptidase activat	MF
mitochondrial fat	CC
long-chain-enoyl-	MF
long-chain-3-hydr	MF
core-binding fact	CC
SWI/SNF complex	CC

interleukin-12 rec	MF
gastric inhibitory	MF
growth hormone-	MF
latrotoxin recepto	MF
sarcoplasm	CC
metallochaperone	MF
copper chaperone	MF
superoxide dismu	MF
protein kinase 5 c	CC
cyclin-dependent	MF
intein-mediated p	BP
protein autoproc	BP
base conversion c	BP
cytidine to uridin	BP
mRNA modificatio	BP
peroxisome mem	BP
protein import in	BP
peroxisome fissio	BP
protein import in	BP
protein import in	BP
covalent chromat	BP
histone modificat	BP
histone phosphor	BP
histone acetylatic	BP
histone ubiquitina	BP
histone deacetyla	BP
histone dephosph	BP
histone demethyl	BP
histone deubiquit	BP
Sin3 complex	CC
NuRD complex	CC
nucleosome posit	BP
RSC-type comple	CC
NURF complex	CC
ACF complex	CC
RNA polymerase	CC
mediator comple	CC
Cdc73/Paf1 comp	CC
glycine binding	MF
glutamate binding	MF
thienylcyclohexyl	MF
amino acid bindir	MF
protein arginylati	BP
flotillin complex	CC
Rac protein signa	BP

CCAAT-binding fa	CC
glutaminyl-peptic	MF
nuclear body	CC
PML body	CC
growth hormone-	MF
oxidoreductase a	MF
malate dehydrog	MF
oxidoreductase a	MF
hydroxypyruvate	MF
oxidoreductase a	MF
oxidoreductase a	MF
oxidoreductase a	MF
oxidoreductase a	MF
oxidoreductase a	MF
oxidoreductase a	MF
oxidoreductase a	MF
oxidoreductase a	MF
oxidoreductase a	MF
oxidoreductase a	MF
oxidoreductase a	MF
oxidoreductase a	MF
oxidoreductase a	MF
oxidoreductase a	MF
oxidoreductase a	MF
oxidoreductase a	MF
oxidoreductase a	MF
oxidoreductase a	MF
2-oxoglutarate-de	MF
oxidoreductase a	MF
flavonoid 3'-mon	MF
oxidoreductase a	MF
oxidoreductase a	MF
oxidoreductase a	MF
oxidoreductase a	MF
oxidoreductase a	MF
hydroxymethyl-, f	MF
carboxyl- or carba	MF
transferase activi	MF
transferase activi	MF
transferase activi	MF
transferase activi	MF
transferase activi	MF
transferase activi	MF
spermine synthas	MF
transferase activi	MF

phosphotransfera	MF
phosphotransfera	MF
phosphotransfera	MF
sulfurtransferase	MF
3-mercaptopyruv	MF
transferase activi	MF
hydrolase activity	MF
thiolester hydrola	MF
phosphatase acti	MF
triphosphoric mo	MF
hydrolase activity	MF
hydrolase activity	MF
dipeptidase activi	MF
cysteine-type car	MF
hydrolase activity	MF
hydrolase activity	MF
hydrolase activity	MF
hydrolase activity	MF
hydrolase activity	MF
hydrolase activity	MF
hydrolase activity	MF
carbon-carbon lya	MF
carboxy-lyase act	MF
oxo-acid-lyase ac	MF
hydro-lyase activi	MF
ammonia-lyase a	MF
strictosidine synt	MF
carbon-sulfur lya	MF
phosphorus-oxyg	MF
isomerase activit	MF
racemase and epi	MF
racemase and epi	MF
cis-trans isomera	MF
intramolecular ox	MF
intramolecular ox	MF
intramolecular tra	MF
intramolecular tra	MF
ligase activity	MF
acid-thiol ligase a	MF
ligase activity, for	MF
acid-amino acid li	MF
carbon-nitrogen l	MF
ligase activity, for	MF
endodeoxyribonu	MF
site-specific endo	MF

endoribonuclease	MF
endoribonuclease	MF
G protein-couple	MF
GABA receptor ac	MF
retinal binding	MF
pyroglutamyl-pep	MF
nuclear receptor	MF
protein sumoylat	BP
protein desumoyl	BP
extracellularly gly	MF
extracellularly gly	MF
glycine-gated chl	CC
galactoside bindi	MF
short-branched-c	MF
kinesin I complex	CC
kinesin II comple	CC
natriuretic peptid	MF
insulin-like growt	CC
alpha-2 macroglo	MF
flavin-linked sulfh	MF
thiol oxidase acti	MF
poly(A)+ mRNA e	BP
lipoate synthase	MF
alpha-sialidase ac	MF
cell wall macrom	BP
antibiotic metab	BP
activin-activated	MF
cytochrome com	BP
3'-tyrosyl-DNA ph	MF
regulation of tran	BP
MAP kinase tyros	MF
myosin phosphat	MF
myosin phosphat	MF
myosin binding	MF
myosin I binding	MF
TBP-class protein	MF
protein import	BP
N-acylsphingosin	MF
galactosylgalacto	MF
peptide hormone	MF
D-erythro-sphing	MF
transcription repr	CC
negative cofactor	CC
negative regulatio	BP
structural constit	MF

6-phosphoglucon	MF
serine C-palmitoy	CC
3-galactosyl-N-ac	MF
S-methyl-5-thioa	MF
fatty acid amide h	MF
single-strand sele	MF
snRNA binding	MF
U6 snRNA binding	MF
intracellular cycli	CC
syntaxin-1 bindin	MF
purine nucleotide	MF
oxidative phosph	MF
sodium channel r	MF
chloride channel	MF
4-galactosyl-N-ac	MF
response to insec	BP
mitochondrial pro	CC
glycolipid transfe	MF
meprin A comple	CC
heparan sulfate 6	MF
acetylserotonin C	MF
sulfonylurea rece	MF
very-long-chain-a	MF
aminoacyl-tRNA s	CC
5'-flap endonucle	MF
glutamate-cystei	CC
nucleoside-dipho	MF
nucleoside-tripho	MF
dihydropyrimidin	MF
single-stranded D	MF
lipoyltransferase	MF
Golgi transport co	CC
plasma membran	BP
protein N-acetylg	CC
SH3 domain bindi	MF
deoxycytidyl tran	MF
nucleologenesis	BP
phospholipid scra	MF
triglyceride bindi	MF
poly(C) RNA bindi	MF
fibroblast growth	MF
NAD-dependent h	MF
insecticide metab	BP
drug metabolic p	BP
stem cell division	BP

NMDA selective g	CC
Wnt-protein bind	MF
negative regulatic	BP
tRNA dihydrourid	MF
DEAD/H-box RNA	MF
sodium:dicarboxy	MF
semaphorin recep	MF
calcium-ion regul	BP
regulation of exo	BP
regulation of calc	BP
pantetheine hydr	MF
inositol-1,3,4-tris	MF
aryl hydrocarbon	MF
vinculin binding	MF
5-oxoprolinase (A	MF
serine hydrolase a	MF
glycine N-methylt	MF
phosphatidylinosi	MF
glucosidase II con	CC
diphthine-ammor	MF
peptidyl-diphthar	BP
peptidyl-lysine hy	BP
peptidyl-pyroglut	BP
peptidyl-glutamic	BP
aspartate N-acety	MF
N-terminal peptic	BP
N-terminal peptic	BP
N-terminal peptic	BP
N-terminal peptic	BP
N-terminal peptic	BP
N-terminal peptic	BP
N-terminal peptic	BP
N-terminal peptic	BP
N-terminal peptic	BP
N-terminal peptic	BP
peptidyl-histidine	BP
peptidyl-lysine m	BP
peptidyl-lysine tri	BP
calmodulin-lysine	MF
peptidyl-lysine m	BP
peptidyl-lysine di	BP
protein amidatior	BP
peptidyl-lysine ox	BP
cytochrome c-hei	BP
protein-histidine	MF

N-terminal peptic	BP
protein polyglycy	BP
protein polygluta	BP
protein citrullinat	BP
peptidyl-serine pl	BP
peptidyl-histidine	BP
peptidyl-threonin	BP
peptidyl-tyrosine	BP
threonine racema	MF
protein adenylyla	BP
peptidyl-cysteine	BP
peptidyl-arginine	BP
peptidyl-cysteine	BP
protein hydroxyla	BP
protein-DNA cova	BP
keratan sulfate bi	BP
peptide cross-link	BP
isopeptide cross-l	BP
protein oxidation	BP
peptidyl-pyrrome	BP
C-terminal protei	BP
peptidyl-cysteine	BP
peptidyl-amino ac	BP
peptidyl-glutamic	BP
peptidyl-lysine m	BP
peptidyl-methion	BP
peptidyl-proline r	BP
peptidyl-arginine	BP
peptidyl-L-cysteir	BP
protein O-linked g	BP
protein O-linked g	BP
biotin-protein liga	MF
protein-pyridoxal	BP
isopeptide cross-l	BP
protein deaminat	BP
protein N-linked g	BP
iron incorporatio	BP
protein-FAD linka	BP
protein-chromop	BP
peptidyl-serine Al	BP
molybdenum inco	BP
protein prenylatic	BP
protein farnesylat	BP
protein geranylge	BP
protein palmitoyl	BP

protein esterifica	BP
protein-pyridoxal	BP
peptidyl-glutamir	BP
internal peptidyl-	BP
peptidyl-lysine ac	BP
peptidyl-lysine hy	BP
peptidyl-proline h	BP
peptidyl-proline h	BP
protein C-linked g	BP
protein C-termina	MF
peptidyl-glutamic	BP
translation releas	CC
alcohol dehydrog	MF
formaldehyde de	MF
benzaldehyde deh	MF
malonate-semialc	MF
benzaldehyde deh	MF
methanethiol oxi	MF
4-nitrophenol 2-n	MF
phenanthrene 9,1	MF
(S)-limonene 6-m	MF
(S)-limonene 7-m	MF
alkane 1-monoox	MF
3,4-dihydrocoum	MF
S-formylglutathio	MF
acetylpyruvate hy	MF
3-hydroxyacyl-Co	MF
benzoate-CoA liga	MF
arsonoacetate me	BP
benzoate metabo	BP
biphenyl metabol	BP
carbon tetrachlor	BP
dibenzo-p-dioxin	BP
benzene metabol	BP
nitrobenzene me	BP
naphthalene met	BP
phenol-containin	BP
4-nitrophenol me	BP
phthalate metabo	BP
propylene metab	BP
thiocyanate meta	BP
trichloroethylene	BP
guanyl nucleotide	MF
GMP binding	MF
SCF ubiquitin liga	CC

molybdopterin sy	CC
release from viral	BP
modulation by vir	BP
suppression by vi	BP
modulation by vir	BP
modulation by vir	BP
viral life cycle	BP
uncoating of virus	BP
virion attachment	BP
fusion of virus me	BP
receptor-mediate	BP
virion assembly	BP
viral RNA genome	BP
virus maturation	BP
viral release from	BP
viral genome repl	BP
viral translation	BP
viral protein proc	BP
viral transcription	BP
early viral transcr	BP
late viral transcrip	BP
transformation of	BP
male germ-line se	BP
male somatic sex	BP
pyrimidine nucleo	MF
DNA N-glycosylas	MF
myristoyltransfer	MF
benzaldehyde del	MF
phenanthrene-9,1	MF
deoxyhypusine m	MF
deoxynucleoside	MF
ADP-sugar diphos	MF
aminobutyraldeh	MF
D-ribulokinase ac	MF
protein-disulfide	MF
mannokinase acti	MF
trans-2-enoyl-CoA	MF
3-hydroxyacyl-[ac	MF
NADP phosphatas	MF
snRNA-activating	CC
carbohydrate kina	MF
amino acid kinase	MF
carbohydrate pho	MF
nucleobase-conta	MF
nucleoside kinase	MF

kinase regulator a	MF
phosphatase regu	MF
kinase activator a	MF
kinase inhibitor a	MF
phosphatase acti	MF
phosphatase inhil	MF
deacetylase activ	MF
intermediate filar	MF
regulation of fatt	BP
regulation of ster	BP
regulation of met	BP
transmission of n	BP
neuronal action p	BP
neuronal action p	BP
regulation of vasc	BP
proprioception	BP
sensory perceptic	BP
response to pher	BP
centromeric DNA	MF
deaminase activit	MF
citrulline biosynth	BP
methylglyoxal bic	BP
methylglyoxal cat	BP
lactate biosynthe	BP
lactate biosynthe	BP
carnitine metabo	BP
glucose 1-phosph	BP
N-acetylneuramir	BP
glycine biosynthe	BP
glycine biosynthe	BP
UDP-N-acetylgala	BP
L-methionine salv	BP
glycine betaine bi	BP
isopentenyl diphc	BP
siderophore bios	BP
D-ribose catabolic	BP
inositol catabolic	BP
hexose metabolic	BP
hexose biosynthe	BP
pentose metaboli	BP
pentose biosynth	BP
pentose catabolic	BP
dibenzo-p-dioxin	BP
cysteine biosynth	BP
cysteine biosynth	BP

transsulfuration	BP
dolichol metaboli	BP
pyridine nucleoti	BP
fatty acid elongat	BP
arachidonic acid r	BP
leukotriene biosy	BP
cyclooxygenase p	BP
lipoxygenase patl	BP
epoxygenase P45	BP
glycolipid catabol	BP
galactose catabol	BP
glucuronoside cat	BP
fatty acid oxidatic	BP
galactitol metabo	BP
dolichol biosynth	BP
acetate biosynthe	BP
sulfur oxidation	BP
acetyl-CoA biosyr	BP
removal of super	BP
triglyceride biosy	BP
triglyceride catab	BP
aromatic compou	BP
aromatic compou	BP
tryptophan catab	BP
tryptophan catab	BP
tryptophan catab	BP
L-cysteine catabo	BP
L-cysteine catabo	BP
L-cysteine catabo	BP
glycine decarboxy	BP
4-hydroxyproline	BP
L-lysine catabolic	BP
D-amino acid cata	BP
beta-alanine meta	BP
beta-alanine bios	BP
L-methionine salv	BP
S-adenosylhomoc	BP
peptidyl-proline h	BP
lactate oxidation	BP
L-threonine catak	BP
D-gluconate meta	BP
taurine metabolic	BP
oxalate transmen	MF
oxalate transport	BP
toxin transmemb	MF

protein metabolic	BP
propionate biosyn	BP
propionate catab	BP
arginine catabolic	BP
arginine catabolic	BP
glutamate catabo	BP
glutamate catabo	BP
histidine catabolic	BP
histidine catabolic	BP
glycerol catabolic	BP
butyrate metabol	BP
2-oxobutyrate ca	BP
short-chain fatty	BP
urea metabolic pr	BP
organophosphate	BP
glucuronate catab	BP
aerobic electron t	BP
GDP-mannose me	BP
NAD metabolic pr	BP
ammonia assimila	BP
NAD catabolic pro	BP
glyceraldehyde-3-	BP
ribose phosphate	BP
choline metabolic	BP
protein-cysteine S	MF
protein-cysteine S	MF
calcium-mediated	BP
B cell mediated ir	BP
cellular homeosta	BP
antimicrobial hun	BP
antibacterial hum	BP
antifungal humor	BP
pentacyclic triter	BP
regulation of isop	BP
carboxylic acid m	BP
immunoglobulin r	MF
IgA receptor activ	MF
IgE receptor activ	MF
low-affinity IgG re	MF
proteasome core	CC
proteasome core	CC
Atg8 ligase activit	MF
Atg12 transferase	MF
Atg12 activating e	MF
Atg8 activating en	MF

FAT10 activating	MF
NEDD8 activating	MF
ISG15 activating	MF
ubiquitin-like pro	MF
NEDD8-specific p	MF
ubiquitin-like pro	MF
NEDD8 transferas	MF
SUMO transferas	MF
procollagen-proli	MF
tubulin N-acetyltr	MF
peptide cross-link	BP
quinolinate biosy	BP
bromide peroxida	MF
aspartoacylase ac	MF
spermidine bindir	MF
putrescine bindin	MF
cocaine binding	MF
immunoglobulin	CC
B cell receptor co	CC
oxygen binding	MF
oxygen sensor ac	MF
stem cell populat	BP
aspartic-type end	MF
ATPase-coupled	MF
phospholipase A2	MF
growth factor bin	MF
retinol binding	MF
vitamin binding	MF
rRNA binding	MF
L-ascorbic acid m	BP
L-ascorbic acid bi	BP
calcium channel i	MF
pyrimidine nucle	BP
cytosine metabol	BP
thymine metabol	BP
uracil metabolic	BP
IgA binding	MF
IgE binding	MF
IgG binding	MF
immunoglobulin	MF
organelle inner m	CC
outer membrane	CC
chloride channel	MF
potassium chann	MF
sodium channel i	MF

lysine biosyntheti	BP
antigen processin	BP
antigen processin	BP
antigen processin	BP
antigen processin	BP
antigen processin	BP
protein kinase re	MF
protein phosphat	MF
kinesin binding	MF
axonal transport	BP
extrinsic compon	CC
extrinsic compon	CC
kinase binding	MF
protein kinase bir	MF
phosphatase binc	MF
protein phosphat	MF
protein domain s	MF
syntaxin binding	MF
cyclin-dependent	CC
nuclear cyclin-de	CC
structural constit	MF
cyclin-dependent	MF
lipid storage	BP
peptidyl-arginine	BP
peptidyl-arginine	BP
second-messenge	BP
cAMP-mediated s	BP
cGMP-mediated s	BP
cyclic-nucleotide-	BP
SUMO activating	MF
sexual reproducti	BP
chemokine bindir	MF
C-C chemokine bi	MF
C-X-C chemokine	MF
interleukin-8 binc	MF
C-X3-C chemokin	MF
type I interferon	MF
interferon-gamm	MF
interleukin-1 binc	MF
interleukin-10 bir	MF
interleukin-11 bir	MF
interleukin-2 binc	MF
interleukin-6 binc	MF
diacylglycerol bin	MF
symbiont-contain	CC

symbiont-contain	CC
ciliary pocket me	CC
hemoglobin meta	BP
heme binding	MF
prechordal plate	BP
neural fold elevat	BP
neural fold bendi	BP
floor plate forma	BP
roof plate format	BP
spinal cord devel	BP
spinal cord patte	BP
spinal cord dorsa	BP
ventral spinal cor	BP
cell differentiatio	BP
dorsal spinal cord	BP
ventral spinal cor	BP
spinal cord moto	BP
ventral spinal cor	BP
spinal cord moto	BP
somatic motor ne	BP
visceral motor ne	BP
medial motor col	BP
spinal cord assoc	BP
commissural neu	BP
spinal cord oligod	BP
spinal cord oligod	BP
neural tube patte	BP
cell differentiatio	BP
cell proliferation	BP
cell migration in	BP
diencephalon dev	BP
telencephalon de	BP
subthalamus dev	BP
corpus callosum	BP
ammon gyrus dev	BP
dentate gyrus de	BP
pallium developm	BP
subpallium devel	BP
cranial nerve dev	BP
rhombomere dev	BP
midbrain-hindbra	BP
pons developmer	BP
cerebellum devel	BP
central nervous s	BP
olfactory nerve d	BP

optic nerve devel	BP
midbrain-hindbra	BP
oculomotor nerv	BP
trochlear nerve d	BP
trigeminal nerve	BP
vestibulocochlea	BP
glossopharyngeal	BP
vagus nerve deve	BP
rhombomere 3 d	BP
rhombomere 4 d	BP
rhombomere 5 d	BP
rhombomere 6 d	BP
hindbrain morph	BP
cerebellum morp	BP
cerebellum struct	BP
ventricular syster	BP
fourth ventricle d	BP
abducens nerve f	BP
cranial nerve mo	BP
facial nerve morp	BP
facial nerve struc	BP
glossopharyngeal	BP
hypoglossal nerv	BP
oculomotor nerv	BP
central nervous s	BP
olfactory nerve st	BP
optic nerve morp	BP
optic nerve struct	BP
trigeminal nerve	BP
trigeminal nerve	BP
vagus nerve mor	BP
vestibulocochlea	BP
vestibulocochlea	BP
rhombomere 3 st	BP
rhombomere 3 fc	BP
rhombomere 5 st	BP
rhombomere 5 fc	BP
lateral ventricle d	BP
nerve developme	BP
third ventricle de	BP
cerebellar Purkin	BP
cerebellar granul	BP
nerve maturation	BP
cerebellar granul	BP
cerebellar granul	BP

cerebellar molecu	BP
cerebellar molecu	BP
cerebellar Purkinj	BP
cerebellar Purkinj	BP
cerebellar Purkinj	BP
cerebellar Purkinj	BP
cerebellar cortex	BP
cerebellar cortex	BP
cerebellar cortex	BP
developmental m	BP
cerebellar Purkinj	BP
cerebellar granul	BP
superior olivary n	BP
principal sensory	BP
cochlear nucleus	BP
vestibular nucleu	BP
facial nucleus dev	BP
striatum develop	BP
caudate nucleus	BP
putamen develop	BP
globus pallidus de	BP
limbic system dev	BP
substantia nigra d	BP
amygdala develop	BP
hippocampus dev	BP
mammillary body	BP
nucleus accumbe	BP
orbitofrontal cort	BP
lateral geniculate	BP
olfactory bulb dev	BP
striatal medium s	BP
smoothened sign	BP
smoothened sign	BP
oligodendrocyte	BP
oligodendrocyte	BP
glial cell fate com	BP
glial cell develop	BP
postganglionic pa	BP
branchiomotor ne	BP
chemorepulsion	BP
thalamus develop	BP
cerebral cortex ce	BP
cerebral cortex re	BP
forebrain anterio	BP
forebrain dorsal/	BP

cerebral cortex ra	BP
cerebral cortex ta	BP
cerebral cortex ra	BP
neuronal-glial int	BP
cell-cell adhesion	BP
cell motility invol	BP
extension of a lea	BP
nucleokinesis inv	BP
layer formation ir	BP
negative regulati	BP
gonadotrophin-re	BP
interneuron migr	BP
embryonic olfact	BP
chemorepulsion i	BP
chemorepulsion i	BP
cell proliferation i	BP
ventricular zone r	BP
neuroblast divisic	BP
pyramidal neuror	BP
cerebral cortex G	BP
hypothalamus de	BP
hypothalamus cel	BP
pyramidal neuror	BP
pyramidal neuror	BP
forebrain radial g	BP
forebrain ventric	BP
Cajal-Retzius cell	BP
forebrain regiona	BP
forebrain generat	BP
forebrain neurobl	BP
Wnt signaling pat	BP
forebrain neuron	BP
forebrain neuron	BP
forebrain cell mig	BP
hypothalamus go	BP
olfactory bulb int	BP
olfactory bulb int	BP
cerebral cortex G	BP
cerebral cortex G	BP
cerebral cortex G	BP
cerebral cortex n	BP
forebrain astrocy	BP
commitment of n	BP
rostrocaudal neu	BP
dorsal/ventral ne	BP

forebrain-midbra	BP
smoothened sign	BP
negative regulatio	BP
neural tube devel	BP
BMP signaling pat	BP
cell proliferation i	BP
cerebellar granule	BP
cerebellar granule	BP
cerebellar Purkinj	BP
smoothened sign	BP
positive regulatio	BP
negative regulatio	BP
radial glia guided	BP
formation of radi	BP
neuronal-glial inte	BP
central nervous sy	BP
central nervous sy	BP
central nervous sy	BP
central nervous sy	BP
central nervous sy	BP
corticospinal trac	BP
anterior commiss	BP
spinal cord ventra	BP
corticospinal neu	BP
corticospinal neu	BP
telencephalon reg	BP
hypothalamus cel	BP
pituitary gland de	BP
adenohypophysis	BP
neurohypophysis	BP
habenula develop	BP
cerebral cortex de	BP
olfactory lobe dev	BP
olfactory cortex d	BP
initiation of neura	BP
neural plate axis s	BP
neural plate medi	BP
neural plate ante	BP
convergent exten	BP
neurogenesis	BP
central nervous sy	BP
central nervous sy	BP
myelination in pe	BP
pallium cell prolif	BP
lateral ganglionic	BP

interkinetic nucle	BP
tangential migrat	BP
telencephalon ce	BP
metencephalon d	BP
corpus callosum c	BP
regulation of rhoc	BP
hair cycle process	BP
membrane dockii	BP
regulation of cell-	BP
negative regulatic	BP
positive regulatio	BP
reproductive proc	BP
protein maturatic	BP
ovulation cycle pi	BP
regulation of cell	BP
mammalian ooge	BP
cellular compone	BP
gland morphoger	BP
ribonucleoprotei	BP
membrane to me	BP
protein to memb	BP
DNA strand elong	BP
extracellular mati	BP
ribonucleoprotei	BP
proteasome acce	CC
cytosolic large rib	CC
cytosolic ribosom	CC
transmitter-gatec	MF
wide pore channe	MF
voltage-gated cha	MF
ion gated channe	MF
leak channel activ	MF
potassium ion lea	MF
voltage-gated cat	MF
acetylcholine-gat	MF
glutamate-gated	MF
serotonin-gated c	MF
GABA-gated chloi	MF
glycine-gated chl	MF
alanine transmen	MF
serine transmeml	MF
inorganic cation t	MF
Intermediate con	MF
regulation of tran	BP
electron transpor	BP

respiratory electr	BP
signal transductic	BP
termination of sig	BP
MHC class I prote	MF
MHC class Ib prot	MF
MHC class II prote	MF
MHC class Ib prot	MF
MHC class Ib prot	MF
CD40 signaling pa	BP
neuronal signal tr	BP
regulation of sign	BP
signaling	BP
signal release	BP
metal ion transpo	BP
cellular cation ho	BP
cellular potassiun	BP
TRAPP complex	CC
establishment of	BP
maintenance of c	BP
CCR4-NOT compl	CC
CCR4-NOT core c	CC
myofibril	CC
sarcomere	CC
extracellular matr	MF
extracellular matr	MF
extracellular matr	MF
cellular mangane	BP
lamellipodium	CC
actin filament-bas	BP
cell projection or	BP
cell projection as	BP
lamellipodium as	BP
microvillus assem	BP
microvillar actin b	BP
microspike assem	BP
actin cytoskeletor	BP
actin filament rec	BP
contractile actin f	BP
actin filament pol	BP
actin filament dep	BP
actin filament fra	BP
parallel actin filar	BP
actin modificatior	BP
actin filament-bas	BP
muscle filament s	BP

vesicle transport	BP
cell-substrate jun	CC
hemidesmosome	CC
desmosome	CC
L-malate dehydrc	MF
mitochondrial cri:	CC
insulin processing	BP
regulation of mit	BP
peptide hormone	BP
insulin secretion	BP
protein repair	BP
hemopoiesis	BP
lymphocyte differ	BP
myeloid cell diffe	BP
regulation of end	BP
natural killer cell	BP
vasopressin secre	BP
water homeostas	BP
HLA-A specific inh	MF
HLA-A specific act	MF
HLA-B specific inh	MF
regulation of Wnt	BP
glial cell-derived	MF
membrane coat	CC
clathrin coat	CC
AP-type membrar	CC
vesicle coat	CC
AP-1 adaptor com	CC
AP-2 adaptor com	CC
AP-3 adaptor com	CC
AP-4 adaptor com	CC
clathrin vesicle cc	CC
COPI vesicle coat	CC
COPII vesicle coat	CC
clathrin coat of er	CC
clathrin coat of tr	CC
clathrin adaptor c	CC
clathrin coat of cc	CC
transport vesicle	CC
COPII-coated ER t	CC
coated vesicle	CC
clathrin-coated ve	CC
COPI-coated vesic	CC
endocytic vesicle	CC
trans-Golgi netwo	CC

secretory granule	CC
COPI-coated Golg	CC
alpha-1,6-manno:	MF
manganese ion bi	MF
sphingolipid biosy	BP
sphingolipid catal	BP
protein import in	BP
molybdenum ion	MF
cell differentiatio	BP
regulation of cell	BP
benzodiazepine r	MF
pancreatic juice s	BP
protein xylosyltra	MF
signaling recepto	MF
synaptic receptor	MF
regulation of prot	BP
protein catabolic	BP
PDZ domain bindi	MF
proteoglycan bios	BP
proteoglycan cata	BP
platelet activatio	BP
low-density lipop	MF
pyridoxal phosph	MF
voltage-gated pro	MF
troponin C bindin	MF
integral compone	CC
regulation of DNA	BP
filopodium	CC
positive regulatio	BP
negative regulatio	BP
neuron differenti	BP
B cell differentiat	BP
nitric oxide transı	MF
nitric oxide transp	BP
melatonin biosyn	BP
regulation of bloo	BP
positive regulatio	BP
negative regulatio	BP
extracellular matı	MF
extracellular matı	BP
collagen fibril org	BP
heparan sulfate p	BP
heparan sulfate p	BP
heparin metaboli	BP
glycosaminoglyca	BP

chondroitin sulfat	BP
dermatan sulfate	BP
chondroitin sulfat	BP
chondroitin sulfat	BP
dermatan sulfate	BP
dermatan sulfate	BP
heparin biosynthe	BP
heparin catabolic	BP
hyaluronan metal	BP
hyaluronan biosy	BP
hyaluronan catab	BP
semaphorin recep	MF
keratinocyte diffe	BP
T cell differentiati	BP
erythrocyte differ	BP
megakaryocyte d	BP
platelet formatio	BP
basophil different	BP
neutrophil differe	BP
monocyte differe	BP
macrophage diffe	BP
apolipoprotein re	MF
lipoprotein partic	MF
very-low-density	MF
deoxynucleotide	MF
enzyme regulator	MF
nitric-oxide synth	MF
female sex deterr	BP
male sex determi	BP
myofibril assemb	BP
skeletal muscle th	BP
skeletal muscle m	BP
autophagy of per	BP
carbohydrate bin	MF
polysaccharide bi	MF
guanylate cyclase	MF
guanylate cyclase	MF
growth hormone	BP
lipid modification	BP
lipid glycosylatior	BP
chromosome con	BP
apoptotic nuclear	BP
apoptotic chromo	BP
nuclear fragment	BP
glyoxylate reduct	MF

5-formyltetrahyd	MF
melanin-concentr	MF
LIM domain bindi	MF
LRR domain bindi	MF
clathrin binding	MF
maintenance of g	BP
regulation of ossi	BP
negative regulatio	BP
structural constit	MF
bone mineralizati	BP
testosterone deh	MF
estrogen recepto	MF
integral compone	CC
dynein complex	CC
protein phosphat	CC
protein serine/th	MF
protein tyrosine k	MF
transmembrane r	MF
receptor signaling	MF
protein kinase act	MF
protein tyrosine k	MF
transmembrane r	MF
receptor signaling	MF
intestinal cholest	BP
regulation of inte	BP
cholesterol transp	BP
deoxynucleotide	BP
heparanase activi	MF
positive regulatio	BP
negative regulatio	BP
poly-N-acetyllacto	BP
junctional membr	CC
T-tubule	CC
osteoclast differe	BP
flagellated sperm	BP
melanocyte differ	BP
transepithelial ch	BP
stabilization of m	BP
respiratory tube d	BP
lung developmen	BP
adrenal gland dev	BP
embryonic limb m	BP
prenylated protei	BP
prenylcysteine ca	BP
DNA damage resp	BP

estrogen recepto	MF
cyclin binding	MF
negative regulatio	BP
DNA polymerase	MF
1-alpha,25-dihydr	MF
vitamin D3 25-hy	MF
structural constit	MF
protein phosphat	MF
syntaxin-3 binding	MF
iron-responsive e	MF
molybdopterin sy	MF
interleukin-17 rec	MF
ICAM-3 receptor	MF
translation repres	MF
urokinase plasmi	MF
serine racemase a	MF
neurotensin rece	MF
sperm mitochonc	BP
fructosamine-3-k	MF
fructose 1,6-bispl	BP
fructosamine me	BP
fructoselysine me	BP
lactose binding	MF
formimidoyltrans	MF
glutamate formin	MF
formimidoyltetra	MF
defecation	BP
production of siR	BP
targeting of mRN	BP
growth cone	CC
site of polarized g	CC
kynureninase acti	MF
sleep	BP
peristalsis	BP
ubiquitin-depend	BP
regulation of com	BP
nuclear migratior	BP
actin cap	CC
actin cortical patc	CC
smooth muscle c	CC
tRNA methylatior	BP
maturation of SSI	BP
hemoglobin bindi	MF
midbody	CC
fatty acid elongat	BP

regulation of bon	BP
negative regulatio	BP
inorganic diphosp	MF
inorganic diphosp	BP
ankyrin binding	MF
BMP signaling pat	BP
regulation of BMI	BP
positive regulatio	BP
negative regulatio	BP
positive regulatio	BP
negative regulatio	BP
snoRNA binding	MF
regulation of axo	BP
negative regulatio	BP
intracellular sterc	BP
intracellular estrc	BP
androgen recepto	BP
dihydrolipoamide	MF
granulocyte macr	CC
structural constit	MF
small nuclear ribc	CC
adult behavior	BP
embryonic genita	BP
male genitalia de	BP
female genitalia c	BP
Hsp70 protein bir	MF
receptor regulato	MF
signaling recepto	MF
receptor inhibito	MF
acetylcholine rec	MF
acetylcholine rec	MF
acetylcholine rec	MF
cyclic nucleotide	MF
cAMP binding	MF
cGMP binding	MF
adenyl nucleotide	MF
bile acid catabolic	BP
collagen catabolic	BP
nuclear body orga	BP
Cajal body organi	BP
PML body organiz	BP
ubiquitin-depend	BP
[methionine synt	MF
DNA ADP-ribosyla	BP
neurotransmitter	MF

leukocyte chemo	BP
U1 snRNA binding	MF
U2 snRNA binding	MF
U4 snRNA binding	MF
U4atac snRNA bir	MF
U5 snRNA binding	MF
U6atac snRNA bir	MF
U12 snRNA bindir	MF
pre-mRNA 5'-splic	MF
pre-mRNA 3'-splic	MF
U6 snRNA 3'-end	MF
regulation of cellu	BP
cellular phosphat	BP
cellular chloride i	BP
transport vesicle	CC
Golgi-associated v	CC
coated vesicle me	CC
COPI-coated vesic	CC
clathrin-coated ve	CC
endocytic vesicle	CC
secretory granule	CC
clathrin-coated er	CC
phagocytic vesicle	CC
synaptic vesicle m	CC
axolemma	CC
protein-macromo	MF
ribonuclease P co	CC
mitochondrial rib	CC
multimeric ribonu	CC
mitigation of host	BP
mitigation of host	BP
preribosome	CC
90S preribosome	CC
preribosome, larg	CC
preribosome, sma	CC
Noc1p-Noc2p cor	CC
Noc2p-Noc3p cor	CC
Noc4p-Nop14p co	CC
GTPase regulator	MF
S-adenosylmethio	MF
vitelline membra	BP
cytoskeleton-dep	BP
germ-line stem ce	BP
P granule organiz	BP
ovulation	BP

acetoacetate-CoA	MF
sequestering of tı	BP
guanidinoacetate	MF
carnosine N-meth	MF
GTP-dependent p	MF
amine N-methyltı	MF
arsenite methyltr	MF
methylarsonite m	MF
regulation of acti	BP
regulation of acti	BP
regulation of acti	BP
negative regulatio	BP
positive regulatio	BP
negative regulatio	BP
positive regulatio	BP
positive regulatio	BP
phospholipase C-	BP
autosome	CC
prostate gland de	BP
granulocyte differ	BP
regulation of gran	BP
negative regulatio	BP
positive regulatio	BP
epithelial cell diff	BP
regulation of epit	BP
negative regulatio	BP
positive regulatio	BP
polarized epitheli	BP
regulation of pola	BP
positive regulatio	BP
cortical cytoskele	CC
cortical actin cyto	CC
cortical cytoskele	BP
cortical actin cyto	BP
rough endoplasm	CC
smooth endoplas	CC
Mre11 complex	CC
beta-catenin dest	CC
thyroid gland dev	BP
mammary gland (	BP
RNA polymerase	CC
beta-2-microglob	MF
lipid antigen bind	MF
endogenous lipid	MF
exogenous lipid a	MF

negative regulatio	BP
regulation of B ce	BP
negative regulatio	BP
positive regulatio	BP
VCB complex	CC
mitotic cohesin co	CC
meiotic cohesin c	CC
replisome	CC
apolipoprotein B	CC
checkpoint clamp	CC
HOPS complex	CC
actin-dependent	MF
calcium-depende	MF
forebrain develop	BP
midbrain develop	BP
hindbrain develop	BP
notochord develo	BP
retromer comple:	CC
retromer, tubulat	CC
retromer, cargo-s	CC
olfactory placode	BP
TPR domain bindi	MF
paranodal junctio	BP
STAGA complex	CC
Smc5-Smc6 comp	CC
otic vesicle forma	BP
midbrain-hindbra	BP
endoplasmic retic	MF
mitochondrion ta	MF
regulation of vasc	BP
negative regulatio	BP
positive regulatio	BP
establishment or	BP
establishment or	BP
astral microtubul	BP
astral microtubul	BP
glutamyl-tRNA(Gl	CC
Tat protein bindir	MF
peptidyl-arginine	BP
endoplasmic retic	BP
retrograde protei	BP
receptor tyrosine	MF
thiamine pyropho	BP
thiamine binding	MF
thiamine pyropho	MF

taurine binding	MF
cortical microtub	CC
mismatched DNA	MF
kininogen binding	MF
intraciliary transp	CC
intraciliary transp	CC
intraciliary transp	CC
regulation of cent	BP
response to caffe	BP
potassium ion-tra	CC
filamin binding	MF
ISWI-type comple	CC
Ino80 complex	CC
extracellular matr	CC
troponin I binding	MF
troponin T bindin	MF
pancreas develop	BP
exocrine pancrea	BP
endocrine pancre	BP
interphase micro	CC
nuclear migration	BP
microtubule orga	BP
actomyosin struc	BP
myosin filament a	BP
dense core granu	CC
gene silencing by	BP
heterochromatin	BP
chromosome bre	BP
primary miRNA p	BP
pre-miRNA proce	BP
regulation of hist	BP
positive regulatio	BP
regulation of hist	BP
negative regulatio	BP
positive regulatio	BP
regulation of hist	BP
negative regulatio	BP
positive regulatio	BP
hair follicle morp	BP
cysteine desulfur	MF
heat shock protei	MF
cholesterol 26-hy	MF
embryonic camer	BP
post-embryonic c	BP
nuclear pore oute	CC

BLOC-1 complex	CC
BLOC-2 complex	CC
BLOC-3 complex	CC
nuclear-transcribe	BP
deadenylation-in	BP
platelet dense gra	CC
platelet dense gra	CC
organelle membr	CC
platelet alpha gra	CC
platelet alpha gra	CC
platelet alpha gra	CC
platelet dense tul	CC
platelet dense tul	CC
stress-activated p	BP
regeneration	BP
animal organ rege	BP
neuron projectior	BP
axon regeneratio	BP
dendrite regener	BP
septin complex	CC
septin ring organi	BP
microtubule poly	BP
regulation of mic	BP
negative regulatic	BP
regulation of mic	BP
regulation of mic	BP
negative regulatic	BP
positive regulatio	BP
positive regulatio	BP
rRNA pseudouridi	BP
tRNA pseudouridi	BP
snRNA pseudouri	BP
cytoplasmic micr	BP
RNA 3'-end proce	BP
mRNA 3'-end pro	BP
rRNA 3'-end proc	BP
alpha-(1,2)-fucos	MF
inductive cell-cell	BP
regulation of axo	BP
sister chromatid	BP
pseudopodium	CC
anaphase-promo	BP
SCF-dependent p	BP
histone methyltr	MF
phosphatidylinosi	BP

rRNA methylatior	BP
neuron projectior	BP
phosphopantethe	MF
peptide modificat	BP
SNARE complex	CC
posttranslational	BP
endoplasmic retic	CC
Sec62/Sec63 com	CC
POZ domain bind	MF
SCAR complex	CC
phosphatidylchol	MF
RSF complex	CC
biomineral tissue	BP
auditory behavior	BP
intrinsic compone	CC
anchored compor	CC
intrinsic compone	CC
intrinsic compone	CC
intrinsic compone	CC
intrinsic compone	CC
extrinsic compon	CC
intrinsic compone	CC
extrinsic compon	CC
intrinsic compone	CC
actin rod assembl	BP
denatured protei	MF
PAN complex	CC
cell leading edge	CC
cell projection me	CC
leading edge mer	CC
lamellipodium me	CC
uropod membrar	CC
pseudopodium m	CC
DNA replication p	CC
Ndc80 complex	CC
death-inducing si	CC
CD95 death-induc	CC
small GTPase bin	MF
pseudopodium or	BP
positive regulatio	BP
regulation of late	BP
regulation of cycl	BP
positive regulatio	BP
regulation of guai	BP
negative regulatic	BP

positive regulatio	BP
retinal ganglion c	BP
Ran protein signa	BP
membrane protei	BP
T cell costimulati	BP
B cell costimulati	BP
replication fork p	BP
replication fork p	CC
intrinsic compone	CC
integral compone	CC
intrinsic compone	CC
integral compone	CC
intrinsic compone	CC
integral compone	CC
intrinsic compone	CC
integral compone	CC
integral compone	CC
extrinsic compon	CC
extrinsic compon	CC
extrinsic compon	CC
extrinsic compon	CC
regulation of cell	BP
negative regulati	BP
positive regulatio	BP
positive regulatio	BP
negative regulati	BP
positive regulatio	BP
regulation of vesi	BP
negative regulati	BP
positive regulatio	BP
positive regulatio	BP
regulation of cell	BP
negative regulati	BP
positive regulatio	BP
anchored compo	CC
N-terminal protei	BP
translation initiat	MF
eukaryotic initiati	MF
ubiquitin conjuga	CC
UBC13-MMS2 co	CC
RNA-directed RN	CC
nuclear RNA-dire	CC
Rad17 RFC-like co	CC
Ctf18 RFC-like co	CC
Elg1 RFC-like com	CC

regulation of pro:	BP
negative regulatic	BP
positive regulatio	BP
regulation of prot	BP
positive regulatio	BP
positive regulatio	BP
sodium ion bindir	MF
lithium ion bindin	MF
chloride ion bindi	MF
lipoic acid binding	MF
carboxylic acid bi	MF
oxylipin biosynthe	BP
NatA complex	CC
NatB complex	CC
NatC complex	CC
L-ascorbic acid bi	MF
cobalamin bindin	MF
RecQ family helic	CC
keratinization	BP
response to meth	BP
box C/D snoRNP (	CC
box H/ACA snoRN	CC
M band	CC
Dbf4-dependent	CC
titin binding	MF
telethonin bindin	MF
mitogen-activatec	MF
mitogen-activatec	MF
BRCA1-BARD1 co	CC
positive regulatio	BP
regulation of mRI	BP
negative regulatic	BP
positive regulatio	BP
fast-twitch skelet	BP
slow-twitch skele	BP
positive regulatio	BP
positive regulatio	BP
glycine betaine tr	BP
cullin-RING ubiqu	CC
Cul2-RING ubiqui	CC
Cul3-RING ubiqui	CC
Cul4A-RING E3 ub	CC
Cul4B-RING E3 ub	CC
Cul5-RING ubiqui	CC
Cul7-RING ubiqui	CC

nuclear envelope	BP
myosin V comple:	CC
myosin VII compl	CC
myosin V binding	MF
chromatin DNA b	MF
nucleosome bind	MF
nucleosomal DNA	MF
nucleosomal hist	MF
chromatin assem	BP
TRAMP complex	CC
mannosyltransfer	CC
protein-containin	BP
heterochromatin	BP
pericentric heter	BP
SUMO activating	CC
Mis6-Sim4 compl	CC
motile cilium	CC
tRNA (m1A) meth	CC
PcG protein comp	CC
Myb complex	CC
brush border mer	CC
filopodium memb	CC
microvillus memb	CC
ruffle organizatio	BP
gonadotropin-rel	MF
thyrotropin-relea	MF
actin cytoskeleto	BP
mRNA cap methy	CC
positive regulatio	BP
peptidyl-proline c	MF
peptidyl-proline 3	MF
peptidyl-proline 4	MF
brain-derived neu	BP
negative regulatic	BP
positive regulatio	BP
mitotic G1 DNA d	BP
intra-S DNA dama	BP
membrane raft o	BP
membrane raft di	BP
hemidesmosome	BP
phospholipase D-	BP
activation of phos	BP
regulation of inos	BP
positive regulatio	BP
nucleotide-activa	CC

cell-substrate adh	BP
wybutosine biosy	BP
centrosomal corc	CC
polyubiquitin mo	MF
neuromuscular ju	CC
nuclear proteaso	CC
cytosolic proteas	CC
spindle pole centr	CC
regulation of feve	BP
positive regulatio	BP
ubiquitin conjuga	MF
beta-endorphin b	MF
telomeric loop fo	BP
opioid receptor b	MF
synaptic vesicle fu	BP
regulation of syna	BP
adenylate cyclase	BP
zymogen activatic	BP
plasminogen activ	BP
killing of cells of c	BP
regulation of mye	BP
negative regulatic	BP
positive regulatio	BP
regulation of nerv	BP
regulation of prot	BP
protein destabiliz	BP
heat generation	BP
negative regulatic	BP
positive regulatio	BP
lipopolysaccharid	BP
regulation of lipo	BP
negative regulatic	BP
positive regulatio	BP
response to nutri	BP
cellular response	BP
cellular response	BP
cellular response	BP
A band	CC
H zone	CC
I band	CC
G-protein beta/ga	CC
G-protein beta-su	MF
G-protein gamma	MF
G-protein beta/ga	MF
adenosine recept	MF

A1 adenosine rec	MF
A2A adenosine re	MF
adrenergic recept	MF
alpha-1A adrener	MF
alpha-1B adrener	MF
alpha-2A adrener	MF
alpha-2B adrener	MF
alpha-2C adrener	MF
beta-1 adrenergic	MF
beta-2 adrenergic	MF
beta-3 adrenergic	MF
adrenomedullin r	MF
angiotensin recep	MF
type 1 angiotensi	MF
type 2 angiotensi	MF
apelin receptor b	MF
endothelin A rece	MF
endothelin B rece	MF
neuromedin B rec	MF
bradykinin recept	MF
C5L2 anaphylatox	MF
haptoglobin bindi	MF
hemoglobin alpha	MF
CXCR5 chemokine	MF
CCR1 chemokine	MF
CCR2 chemokine	MF
CCR3 chemokine	MF
CCR4 chemokine	MF
CCR6 chemokine	MF
CCR7 chemokine	MF
CCR10 chemokine	MF
CX3C chemokine	MF
D1 dopamine rec	MF
D2 dopamine rec	MF
D3 dopamine rec	MF
D4 dopamine rec	MF
D5 dopamine rec	MF
Edg-2 lysophosph	MF
follicle-stimulatin	MF
ghrelin receptor b	MF
kisspeptin recept	MF
lutropin-choriogo	MF
corticotropin hor	MF
type 3 melanocor	MF
type 4 melanocor	MF

type 5 melanocor	MF
type 1 metabotrc	MF
type 2 metabotrc	MF
type 3 metabotrc	MF
type 5 metabotrc	MF
P2Y1 nucleotide r	MF
G protein-couple	MF
type 2A serotonir	MF
haptoglobin-hem	CC
type 1 neuromed	MF
type 2 neuromed	MF
protection from r	BP
olfactory recepto	MF
mu-type opioid re	MF
platelet activating	MF
telomeric 3' overl	BP
EP4 subtype pros	MF
thromboxane A2	MF
proteinase activa	MF
taste receptor bir	MF
V1A vasopressin r	MF
V2 vasopressin re	MF
early endosome r	CC
endosome lumen	CC
early endosome l	CC
late endosome lu	CC
negative regulatic	BP
positive regulatio	BP
TOR signaling	BP
mitochondria-nuc	BP
TORC1 complex	CC
TORC2 complex	CC
negative regulatic	BP
positive regulatio	BP
regulation of chrc	BP
positive regulatio	BP
filamentous actin	CC
regulation of gluc	BP
negative regulatic	BP
regulation of prot	BP
negative regulatic	BP
positive regulatio	BP
medium-chain fat	MF
very long-chain fa	MF
mineralocorticoic	BP

response to corti	BP
mineralocorticoic	MF
nuclear lumen	CC
vesicle lumen	CC
Golgi cisterna	CC
locomotion involv	BP
bombesin recepto	BP
mRNA export fro	BP
insulin-like growt	MF
insulin-like growt	MF
thioesterase bind	MF
N-terminal myrist	MF
regulation of fatty	BP
negative regulatio	BP
positive regulatio	BP
interleukin-28 rec	CC
regulation of TOR	BP
negative regulatio	BP
positive regulatio	BP
early phagosome	CC
phagolysosome	CC
regulation of ARF	BP
positive regulatio	BP
NELF complex	CC
trypsinogen activ	BP
positive regulatio	BP
response to coba	BP
response to magr	BP
myosin light chair	MF
myosin head/nec	MF
myosin tail bindir	MF
myosin II head/ne	MF
myosin heavy cha	MF
myosin II heavy c	MF
integrator comple	CC
small-subunit pro	CC
NAD-dependent l	MF
mitochondrial DN	BP
DSIF complex	CC
guanyl-nucleotide	CC
cardiolipin metab	BP
cardiolipin biosyn	BP
clathrin heavy cha	MF
clathrin light chai	MF
bile acid binding	MF

ciliary basal body	BP
negative regulati	BP
positive regulatio	BP
negative regulati	BP
positive regulatio	BP
bleb	CC
bleb assembly	BP
regulation of end	BP
regulation of rest	BP
negative regulati	BP
positive regulatio	BP
negative regulati	BP
positive regulatio	BP
positive regulatio	BP
negative regulati	BP
NACHT domain bi	MF
Pyrin domain bin	MF
positive regulatio	BP
SAM domain binc	MF
response to food	BP
regulation of resp	BP
positive regulatio	BP
regulation of app	BP
negative regulati	BP
positive regulatio	BP
SMC loading com	CC
sequestering of zi	BP
dense core granu	CC
chromosome pas	CC
DNA insertion or	MF
guanine/thymine	MF
single base insert	MF
dinucleotide inse	MF
single guanine ins	MF
single thymine in	MF
4-aminobutyrate	CC
succinate-semial	MF
activation of prot	BP
activation of prot	BP
cell division site	CC
cleavage furrow	CC
septin cytoskelet	CC
septin collar	CC
dinucleotide repe	MF
ubiquitin-like pro	MF

SUMO binding	MF
SUMO polymer b	MF
septin cytoskelet	BP
acrosin binding	MF
ubiquinone biosy	BP
transposition	BP
transposition, RN	BP
telomere organiz	BP
telomere mainte	BP
telomere assemb	BP
telomere formati	BP
regulation of telo	BP
negative regulati	BP
positive regulatio	BP
negative regulati	BP
regulation of telo	BP
negative regulati	BP
positive regulatio	BP
negative regulati	BP
glucosaminyl-pho	MF
riboflavin transm	MF
riboflavin transpo	BP
negative regulati	BP
positive regulatio	BP
regulation of syn	BP
positive regulatio	BP
negative regulati	BP
regulation of syn	BP
negative regulati	BP
positive regulatio	BP
regulation of acti	BP
negative regulati	BP
positive regulatio	BP
activation of stor	BP
adenosine transp	BP
negative regulati	BP
positive regulatio	BP
secretory granule	BP
purine nucleotide	BP
GMP salvage	BP
IMP salvage	BP
phosphatidylinosi	MF
regulation of cell	BP
negative regulati	BP
positive regulatio	BP

negative regulati	BP
positive regulatio	BP
gonadotropin sec	BP
luteinizing hormo	BP
negative regulati	BP
asymmetric synaр	CC
symmetric synap	CC
AMPA glutamate	CC
central nervous s	BP
peripheral nervo	BP
myelin assembly	BP
central nervous s	BP
peripheral nervo	BP
positive regulatio	BP
ribonuclease H2 c	CC
mismatch repair	CC
MutSalpha compl	CC
MutSbeta comple	CC
negative regulati	BP
positive regulatio	BP
negative regulati	BP
positive regulatio	BP
prostaglandin sec	BP
angiogenin-PRI cc	CC
molybdopterin cc	BP
alanine transport	BP
serine transport	BP
regulation of cho	BP
negative regulati	BP
positive regulatio	BP
aldosterone meta	BP
aldosterone bios	BP
regulation of aldo	BP
negative regulati	BP
positive regulatio	BP
regulation of hor	BP
response to follic	BP
response to estra	BP
oxidized DNA bin	MF
oxidized purine D	MF
oxidized pyrimidi	MF
pyridoxal phosph	BP
oxygen homeosta	BP
intracellular lipid	BP
intracellular sterc	BP

regulation of lipic	BP
negative regulatic	BP
positive regulatio	BP
regulation of chol	BP
negative regulatic	BP
positive regulatio	BP
positive regulatio	BP
regulation of intra	BP
negative regulatic	BP
positive regulatio	BP
regulation of intra	BP
negative regulatic	BP
positive regulatio	BP
MutLalpha compl	CC
photoreceptor co	CC
DNA geometric cl	BP
MHC class I recep	MF
MHC class Ib rece	MF
MHC class II recep	MF
activating MHC cl	MF
MHC class Ib prot	CC
melanosome loca	BP
establishment of	BP
melanosome trar	BP
MutLalpha compl	MF
MutLbeta comple	MF
MutSalpha compl	MF
MutSbeta comple	MF
negative regulatic	BP
positive regulatio	BP
regulation of ion	BP
positive regulatio	BP
regulation of sodi	BP
negative regulatic	BP
positive regulatio	BP
lysosome localiza	BP
extrinsic compon	CC
stereocilium	CC
stereocilium bun	CC
purine-rich negat	MF
regulation of misı	BP
positive regulatio	BP
stereocilium tip	CC
GBD domain bind	MF
beta-N-acetylgala	MF

regulation of pho	BP
positive regulatio	BP
activation of phos	BP
actin filament bu	CC
filopodium tip	CC
regulation of prot	BP
negative regulatic	BP
positive regulatio	BP
cuticular plate	CC
melanosome orga	BP
2-alkenal reducta	MF
activin responsive	CC
protein modificat	BP
protein urmylatic	BP
CBM complex	CC
maltose alpha-glu	MF
demethylase acti	MF
histone demethyl	MF
histone demethyl	MF
histone demethyl	MF
nerve growth fact	BP
endocytic recyclir	BP
slow endocytic re	BP
positive regulatio	BP
negative regulatic	BP
positive regulatio	BP
regulation of cytc	BP
negative regulatic	BP
positive regulatio	BP
Golgi calcium ion	BP
endoplasmic retic	BP
positive regulatio	BP
negative regulatic	BP
Golgi calcium ion	BP
cytoplasmic side	CC
otolith morphoge	BP
otolith formation	BP
Rab protein signa	BP
regulation of Rab	BP
regulation of Ral	BP
Rap protein signa	BP
regulation of Rap	BP
Cdc42 protein sig	BP
regulation of Cdc	BP
detection of mole	BP

detection of mole	BP
response to bacte	BP
response to pepti	BP
response to mura	BP
detection of lipop	BP
detection of mura	BP
detection of pept	BP
muramyl dipeptic	MF
developmental pı	BP
cytokinetic proce	BP
maintenance of p	BP
DNA duplex unwi	BP
endosome transp	BP
endosome to lysc	BP
late endosome tc	BP
negative regulatic	BP
positive regulatio	BP
somite rostral/ca	BP
response to retin	BP
protein exit from	BP
microvillus organ	BP
regulation of mici	BP
regulation of mici	BP
regulation of cellı	BP
regulation of cell	BP
cortical endoplasi	CC
sulfiredoxin activi	MF
mitochondrial tra	BP
CURI complex	CC
ribonucleoside bi	MF
pyrimidine deoxy	MF
pyrimidine ribonı	MF
adenyl deoxyribo	MF
adenyl ribonuclec	MF
guanyl ribonuclec	MF
dATP binding	MF
dGTP binding	MF
response to proge	BP
response to vitan	BP
5'-3' RNA helicase	MF
Golgi cisterna me	CC
ER-dependent pe	BP
growth cone men	CC
multivesicular bo	CC
trans-Golgi netwo	CC

neuron projectior	CC
dendrite membra	CC
dendritic spine m	CC
integral compone	CC
insulin-responsive	CC
protein transport	BP
B cell receptor tra	BP
chemokine recep	BP
interferon-gamm	BP
interleukin-10 prc	BP
tumor necrosis fa	BP
regulation of chei	BP
regulation of inte	BP
regulation of inte	BP
regulation of inte	BP
regulation of inte	BP
regulation of inte	BP
regulation of inte	BP
regulation of inte	BP
regulation of inte	BP
regulation of inte	BP
regulation of inte	BP
regulation of inte	BP
regulation of inte	BP
regulation of tum	BP
negative regulatic	BP
negative regulatic	BP
negative regulatic	BP
negative regulatic	BP
negative regulatic	BP
negative regulatic	BP
negative regulatic	BP
negative regulatic	BP
negative regulatic	BP
negative regulatic	BP
negative regulatic	BP
negative regulatic	BP
negative regulatic	BP
negative regulatic	BP
negative regulatic	BP
negative regulatic	BP
negative regulatic	BP
negative regulatic	BP
negative regulatic	BP
negative regulatic	BP

negative regulati	BP
negative regulati	BP
positive regulatio	BP
positive regulatio	BP
positive regulatio	BP
positive regulatio	BP
positive regulatio	BP
positive regulatio	BP
positive regulatio	BP
positive regulatio	BP
positive regulatio	BP
positive regulatio	BP
positive regulatio	BP
positive regulatio	BP
positive regulatio	BP
positive regulatio	BP
positive regulatio	BP
positive regulatio	BP
positive regulatio	BP
positive regulatio	BP
positive regulatio	BP
negative regulati	BP
positive regulatio	BP
copper-depender	MF
negative regulati	BP
positive regulatio	BP
positive regulatio	BP
RNA biosynthetic	BP
DNA methylation	BP
DNA methylation	BP
Piccolo NuA4 hist	CC
negative regulati	BP
positive regulatio	BP
bile acid secretio	BP
super elongation	CC
regulation of DN/	BP
negative regulati	BP
positive regulatio	BP
monocarboxylic a	BP
saturated monoc	BP
unsaturated mon	BP
ribosome disasse	BP
lead ion binding	MF
negative regulati	BP
positive regulatio	BP

GTPase activating	MF
heterotrimeric G-	MF
uropod organizat	BP
SMN complex	CC
Swi5-Sfr1 comple	CC
low-density lipop	BP
receptor catabolic	BP
low-density lipop	BP
regulation of low-	BP
negative regulatio	BP
positive regulatio	BP
DNA ligase IV con	CC
lacrimal gland dev	BP
neuronal cell bod	CC
sterol response e	MF
negative regulatio	BP
tumor necrosis fa	MF
regulation of natu	BP
negative regulatio	BP
positive regulatio	BP
regulation of natu	BP
positive regulatio	BP
regulation of natu	BP
positive regulatio	BP
regulation of natu	BP
negative regulatio	BP
positive regulatio	BP
positive regulatio	BP
glomerulus devel	BP
glomerular basem	BP
plasma membran	CC
dendrite cytoplas	CC
calcitonin binding	MF
negative regulatio	BP
positive regulatio	BP
response to insuli	BP
cellular response	BP
regulation of stre	BP
negative regulatio	BP
positive regulatio	BP
negative regulatio	BP
positive regulatio	BP
regulation of esta	BP
regulation of prot	BP
regulation of micr	BP

regulation of vacı	BP
negative regulatic	BP
negative regulatic	BP
positive regulatio	BP
nerve growth fact	BP
regulation of tran	BP
negative regulatic	BP
negative regulatic	BP
negative regulatic	BP
positive regulatio	BP
positive regulatio	BP
positive regulatio	BP
spermidine acety	BP
spermine acetyla	BP
putrescine acetyl	BP
circadian regulati	BP
activin receptor s	BP
regulation of acti	BP
negative regulatic	BP
positive regulatio	BP
regulation of sup	BP
negative regulatic	BP
positive regulatio	BP
SREBP signaling p	BP
sterol binding	MF
SREBP-SCAP-Insig	CC
negative regulatic	BP
secretion by cell	BP
mononuclear cell	BP
negative regulatic	BP
positive regulatio	BP
regulation of acti	BP
inositol trisphosp	BP
inositol phosphat	BP
inositol trisphosp	BP
regulation of inos	BP
positive regulatio	BP
collagen metabol	BP
collagen biosynth	BP
negative regulatic	BP
positive regulatio	BP
positive regulatio	BP
regulation of acti	BP
regulation of mus	BP
regulation of mus	BP

release of matrix	BP
membrane insert	MF
protein insertion	BP
keratinocyte activ	BP
mitochondrial res	BP
myosin filament	CC
kainate selective	CC
protein-containin	BP
protein-DNA com	BP
cell part morphog	BP
protein-DNA com	CC
protein-lipid com	CC
Bcl3-Bcl10 compl	CC
Fc-epsilon recept	CC
muscle cell prolif	BP
regulation of mas	BP
negative regulati	BP
positive regulatio	BP
negative regulati	BP
positive regulatio	BP
paranodal junctio	CC
perinuclear theca	CC
tetrapyrrole meta	BP
tetrapyrrole biosy	BP
sarcoplasmic reti	CC
mast cell homeos	BP
regulation of mas	BP
negative regulati	BP
myeloid cell apop	BP
regulation of neu	BP
negative regulati	BP
positive regulatio	BP
regulation of mye	BP
negative regulati	BP
positive regulatio	BP
polysaccharide lo	BP
bitter taste recep	MF
sour taste recept	MF
sweet taste recep	MF
regulation of orga	BP
regulation of chro	BP
regulation of siste	BP
directional locom	BP
cellular pigmenta	BP
Rad51B-Rad51C-I	CC

Rad51C-XRCC3 cc	CC
isoquinoline alkal	BP
T cell differentiati	BP
immature T cell p	BP
regulation of T ce	BP
regulation of extr	BP
negative regulatic	BP
negative regulatic	BP
negative regulatic	BP
positive regulatio	BP
positive regulatio	BP
positive regulatio	BP
Weibel-Palade bc	CC
cis-Golgi network	CC
mitochondrial res	BP
endoplasmic retic	CC
negative regulatic	BP
positive regulatio	BP
regulation of hist	BP
negative regulatic	BP
positive regulatio	BP
acetylcholine rec	MF
negative regulatic	BP
positive regulatio	BP
regulation of pep	BP
negative regulatic	BP
positive regulatio	BP
negative regulatic	BP
positive regulatio	BP
progesterone rec	MF
negative regulatic	BP
regulation of intr	BP
negative regulatic	BP
positive regulatio	BP
FFAT motif bindir	MF
cytoskeletal calyx	CC
V(D)J recombinat	BP
immunoglobulin \	BP
T cell receptor V(I	BP
regulation of intr	BP
melanosome mer	CC
interphotorecept	CC
ARC complex	CC
conversion of ds s	BP
histone H3-K9 de	BP

calcineurin-NFAT	BP
proton-transporti	CC
proton-transporti	CC
proton-transporti	CC
proton-transporti	CC
proton-transporti	CC
plasma membran	CC
regulation of hist	BP
positive regulatio	BP
dolichol-phospha	CC
CAF-1 complex	CC
sphingomyelin sy	MF
response to vitam	BP
calmodulin-deper	MF
Lsd1/2 complex	CC
response to hydr	BP
response to vitam	BP
response to ATP	BP
ribonuclease P RN	MF
meiotic cytokines	BP
leptin-mediated s	BP
adiponectin-activ	BP
iron import into c	BP
siderophore-depe	BP
reductive iron ass	BP
amide binding	MF
dsRNA transport	BP
cysteine transme	MF
regulation of prot	BP
negative regulatio	BP
positive regulatio	BP
I-kappaB/NF-kapp	CC
Bcl3/NF-kappaB2	CC
nuclear DNA repli	BP
regulation of nucl	BP
CORVET complex	CC
choline binding	MF
node of Ranvier	CC
internode region	CC
paranode region	CC
response to vitam	BP
response to vitam	BP
transcription fact	CC
abortive mitotic c	BP
cell proliferation	BP

eukaryotic 48S pr	CC
eukaryotic 80S in	CC
T-tubule organiza	BP
secretion of lysos	BP
dehydroascorbic	MF
cell cycle compris	BP
phytol metabolic	BP
meiotic cell cycle	BP
mitotic DNA repli	BP
meiotic spindle as	BP
UDP-D-xylose bio	BP
cerebrospinal flui	BP
Leydig cell differe	BP
peroxisome mem	MF
negative regulatio	BP
positive regulatio	BP
cholesterol efflux	BP
asparagine catab	BP
S-adenosylmethic	BP
secretory granule	BP
mast cell secreto	BP
protein localizatio	BP
protein localizatio	BP
T cell secretory gr	BP
maintenance of p	BP
maintenance of g	BP
geranyl diphosph	BP
geranylgeranyl di	BP
putrescine biosyn	BP
putrescine biosyn	BP
putrescine biosyn	BP
chromatoid body	CC
beta-alanine bios	BP
S-methylmethion	BP
nitric oxide home	BP
cholesterol biosy	BP
galactose catabol	BP
carbohydrate hor	BP
HULC complex	CC
floor plate develo	BP
L-lysine catabolic	BP
L-lysine catabolic	BP
histone H2A ubiq	BP
histone H2B ubiq	BP
fatty acid beta-ox	BP

fatty acid beta-ox	BP
MAP kinase phos	MF
rDNA heterochro	CC
multicellular orga	BP
Slx1-Slx4 comple:	CC
protein deacetyla	MF
unsaturated fatty	BP
regulation of wat	BP
dorsal/ventral ax	BP
anterior/posterio	BP
ESCRT-0 complex	CC
gamma-tubulin co	BP
DNA replication,	BP
transferrin transp	BP
response to testo	BP
protein glycosylat	BP
protein glycosylat	BP
protein galactosy	BP
Elongator holoen	CC
response to coba	BP
response to L-asc	BP
RNA strand annea	MF
BRCA2-MAGE-D1	CC
response to hydro	BP
TSC1-TSC2 compl	CC
mitotic checkpoir	CC
mammary gland	BP
regulation of mar	BP
negative regulatio	BP
positive regulatio	BP
negative regulatio	BP
positive regulatio	BP
negative regulatio	BP
positive regulatio	BP
oxalate metabolic	BP
receptor serine/t	MF
activating transcr	MF
mitochondrial pro	BP
mitochondrial cyt	BP
membrane protei	BP
integrin activatio	BP
regulation of inte	BP
negative regulatio	BP
positive regulatio	BP
positive regulatio	BP

cell adhesion me	BP
regulation of cell	BP
negative regulati	BP
positive regulatio	BP
cell-cell adhesion	BP
regulation of cell-	BP
negative regulati	BP
positive regulatio	BP
negative regulati	BP
negative regulati	BP
positive regulatio	BP
DNA/RNA helicas	MF
5'-3' DNA/RNA he	MF
3'-5' DNA/RNA he	MF
nucleotide-excisi	BP
negative regulati	BP
positive regulatio	BP
osteoblast prolife	BP
regulation of oste	BP
negative regulati	BP
positive regulatio	BP
sialic acid binding	MF
cellular polysacch	BP
neurofilament bu	BP
DNA 5'-adenosine	MF
phospholipid efflu	BP
3beta-hydroxy-5b	MF
aldehyde dehydro	MF
1-pyrroline dehyd	MF
peptide-methioni	MF
L-methionine:thio	MF
L-methionine-(R)-	MF
histone demethyl	MF
histone demethyl	MF
indoleamine 2,3-d	MF
response to gluca	BP
5beta-cholestane	MF
cholesterol 24-hy	MF
25-hydroxycholes	MF
cyanocobalamin r	MF
3alpha,7alpha,12	MF
thyroxine 5-deiod	MF
propanoyl-CoA C-	MF
lipoyl(octanoyl) tr	MF
procollagen gluco	MF

O-fucosylpeptide	MF
N-acetyl-beta-glu	MF
diphosphoinosito	MF
N-acetylgalactosa	MF
negative regulatio	BP
positive regulatio	BP
Fas-activated seri	MF
[heparan sulfate]-	MF
[heparan sulfate]-	MF
hormone-sensitiv	MF
choloyl-CoA hydr	MF
pyridoxal phosph	MF
10-hydroxy-9-(ph	MF
ribonuclease T2 a	MF
hyaluronoglucuro	MF
glucan 1,3-alpha-	MF
mannosyl-glycopr	MF
cis-stilbene-oxide	MF
cytoplasmic mRN	BP
box H/ACA snoRN	BP
D-dopachrome de	MF
3alpha,7alpha,12	MF
response to lipid	BP
FAD-AMP lyase (c	MF
response to silico	BP
deoxyhypusine sy	MF
8-oxo-7,8-dihydro	MF
ATPase-coupled l	MF
ATPase-coupled s	MF
phagophore asse	CC
poly(G) binding	MF
suppression by sy	BP
estrogen respons	MF
RNA strand-excha	MF
endosomal vesicl	BP
response to anox	BP
DNA polymerase	MF
5'-3' RNA polyme	MF
stress granule ass	BP
RIC1-RGP1 guany	CC
protein localizatio	BP
CENP-A containin	BP
establishment of	BP
establishment of	BP
maintenance of n	BP

VCP-NPL4-UFD1 A	CC
erythrocyte home	BP
regulation of tissu	BP
negative regulatio	BP
positive regulatio	BP
homotypic cell-ce	BP
negative regulatio	BP
positive regulatio	BP
heterotypic cell-c	BP
negative regulatio	BP
positive regulatio	BP
positive regulatio	BP
regulation of toll-	BP
negative regulatio	BP
positive regulatio	BP
negative regulatio	BP
negative regulatio	BP
toll-like receptor	BP
toll-like receptor	BP
negative regulatio	BP
positive regulatio	BP
toll-like receptor	BP
negative regulatio	BP
positive regulatio	BP
toll-like receptor	BP
regulation of toll-	BP
negative regulatio	BP
positive regulatio	BP
toll-like receptor	BP
negative regulatio	BP
toll-like receptor	BP
toll-like receptor	BP
positive regulatio	BP
toll-like receptor	BP
regulation of toll-	BP
negative regulatio	BP
toll-like receptor	BP
positive regulatio	BP
apolipoprotein bi	MF
apolipoprotein A-	MF
apolipoprotein A-	MF
very-low-density	MF
apolipoprotein re	MF
apolipoprotein A-	MF
triglyceride trans	BP

cellular response	BP
activation of prot	BP
response to oleic	BP
glycolipid translo	BP
lipid translocation	BP
amyloid-beta forr	BP
GTP-dependent p	MF
quinolinate catab	BP
protein hexameri	BP
carbohydrate tra	BP
cellular response	BP
tRNA thio-modific	BP
enkephalin proce	BP
islet amyloid poly	BP
GPI anchor bindir	MF
protein kinase A c	MF
protein kinase A r	MF
macrophage fusic	BP
positive regulatio	BP
negative regulatic	BP
regulation of tran	BP
negative regulatic	BP
mitochondrial DN	CC
mitochondrial sec	MF
snoRNA splicing	BP
negative regulatic	BP
positive regulatio	BP
phosphatidylinosi	CC
phosphatidylinosi	CC
Atg12-Atg5-Atg16	CC
kynurenic acid bic	BP
Arp2/3 complex-r	BP
regulation of Arp2	BP
negative regulatic	BP
cell junction asse	BP
cell junction mair	BP
adherens junctior	BP
adherens junctior	BP
adherens junctior	BP
RNA folding	BP
short-chain carbo	MF
response to inter	BP
response to type	BP
positive regulatio	BP
glial cell apoptoti	BP

negative regulatio	BP
positive regulatio	BP
RNA pyrophospho	MF
'de novo' NAD bio	BP
NAD salvage	BP
NAD biosynthesis	BP
plasma lipoprotei	CC
mature chylomicr	CC
chylomicron remi	CC
very-low-density	CC
low-density lipop	CC
intermediate-den	CC
high-density lipop	CC
discoidal high-der	CC
spherical high-de	CC
plasma lipoprotei	BP
triglyceride-rich li	BP
chylomicron remo	BP
very-low-density	BP
intermediate-den	BP
low-density lipop	BP
high-density lipop	BP
plasma lipoprotei	BP
chylomicron asse	BP
very-low-density	BP
high-density lipop	BP
plasma lipoprotei	BP
chylomicron remi	BP
low-density lipop	BP
high-density lipop	BP
4-aminobutyrate:	MF
Pwp2p-containing	CC
lipid droplet orga	BP
regulation of smo	BP
negative regulatio	BP
positive regulatio	BP
protein localizatio	BP
regulation of tran	BP
telomere localiza	BP
nuclear periphery	CC
chromatin organi	BP
recruitment of 3'-	BP
response to fluid	BP
tRNA 3'-trailer cle	BP
bisphosphoglycer	MF

urate biosyntheti	BP
post-translationa	BP
autophagosome l	CC
nuclear-transcrib	BP
bis(5'-adenosyl)-l	MF
bis(5'-adenosyl)-p	MF
cholesterol esteri	BP
glycoprotein tran	BP
lipid oxidation	BP
plasma lipoprotei	BP
negative regulatio	BP
substrate adhesio	BP
very-low-density	BP
ubiquitin-ubiquiti	MF
centriolar satellit	CC
dynactin binding	MF
microtubule anch	BP
microtubule anch	BP
t-UTP complex	CC
UTP-C complex	CC
Mpp10 complex	CC
3'-5' RNA helicase	MF
small-subunit pro	BP
90S preribosome	BP
BBSome	CC
response to carbo	BP
ncRNA processing	BP
snRNA 3'-end pro	BP
U1 snRNA 3'-end	BP
U2 snRNA 3'-end	BP
U4 snRNA 3'-end	BP
U5 snRNA 3'-end	BP
U6 snRNA 3'-end	BP
phosphatidylglyce	BP
phosphatidylinosi	MF
vacuolar transme	BP
protein localizatio	BP
early endosome t	BP
late endosome to	BP
protein localizatio	BP
protein localizatio	BP
protein localizatio	BP
tooth mineralizat	BP
chromosome, cer	CC
centromere comp	BP

U3 snoRNA bindi	MF
box C/D snoRNA	MF
box H/ACA snoRN	MF
mitochondrial un	BP
proteasome stora	CC
response to vitan	BP
RNA cap binding	CC
fumarylpyruvate	MF
mitochondrial res	BP
mitochondrial res	BP
piRNA binding	MF
piRNA metabolic	BP
hydroxyproline tr	BP
L-hydroxyproline	MF
phosphatidylinosi	MF
phosphatidylinosi	MF
phosphatidylinosi	MF
phosphatidylinosi	MF
phosphatidylinosi	MF
cellular response	BP
oxoglutarate deh	MF
pyruvate dehydrc	MF
response to tumc	BP
cellular protein lc	BP
cellular response	BP
response to lamir	BP
tetrahydrobiopte	MF
arginine binding	MF
cellular response	BP
cellular protein-c	BP
fatty acid elongat	BP
fatty acid elongat	BP
'de novo' NAD bic	BP
cellular protein-c	BP
retinol transmem	MF
retinol transport	BP
glutathione trans	MF
glutathione trans	BP
phosphatidylchol	BP
establishment of	BP
mitochondrion m	BP
cellular response	BP
cellular macromo	BP
histone demethyl	MF
histone demethyl	MF

histone demethyl	MF
cortisol metaboli	BP
cortisol biosynthe	BP
retinoic acid catal	BP
nucleobase-conta	BP
GID complex	CC
endoplasmic retic	CC
integrin alpha1-b	CC
integrin alpha2-b	CC
integrin alpha3-b	CC
integrin alpha4-b	CC
integrin alpha4-b	CC
chemotaxis to ara	BP
inhibin-betaglyca	CC
integrin alpha5-b	CC
integrin alpha6-b	CC
integrin alpha7-b	CC
integrin alpha8-b	CC
integrin alpha9-b	CC
integrin alpha10-	CC
integrin alpha11-	CC
integrin alphav-b	CC
integrin alphav-b	CC
integrin alphav-b	CC
integrin alphav-b	CC
integrin alphaL-b	CC
integrin alphaM-	CC
integrin alphaX-b	CC
U11/U12 snRNP	CC
response to prost	BP
response to prost	BP
response to gona	BP
response to lutei	BP
ion channel comp	CC
cation channel co	CC
potassium chann	CC
sodium channel c	CC
chloride channel	CC
methyltransferas	CC
methylosome	CC
inhibin binding	MF
type I transformi	MF
type III transform	MF
pICln-Sm protein	CC
SMN-Gemin2 con	CC

SMN-Sm protein	CC
histone H3-K4 de	BP
histone H3-K4 de	BP
gamma-glutamyl-	MF
DNA replication-i	BP
piecemeal microa	BP
nucleosome orga	BP
histone H3-K79 m	BP
cholesterol O-acy	MF
histone deacetyla	MF
Scrib-APC-beta-ca	CC
aryl hydrocarbon	CC
cytosolic aryl hyd	CC
iron ion transmer	BP
regulation of iron	BP
negative regulatio	BP
negative regulatio	BP
regulation of ion	BP
negative regulatio	BP
positive regulatio	BP
basement membr	BP
histone H4-K20 m	BP
histone H4-K20 d	BP
histone H4-K20 tr	BP
secretory granule	CC
glutathione trans	BP
response to hista	BP
recycling endosor	CC
caffeine oxidase a	MF
trimethylamine m	MF
endothelin matur	BP
histone lysine me	BP
histone arginine r	BP
histone H3-R2 me	BP
histone H3-R17 m	BP
histone H3-R26 m	BP
protein folding in	BP
response to endo	BP
NAD-dependent p	MF
mitochondrial pro	BP
peptidyl-lysine de	BP
iron chaperone ac	MF
immunoglobulin r	MF
Fc-gamma recept	MF
nuclear mitotic co	CC

nuclear meiotic c	CC
meiotic nuclear m	CC
oligosaccharyltra	CC
oligosaccharyltra	CC
phosphatidylinosi	MF
1-phosphatidylin	MF
phosphatidylinosi	MF
somatic stem cell	BP
regulation of Rac	BP
negative regulati	BP
positive regulatio	BP
regulation of Rho	BP
negative regulati	BP
positive regulatio	BP
leading edge cell	BP
phosphatidylinosi	CC
histone deacetyla	MF
histone acetyltra	MF
sperm-egg recogn	BP
sperm entry	BP
female pronucleu	BP
male pronucleus	BP
embryonic heart	BP
cardiocyte differe	BP
embryonic heart	BP
brahma complex	CC
interchromatin gr	CC
nuclear speck org	BP
methylated histo	MF
regulation of hist	BP
positive regulatio	BP
negative regulati	BP
axoneme assemb	BP
siRNA loading ont	BP
establishment or	BP
establishment of	BP
phosphatidylinosi	MF
sperm chromatin	BP
spermatogenesis,	BP
response to nicot	BP
behavioral respor	BP
histone methyltra	CC
ESC/E(Z) complex	CC
FACT complex	CC
PRC1 complex	CC

operant condition	BP
limb morphogene	BP
genitalia morpho	BP
embryonic forelir	BP
embryonic hindlir	BP
post-embryonic f	BP
forelimb morpho	BP
hindlimb morpho	BP
exon-exon juncti	CC
tube formation	BP
regulation of tub	BP
embryonic hemo	BP
post-embryonic h	BP
histone kinase ac	MF
histone serine kir	MF
histone kinase ac	MF
social behavior	BP
Rb-E2F complex	CC
post-transcriptior	BP
gene silencing by	BP
production of mi	BP
siRNA binding	MF
miRNA binding	MF
cell competition i	BP
glutamate-cystei	MF
positive regulatio	BP
ectopic germ cell	BP
ionotropic glutar	BP
vitamin A biosynt	BP
tube morphogene	BP
dopamine binding	MF
protein-arginine	MF
protein-arginine	MF
protein-arginine	MF
peptidyl-arginine	BP
peptidyl-arginine	BP
alpha-1,4-N-acety	MF
synaptic transmis	BP
UDP-galactosyltra	MF
UDP-glucosyltran	MF
UDP-xylosyltrans	MF
ciliary rootlet	CC
glutamate recept	MF
ionotropic glutar	MF
G protein-couple	MF

glucocorticoid rec	MF
external genitalia	BP
gonad morphoge	BP
multicellular orga	BP
organ growth	BP
NuA4 histone ace	CC
protein mannosyl	BP
protein O-linked	BP
endocrine system	BP
exocrine system	BP
ethanol binding	MF
miRNA mediated	BP
mRNA cleavage ir	BP
miRNA loading or	BP
pre-miRNA expor	BP
segmentation	BP
brain segmentati	BP
tube developmer	BP
inositol pentakisp	MF
regulation of dep	BP
regulation of prot	BP
negative regulatio	BP
positive regulatio	BP
positive regulatio	BP
negative regulatio	BP
5'-3' exodeoxyrib	MF
wound healing, sp	BP
hair cell different	BP
Toll-like receptor	MF
transcriptionally a	CC
hippo signaling	BP
regulation of hipp	BP
negative regulatio	BP
positive regulatio	BP
Notch receptor pr	BP
peptidyl-tyrosine	BP
long-chain fatty-a	BP
long-chain fatty-a	BP
SPOTS complex	CC
inosine transport	BP
hypoxanthine tra	BP
coenzyme A trans	BP
FAD transmembra	BP
heme transmemb	BP
NAD transmembr	BP

Toll-like receptor	CC
Toll-like receptor	CC
cellular triglyceric	BP
peroxisome prolit	BP
regulation of pero	BP
negative regulatic	BP
positive regulatio	BP
histone locus bod	CC
thymine transpor	BP
selenocysteine in	MF
UBC13-UEV1A co	CC
microtubule plus-	CC
protein localizatic	BP
chondroitin sulfat	MF
chondroitin sulfat	MF
zymogen binding	MF
transepithelial wa	BP
carbon dioxide tra	BP
carbon dioxide tra	MF
Roundabout signa	BP
helper T cell enha	BP
histone kinase ac	MF
histone kinase ac	MF
histone kinase ac	MF
histone-serine ph	BP
histone H3-T11 p	BP
histone H3-T6 ph	BP
histone H3-Y41 p	BP
dihydrotestostero	MF
protein localizatic	BP
autocrine signalir	BP
extracellular matı	BP
copper ion transn	BP
phosphate ion tra	BP
maintenance of p	BP
cyclic-di-GMP bin	MF
cell migration inv	BP
nickel cation tran	BP
borate transmem	BP
vesicle cargo loac	BP
vitamin transmen	BP
determination of	BP
positive regulatio	BP
lipase binding	MF
positive regulatio	BP

gastric emptying	BP
adenine/guanine	MF
positive regulatio	BP
SNARE complex a	BP
SNARE complex d	BP
cAMP response e	MF
carnosine metabo	BP
carnosine biosynt	BP
MH2 domain binc	MF
MH1 domain binc	MF
metanephric part	BP
positive regulatio	BP
regulation of myc	BP
positive regulatio	BP
negative regulatic	BP
oxidative DNA de	BP
oxidative RNA de	BP
oxidative RNA de	MF
oxidative DNA de	MF
PR-DUB complex	CC
histone H2A mon	BP
protein K29-linke	BP
monoubiquitinate	BP
monoubiquitinate	BP
protein K29-linke	BP
proline transmem	BP
NF-kappaB p50/p	CC
retrograde transp	BP
NADH pyrophosp	MF
carbohydrate res	MF
8-oxo-7,8-dihydrc	MF
regulation of SNA	BP
positive regulatio	BP
negative regulatic	BP
determination of	BP
oxidative single-s	BP
oxidative single-s	BP
intracellular signa	BP
negative regulatic	BP
positive regulatio	BP
regulation of kidr	BP
regulation of proi	BP
non-canonical Wi	BP
N-terminal peptic	BP
N-terminal peptic	BP

N-terminal peptic	BP
N-terminal peptic	BP
histone H4-K20 d	BP
histone demethyl	MF
azurophil granule	CC
azurophil granule	CC
specific granule m	CC
specific granule lu	CC
sequestering of e	BP
sequestering of B	BP
sequestering of T	BP
calcium-mediatec	BP
calcium-mediatec	BP
G protein-couple	BP
purinergic nucleo	BP
signaling adaptor	MF
ganglioside bindi	MF
methylthiotransf	MF
N6-isopentenylac	MF
N6-threonylcarbc	MF
tRNA methylthiol	BP
fibroblast growth	BP
fibroblast growth	BP
fibroblast growth	BP
peptidyl-cysteine	MF
peptidyl-cysteine	BP
fibroblast growth	BP
protein deglutam	BP
C-terminal protei	BP
protein side chair	BP
protein branching	BP
AP-2 adaptor com	MF
RNA stem-loop bi	MF
clathrin adaptor a	MF
histone H2B cons	BP
stress granule dis	BP
ER to Golgi ceram	BP
intrahepatic bile	BP
renal glucose abs	BP
receptor transact	BP
ceramide transpo	BP
bone mineralizati	BP
CD40 receptor co	CC
mitochondrial pro	CC
maintenance of b	BP

response to stilbe	BP
entry of bacteriur	BP
purine ribonucleo	MF
exploration beha	BP
locomotory explo	BP
histone methyltra	MF
L-DOPA receptor	MF
phosphoanandan	BP
enteric smooth m	BP
endosome to mel	BP
AP-1 adaptor con	MF
AP-3 adaptor con	MF
interleukin-18-me	BP
eRF1 methyltrans	CC
Mon1-Ccz1 comp	CC
Toll-like receptor	MF
Toll-like receptor	MF
TIRAP-dependent	BP
TRAM-dependent	BP
oligopeptide tran	BP
oligopeptide tran	MF
tricarboxylic acid	BP
neuromast hair ce	BP
memory T cell ext	BP
helper T cell extra	BP
helper T cell diap	BP
sperm fibrous she	CC
cellular response	BP
macrophage migr	BP
macrophage migr	CC
NOS2-CD74 comp	CC
mitochondrial pro	BP
mitophagy by ind	BP
monocyte extrava	BP
hematopoietic ste	BP
monocyte homeo	BP
memory T cell act	BP
T-helper 1 cell act	BP
T-helper 2 cell act	BP
response to nitro	BP
macrophage migr	MF
intraciliary antero	BP
intraciliary retrog	BP
interleukin-12-me	BP
interleukin-15-me	BP

sodium ion transı	BP
common myeloid	BP
lysophosphatidic	MF
response to hepa	BP
cellular response	BP
S-nitrosoglutathic	MF
dinitrosyl-iron coı	MF
nitric oxide storag	BP
hepatic stellate c	BP
intraciliary transp	BP
natural killer cell	BP
myelin sheath ab	CC
myelin sheath ad	CC
protein localizatic	BP
regulation of lyso	BP
lysosomal lumen	BP
B cell chemotaxis	BP
cardiolipin hydrol	MF
chemokine (C-C n	MF
chemokine (C-C n	MF
mesangial cell-ma	BP
endothelial cell cl	BP
endothelial cell cl	BP
ribonucleoproteir	CC
interleukin-4-mec	BP
interleukin-13-me	BP
insulin secretion i	BP
positive regulatio	BP
CD80 biosyntheti	BP
CD86 biosyntheti	BP
CD4-positive, alpl	BP
cell migration inv	BP
metanephric mes	BP
platelet-derived g	BP
platelet-derived g	BP
positive regulatio	BP
positive regulatio	BP
negative regulatic	BP
2-alkenal reducta	MF
ureter maturatioı	BP
deubiquitinase ac	MF
adrenal cortex de	BP
adrenal cortex foı	BP
egg coat formatic	BP
structural constit	MF

egg coat	CC
regulation of urin	BP
positive regulatio	BP
negative regulatio	BP
renal sodium excı	BP
regulation of rena	BP
negative regulatio	BP
positive regulatio	BP
modulation of pro	BP
gene conversion	BP
homologous reco	BP
growing cell tip	CC
new growing cell	CC
cloaca developme	BP
photoreceptor ce	BP
oviduct epitheliur	BP
uterine epitheliur	BP
nephric duct elon	BP
Krueppel-associat	MF
horizontal cell loc	BP
chromosome pas	BP
megakaryocyte d	BP
Seh1-associated c	CC
glial cell-derived r	BP
site of double-str	CC
dITP catabolic pro	BP
response to potas	BP
cellular response	BP
alphav-beta3 inte	CC
alphav-beta3 inte	CC
alphav-beta3 inte	CC
ciliary transition z	CC
dITP diphosphata	MF
protein K11-linke	BP
nucleotide-bindin	BP
lactate transmem	BP
cellular response	BP
death effector do	MF
nail development	BP
plasma membran	BP
embryonic nail pl	BP
amacrine cell diff	BP
enteroendocrine	BP
vascular smooth r	BP
parathyroid horm	BP

response to isolat	BP
response to immo	BP
aorta developme	BP
ascending aorta d	BP
descending aorta	BP
dorsal aorta deve	BP
aorta morphogen	BP
ascending aorta m	BP
dorsal aorta morp	BP
skeletal muscle ce	BP
pore formation in	BP
desmosome disas	BP
cellular response	BP
mRNA 3'-UTR AU-	MF
RNA import into m	BP
rRNA import into	BP
corticosterone se	BP
mitochondrial ncl	BP
mitochondrial mR	BP
cellular response	BP
COPI-coated vesic	BP
cardiolipin acyl-ch	BP
cellular response	BP
peptidyl-threonin	BP
peptidyl-histidine	BP
aggrephagy	BP
transcription fact	CC
histone H2A-S139	BP
cellular response	BP
senescence-assoc	CC
senescence-assoc	BP
endodermal cell d	BP
chondrocyte prol	BP
tendon developm	BP
tendon cell differ	BP
tendon formation	BP
deltoid tuberosity	BP
response to musc	BP
detection of musc	BP
7,8-dihydroneopt	BP
tetrahydrofolate	BP
pre-mRNA bindin	MF
positive regulatio	BP
GAF domain bind	MF
cellular response	BP

protein-glutamine	MF
protein localizatio	BP
cellular response	BP
response to eryth	BP
cellular response	BP
endolysosome	CC
endolysosome m	CC
endolysosome lu	CC
limb joint morpho	BP
embryonic skelet	BP
recruitment of m	BP
mediator comple	MF
osteoclast develo	BP
CD8-positive, alpl	BP
MKS complex	CC
long-chain fatty a	MF
long-chain fatty a	MF
protein demalony	BP
peptidyl-lysine de	BP
protein desucciny	BP
peptidyl-lysine de	BP
glomerular endot	CC
protein-malonylly	MF
protein-succinylly	MF
slit diaphragm	CC
slit diaphragm as	BP
ciliary basal body	CC
fucosylation	BP
protein O-linked f	BP
N-glycan fucosyla	BP
GDP-fucose impo	BP
cleavage furrow f	BP
cleavage furrow i	BP
positive regulatio	BP
phosphatidylinosi	BP
small molecule bi	MF
leukotriene B4 ca	BP
leukotriene B4 m	BP
peroxisome mem	MF
alpha-linolenic ac	BP
very long-chain fa	BP
medium-chain fat	BP
very long-chain fa	BP
medium-chain fat	BP
fatty-acyl-CoA cat	BP

long-chain fatty-a	BP
hyaluranon cable	CC
response to plate	BP
cellular response	BP
double-stranded	MF
BMP binding	MF
histone H3-K9 dir	BP
histone H3-K9 trir	BP
sperm flagellum	CC
CatSper complex	CC
prostaglandin H2	MF
prostaglandin D2	MF
13-prostaglandin	MF
12-hydroxyhepta	MF
kynurenine amino	MF
peptidyl-histidine	BP
peptidyl-histidine	MF
peptidyl-asparagi	MF
kringle domain bi	MF
dendritic cell hom	BP
cellular response	BP
phosphatidylglyce	BP
phosphatidylinosi	BP
phosphatidylserin	BP
phosphatidylchol	BP
phosphatidyletha	BP
triglyceride acyl-c	BP
acylglycerol acyl-c	BP
inner dynein arm	CC
outer dynein arm	CC
outer dynein arm	BP
inner dynein arm	BP
cell-abiotic substr	BP
phenotypic switcl	BP
13-lipoxin reducta	MF
early phagosome	CC
muscle cell projec	CC
protein modificat	BP
dTTP diphosphata	MF
UTP diphosphatas	MF
protein localizatio	BP
cellular response	BP
peroxisome trans	BP
response to meth	BP
multivesicular bo	BP

multivesicular bo	BP
7-methylguanosir	BP
RNA (guanine-N7	BP
swimming	BP
swimming behavi	BP
tubulobulbar con	CC
peptidyl-serine au	BP
DNA rewinding	BP
response to decre	BP
cellular response	BP
cellular response	BP
interstrand cross-	BP
recombinational i	BP
atrioventricular c	BP
lymph vessel mor	BP
umbilical cord mc	BP
ameloblast differ	BP
protein localizatic	BP
annealing helicas	MF
phosphatidylinosi	MF
phosphatidylinosi	MF
cellular response	BP
SREBP-SCAP com	BP
tyrosyl-RNA phos	MF
ghrelin secretion	BP
vascular endothe	BP
vascular endothe	BP
placental growth	MF
hepatocyte home	BP
intestinal stem ce	BP
dendritic cell mig	BP
Fas signaling path	BP
lymphocyte adhe	BP
post-anal tail mor	BP
psychomotor beh	BP
platelet morphog	BP
platelet maturatic	BP
histone H2A-K13	BP
histone H2A-K15	BP
histone H2A-K119	BP
renal potassium e	BP
transforming grov	BP
transforming grov	BP
transcription fact	BP
protein localizatic	BP

opsin transport	BP
L-fucose mutarot	MF
glutathione hydrc	MF
sodium ion expor	BP
calcitriol biosynth	BP
myofilament	CC
cytidine diphosph	MF
pre-replicative co	BP
thiocyanate pero:	MF
RNA N6-methylac	CC
proteasome-activ	MF
arachidonate 8(S)	MF
conversion of ds :	BP
histone H3-K14 a	CC
histone H3-R26 ci	BP
histone citrullinat	BP
tRNA stabilizatior	BP
L-phosphoserine	MF
D-phosphoserine	MF
K48-linked polyul	MF
maintenance of le	BP
citrate synthase a	MF
calcium import in	BP
microtubule minu	CC
L-methionine-(S)-	MF
keratohyalin gran	CC
hepatocyte growt	MF
BLOC-2 complex l	MF
TRAIL-activated a	BP
TRAIL receptor ac	MF
cytoplasmic ribor	CC
synaptic vesicle re	BP
synaptic vesicle re	BP
5-hydroxy-L-trypt	MF
L-dopa decarboxy	MF
tyrosine 3-monoc	MF
cellular response	BP
cell death in resp	BP
cell death in resp	BP
neuron death in r	BP
somatodendritic c	CC
L-dopa decarboxy	MF
peroxidase inhibi	MF
intrinsic apoptoti	BP
ventral trunk neu	BP

nitric-oxide synth	MF
CHOP-C/EBP com	CC
eiF2alpha phosph	BP
positive regulatio	BP
positive regulatio	BP
regulation of tran	BP
IRE1-mediated ur	BP
PERK-mediated u	BP
ATF6-mediated u	BP
UFD1-NPL4 comp	CC
Derlin-1-VIMP co	CC
ERAD pathway	BP
prosaposin recep	MF
protein alpha-1,2	BP
trimming of term	BP
trimming of term	BP
trimming of first	BP
trimming of seco	BP
Derlin-1 retrotrar	CC
dopaminergic neu	BP
serotonergic neu	BP
chemoattraction	BP
chemorepulsion	BP
astrocyte-dopam	BP
protein deglycase	MF
protein deglycati	BP
peptidyl-cysteine	BP
peptidyl-arginine	BP
peptidyl-lysine de	BP
protein deglycati	BP
protein deglycati	BP
glutathione degly	BP
paracrine signalin	BP
endocrine signali	BP
opioid receptor si	BP
netrin-activated s	BP
TRAF-mediated si	BP
insulin receptor i	BP
Wnt receptor cat	BP
insulin receptor r	BP
leptin receptor ac	MF
signaling recepto	MF
cargo receptor ac	MF
reelin receptor ac	MF
reelin-mediated s	BP

apolipoprotein A-	BP
insulin receptor s	BP
epidermal growtl	BP
non-canonical Wı	BP
non-canonical Wı	BP
positive regulatio	BP
sphingosine-1-ph	MF
G protein-couplei	CC
G protein-couplei	CC
G protein-couplei	CC
cross-receptor inl	BP
interleukin-5-mec	BP
enkephalin recep	MF
morphine recept	MF
dynorphin recept	MF
nitric oxide-cGMI	BP
NIK/NF-kappaB si	BP
protein tyrosine k	MF
collagen-activate	BP
collagen receptor	MF
collagen-activate	BP
p38MAPK cascad	BP
peptidyl-tyrosine	BP
vascular endothe	BP
vascular endothe	MF
positive regulatio	BP
positive regulatio	BP
nodal signaling pa	BP
Fc receptor signal	BP
Fc-gamma recept	BP
Fc-epsilon recept	BP
Fc-gamma recept	BP
positive regulatio	BP
sequestering of B	BP
nodal binding	MF
sequestering of n	BP
activin receptor a	MF
negative regulatic	BP
Kit signaling path	BP
interleukin-2-mec	BP
interleukin-7-mec	BP
interleukin-8-mec	BP
chemokine (C-C n	BP
chemokine (C-C n	BP
C-C motif chemol	MF

C-C motif chemol	MF
toll-like receptor	BP
toll-like receptor	BP
ERBB signaling pa	BP
ERBB2 signaling p	BP
ERBB3 signaling p	BP
ERBB4 signaling p	BP
neuregulin recept	MF
neuregulin bindin	MF
ERBB3:ERBB2 cor	CC
macrophage colo	BP
chemokine (C-X-C	BP
C-X-C motif chem	MF
interleukin-11-me	BP
interleukin-23-me	BP
interleukin-3-mec	BP
granulocyte color	BP
CXCL12-activated	BP
prolactin signalin	BP
erythropoietin-m	BP
thrombopoietin-r	BP
thrombopoietin r	MF
oncostatin-M-me	BP
angiotensin-activ	BP
epidermal growtl	BP
somatostatin sigr	BP
cannabinoid signa	BP
interleukin-33-me	BP
interleukin-17A-n	BP
death receptor ag	MF
complement com	BP
neurotrophin sigr	BP
nerve growth fact	BP
bile acid receptor	MF
G protein-couple	MF
bile acid signaling	BP
cell surface bile a	BP
lithocholic acid re	MF
pattern recogniti	MF
neuropilin signali	BP
VEGF-activated n	BP
neuropilin bindin	MF
gastric inhibitory	BP
thromboxane A2	BP
thyroid-stimulatir	BP

urokinase plasmi	BP
TORC1 signaling	BP
TORC2 signaling	BP
pronephric field s	BP
pronephric nephr	BP
pronephric nephr	BP
pronephric nephr	BP
pronephric duct r	BP
induction by virus	BP
suppression by vi	BP
negative regulatio	BP
regulation of RIG-	BP
RIG-I binding	MF
suppression by vi	BP
suppression by vi	BP
suppression by vi	BP
fusion of virus me	BP
negative strandec	BP
single stranded vi	BP
viral RNA genome	BP
DNA-templated v	BP
viral budding via l	BP
co-receptor bindi	MF
establishment of	BP
regulation of grov	BP
regulation of grov	BP
positive regulatio	BP
locomotion	BP
regulation of locc	BP
negative regulatio	BP
regulation of mul	BP
negative regulatio	BP
embryonic cleava	BP
positive regulatio	BP
positive regulatio	BP
positive regulatio	BP
regulation of mei	BP
regulation of gen	BP
regulation of mol	BP
snRNA modificati	BP
regulation of dev	BP
regulation of fibr	BP
negative regulatio	BP
polar body extrus	BP
thermosensory b	BP

interleukin-18 bir	MF
interleukin-18 rec	MF
interleukin-15 rec	MF
interleukin-16 bir	MF
interleukin-16 rec	MF
interleukin-20 bir	MF
interleukin-23 bir	MF
interleukin-23 rec	MF
interleukin-12 rec	CC
DNA endoredupli	BP
protein refolding	BP
ATPase inhibitor a	MF
peptidyl-histidine	BP
neurexin family p	MF
fluid transport	BP
epithelial fluid tra	BP
olfactory behavio	BP
regulation of dop	BP
negative regulatio	BP
wound healing	BP
gliogenesis	BP
intraciliary transp	BP
cell migration inv	BP
T-helper 1 type in	BP
type 2 immune re	BP
T-helper cell diffe	BP
T cell proliferatio	BP
B cell proliferatio	BP
T cell receptor co	CC
positive regulatio	BP
positive regulatio	BP
alpha-beta T cell r	CC
T cell activation	BP
B cell activation	BP
macrophage activ	BP
monocyte activat	BP
neutrophil activat	BP
nitrate metabolic	BP
regulation of T ce	BP
negative regulatio	BP
thiamine phospha	MF
fructose 1,6-bispl	MF
neurotransmitter	BP
rRNA primary tra	MF
neurotransmitter	BP

neurotransmitter	BP
meiotic DNA doul	BP
vacuole fusion, no	BP
retrograde transp	BP
strand invasion	BP
cellular response	BP
lipoprotein metal	BP
lipoprotein biosyı	BP
lipoprotein catab	BP
telomeric DNA bi	MF
interleukin-12 be	MF
neurotransmitter	MF
acetylcholine bin	MF
heme catabolic p	BP
heme metabolic p	BP
SH2 domain bindi	MF
lysophosphatidic	MF
nuclear outer me	CC
regulation of prot	BP
negative regulatio	BP
xenobiotic catabo	BP
cellular ketone m	BP
ketone catabolic	BP
halogenated hydr	BP
1-aminocyclopro	BP
cellular modified	BP
response to cocai	BP
RNA repair	BP
tissue regeneratio	BP
establishment of	BP
establishment of	BP
ribosome biogene	BP
ribosome assemb	BP
mature ribosome	BP
DNA protection	BP
peptidyl-aspartic	BP
peptidyl-asparagi	BP
natural killer cell	BP
regulation of natu	BP
protection from r	BP
susceptibility to n	BP
nuclear RNA expo	CC
ribosomal large s	BP
ribosomal small s	BP
error-free postre	BP

error-prone trans	BP
peptide binding	MF
purine nucleoside	BP
dolichyl pyrophos	MF
hydroxymethylglu	MF
dolichyl pyrophos	MF
sphingolipid delta	MF
xylosyltransferase	MF
MHC protein binc	MF
MHC class I prote	MF
MHC class II prote	MF
URM1 activating	MF
vocal learning	BP
beta-amyrin synth	MF
phosphate ion bir	MF
regulation of fatty	BP
regulation of prot	BP
positive regulatio	BP
negative regulatio	BP
vasoconstriction	BP
vasodilation	BP
protein kinase C c	BP
regulation of circa	BP
negative regulatio	BP
negative regulatio	BP
negative regulatio	BP
regulation of pho	BP
negative regulatio	BP
positive regulatio	BP
heparan sulfate N	MF
cuticle developme	BP
keratan sulfate m	BP
keratan sulfate ca	BP
'de novo' GDP-L-f	BP
GDP-L-fucose salv	BP
L-fucose catabolic	BP
GDP-4-dehydro-D	MF
thiamine diphosp	BP
vitamin D metabo	BP
vitamin E metabo	BP
menaquinone cat	BP
vitamin D biosynt	BP
vitamin D catabol	BP
vitamin K biosynt	BP
vitamin K metabo	BP

phylloquinone ca	BP
vitamin K catabol	BP
chemokine recep	MF
paraspeckles	CC
regulation of mer	BP
sphingosine-1-ph	MF
thyroid hormone	BP
thyroid hormone	BP
nuclear inclusion	CC
extrinsic compon	CC
cristae formation	BP
taurine biosynthe	BP
carnitine cataboli	BP
epinephrine meta	BP
norepinephrine m	BP
dopamine biosyn	BP
dopamine metab	BP
epinephrine biosy	BP
dopamine catabo	BP
norepinephrine b	BP
catecholamine bi	BP
catecholamine ca	BP
choline catabolic	BP
serotonin biosynt	BP
serotonin metabo	BP
indole metabolic	BP
melanin biosynth	BP
hormone metabo	BP
hormone biosynt	BP
hormone catabol	BP
progesterone me	BP
arginine biosynth	BP
photoreceptor ce	BP
eye photorecepto	BP
melanosome	CC
ear morphogenes	BP
inner ear morpho	BP
outer ear morpho	BP
middle ear morph	BP
odontogenesis of	BP
odontogenesis	BP
regulation of odo	BP
positive regulatio	BP
regulation of odo	BP
positive regulatio	BP

negative regulatio	BP
mechanorecepto	BP
inner ear auditory	BP
gamma-delta T ce	BP
detection of bact	BP
detection of triac	BP
detection of diacy	BP
triacyl lipopeptide	MF
aspartic endopep	MF
serine phosphory	BP
regulation of tyro	BP
positive regulatio	BP
negative regulatio	BP
hyperosmotic sali	BP
hemoglobin biosy	BP
protein N-linked g	BP
neuron maturatio	BP
myelination	BP
superoxide anion	BP
MCM complex	CC
pteridine-contain	BP
pteridine-contain	BP
hormone binding	MF
NLS-dependent p	CC
RNA nuclear expo	CC
insulin-like growt	CC
insulin-like growt	CC
immunoglobulin c	CC
retinol metabolic	BP
retinoic acid meta	BP
retinal metabolic	BP
DNA polymerase	CC
lipid phosphatase	MF
phosphoric ester	MF
specific granule	CC
azurophil granule	CC
chromaffin granu	CC
chromaffin granu	CC
germinal vesicle	CC
peptide deformyl	MF
glycogen granule	CC
zymogen granule	CC
zymogen granule	CC
antigen processin	BP
homeostatic proc	BP

glucose homeosta	BP
response to starv	BP
behavioral respor	BP
fear response	BP
lamellar body	CC
riboflavin reducta	MF
peptide antigen b	MF
T cell receptor bir	MF
CD4 receptor bin	MF
CD8 receptor bin	MF
MHC class I prote	CC
MHC class II prot	CC
photoreceptor ou	CC
ATPase-coupled i	MF
ATPase-coupled t	MF
chylomicron	CC
mating plug form	BP
mast cell granule	CC
cellular response	BP
cholesterol home	BP
hair cycle	BP
regulation of hair	BP
positive regulatio	BP
negative regulati	BP
actomyosin	CC
mitochondrial nu	CC
MHC class II prot	MF
regulation of cell	BP
positive regulatio	BP
negative regulati	BP
regulation of end	BP
negative regulati	BP
negative regulati	BP
regulation of inne	BP
muscle cell differ	BP
muscle cell fate c	BP
ovulation cycle	BP
follicle-stimulatin	BP
luteinizing hormo	BP
progesterone sec	BP
uterine wall breal	BP
succinate-CoA lig	CC
maternal behavio	BP
sperm ejaculatior	BP
mitochondrial int	CC

mitochondrial inr	CC
TIM22 mitochonc	CC
thiamine-contain	BP
fibrinolysis	BP
PH domain bindir	MF
D-xylose metabol	BP
embryonic digit n	BP
presynaptic mem	CC
drug catabolic prc	BP
exogenous drug c	BP
hydrogen peroxic	BP
hydrogen peroxic	BP
circadian sleep/w	BP
circadian sleep/w	BP
circadian sleep/w	BP
regulation of circa	BP
regulation of circa	BP
positive regulatio	BP
negative regulatic	BP
eating behavior	BP
drinking behavior	BP
long-chain fatty a	BP
long-chain fatty a	BP
very long-chain fa	BP
very long-chain fa	BP
GPI-anchor transa	CC
nucleosome mob	BP
DNA damage resp	BP
signal transductic	BP
intrinsic apoptoti	BP
DNA damage resp	BP
ATP synthesis cou	BP
plasma membran	BP
mitochondrial AT	BP
mitochondrial AT	BP
tRNA 3'-trailer cle	BP
tRNA 3'-end proc	BP
3'-tRNA processir	MF
polysomal riboso	CC
mRNA transcripti	BP
nucleolar large rR	BP
5S class rRNA trar	BP
snRNA transcripti	BP
snRNA transcripti	BP
tRNA transcriptio	BP

histone methyltra	MF
actinin binding	MF
fucose binding	MF
vitamin D recepto	MF
Wnt-activated re	MF
vitamin B6 metab	BP
pyridoxal phosph	BP
MHC class I pepti	CC
TAP complex	CC
histone deacetyla	MF
platelet dense gra	CC
defense response	BP
peptidoglycan bir	MF
BRE binding	MF
D-glucuronate ca	BP
D-xylose catabolic	BP
L-alanine metabo	BP
L-alanine cataboli	BP
pyruvate biosynth	BP
cysteine transpor	BP
amide transport	BP
amide transmeml	MF
tubulin deacetyla	MF
9-cis-retinoic acid	BP
xenobiotic transp	BP
xenobiotic transm	MF
glucocorticoid rec	BP
neuromedin U re	MF
neuropeptide bin	MF
neuromedin U bir	MF
enterobactin tran	BP
enterobactin tran	MF
tripeptide transm	MF
dipeptide transpo	BP
D-serine transpor	BP
D-serine transme	MF
glucoside transpo	BP
glucoside transm	MF
lipoprotein transp	BP
retinoic acid rece	MF
peroxisome prolif	MF
activation of Janu	BP
ornithine decarbo	MF
amyloid precurso	BP
amyloid precurso	BP

regulation of amy	BP
negative regulati	BP
positive regulatio	BP
amyloid precurso	BP
X11-like protein b	MF
sequestering of a	BP
cytoplasmic sequ	BP
negative regulati	BP
positive regulatio	BP
Golgi to plasma m	BP
Golgi to plasma m	BP
cytoplasmic sequ	BP
neuron projection	CC
activation of phos	BP
maintenance of r	BP
ATP-dependent p	MF
chordate embryo	BP
camera-type eye	BP
myeloid dendritic	BP
regulation of fusi	BP
alpha-tubulin bin	MF
gamma-tubulin b	MF
NADPH oxidase c	CC
ribonucleoprotein	MF
ribosome binding	MF
ribosomal large s	MF
ribosomal small s	MF
neuronal cell bod	CC
cysteine-type enc	MF
cysteine-type enc	MF
T cell homeostasi	BP
regulation of mac	BP
negative regulati	BP
positive regulatio	BP
isoamylase comp	CC
costamere	CC
chromatin insulat	MF
tRNA aminoacyla	BP
amino acid activa	BP
peptide biosynthe	BP
ATP-dependent c	BP
DNA methylation	BP
DNA methylation	BP
single-stranded te	MF
dolichyl monopho	BP

otic placode form	BP
meiotic metapha:	BP
extracellular stru	BP
intercellular bridg	BP
regulation of prog	BP
positive regulatio	BP
negative regulatic	BP
synaptic cleft	CC
penile erection	BP
positive regulatio	BP
negative regulatic	BP
regulation of GTP	BP
pyrimidine nuclec	BP
hypoxanthine sal	BP
negative regulatic	BP
replication fork a	BP
receptor metabol	BP
receptor clusterir	BP
regulation of vasc	BP
negative regulatic	BP
positive regulatio	BP
tumor necrosis fa	MF
neurotrophin bin	MF
regulation of I-ka	BP
positive regulatio	BP
ErbB-3 class rece	MF
positive regulatio	BP
surfactant homec	BP
ubiquitin binding	MF
glycerol-3-phospl	MF
DNA replication,	BP
3'-5' DNA helicase	MF
5'-3' DNA helicase	MF
stress fiber assen	BP
induction of bact	BP
entrainment of ci	BP
acrosomal matrix	CC
ubiquitin-depend	BP
cell envelope org	BP
ion binding	MF
cation binding	MF
macromolecule n	BP
peptide catabolic	BP
RNA polymerase	MF
organic acid bindi	MF

alcohol binding	MF
rhythmic excitatio	BP
vacuolar sequeste	BP
vascular endothe	MF
vascular endothe	MF
vascular endothe	MF
P granule	CC
H4/H2A histone a	CC
ATP-binding casse	CC
axon initial segme	CC
terminal bouton	CC
varicosity	CC
dendritic spine	CC
dendritic shaft	CC
sulfate binding	MF
response to amin	BP
response to leuci	BP
lysosomal lumen	CC
axon hillock	CC
perikaryon	CC
glycosphingolipid	MF
myelin sheath	CC
myelin maintenar	BP
compact myelin	CC
lateral loop	CC
Schmidt-Lanterm	CC
SMC family prote	MF
membrane-bound	CC
intracellular orga	CC
receptor complex	CC
laminin binding	MF
laminin-1 binding	MF
Fanconi anaemia	CC
negative regulatio	BP
positive regulatio	BP
regulation of prot	BP
telomere mainter	BP
proteasome asser	BP
erythrocyte matu	BP
sodium-depender	MF
sodium-depender	BP
sodium-independ	BP
regulation of prot	BP
laminin complex	CC
laminin-8 comple	CC

laminin-10 compl	CC
laminin-11 compl	CC
adenosine-diphos	MF
ectoplasm	CC
regulation of pota	BP
negative regulatio	BP
positive regulatio	BP
regulation of ion	BP
positive regulatio	BP
negative regulatio	BP
CTPase activity	MF
phospholipase bi	MF
anoikis	BP
apoptotic cell clea	BP
response to morp	BP
response to alkalo	BP
positive regulatio	BP
regulation of cyst	BP
RAVE complex	CC
contractile fiber	CC
apoptosome	CC
glutathione bindi	MF
apical junction co	CC
apical junction as	BP
leukocyte degran	BP
mast cell degranu	BP
regulation of mas	BP
negative regulatio	BP
positive regulatio	BP
eosinophil degran	BP
positive regulatio	BP
neutrophil degran	BP
regulation of neu	BP
positive regulatio	BP
cytotoxic T cell de	BP
negative regulatio	BP
natural killer cell	BP
negative regulatio	BP
positive regulatio	BP
phosphatidylinosi	MF
protein transport	BP
response to dsRN	BP
2-octaprenyl-6-m	MF
protein unfolding	BP
CDP-diacylglycero	MF

enucleate erythrc	BP
beta selection	BP
positive T cell sel	BP
CD4-positive or C	BP
regulation of CD4	BP
negative regulatic	BP
positive regulatio	BP
CD4-positive, alpl	BP
CD8-positive, alpl	BP
CD8-positive, alpl	BP
regulation of CD8	BP
negative regulatic	BP
positive regulatio	BP
memory T cell dif	BP
regulation of mer	BP
negative regulatic	BP
positive regulatio	BP
negative T cell sel	BP
positive regulatio	BP
negative regulatic	BP
regulation of prot	BP
proteoglycan binc	MF
heparan sulfate p	MF
HLH domain bind	MF
cortisol secretion	BP
steroid hormone	BP
glucocorticoid me	BP
skeletal muscle ti	BP
regulation of MAI	BP
positive regulatio	BP
negative regulatic	BP
regulation of MAI	BP
negative regulatic	BP
positive regulatio	BP
macromolecule g	BP
positive regulatio	BP
regulation of skel	BP
negative regulatic	BP
homocysteine cat	BP
anthranilate meta	BP
protein kinase B k	MF
3-phosphoinositic	MF
bHLH transcriptio	MF
MRF binding	MF
2-decaprenyl-6-m	MF

negative regulati	BP
acetoacetic acid r	BP
regulation of pen	BP
regulation of cellu	BP
proton-transporti	BP
regulation of ATP	BP
regulation of cellu	BP
pigmentation	BP
pigment metabol	BP
cellular pigment a	BP
regulation of RNA	BP
histone exchange	BP
regulation of mRI	BP
RNA stabilization	BP
malate-aspartate	BP
protein kinase B s	BP
protein-membrar	MF
skeletal muscle fi	BP
mitochondrial DN	BP
regulation of JUN	BP
positive regulatio	BP
negative regulati	BP
activin A complex	CC
inhibin A comple	CC
inhibin B comple	CC
interleukin-12 co	CC
kinetochore bindi	MF
regulation of DNA	BP
positive regulatio	BP
negative regulati	BP
regulation of myo	BP
leucine zipper do	MF
regulation of neu	BP
negative regulati	BP
positive regulatio	BP
tRNA methyltrans	CC
GET complex	CC
adenosine 5'-mor	MF
ADP binding	MF
angiostatin bindir	MF
inositol 1,3,4,5 te	MF
blood vessel endo	BP
regulation of bloo	BP
positive regulatio	BP
negative regulati	BP

regulation of acti	BP
protein serine/th	MF
6-phosphofructo-	CC
UDP-N-acetylgluc	CC
endothelial cell m	BP
protein acylation	BP
lipoamide binding	MF
molybdopterin co	BP
molybdopterin co	MF
positive regulatio	BP
phosphatidylinosi	MF
regulation of kina	BP
regulation of lipic	BP
regulation of pho	BP
positive regulatio	BP
negative regulatio	BP
regulation of tran	BP
regulation of tran	BP
insulin binding	MF
insulin receptor s	MF
Ku70:Ku80 compl	CC
regulation of insu	BP
positive regulatio	BP
negative regulatio	BP
maintenance of D	BP
regulation of resp	BP
ear development	BP
nose developmer	BP
nose morphogene	BP
tongue developm	BP
tongue morphoge	BP
skin development	BP
skin morphogene	BP
nuclear replicatio	CC
nitrate catabolic p	BP
cellular amide me	BP
cellular amide cat	BP
regulation of carb	BP
multi-eIF complex	CC
astrocyte cell mig	BP
keratinocyte prol	BP
regulation of tran	BP
regulation of tran	BP
regulation of DNA	BP
cortical microtub	BP

cellular protein co	BP
delta DNA polyme	CC
PCNA complex	CC
response to estro	BP
ncRNA polyadeny	BP
ncRNA polyadeny	BP
RNA polyadenylat	BP
inositol phosphat	BP
dicarboxylic acid	BP
dicarboxylic acid	BP
linoleic acid meta	BP
engulfment of ap	BP
mitochondrial fra	BP
recognition of ap	BP
regulation of pho	BP
axon terminus	CC
copper transmem	MF
co-translational p	BP
post-translationa	BP
reverse cholester	BP
cell dedifferentiat	BP
2,3-diketo-5-metl	MF
2-hydroxy-3-keto	MF
5-amino-4-imidaz	MF
DNA-N1-methyla	MF
G/U mismatch-sp	MF
dihydrolipoyllysin	MF
branched-chain a	MF
oxidoreductase a	MF
glyceraldehyde o	MF
phosphatidylinosi	MF
phosphatidylinosi	MF
ADP-specific gluc	MF
Ras palmitoyltran	MF
cyclic nucleotide-	MF
acireductone syn	MF
glyceraldehyde-3-	MF
N-acetylgalactosa	MF
NADPH:sulfur oxi	MF
L-seryl-tRNA(Sec)	MF
DNA-7-methylgua	MF
modulation by ho	BP
negative regulatio	BP
positive regulatio	BP
suramin binding	MF

exonucleolytic ca	BP
ossification involv	BP
ossification involv	BP
protein-containin	BP
positive regulatio	BP
regulation of cAN	BP
positive regulatio	BP
negative regulatic	BP
3-hydroxypropior	MF
histone H3 acetyl	BP
histone H4 acetyl	BP
histone H2A acety	BP
histone H2B acety	BP
histone H3-K9 ace	BP
histone H3-K23 ac	BP
histone H3-K4 ace	BP
histone H2A-K5 a	BP
histone H2B-K5 a	BP
histone H2B-K12	BP
histone H4-K5 ace	BP
histone H4-K8 ace	BP
histone H4-K12 ac	BP
histone H4-K16 ac	BP
histone H4-R3 me	BP
histone H3-S10 pl	BP
histone H3-S28 pl	BP
histone H2A-S1 p	BP
histone acetyltrar	MF
histone acetyltrar	MF
histone acetyltrar	MF
histone acetyltrar	MF
H2A histone acety	MF
modulation by sy	BP
H2B histone acety	MF
histone methyltra	MF
hypermethylatior	BP
hypomethylation	BP
regulation of DNA	BP
translocation of p	BP
regulation of dige	BP
regulation of excr	BP
regulation of resp	BP
regulation of anic	BP
regulation of vacu	BP
positive regulatio	BP

membrane bioge	BP
positive regulatio	BP
L-xylulose reducta	MF
development invo	BP
negative regulatio	BP
histone H3-K14 a	BP
protein folding ch	MF
cytolytic granule	CC
nucleoplasmic ret	CC
'de novo' UMP bi	BP
UMP salvage	BP
translation initiat	CC
'de novo' AMP bi	BP
AMP salvage	BP
'de novo' CTP bio	BP
CTP salvage	BP
spanning compor	CC
other organism c	CC
other organism c	CC
juxtaparanode re	CC
apical pole of neu	CC
host cell surface	CC
organelle membr	CC
mitochondria-ass	CC
cellular metabolic	BP
lipid digestion	BP
cellular lipid catal	BP
polysaccharide di	BP
cellular biosynthe	BP
cellular lipid meta	BP
cellular protein ca	BP
intestinal lipid cat	BP
cellular macromo	BP
cellular carbohyd	BP
cellular macromo	BP
cellular protein m	BP
cellular carbohyd	BP
mitochondrial cris	CC
cell-cell contact z	CC
dendrite terminus	CC
dendritic growth	CC
axonal growth co	CC
cell body	CC
cell body membra	CC
C-fiber	CC

cerebellar mossy	CC
climbing fiber	CC
dentate gyrus mo	CC
main axon	CC
calyx of Held	CC
neuron projection	CC
dendritic branch	CC
axonal spine	CC
neuron spine	CC
protein K6-linked	BP
protein K27-linke	BP
cone cell pedicle	CC
wound healing, sp	BP
cellular response	BP
response to leptin	BP
endoplasmic retic	CC
retinoic acid-resp	MF
ion channel bindi	MF
dendritic spine ne	CC
canonical Wnt sig	BP
canonical Wnt sig	BP
canonical Wnt sig	BP
cell-cell adhesion	BP
canonical Wnt sig	BP
canonical Wnt sig	BP
canonical Wnt sig	BP
canonical Wnt sig	BP
canonical Wnt sig	BP
canonical Wnt sig	BP
sodium-depender	BP
type B pancreatic	BP
cellular response	BP
stromal-epithelia	BP
fibroblast apopto	BP
macropinocytosis	BP
macropinosome	CC
clearance of forei	BP
regulation of rRN	BP
regulation of pero	BP
RNA polymerase	MF
non-sequence-sp	MF
protein localizatio	BP
glucose import in	BP
negative regulatio	BP
small protein acti	MF

ubiquitin-like pro	MF
microspike	CC
adhesion of symb	BP
entry into host	BP
motile cilium asse	BP
glutaryl-CoA hydr	MF
protein sulfhydra	BP
regulation of mit	BP
supraspliceosoma	CC
long-chain fatty a	BP
L-cystine L-cystei	MF
NSL complex	CC
NLRP3 inflammas	BP
DNA topoisomera	MF
S100 protein binc	MF
GTP cyclohydrola	MF
relaxation of smo	BP
dendritic cell prol	BP
[2Fe-2S] cluster a	BP
butyryl-CoA catal	BP
17-beta-hydroxys	MF
decaprenyldihydr	MF
3-demethylubiqu	MF
daunorubicin met	BP
doxorubicin meta	BP
AP-5 adaptor com	CC
protein deadenyl	BP
protein adenylylh	MF
DBIRD complex	CC
FMN transmemb	MF
nuclear pore inne	CC
nuclear pore cent	CC
nuclear pore cyto	CC
nuclear pore nucl	CC
histone H3-K4 dir	BP
adhesion of symb	BP
MLL3/4 complex	CC
tooth eruption	BP
8-oxo-dGDP phos	MF
8-oxo-GDP phosp	MF
8-hydroxy-dADP	MF
protein import in	BP
protection of DN	BP
DNA demethylati	BP
hemi-methylated	MF

bone sialoprotein	MF
acid-sensing ion c	MF
response to huma	BP
amphisome	CC
autolysosome	CC
cell cycle phase tr	BP
mitotic cell cycle	BP
mitotic DNA dam	BP
mitotic DNA integ	BP
meiotic DNA integ	BP
meiotic spindle ch	BP
cilium organizatic	BP
G1 DNA damage c	BP
modulation by ho	BP
negative regulatio	BP
positive regulatio	BP
negative regulatio	BP
positive regulatio	BP
trans-Golgi netwo	BP
multi-organism m	BP
autophagy of nuc	BP
late nucleophagy	BP
G-quadruplex DN	BP
mitotic G2/M tra	BP
mitotic G1/S tran	BP
negative regulatio	BP
positive regulatio	BP
modulation by ho	BP
cell cycle G2/M p	BP
cell cycle G1/S ph	BP
estrous cycle	BP
plasma membran	CC
plasma membran	BP
plasma membran	BP
plasma membran	BP
protein localizatic	BP
protein transport	BP
lipoprotein locali	BP
protein-containin	MF
mitotic cytokines	BP
double-strand bre	BP
double-strand bre	BP
DNA replication p	BP
DNA deaminatior	BP
depurination	BP

depyrimidination	BP
actin nucleation	BP
mitochondrial ma	BP
negative regulatio	BP
early endosome t	BP
G0 to G1 transitio	BP
mitochondrial de	CC
plasma membran	BP
DNA end binding	MF
G protein-couple	MF
G protein-couple	MF
G protein-couple	MF
G protein-couple	MF
protein insertion	BP
protein insertion	BP
protein import in	BP
protein import in	BP
protein targeting	BP
protein insertion	BP
protein insertion	BP
protein retention	BP
constitutive secre	BP
regulated exocyto	BP
transcytosis	BP
T cell selection	BP
positive thymic T	BP
negative thymic T	BP
thymic T cell sele	BP
extrathymic T cel	BP
T-helper 1 cell dif	BP
T-helper 2 cell dif	BP
cytotoxic T cell di	BP
regulatory T cell	BP
regulation of vira	BP
positive regulatio	BP
regulation of sing	BP
interleukin-18 rec	CC
keratin filament	CC
type III intermedi	CC
intermediate filar	BP
intermediate filar	BP
intermediate filar	BP
intermediate filar	BP
intermediate filar	BP
integrin biosynth	BP

regulation of inte	BP
protein neddylati	BP
azole transmemb	BP
pronucleus	CC
membrane raft	CC
cellular extravasa	BP
regulation of bon	BP
bioactive lipid rec	MF
N-acetylglucosam	MF
keratan sulfotran	MF
pre-mRNA branch	MF
meiotic chromoso	BP
uridine-diphospha	MF
development of s	BP
development of p	BP
meiotic telomere	BP
triplex DNA bindi	MF
homologous chro	BP
single-stranded D	MF
tripeptide aminop	MF
myosin II binding	MF
neuronal ion chan	BP
clustering of volta	BP
clustering of volta	BP
cell fate commitm	BP
asymmetric prote	BP
intercellular bridg	CC
glutathione dehy	MF
basal protein loca	BP
apical protein loc	BP
apical part of cell	CC
basal part of cell	CC
apical cortex	CC
basal cortex	CC
translation regula	MF
translation factor	MF
establishment of	BP
maintenance of p	BP
zonula adherens a	BP
regulation of circa	BP
regulation of circa	BP
isotype switching	BP
regulation of isot	BP
establishment or	BP
establishment of	BP

maintenance of e	BP
establishment of	BP
synapse	CC
MAPK export fror	BP
MAPK phosphata	BP
FasL biosynthetic	BP
postsynaptic mer	CC
neurotransmitter	BP
sarcomere organi	BP
cell-cell junction (	BP
cell-cell junction r	BP
zonula adherens r	BP
negative regulatic	BP
negative regulatic	BP
extracellular poly	BP
protein palmitole	BP
CXCR1 chemokine	MF
tricarboxylic acid	CC
succinate-CoA lig	CC
oxoglutarate deh	CC
pyruvate dehydrc	CC
proton-transporti	CC
proton-transporti	CC
proton-transporti	CC
respiratory chain	CC
respiratory chain	CC
respiratory chain	CC
respiratory chain	CC
succinate dehydr	CC
mRNA cis splicing	BP
alpha-catenin bin	MF
gamma-catenin b	MF
tubulin complex	CC
protein phosphor	MF
leukocyte activati	BP
unmethylated Cp	MF
late endosome tc	BP
carnitine biosynth	BP
phospholipid trar	BP
cellular respiratio	BP
clathrin-coated er	CC
phagocytic vesicle	CC
farnesyl diphosph	BP
farnesyl diphosph	BP
mercury ion bindi	MF

MHC class II biosy	BP
regulation of MH(	BP
negative regulati(	BP
positive regulatio	BP
negative regulati(	BP
positive regulatio	BP
interleukin-1 type	MF
interleukin-1 type	MF
regulation of nitri	BP
positive regulatio	BP
fat cell differentia	BP
myoblast differer	BP
endothelial cell d	BP
bone resorption	BP
cell redox homeo	BP
response to ethai	BP
response to ethei	BP
locomotor rhythr	BP
photoreceptor ce	BP
pole plasm	CC
chemorepellent a	MF
dynein light chain	MF
dynein heavy cha	MF
dynein intermedi	MF
interleukin-27 rec	MF
interleukin-27 bir	MF
interleukin-18 rec	MF
interleukin-23 rec	MF
negative regulati(	BP
positive regulatio	BP
syndecan binding	MF
dehydrodolichyl (	MF
geranylgeranyl re	MF
TRAIL binding	MF
mast cell activati(	BP
regulation of B ce	BP
negative regulati(	BP
positive regulatio	BP
regulation of T ce	BP
negative regulati(	BP
positive regulatio	BP
negative regulati(	BP
positive regulatio	BP
regulation of gan	BP
positive regulatio	BP

regulation of regu	BP
negative regulatio	BP
positive regulatio	BP
regulation of cell	BP
negative regulatio	BP
regulation of fat c	BP
negative regulatio	BP
positive regulatio	BP
regulation of end	BP
negative regulatio	BP
positive regulatio	BP
regulation of epic	BP
negative regulatio	BP
positive regulatio	BP
regulation of inne	BP
negative regulatio	BP
regulation of kera	BP
negative regulatio	BP
positive regulatio	BP
regulation of lym	BP
negative regulatio	BP
positive regulatio	BP
regulation of T-he	BP
negative regulatio	BP
positive regulatio	BP
negative regulatio	BP
positive regulatio	BP
negative regulatio	BP
positive regulatio	BP
regulation of mel	BP
positive regulatio	BP
regulation of mye	BP
negative regulatio	BP
positive regulatio	BP
positive regulatio	BP
regulation of eryt	BP
negative regulatio	BP
negative regulatio	BP
positive regulatio	BP
regulation of meg	BP
negative regulatio	BP
positive regulatio	BP
negative regulatio	BP
positive regulatio	BP
negative regulatio	BP

positive regulatio	BP
regulation of myc	BP
positive regulatio	BP
regulation of neu	BP
negative regulatio	BP
positive regulatio	BP
regulation of oste	BP
negative regulatio	BP
positive regulatio	BP
regulation of oste	BP
negative regulatio	BP
positive regulatio	BP
regulation of epic	BP
positive regulatio	BP
regulation of glial	BP
negative regulatio	BP
positive regulatio	BP
ketoreductase ac	MF
negative regulatio	BP
negative regulatio	BP
negative regulatio	BP
negative regulatio	BP
positive regulatio	BP
positive regulatio	BP
positive regulatio	BP
positive regulatio	BP
positive regulatio	BP
positive regulatio	BP
respiratory burst	BP
respiratory burst	BP
positive regulatio	BP
negative regulatio	BP
positive regulatio	BP
negative regulatio	BP
positive regulatio	BP
positive regulatio	BP
positive regulatio	BP
positive regulatio	BP
positive regulatio	BP
negative regulatio	BP
positive regulatio	BP
negative regulatio	BP
positive regulatio	BP
negative regulatio	BP
negative regulatio	BP

positive regulatio	BP
regulation of ade	BP
positive regulatio	BP
negative regulatio	BP
positive regulatio	BP
regulation of angi	BP
positive regulatio	BP
negative regulatio	BP
positive regulatio	BP
negative regulatio	BP
positive regulatio	BP
positive regulatio	BP
negative regulatio	BP
positive regulatio	BP
positive regulatio	BP
negative regulatio	BP
positive regulatio	BP
negative regulatio	BP
positive regulatio	BP
negative regulatio	BP
positive regulatio	BP
negative regulatio	BP
positive regulatio	BP
positive regulatio	BP
negative regulatio	BP
positive regulatio	BP
positive regulatio	BP
negative regulatio	BP
positive regulatio	BP
positive regulatio	BP
negative regulatio	BP
positive regulatio	BP
negative regulatio	BP
positive regulatio	BP
negative regulatio	BP
positive regulatio	BP
negative regulatio	BP
positive regulatio	BP
negative regulatio	BP
positive regulatio	BP
negative regulatio	BP
positive regulatio	BP
negative regulatio	BP
positive regulatio	BP
negative regulatio	BP

positive regulatio	BP
negative regulatio	BP
positive regulatio	BP
pH reduction	BP
pH elevation	BP
regulation of prot	BP
positive regulatio	BP
negative regulatio	BP
positive regulatio	BP
negative regulatio	BP
positive regulatio	BP
negative regulatio	BP
positive regulatio	BP
negative regulatio	BP
positive regulatio	BP
positive regulatio	BP
negative regulatio	BP
regulation of RNA	BP
positive regulatio	BP
negative regulatio	BP
positive regulatio	BP
positive regulatio	BP
positive regulatio	BP
negative regulatio	BP
positive regulatio	BP
negative regulatio	BP
positive regulatio	BP
negative regulatio	BP
positive regulatio	BP
negative regulatio	BP
negative regulatio	BP
positive regulatio	BP
negative regulatio	BP
positive regulatio	BP
regulation of fem	BP
positive regulatio	BP
negative regulatio	BP
positive regulatio	BP
negative regulatio	BP
positive regulatio	BP
negative regulatio	BP
positive regulatio	BP
positive regulatio	BP
negative regulatio	BP
positive regulatio	BP

positive regulatio	BP
positive regulatio	BP
negative regulatio	BP
positive regulatio	BP
negative regulatio	BP
positive regulatio	BP
negative regulatio	BP
positive regulatio	BP
negative regulatio	BP
positive regulatio	BP
negative regulatio	BP
negative regulatio	BP
negative regulatio	BP
positive regulatio	BP
negative regulatio	BP
positive regulatio	BP
negative regulatio	BP
positive regulatio	BP
negative regulatio	BP
negative regulatio	BP
positive regulatio	BP
negative regulatio	BP
positive regulatio	BP
carbon catabolite	BP
negative regulatio	BP
negative regulatio	BP
positive regulatio	BP
regulation of emb	BP
regulation of acti	BP
negative regulatio	BP
positive regulatio	BP
regulation of T ce	BP
regulation of tran	BP
positive regulatio	BP
inositol trisphosp	MF
ADP metabolic pr	BP
ADP catabolic pro	BP
AMP metabolic p	BP
ATP metabolic pr	BP
CTP metabolic pr	BP
GMP metabolic p	BP
GMP catabolic pr	BP
GTP metabolic pr	BP
IMP metabolic pr	BP
ITP metabolic pro	BP

UMP catabolic pr	BP
UTP metabolic pr	BP
UTP catabolic pro	BP
dGMP metabolic	BP
dGMP catabolic p	BP
dADP catabolic pr	BP
cAMP metabolic	BP
dAMP catabolic p	BP
dATP metabolic p	BP
dATP catabolic pr	BP
dGDP catabolic pr	BP
cGMP metabolic	BP
cGMP catabolic p	BP
dGTP metabolic p	BP
dTMP catabolic p	BP
dUMP catabolic p	BP
dUTP catabolic pr	BP
adenine metaboli	BP
adenine biosynth	BP
adenosine metab	BP
adenosine biosyn	BP
deoxycytidine me	BP
guanine metaboli	BP
hypoxanthine me	BP
hypoxanthine bio	BP
inosine biosynthe	BP
thymidine metab	BP
thymidine biosyn	BP
uridine metabolic	BP
xanthine biosynth	BP
deoxyribonucleos	BP
purine deoxyribo	BP
purine ribonucleo	BP
pyrimidine nucleo	BP
pyrimidine nucleo	BP
tetrahydrobiopte	BP
alcohol catabolic	BP
glyceraldehyde-3-	BP
glycerol-3-phosph	BP
glycerol-3-phosph	BP
D-gluconate catal	BP
aldehyde catabol	BP
spermidine catab	BP
nor-spermidine m	BP
spermine catabol	BP

nitric oxide metal	BP
nitric oxide catab	BP
indolalkylamine b	BP
aflatoxin metabol	BP
aflatoxin cataboli	BP
coumarin catabol	BP
stilbene catabolic	BP
formaldehyde bic	BP
formaldehyde cat	BP
glycolate biosynt	BP
phosphocreatine	BP
gluconokinase ac	MF
negative regulati	BP
regulation of fatt	BP
positive regulatio	BP
negative regulati	BP
glucose import	BP
regulation of gluc	BP
negative regulati	BP
positive regulatio	BP
glycerol biosynth	BP
regulation of JNK	BP
negative regulati	BP
positive regulatio	BP
lateral inhibition	BP
SMAD binding	MF
phosphatidyletha	BP
phosphatidyletha	BP
diacylglycerol me	BP
diacylglycerol cat	BP
CDP-diacylglycerc	BP
acetyl-CoA catabc	BP
butyrate cataboli	BP
2-oxobutyrate bic	BP
GDP-L-fucose me	BP
fructose biosynth	BP
L-arabinose meta	BP
N-acetylneuramir	BP
deoxyribose phos	BP
ribose phosphate	BP
carboxylic acid ca	BP
UDP-glucuronate	BP
polynucleotide 3'	MF
ATP-dependent p	MF
urate metabolic p	BP

D-amino acid met	BP
regulation of rece	BP
negative regulatio	BP
positive regulatio	BP
organophosphate	BP
creatinine metab	BP
dihydrofolate me	BP
icosanoid biosynt	BP
hexadecanal met	BP
short-chain fatty	BP
acylglycerol catab	BP
membrane lipid c	BP
platelet activating	BP
phosphatidylchol	BP
phosphatidylglyce	BP
phosphatidic acid	BP
glycerophospholi	BP
glycerophospholi	BP
glycosylceramide	BP
glycosphingolipid	BP
heterocycle meta	BP
ether lipid metab	BP
glycerolipid meta	BP
glyoxylate metab	BP
phosphatidylinosi	BP
L-methylmalonyl-	BP
nicotinamide nuc	BP
S-adenosylhomoc	BP
S-adenosylmethio	BP
protoporphyrinog	BP
sphinganine biosy	BP
sphingosine biosy	BP
ceramide biosynt	BP
ceramide catabol	BP
sphingoid biosynt	BP
sphingoid catabol	BP
S-methyl-5-thiori	MF
xylosylprotein 4-b	MF
D-xylulose reduct	MF
glucosyltransfera	MF
negative regulatio	BP
2,3-bisphosphogly	MF
histamine N-meth	MF
U4/U6 x U5 tri-sn	CC
saliva secretion	BP

development of s	BP
development of s	BP
development of p	BP
development of p	BP
retinal rod cell de	BP
retinal cone cell c	BP
malate dehydrog	MF
alpha-L-arabinofu	MF
methylthioribulos	MF
long-chain-alcohc	MF
regulation of Ras	BP
positive regulatio	BP
negative regulatic	BP
intercellular cana	CC
positive regulatio	BP
negative regulatic	BP
polyamine oxidas	MF
regulation of vira	BP
negative regulatic	BP
positive regulatio	BP
regulation of cent	BP
negative regulatic	BP
positive regulatio	BP
regulation of mitc	BP
positive regulatio	BP
regulation of cent	BP
lysosomal proton	CC
drug export	BP
optic placode for	BP
regulation of orga	BP
negative regulatic	BP
positive regulatio	BP
sphingolipid flopp	MF
sphingolipid trans	MF
sphingolipid bindi	MF
regulation of insu	BP
negative regulatic	BP
positive regulatio	BP
gamma-delta T ce	BP
alpha-beta T cell	BP
alpha-beta T cell	BP
alpha-beta T cell	BP
positive regulatio	BP
negative regulatic	BP
positive regulatio	BP

negative regulati	BP
positive regulatio	BP
negative regulati	BP
positive regulatio	BP
lymphocyte activ	BP
lymphocyte proli	BP
tetrahydrofolate	BP
tetrahydrofolate	BP
folic acid metabo	BP
folic acid cataboli	BP
anchored compo	CC
male sex differen	BP
retinal cell progra	BP
regulation of reti	BP
negative regulati	BP
negative regulati	BP
response to antib	BP
response to DDT	BP
response to orga	BP
response to pyre	BP
response to arser	BP
response to cadm	BP
response to copp	BP
response to merc	BP
response to tellu	BP
intracellular cana	CC
SLIK (SAGA-like) c	CC
lipopolysaccharid	CC
decidualization	BP
natural killer cell	MF
CDP biosynthetic	BP
IDP catabolic pro	BP
GDP metabolic pr	BP
GDP biosynthetic	BP
GDP catabolic pro	BP
borate transport	BP
active borate tra	MF
muscle cell cellul	BP
acid secretion	BP
viral entry into ho	BP
lactic acid secreti	BP
oxalic acid secreti	BP
positive regulatio	BP
viral capsid secon	BP
viral exocytosis	BP

viral budding	BP
protein autophos	BP
modification by v	BP
regulation of vira	BP
viral mRNA expor	BP
microtubule poly	BP
viral replication c	BP
host cell surface r	MF
virion binding	MF
histone deacetyla	MF
receptor-mediate	BP
coreceptor-media	BP
chemokine recep	MF
positive regulatio	BP
negative regulatic	BP
positive regulatio	BP
regulation of RNA	BP
negative regulatic	BP
positive regulatio	BP
lipid phosphoryla	BP
carbohydrate pho	BP
glycolipid transpo	BP
phosphorylated c	BP
phospholipid dep	BP
filopodium assem	BP
hydroxyapatite bi	MF
bone remodeling	BP
regulation of bon	BP
negative regulatic	BP
phosphatidylinosi	BP
inositol phosphat	BP
phosphatidylinosi	BP
terpenoid transpo	BP
cadmium ion binc	MF
metal ion transm	MF
quinolinate meta	BP
ephrin receptor b	MF
regulation of saliv	BP
positive regulatio	BP
hormone secretic	BP
regulation of folli	BP
positive regulatio	BP
negative regulatic	BP
regulation of horr	BP
follicle-stimulatin	BP

regulation of horr	BP
positive regulatio	BP
positive regulatio	BP
negative regulatio	BP
positive regulatio	BP
regulation of lipic	BP
response to cyclo	BP
nucleoside tripho	MF
tetrahydrofolylpc	BP
tetrahydrofolylpc	BP
regulation of mitc	BP
secretion	BP
tetrapyrrole bindi	MF
intracellular trans	BP
metal chelating a	MF
transferase activi	MF
transition metal i	MF
transition metal i	MF
alpha-(1->3)-fuco	MF
alpha-(1->6)-fuco	MF
peptide-O-fucosy	MF
ER retention sequ	MF
regulation of neu	BP
negative regulatio	BP
pore complex	CC
pore complex ass	BP
proton-transporti	MF
phosphatidylinosi	MF
1-phosphatidylinc	MF
nucleotide phosp	BP
carboxylic acid tra	MF
hydroxylysine bio	BP
cellular ketone bc	BP
ketone body bios	BP
ketone body cata	BP
nonassociative le	BP
habituation	BP
sensitization	BP
proton-transporti	MF
3'-phosphoadeno	BP
3'-phosphoadeno	MF
retinoid X recepto	MF
cytosol to endopl	BP
peptide antigen t	BP
NAD-dependent I	MF

NAD-dependent h	MF
histone acetyltrar	MF
histone methyltra	MF
histone methyltra	MF
TAP binding	MF
TAP1 binding	MF
TAP2 binding	MF
tapasin binding	MF
protein heterodir	MF
protein dimerizat	MF
regulation of hem	BP
positive regulatio	BP
negative regulatic	BP
17-alpha,20-alpha	MF
sterol-4-alpha-car	MF
3-hydroxy-2-meth	MF
cholest-5-ene-3-b	MF
prostaglandin-F s	MF
15-hydroxyprosta	MF
15-hydroxyprosta	MF
androsterone deh	MF
5alpha-androstan	MF
3-oxoacyl-[acyl-ca	MF
testosterone deh	MF
androsterone deh	MF
androstan-3-alph	MF
testosterone 17-b	MF
vitamin-K-epoxide	MF
phospholipid-hyd	MF
ketosteroid mono	MF
27-hydroxychole s	MF
aminomuconate-	MF
4-trimethylammo	MF
trans-1,2-dihydro	MF
enoyl-[acyl-carrie	MF
thiomorpholine-c	MF
saccharopine deh	MF
saccharopine deh	MF
protein-disulfide	MF
betaine-homocys	MF
1-alkenylglycerop	MF
1-alkenylglycerop	MF
phosphatidylchol	MF
platelet-activatin	MF
1-acylglycerophos	MF

N-acetylneuramir	MF
1-alkylglycerophc	MF
1-alkylglycerophc	MF
galactosylxylosylp	MF
beta-1,3-galactos	MF
acetylgalactosam	MF
acetylgalactosam	MF
glucuronylgalactc	MF
glucuronosyl-N-a	MF
lactosylceramide	MF
N-acylsphingosine	MF
galactosylgalacto	MF
glucosaminylgala	MF
globoside alpha-N	MF
nicotinamide pho	MF
monosialoganglic	MF
(alpha-N-acetylne	MF
lactosylceramide	MF
4-hydroxybenzoa	MF
(S)-3-amino-2-me	MF
kynurenine-glyox	MF
glutamine-phenyl	MF
[hydroxymethylgl	MF
[3-methyl-2-oxob	MF
inositol tetrakispl	MF
inositol tetrakispl	MF
fucose-1-phospha	MF
D-ribitol-5-phosp	MF
succinate-hydrox	MF
acylglycerol lipase	MF
methylumbellifer	MF
all-trans-retinyl-p	MF
dodecanoyl-[acyl-	MF
glycerophosphocl	MF
glycerophosphocl	MF
alkylglycerophosp	MF
glycerophosphoir	MF
glycerophosphoir	MF
protein-glucosylg	MF
alkenylglyceroph	MF
alkenylglyceroph	MF
N-(long-chain-acy	MF
N-acetylgalactosa	MF
nucleoside-tripho	MF
17-alpha-hydroxy	MF

N-acylneuramina	MF
3-hydroxyoctano	MF
ATP-dependent N	MF
heparosan-N-sulf	MF
regulation of resp	BP
protein N-termin	MF
ceramide choline	MF
vesicle transport	BP
mitochondrion tr	BP
calcium-depende	MF
calcium-indepenc	MF
15-oxoprostaglan	MF
2-aminoadipate t	MF
2-hydroxyglutara	MF
3',5'-cyclic-GMP p	MF
3-dehydrosphinga	MF
acetoacetyl-CoA l	MF
acetylputrescine	MF
acetylspermidine	MF
acyl-CoA hydrolas	MF
acylglycerol kinas	MF
acylpyruvate hyd	MF
ADP-ribose dipho	MF
allyl-alcohol dehy	MF
arachidonate-CoA	MF
arachidonate 8(R	MF
amine sulfotransf	MF
aspartyltransfera	MF
ATP diphosphatas	MF
beta-adrenergic r	MF
bile-salt sulfotran	MF
biotinidase activi	MF
bis(5'-adenosyl)-t	MF
indanol dehydrog	MF
iron-cytochrome-	MF
carnosine synthas	MF
CDP-glycerol diph	MF
chlordecone redu	MF
cholate-CoA ligas	MF
cholestanetetrao	MF
cholestanetriol 26	MF
cholestenol delta	MF
cholestenone 5-a	MF
chondroitin 4-sul	MF
chondroitin-glucu	MF

butyrate-CoA liga	MF
(S)-citramalyl-CoA	MF
citrate dehydrata	MF
delta4-3-oxoster	MF
cysteamine dioxy	MF
L-cysteine:2-oxog	MF
cysteine-S-conjug	MF
cytidylate cyclase	MF
D-glutamate cycla	MF
D-threo-aldose 1-	MF
D-xylose 1-dehyd	MF
dCTP diphosphata	MF
deoxycytidine dea	MF
deoxyuridine pho	MF
diiodophenylpyru	MF
dimethylglycine d	MF
dolichyldiphospha	MF
farnesol dehydrog	MF
fatty acid peroxid	MF
flavonol 3-sulfotr	MF
ganglioside galact	MF
GDP-mannose 3,5	MF
glucosamine kina	MF
glucose-1,6-bisph	MF
glucose 1-dehydr	MF
glucose 1-dehydr	MF
glucose 1-dehydr	MF
L-glucuronate rec	MF
glucuronolactone	MF
glutamine N-acylt	MF
glycerol dehydrog	MF
glycine N-acyltran	MF
glycine N-choloylt	MF
guanosine phospl	MF
hepoxilin-epoxide	MF
hydroxyacid-oxoa	MF
hydroxylysine kin	MF
hydroxymethylglu	MF
antigen processin	BP
antigen processin	BP
antigen processin	BP
platelet-derived g	BP
insulin-like growt	BP
vascular endothe	BP
neurotrophin TRK	BP

hepatocyte growt	BP
ephrin receptor s	BP
Tie signaling path	BP
phosphatidylinosi	BP
inositol phosphat	BP
inositol lipid-med	BP
receptor ligand ac	MF
receptor antagon	MF
CCR chemokine re	MF
regulation of mel	BP
negative regulatic	BP
positive regulatio	BP
regulation of mRI	BP
negative regulatic	BP
positive regulatio	BP
mRNA 5'-UTR bin	MF
monosaccharide l	MF
disaccharide bind	MF
heme O biosynth	BP
quinone binding	MF
ubiquinone bindi	MF
UDP-glucuronate	MF
focal adhesion as	BP
embryonic eye m	BP
post-embryonic e	BP
developmental pi	BP
eye pigmentation	BP
regulation of dev	BP
regulation of eye	BP
negative regulatic	BP
positive regulatio	BP
positive regulatio	BP
chromatin-media	BP
calcium- and caln	MF
autophagic cell de	BP
somatic stem cell	BP
establishment of	BP
female germ-line	BP
male germ-line st	BP
spermatocyte div	BP
astrocyte activati	BP
fibroblast prolifer	BP
regulation of fibro	BP
positive regulatio	BP
negative regulatic	BP

behavioral respor	BP
behavioral respor	BP
tau protein bindir	MF
primary follicle st	BP
regulation of syna	BP
regulation of neu	BP
regulation of long	BP
positive regulatio	BP
regulation of shor	BP
activin receptor c	CC
activin binding	MF
Set1C/COMPASS	CC
Golgi vesicle trans	BP
Golgi vesicle budc	BP
vesicle targeting,	BP
vesicle targeting,	BP
COPI coating of G	BP
COPII vesicle coat	BP
regulation of vesi	BP
Golgi vesicle fusic	BP
Golgi vesicle dock	BP
Golgi vesicle prefu	BP
inter-Golgi cistern	BP
plasma membran	BP
male gamete gen	BP
rough endoplasm	CC
negative regulatic	BP
sperm capacitatic	BP
epinephrine trans	BP
epinephrine secre	BP
norepinephrine se	BP
phytanoyl-CoA di	MF
eosinophil chemo	BP
macrophage cher	BP
lymphocyte chem	BP
iron import into t	BP
elastic fiber asser	BP
lauric acid metab	BP
snoRNA localizati	BP
mRNA stabilizatic	BP
flap endonucleas	MF
3'-flap endonucle	MF
regulation of rece	BP
positive regulatio	BP
negative regulatic	BP

determination of	BP
determination of	BP
response to pain	BP
behavioral respor	BP
clathrin coat asse	BP
methionine aden	CC
methionine aden	MF
mitogen-activate	MF
vesicle docking	BP
vesicle fusion wit	BP
organelle fusion	BP
lung alveolus dev	BP
isotype switching	BP
isotype switching	BP
negative regulati	BP
positive regulatio	BP
negative regulati	BP
positive regulatio	BP
regulation of isot	BP
positive regulatio	BP
calcium-depende	MF
mitochondrion di	BP
intracellular distri	BP
axial mesoderm c	BP
axial mesoderm r	BP
axial mesoderm f	BP
mesoderm morpl	BP
mesodermal cell	BP
positive regulatio	BP
paraxial mesoder	BP
paraxial mesoder	BP
paraxial mesoder	BP
paraxial mesoder	BP
paraxial mesoder	BP
lateral mesoderm	BP
regulation of late	BP
mesendoderm de	BP
retinoic acid rece	BP
regulation of reti	BP
positive regulatio	BP
negative regulati	BP
endosomal lumer	BP
intermediate me:	BP
intermediate me:	BP
brain-derived neu	MF

nerve growth fac	MF
platelet-derived g	MF
epidermal growth	MF
cell development	BP
cell maturation	BP
Holliday junction	CC
oogenesis	BP
replication fork p	BP
enteric nervous s	BP
sympathetic nerv	BP
beta-tubulin bind	MF
synaptic vesicle e	BP
synaptic vesicle t	BP
anterograde syna	BP
Roundabout bind	MF
maintenance of a	BP
synaptic vesicle n	BP
signal recognition	CC
regulation of timi	BP
rhythmic process	BP
circadian behavio	BP
animal organ dev	BP
blood vessel mor	BP
spermatid differe	BP
positive regulatio	BP
negative regulatio	BP
positive regulatio	BP
negative regulatio	BP
hematopoietic or	BP
lymph node deve	BP
spleen developm	BP
thymus developm	BP
bone marrow dev	BP
Peyer's patch dev	BP
response to stero	BP
digestive tract mo	BP
positive regulatio	BP
negative regulatio	BP
embryonic digest	BP
establishment of	BP
establishment of	BP
embryonic organ	BP
post-embryonic a	BP
digestive tract de	BP
embryonic digest	BP

embryonic organ	BP
post-embryonic a	BP
notochord morph	BP
developmental ce	BP
developmental gr	BP
eye morphogene:	BP
camera-type eye	BP
embryonic camer	BP
post-embryonic c	BP
embryonic morph	BP
oocyte developm	BP
reproductive stru	BP
multicellular orga	BP
embryonic ectod	BP
embryonic foregu	BP
embryonic hindgu	BP
myoblast fate cor	BP
myoblast develop	BP
skeletal muscle ti	BP
regulation of skel	BP
negative regulatio	BP
positive regulatio	BP
negative regulatio	BP
regulation of dev	BP
positive regulatio	BP
negative regulatio	BP
regulation of skel	BP
negative regulatio	BP
positive regulatio	BP
muscle organ mo	BP
animal organ forr	BP
anatomical struct	BP
smooth muscle ce	BP
regulation of smo	BP
negative regulatio	BP
neuron fate comr	BP
neuron fate dete	BP
neuron fate speci	BP
neuron developm	BP
cell morphogene:	BP
collateral sprouti	BP
collateral sprouti	BP
regulation of colla	BP
negative regulatio	BP
positive regulatio	BP

axon extension	BP
axon extension in	BP
response to axon	BP
regulation of axo	BP
positive regulatio	BP
negative regulatio	BP
negative regulatio	BP
positive regulatio	BP
negative regulatio	BP
positive regulatio	BP
positive regulatio	BP
negative regulatio	BP
generation of neu	BP
embryonic crania	BP
embryonic neuro	BP
embryonic viscer	BP
embryonic skelet	BP
skeletal system m	BP
embryonic skelet	BP
astrocyte differer	BP
oligodendrocyte	BP
regulation of astr	BP
positive regulatio	BP
negative regulatio	BP
regulation of olig	BP
positive regulatio	BP
negative regulatio	BP
tissue morphoger	BP
epidermis morph	BP
system developm	BP
gland developme	BP
sebaceous gland	BP
cardiac muscle tis	BP
cardiac muscle fib	BP
skeletal muscle fi	BP
regulation of skel	BP
positive regulatio	BP
smooth muscle ti	BP
muscle fiber deve	BP
semicircular cana	BP
pigment granule	BP
branching morph	BP
branching morph	BP
pigment granule	BP
mesenchymal cel	BP

sarcomerogenesi	BP
tissue remodeling	BP
presynaptic active	CC
presynaptic active	CC
cytoskeleton of p	CC
cytoskeletal matr	BP
maintenance of p	BP
calcium ion-regul	BP
spontaneous exo	BP
pronephros devel	BP
genitalia develop	BP
female genitalia r	BP
male genitalia mo	BP
neuron projection	BP
dendrite morpho	BP
regulation of den	BP
hair follicle matur	BP
erythrocyte deve	BP
enucleate erythro	BP
inner ear develop	BP
otolith developm	BP
regulation of axo	BP
positive regulatio	BP
negative regulatio	BP
artery morphoge	BP
venous blood ves	BP
axon extension in	BP
hypophysis morp	BP
diencephalon mo	BP
forebrain morpho	BP
brain morphogen	BP
adenohypophysis	BP
anatomical struct	BP
neural nucleus de	BP
cell projection mo	BP
formation of anat	BP
leukemia inhibito	BP
stem cell differen	BP
stem cell develop	BP
stem cell fate spe	BP
cellular developm	BP
cell motility	BP
multicellular orga	BP
homeostasis of nu	BP
homeostasis of nu	BP

host-mediated re	BP
chemical homeos	BP
sensory system d	BP
peripheral nervou	BP
peripheral nervou	BP
itaconyl-CoA hydı	MF
L-fuconate dehyd	MF
L-pipecolate oxid	MF
L-xylulose reducta	MF
lathosterol oxidas	MF
L-leucine:2-oxoglı	MF
leukotriene-B4 2(	MF
long-chain-aldehy	MF
long-chain-fatty-a	MF
m7G(5')pppN dip	MF
malonyl-CoA deca	MF
L-gulonate 3-deh	MF
inositol oxygenas	MF
myosin-light-chai	MF
N-acylglucosamin	MF
N-acylneuramina	MF
NAD(P)+ nucleosi	MF
NADH dehydroge	MF
omega-amidase a	MF
omega-hydroxyde	MF
ornithine(lysine) t	MF
phenylpyruvate ta	MF
phosphatidylinosi	MF
[phosphorylase] p	MF
phytanate-CoA lig	MF
plasmalogen synt	MF
fucokinase activit	MF
plasmanylethano	MF
procollagen galac	MF
progesterone 5-a	MF
propionate-CoA li	MF
prostaglandin-E s	MF
prostaglandin-E2	MF
retinol isomerase	MF
retinol O-fatty-ac	MF
retinyl-palmitate	MF
rhodopsin kinase	MF
ribitol 2-dehydrog	MF
ribosylnicotinami	MF
RNA uridylyltrans	MF

sedoheptulokinas	MF
sphingomyelin ph	MF
sphingosine N-ac	MF
steroid sulfotrans	MF
steryl-beta-gluco	MF
sulfur dioxygenas	MF
tau-protein kinas	MF
thiamin-triphospl	MF
thiosulfate-thiol s	MF
trans-L-3-hydroxy	MF
trans-octaprenylt	MF
trimethyllysine di	MF
triokinase activity	MF
triphosphatase ac	MF
[acetyl-CoA carbc	MF
nitrite reductase	MF
3'-phosphoadeno	BP
3'-phosphoadeno	BP
calcium-depende	MF
transforming grov	MF
positive regulatio	BP
amyloid-beta met	BP
microfibril bindin	MF
ethanolamine-ph	MF
N-acetylneuramir	MF
arachidonate 15-l	MF
glyceryl-ether mc	MF
imidazolonepropi	MF
arachidonic acid s	BP
IMP 5'-nucleotida	MF
GMP 5'-nucleotid	MF
intramolecular tra	MF
hyaluronan synth	MF
glucuronosyl-N-ac	MF
N-acetylglucosam	MF
N-acetylgalactosa	MF
lactosylceramide	MF
acyloxyacyl hydrc	MF
icosatetraenoic a	MF
arachidonic acid b	MF
aspartate-tRNA(A	MF
glutamate-tRNA((	MF
asparaginyl-tRNA	MF
glutaminyl-tRNA s	MF
protein-glutamin	MF

1,5-anhydro-D-fru	MF
GDP-L-fucose syn	MF
quinine 3-monoo	MF
methylarsonate r	MF
delta14-sterol red	MF
delta24-sterol red	MF
propionyl-CoA C2	MF
acetyl-CoA C-myr	MF
5-oxo-6E,8Z,11Z,1	MF
5-hydroxy-6E,8Z,1	MF
5(S)-hydroxypero	MF
testosterone 6-be	MF
chondroitin sulfat	BP
dermatan sulfate	BP
dermatan sulfate	BP
chondroitin sulfat	BP
dermatan sulfate	BP
3'-phosphoadeno	MF
RNA transport	BP
N-acetylgalactosa	MF
flavin adenine dir	MF
oxidoreductase a	MF
hydrogen peroxic	BP
regulation of hom	BP
homocysteine me	BP
positive regulatio	BP
positive regulatio	BP
negative regulatic	BP
epithelial cell pro	BP
regulation of epit	BP
positive regulatio	BP
negative regulatic	BP
androgen recepto	MF
AF-2 domain binc	MF
AF-1 domain binc	MF
regulation of mRN	BP
negative regulatic	BP
negative regulatic	BP
regulation of defe	BP
regulation of defe	BP
DNA binding dom	MF
LBD domain bindi	MF
galactose 3-O-sul	MF
proteoglycan sulf	MF
WW domain bind	MF

CARD domain bin	MF
regulation of prot	BP
positive regulatio	BP
regulation of infla	BP
negative regulatio	BP
positive regulatio	BP
regulation of pep	BP
positive regulatio	BP
negative regulatio	BP
RS domain bindin	MF
regulation of lipo	BP
positive regulatio	BP
negative regulatio	BP
low-density lipop	MF
regulation of pha	BP
negative regulatio	BP
positive regulatio	BP
regulation of neu	BP
negative regulatio	BP
positive regulatio	BP
regulation of axo	BP
negative regulatio	BP
positive regulatio	BP
regulation of den	BP
negative regulatio	BP
positive regulatio	BP
negative regulatio	BP
RNA destabilizatio	BP
dopamine recept	MF
advanced glycatio	MF
RAGE receptor bi	MF
detoxification of	BP
regulation of cata	BP
regulation of vira	BP
regulation of dev	BP
regulation of cell	BP
regulation of beh	BP
regulation of insu	BP
activated T cell p	BP
ion homeostasis	BP
modulation of ch	BP
negative regulatio	BP
positive regulatio	BP
regulation of syn	BP
regulation of ster	BP

GABA receptor bi	MF
regulation of acyl	BP
phosphoserine re	MF
phosphothreonin	MF
negative regulatio	BP
positive regulatio	BP
protein stabilizati	BP
peptide antigen s	BP
regulation of liqu	BP
defense response	BP
defense response	BP
defense response	BP
pyruvate transme	MF
cell adhesion mol	MF
extracellular mat	MF
progesterone rec	BP
negative regulatio	BP
positive regulatio	BP
antigen receptor-	BP
T cell receptor sig	BP
B cell receptor sig	BP
regulation of B ce	BP
regulation of T ce	BP
positive regulatio	BP
negative regulatio	BP
positive regulatio	BP
positive regulatio	BP
regulation of T ce	BP
regulation of B ce	BP
regulation of cell	BP
negative regulatio	BP
positive regulatio	BP
negative regulatio	BP
negative regulatio	BP
positive regulatio	BP
positive regulatio	BP
white fat cell diffe	BP
brown fat cell diff	BP
nervous system p	BP
regulation of bod	BP
musculoskeletal r	BP
voluntary muscul	BP
musculoskeletal r	BP
neuromuscular pr	BP
neuromuscular pr	BP

cognition	BP
multicellular orga	BP
intestinal absorpt	BP
sensory processir	BP
response to stimu	BP
cobalt ion binding	MF
leukocyte migrati	BP
leukocyte tetheri	BP
leukocyte adhesiv	BP
diapedesis	BP
neuromuscular pr	BP
detection of stim	BP
detection of chen	BP
detection of light	BP
sensory perceptic	BP
detection of mecl	BP
detection of chen	BP
detection of chen	BP
sensory perceptic	BP
sensory perceptic	BP
sensory perceptic	BP
sensory perceptic	BP
negative chemota	BP
regulation of che	BP
positive regulatio	BP
negative regulatic	BP
regulation of nega	BP
negative regulatic	BP
positive regulatio	BP
negative regulatic	BP
induction of nega	BP
induction of posit	BP
positive regulatio	BP
sensory perceptic	BP
sensory perceptic	BP
sensory perceptic	BP
thermoception	BP
equilibrioception	BP
detection of temp	BP
detection of temp	BP
detection of temp	BP
detection of mecl	BP
detection of chen	BP
detection of mecl	BP
detection of mecl	BP

sensory perceptic	BP
detection of mecl	BP
detection of mecl	BP
dimethylallyl diph	BP
regulation of lipic	BP
negative regulatic	BP
positive regulatio	BP
nitric-oxide synth	MF
regulation of nitri	BP
positive regulatio	BP
negative regulatic	BP
regulation of lipo	BP
negative regulatic	BP
positive regulatio	BP
Hsp27 protein bir	MF
microtubule plus-	MF
microtubule minu	MF
microtubule slidir	BP
microtubule seve	BP
actin filament sev	BP
actin filament bin	MF
barbed-end actin	BP
actin filament bur	BP
protein kinase A k	MF
mitogen-activate	MF
GTPase binding	MF
GDP-dissociation	MF
Rho GDP-dissocia	MF
chiasma assembly	BP
rRNA transport	BP
tRNA transport	BP
nucleic acid trans	MF
RNA transmembr	MF
regulation of end	BP
positive regulatio	BP
positive regulatio	BP
negative regulatic	BP
positive regulatio	BP
negative regulatic	BP
regulation of secr	BP
positive regulatio	BP
negative regulatic	BP
regulation of tran	BP
positive regulatio	BP
negative regulatic	BP

negative regulatic	BP
positive regulatio	BP
negative regulatic	BP
regulation of sma	BP
positive regulatio	BP
negative regulatic	BP
NF-kappaB bindin	MF
dihydrobiopterin	BP
dihydrolipoamide	BP
nuclear envelope	BP
unfolded protein	MF
'de novo' cotrans	BP
'de novo' posttra	BP
chaperone cofact	BP
chaperone media	BP
chaperone bindin	MF
constitutive prote	BP
regulation of DNA	BP
positive regulatio	BP
positive regulatio	BP
regulation of binc	BP
positive regulatio	BP
negative regulatic	BP
regulation of DNA	BP
DNA ligation invo	BP
DNA ligation invo	BP
positive regulatio	BP
ATPase binding	MF
sugar transmemb	MF
hepoxilin A3 synt	MF
hepoxilin metabo	BP
hepoxilin biosynt	BP
RNA polymerase	BP
synaptic growth a	BP
regulation of acti	BP
negative regulatic	BP
positive regulatio	BP
regulation of cellu	BP
positive regulatio	BP
chaperone-media	BP
positive regulatio	BP
regulation of NK	BP
positive regulatio	BP
metal ion:proton	MF
negative regulatic	BP

positive regulatio	BP
smooth muscle ce	BP
striated muscle ce	BP
regulation of mus	BP
negative regulatic	BP
positive regulatio	BP
regulation of smo	BP
negative regulatic	BP
positive regulatio	BP
regulation of stria	BP
negative regulatic	BP
positive regulatio	BP
glucose 6-phosph	BP
L-xylitol catabolic	BP
L-xylitol metaboli	BP
nuclear export	BP
nuclear transport	BP
import into nucle	BP
positive regulatio	BP
meiotic sister chr	BP
vitamin transport	BP
protein insertion	BP
protein insertion	BP
sequestering of ca	BP
dioxygenase activ	MF
cartilage develop	BP
phosphoprotein b	MF
cytoplasmic sequ	BP
positive regulatio	BP
regulation of prot	BP
negative regulatic	BP
spindle assembly	BP
mitotic spindle di	BP
spindle midzone	CC
sequestering of m	BP
regulation of prot	BP
positive regulatio	BP
negative regulatic	BP
regulation of lym	BP
negative regulatic	BP
positive regulatio	BP
regulation of RNA	BP
spindle midzone a	BP
mitotic spindle m	BP
meiotic spindle m	BP

protein polymeriz	BP
protein complex (	BP
protein homoolig	BP
protein depolyme	BP
protein tetrameri	BP
mono-olein trans	MF
diolein transacyla	MF
CP2 mannose-eth	MF
regulation of cellu	BP
negative regulatio	BP
positive regulatio	BP
chromosome orga	BP
regulation of rele	BP
negative regulatio	BP
regulation of sequ	BP
negative regulatio	BP
positive regulatio	BP
cell tip	CC
NAD binding	MF
protein heterotet	BP
protein heterooli	BP
nuclear pore com	BP
establishment of	BP
establishment of	BP
establishment of	BP
centrosome dupli	BP
centrosome sepa	BP
cell division	BP
regulation of cell	BP
establishment of	BP
chromosome sep	BP
mitotic sister chro	BP
meiotic chromoso	BP
male meiosis chro	BP
female meiosis ch	BP
metaphase plate	BP
meiotic metapha	BP
attachment of mi	BP
meiotic cell cycle	BP
regulation of oxid	BP
positive regulatio	BP
negative regulatio	BP
positive regulatio	BP
negative regulatio	BP
positive regulatio	BP

negative regulati	BP
positive regulatio	BP
positive regulatio	BP
negative regulati	BP
cellular response	BP
muscle alpha-acti	MF
FATZ binding	MF
mannose-ethanol	MF
serotonin binding	MF
epinephrine bindi	MF
norepinephrine b	MF
kinetochore asse	BP
kinetochore orga	BP
response to gluc	BP
response to mine	BP
regulation of neu	BP
negative regulati	BP
positive regulatio	BP
inactivation of M	BP
tRNA acetylation	BP
alpha-actinin bin	MF
BH domain bindir	MF
neuron apoptotic	BP
stress-activated M	BP
response to nitro	BP
detoxification of	BP
response to corti	BP
response to corti	BP
response to corti	BP
microtubule nucl	BP
microtubule nucl	BP
PTB domain bindi	MF
hormone recepto	MF
peptide hormone	MF
corticotropin-rele	MF
corticotropin-rele	MF
corticotropin-rele	MF
BH2 domain bind	MF
BH3 domain bind	MF
regulation of ubi	BP
positive regulatio	BP
negative regulati	BP
regulation of mei	BP
negative regulati	BP
myoblast prolifer	BP

myoblast migratio	BP
intracellular pH re	BP
regulation of intra	BP
intracellular pH e	BP
monopolar spindl	BP
maintenance of p	BP
positive regulatio	BP
negative regulatio	BP
positive regulatio	BP
positive regulatio	BP
regulation of cyto	BP
positive regulatio	BP
regulation of filop	BP
negative regulatio	BP
positive regulatio	BP
regulation of stre	BP
regulation of cyto	BP
negative regulatio	BP
positive regulatio	BP
positive regulatio	BP
negative regulatio	BP
D-aminoacyl-tRNA	MF
D-tyrosyl-tRNA(Ty	MF
adenine nucleotid	BP
NFAT protein bind	MF
2 iron, 2 sulfur clu	MF
3 iron, 4 sulfur clu	MF
elastin metabolic	BP
elastin biosynthet	BP
negative regulatio	BP
keratinocyte migr	BP
negative regulatio	BP
positive regulatio	BP
flavone metabolic	BP
mitochondrial cal	BP
positive regulatio	BP
negative regulatio	BP
smooth endoplas	BP
histone H3-K9 me	BP
histone H3-K4 me	BP
regulation of histo	BP
regulation of histo	BP
positive regulatio	BP
negative regulatio	BP
negative regulatio	BP

positive regulatio	BP
5'-deoxyribose-5-	MF
regulation of neu	BP
positive regulatio	BP
dopamine uptake	BP
regulation of dop	BP
negative regulatic	BP
positive regulatio	BP
response to calciu	BP
response to folic	BP
detection of gluc	BP
response to meth	BP
response to meth	BP
meiotic recombin	BP
response to hydr	BP
exocyst localizatic	BP
response to elect	BP
proteolysis involv	BP
protein maturatic	BP
detection of stim	BP
histamine transp	BP
serotonin uptake	BP
regulation of sero	BP
negative regulatic	BP
histamine uptake	BP
norepinephrine u	BP
regulation of nor	BP
negative regulatic	BP
positive regulatio	BP
epinephrine upta	BP
barbed-end actin	BP
actin filament ne	BP
organelle localiza	BP
cellular localizatic	BP
centrosome local	BP
endoplasmic retic	BP
Golgi localization	BP
mitochondrion lo	BP
nucleus localizati	BP
vesicle localizatio	BP
establishment of	BP
establishment of	BP
maintenance of l	BP
spindle localizatic	BP
establishment of	BP

establishment of	BP
maintenance of n	BP
establishment of	BP
maintenance of c	BP
nuclear pore loca	BP
membrane raft lo	BP
actin cortical patc	BP
localization withi	BP
membrane disrup	BP
establishment of	BP
maintenance of G	BP
maintenance of E	BP
actin filament cap	BP
pointed-end actir	BP
protein delipidati	BP
interaction with h	BP
interaction with s	BP
response to other	BP
regulation of killir	BP
positive regulatio	BP
inositol-1,3,4,5-te	MF
protein C-termina	MF
protein methyles	MF
NAD transmembr	MF
protein de-ADP-ri	BP
germline cell cycl	BP
polynucleotide 5'	MF
polydeoxyribonu	MF
ATP-dependent p	MF
ATP-dependent p	MF
cytosine C-5 DNA	MF
delta3,5-delta2,4	MF
alpha-1,4-manno	MF
meiotic sister chr	BP
homologous chro	BP
actin crosslink for	BP
inositol tetrakispl	MF
negative regulatic	BP
response to redo	BP
behavioral respor	BP
positive regulatio	BP
negative regulatic	BP
all-trans-retinol 1	MF
misfolded proteir	MF
response to misfc	BP

short-chain fatty .	BP
medium-chain fat	BP
medium-chain fat	BP
medium-chain fat	BP
regulation of timi	BP
positive regulatio	BP
negative regulatic	BP
regulation of hair	BP
positive regulatio	BP
negative regulatic	BP
phosphatidylinosi	MF
regulation of syna	BP
positive regulatio	BP
cytolysis by host (	BP
glycolipid binding	MF
histone demethyl	MF
protein autoubiqu	BP
general adaptatic	BP
methotrexate bin	MF
killing by host of s	BP
pigment granule l	BP
pigment granule a	BP
lateral element as	BP
G-quadruplex DN	MF
regulation of mitc	BP
mitochondrial de	BP
regulation of timi	BP
positive regulatio	BP
negative regulatic	BP
positive regulatio	BP
regulation of foca	BP
positive regulatio	BP
negative regulatic	BP
regulation of prot	BP
positive regulatio	BP
negative regulatic	BP
membrane depol	BP
regulation of mitc	BP
positive regulatio	BP
S-(hydroxymethyl	MF
pigment granule t	BP
double-stranded	MF
induction of syna	BP
granulocyte colon	MF
regulation of fibri	BP

negative regulatio	BP
positive regulatio	BP
peroxiredoxin act	MF
sulfation	BP
regulation of calc	BP
negative regulatio	BP
positive regulatio	BP
regulation of sens	BP
synaptic transmis	BP
glutamate reupta	BP
gamma-aminobu	BP
L-glutamate impo	BP
gamma-aminobu	BP
regulation of glut	BP
positive regulatio	BP
negative regulatio	BP
positive regulatio	BP
methotrexate tra	BP
dynein light inter	MF
regulation of nerv	BP
negative regulatio	BP
regulation of syna	BP
negative regulatio	BP
positive regulatio	BP
regulation of syna	BP
negative regulatio	BP
positive regulatio	BP
regulation of tran	BP
negative regulatio	BP
positive regulatio	BP
regulation of telo	BP
positive regulatio	BP
negative regulatio	BP
lysophospholipid	BP
lysophospholipid:	MF
regulation of chro	BP
positive regulatio	BP
positive regulatio	BP
regulation of atta	BP
(R)-2-hydroxyglut	MF
squalene synthas	MF
protein carboxyl (	MF
modulation by sy	BP
phytoalexin meta	BP
catabolism by hos	BP

suppression of sy	BP
tRNA dimethylally	MF
negative regulatio	BP
regulation of pep	BP
regulation of end	BP
sn-glycerol-3-pho	MF
sn-glycerol-3-pho	MF
tryptamine:oxyge	MF
aminoacetone:ox	MF
aliphatic-amine o	MF
phenethylamine:o	MF
diamine oxidase a	MF
histamine oxidase	MF
methylputrescine	MF
propane-1,3-diam	MF
phosphatidylinosi	MF
UDP-N-acetylgala	MF
lysophosphatidic	MF
NADP-retinol deh	MF
monoacylglycerol	BP
cyclic purine nucl	BP
L-leucine transam	MF
L-valine transami	MF
L-isoleucine trans	MF
guanine phospho	MF
inositol-1,4,5-tris	MF
inositol-1,3,4,5-te	MF
tRNA (cytosine-2'	MF
carboxylic ester h	MF
raffinose alpha-ga	MF
cellular glucuroni	BP
flavonoid glucuro	BP
xenobiotic glucur	BP
tRNA-specific ade	MF
fatty acid in-chair	MF
inositol hexakispł	MF
inositol hexakispł	MF
inositol-1,3,4-tris	MF
inositol-1,3,4-tris	MF
tRNA (cytosine-3-	MF
phosphatidylserir	MF
1-acyl-2-lysophos	MF
(R)-limonene 6-m	MF
phosphatidylinosi	MF
inositol phosphat	MF

inositol phosphor	BP
GDP-mannose hy	MF
exo-alpha-(2->3)-	MF
exo-alpha-(2->6)-	MF
exo-alpha-(2->8)-	MF
beta-galactoside	MF
1-phosphatidylin	MF
phosphatidylinosi	MF
medium-chain-al	MF
medium-chain ac	MF
long-chain acyl-C	MF
very long chain a	MF
DNA-7-methylad	MF
DNA-3-methylgu	MF
dolichyl-pyropho	MF
inositol hexakisp	MF
inositol-3,4-bisph	MF
inositol monopho	MF
inositol monopho	MF
inositol monopho	MF
inositol diphosph	MF
inositol diphosph	MF
ferric-chelate red	MF
ADP-dependent	MF
NADHX epimeras	MF
NADPHX epimera	MF
peptidyl-lysine ac	MF
phosphatidylinosi	MF
arachidonic acid	MF
alpha-tocopherol	MF
tocotrienol omeg	MF
all-trans-retinyl-p	MF
all-trans-retinyl-e	MF
norspermine:oxy	MF
N1-acetylspermir	MF
N(1),N(12)-diacet	MF
spermine:oxygen	MF
spermidine:oxyge	MF
N1-acetylspermir	MF
N1-acetylspermic	MF
tRNA (guanine(9)	MF
tRNA (guanine(37	MF
23S rRNA (adenin	MF
18S rRNA (adenin	MF
dol-P-Man:Man(7	MF

dol-P-Man:Man(8	MF
dol-P-Man:Man(5	MF
dol-P-Man:Man(6	MF
CTP:tRNA cytidyly	MF
CTP:3'-cytidine-tR	MF
ATP:3'-cytidine-cy	MF
muscle cell devel	BP
striated muscle ce	BP
cardiac myofibril	BP
ventricular cardia	BP
cardiac cell devel	BP
cardiac muscle ce	BP
cardiac muscle tis	BP
atrial cardiac mus	BP
ventricular cardia	BP
ventricular cardia	BP
cardiac muscle ce	BP
atrial cardiac mus	BP
ventricular cardia	BP
cardiac muscle tis	BP
positive regulatio	BP
negative regulatio	BP
regulation of card	BP
positive regulatio	BP
negative regulatio	BP
cortical microtub	CC
recycling endoson	CC
recycling endoson	CC
anastral spindle a	BP
D-glucose transm	MF
asymmetric neuro	BP
phosphate ion ho	BP
chloride ion home	BP
metal ion homeo	BP
zinc ion homeosta	BP
copper ion home	BP
manganese ion ho	BP
iron ion homeost	BP
cadmium ion hom	BP
calcium ion home	BP
potassium ion ho	BP
gap junction hem	MF
sodium ion home	BP
cation homeostas	BP
anion homeostas	BP

cellular chemical	BP
Ski complex	CC
lipid homeostasis	BP
fatty acid homeos	BP
phospholipid hom	BP
sterol homeostas	BP
response to hype	BP
low-density lipop	BP
adiponectin bindi	MF
lipase inhibitor ac	MF
ligase regulator a	MF
ubiquitin-protein	MF
ubiquitin-protein	MF
Golgi to secretory	BP
Golgi to transport	BP
ingression involve	BP
epiboly involved i	BP
regulation of carc	BP
negative regulatic	BP
relaxation of card	BP
striated muscle d	CC
digestive system	BP
L-proline biosynth	BP
C3HC4-type RING	MF
minus-end directe	MF
plus-end directed	MF
copper ion export	BP
vestibular reflex	BP
linear vestibuloo	BP
Sertoli cell differe	BP
Sertoli cell develc	BP
Sertoli cell fate cc	BP
Sertoli cell prolife	BP
synaptic transmis	BP
righting reflex	BP
granulosa cell dev	BP
parathyroid glanc	BP
astrocyte fate cor	BP
radial glial cell dif	BP
Bergmann glial ce	BP
roof of mouth de	BP
hard palate devel	BP
soft palate develc	BP
rhythmic synaptic	BP
regulation of syna	BP

convergent exten	BP
convergent exten	BP
convergent exten	BP
convergent exten	BP
notochord regres	BP
anatomical struct	BP
notochord cell de	BP
pharyngeal syster	BP
cardiac muscle ce	BP
pericardium deve	BP
retinal bipolar ne	BP
retina developme	BP
retina morphoger	BP
regulation of carc	BP
negative regulatic	BP
positive regulatio	BP
regulation of acrc	BP
heart contraction	BP
cardiac muscle cc	BP
regulation of prot	BP
positive regulatio	BP
negative regulatic	BP
neurofilament cyt	BP
neurofilament cyt	CC
positive regulatio	BP
angiogenesis invc	BP
mammary gland i	BP
positive regulatio	BP
embryonic retina	BP
post-embryonic r	BP
Spemann organiz	BP
Spemann organiz	BP
uterus developm	BP
oviduct developm	BP
cervix developme	BP
vagina developm	BP
canonical Wnt sig	BP
Wnt signaling pat	BP
large conductanc	MF
micturition	BP
synapse maturati	BP
regulation of rest	BP
excitatory synaps	CC
inhibitory synaps	CC
regulation of post	BP

excitatory postsy	BP
inhibitory postsyr	BP
membrane hyper	BP
smooth muscle c	BP
synaptic transmis	BP
circadian temper	BP
relaxation of vasc	BP
auditory receptor	BP
molecular adapto	MF
kinocilium	CC
positive regulatio	BP
generation of ovu	BP
inner ear recepto	BP
vestibular recept	BP
auditory receptor	BP
inner ear recepto	BP
vestibular recept	BP
inner ear recepto	BP
regulation of grov	BP
positive regulatio	BP
negative regulatic	BP
somatotropin sec	BP
prolactin secretin	BP
corticotropin hor	BP
thyroid-stimulatir	BP
prepulse inhibitio	BP
maternal process	BP
embryonic proce:	BP
maternal process	BP
positive regulatio	BP
positive regulatio	BP
microtubule-base	BP
platelet dense gr	BP
urinary bladder d	BP
phospholipase C-	BP
regulation of dop	BP
negative regulatic	BP
positive regulatio	BP
subpallium neuro	BP
regulation of timi	BP
regulation of timi	BP
olfactory pit deve	BP
positive regulatio	BP
negative regulatic	BP
ciliary membrane	CC

stereocilium men	CC
limb developmen	BP
limb bud formatic	BP
brain-derived neu	MF
regulation of angi	BP
regulation of exo	BP
male mating beha	BP
apelin receptor si	BP
cell pole	CC
negative regulatic	BP
positive regulatio	BP
cloacal septation	BP
clathrin-sculpted	CC
clathrin-sculpted	CC
cytoplasmic vesic	CC
negative regulatic	BP
positive regulatio	BP
endocardium forr	BP
primitive hemopc	BP
definitive hemop	BP
hemangioblast ce	BP
hematopoietic st	BP
camera-type eye	BP
camera-type eye	BP
retinal rod cell di	BP
phosphatidylchol	MF
lipoprotein lipase	MF
mesenchymal to	BP
delamination	BP
neuroblast delam	BP
lens induction in	BP
regulation of mit	BP
contact inhibition	BP
negative regulatic	BP
negative regulatic	BP
regulation of glial	BP
positive regulatio	BP
negative regulatic	BP
regulation of fee	BP
regulation of tran	BP
positive regulatio	BP
negative regulatic	BP
regulation of resp	BP
positive regulatio	BP
negative regulatic	BP

positive regulatio	BP
cilium assembly	BP
embryonic skelet	BP
positive regulatio	BP
positive regulatio	BP
negative regulatio	BP
cilium-dependent	BP
epithelial cilium n	BP
transdifferentiati	BP
long-term synapt	BP
long-term synapt	BP
cilium movement	BP
regulation of ciliu	BP
regulation of sarc	BP
positive regulatio	BP
regulation of cytc	BP
positive regulatio	BP
negative regulatio	BP
regulation of pho	BP
regulation of cell	BP
regulation of mer	BP
regulation of ven	BP
elastin catabolic p	BP
negative regulatio	BP
regulation of bloc	BP
negative regulatio	BP
regulation of ryar	BP
negative regulatio	BP
positive regulatio	BP
cardiac epithelial	BP
definitive erythro	BP
primitive erythro	BP
head developmer	BP
head morphogen	BP
face developmen	BP
face morphogene	BP
cell chemotaxis	BP
cytoplasmic actin	BP
regulation of resp	BP
positive regulatio	BP
positive regulatio	BP
negative regulatio	BP
regulation of type	BP
regulation of cellu	BP
photoreceptor in	CC

bone trabecula fc	BP
heart trabecula fc	BP
bone developmei	BP
bone morphogen	BP
endochondral boi	BP
cartilage develop	BP
cell adhesion mol	BP
negative regulatic	BP
positive regulatio	BP
negative regulatic	BP
response to amm	BP
cranial suture mo	BP
frontal suture mc	BP
lambdoid suture i	BP
sagittal suture mc	BP
regulation of Fc r	BP
positive regulatio	BP
susceptibility to T	BP
regulation of atria	BP
regulation of atria	BP
regulation of vent	BP
mast cell differen	BP
regulation of mas	BP
positive regulatio	BP
negative regulatic	BP
regulation of bro	BP
cardiac muscle ce	BP
positive regulatio	BP
regulation of DNA	BP
positive regulatio	BP
innervation	BP
axonogenesis inv	BP
synapse assembly	BP
pathway-restricte	BP
regulation of SMA	BP
positive regulatio	BP
negative regulatic	BP
regulation of patl	BP
negative regulatic	BP
SMAD protein sig	BP
growth hormone	BP
growth hormone	BP
regulation of grov	BP
positive regulatio	BP
negative regulatic	BP

cytosolic calcium	BP
calcium ion trans|	BP
axonemal microtu	BP
regulation of pen	BP
positive regulatio	BP
negative regulatic	BP
cardiac septum m	BP
ventricular septu	BP
atrial septum mo	BP
aorta smooth mu	BP
muscle tissue mo	BP
response to grow	BP
regulation of hea	BP
positive regulatio	BP
peptidyl-dipeptid	MF
lung morphogene	BP
lung vasculature	BP
lung epithelium d	BP
epithelium develc	BP
lung saccule deve	BP
primary lung bud	BP
bronchus develop	BP
bronchus morphc	BP
bronchiole develc	BP
lung growth	BP
trachea developn	BP
trachea morphog	BP
trachea formatio	BP
epithelial tube br	BP
branching involve	BP
mammary gland	BP
branching involve	BP
branching involve	BP
bud outgrowth in	BP
dichotomous sub	BP
bud elongation in	BP
positive regulatio	BP
regulation of gast	BP
positive regulatio	BP
negative regulatic	BP
right lung develo	BP
left lung develop	BP
lung lobe morphc	BP
pharynx develop	BP
activation of meic	BP

prevention of pol	BP
cortical granule e	BP
cortical granule	CC
positive regulatio	BP
acrosomal vesicle	BP
lung cell different	BP
lung goblet cell di	BP
lobar bronchus ep	BP
lobar bronchus de	BP
lung-associated m	BP
mesenchyme dev	BP
club cell different	BP
lung epithelial cel	BP
orthogonal dicho	BP
planar dichotomo	BP
lateral sprouting i	BP
regulation of cell	BP
lung induction	BP
positive regulatio	BP
epithelial cell pro	BP
bud dilation invol	BP
smoothened sign	BP
type I pneumocyt	BP
type II pneumocy	BP
prostate gland m	BP
prostatic bud forr	BP
primary prostatic	BP
epithelial cell pro	BP
activation of pros	BP
prostate epithelia	BP
prostate epithelia	BP
secretory column	BP
squamous basal e	BP
bronchus cartilag	BP
trachea cartilage	BP
trachea cartilage	BP
cartilage morpho	BP
muscle tissue dev	BP
skeletal muscle o	BP
diaphragm develo	BP
respiratory syster	BP
regulation of necı	BP
positive regulatio	BP
negative regulatio	BP
negative regulatio	BP

negative regulatic	BP
positive regulatio	BP
regulation of calc	BP
positive regulatio	BP
developmental gr	BP
apoptotic process	BP
epithelial tube m	BP
neuroepithelial ce	BP
positive regulatio	BP
morphogenesis o	BP
intestinal epitheli	BP
intestinal epitheli	BP
intestinal epitheli	BP
ventral spinal cor	BP
positive regulatio	BP
multicellular orga	BP
regulation of lipo	BP
negative regulatic	BP
ATPase regulator	MF
chondroblast diff	BP
mammary gland f	BP
mammary gland s	BP
fibroblast growth	BP
mammary placod	BP
dichotomous sub	BP
lateral sprouting i	BP
lateral sprouting f	BP
mammary gland c	BP
tube closure	BP
mammary gland f	BP
adipose tissue de	BP
fat pad developm	BP
mammary gland b	BP
regulation of chol	BP
regulation of chrc	BP
regulation of vesi	BP
regulation of ER t	BP
regulation of hor	BP
regulation of mei	BP
regulation of mici	BP
negative regulatic	BP
mesenchymal-epi	BP
mammary gland e	BP
mammary gland b	BP
submandibular sa	BP

salivary gland cav	BP
epithelial cell pro	BP
regulation of bran	BP
dichotomous sub	BP
branch elongatio	BP
regulation of bran	BP
embryonic placen	BP
branching involve	BP
epithelial cell mo	BP
placenta blood ve	BP
ureteric bud mor	BP
ureteric bud form	BP
dichotomous sub	BP
branch elongatio	BP
primary ureteric b	BP
regulation of bran	BP
epithelial-mesenc	BP
regulation of pros	BP
negative regulatio	BP
regulation of bran	BP
regulation of mor	BP
cell differentiatio	BP
regulation of bran	BP
regulation of chol	BP
regulation of pho	BP
positive regulatio	BP
endoribonuclease	MF
regulation of end	BP
negative regulatio	BP
deoxyribonucleas	MF
cell differentiatio	BP
trophoblast giant	BP
spongiotrophobla	BP
glycogen cell diffe	BP
chorio-allantoic fu	BP
labyrinthine layer	BP
spongiotrophobla	BP
labyrinthine layer	BP
syncytiotrophobla	BP
labyrinthine layer	BP
chorion developm	BP
chorionic trophob	BP
spongiotrophobla	BP
regulation of spon	BP
intestinal epitheli	BP

positive regulatio	BP
positive regulatio	BP
regulation of end	BP
regulation of eIF2	BP
prostate gland gr	BP
epithelial-mesenc	BP
mesenchymal-epi	BP
prostate gland ep	BP
prostate gland str	BP
epithelial cell diff	BP
epithelial cell mat	BP
mammary gland b	BP
mammary gland b	BP
parental behavior	BP
tertiary branching	BP
mammary gland a	BP
epithelial cell pro	BP
branch elongation	BP
regulation of mas	BP
positive regulatio	BP
negative regulatio	BP
negative regulatio	BP
regulation of bran	BP
mammary duct te	BP
cell-cell signaling	BP
regulation of and	BP
negative regulatio	BP
regulation of epit	BP
positive regulatio	BP
negative regulatio	BP
planar cell polarit	BP
regulation of mes	BP
mesenchymal sm	BP
regulation of cell	BP
hair follicle placo	BP
cell fate commitm	BP
regulation of cell	BP
BMP signaling pat	BP
positive regulatio	BP
negative regulatio	BP
mesodermal to m	BP
random inactivati	BP
inactivation of pa	BP
inactivation of X c	BP
inactivation of X c	BP

inactivation of X c	BP
fibroblast growth	BP
negative regulatio	BP
ciliary receptor cl	BP
smoothened sign	BP
lymphatic endoth	BP
blood vessel endo	BP
lymphatic endoth	BP
artery developme	BP
venous blood ves	BP
arterial endotheli	BP
venous endotheli	BP
arterial endotheli	BP
Notch signaling p	BP
branching involve	BP
establishment of	BP
establishment of	BP
anterior semicircu	BP
lateral semicircula	BP
limb epidermis de	BP
neural plate patte	BP
embryonic camer	BP
regulation of hair	BP
positive regulatio	BP
regulation of prot	BP
positive regulatio	BP
cardiac cell fate s	BP
cardiac cell fate d	BP
heart formation	BP
mesenchymal cel	BP
mesenchymal cel	BP
cardiac pacemake	BP
sinoatrial node ce	BP
cardiac muscle ce	BP
atrioventricular n	BP
sinoatrial node ce	BP
sinoatrial node ce	BP
His-Purkinje syste	BP
epicardium-deriv	BP
epithelial to mese	BP
cardiac neuron di	BP
cardiac vascular s	BP
cardiac vascular s	BP
endocardial cell d	BP
regulation of gen	BP

negative regulatic	BP
regulation of gen	BP
embryonic heart	BP
left/right pattern	BP
cell migration inv	BP
cardioblast migra	BP
coronary vasculat	BP
coronary vasculat	BP
angiogenesis invc	BP
vasculogenesis in	BP
cell migration inv	BP
coronary artery n	BP
lipid tube	CC
lipid tube assemb	BP
lipid tube assemb	BP
response to fungi	BP
kidney morphoge	BP
regulation of tran	BP
dendritic spine de	BP
dendritic spine m	BP
regulation of den	BP
positive regulatio	BP
negative regulatic	BP
regulation of den	BP
negative regulatic	BP
positive regulatio	BP
cell differentiatio	BP
common bile duc	BP
gall bladder devel	BP
positive regulatio	BP
snRNA import int	BP
membrane organ	BP
cardiac muscle tis	BP
establishment of	BP
eyelid developme	BP
epithelial cell diff	BP
endodermal diges	BP
secretion by lung	BP
olfactory bulb mit	BP
regulation of cart	BP
positive regulatio	BP
negative regulatic	BP
uterus morphoge	BP
female gonad mo	BP
regulation of wou	BP

vascular wound h	BP
regulation of vasc	BP
negative regulatic	BP
negative regulatic	BP
regulation of brar	BP
positive regulatio	BP
negative regulatic	BP
cell growth involv	BP
positive regulatio	BP
negative regulatic	BP
somite developm	BP
myotome develo|	BP
sclerotome devel	BP
muscle structure	BP
iris morphogenes	BP
ciliary body morp	BP
regulation of neu	BP
chaperone-media	BP
negative regulatic	BP
regulation of hist	BP
negative regulatic	BP
positive regulatio	BP
regulation of seq	BP
positive regulatio	BP
positive regulatio	BP
regulation of prot	BP
positive regulatio	BP
negative regulatic	BP
lung neuroendocı	BP
neuroendocrine c	BP
stomach neuroen	BP
carotid body glon	BP
adrenal chromaff	BP
negative regulatic	BP
seminal vesicle e|	BP
pancreas morpho	BP
branching involve	BP
endopeptidase ac	MF
endopeptidase re	MF
regulation of prot	BP
morphogenesis o	BP
lung ciliated cell c	BP
alveolar primary s	BP
alveolar secondaı	BP
lung smooth mus	BP

Peyer's patch mo	BP
BMP signaling pat	BP
BMP signaling pat	BP
trachea gland dev	BP
endothelial tube	BP
pulmonary artery	BP
pulmonary artery	BP
mRNA destabiliza	BP
3'-UTR-mediated	BP
establishment of	BP
establishment of	BP
endoplasmic retic	BP
negative regulatic	BP
regulation of insu	BP
negative regulatic	BP
mammary gland e	BP
regulation of cho	BP
negative regulatic	BP
positive regulatio	BP
negative regulatic	BP
negative regulatic	BP
positive regulatio	BP
taste bud develop	BP
fungiform papilla	BP
fungiform papilla	BP
fungiform papilla	BP
clathrin-sculpted	CC
paramesonephric	BP
canonical Wnt sig	BP
retina vasculature	BP
retina vasculature	BP
cerebellum vascu	BP
cerebellum vascu	BP
smooth muscle ce	BP
cornea developm	BP
retinal blood vess	BP
cardiac neural cre	BP
cardiac neural cre	BP
BMP signaling pat	BP
Notch signaling ir	BP
canonical Wnt sig	BP
canonical Wnt sig	BP
cell proliferation i	BP
renal tubule morp	BP
cardiac conductic	BP

establishment or	BP
cell adhesion invo	BP
planar cell polarit	BP
planar cell polarit	BP
planar cell polarit	BP
planar cell polarit	BP
neural precursor	BP
planar cell polarit	BP
Wnt protein secre	BP
positive regulatio	BP
negative regulatio	BP
apoptotic process	BP
behavioral respor	BP
negative regulatio	BP
testosterone bios	BP
determination of	BP
mammillothalami	BP
mammary gland l	BP
inferior colliculus	BP
cell migration in c	BP
trabecula morpho	BP
heart trabecula m	BP
closure of optic fi	BP
regulation of tran	BP
positive regulatio	BP
positive regulatio	BP
positive regulatio	BP
positive regulatio	BP
regulation of tran	BP
positive regulatio	BP
negative regulatio	BP
bone trabecula m	BP
cellular response	BP
establishment of	BP
renal artery morp	BP
endocardial cushi	BP
endocardial cushi	BP
connective tissue	BP
trophoblast cell n	BP
reproductive syst	BP
L-arginine transm	MF
protein localizatio	BP
O-acetyl-ADP-ribo	MF
regulation of type	BP
T follicular helper	BP

phagolysosome n	CC
response to TNF a	BP
hematopoietic st	BP
memory T cell pr	BP
cyclic-GMP-AMP	MF
early endosome t	BP
cyclic-GMP-AMP	MF
CDP phosphoryla	BP
centriole elongati	BP
protein localizatio	BP
glucose 6-phosph	MF
interleukin-34-me	BP
myeloid cell deve	BP
microglial cell pro	BP
macrophage hom	BP
Langerhans cell d	BP
hindgut developn	BP
acetylcholine sec	BP
glycogen synthas	MF
ganglion develop	BP
sympathetic gang	BP
cranial ganglion d	BP
trigeminal ganglio	BP
axon developmen	BP
dAMP phosphory	BP
CMP phosphoryla	BP
dCMP phosphory	BP
GDP phosphoryla	BP
UDP phosphoryla	BP
dCDP phosphoryl	BP
TDP phosphorylat	BP
actin filament bu	BP
actin filament bu	BP
ASAP complex	CC
cyclin-dependent	MF
calcium ion trans	BP
Lys63-specific de	MF
colon epithelial c	BP
intestinal epitheli	BP
positive regulatio	BP
calcium activated	BP
calcium activated	BP
calcium activated	BP
calcium activated	BP
molybdopterin ac	MF

molybdopterin m	MF
molybdopterin-sy	MF
molybdopterin-sy	MF
nuclear import si	MF
fructose-1-phosp	MF
mannose to fruct	BP
pri-miRNA transc	BP
glycolytic process	BP
MICOS complex	CC
glycolytic process	BP
canonical glycoly	BP
glycolytic process	BP
fructose catabolic	BP
pharyngeal arch a	BP
S-methylmethion	MF
H3K27me3 modif	MF
RNA polymerase	MF
ubiquitin conjuga	MF
RNA lariat debran	MF
regulation of prot	BP
cytoskeleton-dep	BP
chemoattraction	BP
chemorepulsion c	BP
positive regulatio	BP
histone H3-K9 mo	BP
ubiquitin modifica	MF
NEDD8 conjugatin	MF
SUMO conjugatin	MF
UFM1 conjugating	MF
NEDD8 ligase acti	MF
SUMO ligase activ	MF
UFM1 ligase activ	MF
mitochondrial rib	BP
spontaneous neu	BP
evoked neurotran	BP
mitotic spindle as	CC
importin-alpha fa	MF
chaperone-media	BP
diphthine methyl	MF
tricellular tight ju	CC
lipoamidase activ	MF
protein-glutarylly	MF
protein deglutary	BP
peptidyl-lysine de	BP
GATOR2 complex	CC

inflammasome cc	CC
tRNA-5-taurinom	MF
reticulophagy	BP
N(6)-L-threonylca	MF
tRNA (N(6)-L-thre	MF
anterior neural tu	BP
folic acid recepto	MF
miRNA 2'-O-meth	BP
glycophagy	BP
lipophagy	BP
methylglyoxal cat	BP
ribonucleoside-di	MF
mitochondrial ace	BP
peptide-lysine-N-	MF
parkin-mediated	BP
engulfment of tar	BP
leukotriene signa	BP
late endosomal m	BP
protein lipidation	BP
protein targeting	BP
chaperone-media	BP
chaperone-media	CC
motor learning	BP
motor behavior	BP
forked DNA-depe	MF
acid sphingomyel	MF
neutral sphingom	MF
telomeric repeat-	MF
substrate localiza	BP
leukocyte adhesio	BP
antifungal innate	BP
CAMKK-AMPK sig	BP
multivesicular bo	BP
positive regulatio	BP
negative regulatio	BP
magnesium:sodiu	MF
ribosylnicotinate	MF
translation elonga	MF
response to calor	BP
xenobiotic transp	BP
dense core granu	BP
pH-gated chloride	MF
GTP 3',8'-cyclase	MF
cyclic pyranopter	MF
NAD+ nucleotidas	MF

ADP-ribosyl cycla:	MF
cyclic ADP-ribose	MF
endoplasmic retic	BP
telomeric DNA-cc	BP
telomeric D-loop	BP
telomeric D-loop	MF
podosome core	CC
sperm head	CC
apical tubulobulb	CC
basal tubulobulba	CC
concave side of s|	CC
apical ectoplasmi	CC
basal ectoplasmic	CC
protein localizatic	BP
neuropeptide prc	BP
microtubule orga	BP
neuron projectior	CC
dendritic spine cy	CC
response to chole	BP
telomeric G-quad	MF
growth cone lead	CC
leading edge of la	CC
negative regulatic	BP
endoplasmic retic	BP
DNA clamp unloa	MF
microtubule plus	MF
positive regulatio	BP
negative regulatic	BP
positive regulatio	BP
positive regulatio	BP
regulation of astr	BP
negative regulatic	BP
positive regulatio	BP
calcium ion sensc	MF
all-trans retinol 3,	MF
all-trans retinal 3,	MF
all-trans retinoic a	MF
11-cis-retinal 3,4-	MF
positive regulatio	BP
autophagosome-l	BP
autophagosome-(	BP
selective autopha	BP
protein propionyl	MF
peptidyl-lysine pr	BP
glutathione speci	MF

gamma-glutamyla	MF
negative regulatio	BP
establishment of	BP
midbody abscissio	BP
mRNA (adenine-N	MF
penetration of cu	BP
establishment of	BP
perichondral bon	BP
articular cartilage	BP
3-hydroxykynurer	MF
negative regulatio	BP
negative regulatio	BP
secondary palate	BP
collagen-containi	CC
negative regulatio	BP
regulation of stre	BP
positive regulatio	BP
negative regulatio	BP
D-loop DNA bindi	MF
positive regulatio	BP
GPI-mannose eth	MF
TAP complex binc	MF
box C/D snoRNP c	MF
box H/ACA snoRN	MF
GARP complex bi	MF
H3K9me3 modifie	MF
acyl-CoA delta5-d	MF
TSC1-TSC2 compl	MF
HLA-E specific inh	MF
stomach develop	BP
regulation of prog	BP
negative regulatio	BP
positive regulatio	BP
peptidyl-aspartic	MF
double-stranded	BP
RNA 2'-O-methylt	MF
fatty acid primary	BP
4-hydroxybutyrat	MF
mRNA (cytidine-5	MF
C5-methylcytidin	MF
N6-mAMP deami	MF
mitochondrial AT	MF
mitochondrial AT	CC
brexanolone met	BP
brexanolone cata	BP

R-loop disassemb	BP
radial spoke asse	BP
25-hydroxycholec	MF
1-alpha,25-dihydı	MF
all-trans retinoic a	MF
all-trans retinoic a	MF
testosterone 16-b	MF
secalciferol 1-mo	MF
anandamide 8,9 e	MF
anandamide 11,1	MF
anandamide 14,1	MF
positive regulatio	BP
peroxynitrite isor	MF
platelet activating	BP
protein localizatic	BP
specification of a:	BP
intracellular prote	BP
protein-containin	BP
protein-DNA com	BP
extracellular men	CC
aspartic-type pep	MF
glutamic-type pe	MF
cysteine-type exc	MF
metalloaminopep	MF
serine-type exope	MF
oligopeptidase ac	MF
armadillo repeat	MF
carbon monoxide	MF
nitric oxide bindir	MF
synaptobrevin 2-S	CC
synaptobrevin 2-S	CC
telomerase RNA b	MF
rRNA (pseudouric	MF
rRNA (guanosine-	MF
rRNA (uridine-N3	MF
synaptobrevin 2-S	CC
neuron cellular h	BP
fibrinogen bindin	MF
collagen V bindin	MF
thrombospondin	MF
mRNA splicing, vi	BP
intrinsic apoptoti	BP
'de novo' actin fil	BP
fructose binding	MF
extracellular exos	CC

RNA polymerase	MF
proline-rich regio	MF
cytochrome comp	CC
proton-transporti	BP
vacuolar proton-t	BP
histone lysine der	BP
histone H3-R2 de	BP
histone H4-R3 de	BP
titin Z domain bin	MF
clathrin-sculpted	CC
protein initiator r	BP
glycosylation	BP
ubiquitin-depend	BP
chromo shadow c	MF
chloride-activate	MF
glucagon secretio	BP
regulation of gluc	BP
negative regulatio	BP
positive regulatio	BP
fructose-6-phosp	MF
delta-catenin bin	MF
negative regulatio	BP
positive regulatio	BP
negative regulatio	BP
positive regulatio	BP
ciliary neurotroph	CC
ciliary neurotroph	MF
ciliary neurotroph	BP
Kupffer's vesicle c	BP
isopeptidase activ	MF
transforming grov	MF
mitochondrial tra	BP
mitochondrial tra	BP
mitochondrial tra	BP
tRNA aminoacyla	BP
regulation of mit	BP
negative regulatio	BP
positive regulatio	BP
SUMO-specific er	MF
SUMO-specific iso	MF
response to UV-A	BP
synaptic vesicle b	BP
mitochondrial ala	BP
mitochondrial asp	BP
mitochondrial gly	BP

mitochondrial ser	BP
mitochondrial thr	BP
tight junction	CC
negative regulatio	BP
positive regulatio	BP
enamel mineraliz	BP
negative regulatio	BP
positive regulatio	BP
negative regulatio	BP
positive regulatio	BP
positive regulatio	BP
DRM complex	CC
D-serine metabol	BP
D-serine biosynth	BP
large ribosomal s	MF
small ribosomal s	MF
DNA polymerase	MF
mitochondrial try	BP
mitochondrial tyr	BP
shelterin complex	CC
kynurenine metal	BP
methionine-R-sul	MF
chromosome org	BP
synaptonemal co	BP
synaptonemal co	BP
growth hormone	CC
meiotic attachme	BP
protein localizatio	BP
establishment of	BP
regulation of esta	BP
regulation of esta	BP
protein trimerizat	BP
protein homotrim	BP
protein heterotri	BP
ASTRA complex	CC
protein poly-ADP	BP
protein auto-ADP	BP
sulfide oxidation,	BP
sulfide:quinone o	MF
regulation of lym	BP
T cell apoptotic p	BP
regulation of T ce	BP
negative regulatio	BP
positive regulatio	BP
regulation of acti	BP

negative regulati	BP
positive regulatio	BP
thymocyte apopt	BP
regulation of thy	BP
negative regulati	BP
positive regulatio	BP
pristanate-CoA lig	MF
actin-mediated ce	BP
mucus secretion	BP
negative regulati	BP
positive regulatio	BP
5'-tyrosyl-DNA ph	MF
peptidyl-serine de	BP
necrotic cell deat	BP
necroptotic proce	BP
oncosis	BP
cornification	BP
pyroptosis	BP
phosphatidylinosi	MF
RES complex	CC
extracellular mat	BP
vitamin B6 bindin	MF
pyridoxal binding	MF
axonemal dynein	BP
N-acylphosphatid	MF
N-acylethanolami	BP
N-acylphosphatid	BP
renal absorption	BP
renal sodium ion	BP
renal water abso	BP
sarcoplasmic reti	BP
phosphatidic acid	MF
cellular response	BP
regulation of stre	BP
positive regulatio	BP
response to cGM	BP
lens fiber cell diff	BP
lens fiber cell dev	BP
lens fiber cell mo	BP
G1 to G0 transitic	BP
G1 to G0 transitic	BP
regulation of G0 t	BP
negative regulati	BP
positive regulatio	BP
Golgi to plasma m	CC

inward rectifier p	MF
thyroid hormone	MF
lipoprotein partic	MF
very-low-density	MF
thyroid hormone	BP
triglyceride home	BP
aromatase activit	MF
aspartate binding	MF
flap-structured D	MF
3'-flap-structured	MF
negative regulatio	BP
positive regulatio	BP
positive regulatio	BP
regulation of whi	BP
actin polymerizat	BP
hepatocyte differ	BP
negative regulatio	BP
positive regulatio	BP
beta-catenin-TCF	CC
cellular heat accli	BP
ERK1 and ERK2 ca	BP
regulation of ERK	BP
positive regulatio	BP
ERK5 cascade	BP
positive regulatio	BP
endosome to plas	CC
exocytic vesicle	CC
DNA cytosine dea	BP
Harderian gland c	BP
transcription exp	CC
detection of lipot	BP
NADP+ binding	MF
NADPH binding	MF
NAD+ binding	MF
NADH binding	MF
oxidation-depend	BP
carbamoyl phosp	BP
co-SMAD binding	MF
I-SMAD binding	MF
R-SMAD binding	MF
trehalose metabo	BP
cellular response	BP
DNA-dependent p	CC
nonhomologous e	CC
DNA ligase III-XRC	CC

nucleotide-bindin	BP
regulation of nucl	BP
nucleotide-bindin	BP
negative regulatic	BP
positive regulatio	BP
nucleotide-bindin	BP
negative regulatic	BP
positive regulatio	BP
Shc-EGFR comple	CC
Grb2-EGFR compl	CC
regulation of oligo	BP
negative regulatic	BP
positive regulatio	BP
elongin complex	CC
regulation of hem	BP
positive regulatio	BP
cellular detoxifica	BP
prolactin secretio	BP
thyroid-stimulatir	BP
SAGA-type compl	CC
plus-end specific	BP
tubulin-depender	MF
respirasome	CC
uterine smooth m	BP
regulation of uter	BP
negative regulatic	BP
positive regulatio	BP
rRNA base methy	BP
rRNA (guanine-N7	BP
nuclear-transcrib	BP
nuclear-transcrib	BP
detection of hypo	BP
leukocyte aggrega	BP
monocyte aggreg	BP
neutrophil aggreg	BP
T cell aggregation	BP
oligosaccharide b	MF
thrombin-activate	BP
negative regulatic	BP
interleukin-1-mec	BP
poly-gamma-gluta	BP
high-density lipop	MF
regulation of micı	BP
calcium ion impoı	BP
positive regulatio	BP

death domain bin	MF
CAK-ERCC2 comp	CC
ERCC4-ERCC1 cor	CC
11-beta-hydroxys	MF
tRNA threonylcar	BP
platelet aggregati	BP
protein kinase C s	BP
K63-linked polyul	MF
BRCA1-A comple›	CC
protein K63-linke	BP
histone H2A K63-	BP
protein K63-linke	BP
histone H2A K63-	BP
oleic acid binding	MF
linoleic acid bindi	MF
response to platir	BP
response to linole	BP
histone H3-K36 d	BP
PeBoW complex	CC
L-glutamine amin	MF
endoribonuclease	MF
BRISC complex	CC
nicotinic acid rece	MF
synaptobrevin 2-S	CC
response to inter	BP
PCNA-p21 comple	CC
protein secretion	BP
vitamin D recepto	BP
regulation of vita	BP
negative regulatio	BP
positive regulatio	BP
adenylyltransfera	MF
cytidylyltransfera	MF
guanylyltransfera	MF
uridylyltransferas	MF
regulation of neu	BP
negative regulatio	BP
positive regulatio	BP
metallodipeptidas	MF
cadmium ion trar	BP
vitamin D 24-hyd	MF
lysine-acetylated	MF
RISC-loading com	CC
methylcytosine d	MF
mitochondrion m	BP

protein localizatio	BP
cell-cell adhesion	BP
dendrite self-avoi	BP
regulation of cent	BP
histone methyltra	MF
histone methyltra	MF
regulation of prot	BP
nucleosome-depe	MF
Grb2-Sos complex	CC
zymogen granule	BP
(S)-2-(5-amino-1-	MF
proteasome bind	MF
transepithelial tra	BP
transepithelial am	BP
vitamin D3 metab	BP
vitamin D 25-hyd	MF
vitamin D respons	MF
protein modificat	BP
formin-nucleated	BP
nonfunctional rRN	BP
HAUS complex	CC
high-density lipop	MF
leukocyte prolifer	BP
mast cell prolifera	BP
regulation of leuk	BP
negative regulatio	BP
positive regulatio	BP
negative regulatio	BP
positive regulatio	BP
response to inter	BP
response to inter	BP
response to inter	BP
response to inter	BP
response to inter	BP
hypoxanthine deh	MF
hypoxanthine oxi	MF
intralumenal vesi	BP
preprotein bindin	MF
inositol 1,4,5 trisp	MF
glutaminyl-tRNAG	BP
proteasome regu	BP
seminal clot lique	BP
macropinocytic c	CC
P-TEFb complex	CC
deoxyribonucleos	MF

FHF complex	CC
activin receptor b	MF
type I activin rece	MF
type II activin rec	MF
BMP receptor bin	MF
mucus layer	CC
sodium-depende	BP
mismatch repair i	BP
response to chole	BP
BMP receptor cor	CC
leucine binding	MF
cAMP transport	BP
cGMP transport	BP
protein adenylylt	MF
histone H3-K27 m	BP
protein-glycine lig	MF
protein-glycine lig	MF
protein-glutamic	MF
tubulin-glutamic a	MF
response to inter	BP
C2H2 zinc finger c	MF
interleukin-23 co	CC
pre-snoRNP comp	CC
nuclear pore tran	CC
gamma-secretase	CC
PAS complex	CC
protein-N-termin	MF
phytoceramidase	MF
MOZ/MORF histo	CC
D-aspartate trans	BP
L-aspartate trans	BP
D-aspartate impo	BP
dihydrosphingosi	MF
response to bioti	BP
phosphatidylserir	BP
hydrogen sulfide	BP
hydrogen sulfide	BP
peptidyl-lysine 5-	MF
phosphorylation c	BP
tertiary granule	CC
tertiary granule n	CC
Sin3-type comple	CC
paraferritin comp	CC
chromatin mainte	BP
heterochromatin	BP

heterochromatin	BP
bicellular tight jur	BP
basement membr	BP
caveola assembly	BP
dehydroascorbic	BP
divalent metal ior	BP
dynein complex b	MF
aggresome assem	BP
polyubiquitinatec	BP
Hsp90 deacetylat	BP
core mediator co	CC
response to grow	BP
response to epide	BP
growth factor rec	MF
cell body fiber	CC
myosin VI binding	MF
myosin VI light ch	MF
regulation of bile	BP
negative regulatio	BP
RNA polymerase	CC
regulation of prot	BP
negative regulatio	BP
positive regulatio	BP
heterochromatin	BP
regulation of glyc	BP
SOSS complex	CC
microprocessor c	CC
primary miRNA bi	MF
pre-miRNA bindir	MF
regulation of calc	BP
negative regulatio	BP
positive regulatio	BP
E-box binding	MF
platelet alpha gra	BP
lipoteichoic acid k	MF
lipoteichoic acid i	MF
negative regulatio	BP
transcription prei	BP
RNA polymerase	BP
mitochondrial tRI	BP
mitochondrial tRI	BP
mitochondrial tRI	BP
mitochondrial tRI	BP
transepithelial L-a	BP
serine binding	MF

global genome nu	BP
UV-damage excis	BP
lysophosphatidic	MF
small RNA loading	BP
organelle assemb	BP
regulation of ATP	BP
Golgi-associated v	CC
histone H3 deace	BP
histone H4 deace	BP
CRD-mediated ml	BP
3'-UTR-mediated	BP
CRD-mediated ml	CC
contractile ring	CC
Dsl1/NZR comple	CC
dephosphorylatic	BP
neutrophil-media	BP
neutrophil-media	BP
regulation of neu	BP
regulation of neu	BP
positive regulatio	BP
nuclear-transcribe	BP
endoplasmic retic	CC
protein localizatic	BP
protein localizatic	BP
POU domain bind	MF
FHA domain bind	MF
TIR domain bindir	MF
bone maturation	BP
protein K11-linke	BP
biphenyl cataboli	BP
L-asparagine bios	BP
L-asparagine met	BP
transcription fact	CC
left/right axis spe	BP
error-free transle	BP
demethylation	BP
oxidative demeth	BP
snRNP binding	MF
medium-chain-ac	MF
detection of oxida	BP
NADPH oxidation	BP
type 1 melanocor	MF
neuron death	BP
response to magr	BP
U4/U6 snRNP	CC

U2-type presplice	CC
U2-type precataly	CC
U2-type catalytic	CC
U2-type catalytic	CC
U2-type post-mRI	CC
precatalytic splice	CC
catalytic step 2 sp	CC
post-mRNA releas	CC
post-spliceosoma	CC
U2-type post-spli	CC
RNA surveillance	BP
nuclear mRNA su	BP
nuclear retention	BP
CUT catabolic prc	BP
nuclear polyaden	BP
nuclear polyaden	BP
nuclear polyaden	BP
nuclear polyaden	BP
nuclear polyaden	BP
nuclear polyaden	BP
nuclear polyaden	BP
histone mRNA ca	BP
nuclear histone m	BP
nuclear retention	BP
nuclear retention	BP
snoRNA polyaden	BP
polyadenylation-c	BP
alphav-beta3 inte	CC
alpha9-beta1 inte	CC
regulation of pho	BP
negative regulatio	BP
positive regulatio	BP
eukaryotic initiati	MF
RNA 3' uridylation	BP
DNA conformatio	BP
response to inter	BP
response to parat	BP
protein K48-linke	BP
superior tempora	BP
histone biotinylat	BP
alpha9-beta1 inte	CC
resolution of recc	BP
resolution of mitc	BP
SMAD protein co	CC
heteromeric SMA	CC

regulation of cell	BP
negative regulatic	BP
positive regulatio	BP
NF-kappaB compl	CC
CMG complex	CC
DNA replication p	BP
RNA trimethylgua	MF
ribonucleoprotein	BP
protein localizatic	BP
establishment of	BP
site-specific DNA	BP
WASH complex	CC
histone pre-mRN/	CC
protein localizatic	BP
establishment of	BP
histone pre-mRN/	MF
histone pre-mRN/	MF
U7 snRNA binding	MF
protein targeting	BP
cellular response	BP
cellular response	BP
cellular response	BP
cellular response	BP
cellular response	BP
cellular response	BP
cellular response	BP
cellular response	BP
cellular response	BP
cellular response	BP
cellular response	BP
cellular response	BP
cellular response	BP
cellular response	BP
cellular response	BP
cellular response	BP
cellular response	BP
cellular response	BP
cellular response	BP
connexin binding	MF
cellular response	BP
cellular response	BP
cellular response	BP
cellular response	BP
Ssh1 translocon c	CC
positive regulatio	BP

L-methionine salv	BP
cellular response	BP
cellular response	BP
cellular response	BP
cellular response	BP
cellular response	BP
cellular response	BP
cellular response	BP
cellular response	BP
cellular response	BP
cellular response	BP
cellular response	BP
cellular response	BP
cellular response	BP
cellular response	BP
cellular response	BP
cellular response	BP
cellular response	BP
cellular response	BP
cellular response	BP
cellular response	BP
cellular response	BP
cellular response	BP
cellular response	BP
cellular response	BP
cellular response	BP
cellular response	BP
cellular response	BP
cellular response	BP
MLL1 complex	CC
skeletal muscle a	BP
diphosphate meta	BP
cellular response	BP
cellular response	BP
cellular response	BP
cellular response	BP
cellular response	BP
cellular response	BP
cellular response	BP
cellular response	BP
cellular response	BP
cellular response	BP
cellular response	BP
cellular response	BP

cellular response	BP
cellular response	BP
cellular response	BP
cellular response	BP
cellular response	BP
cellular response	BP
cellular response	BP
cellular response	BP
cellular response	BP
cellular response	BP
cellular response	BP
cellular response	BP
cellular response	BP
cellular response	BP
cellular response	BP
cellular response	BP
cellular response	BP
cellular response	BP
cellular response	BP
cellular response	BP
cellular response	BP
cellular response	BP
cellular response	BP
cellular response	BP
cellular response	BP
cellular response	BP
cellular response	BP
cellular response	BP
cellular response	BP
cellular response	BP
manganese ion tr	BP
succinate transm	BP
malate transmem	BP
rRNA (cytosine-N	MF
hematopoietic st	BP
clathrin complex	CC
regulation of hist	BP
negative regulatio	BP
positive regulatio	BP
cellular response	BP
cellular response	BP
cellular response	BP
cellular response	BP
cellular response	BP
cellular response	BP

cellular response	BP
integral compone	CC
protein localizatic	BP
cellular response	BP
cellular response	BP
cellular response	BP
cellular response	BP
cellular response	BP
cellular response	BP
cellular response	BP
cellular response	BP
cellular response	BP
cellular hyperosm	BP
cellular hyperosm	BP
cellular hypotoni	BP
cellular hypotoni	BP
cellular response	BP
cellular response	BP
cellular response	BP
cellular response	BP
cellular response	BP
cellular response	BP
cellular response	BP
cellular response	BP
cellular response	BP
cellular response	BP
cellular response	BP
cellular response	BP
cellular response	BP
cellular response	BP
response to hepa	BP
cellular response	BP
cellular response	BP
phosphopantothe	CC
genetic imprintin	BP
maintenance of i	BP
semaphorin-plexi	BP
tRNA re-export fr	BP
cementum miner	BP
ankyrin repeat bi	MF
RING-like zinc fin	MF
protein localizatic	BP
eukaryotic transl	CC
eukaryotic transl	CC
dopaminergic ne	BP

diphosphoinosito	BP
diphosphoinosito	BP
pi-body	CC
piP-body	CC
response to dexa	BP
cellular response	BP
death-inducing si	BP
integral compone	CC
histone H3-K27 d	BP
histone demethyl	MF
response to trans	BP
cellular response	BP
nucleus-vacuole j	CC
npBAF complex	CC
nBAF complex	CC
UFM1 activating	MF
UFM1 hydrolase	MF
UFM1 transferase	MF
protein ufmylatio	BP
integral compone	CC
zinc ion transmer	BP
zinc ion import ac	BP
regulation of zinc	BP
negative regulatio	BP
detoxification of	BP
CAAX-box proteir	BP
lymphocyte aggre	BP
Nem1-Spo7 phos	CC
ubiquitin-depend	BP
neuronal ribonuc	CC
otic vesicle devel	BP
otic vesicle morpl	BP
phytosphingosine	BP
endothelial cell-c	BP
transforming grov	BP
linoleic acid epox	MF
lysophospholipid	MF
granulocyte chem	BP
regulation of gran	BP
positive regulatio	BP
vocalization beha	BP
cytoplasm protei	BP
nuclear protein q	BP
dihydroceramida	MF
negative regulatio	BP

positive regulatio	BP
negative regulatio	BP
regulation of mac	BP
negative regulatio	BP
positive regulatio	BP
negative regulatio	BP
positive regulatio	BP
positive regulatio	BP
negative regulatio	BP
positive regulatio	BP
positive regulatio	BP
positive regulatio	BP
catenin-TCF7L2 co	CC
gamma-catenin-T	CC
smooth muscle ce	BP
negative regulatio	BP
positive regulatio	BP
mononuclear cell	BP
negative regulatio	BP
positive regulatio	BP
olfactory bulb axo	BP
commissural neu	BP
cellular response	BP
endocytic vesicle	CC
cardiac muscle th	BP
regulation of MA	BP
organic substance	BP
organic substance	BP
nitrogen compou	BP
immunoglobulin	BP
membrane assem	BP
basement memb	BP
ER-associated mis	BP
icosanoid transm	MF
leukotriene trans	BP
sodium-independ	BP
lipopeptide bindi	MF
cellular response	BP
cellular response	BP
response to nitric	BP
cellular response	BP
transcriptional ac	BP
secretory IgA imn	CC
nuclear membrar	BP
nuclear inner me	BP

cellular response	BP
response to fibro	BP
endoplasmic retic	CC
endoplasmic retic	BP
endoplasmic retic	BP
endoplasmic retic	BP
K11-linked polyul	MF
K6-linked polyubi	MF
LUBAC complex	CC
cellular response	BP
podosome assem	BP
regulation of pod	BP
positive regulatio	BP
potassium ion tra	BP
protein transmen	BP
positive regulatio	BP
lipoprotein partic	MF
tail-anchored me	BP
MMXD complex	CC
BAT3 complex	CC
DUBm complex	CC
N-box binding	MF
FANCM-MHF com	CC
triglyceride-rich li	BP
intermediate-den	BP
HMG box domain	MF
cell proliferation i	BP
apoptotic process	BP
actin filament del	BP
TNFSF11-mediate	BP
positive regulatio	BP
mitotic cell cycle	BP
neuropeptide rec	MF
regulation of cell	BP
positive regulatio	BP
negative regulatio	BP
response to catec	BP
cellular response	BP
response to epine	BP
cellular response	BP
response to norep	BP
cellular response	BP
adrenergic recept	BP
negative regulatio	BP
positive regulatio	BP

adenylate cyclase	BP
adenylate cyclase	BP
phospholipase C-a	BP
N-terminal protei	MF
leukocyte apopto	BP
macrophage apop	BP
14-3-3 protein bi	MF
bicarbonate bindi	MF
BMP signaling pat	BP
histone H2B cons	BP
odontoblast diffe	BP
protein localizatio	BP
DNA biosynthetic	BP
negative regulatio	BP
regulation of prot	BP
negative regulatio	BP
positive regulatio	BP
determination of	BP
determination of	BP
determination of	BP
determination of	BP
synchronous neur	BP
citrate secondary	MF
prominosome	CC
dipeptide transm	MF
urea transmembr	BP
G-quadruplex DN	BP
cleavage body	CC
cohesin loading	BP
regulation of coh	BP
endocannabinoid	BP
alpha-tubulin ace	BP
negative regulatio	BP
positive regulatio	BP
replication fork re	BP
Arp2/3 complex b	MF
thiamine transme	BP
coreceptor activit	MF
vitamin A import	BP
XPC complex	CC
cell periphery	CC
protein deubiquit	BP
FAD binding	MF
conversion of me	BP
elastic fiber	CC

chemokine (C-C n	BP
recycling endosor	BP
mitotic sister chr	BP
establishment or	BP
fungal-type cell w	BP
extracellular exos	BP
cell gliding	BP
multivesicular bo	BP
Ragulator comple	CC
WD40-repeat dor	MF
kidney rudiment f	BP
nephron develop	BP
glomerular epithe	BP
glomerular endot	BP
glomerulus vascu	BP
proximal tubule c	BP
glomerular viscer	BP
glomerular pariet	BP
distal tubule deve	BP
nephron morpho	BP
renal vesicle form	BP
renal vesicle indu	BP
mesenchymal ste	BP
negative regulatio	BP
collecting duct de	BP
establishment of	BP
proximal/distal pa	BP
comma-shaped b	BP
S-shaped body m	BP
renal inner medu	BP
renal outer medu	BP
outer medullary c	BP
inner medullary c	BP
loop of Henle dev	BP
kidney epithelium	BP
metanephric mes	BP
nephrogenic mes	BP
renal vesicle mor	BP
nephron tubule m	BP
nephron tubule fo	BP
nephron tubule d	BP
specification of lo	BP
stem cell prolifera	BP
regulation of ster	BP
ureteric bud inva	BP

regulation of brar	BP
negative regulatic	BP
specification of u	BP
glomerulus morp	BP
glomerular capilla	BP
ureteric peristalsi	BP
positive regulatio	BP
positive regulatio	BP
glomerular mesar	BP
cell proliferation i	BP
glomerular viscer	BP
negative regulatic	BP
positive regulatio	BP
metanephric mes	BP
nephrogenic mes	BP
condensed meser	BP
mesenchymal cel	BP
glomerular pariet	BP
renal interstitial f	BP
mesangial cell de	BP
glomerular mesar	BP
epithelial cell fate	BP
distal tubule mor	BP
nephron tubule e	BP
mesenchymal cel	BP
mesonephric epit	BP
mesonephric tubu	BP
posterior mesone	BP
specification of ar	BP
specification of p	BP
metanephric tubu	BP
mesonephric tubu	BP
metanephric tubu	BP
metanephric tubu	BP
epithelial tube fo	BP
mesonephric duc	BP
nephric duct mor	BP
nephric duct form	BP
mesonephric duc	BP
mesonephric duc	BP
regulation of nep	BP
negative regulatic	BP
renal vesicle prog	BP
ureter developme	BP
ureter urothelium	BP

ureter epithelial c	BP
ureter smooth mu	BP
ureter morphoge	BP
mesenchymal cel	BP
negative regulatio	BP
negative regulatio	BP
cell differentiatio	BP
cell proliferation i	BP
metanephric colle	BP
metanephric epit	BP
metanephric smo	BP
metanephric nep	BP
metanephric caps	BP
metanephric cort	BP
metanephric asce	BP
metanephric cort	BP
metanephric dista	BP
metanephric glon	BP
metanephric prox	BP
metanephric nep	BP
metanephric dista	BP
metanephric loop	BP
metanephric prox	BP
metanephric glon	BP
metanephric glon	BP
metanephric glon	BP
metanephric glon	BP
metanephric inte	BP
metanephric glon	BP
metanephric caps	BP
pattern specificat	BP
proximal/distal pa	BP
metanephric nep	BP
metanephric glon	BP
metanephric glon	BP
metanephric glon	BP
metanephric com	BP
metanephric nep	BP
metanephric rena	BP
metanephric S-sh	BP
mesenchymal to e	BP
metanephric con	BP
metanephric dista	BP
metanephric nep	BP
positive regulatio	BP

positive regulatio	BP
negative regulatio	BP
regulation of met	BP
clathrin coat disa:	BP
vesicle uncoating	BP
volume-sensitive	MF
chaperone-media	BP
signal transductio	BP
intrinsic apoptoti	BP
UDP-galactose tra	BP
modified amino a	MF
rescue of stalled	BP
NAADP-sensitive	MF
response to anest	BP
tricarboxylic acid	BP
histone kinase ac	MF
histone H3-T3 ph	BP
chromosome pas	BP
PTW/PP1 phosph	CC
circulatory systen	BP
blood coagulation	BP
TRC complex	CC
minus-end-direct	BP
plus-end-directec	BP
organelle transpo	BP
minus-end-direct	BP
plus-end-directec	BP
flavin adenine dir	BP
microtubule anch	BP
detection of stim	BP
signal transductio	BP
detection of DNA	BP
signal transductio	BP
response to DNA	BP
signal transductio	BP
signal transductio	BP
response to intra	BP
signal transductio	BP
response to G1 D	BP
signal transductio	BP
MSL complex	CC
ammonium trans	BP
mesenchymal ste	BP
embryonic skelet	BP
divalent inorganic	BP

positive regulatio	BP
Rho-dependent p	MF
seminiferous tubu	BP
purine-containing	BP
pyrimidine-contai	BP
perineuronal net	CC
interleukin-23 rec	CC
fibroblast activati	BP
T-helper 17 type i	BP
T-helper 17 cell d	BP
T-helper 17 cell li	BP
protein phosphat	MF
L-DOPA binding	MF
tyrosine binding	MF
ER membrane pro	CC
terminal button c	BP
blood vessel lume	BP
17-beta-ketostero	MF
IPAF inflammasor	CC
NLRP1 inflammas	CC
NLRP3 inflammas	CC
type B pancreatic	BP
blood micropartic	CC
endothelial micro	CC
ADP-D-ribose bin	MF
poly-ADP-D-ribos	MF
tolerance inductio	BP
hepatocyte prolif	BP
liver morphogene	BP
endothelial cell a	BP
glycine receptor c	BP
17-beta-hydroxys	MF
clathrin-depende	BP
caveolin-mediate	BP
DNA topoisomera	MF
box H/ACA scaRN	CC
N-acetyl-L-aspart	MF
citrate-L-glutama	MF
oxygen metabolic	BP
establishment of	BP
establishment of	BP
maintenance of p	BP
protein localizatic	BP
protein localizatic	BP
maintenance of p	BP

establishment of	BP
establishment of	BP
tRNA-splicing liga	CC
neutrophil extrav	BP
lamellipodium mo	BP
multinuclear oste	BP
osteoclast fusion	BP
lymphocyte migra	BP
T cell migration	BP
thymocyte migrat	BP
T cell extravasatic	BP
mitochondrial tRI	BP
mitotic spindle	CC
meiotic spindle	CC
protein localizatic	BP
cellular response	BP
cellular response	BP
response to sorbi	BP
cellular response	BP
response to hydro	BP
cellular response	BP
cellular response	BP
response to actin	BP
cellular response	BP
response to cispla	BP
cellular response	BP
response to dithio	BP
cellular response	BP
cellular response	BP
cellular response	BP
response to aniso	BP
cellular response	BP
cellular response	BP
cellular response	BP
cellular response	BP
cellular response	BP
cellular response	BP
positive regulatio	BP
entry of viral gen	BP
microtubule-depe	BP
IRES-dependent v	BP
viral translational	BP
viral translational	BP
transport of viral	BP
establishment of	BP

viral penetration	BP
intracellular trans	BP
Cul4-RING E3 ubi	CC
mRNA methylatic	BP
fatty-acyl-CoA re	MF
phosphatidylinosi	MF
methyl indole-3-a	MF
GDP-D-glucose pl	MF
protein deglutath	BP
regulation of prin	BP
phosphatidyl-N-d	MF
DNA demethylati	BP
AMP transport	BP
AMP transmembr	MF
proteasome core	BP
fatty acid alpha-h	MF
regulation of cell	BP
amino acid home	BP
L-cysteine desulfl	MF
regulation of ferti	BP
regulation of prot	BP
regulation of nitri	BP
hydrogen peroxic	BP
response to phen	BP
entry into host ce	BP
maintenance of s	BP
protein K6-linked	BP
extracellular mat	BP
modulation by sy	BP
cardiac muscle ce	BP
cardiac muscle ce	BP
cardiac muscle ce	BP
regulation of carc	BP
ventricular cardia	BP
voltage-gated soc	MF
voltage-gated cal	MF
voltage-gated pot	MF
membrane repola	BP
membrane depol	BP
membrane repola	BP
membrane depol	BP
membrane repola	BP
atrial cardiac mus	BP
SA node cell actic	BP
AV node cell actic	BP

cell-cell signaling	BP
gap junction char	MF
SA node cell to at	BP
adenylate cyclase	BP
adenylate cyclase	BP
Purkinje myocyte	BP
adenylate cyclase	BP
regulation of carc	BP
calcium:sodium a	MF
calcium-transport	MF
sodium:proton ar	MF
voltage-gated pot	MF
cardiac muscle ce	BP
bundle of His cell	BP
atrial cardiac mus	BP
membrane depol	BP
membrane depol	BP
membrane depol	BP
membrane depol	BP
AV node cell to bu	BP
bundle of His cell	BP
Purkinje myocyte	BP
voltage-gated cal	MF
voltage-gated cal	MF
voltage-gated cal	MF
voltage-gated soc	MF
voltage-gated soc	MF
voltage-gated soc	MF
voltage-gated soc	MF
cell communicati	BP
atrial cardiac mus	BP
AV node cell to bu	BP
bundle of His cell	BP
SA node cell to at	BP
AV node cell-bun	BP
bundle of His cell-	BP
gap junction char	MF
gap junction char	MF
gap junction char	MF
gap junction char	MF
gap junction char	MF
protein binding ir	MF
cell adhesive prot	MF
cell adhesive prot	MF
voltage-gated pot	MF

regulation of hea	BP
regulation of the	BP
angiotensin-activ	BP
endothelin recep	BP
protein kinase D	BP
U2AF	CC
L-histidine transn	BP
L-histidine transn	BP
spanning compor	CC
amino acid impor	BP
caspase binding	MF
primitive streak f	BP
regulation of neu	BP
positive regulatio	BP
negative regulatio	BP
regulation of mor	BP
negative regulatio	BP
positive regulatio	BP
positive regulatio	BP
regulation of pro	BP
positive regulatio	BP
negative regulatio	BP
tubulin deacetyla	BP
regulation of tub	BP
positive regulatio	BP
positive regulatio	BP
regulation of cell	BP
positive regulatio	BP
negative regulatio	BP
positive regulatio	BP
regulation of pro	BP
regulation of thal	BP
positive regulatio	BP
negative regulatio	BP
negative regulatio	BP
relaxation of mus	BP
relaxation of skel	BP
positive regulatio	BP
regulation of hea	BP
positive regulatio	BP
regulation of incl	BP
negative regulatio	BP
regulation of pro	BP
negative regulatio	BP
negative regulatio	BP

positive regulatio	BP
metanephric cap	BP
positive regulatio	BP
negative regulatio	BP
cochlea developm	BP
cochlea morphog	BP
regulation of high	BP
positive regulatio	BP
COPII-coated vesi	BP
COPII-coated vesi	BP
C-5 methylation c	BP
endosome to lyso	BP
receptor-mediate	BP
vesicle-mediated	BP
cholesterol ester	BP
protein-containin	BP
regulation of syna	BP
positive regulatio	BP
mesenchyme mig	BP
epithelium migra	BP
mesendoderm m	BP
cell migration inv	BP
actin filament bra	BP
epithelial cell-cell	BP
regulation of acti	BP
regulation of mito	BP
positive regulatio	BP
mitochondrial nu	BP
membrane fission	BP
mitochondrial me	BP
establishment of	BP
establishment of	BP
establishment of	BP
regulation of sphi	BP
negative regulatio	BP
cellular sphingolip	BP
endoplasmic retic	BP
Golgi to lysosome	BP
Golgi ribbon form	BP
establishment of	BP
asymmetric Golgi	BP
Golgi disassembly	BP
Golgi reassembly	BP
regulation of spin	BP
regulation of Golg	BP

microtubule cyto:	BP
regulation of esta	BP
microtubule cyto:	BP
establishment of	BP
regulation of esta	BP
planar cell polarit	BP
regulation of chol	BP
positive regulatio	BP
positive regulatio	BP
negative regulatic	BP
regulation of brar	BP
positive regulatio	BP
negative regulatic	BP
positive regulatio	BP
negative regulatic	BP
regulation of rele	BP
positive regulatio	BP
negative regulatic	BP
protein localizatic	BP
positive regulatio	BP
regulation of trigl	BP
negative regulatic	BP
regulation of esta	BP
negative regulatic	BP
positive regulatio	BP
negative regulatic	BP
positive regulatio	BP
negative regulatic	BP
chromosome loca	BP
centrosome-temp	BP
regulation of micr	BP
regulation of cent	BP
regulation of kine	BP
regulation of met	BP
regulation of arac	BP
positive regulatio	BP
regulation of hist	BP
positive regulatio	BP
negative regulatic	BP
retinoic acid rece	BP
Wnt signaling pat	BP
axis elongation in	BP
convergent exten	BP
regulation of cell	BP
protein localizatic	BP

negative regulatio	BP
regulation of reti	BP
negative regulatio	BP
positive regulatio	BP
positive regulatio	BP
regulation of imm	BP
regulation of mit	BP
positive regulatio	BP
positive regulatio	BP
negative regulatio	BP
negative regulatio	BP
regulation of pep	BP
positive regulatio	BP
regulation of calc	BP
positive regulatio	BP
negative regulatio	BP
positive regulatio	BP
positive regulatio	BP
negative regulatio	BP
regulation of cell	BP
negative regulatio	BP
negative regulatio	BP
nuclear matrix an	BP
regulation of mit	BP
positive regulatio	BP
negative regulatio	BP
negative regulatio	BP
positive regulatio	BP
nucleic acid phos	BP
spindle assembly	BP
mitotic spindle as	BP
regulation of DNA	BP
positive regulatio	BP
negative regulatio	BP
regulation of prot	BP
positive regulatio	BP
regulation of prot	BP
positive regulatio	BP
negative regulatio	BP
positive regulatio	BP
negative regulatio	BP
regulation of sup	BP
negative regulatio	BP
regulation of loco	BP
negative regulatio	BP

regulation of DNA	BP
regulation of plat	BP
negative regulatio	BP
regulation of brov	BP
positive regulatio	BP
positive regulatio	BP
negative regulatio	BP
positive regulatio	BP
negative regulatio	BP
cellular organoflu	BP
negative regulatio	BP
regulation of plat	BP
positive regulatio	BP
negative regulatio	BP
negative regulatio	BP
phagosome matu	BP
phagosome acidif	BP
phagosome-lysos	BP
phagosome-lysos	BP
phagolysosome a	BP
phagosome-lysos	BP
negative regulatio	BP
cellular senescen	BP
replicative senesc	BP
stress-induced pr	BP
oncogene-induce	BP
oxidative stress-ir	BP
malonyl-CoA synt	MF
malonate catabol	BP
nicotinate transm	MF
thiamine pyropho	MF
acinar cell differe	BP
palmitoyl-CoA liga	MF
oleoyl-CoA ligase	MF
protein localizatio	BP
FAR/SIN/STRIPAK	CC
glutamate homeo	BP
dibasic protein pr	BP
pyrimidine nucleo	BP
vitamin transmen	MF
small RNA 2'-O-m	MF
dopamine uptake	BP
mesenchymal cel	BP
extrinsic compon	CC
endocardial cushi	BP

RNA phosphodies	BP
RNA phosphodies	BP
sphingolipid med	BP
glomerular viscer	BP
vesicle tethering i	BP
actin filament rec	BP
cation-transportii	CC
calcium ion-trans	CC
CERF complex	CC
MIT domain bindi	MF
Flemming body	CC
phosphatidylchol	MF
phosphatidyletha	MF
phosphatidylserir	MF
establishment of	BP
regulation of mer	BP
2-(3-amino-3-carl	MF
RNA polymerase	CC
RNA polymerase	CC
alpha-glucosidase	MF
ubiquitin-indeper	BP
mitochondrial mI	BP
DNA clamp unloa	BP
meiotic spindle p	CC
endoribonuclease	MF
mRNA cleavage ir	BP
activation of GTP	BP
outer dense plaqu	CC
inner dense plaqu	CC
mitochondrial tRI	BP
modulation of ag	BP
response to envir	BP
response to oxyg	BP
cellular response	BP
apical cytoplasm	CC
t-circle formation	BP
telomeric loop di	BP
walking behavior	BP
cerebrospinal flui	BP
box H/ACA telom	CC
galanin-activated	BP
scaRNA localizatio	BP
endothelial cell cl	BP
telomerase RNA s	BP
telomerase RNA l	BP

endothelial cell-m	BP
intermicrovillar a	BP
calcium ion trans	BP
Scc2-Scc4 cohesir	CC
primary adaptive	BP
receptor-recepto	MF
central region of	CC
peripheral region	CC
Las1 complex	CC
site of DNA dama	CC
telomere mainter	BP
glycolysis from st	BP
acetylcholine rec	BP
ceramide binding	MF
adipokinetic horn	MF
regulation of plas	BP
energy homeosta	BP
eukaryotic transla	BP
cellular response	BP
phagocytic vesicle	CC
ciliary plasm	CC
L27 domain bindi	MF
renal protein abs	BP
COPII adaptor act	MF
lymphocyte migra	BP
lymphocyte migra	BP
fructose 6-phospl	MF
MPP7-DLG1-LIN7	CC
ubiquitin-protein	MF
dendritic cell diffe	BP
mature conventic	BP
regulation of mer	BP
regulation of plas	BP
heme export	BP
perinuclear endo	CC
protein linear pol	BP
phosphatidylserir	BP
motor neuron ap	BP
type B pancreatic	BP
establishment of	BP
L-kynurenine met	BP
L-kynurenine cata	BP
agmatine biosynt	BP
selenocysteinyl-t	BP
TRAF2-GSTP1 cor	CC

CRLF-CLCF1 comp	CC
CNTFR-CLCF1 con	CC
synaptic membra	CC
dendritic spine or	BP
dendritic spine m	BP
anterior head dev	BP
response to thyrc	BP
cellular response	BP
response to thyrc	BP
cellular response	BP
ductus arteriosus	BP
selenite:proton sy	MF
plasma membran	BP
vascular smooth	BP
methyl-branched	BP
presynaptic mem	BP
synaptic vesicle c	BP
craniofacial sutur	BP
supercoiled DNA	MF
blood vessel endo	BP
endothelial tip ce	BP
postsynaptic mer	BP
presynaptic mem	BP
postsynaptic den:	BP
postsynaptic den:	BP
hedgehog family	MF
neuroligin family	MF
scaffold protein b	MF
endoplasmic retic	BP
gamma-aminobu	BP
AMPA glutamate	BP
NMDA glutamate	BP
neurexin clusterir	BP
gephyrin clusterir	BP
guanylate kinase-	BP
neuroligin cluster	BP
postsynaptic den:	BP
receptor localizat	BP
cyclin A1-CDK2 cc	CC
cyclin A2-CDK2 cc	CC
cyclin B1-CDK1 cc	CC
cyclin D2-CDK4 cc	CC
cyclin E1-CDK2 cc	CC
cyclin E2-CDK2 cc	CC
Bcl-2 family prote	CC

BIM-BCL-xl compl	CC
BIM-BCL-2 compl	CC
BAX complex	CC
BAK complex	CC
centralspindlin cc	CC
neuronal stem ce	BP
positive regulatio	BP
mesenchymal cel	BP
cysteine-type enc	MF
GABAergic neuro	BP
fasciculation of se	BP
fasciculation of m	BP
pre-mRNA intron	MF
pre-mRNA intron	MF
organic cyclic con	MF
polychlorinated b	MF
DH domain bindir	MF
MADS box domai	MF
sulfur carrier acti	MF
nuclear stress gra	CC
circadian regulati	BP
mesenchymal ste	BP
AIM2 inflammasc	CC
epoxide metaboli	BP
mitochondrial rib	MF
ruffle assembly	BP
protease inhibito	CC
serine protease ir	CC
cellular response	BP
amelogenesis	BP
dentinogenesis	BP
dentin mineraliza	BP
extrinsic apoptoti	BP
execution phase c	BP
pilomotor reflex	BP
Shu complex	CC
tetraspanin-enric	CC
histone H3-K36 tr	BP
cysteine-type enc	MF
cysteine-type enc	MF
negative regulatic	BP
activation of cyst	BP
renal filtration	BP
alveolar lamellar	CC
epidermal lamella	CC

cellular response	BP
lysosomal membi	BP
regulation of lyso	BP
positive regulatio	BP
mitochondrial mF	BP
sperm connecting	CC
sperm midpiece	CC
sperm mitochonc	CC
sperm annulus	CC
sperm principal p	CC
sperm end piece	CC
lamellar body me	CC
epidermal lamell	CC
cellular response	BP
cellular response	BP
hematopoietic st	BP
amyloid-beta clea	BP
flavonoid binding	MF
mitochondrial res	BP
oligodendrocyte a	BP
R2TP complex	CC
leukotriene B4 12	MF
self proteolysis	BP
omega-hydroxyla	BP
cytoophidium	CC
all-trans-decapre	MF
urea homeostasis	BP
cellular ammonia	BP
cellular creatinine	BP
cellular urea hom	BP
complement-dep	BP
immune complex	BP
hepatocyte apopt	BP
renal phosphate i	BP
activation of cyst	BP
regulation of nucl	BP
programmed nec	BP
response to alcol	BP
cap1 mRNA meth	BP
cap2 mRNA meth	BP
plasma membran	BP
7SK snRNA bindin	MF
B cell adhesion	BP
melanocyte migra	BP
melanocyte proli	BP

melanocyte adhe	BP
response to antin	BP
response to antip	BP
response to cloza	BP
inhibition of cyste	BP
ripoptosome	CC
ripoptosome asse	BP
Rix1 complex	CC
mitochondrial ou	BP
neutrophil cleara	BP
autophagosome r	BP
protein localizatic	BP
perinucleolar con	CC
UDP-glucosylatio	BP
CIA complex	CC
MCM8-MCM9 co	CC
protein O-GlcNAc	MF
carbohydrate der	MF
establishment of	BP
protein O-GlcNAc	BP
MDM2/MDM4 fa	MF
NAD-dependent I	MF
sensory neuron a	BP
interneuron axon	BP
dorsal spinal cord	BP
dorsal spinal cord	BP
photoreceptor di	CC
dIDP diphosphata	MF
glial cell projectio	CC
interleukin-17-me	BP
synaptic vesicle lu	BP
neuroblast migra	BP
cellular response	BP
glial cytoplasmic i	CC
hypoxia-inducible	BP
Lewy body	CC
classical Lewy bo	CC
neurofibrillary ta	CC
liver regeneratior	BP
tubular endosom	CC
microtubule bunc	CC
protein maturatic	BP
mitotic spindle pc	CC
dense body	CC
supramolecular fi	BP

apical dendrite	CC
basal dendrite	CC
CA3 pyramidal ce	CC
sorting endosome	CC
spine apparatus	CC
dendritic tree	CC
astrocyte projecti	CC
astrocyte end-foo	CC
glial limiting end-	CC
GAIT complex	CC
Schwann cell mici	CC
hippocampal mos	CC
Lewy neurite	CC
ubiquitin-depend	BP
programmed cell	BP
ribbon synapse	CC
cyclin-dependent	MF
retinal rod cell ap	BP
retinal cone cell a	BP
motor neuron mi	BP
lateral motor colu	BP
dendrite extensio	BP
neuron projectio	BP
multivesicular bo	CC
multivesicular bo	CC
multivesicular bo	CC
sympathetic neur	BP
sympathetic neur	BP
structural molecu	MF
regulation of vesi	BP
blood vessel endo	BP
endothelial tube	BP
protein localizatio	BP
receptor localizat	BP
stress response to	BP
mannosylation	BP
sialylation	BP
Gemini of coiled	CC
Rad6-Rad18 comp	CC
base-excision rep	BP
cardiac myofibril	CC
myosin II filamen	CC
DNA recombinase	CC
sperm plasma me	CC
spliceosomal snR	CC

spliceosomal tri-s	CC
necroptotic signa	BP
execution phase (	BP
granulocyte migra	BP
mast cell migratic	BP
cellular stress res	BP
lymphoid lineage	BP
thymus epitheliur	BP
ciliary transition f	CC
axonemal basal p	CC
ciliary tip	CC
ciliary inversin co	CC
axonemal outer c	CC
ciliary base	CC
transcription prei	CC
mitochondrial do	BP
calcium ion transı	BP
glutathione oxido	MF
lateral cell cortex	CC
sequestering of ir	BP
lamellipodium or	BP
sperm cytoplasm	CC
cullin family prote	MF
temperature-gate	MF
monoamine oxida	MF
potassium ion ex	BP
extrinsic compon	CC
integral compone	CC
extrinsic compon	CC
extrinsic compon	CC
integral compone	CC
L-arginine import	BP
amylin receptor a	MF
amylin receptor s	BP
A axonemal micro	CC
serpin family prot	MF
3',5'-nucleotide b	MF
histone H3-K36 d	BP
STAT family prote	MF
double-strand bre	BP
double-strand bre	BP
intracellular phos	MF
histone H3-K4 mc	BP
establishment of	BP
establishment of	BP

telomere mainte	BP
vascular endothe	BP
intracellular vesic	CC
ciliary basal body	BP
vesicle targeting,	BP
disordered doma	MF
calcineurin-medi	BP
LEM domain bind	MF
9+0 motile cilium	CC
9+2 motile cilium	CC
non-motile cilium	CC
9+0 non-motile ci	CC
photoreceptor ce	CC
extracellular exos	BP
urate salt excreti	BP
mitochondrial tR	BP
regulation of bloc	BP
membrane tubul	BP
endosome memb	BP
regulation of DN	BP
membrane bendi	BP
non-replicative tr	BP
G-rich strand telo	MF
polynucleotide 3'	BP
polynucleotide 5'	BP
endothelial to he	BP
nucleotide phosp	MF
skeletal muscle fi	BP
positive regulatio	BP
ligand-activated t	MF
histone H3-K27 tr	BP
centriole assembl	BP
de novo centriole	BP
deuterosome	CC
defense response	BP
lumenal side of G	CC
cytoplasmic side	CC
cytoplasmic side	CC
cytoplasmic side	CC
cytoplasmic side	CC
cytoplasmic side	CC
cytoplasmic side	CC
lumenal side of ly	CC
lumenal side of m	CC
inactive sex chror	CC

innate vocalizatio	BP
external side of a	CC
cytoplasmic side (	CC
mucin granule	CC
perivitelline spac	CC
palmitoyl hydrola	MF
cell-cell adhesion	BP
trans-Golgi netwo	BP
cell-cell adhesion	MF
collagen fibril bin	MF
protein complex i	CC
collagen binding i	MF
integrin binding i	MF
cadherin binding	MF
cation transmem	BP
anion transmemb	BP
import into cell	BP
inorganic anion ir	BP
inorganic cation i	BP
inorganic cation t	BP
G protein-couple	BP
G protein-couple	CC
extrinsic compon	CC
template-free RN	MF
photoreceptor rib	CC
Schaffer collatera	CC
hippocampal mos	CC
parallel fiber to P	CC
glycinergic synaps	CC
dopaminergic syn	CC
regulation of syna	BP
regulation of syna	BP
inositol 1,4,5-tris	MF
regulation of neu	BP
postsynaptic spec	BP
neurotransmitter	BP
calcium ion impo	BP
copper ion impor	BP
iron ion import a	BP
glucose import ac	BP
iron ion import a	BP
L-glutamate impo	BP
leucine import ac	BP
serine import acr	BP
sodium ion impor	BP

symmetric cell di\	BP
macromolecule d	BP
positive regulatio	BP
negative regulati	BP
import across pla	BP
cell-cell adhesion	BP
cell aggregation	BP
fast, calcium ion-(	BP
cerebellar neuror	BP
integral compone	CC
anchored compo	CC
cellular response	BP
cellular response	BP
FBXO family prot	MF
skin epidermis de	BP
positive regulatio	BP
response to mito	BP
ncRNA transcripti	BP
mRNA cleavage ir	BP
pre-mRNA cleava	BP
xenophagy	BP
postsynapse	CC
membrane protei	CC
plasma membran	CC
outer mitochondı	CC
inner mitochondr	CC
mRNA cap bindin	MF
nitrite reductase	MF
neurotransmitter	BP
nuclear chromosc	BP
spontaneous syn	BP
modulation of ex	BP
BMP receptor act	MF
endoplasmic retic	CC
intestinal folate a	BP
presynaptic endo	CC
presynaptic activ	CC
presynaptic endo	CC
presynaptic endo	CC
postsynaptic recy	CC
folate transmemk	BP
postsynaptic den	CC
protein transport	BP
postsynaptic earl	CC
postsynaptic end	CC

postsynaptic endo	CC
sequence-specific	MF
cellular detoxifica	BP
extrinsic compon	CC
HCN channel com	CC
intestinal lipid ab	BP
membrane micro	CC
actin-based cell p	CC
bone growth	BP
cellular oxidant d	BP
postsynaptic actin	CC
G protein-couple	MF
epididymosome	CC
neurotransmitter	BP
maintenance of p	BP
structural constit	MF
synapse pruning	BP
modification of p	BP
modification of d	BP
neurotransmitter	BP
extrinsic compon	CC
extrinsic compon	CC
extrinsic compon	CC
extrinsic compon	CC
extrinsic compon	CC
regulation of acti	BP
regulation of card	BP
regulation of mem	BP
regulation of mem	BP
regulation of AV r	BP
regulation of bun	BP
regulation of Purk	BP
regulation of SA n	BP
regulation of neu	BP
regulation of card	BP
regulation of atria	BP
regulation of vent	BP
membrane depol	BP
membrane repola	BP
membrane repola	BP
structural constit	MF
structural constit	MF
retrograde trans-	BP
retrograde trans-	BP
retrograde trans-	BP

axonal transport	BP
anterograde denc	BP
dendritic transpo	BP
retrograde trans-	BP
neurotransmitter	BP
anterograde axor	BP
retrograde axona	BP
regulation of post	BP
dendritic transpo	BP
perisynaptic extra	CC
exocytic insertion	BP
neurotransmitter	BP
neurotransmitter	BP
postsynaptic neur	BP
anterograde denc	BP
anterograde denc	BP
structural constitu	MF
postsynaptic actin	BP
excitatory chemic	BP
inhibitory chemic	BP
glutamatergic syn	CC
cholinergic synap	CC
GABA-ergic synap	CC
symmetric, GABA	CC
neuron to neuron	CC
asymmetric, gluta	CC
NMDA selective g	BP
neuronal dense c	CC
anchored compon	CC
extrinsic compon	CC
extrinsic compon	CC
vesicle-mediated	BP
calmodulin depen	BP
modification of p	BP
neuronal dense c	BP
neuronal dense c	CC
neuronal dense c	CC
perinuclear endo	CC
vesicle tethering	CC
anchored compon	CC
anchored compon	CC
anchored compon	CC
anchored compon	CC
anchored compon	CC
ceramide floppas	MF

sphingolipid trans	BP
ceramide transloc	BP
vesicle tethering	BP
vesicle tethering	BP
vesicle scission	BP
activating signal c	CC
presynapse assen	BP
integral compone	CC
integral compone	CC
integral compone	CC
integral compone	CC
integral compone	CC
integral compone	CC
integral compone	CC
postsynapse asse	BP
regulation of post	BP
mitochondrion-de	CC
mitochondrion to	BP
histone-depende	MF
BORC complex	CC
anterograde axor	BP
postsynaptic spec	CC
postsynaptic dens	CC
calcium export fro	BP
potassium chann	MF
ion channel regul	MF
microtubule-base	BP
chromosome, sub	CC
tRNA 5'-end proc	BP
RNA polymerase	MF
anchored compo	CC
extrinsic compon	CC
regulation of post	BP
regulation of post	BP
regulation of post	BP
regulation of neu	BP
serotonergic syna	CC
cell-cell signaling	BP
regulation of recy	BP
regulation of moc	BP
postsynaptic inte	CC
postsynaptic moc	BP
presynaptic modu	BP
presynapse orgar	BP
postsynapse orga	BP

regulation of pres	BP
regulation of post	BP
regulation of retr	BP
regulation of syna	BP
zinc ion import in	BP
structural constit	MF
presynaptic intern	CC
trans-synaptic sig	BP
structural constit	MF
postsynaptic inte	BP
structural constit	MF
postsynaptic spec	CC
caveola neck	CC
calcium-depende	BP
secretory vesicle	CC
synaptic vesicle c	BP
regulation of pres	BP
ligand-gated ion c	MF
voltage-gated ion	MF
regulation of pres	BP
supramolecular fi	CC
vesicle cytoskelet	BP
dense core granu	BP
ion antiporter act	MF
presynaptic cytos	CC
postsynaptic cyto	CC
presynaptic dens	BP
presynapse to nu	BP
postsynapse to nu	BP
neurotransmitter	MF
G protein-couple	MF
positive regulatio	BP
calcium ion bindi	MF
synaptic signaling	BP
trans-synaptic sig	BP
perisynaptic spac	CC
trans-synaptic sig	BP
protein catabolic	BP
regulation of tran	BP
trans-synaptic sig	BP
trans-synaptic sig	BP
trans-synaptic sig	BP
trans-synaptic sig	BP
trans-synaptic sig	BP
maintenance of s	BP

synaptic membra	BP
maintenance of p	BP
modification of sy	BP
modification of sy	BP
regulation of post	BP
calcium ion bindi	MF
cytoplasmic regio	CC
presynaptic cytos	CC
postsynaptic spec	CC
regulation of prot	BP
regulation of prot	BP
regulation of tran	BP
regulation of tran	BP
G protein-couple	MF
ion antiporter act	MF
neurotransmitter	MF
neurotransmitter	MF
neurotransmitter	BP
regulation of neu	BP
ligand-gated calci	MF
microtubule plus-	BP
lateral attachmer	BP
regulation of acti	BP
microtubule later	MF
protein localizatio	BP
matrix side of mit	CC
ventricular cardia	BP
voltage-gated cal	MF
neurotransmitter	BP
postsynaptic spec	CC
postsynaptic neu	BP
postsynaptic endo	CC
postsynaptic spec	CC
voltage-gated cal	MF
neurotransmitter	BP
endosome to plas	BP
neurotransmitter	BP
anterograde axor	BP
retrograde axona	BP
neurotransmitter	BP
integral compone	CC
induction of syna	BP
cell cortex region	CC
negative regulatio	BP
positive regulatio	BP

ficolin-1-rich gran	CC
cytolytic granule	CC
ubiquitinyl hydrol	MF
protein histidine	MF
estrogen 16-alpha	MF
estrogen 2-hydro	MF
tRNA-guanine tra	BP
chaperone compl	CC
acyl-L-homoserin	MF
proline dipeptida	MF
fatty acid omega-	MF
isobutyryl-CoA:FA	MF
beta,beta-carote	MF
oleamide hydrola	MF
L-dopa O-methylt	MF
5-diphosphoinosi	MF
homocarnosine s	MF
hypoxia-inducible	MF
laurate hydroxyla	MF
ceramidase activi	MF
3-oxo-glutaryl-[ac	MF
3-oxo-pimeloyl-[a	MF
heparan sulfate N	MF
N-acetyl-beta-D-g	MF
very-long-chain 3	MF
[protein]-3-O-(N-a	MF
[protein]-3-O-(N-a	MF
3-beta-hydroxyst	MF
protein-ribulosam	MF
protein-fructosan	MF
N-acetylphosphat	MF
very long chain fa	MF
linear malto-oligo	MF
1,3-diacylglycerol	MF
1,2-diacylglycerol	MF
tRNA-dihydrourid	MF
lecithin:11-cis ret	MF
1,8-cineole 2-exo	MF
3-oxo-arachidoyl-	MF
3-oxo-cerotoyl-Co	MF
3-oxo-lignocerony	MF
3-oxo-arachidoyl-	MF
3-oxo-behenoyl-C	MF
3-oxo-lignoceroyl	MF
3-oxo-cerotoyl-Co	MF

3-hydroxy-arachi	MF
3-hydroxy-behen	MF
3-hydroxy-lignoce	MF
11-cis-retinol deh	MF
polyprenol reduc	MF
mycophenolic aci	MF
decanoate-CoA li	MF
sn-1-glycerol-3-p	MF
dATP phosphohy	MF
dCTP phosphohy	MF
dUTP phosphohy	MF
dTTP phosphohy	MF
GTP phosphohyd	MF
8-oxo-dGTP phos	MF
dGTP phosphohy	MF
SHG alpha-glucan	MF
beta-maltose 4-a	MF
tRNA-4-demethyl	MF
tRNA 4-demethyl	MF
tRNAPhe (7-(3-an	MF
phosphatidyl pho	MF
lipoyl synthase a	MF
lipoyl synthase a	MF
octanoyl transfer	MF
protein-(glutamir	MF
phospholipase A2	MF
phospholipase A2	MF
[protein]-3-O-(N-	MF
malonate-semial	MF
GDP-Man:Man2G	MF
S-adenosyl-L-met	MF
myo-inositol-1,2,	MF
UDP-alpha-D-glu	MF
1,4-alpha-glucan	MF
very-long-chain 3	MF
NADPH phosphat	MF
very-long-chain e	MF
dihydroceramide	MF
dihydroceramide	MF
geranial:oxygen c	MF
heptaldehyde:ox	MF
molybdenum cof	MF
orcinol O-methyl	MF
hypoglycin A gam	MF
alcohol-forming f	MF

myristoyl-CoA hy(	MF
16-hydroxypalmi1	MF
alpha-amylase ac	MF
fructose-1-phosp	MF
methione N-acylt	MF
alanylglutamate (	MF
1-ethyladenine d(	MF
4alpha-carboxy-4	MF
4alpha-carboxy-5	MF
leukotriene C4 ga	MF
17-hydroxyproge:	MF
anandamide amic	MF
intestinal hexose	BP
amyloid-beta con	CC
tRNA (guanine-N7	BP
RNA 5'-cap (guan	BP
cytoskeletal prot(	MF
2-oxoglutaramat(	MF
regulation of infl	BP
negative regulati(	BP
positive regulatio	BP
phosphatidylinosi	MF
phosphatidylinosi	MF
positive regulatio	BP
neuron projectior	BP
neuron projectior	BP
tRNA pseudouridi	MF
neuron projectior	BP
protein maturatic	BP
protein maturatic	BP
guanine deglycati	BP
guanine deglycati	BP
guanine deglycati	BP
polyamine deace1	BP
spermidine deace	BP
regulation of cell	BP
tRNA 2'-O-methyl	MF
regulation of calc	BP
regulation of cob	BP
SUMO ligase com	CC
regulation of ade	BP
positive regulatio	BP
negative regulati(	BP
dolichyl pyrophos	MF
aminoacyl-tRNA r	BP

histone succinyla	BP
histone succinyltr	MF
negative regulatio	BP
positive regulatio	BP
glial cell projectio	BP
ER-dependent pe	BP
Ala-tRNA(Thr) hy	MF
positive regulatio	BP
positive regulatio	BP
IkappaB kinase co	MF
Sec61 translocon	MF
P-TEFb complex b	MF
phosphorylated h	MF
mRNA N-acetyltra	MF
cyclic-GMP-AMP	MF
tRNA C3-cytosine	BP
peptide 2-hydrox	MF
peptidyl-lysine gl	BP
peptide glutaryltr	MF
histone glutaryltr	MF
protein depropio	BP
protein-propiony	MF
ceramide-1-phos	MF
L-serine-phospha	MF
hydroperoxy icos	MF
hydroperoxy icos	MF
L-serine-phospha	MF
NA	NA
NA	NA
NA	NA
NA	NA
NA	NA
NA	NA
NA	NA
NA	NA
NA	NA
NA	NA
NA	NA
NA	NA
NA	NA
NA	NA
NA	NA
NA	NA
NA	NA
NA	NA

NA	NA
NA	NA
NA	NA
NA	NA
NA	NA
NA	NA
NA	NA
NA	NA
NA	NA
NA	NA
NA	NA
NA	NA
NA	NA
NA	NA
NA	NA
NA	NA
NA	NA
NA	NA
NA	NA
ncRNA deadenyla	BP
positive regulatio	BP
positive regulatio	BP
DNA strand resec	BP
regulation of DNA	BP
positive regulatio	BP
deaminated gluta	MF
metabolite repair	BP
regulation of acti	BP
negative regulatio	BP
regulation of angi	BP
cellularization cle	CC
negative regulatio	BP
vesicle-mediated	BP
positive regulatio	BP
negative regulatio	BP
cellular detoxifica	BP
regulation of calc	BP
negative regulatio	BP
mRNA alternative	BP
positive regulatio	BP
Norrin signaling p	BP
positive regulatio	BP
RNA NAD-cap (NA	MF
RNA NAD-cap (NM	MF
NAD-cap decappi	BP

apical plasma me	CC
intermembrane s	BP
lipid transfer acti\	MF
phospholipid trar	MF
sterol transfer act	MF
ceramide transfer	MF
phosphatidylchol	MF
plasma membran	CC
positive regulatio	BP
regulation of plas	BP
plasma membran	BP
positive regulatio	BP
negative regulatic	BP
stereocilium shaf	CC
stereocilium base	CC
stereocilium mair	BP
U6 snRNA (adenir	MF
snRNA (adenine-I	BP
ribitol beta-1,4-x)	MF
positive regulatio	BP
negative regulatic	BP
positive regulatio	BP
positive regulatio	BP
cell adhesion invc	BP
crotonyl-CoA hyd	MF
negative regulatic	BP
vacuole-isolation	CC
centriolar subdist	CC
DNA-3'-diphosph	MF
Sm-like protein fa	CC
Lsm2-8 complex	CC
T cell meandering	BP
proximal portion	CC
distal portion of a	CC
sulfatide binding	MF
Formylglycine-ge	MF
calcium-depende	MF
positive regulatio	BP
intraciliary transp	MF
regulation of cold	BP
negative regulatic	BP
detection of cold	BP
intraciliary transp	MF
positive regulatio	BP
regulation of bile	BP

positive regulatio	BP
tight junction ass	BP
tight junction org	BP
mucociliary cleara	BP
cone photorecep	CC
rod photorecepto	CC
photoreceptor di	CC
proacrosomal ves	BP
sperm head-tail c	CC
subapical part of	CC
basal body patch	CC
regulation of blas	BP
larynx morphoge	BP
NA	NA
NA	NA
NA	NA
NA	NA
NA	NA
NA	NA
NA	NA
NA	NA
NA	NA
NA	NA
NA	NA
NA	NA
NA	NA
NA	NA
NA	NA
NA	NA
KICSTOR complex	CC
L-aspartate impo	BP
D-aspartate trans	MF
meiotic nuclear d	BP
mitotic nuclear di	BP
regulation of cyto	BP
mitochondrial AD	BP
modification-dep	MF
phosphorylation-	MF
ubiquitin-depend	MF
sumo-dependent	MF
cell-cell adhesion	BP
cellular detoxifica	BP
lipid droplet form	BP
cellular response	BP
organelle localiza	BP

neuron projectio	BP
dendrite arboriza	BP
peptide butyryltr	MF
peptidyl-lysine cr	BP
peptidyl-lysine bu	BP
histone crotonylt	MF
histone butyryltr	MF
cardiac endotheli	BP
class I DNA-(apuri	MF
glycosylated regi	MF
membrane curva	MF
interleukin-10-me	BP
extracellular vesi	BP
export across pla	BP
ammonium impo	BP
monocarboxylate	MF
negative regulati	BP
negative regulati	BP
dipeptide import	BP
tripeptide import	BP
regulation of long	BP
positive regulatio	BP
general transcrip	MF
cell-cell fusion	BP
mitochondrial pr	MF
BCOR complex	CC
mRNA cap bindin	MF
endoplasmic reti	CC
exogenous protei	MF
MIB complex	CC
endoplasmic reti	CC
endosome fission	BP
protein mono-AD	BP
peptidyl-serine A	BP
peptidyl-glutama	BP
ADP-ribosylserine	MF
ADP-ribosylgluta	MF
NAD DNA ADP-ri	MF
general transcrip	MF
DNA-binding tran	MF
endocytic iron im	BP
serine import int	BP
protein sequeste	MF
cargo adaptor ac	MF
protein transport	MF

ATPase-coupled i	MF
flippase activity	MF
floppase activity	MF
lysophospholipid	BP
aminophospholip	BP
diacylglyceride tr	MF
sphingomyelin tra	MF
phosphatidylglyce	MF
cerebroside trans	MF
phosphatidylserir	MF
triglyceride transf	MF
phosphatidylchol	MF
phosphatidylserir	MF
N-retinylidene-ph	MF
lysophosphatidyl	MF
glycosylceramide	MF
ABC-type transme	MF
cyclic-GMP-AMP	MF
cyclic-GMP-AMP	BP
zinc:bicarbonate	MF
DNA-binding tran	MF
protein-cysteine S	MF
cytoskeleton-nuc	MF
cytokine precurso	BP
signaling recepto	BP
protein targeting	BP
NA	NA
NA	NA
NA	NA
NA	NA
NA	NA
NA	NA
NA	NA
NA	NA
NA	NA
NA	NA
NA	NA
NA	NA
NA	NA
NA	NA
NA	NA
NA	NA
NA	NA
NA	NA
NA	NA

NA	NA
NA	NA
NA	NA
NA	NA
NA	NA
primary dendrite	CC
distal dendrite	CC
regulation of spoı	BP
clathrin-depende	BP
regulation of neu	BP
positive regulatio	BP
negative regulatic	BP
apical distal dend	CC
basal dendrite de	BP
basal dendrite mc	BP
basal dendrite arl	BP
oxidised low-dens	BP
oxidised low-dens	MF
positive regulatio	BP
negative regulatic	BP
distal axon	CC
postsynaptic Golg	CC
amylin receptor c	CC
amylin receptor c	CC
amylin receptor c	CC
complement-mec	BP
vertebrate eye-sp	BP
positive regulatio	BP
positive regulatio	BP
neuroinflammato	BP
regulation of neu	BP
positive regulatio	BP
negative regulatic	BP
multiple spine syı	BP
amyloid-beta clea	BP
amyloid-beta clea	BP
glial cell-neuron s	BP
neuron-glial cell s	BP
regulation of micı	BP
negative regulatic	BP
reactive gliosis	BP
transport across l	BP
protein localizatic	BP
regulation of prot	BP
positive regulatio	BP

negative regulatio	BP
regulation of tran	BP
cell-substrate jun	BP
regulation of cell-	BP
negative regulatio	BP
positive regulatio	BP
positive regulatio	BP
positive regulatio	BP
regulation of pho	BP
regulation of pho	BP
regulation of pho	BP
cell-cell signaling	BP
negative regulatio	BP
positive regulatio	BP
positive regulatio	BP
regulation of cyto	BP
negative regulatio	BP
positive regulatio	BP
positive regulatio	BP
regulation of D-er	BP
regulation of subs	BP
positive regulatio	BP
regulation of ruffl	BP
negative regulatio	BP
positive regulatio	BP
regulation of cellu	BP
negative regulatio	BP
positive regulatio	BP
regulation of cellu	BP
negative regulatio	BP
positive regulatio	BP
regulation of prot	BP
negative regulatio	BP
regulation of hem	BP
negative regulatio	BP
positive regulatio	BP
negative regulatio	BP
positive regulatio	BP
negative regulatio	BP
regulation of pero	BP
regulation of cellu	BP
positive regulatio	BP
regulation of cellu	BP
negative regulatio	BP
positive regulatio	BP

regulation of argi	BP
negative regulatic	BP
negative regulatic	BP
positive regulatio	BP
positive regulatio	BP
regulation of dosa	BP
negative regulatic	BP
positive regulatio	BP
regulation of end	BP
negative regulatic	BP
positive regulatio	BP
positive regulatio	BP
regulation of nod	BP
negative regulatic	BP
negative regulatic	BP
regulation of histo	BP
negative regulatic	BP
positive regulatio	BP
extracellular nega	BP
negative regulatic	BP
positive regulatio	BP
regulation of rece	BP
negative regulatic	BP
positive regulatio	BP
regulation of hyal	BP
negative regulatic	BP
positive regulatio	BP
negative regulatic	BP
negative regulatic	BP
positive regulatio	BP
negative regulatic	BP
negative regulatic	BP
negative regulatic	BP
positive regulatio	BP
negative regulatic	BP
positive regulatio	BP
positive regulatio	BP
negative regulatic	BP
negative regulatic	BP
positive regulatio	BP
positive regulatio	BP
nodal signaling pa	BP
positive regulatio	BP
regulation of nod	BP
negative regulatic	BP

regulation of prot	BP
negative regulatio	BP
positive regulatio	BP
negative regulatio	BP
negative regulatio	BP
positive regulatio	BP
regulation of carc	BP
positive regulatio	BP
negative regulatio	BP
negative regulatio	BP
negative regulatio	BP
semaphorin-plexi	BP
regulation of amy	BP
negative regulatio	BP
positive regulatio	BP
positive regulatio	BP
regulation of NLR	BP
negative regulatio	BP
positive regulatio	BP
negative regulatio	BP
negative regulatio	BP
regulation of syna	BP
negative regulatio	BP
positive regulatio	BP
negative regulatio	BP
negative regulatio	BP
positive regulatio	BP
regulation of long	BP
negative regulatio	BP
positive regulatio	BP
negative regulatio	BP
regulation of prot	BP
negative regulatio	BP
positive regulatio	BP
regulation of mRN	BP
negative regulatio	BP
positive regulatio	BP
negative regulatio	BP
positive regulatio	BP
regulation of syna	BP
regulation of cellu	BP
negative regulatio	BP
regulation of defe	BP
negative regulatio	BP
positive regulatio	BP

regulation of glut	BP
negative regulatic	BP
positive regulatio	BP
regulation of long	BP
negative regulatic	BP
positive regulatio	BP
negative regulatic	BP
regulation of diac	BP
palmitic acid bios	BP
regulation of puri	BP
positive regulatio	BP
positive regulatio	BP
negative regulatic	BP
positive regulatio	BP
positive regulatio	BP
regulation of p38	BP
positive regulatio	BP
regulation of vasc	BP
negative regulatic	BP
positive regulatio	BP
doxorubicin trans	BP
regulation of mer	BP
positive regulatio	BP
negative regulatic	BP
negative regulatic	BP
ubiquinone-6 bios	BP
regulation of pota	BP
negative regulatic	BP
positive regulatio	BP
regulation of calc	BP
negative regulatic	BP
positive regulatio	BP
regulation of mitc	BP
negative regulatic	BP
positive regulatio	BP
regulation of resp	BP
sarcosine metabc	BP
sarcosine catabol	BP
regulation of eng	BP
positive regulatio	BP
regulation of rela	BP
negative regulatic	BP
benzylpenicillin m	BP
regulation of autc	BP
negative regulatic	BP

positive regulatio	BP
gentamycin meta	BP
carbohydrate der	BP
carbohydrate der	BP
carbohydrate der	BP
insulin metabolic	BP
insulin catabolic p	BP
regulation of trop	BP
negative regulatio	BP
positive regulatio	BP
neural crest cell n	BP
regulation of ERB	BP
negative regulatio	BP
positive regulatio	BP
regulation of eph	BP
positive regulatio	BP
negative regulatio	BP
regulation of extr	BP
negative regulatio	BP
positive regulatio	BP
regulation of hea	BP
negative regulatio	BP
negative regulatio	BP
regulation of neu	BP
negative regulatio	BP
positive regulatio	BP
regulation of NIK/	BP
negative regulatio	BP
positive regulatio	BP
negative regulatio	BP
positive regulatio	BP
positive regulatio	BP
negative regulatio	BP
(R)-carnitine trans	MF
positive regulatio	BP
negative regulatio	BP
positive regulatio	BP
nucleotide-excisio	BP
regulation of mac	BP
positive regulatio	BP
carbohydrate der	BP
negative regulatio	BP
nucleoside phosp	BP
negative regulatio	BP
positive regulatio	BP

negative regulatio	BP
positive regulatio	BP
regulation of carg	BP
negative regulatio	BP
positive regulatio	BP
positive regulatio	BP
negative regulatio	BP
regulation of flag	BP
negative regulatio	BP
regulation of odo	BP
positive regulatio	BP
regulation of stor	BP
negative regulatio	BP
positive regulatio	BP
regulation of vasc	BP
negative regulatio	BP
response to rapar	BP
organic cyclic con	BP
heterocyclic comp	MF
lipid hydroperoxi	BP
acetate ester trar	BP
regulation of pota	BP
negative regulatio	BP
positive regulatio	BP
regulation of chol	BP
negative regulatio	BP
regulation of volt	BP
negative regulatio	BP
positive regulatio	BP
regulation of tran	BP
negative regulatio	BP
positive regulatio	BP
positive regulatio	BP
regulation of tran	BP
regulation of pho	BP
response to benz	BP
azole transmemb	MF
pyruvate transme	BP
oleate transmem	MF
positive regulatio	BP
negative regulatio	BP
positive regulatio	BP
regulation of cyst	BP
positive regulatio	BP
negative regulatio	BP

positive regulatio	BP
positive regulatio	BP
regulation of hem	BP
negative regulatio	BP
positive regulatio	BP
negative regulatio	BP
positive regulatio	BP
response to paclit	BP
response to fenof	BP
response to metf	BP
response to camp	BP
organonitrogen c	BP
negative regulatio	BP
positive regulatio	BP
regulation of acid	BP
dendritic microtu	CC
response to capsa	BP
alpha-amino acid	BP
positive regulatio	BP
phosphatidylglyce	MF
cardiolipin bindin	MF
regulation of smo	BP
negative regulatio	BP
positive regulatio	BP
regulation of lym	BP
negative regulatio	BP
cellular response	BP
regulation of post	BP
positive regulatio	BP
XTP binding	MF
ITP binding	MF
nucleoside transm	BP
positive regulatio	BP
response to pepti	BP
cellular response	BP
calcium ion expor	BP
quinone metabol	BP
quinone catabolic	BP
positive regulatio	BP
regulation of supe	BP
positive regulatio	BP
regulation of mito	BP
negative regulatio	BP
positive regulatio	BP
nucleotide transm	BP

arsenate ion tran	BP
glutathione deriv	BP
cannabinoid biosy	BP
response to nitro	BP
mesenchymal cel	BP
leptomycin B binc	MF
negative regulatic	BP
positive regulatio	BP
regulation of hist	BP
negative regulatic	BP
positive regulatio	BP
positive regulatio	BP
positive regulatio	BP
leukotriene D4 bi	BP
leukotriene A4 m	BP
leukotriene A4 bi	BP
regulation of sign	BP
negative regulatic	BP
positive regulatio	BP
positive regulatio	BP
beta-glucoside ca	BP
beta-carotene me	BP
zeaxanthin biosyr	BP
positive regulatio	BP
regulation of tran	BP
negative regulatic	BP
positive regulatio	BP
regulation of high	BP
negative regulatic	BP
positive regulatio	BP
regulation of cell	BP
negative regulatic	BP
negative regulatic	BP
positive regulatio	BP
regulation of mit	BP
positive regulatio	BP
negative regulatic	BP
positive regulatio	BP
regulation of pos	BP
negative regulatic	BP
negative regulatic	BP
regulation of cell	BP
positive regulatio	BP
negative regulatic	BP
positive regulatio	BP

regulation of rela	BP
negative regulatio	BP
positive regulatio	BP
diadenosine pent	BP
diadenosine hexa	BP
adenosine 5'-(hex	BP
positive regulatio	BP
positive regulatio	BP
S-adenosyl-L-met	BP
positive regulatio	BP
positive regulatio	BP
regulation of inwa	BP
positive regulatio	BP
phosphatidylinosi	MF
negative regulatio	BP
positive regulatio	BP
response to ketar	BP
regulation of cell	BP
positive regulatio	BP
regulation of mito	BP
negative regulatio	BP
positive regulatio	BP
negative regulatio	BP
toxin transport	BP
homogentisate ca	BP
regulation of amy	BP
positive regulatio	BP
negative regulatio	BP
regulation of ciliu	BP
negative regulatio	BP
L-arginine transpo	BP
regulation of NAD	BP
regulation of hem	BP
positive regulatio	BP
regulation of hem	BP
negative regulatio	BP
positive regulatio	BP
regulation of extr	BP
negative regulatio	BP
positive regulatio	BP
regulation of Fas	BP
negative regulatio	BP
polyamine transm	BP
regulation of tran	BP
response to L-glu	BP

positive regulatio	BP
positive regulatio	BP
response to salt	BP
positive regulatio	BP
negative regulatio	BP
positive regulatio	BP
negative regulatio	BP
positive regulatio	BP
calcitriol binding	MF
regulation of mito	BP
negative regulatio	BP
positive regulatio	BP
regulation of orga	BP
positive regulatio	BP
lithocholic acid bi	MF
regulation of resp	BP
positive regulatio	BP
positive regulatio	BP
regulation of intri	BP
negative regulatio	BP
positive regulatio	BP
cellular response	BP
regulation of kera	BP
negative regulatio	BP
positive regulatio	BP
regulation of oxid	BP
negative regulatio	BP
positive regulatio	BP
fibroblast growth	BP
regulation of vira	BP
negative regulatio	BP
positive regulatio	BP
regulation of hep	BP
negative regulatio	BP
positive regulatio	BP
negative regulatio	BP
negative regulatio	BP
positive regulatio	BP
negative regulatio	BP
positive regulatio	BP
ketone body met	BP
negative regulatio	BP
positive regulatio	BP
negative regulatio	BP
positive regulatio	BP

regulation of posi	BP
negative regulatio	BP
regulation of end	BP
negative regulatio	BP
positive regulatio	BP
negative regulatio	BP
regulation of intri	BP
negative regulatio	BP
positive regulatio	BP
regulation of apo	BP
negative regulatio	BP
regulation of dela	BP
negative regulatio	BP
positive regulatio	BP
apoptotic process	BP
apoptotic process	BP
negative regulatio	BP
positive regulatio	BP
(R)-carnitine tran	BP
D3 vitamins bindi	MF
regulation of chro	BP
voltage-gated pot	MF
negative regulatio	BP
semaphorin-plexi	BP
semaphorin-plexi	BP
regulation of sodi	BP
negative regulatio	BP
positive regulatio	BP
negative regulatio	BP
positive regulatio	BP
regulation of cop	BP
positive regulatio	BP
positive regulatio	BP
oxaloacetate(2-) t	BP
sulfate transmem	BP
melanocyte apop	BP
positive regulatio	BP
negative regulatio	BP
negative regulatio	BP
negative regulatio	BP
VEGF-activated n	BP
chemoattractant	MF
positive regulatio	BP
ceramide 1-phosp	MF
ceramide 1-phosp	MF

ceramide 1-phos	BP
protein localizatio	BP
mitotic cytokineti	BP
regulation of mito	BP
protein localizatio	BP
regulation of mRI	BP
positive regulatio	BP
positive regulatio	BP
deactivation of m	BP
negative regulatio	BP
positive regulatio	BP
protein localizatio	BP
negative regulatio	BP
riboflavin binding	MF
regulation of mito	BP
negative regulatio	BP
positive regulatio	BP
positive regulatio	BP
protein localizatio	BP
positive regulatio	BP
regulation of prot	BP
positive regulatio	BP
L-alpha-amino aci	BP
chloride transmem	BP
protein localizatio	BP
cholangiocyte apo	BP
regulation of sper	BP
negative regulatio	BP
catalytic complex	CC
transmembrane t	CC
regulation of prot	BP
positive regulatio	BP
lysosomal HOPS c	CC
regulation of sign	BP
regulation of apo	BP
negative regulatio	BP
positive regulatio	BP
regulation of orga	BP
regulation of calc	BP
positive regulatio	BP
positive regulatio	BP
regulation of prot	BP
positive regulatio	BP
positive regulatio	BP
regulation of intra	BP

negative regulatic	BP
positive regulatio	BP
regulation of DNA	BP
positive regulatio	BP
negative regulatic	BP
positive regulatio	BP
serine/threonine	CC
3'-phospho-5'-ad	BP
H4 histone acetyl	CC
regulation of neu	BP
negative regulatic	BP
protein localizatic	BP
negative regulatic	BP
multi-organism in	BP
regulation of DNA	BP
proton transmem	BP
carnitine transme	BP
heterotrimeric G-	BP
negative regulatic	BP
positive regulatio	BP
negative regulatic	BP
acyl carnitine trar	BP
response to fluor	BP
cellular response	BP
regulation of neu	BP
positive regulatio	BP
assembly of large	BP
regulation of mRI	BP
regulation of mer	BP
negative regulatic	BP
1-phosphatidyl-1I	BP
kinociliary basal k	CC
calcium ion impo	BP
positive regulatio	BP
regulation of axo	BP
positive regulatio	BP
negative regulatic	BP
positive regulatio	BP
regulation of rece	BP
negative regulatic	BP
positive regulatio	BP
mitochondrial ou	BP
respiratory basal	BP
regulation of neu	BP
GABA receptor cc	CC

GABA-A receptor	CC
positive regulatio	BP
negative regulatio	BP
negative regulatio	BP
positive regulatio	BP
negative regulatio	BP
positive regulatio	BP
negative regulatio	BP
positive regulatio	BP
dendritic filopodi	CC
apoptotic process	BP
positive regulatio	BP
regulation of lens	BP
negative regulatio	BP
positive regulatio	BP
regulation of cell	BP
negative regulatio	BP
positive regulatio	BP
skeletal muscle sa	BP
positive regulatio	BP
GTPase activator	CC
late endosome to	BP
mitochondrial lar	BP
regulation of syna	BP
regulation of cell	BP
negative regulatio	BP
positive regulatio	BP
regulation of skel	BP
positive regulatio	BP
negative regulatio	BP
negative regulatio	BP
positive regulatio	BP
negative regulatio	BP
positive regulatio	BP
regulation of neu	BP
microtubule cytos	BP
negative regulatio	BP
positive regulatio	BP
propionyl-CoA ca	BP
propionyl-CoA bio	BP
regulation of retir	BP
positive regulatio	BP
regulation of resp	BP
negative regulatio	BP
positive regulatio	BP

protein localizatic	BP
negative regulatic	BP
positive regulatio	BP
terminal web ass	BP
regulation of post	BP
negative regulatic	BP
regulation of supi	BP
negative regulatic	BP
positive regulatio	BP
positive regulatio	BP
protein kinase co	CC
regulation of prot	BP
negative regulatic	BP
positive regulatio	BP
phosphatidylinosi	MF
inward rectifier p	CC
regulation of intra	BP
negative regulatic	BP
positive regulatio	BP
metalloendopept	MF
protein localizatic	BP
regulation of tau-	BP
negative regulatic	BP
positive regulatio	BP
regulation of den	BP
negative regulatic	BP
positive regulatio	BP
positive regulatio	BP
regulation of earl	BP
positive regulatio	BP
negative regulatic	BP
positive regulatio	BP
regulation of aspa	BP
negative regulatic	BP
positive regulatio	BP
negative regulatic	BP
positive regulatio	BP
mitotic DNA repli	BP
mitotic DNA repli	BP
mitotic DNA repli	BP
mitotic DNA repli	BP
DNA strand elong	BP
neurofibrillary tar	BP
mitotic telomere	BP
regulation of amy	BP

negative regulati	BP
positive regulatio	BP
positive regulatio	BP
negative regulati	BP
positive regulatio	BP
positive regulatio	BP
positive regulatio	BP
positive regulatio	BP
positive regulatio	BP
organelle disasse	BP
regulation of bon	BP
negative regulati	BP
positive regulatio	BP
positive regulatio	BP
regulation of RNA	BP
negative regulati	BP
regulation of ops	BP
positive regulatio	BP
positive regulatio	BP
regulation of resp	BP
positive regulatio	BP
regulation of leuk	BP
negative regulati	BP
positive regulatio	BP
positive regulatio	BP
protein localizati	BP
neural crest cell n	BP
negative regulati	BP
negative regulati	BP
positive regulatio	BP
regulation of extr	BP
negative regulati	BP
positive regulatio	BP
regulation of mel	BP
regulation of prot	BP
negative regulati	BP
positive regulatio	BP
positive regulatio	BP
regulation of ER-a	BP
negative regulati	BP
positive regulatio	BP
negative regulati	BP
positive regulatio	BP
positive regulatio	BP
regulation of prot	BP

negative regulatio	BP
protein localizatio	BP
1-phosphatidyl-1I	BP
regulation of mito	BP
positive regulatio	BP
urate homeostasi	BP
protein localizatio	BP
negative regulatio	BP
negative regulatio	BP
negative regulatio	BP
cupric ion binding	MF
cuprous ion bindi	MF
regulation of esta	BP
negative regulatio	BP
positive regulatio	BP
adrenomedullin r	CC
regulation of auto	BP
negative regulatio	BP
positive regulatio	BP
regulation of calc	BP
negative regulatio	BP
positive regulatio	BP
positive regulatio	BP
glyoxal metabolic	BP
glyoxal catabolic	BP
positive regulatio	BP
positive regulatio	BP
negative regulatio	BP
regulation of oxid	BP
negative regulatio	BP
regulation of hyd	BP
negative regulatio	BP
negative regulatio	BP
positive regulatio	BP
regulation of prot	BP
negative regulatio	BP
negative regulatio	BP
positive regulatio	BP
negative regulatio	BP
positive regulatio	BP
mRNA binding inv	MF
melanosome asse	BP
regulation of calc	BP
positive regulatio	BP
regulation of leuk	BP

negative regulati	BP
positive regulatio	BP
U2-type presplice	BP
negative regulati	BP
positive regulatio	BP
negative regulati	BP
multi-ciliated epi	BP
exon-exon juncti	BP
positive regulatio	BP
positive regulatio	BP
negative regulati	BP
positive regulatio	BP
positive regulatio	BP
positive regulatio	BP
regulation of pot	BP
positive regulatio	BP
protein localizati	BP
positive regulatio	BP
negative regulati	BP
positive regulatio	BP
regulation of pyr	BP
regulation of reg	BP
positive regulatio	BP
positive regulatio	BP
positive regulatio	BP
positive regulatio	BP
negative regulati	BP
positive regulatio	BP
response to dopa	BP
cellular response	BP
L-ornithine trans	BP
regulation of Gol	BP
protein localizati	BP
regulation of cell	BP
negative regulati	BP
positive regulatio	BP
regulation of end	BP
positive regulatio	BP
facioacoustic gan	BP
regulation of oxid	BP
negative regulati	BP
positive regulatio	BP
negative regulati	BP
negative regulati	BP
regulation of hor	BP

positive regulatio	BP
negative regulatic	BP
positive regulatio	BP
protein localizatic	BP
L-arginine transm	BP
L-lysine transmen	BP
negative regulatic	BP
positive regulatio	BP
reactive oxygen s	BP
cellular response	BP
response to glyco	BP
protein localizatic	BP
regulation of syna	BP
negative regulatic	BP
positive regulatio	BP
regulation of reac	BP
negative regulatic	BP
positive regulatio	BP
regulation of cell	BP
positive regulatio	BP
regulation of TOR	BP
negative regulatic	BP
positive regulatio	BP
positive regulatio	BP
protein localizatic	BP
response to lipoic	BP
negative regulatic	BP
protein transport	BP
positive regulatio	BP
lactate catabolic		BP
Okazaki fragment	BP
regulation of mitc	BP
negative regulatic	BP
positive regulatio	BP
response to dehy	BP
response to 11-de	BP
regulation of nucl	BP
negative regulatic	BP
positive regulatio	BP
phytanic acid met	BP
release of seques	BP
calcium ion trans	BP
positive regulatio	BP
regulation of bloc	BP
negative regulatic	BP

negative regulati	BP
positive regulatio	BP
negative regulati	BP
regulation of prot	BP
regulation of mei	BP
protein localizatic	BP
establishment of	BP
negative regulati	BP
positive regulatio	BP
cellular response	BP
protein localizatic	BP
regulation of grov	BP
regulation of extr	BP
positive regulatio	BP
negative regulati	BP
extracellular vesi	CC
regulation of prot	BP
negative regulati	BP
positive regulatio	BP
negative regulati	BP
positive regulatio	BP
positive regulatio	BP
negative regulati	BP
cornified envelop	BP
response to L-arg	BP
cellular response	BP
regulation of ATP	BP
negative regulati	BP
positive regulatio	BP
positive regulatio	BP
regulation of bloc	BP
negative regulati	BP
positive regulatio	BP
positive regulatio	BP
negative regulati	BP
positive regulatio	BP
positive regulatio	BP
protein localizatic	BP
negative regulati	BP
negative regulati	BP
positive regulatio	BP
negative regulati	BP
positive regulatio	BP
positive regulatio	BP
protein localizatic	BP

negative regulati	BP
positive regulatio	BP
positive regulatio	BP
positive regulatio	BP
negative regulati	BP
positive regulatio	BP
regulation of cha	BP
negative regulati	BP
positive regulatio	BP
regulation of com	BP
negative regulati	BP
regulation of spro	BP
negative regulati	BP
positive regulatio	BP
mitotic cleavage f	BP
positive regulatio	BP
positive regulatio	BP
negative regulati	BP
positive regulatio	BP
negative regulati	BP
tarsal gland devel	BP
substantia propri	BP
negative regulati	BP
regulation of hem	BP
negative regulati	BP
uterine gland dev	BP
spermine transm	BP
cysteine transme	BP
asparagine transr	BP
regulation of aero	BP
negative regulati	BP
positive regulatio	BP
negative regulati	BP
positive regulatio	BP
regulation of pho	BP
regulation of plas	BP
regulation of pho	BP
negative regulati	BP
positive regulatio	BP
negative regulati	BP
negative regulati	BP
negative regulati	BP
cortical microtub	CC
positive regulatio	BP
regulation of volt	BP

positive regulatio	BP
gap junction char	MF
regulation of pota	BP
negative regulatio	BP
positive regulatio	BP
sweet taste recep	CC
negative regulatio	BP
positive regulatio	BP
regulation of vira	BP
positive regulatio	BP
regulation of DNA	BP
protein localizatio	BP
negative regulatio	BP
regulation of glut	BP
positive regulatio	BP
guanine nucleotid	BP
regulation of proc	BP
negative regulatio	BP
positive regulatio	BP
L-leucine import a	BP
L-glutamine impo	BP
glycine import ac	BP
L-isoleucine impo	BP
L-histidine import	BP
L-serine import ac	BP
negative regulatio	BP
positive regulatio	BP
negative regulatio	BP
organic acid trans	BP
arginine transmer	BP
regulation of cellu	BP
negative regulatio	BP
positive regulatio	BP
magnesium ion tr	BP
positive regulatio	BP
cellular response	BP
cellular response	BP
negative regulatio	BP
regulation of aort	BP
positive regulatio	BP
regulation of crist	BP
positive regulatio	BP
negative regulatio	BP
positive regulatio	BP
positive regulatio	BP

positive regulatio	BP
positive regulatio	BP
regulation of ATF	BP
negative regulatio	BP
positive regulatio	BP
regulation of IRE1	BP
negative regulatio	BP
positive regulatio	BP
regulation of PER	BP
negative regulatio	BP
positive regulatio	BP
negative regulatio	BP
positive regulatio	BP
regulation of esta	BP
negative regulatio	BP
positive regulatio	BP
regulation of plas	BP
regulation of rece	BP
positive regulatio	BP
negative regulatio	BP
positive regulatio	BP
negative regulatio	BP
positive regulatio	BP
positive regulatio	BP
estradiol binding	MF
cellular response	BP
primary palate de	BP
response to sodiu	BP
cellular response	BP
response to acryl	BP
negative regulatio	BP
negative regulatio	BP
positive regulatio	BP
positive regulatio	BP
positive regulatio	BP
nitric-oxide synth	CC
monounsaturated	BP
positive regulatio	BP
negative regulatio	BP
positive regulatio	BP
regulation of mici	BP
negative regulatio	BP
positive regulatio	BP
enterobactin binc	MF
positive regulatio	BP

iron ion export ac	BP
negative regulatio	BP
positive regulatio	BP
negative regulatio	BP
positive regulatio	BP
negative regulatio	BP
cellular response	BP
response to Arocl	BP
cellular response	BP
response to Thyro	BP
cellular response	BP
epithelial cell apo	BP
negative regulatio	BP
positive regulatio	BP
negative regulatio	BP
regulation of colla	BP
negative regulatio	BP
positive regulatio	BP
negative regulatio	BP
positive regulatio	BP
negative regulatio	BP
positive regulatio	BP
negative regulatio	BP
response to aldos	BP
cellular response	BP
negative regulatio	BP
S-adenosyl-L-met	MF
negative regulatio	BP
positive regulatio	BP
regulation of chol	BP
positive regulatio	BP
negative regulatio	BP
positive regulatio	BP
regulation of loco	BP
regulation of catio	BP
positive regulatio	BP
presynaptic active	BP
positive regulatio	BP
negative regulatio	BP
positive regulatio	BP
positive regulatio	BP
protein localizatio	BP
positive regulatio	BP
axon cytoplasm	CC
cellular response	BP

positive regulatio	BP
phosphatidyletha	MF
microglial cell mi	BP
regulation of mic	BP
negative regulati	BP
positive regulatio	BP
positive regulatio	BP
regulation of retr	BP
negative regulati	BP
positive regulatio	BP
DN2 thymocyte d	BP
DN3 thymocyte d	BP
axonemal central	BP
DNA synthesis in	BP
positive regulatio	BP
regulation of adi	BP
negative regulati	BP
positive regulatio	BP
regulation of pyr	BP
positive regulatio	BP
negative regulati	BP
negative regulati	BP
positive regulatio	BP
positive regulatio	BP
iodide transmem	BP
negative regulati	BP
positive regulatio	BP
membrane prote	BP
negative regulati	BP
positive regulatio	BP
regulation of seri	BP
negative regulati	BP
positive regulatio	BP
negative regulati	BP
negative regulati	BP
positive regulatio	BP
positive regulatio	BP
positive regulatio	BP
pericyte cell diffe	BP
negative regulati	BP
negative regulati	BP
positive regulatio	BP
regulation of bile	BP
positive regulatio	BP
positive regulatio	BP

negative regulatio	BP
positive regulatio	BP
positive regulatio	BP
pyroptosome con	BP
L-proline import a	BP
L-alanine import a	BP
tricellular tight ju	BP
negative regulatio	BP
BAT3 complex bir	MF
negative regulatio	BP
positive regulatio	BP
regulation of ERA	BP
negative regulatio	BP
positive regulatio	BP
positive regulatio	BP
positive regulatio	BP
positive regulatio	BP
response to desip	BP
response to meth	BP
transmitter-gatec	MF
response to 2-O-a	BP
cellular response	BP
cellular response	BP
regulation of myc	BP
regulation of dop	BP
negative regulatio	BP
positive regulatio	BP
positive regulatio	BP
positive regulatio	BP
positive regulatio	BP
regulation of telo	BP
negative regulatio	BP
positive regulatio	BP
regulation of telo	BP
negative regulatio	BP
positive regulatio	BP
positive regulatio	BP
response to kaini	BP
regulation of prot	BP
maintenance of u	BP
protein localizatio	BP
endoplasmic retic	BP
Golgi apparatus n	BP
mannose trimmir	BP
response to sodiu	BP

cellular response	BP
response to L-phe	BP
rod bipolar cell di	BP
response to ciliar	BP
regulation of skel	BP
negative regulatic	BP
positive regulatio	BP
negative regulatic	BP
positive regulatio	BP
heparan sulfate b	MF
response to form	BP
positive regulatio	BP
melatonin bindin	MF
positive regulatio	BP
positive regulatio	BP
positive regulatio	BP
dehydrodolichyl c	CC
regulation of GTP	BP
negative regulatic	BP
positive regulatio	BP
positive regulatio	BP
negative regulatic	BP
negative regulatic	BP
positive regulatio	BP
positive regulatio	BP
positive regulatio	BP
negative regulatic	BP
positive regulatio	BP
negative regulatic	BP
folate import acr	BP
positive regulatio	BP
negative regulatic	BP
positive regulatio	BP
regulation of mat	BP
negative regulatic	BP
positive regulatio	BP
positive regulatio	BP
regulation of inte	BP
negative regulatic	BP
cellular response	BP
response to 17alp	BP
protein localizatic	BP
Ac-Asp-Glu bindir	MF
tetrahydrofolyl-p	MF
positive regulatio	BP

negative regulatio	BP
positive regulatio	BP
regulation of mici	BP
negative regulatio	BP
positive regulatio	BP
negative regulatio	BP
positive regulatio	BP
positive regulatio	BP
negative regulatio	BP
positive regulatio	BP
response to arach	BP
L-tryptophan trar	BP
L-alanine transme	BP
phosphatidylinosi	BP
cellular response	BP
cellular response	BP
negative regulatio	BP
negative regulatio	BP
cellular response	BP
cellular response	BP
positive regulatio	BP
cellular response	BP
cellular response	BP
regulation of prot	BP
positive regulatio	BP
negative regulatio	BP
serotonin-activat	CC
response to 2,3,7	BP
cellular response	BP
regulation of acti	BP
cellular response	BP
cellular response	BP
response to gluco	BP
positive regulatio	BP
cellular response	BP
cellular response	BP
response to meth	BP
response to curcu	BP
response to amyl	BP
cellular response	BP
response to roter	BP
regulation of fat c	BP
positive regulatio	BP
glucose transmer	BP
regulation of ubic	BP

negative regulatic	BP
positive regulatio	BP
ATP export	BP
regulation of som	BP
positive regulatio	BP
positive regulatio	BP
myo-inositol impc	BP
peptide transmer	MF
response to 3-me	BP
negative regulatic	BP
positive regulatio	BP
positive regulatio	BP
negative regulatic	BP
positive regulatio	BP
midbrain morpho	BP
negative regulatic	BP
positive regulatio	BP
regulation of prot	BP
negative regulatic	BP
positive regulatio	BP
regulation of grar	BP
positive regulatio	BP
beta-catenin dest	MF
regulation of cha|	BP
negative regulatic	BP
positive regulatio	BP
regulation of AMI	BP
positive regulatio	BP
negative regulatic	BP
tertiary granule lu	CC
negative regulatic	BP
negative regulatic	BP
vascular associate	BP
negative regulatic	BP
positive regulatio	BP
regulation of prot	BP
negative regulatic	BP
positive regulatio	BP
negative regulatic	BP
positive regulatio	BP
negative regulatic	BP
positive regulatio	BP
chaperone-media	BP
negative regulatic	BP
all-trans-retinol b	MF

response to tetra	BP
regulation of prot	BP
negative regulatio	BP
positive regulatio	BP
regulation of prot	BP
negative regulatio	BP
positive regulatio	BP
negative regulatio	BP
positive regulatio	BP
NLRP1 inflammas	BP
positive regulatio	BP
regulation of core	BP
negative regulatio	BP
positive regulatio	BP
negative regulatio	BP
regulation of tran	BP
positive regulatio	BP
rRNA acetylation	BP
ficolin-1-rich gran	CC
negative regulatio	BP
positive regulatio	BP
purine nucleobas	BP
protein localizatio	BP
positive regulatio	BP
positive regulatio	BP
dorsal root gangli	BP
beta-catenin-TCF	BP
TORC2 complex b	MF
response to nitro	BP
cellular response	BP
response to L-glu	BP
cellular response	BP
negative regulatio	BP
positive regulatio	BP
proteasome core	MF
proteasome regu	MF
excitatory synaps	BP
inhibitory synaps	BP
negative regulatio	BP
protein localizatio	BP
positive regulatio	BP
regulation of telo	BP
positive regulatio	BP
negative regulatio	BP
positive regulatio	BP

negative regulati	BP
positive regulatio	BP
response to hydr	BP
cellular response	BP
positive regulatio	BP
beta-catenin dest	BP
beta-catenin dest	BP
Wnt signalosome	BP
cranial skeletal sy	BP
positive regulatio	BP
negative regulati	BP
positive regulatio	BP
ESCRT complex di	BP
positive regulatio	BP
ESCRT III complex	BP
ESCRT III complex	BP
negative regulati	BP
positive regulatio	BP
negative regulati	BP
negative regulati	BP
negative regulati	BP
negative regulati	BP
positive regulatio	BP
coreceptor activit	MF
coreceptor activit	MF
MCM complex bi	MF
negative regulati	BP
planar cell polarit	BP
folate import intc	BP
midbrain dopami	BP
ATPase complex	CC
negative regulati	BP
positive regulatio	BP
Wnt signaling pat	BP
canonical Wnt sig	BP
planar cell polarit	BP
regulation of mid	BP
regulation of cytc	BP
positive regulatio	BP
brush border asse	BP
positive regulatio	BP
cellular response	BP
lymphatic endoth	BP
regulation of end	BP
positive regulatio	BP

glycine import int	BP
positive regulatio	BP
positive regulatio	BP
regulation of leuk	BP
negative regulatio	BP
positive regulatio	BP
regulation of mer	BP
negative regulatio	BP
positive regulatio	BP
positive regulatio	BP
negative regulatio	BP
voltage-gated ion	MF
regulation of mer	BP
negative regulatio	BP
autophagosome (	BP
carboxylic acid tra	BP
otic placode deve	BP
regulation of epit	BP
negative regulatio	BP
negative regulatio	BP
regulation of met	BP
negative regulatio	BP
positive regulatio	BP
positive regulatio	BP
calcium-transport	MF
calcium-transport	MF
calcium:cation an	MF
positive regulatio	BP
negative regulatio	BP
positive regulatio	BP
allantois develop	BP
cardiac jelly deve	BP
positive regulatio	BP
negative regulatio	BP
negative regulatio	BP
integral compone	CC
positive regulatio	BP
cell surface recep	BP
positive regulatio	BP
regulation of gluc	BP
positive regulatio	BP
biotin import acr	BP
eukaryotic transla	BP
response to acety	BP
cellular response	BP

lysosomal protei	BP
regulation of volt	BP
positive regulatio	BP
negative regulatio	BP
regulation of pha	BP
regulation of lyso	BP
negative regulatio	BP
positive regulatio	BP
positive regulatio	BP
positive regulatio	BP
RISC complex bin	MF
regulation of vas	BP
positive regulatio	BP
negative regulatio	BP
negative regulatio	BP
positive regulatio	BP
positive regulatio	BP
manchette assem	BP
manchette disass	BP
methylcrotonoyl-	CC
regulation of con	BP
positive regulatio	BP
positive regulatio	BP
negative regulatio	BP
positive regulatio	BP
positive regulatio	BP
positive regulatio	BP
atrioventricular c	BP
epicardium morp	BP
clathrin-coated p	BP
regulation of adh	BP
cellular response	BP
cellular response	BP
negative regulatio	BP
response to 3,3',5	BP
cellular response	BP
regulation of mo	BP
negative regulatio	BP
positive regulatio	BP
negative regulatio	BP
endonucleolytic c	BP
positive regulatio	BP
regulation of mo	BP
positive regulatio	BP
regulation of retr	BP

negative regulatio	BP
positive regulatio	BP
fibrous ring of he	BP
serine-type pepti	CC
regulation of CAN	BP
positive regulatio	BP
negative regulatio	BP
positive regulatio	BP
negative regulatio	BP
positive regulatio	BP
transforming grov	BP
semi-lunar valve	BP
inferior endocard	BP
positive regulatio	BP
telomerase holoe	BP
tracheoesophage	BP
positive regulatio	BP
regulation of gast	BP
positive regulatio	BP
negative regulatio	BP
positive regulatio	BP
prostaglandin cat	BP
endodeoxyribonu	CC
ciliary transition z	BP
spine apparatus a	BP
regulation of end	BP
negative regulatio	BP
peptidase comple	CC
serine-type endo	CC
ceramide phosph	BP
response to D-gal	BP
negative regulatio	BP
positive regulatio	BP
positive regulatio	BP
retromer comple:	MF
response to flavo	BP
cellular response	BP
negative regulatio	BP
positive regulatio	BP
positive regulatio	BP
negative regulatio	BP
regulation of den	BP
positive regulatio	BP
response to glycir	BP
cellular response	BP

positive regulatio	BP
non-canonical Wı	BP
positive regulatio	BP
positive regulatio	BP
negative regulatio	BP
positive regulatio	BP
regulation of mye	BP
negative regulatio	BP
regulation of lym	BP
negative regulatio	BP
positive regulatio	BP
negative regulatio	BP
negative regulatio	BP
canonical Wnt sig	BP
regulation of prot	BP
positive regulatio	BP
positive regulatio	BP
acetyl-CoA bindin	MF
positive regulatio	BP
negative regulatio	BP
non-motile cilium	BP
positive regulatio	BP
macrophage migr	BP
positive regulatio	BP
regulation of mac	BP
negative regulatio	BP
positive regulatio	BP
negative regulatio	BP
regulation of Golg	BP
positive regulatio	BP
positive regulatio	BP
polysome binding	MF
positive regulatio	BP
positive regulatio	BP
regulation of bloc	BP
positive regulatio	BP
negative regulatio	BP
positive regulatio	BP
ganglioside GM1	BP
ganglioside GM1	MF
ganglioside GM2	MF
ganglioside GM3	MF
ganglioside GT1b	MF
ganglioside GP1c	MF
positive regulatio	BP

positive regulatio	BP
outer hair cell ap	BP
positive regulatio	BP
fibronectin fibril	BP
resveratrol bindir	MF
negative regulati	BP
positive regulatio	BP
negative regulati	BP
positive regulatio	BP
negative regulati	BP
positive regulatio	BP
negative regulati	BP
positive regulatio	BP
regulation of pre:	BP
positive regulatio	BP
positive regulatio	BP
positive regulatio	BP
regulation of prot	BP
positive regulatio	BP
negative regulati	BP
positive regulatio	BP
proline import ac	BP
positive regulatio	BP
negative regulati	BP
positive regulatio	BP
regulation of calc	BP
positive regulatio	BP
negative regulati	BP
positive regulatio	BP
regulation of lyso	BP
negative regulati	BP
positive regulatio	BP
negative regulati	BP
positive regulatio	BP
regulation of plas	BP
positive regulatio	BP
regulation of poly	BP
positive regulatio	BP
negative regulati	BP
positive regulatio	BP
cellular response	BP
regulation of mit	BP
negative regulati	BP
positive regulatio	BP
response to phos	BP

negative regulati	BP
protein localizati	BP
cytoplasmic micr	CC
mitotic spindle as	CC
positive regulatio	BP
positive regulatio	BP
CCR4-NOT compl	MF
negative regulati	BP
negative regulati	BP
positive regulatio	BP
8-hydroxy-2'-deo	MF
regulation of DN	BP
negative regulati	BP
positive regulatio	BP
negative regulati	BP
positive regulatio	BP
negative regulati	BP
protein localizati	BP
cellular response	BP
regulation of intr	BP
regulation of cell	BP
regulation of chr	BP
positive regulatio	BP
regulation of pro	BP
positive regulatio	BP
negative regulati	BP
cellular response	BP
negative regulati	BP
regulation of cell	BP
positive regulatio	BP
negative regulati	BP
positive regulatio	BP
epididymis devel	BP
negative regulati	BP
positive regulatio	BP
negative regulati	BP
positive regulatio	BP
regulation of cell	BP
negative regulati	BP
negative regulati	BP
regulation of res	BP
positive regulatio	BP
regulation of am	BP
negative regulati	BP
positive regulatio	BP

regulation of calc	BP
negative regulati	BP
positive regulatio	BP
negative regulati	BP
positive regulatio	BP
negative regulati	BP
positive regulatio	BP
amyloid fibril forr	BP
inhibition of cyste	BP
methylglyoxal red	MF
IDP phosphatase	MF
granular vesicle	CC
neurosecretory v	CC
mitotic spindle m	CC
hippocampal mos	CC
vasomotion	BP
pericellular baske	CC
pinceau fiber	CC
parallel fiber	CC
calcium ion expor	BP
calcium ion impoi	BP
Lewy body core	CC
Lewy body coron	CC
protein localizatio	BP
stress-induced mi	BP
spindle matrix	CC
anterograde neur	BP
retrograde neuro	BP
phosphatidic acid	MF
activation of prot	BP
TRAPPI protein cc	CC
TRAPPII protein c	CC
TRAPPIII protein	CC
polyuridylation-d	BP
periciliary membr	CC
cartilage homeos	BP
lens fiber cell apo	BP
response to nerve	BP
cellular response	BP
calcium-depende	BP
protein linear deu	BP
spermatoproteas	CC
RQC complex	CC
RNA polymerase	BP
ribosome-associa	BP

B cell receptor ap	BP
messenger ribonu	BP
messenger ribonu	CC
GATOR1 complex	CC
Gtr1-Gtr2 GTPase	CC
epithelial cell apo	BP
linoleate 9S-lipox	MF
neuron projection	BP
intrinsic apoptoti	BP
maintenance of t	BP
talin binding	MF
histone H2A phos	BP
single-strand brea	MF
protein localizatio	BP
protein K27-linke	BP
protein K33-linke	BP
G protein-couple	BP
protein localizatio	BP
phosphodiesteras	MF
EH domain bindir	MF
mitochondrial tRI	BP
exosomal secretio	BP
amino acid transp	CC
peptide-serine-N-	MF
peptide-glutamat	MF
oxidoreductase c	CC
L-cysteine desulfu	CC
histone methyltra	MF
paranodal junctio	BP
transferase comp	CC
double-stranded	MF
steroid hormone	MF
histone kinase ac	MF
histone H2A-T12(	BP
uniplex complex	CC
N6-methyladenos	MF
regulation of tran	BP
cellular response	BP
keratin filament b	MF
signal clustering	BP
histone glutamine	BP
histone-glutamin	MF
pre-mRNA catabo	BP
peptidyl-tyrosine	BP
neutrophil migrat	BP

response to trans	BP
RNA polymerase	MF
preribosome binc	MF
RNA 5'-methyltra	MF
RNA localization t	BP
bub1-bub3 comp	CC
cellular response	BP
Atg1/ULK1 kinase	CC
Ire1 complex	CC
gap junction-med	BP
sumoylated E2 lig	CC
terminal web	CC
lipid transport acı	BP
Lys48-specific deı	MF
ubiquitin-specific	MF
hyaloid vascular p	BP
meiotic spindle m	CC
mitotic cleavage f	BP
protein K33-linke	BP
DNA repair comp	CC
3M complex	CC
mitochondrial rib	MF
embryonic lung d	BP
embryonic liver d	BP
embryonic brain (	BP
protein ADP-ribos	MF
protein antigen b	MF
CGRP receptor co	CC
calcitonin gene-re	MF
calcitonin gene-re	BP
adrenomedullin b	MF
adrenomedullin r	BP
replication-born (	BP
cellular response	BP
response to insuli	BP
glyoxalase (glycol	MF
RZZ complex	CC
ryanodine recept	CC
mitotic recombin	BP
miRNA transport	BP
peroxisomal impc	CC
extracellular matı	MF
upper tip-link der	CC
U6 2'-O-snRNA m	BP
MAP kinase serin	MF

positive regulatio	BP
negative regulatio	BP
intrinsic apoptoti	BP
peptidyl-threonin	BP
F-box domain bin	MF
U1 snRNP binding	MF
U2 snRNP binding	MF
exon-exon junctio	MF
linear polyubiquit	MF
cellular stress res	BP
Parkin-FBXW7-Cu	CC
L-type voltage-ga	CC
mitochondrion-er	BP
transferrin recept	MF
leptin receptor bi	MF
omegasome	CC
protein autosumo	BP
NuA3a histone ac	CC
NuA3b histone ac	CC
response to ultra	BP
mRNA pseudouri	BP
mitotic spindle m	CC
dense core granu	BP
dense core granu	BP
piRNA biosynthet	BP
CLOCK-BMAL trar	CC
single-stranded 3	MF
pyrimidine nucleo	BP
bone regeneratio	BP
glycosylphosphat	CC
phospholipid-trar	CC
neuron projectio	BP
fructose import a	BP
mitochondrial ma	BP
mitochondrial tra	BP
mitochondrial AT	BP
mitochondrial ph	BP
mitochondrial FA	BP
mitochondrial NA	BP
mitochondrial alp	BP
HSP90-CDC37 cha	CC
UDP-N-acetylgluc	BP
TERT-RMRP comp	CC
potassium ion im	BP
mitochondrial L-c	BP

perinuclear endo|	CC
peptidyl-serine tr	BP
phospholipase D	MF
cardiac Troponin	CC
ATF4-CREB1 trans	CC
ATF1-ATF4 transc	CC
protein K69-linke	BP
AIP1-IRE1 comple	CC
3' overhang single	MF
dark adaptation	BP
IRE1-TRAF2-ASK1	CC
mitochondrial me	BP
CHOP-ATF4 comp	CC
histone H3-K9 de	BP
CHOP-ATF3 comp	CC
IRE1-RACK1-PP2A	CC
proximal dendrite	CC
response to prola	BP
response to iron i	BP
cellular response	BP
sebum secreting	BP
AnxA2-p11 comp	CC
PCSK9-LDLR comp	CC
PCSK9-AnxA2 con	CC
vesicle fusion wit	BP
Golgi medial ciste	CC
Golgi trans cister	CC
histone H4-K16 d	BP
CSF1-CSF1R comp	CC
DNA double-strar	BP
USH2 complex	CC
protein depalmit	BP
palmitoleoyltrans	MF
palmitoleyl hydro	MF
nucleolar chroma	BP
integral compone	CC
cholangiocyte pro	BP
conditioned place	BP
presynaptic active	BP
HFE-transferrin re	CC
mRNA CDS bindin	MF
axonemal central	CC
axonemal central	CC
cytoplasmic perip	CC
Lsm1-7-Pat1 com	CC

VCP-NSFL1C comp	CC
UV-damage excis	BP
response to mang	BP
pseudouridine 5'-	MF
microvesicle	CC
protein sialylation	BP
primary miRNA m	BP
EARP complex	CC
cellular detoxifica	BP
microtubule end	CC
mitotic spindle m	BP
ubiquitin ligase-su	MF
ubiquitin ligase ac	MF
mitotic sister chro	BP
osmolarity-sensin	MF
growth cone lame	CC
arrestin family pr	MF
myofibroblast cor	BP
proximal neuron	CC
clathrin-depende	BP
response to angio	BP
lipoprotein partic	CC
protein localizatio	BP
cytoplasmic side o	CC
protein tyrosine k	MF
response to wate	BP
cellular response	BP
negative regulatio	BP
dorsal root gangli	BP
cellular response	BP
endoplasmic retic	BP
MWP complex	CC
growth cone filop	CC
DNA/DNA anneal	MF
RNA adenylyltran	MF
basic amino acid	BP
response to leuke	BP
sequence-specific	MF
hepatocyte dedifi	BP
C-rich single-stran	MF
cellular response	BP
slow axonal trans	BP
clathrin-uncoating	MF
response to odor	BP
lysosomal matrix	CC

poly(U)-specific e	MF
response to endo	BP
adaptive thermog	BP
Wnt-Frizzled-LRP!	CC
cellular response	BP
cellular response	BP
cellular response	BP
cytoplasmic side	CC
CST complex	CC
rRNA cytidine N-a	MF
H4K20me3 modif	MF
netrin receptor bi	MF
beta-catenin-TCF	CC
Wnt signalosome	CC
response to hypo	BP
response to psycl	BP
sperm head plasn	CC
ooplasm	CC
double-strand bre	BP
PET complex	CC
short-term synap	BP
calcium ion regul	BP
response to amin	BP
RNA N1-methylac	MF
RNA N6-methylac	MF
5.8S rRNA binding	MF
splicing factor bin	MF
peptidyl-aspartic	BP
ATP-dependent n	MF
ubiquitin ligase in	MF
intramanchette t	BP
G-rich single-stra	MF
eosinophil homeo	BP
basophil homeost	BP
xenobiotic detoxi	BP
xenobiotic transp	BP
establishment of	BP
ATP generation fr	BP
modulation by hc	BP
modulation by hc	BP
EMILIN complex	CC
tRNA demethylat	BP
tRNA demethylas	MF
regulation of DN/	BP
negative regulatic	BP

positive regulatio	BP
negative regulatio	BP
negative regulatio	BP
regulation of prot	BP
negative regulatio	BP
positive regulatio	BP
regulation of ada	BP
negative regulatio	BP
positive regulatio	BP
negative regulatio	BP
positive regulatio	BP
regulation of anir	BP
regulation of ster	BP
regulation of ster	BP
negative regulatio	BP
negative regulatio	BP
regulation of G1/	BP
negative regulatio	BP
positive regulatio	BP
regulation of non	BP
negative regulatio	BP
positive regulatio	BP
negative regulatio	BP
regulation of ubic	BP
negative regulatio	BP
positive regulatio	BP
negative regulatio	BP
positive regulatio	BP
regulation of cort	BP
negative regulatio	BP
positive regulatio	BP
regulation of type	BP
negative regulatio	BP
positive regulatio	BP
negative regulatio	BP
positive regulatio	BP
regulation of Wnt	BP
positive regulatio	BP
regulation of smc	BP
negative regulatio	BP
positive regulatio	BP
negative regulatio	BP
positive regulatio	BP
negative regulatio	BP
regulation of mac	BP

positive regulatio	BP
regulation of esta	BP
regulation of cyst	BP
negative regulatio	BP
regulation of sodi	BP
positive regulatio	BP
regulation of rem	BP
regulation of end	BP
negative regulatio	BP
negative regulatio	BP
positive regulatio	BP
negative regulatio	BP
positive regulatio	BP
regulation of cell	BP
negative regulatio	BP
positive regulatio	BP
regulation of ubic	BP
regulation of retr	BP
negative regulatio	BP
positive regulatio	BP
regulation of plar	BP
positive regulatio	BP
negative regulatio	BP
regulation of brar	BP
negative regulatio	BP
positive regulatio	BP
regulation of neu	BP
negative regulatio	BP
positive regulatio	BP
negative regulatio	BP
regulation of prog	BP
positive regulatio	BP
positive regulatio	BP
regulation of fatty	BP
negative regulatio	BP
positive regulatio	BP
regulation of fem	BP
negative regulatio	BP
positive regulatio	BP
regulation of ano	BP
positive regulatio	BP
regulation of glut	BP
negative regulatio	BP
regulation of test	BP
negative regulatio	BP

negative regulatic	BP
negative regulatic	BP
regulation of rRN.	BP
negative regulatic	BP
positive regulatio	BP
regulation of acti	BP
negative regulatic	BP
positive regulatio	BP
negative regulatic	BP
positive regulatio	BP
negative regulatic	BP
positive regulatio	BP
negative regulatic	BP
regulation of bloc	BP
negative regulatic	BP
regulation of fibr	BP
negative regulatic	BP
positive regulatio	BP
negative regulatic	BP
positive regulatio	BP
negative regulatic	BP
positive regulatio	BP
regulation of DN/	BP
negative regulatic	BP
receptor internali	BP
positive regulatio	BP
positive regulatio	BP
regulation of myc	BP
negative regulatic	BP
negative regulatic	BP
regulation of Rho	BP
negative regulatic	BP
regulation of syn:	BP
negative regulatic	BP
positive regulatio	BP
regulation of cera	BP
positive regulatio	BP
negative regulatic	BP
positive regulatio	BP
regulation of NM	BP
regulation of AMI	BP
regulation of kain	BP
regulation of fibr	BP
negative regulatic	BP
negative regulatic	BP

positive regulatio	BP
negative regulatio	BP
positive regulatio	BP
negative regulatio	BP
positive regulatio	BP
negative regulatio	BP
positive regulatio	BP
regulation of tern	BP
positive regulatio	BP
regulation of che	BP
positive regulatio	BP
regulation of che	BP
positive regulatio	BP
positive regulatio	BP
regulation of hep	BP
negative regulatio	BP
positive regulatio	BP
regulation of CD4	BP
negative regulatio	BP
positive regulatio	BP
regulation of end	BP
negative regulatio	BP
positive regulatio	BP
regulation of ova	BP
negative regulatio	BP
negative regulatio	BP
positive regulatio	BP
regulation of binc	BP
negative regulatio	BP
positive regulatio	BP
regulation of acro	BP
positive regulatio	BP
regulation of clatl	BP
positive regulatio	BP
positive regulatio	BP
regulation of oxyg	BP
positive regulatio	BP
regulation of reac	BP
negative regulatio	BP
positive regulatio	BP
regulation of mes	BP
negative regulatio	BP
positive regulatio	BP
negative regulatio	BP
positive regulatio	BP

positive regulatio	BP
positive regulatio	BP
regulation of lam	BP
negative regulatio	BP
positive regulatio	BP
positive regulatio	BP
regulation of lym	BP
negative regulatio	BP
positive regulatio	BP
regulation of T ce	BP
negative regulatio	BP
negative regulatio	BP
regulation of thyr	BP
positive regulatio	BP
positive regulatio	BP
negative regulatio	BP
positive regulatio	BP
regulation of apo	BP
negative regulatio	BP
positive regulatio	BP
regulation of cyto	BP
regulation of prot	BP
negative regulatio	BP
positive regulatio	BP
negative regulatio	BP
positive regulatio	BP
regulation of mac	BP
positive regulatio	BP
regulation of CD8	BP
positive regulatio	BP
regulation of astr	BP
positive regulatio	BP
regulation of glyc	BP
negative regulatio	BP
positive regulatio	BP
negative regulatio	BP
positive regulatio	BP
regulation of opic	BP
positive regulatio	BP
negative regulatio	BP
positive regulatio	BP
negative regulatio	BP
positive regulatio	BP
negative regulatio	BP
positive regulatio	BP

positive regulatio	BP
negative regulati	BP
positive regulatio	BP
positive regulatio	BP
positive regulatio	BP
regulation of CD4	BP
negative regulati	BP
positive regulatio	BP
negative regulati	BP
positive regulatio	BP
negative regulati	BP
positive regulatio	BP
positive regulatio	BP
negative regulati	BP
positive regulatio	BP
regulation of ent	BP
negative regulati	BP
positive regulatio	BP
positive regulatio	BP
positive regulatio	BP
regulation of end	BP
negative regulati	BP
positive regulatio	BP
regulation of den	BP
positive regulatio	BP
negative regulati	BP
positive regulatio	BP
positive regulatio	BP
positive regulatio	BP
negative regulati	BP
positive regulatio	BP
regulation of CD8	BP
negative regulati	BP
positive regulatio	BP
positive regulatio	BP
positive regulatio	BP
positive regulatio	BP
positive regulatio	BP
regulation of mic	BP
negative regulati	BP
positive regulatio	BP
positive regulatio	BP
regulation of plat	BP
negative regulati	BP
regulation of plat	BP

negative regulatio	BP
positive regulatio	BP
negative regulatio	BP
positive regulatio	BP
positive regulatio	BP
positive regulatio	BP
negative regulatio	BP
regulation of thyr	BP
positive regulatio	BP
regulation of thyr	BP
regulation of histo	BP
negative regulatio	BP
positive regulatio	BP
regulation of histo	BP
negative regulatio	BP
positive regulatio	BP
negative regulatio	BP
negative regulatio	BP
positive regulatio	BP
regulation of miR	BP
positive regulatio	BP
negative regulatio	BP
negative regulatio	BP
positive regulatio	BP
regulation of SREI	BP
negative regulatio	BP
positive regulatio	BP
regulation of earl	BP
negative regulatio	BP
positive regulatio	BP
negative regulatio	BP
positive regulatio	BP
negative regulatio	BP
positive regulatio	BP
regulation of sodi	BP
negative regulatio	BP
positive regulatio	BP
regulation of gen	BP
negative regulatio	BP
negative regulatio	BP
regulation of inte	BP
negative regulatio	BP
positive regulatio	BP
negative regulatio	BP
positive regulatio	BP

regulation of mot	BP
negative regulatic	BP
negative regulatic	BP
positive regulatio	BP
regulation of tran	BP
negative regulatic	BP
positive regulatio	BP
regulation of cellu	BP
positive regulatio	BP
negative regulatic	BP
regulation of epit	BP
negative regulatic	BP
negative regulatic	BP
negative regulatic	BP
positive regulatio	BP
negative regulatic	BP
positive regulatio	BP
negative regulatic	BP
positive regulatio	BP
negative regulatic	BP
positive regulatio	BP
positive regulatio	BP
regulation of tern	BP
negative regulatic	BP
regulation of ster	BP
negative regulatic	BP
positive regulatio	BP
regulation of mes	BP
negative regulatic	BP
positive regulatio	BP
positive regulatio	BP
histone H3-T3 ph	BP
positive regulatio	BP
positive regulatio	BP
negative regulatic	BP
positive regulatio	BP
positive regulatio	BP
regulation of cytc	BP
negative regulatic	BP
positive regulatio	BP
positive regulatio	BP
positive regulatio	BP
regulation of cellu	BP
negative regulatic	BP
positive regulatio	BP

histone H3-S10 pl	BP
positive regulatio	BP
regulation of dou	BP
negative regulatio	BP
positive regulatio	BP
regulation of auto	BP
positive regulatio	BP
negative regulatio	BP
regulation of epit	BP
negative regulatio	BP
negative regulatio	BP
negative regulatio	BP
positive regulatio	BP
regulation of syna	BP
negative regulatio	BP
positive regulatio	BP
regulation of bice	BP
negative regulatio	BP
regulation of bark	BP
negative regulatio	BP
regulation of mRN	BP
negative regulatio	BP
negative regulatio	BP
regulation of nucl	BP
negative regulatio	BP
regulation of groo	BP
regulation of beh	BP
positive regulatio	BP
regulation of hea	BP
mitochondrial RN	BP
positive regulatio	BP
positive regulatio	BP
negative regulatio	BP
regulation of cort	BP
positive regulatio	BP
regulation of estr	BP
positive regulatio	BP
regulation of prog	BP
positive regulatio	BP
negative regulatio	BP
regulation of pro-	BP
negative regulatio	BP
positive regulatio	BP
negative regulatio	BP
positive regulatio	BP

negative regulati	BP
negative regulati	BP
negative regulati	BP
positive regulatio	BP
mesenchymal cel	BP
epithelial cell pro	BP
regulation of skel	BP
positive regulatio	BP
positive regulatio	BP
regulation of resp	BP
negative regulati	BP
positive regulatio	BP
regulation of resp	BP
negative regulati	BP
positive regulatio	BP
negative regulati	BP
positive regulatio	BP
regulation of dou	BP
negative regulati	BP
positive regulatio	BP
positive regulatio	BP
regulation of cell	BP
regulation of inte	BP
negative regulati	BP
positive regulatio	BP
positive regulatio	BP
negative regulati	BP
positive regulatio	BP
mannan binding	MF
glycogen binding	MF
starch binding	MF
positive regulatio	BP
regulation of Rho	BP
negative regulati	BP
positive regulatio	BP
positive regulatio	BP
negative regulati	BP
negative regulati	BP
regulation of end	BP
negative regulati	BP
positive regulatio	BP
positive regulatio	BP
regulation of RNA	BP
nicotinate transp	BP
negative regulati	BP

positive regulatio	BP
positive regulatio	BP
negative regulatio	BP
positive regulatio	BP
positive regulatio	BP
positive regulatio	BP
regulation of ATP	BP
negative regulatio	BP
positive regulatio	BP
regulation of histo	BP
positive regulatio	BP
regulation of CD8	BP
negative regulatio	BP
positive regulatio	BP
negative regulatio	BP
positive regulatio	BP
positive regulatio	BP
regulation of den	BP
negative regulatio	BP
positive regulatio	BP
regulation of oste	BP
negative regulatio	BP
positive regulatio	BP
regulation of vasc	BP
negative regulatio	BP
positive regulatio	BP
regulation of neu	BP
negative regulatio	BP
positive regulatio	BP
regulation of chlo	BP
negative regulatio	BP
negative regulatio	BP
regulation of apo	BP
negative regulatio	BP
positive regulatio	BP
regulation of extr	BP
negative regulatio	BP
positive regulatio	BP
regulation of extr	BP
positive regulatio	BP
regulation of intri	BP
negative regulatio	BP
positive regulatio	BP
regulation of pho	BP
negative regulatio	BP

positive regulatio	BP
negative regulatio	BP
positive regulatio	BP
regulation of hist	BP
positive regulatio	BP
regulation of stor	BP
regulation of cati	BP
negative regulatio	BP
positive regulatio	BP
regulation of sem	BP
negative regulatio	BP
positive regulatio	BP
negative regulatio	BP
positive regulatio	BP
positive regulatio	BP
regulation of cave	BP
negative regulatio	BP
positive regulatio	BP
malonyl-CoA cata	BP
malonyl-CoA bios	BP
lipoxin biosynthe	BP
lipoxin A4 metab	BP
lipoxin A4 biosyn	BP
lipoxin B4 biosyn	BP
lysobisphosphati	BP
NA	NA
NA	NA
magnesium ion b	MF
spindle pole	CC
endocytosis	BP
cell migration	BP
chromosome, tel	CC
sterol transport	BP
superoxide-gener	MF

Fig 4A. Expression of interferon associated genes in ancestral SARS-CoV-2 (QLD02) infected epithelial cells relative to uninfected controls (48 hours post-infection).

Donor	Adult_IFIT1	Child_IFIT1	Adult_CXCL10	Child_CXCL10	Adult_ISG15	Child_ISG15
1	NA	0.53004	0.069	0.03409	0.46711	0.11066
2	0.00513	0.33641	0.00025	0.00171	0.01576	0.01282
3	0.01189	0.12405	0.0005	0.00012	0.02069	0.45572
4	0.0108	0.0425	0.00523	0.00853	0.01715	0.03612
5	0.01281	0.42629	0.00026	NA	0.01518	0.182
6	0.10942	0.15472	0.03255	0.02308	0.1931	0.20152
7	NA	0.01317	NA	0.0003	0.23492	0.02203
8	0.06867	0.241	0.02024	NA	0.05671	0.42259
9	-	0.0082	-	0.00027	-	0.01247
10	-	0.01278	-	0.00122	-	0.02334

N=8 adults: 5 females, 3 males and N=10 pediatric: 5 females, 5 males

Fig 3D. Raw data of Gene ontology (GO) analysis of DEGs in adult NECs were displayed by the bar chart (bold). The bars of significantly enriched GO (Overrepresented p value < 0.05) enrichment results were marked in purple and represents the gene count hits (as a percentage over number of genes in a

category	over_repre	under_repr	numDEInC	numInCat	term
GO:007086	0.000188	1	1	1	**negative regulation of protein exit from end**
GO:190130	0.000188	1	1	1	**negative regulation of cargo loading into CO**
GO:003631	0.000378	1	1	2	**SREBP-SCAP complex retention in endoplasr**
GO:003631	0.000536	1	1	3	**cellular response to sterol**
GO:001089	0.00073	1	1	4	**negative regulation of steroid biosynthetic p**
GO:000699	0.000746	1	1	4	**response to sterol depletion**
GO:006036	0.000793	1	1	4	**cranial suture morphogenesis**
GO:003293	0.001618	0.999999	1	9	**SREBP signaling pathway**
GO:004571	0.002138	0.999998	1	11	**negative regulation of fatty acid biosyntheti**
GO:000622	0.002294	0.999998	1	10	**UTP biosynthetic process**
GO:000624	0.002484	0.999998	1	11	**CTP biosynthetic process**
GO:000618	0.002536	0.999998	1	11	**GTP biosynthetic process**
GO:004247	0.003213	0.999996	1	16	**middle ear morphogenesis**
GO:000694	0.003564	0.997388	2	13	regulation of smooth muscle contraction
GO:000616	0.00405	0.999994	1	19	**nucleoside diphosphate phosphorylation**
GO:000664	0.004812	0.999992	1	25	triglyceride metabolic process
GO:001612	0.00589	0.999987	1	30	**sterol biosynthetic process**
GO:004433	0.006598	0.999653	1	2	**canonical Wnt signaling pathway involved ir**
GO:000669	0.006799	0.999983	1	35	**cholesterol biosynthetic process**
GO:004559	0.007863	0.999977	1	42	**negative regulation of fat cell differentiatior**
GO:004247	0.008294	0.999975	1	45	**inner ear morphogenesis**
GO:000018	0.008427	0.997668	2	12	**activation of MAPKKK activity**
GO:006002	0.010602	0.999958	1	57	roof of mouth development
GO:004263	0.012633	0.999941	1	65	cholesterol homeostasis
GO:003286	0.016826	0.999894	1	92	cellular response to insulin stimulus
GO:000820	0.017315	0.999888	1	91	cholesterol metabolic process
GO:200067	0.018956	0.9993	1	4	**negative regulation of type B pancreatic cell**
GO:000820	0.022318	0.999813	1	112	steroid metabolic process
GO:005091	0.024734	0.99977	1	103	detection of chemical stimulus involved in se
GO:000760	0.02749	0.999716	1	119	sensory perception of smell
GO:000803	0.034699	0.999284	1	4	**neuron recognition**
GO:004862	0.035692	0.999466	1	3	**myoblast fate commitment**
GO:004357	0.036513	0.999311	1	4	**maintenance of DNA repeat elements**
GO:190483	0.04127	0.994732	1	29	**beta-catenin-TCF complex assembly**
GO:003027	0.042358	0.997957	1	10	**maintenance of gastrointestinal epithelium**
GO:003202	0.042689	0.993238	2	36	**positive regulation of insulin secretion**
GO:001090	0.045689	0.999466	1	3	**positive regulation of heparan sulfate prote**
GO:000941	0.045893	0.990857	3	48	**response to UV**
GO:001081	0.046589	0.999263	2	4	regulation of hormone levels
GO:005089	0.047561	0.999138	1	226	response to stimulus

GO:00072:	0.048204	0.994036	3	34	**Wnt signaling pathway, calcium modulating**
GO:00015€	0.048965	0.990923	1	50	**blood vessel development**
GO:00194:	0.049025	0.99688	1	17	**triglyceride biosynthetic process**
GO:00468:	0.049568	0.99634	2	19	**positive regulation of protein export from nu**
GO:00353:	0.049856	0.99926	1	4	**positive regulation of hippo signaling**
GO:003101	0.049876	0.996286	2	20	**pancreas development**
GO:19020(	0.049986	0.993594	1	33	**negative regulation of extrinsic apoptotic sig**
GO:003461	0.038192	0.99	1	11	**response to tumor necrosis factor**
GO:00486€	0.044284	0.99	1	7	**regulation of smooth muscle cell proliferatic**
GO:00424(	GO:004244	0.99	1	3	**egulation of hormone metabolic process**
GO:00064€	0.049988	0.900073	1	572	**protein phosphorylation**

ı positive regulation of epithelial to mesenchymal transition

Fig 4B. Levels of CXCL10 (pg/mL) in epithelial cell supernatant at 24 and 48 hours post-infection (h.p.i) with ancestral SARS-CoV-2 (QLD02).

Donor	Adult_24hpi	Kid_24hpi	Adult_48hpi	Kid_48hpi
1	0	0	*45.6713**	32.6
2	0	0	0	0
3	0	0	0	0
4	0	0	0	0
5	0	0	0	18.2
6	0	0	0	40.9306
7	0	0	0	0
8	0	0	0	85.7205
9	-	0	-	0
10	-	0	-	0

Fig 4C. Levels of IFN-beta (pg/ supernatant at 24 and 48 ho (h.p.i) with ancestral SARS-

Donor	Adult_24hp	Kid_24hpi
1	0	66.66
2	9.08	170.57
3	6.66	0
4	0	0
5	*28.15**	123.92
6	0	2.02
7	0	0
8	0	214.24
9	-	0
10	-	213.37

*Note: Outliers were removed using ROUT's test (Q = 1%).

Number of donors shown in Fig 4B&C (N=8 adults: 5 females, 3 males and N=10 children: 5 females,

mL) in epithelial cell
urs post-infection
-CoV-2 (QLD02).

Adult_48hp	Kid_48hpi
0	4.41*
0	0
0	0
0	0
0	0
0	0
0	0
0	0
-	123.23*
-	0

Fig 4D. Levels of IFN-alpha (pg/mL) in epithelial cell supernatant at 24 and 48 hours post-infection (h.p.i) with ancestral SARS-CoV-2 (QLD02).

Donor	Adult_24hp	Kid_24hpi	Adult_48hp	Kid_48hpi
1	NA	308.09	NA	299.98
2	297.82	295.93	296.99	297.93
3	286.4	299.82	300.89	297.65
4	287.57	339.81	294.22	330.69
5	294.31	NA	296.77	NA
6	-	-	-	-
7	-	-	-	-
8	-	-	-	-
9	-	-	-	-
10	-	-	-	-

5 males) and 4D (N=5 adults: 4 females, 1 male and N=5 children: 2 females, 3 males) were different.

Fig 4E. Plaque forming units (PFU) of SARS-CoV-2 from the apical surface of nasal epithelial cells NE(
hours post-infection (h.p.i).

Donor	Adult_QLD02	Child_QLD02	Adult_Delta	Child_Delta	Adult_Omicron
1	400	NA	20000	*4800**	360
2	240	NA	5600	720	NA
3	440	240	40000	1280	*56000**
4	360	160	60000	1360	12000
5	*12000**	320	68000	600	440
6	*2000**	240	28000	520	2000
7	760	200	24000	600	5200
8	160	40	36000	*5200**	NA
9	240	240	920	2800	360
10	360	240	24000	1200	NA

*Note: Outliers were removed using ROUT's test (Q = 1%).

N=10 adults: 5 females, 5 males and N=10 children: 5 females, 5 males

Cs obtained at 24

Child_Omicron
360
160
160
680
520
280
760
120
NA
NA

Fig 4E. Expression of ORF3a RNA in in

Donor	Adult_QLD02	Child_QLD02	Adult_Delta
1	5255.57	333.74	1717274.95
2	9913.15	*6930.29**	683064.15
3	15169.12	35.284	1262284.72
4	772.18	158.162	*6822313.3**
5	11991.24	316.247	2538679.34
6	7900.85	*0.002**	344038.2
7	2904.24	111.385	636203.86
8	550.77	1011.895	1236879.3
9	167.48	1044.655	21226.02
10	1457.2	164.821	792260.34

ıfected cells at relative to GAPDH expression.

Child_Delta	Adult_Omicron	Child_Omicron
*651873.57**	101134.5	NA
7744.09	NA	NA
112300.378	*325984729e+007**	400865.784
15199.927	*736008039e+007**	59349.521
71076.044	NA	486031.319
143943.514	74312.21	791524.651
22925.201	707687.05	553489.244
13945.082	158444.85	594667.521
6516.539	NA	223005.885
50056.603	1690751.82	43188.236

S3 Fig. Relative ACE2 levels compared to GAPDH in pediatric and adult NECs.

Donor	Adult_24h	Child_24h
1	0.199	0.176
2	0.218	0.142
3	0.522	0.091
4	-	0.037
